# Redox control of β_2_-glycoprotein I–von Willebrand factor interaction by thioredoxin-1

**DOI:** 10.1111/j.1538-7836.2010.03944.x

**Published:** 2010-08

**Authors:** F H PASSAM, S RAHGOZAR, M QI, M J RAFTERY, J W H WONG, K TANAKA, Y IOANNOU, J Y ZHANG, R GEMMELL, J C QI, B GIANNAKOPOULOS, W E HUGHES, P J HOGG, S A KRILIS

**Affiliations:** * Department of Immunology, Allergy and Infectious Diseases and Department of Medicine, University of New South Wales, St George HospitalSydney, Australia; † Department of Hematology, St George HospitalSydney, Australia; ‡ Bioanalytical Mass Spectrometry Facility, University of New South WalesSydney, Australia; § UNSW Cancer Research Centre, University of New South WalesSydney, Australia; ¶ The Garvan Institute of Medical ResearchSydney, Australia

**Keywords:** β_2_-glycoprotein I, oxidoreductase, platelet adhesion, thioredoxin, von Willebrand factor

## Abstract

*Background:*β_2_-Glycoprotein I (β_2_GPI) is an abundant plasma protein that is closely linked to blood clotting, as it interacts with various protein and cellular components of the coagulation system. However, the role of β_2_GPI in thrombus formation is unknown. We have recently shown that β_2_GPI is susceptible to reduction by the thiol oxidoreductases thioredoxin-1 and protein disulfide isomerase, and that reduction of β_2_GPI can take place on the platelet surface. *Methods:*β_2_GPI, reduced by thioredoxin-1, was labeled with the selective sulfhydryl probe *N*^a^-(3-maleimidylpropionyl)biocytin and subjected to mass spectrometry to identify the specific cysteines involved in the thiol exchange reaction. Binding assays were used to examine the affinity of reduced β_2_GPI for von Willebrand factor (VWF) and the effect of reduced β2GPI on glycoprotein (GP)Ibα binding to VWF. Platelet adhesion to ristocetin-activated VWF was studied in the presence of reduced β_2_GPI. *Results:* We demonstrate that the Cys288–Cys326 disulfide in domain V of β_2_GPI is the predominant disulfide reduced by thioredoxin-1. Reduced β_2_GPI *in vitro* displays increased binding to VWF that is dependent on disulfide bond formation. β_2_GPI reduced by thioredoxin-1, in comparison with non-reduced β_2_GPI, leads to increased binding of GPIbα to VWF and increased platelet adhesion to activated VWF. *Conclusions:* Given the importance of thiol oxidoreductases in thrombus formation, we provide preliminary evidence that the thiol-dependent interaction of β_2_GPI with VWF may contribute to the redox regulation of platelet adhesion.

## Introduction

β_2_-Glycoprotein I (β_2_GPI) is a circulating plasma protein that consists of five repeating amino acid domains [1,2]. β_2_GPI may play a role in platelet adhesion as, *in vitro*, it interacts with von Willebrand factor (VWF) [3] and the platelet receptors glycoprotein (GP)Ibα and ApoER2 [4,5]. β_2_GPI contains 11 disulfide bonds and no unpaired cysteine. The first four domains include four cysteines each, with disulfide bridges joining the first to the third and the second to the fourth cysteine. The fifth domain has an extra 20 amino acid tail, with an unusual termination in a cysteine, that forms a loop-back disulfide link with an extra cysteine found midway between the standard second and third cysteine positions [1].

We have recently shown that β_2_GPI can be reduced by thioredoxin-1 (TRX-1) and protein disulfide isomerase (PDI) *in vitro* [6,7]. Thiol oxidoreductases are becoming increasingly recognized as important mediators of platelet function [8,9]. The prototype member, PDI, is involved in regulation of activation of the fibrinogen receptor α_IIb_β_3_ [10] and tissue factor [11]. Several novel members of the thiol isomerase family have been recently shown to translocate to the platelet surface following platelet activation [12]. TRX-1 is another member of the superfamily, and has been shown to protect endothelial cells from oxidative stress [13,14]. Extracellular TRX-1 mediates lymphocyte effector function [15] and may regulate platelet adhesion [16]. We have shown that reduction of β_2_GPI is achieved on the platelet surface and endothelial cells [6,7]. In the current study, we show that β_2_GPI is reduced by TRX-1 at Cys288–Cys326 in domain V of β_2_GPI, and that this reduction results in a significantly increased affinity of β_2_GPI for VWF.

## Materials and methods

### Materials

#### Chemicals

Reduced l-glutathione (GSH), apyrase, 1-chloro-2,4-dinitrobenzene (DNCB), α-thrombin, HEPES, dithiothre-itol (DTT), bovine serum albumin (BSA) and human serum albumin (HSA) were from Sigma-Aldrich (St Louis, MO, USA). *N*^a^-(3-maleimidylpropionyl)biocytin (MPB) and NuPAGE 4–12% Bis–Tris gels were from Invitrogen Corporation (Carlsbad, CA, USA). Ristocetin was from Chrono-log (Havertown, PA, USA). NADPH was from Calbiochem-Novabiochem (San Diego, CA, USA). The bicinchoninic acid (BCA) Protein Assay Reagent and *p*-nitrophenyl phosphate (pNPP) substrate were from Pierce (Rockford, IL, USA). Streptavidin 96-well plates were from NUNC (Rochester, NY, USA).

#### Proteins

Native β_2_GPI (nβ_2_GPI) was from Haematologic Technologies (Essex Junction, VT, USA) or was a generous gift of I. Schousboe (University of Copenhagen, Denmark). Recombinant β_2_GPI (rβ_2_GPI), anti-β_2_GPI monoclonal antibody (mAb) (clone 4B2E7) and affinity-purified rabbit polyclonal anti-β_2_GPI were generated in our laboratory as previously described [17,18].

Recombinant human TRX-1 and recombinant GPIba were from R&D (Minneapolis, MN, USA) or American Diagnostica (Stamford, CT, USA). Recombinant rat thioredoxin reductase (TRX-R) was from American Diagnostica. Recombinant human PDI was from Medical & Biological Laboratories (Woburn, MA, USA). VWF was from Calbiochem-Novabiochem.

#### Antibodies

Mouse anti-TRX-1 was from BD Biosciences (Cowley, UK). Mouse anti-human TRX-R was from Santa Cruz Biotechnology (Santa Cruz, CA, USA). Streptavidin–horseradish peroxidase (HRP), rabbit polyclonal anti-mouse HRP and goat polyclonal anti-rabbit HRP antibodies were from Dako (Glostrup, Denmark). Mouse anti-PDI (clone RL90) and mouse anti-VWF were from AbCam (Cambridge, UK). Mouse anti-CD42b was from ABR-Affinity Bioreagents (Golden, CO, USA).

### Methods

#### Structural analysis of β_2_GPI

The structural features of all disulfide bonds in two structures of β_2_GPI (Protein Data Bank ID: 1C1Z and 1QUB) were determined with the disulfide bond analysis tool available at http://www.cancerresearch.unsw.edu.au/CRCWeb.nsf/page/Disulfide+Bond+Analysis.

The secondary structures, in which the cysteines reside, and their solvent accessibility values are from DSSP (http://swift.cmbi.ru.nl/gv/dssp/). The dihedral strain energy of the disulfides was estimated from the magnitude of the five χ angles that constitute the bond [19]. The analysis of the disulfide bonds in β_2_GPI is shown in Table 1.

**Table 1:** Properties of the β_2_-glycoprotein I (β_2_GPI) disulfide bonds in two X-ray structures of the protein [1,2]; analysis of the properties of the disulfide bonds in β_2_GPI was performed as previously described [36,37]

PDB ID	Cys1 residue	Secondary structure	Solvent exposure (∼ Å^2^)	Cys2 residue	Secondary structure	Solvent exposure (∼ Å^2^)	Dihedral strain energy (kJ mol^−1^)	Classification
1C1Z	4	β-Strand	0	47	Irregular	2	11.6	+/−RHSpiral
1C1Z	32	β-Strand	28	60	β-Strand	10	8.2	−LHSpiral
1C1Z	65	Irregular	6	105	β-Strand	2	14.7	−RHSpiral
1C1Z	91	β-Strand	28	118	β-Strand	11	7.9	−LHSpiral
1C1Z	123	β-Strand	0	169	β-Bridge	1	14.5	+/−RHSpiral
1C1Z	155	β-Strand	21	181	β-Strand	10	4.3	−LHSpiral
1C1Z	186	β-Strand	7	229	β-Bridge	0	15.7	−RHSpiral
1C1Z	215	β-Strand	32	241	β-Strand	7	7.4	−LHSpiral
1C1Z	245	β-Bridge	2	296	Irregular	0	9.3	+/−RHSpiral
1C1Z	281	Strand	2	306	Turn	33	18.7	+LHHook
1C1Z	288	Irregular	6	326	Irregular	117	11.1	−/+RHHook
1QUB	4	β-Strand	0	47	Irregular	1	9.2	+/−RHSpiral
1QUB	32	β-Strand	33	60	β-Strand	10	6.8	−LHSpiral
1QUB	65	Irregular	4	105	β-Strand	2	13.6	+/−RHSpiral
1QUB	91	β-Strand	32	118	β-Strand	18	6.3	−LHSpiral
1QUB	123	β-Strand	0	169	β-Bridge	1	10.1	+/−RHSpiral
1QUB	155	β-Strand	24	181	β-Strand	7	7.0	−LHSpiral
1QUB	186	β-Strand	10	229	β-Bridge	1	18.4	+/−RHSpiral
1QUB	215	β-Strand	33	241	β-Strand	8	4.8	−LHSpiral
1QUB	245	β-Bridge	4	296	Irregular	0	7.8	+/−RHSpiral
1QUB	281	β-Strand	1	306	Turn	31	12.2	+/−RHHook
1QUB	288	β-Strand	4	326	Irregular	103	12.2	−/+RHHook

PDB, Protein Data Bank; LH, left-handed; RH, right-handed.

#### Mass spectrometry

To localize the specific cysteine(s) of β_2_GPI that are reduced by TRX-1, nβ_2_GPI was treated with a TRX-1/TRX-R/NADPH mixture and labeled with the selective sulfhydryl reagent MPB, on the basis of a previously described method [20]. Specifically, TRX-1 (5 μm) was reduced by incubation for 1 h at 37 °C with TRX-R (10 nm) and NADPH (200 μm) in a total volume of 300 μL. nβ_2_GPI or rβ_2_GPI was added at a concentration of 0.2 μm to TRX-1/TRX-R/NADPH and incubated for 1 h at 37 °C. To label free thiols, MPB at a concentration of 100 μm was added to β_2_GPI/TRX-1/TRX-R/NADPH and incubated for 10 min at 37 °C. The reaction was quenched by the addition of GSH at a concentration of 200 μm for 10 min at 37 °C. All reactions were performed in 20 mm HEPES buffer containing 0.14 m NaCl (pH 7.4) (HBS).

Samples were separated by sodium dodecylsulfate polyacrylamide gel electrophoresis (SDS-PAGE) (4–12%) under non-reducing conditions, and stained with Coomassie Blue stain. The position of reduced (by TRX-1/TRX-R/NADPH) β_2_GPI ± MPB on Bis–Tris gel after separation by SDS-PAGE has been previously determined [6]. The corresponding bands of β_2_GPI, reduced β_2_GPI and reduced β_2_GPI + MPB were excised from the gel, and β_2_GPI peptides were prepared and analyzed by mass spectrometry and Mascot and X!Tandem searches (the preparation of β_2_GPI peptides and analysis of biotinylation are provided in detail in Data S1).

### Assays for binding of β_2_GPI to immobilized VWF

#### Binding of β_2_GPI reduced by TRX-1/TRX-R/NADPH to immobilized VWF

Ninety-six well microtiter plates were coated with 100 μL of human VWF (10 μg mL^−1^) in 0.1 m NaHCO_3_ (pH 8.3), as previously described [21]. Wells were washed with HBS, and non-specific binding sites were blocked by adding 200 μL of 2% BSA in HBS for 2 h at room temperature; this was followed by washing with HBS. Eight micromolar TRX-1 was incubated with 35 nm TRX-R and 180 μm NADPH for 1 h at 37 °C. Reduced nβ_2_GPI was prepared by incubating 187 μL of nβ_2_GPI (1.8 μm) or HBS with 38 μL of TRX-1/TRX-R/NADPH for 1 h at 37 °C. Reactions were diluted 1 : 1 in HBS, and VWF-coated wells were incubated with 100-μL reaction mixtures for 1 h at room temperature. Wells were washed four times with HBS containing 1 m NaCl, and 100 μL of anti-β_2_GPI mAb (20 μg mL^−1^) was added and incubated for 1 h at room temperature. After washing with HBS, 100 μL of a 1 : 1000 dilution of goat anti-mouse alkaline phosphatase (AP)-conjugated antibody was added and incubated for 1 h at room temperature. Wells were washed with HBS, and 100 μL of pNPP (1 mg mL^−1^) in 1 m diethanolamine buffer and 0.5 mm MgCl_2_ (pH 9.8) was added to each well. In some experiments, free thiols in β_2_GPI/TRX-1/TRX-R/NADPH were blocked by adding MPB (100 μm) for 10 min at 37 °C before addion of the mixture to VWF-coated plates. In other experiments, TRX-R was inhibited, as previously described [22], by incubating 3.5 μm DNCB with TRX-R/NADPH before addition to TRX-1 (the final concentration of DNCB was 0.25 μm per well). The dose response of binding of (reduced and non-reduced) nβ_2_GPI to immobilized VWF (5 μg mL^−1^) was measured by adding β_2_GPI at concentrations between 0.01 and 4 μm.

To assess the effect of TRX-1/TRX-R/NADPH on the capacity of immobilized VWF to bind to β_2_GPI, VWF-coated wells were treated with or without 100 μL of TRX-1/TRX-R/NADPH at concentrations equimolar to that used for reduction of β_2_GPI. Subsequently, β_2_GPI was reduced with TRX-1/TRX-R/NADPH, half of which was subsequently incubated with DNCB (as described above), to inactivate residual activity of TRX-R before it was added to VWF.

#### Binding of recombinant GPIba to immobilized VWF in the presence of reduced nβ_2_GPI vs. non-reduced β_2_GPI

nβ_2_GPI or equimolar BSA was treated with TRX-1/TRX-R/NADPH as above. Fifty-microliter volumes of the reaction mixtures were added to 50 μL of recombinant GPIba (10 μg mL^−1^) and incubated for 1 h at 37 °C. One-hundred-microliter volumes of the reaction mixture were applied to VWF-coated wells. The amount of GPIba bound was determined using 100 μL of 1 μg mL^−1^ anti-CD42b mAb and secondary anti-mouse AP-conjugated antibody (1 : 1000).

### Assays for binding of VWF to immobilized β_2_GPI

#### Binding of VWF in solution, in the presence or absence of ristocetin, to immobilized reduced vs. non-reduced β_2_GPI

Non-reduced and reduced rβ_2_GPI (reduced by TRX-1/TRX-R/NADPH) were coated on ELISA plates at a concentration of 10 μg mL^−1^ for the β_2_GPI component, according to Hulstein *et al.* [3]. One hundred microliters of VWF (10 μg mL^−1^) alone or after preincubation with 1 mg mL^−1^ ristocetin for 5 min at room temperature was added to the wells and incubated for 1 h at room temperature. For some experiments, 3.5 μm DNCB was incubated with VWF or VWF/ristocetin before addition to immobilized β_2_GPI.

To study the effect of TRX-1/TRX-R/NADPH on VWF in solution, VWF (10 μg mL^−1^) was incubated with or without TRX-1/TRX-R/NADPH as above; half of the VWF mixture was subsequently activated with ristocetin. These VWF mixtures were then added to wells coated with β_2_GPI. The amount of bound VWF was assessed with the use of 100 μL of anti-VWF mAb (5 μg mL^−1^) and secondary anti-mouse AP-conjugated antibody (1 : 1000).

All enzyme-linked immunosorbent assay (ELISA) incubations were performed under argon, as exposure of reduced β_2_GPI to air led to its gradual reoxidation [7].

The optical density (OD) for all binding assays was read at 405 nm with a Microplate Scanning Spectrophotometer (Bio-Tek Instruments, Winooski, VT, USA).

#### Detection of TRX-1 and TRX-R in platelet lysates

Washed platelets from healthy donors were prepared as previously described [20,23]. A platelet suspension, 4 × 10^11^ L^−1^ in 20 mm HEPES, 137 mm NaCl, 4 mm KCl, 0.5 mm Na_2_HPO_4_ and 0.1 mm CaCl_2_ (pH 7.4) was activated with thrombin (100 nm) at 37 °C for 10 min. Platelets were centrifuged at 2000 ×*g* for 20 min at 4 °C to isolate the platelet releasate, according to Burgess *et al.* [20]. The pellet was washed twice with phosphate-buffered saline, and lysed with lysis buffer NP40 containing 10% of a cocktail of proteinase inhibitors [4-(2-aminoethyl)benzenesulfonyl fluoride, pepstatin A, E-64, bestatin, leupeptin and aprotinin]. The platelet lysate was obtained by centrifugation at 2000 ×*g* for 20 min at 4 °C. The protein concentration was calculated with the microBCA assay. Equal amounts of platelet lysate and releasate were subjected to 4–12% Bis–Tris NuPage gel electrophoresis. Proteins were transferred to poly(vinylidene difluoride) membranes, and TRX-1 and TRX-R were detected with mouse anti-human TRX-1 (1 : 500) and mouse anti-human TRX-R (1 : 500) antibodies. Anti-mouse HRP-conjugated (1 : 2000) antibody was used as secondary antibody.

#### Platelet adhesion to reduced β_2_GPI coated on microtiter wells in the presence of VWF

The method is based on that reported by Lahav *et al.* [8]. Washed platelets in Jamieson buffer were pelleted and resuspended in platelet adhesion buffer (Tris-buffered saline containing 0.05 m Tris-HCl, 140 mm NaCl, 2 mm Mg^2+^ and 0.1% BSA, pH 7.4) at 12 × 10^11^ L^−1^. Ninety-six-well plates were coated with non-reduced or reduced rβ_2_GPI (reduced by TRX-1/TRX-R/NADPH), washed three times with HBS, and incubated with VWF in the presence of ristocetin (1 mg mL^−1^), as described above. Platelets in adhesion buffer with or without TRX-1/TRX-R/NADPH or DNCB were aliquoted into the corresponding (± TRX-1/TRX-R/NADPH) wells and incubated at room temperature for 1 h. After washing with phosphate-buffered saline (five times), platelets that had adhered to the wells were visualized with a Leica DM IRB (Leica, Wetzlar, Germany) microscope. Images were obtained with a Leica DC200 (Leica) camera (× 10 magnification). The number of adherent platelets was quantified by adding lysis buffer [0.07 m trisodium citrate, 0.3 m citric acid, 5 mm pNPP and 0.1% (v/v) Triton X-100, pH 5.4] for 1 h. The reaction was terminated by addition of 2 m NaOH, and absorbance was read at 405 nm.

### Statistical analysis

The spss 17.0 statistics software (SPSS Inc., Chicago, IL, USA) was used for the analysis of data. Data were analyzed by anova followed by Dunnett’s correction for multiple comparisons. A *P*-value of < 0.05 was considered to be statistically significant.

## Results

### Identification of a free thiol in Cys326 of β_2_GPI after reaction with TRX-1

Mass spectrometry showed biotinylation of nβ_2_GPI treated with TRX-1/TRX-R/NADPH/MPB (see Tables S1 and S2 for a full list of peptides for each sample). As a number of cysteines were found to be biotinylated in β_2_GPI, the MBP/iodoacetamide-labeled peptide ratio was used to determine the cysteine target of reduced TRX-1. As shown in Table S3, Cys326 is by far the most heavily modified cysteine in the protein.

### Structural analysis of disulfide bond Cys288–Cys326 of the β_2_GPI molecule

Nine of the 11 disulfides in β2GPI have a spiral configuration (RHS, right-handed spiral or LHS, left-handed spiral), the most common configuration and one associated with structural disulfides (Table 1). The Cys288–Cys326 disulfide has a −/+ right-handed hook (−/+RHHook) configuration in both crystal structures of the protein [1,2]. None of the bonds are particularly strained based on the measure of the dihedral strain energy. Notably, Cys326 is particularly exposed to solvent. This exposure is likely a factor in the reduction of the Cys288-Cys326 disulfide bond by protein oxidoreductases.

### Redox control of the interaction of β_2_GPI with VWF

#### Assays for binding of β_2_GPI to immobilized VWF

**Fig. 1:**
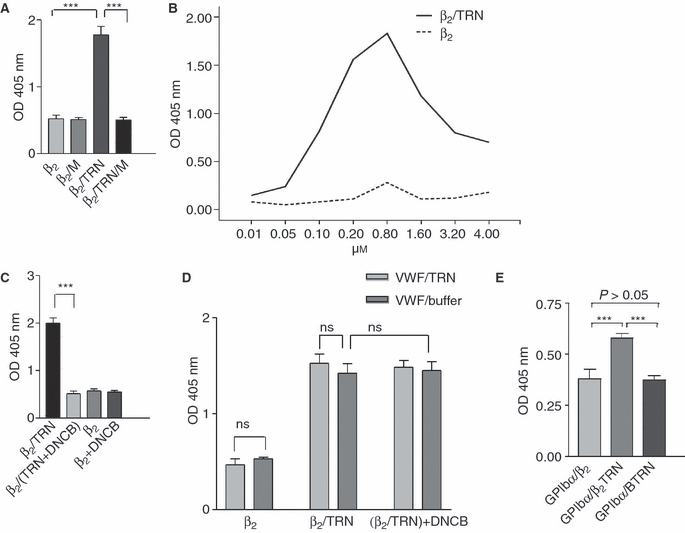
Assays for binding of reduced vs. non-reduced β_2_-glycoprotein I (β_2_GPI) to immobilized von Willebrand factor (VWF). Thiol dependence of the β_2_GPI–VWF interaction. Reduced β_2_GPI promotes binding of glycoprotein (GP)Ibα to VWF. (A) Increased binding of reduced native β_2_GPI (nβ_2_GPI) to VWF; inhibition by the thiol blocker *N*^a^-(3-maleimidylpropionyl)biocytin (MPB). Plates were coated with VWF, and β_2_GPI mixtures were applied to the wells and incubated for 1 h at room temperature. The amount of bound β_2_GPI was assessed with an anti-β_2_GPI monoclonal antibody (mAb). β_2_GPI reduced by thioredoxin-1 (TRX-1)/thioredoxin reductase (TRX-R)/NADPH showed a significantly higher level of binding to VWF than non-reduced β_2_GPI. The increased binding of reduced β_2_GPI to VWF was inhibited when free thiols introduced into β_2_GPI by TRX-1/TRX-R/NADPH were previously blocked with MPB. (B) Dose–response curves of nβ_2_GPI (reduced and non-reduced) binding to immobilized VWF. The amount of bound β_2_GPI was assessed with an anti-β_2_GPI mAb. Maximal binding for β_2_GPI reduced by TRX-1/TRX-R/NADPH is at 0.8 μm (*n* = 2). (C) Inhibition of reduced nβ_2_GPI binding to VWF by the TRX-R inhibitor 1-chloro-2,4-dinitrobenzene (DNCB). Plates were coated with VWF and blocked. TRX-R activity was inhibited by DNCB before incubation with TRX-1. β_2_GPI was treated with the inactive TRX-1 preparations and applied to the wells. The amount of β_2_GPI bound to VWF was significantly reduced when TRX-R had been inactivated with DNCB. (D) The increased binding of reduced nβ_2_GPI to VWF is mediated through the effect of the TRX-1 reducing mixture on β_2_GPI and not on VWF. Plates were coated with VWF and blocked. VWF-coated wells were then treated with TRX-1/TRX-R/NADPH or buffer alone. Separately, nβ_2_GPI was incubated with TRX-1/TRX-R/NADPH, and half of the mixture was incubated with DNCB to inactivate residual TRX-R activity before addition of the β_2_GPI preparations to the wells. Binding of β_2_GPI was determined with an anti-β_2_GPI mAb. The binding of reduced β_2_GPI to VWF was not affected by pretreatment of the coated VWF with TRX-1/TRX-R/NADPH before addition of the β_2_GPI mixtures. Also, inhibition of the residual TRX-R activity contained in the β_2_GPI/TRX-1/TRX-R/NADPH mixture before addition to coated VWF did not alter the binding of already reduced β_2_GPI to VWF (whether or not VWF was exposed to active TRX-R or DNCB-inactivated TRX-R). (E) Binding of recombinant GPIba to immobilized VWF in the presence of reduced nβ_2_GPI. Plates were coated with VWF and blocked. β_2_GPI with or without TRX-1/TRX-R/NADPH treatment and bovine serum albumin treated with TRX-1/TRX-R/NADPH as control were incubated with GPIba. The reaction mixtures were then applied to the wells and incubated. The amount of GPIba bound was determined with a specific anti-GPIba mAb, and was increased in the presence of reduced β_2_GPI. OD, optical density; β_2_, β_2_GPI; M, MPB; TRN, TRX-1/TRX-R/NADPH; B, bovine serum albumin; NS, not significant. For all enzyme-linked immunosorbent assays, values are the mean ± standard error of the mean (*n* = 3). ***P < 0.001.

β_2_GPI reduced by TRX-1 shows increased binding to immobilized VWF. Given the importance of thiol linkage in VWF multimerization [24] and its previously described ability to bind β_2_GPI [3], we proceeded to examine whether free thiols generated in β_2_GPI are involved in its interaction with VWF. We applied β_2_GPI reduced by TRX-1/TRX-R/NADPH to VWF-coated ELISA plates, and detected the amount of β_2_GPI bound to VWF with the mAb 4B2E7. The binding of β_2_GPI treated with TRX-1/TRX-R/NADPH to immobilized VWF was increased 3.5-fold when compared with untreated β_2_GPI (Fig. 1A). Non-reduced β_2_GPI displayed low binding to VWF, regardless of the concentrations used, whereas binding of reduced β_2_GPI to VWF showed a ‘bell-shaped’ curve with maximal binding at 0.8 μm (Fig. 1B) and decreased binding at higher concentrations. This can be attributed to the ‘high-dose hook effect’ described for high concentration of some ligands in ELISA systems [25]. For a monotonic increase in signal with concentration, there must be an excess of antibodies, both capture and enzyme-conjugated, relative to the analyte being detected. As the concentration of analyte (in this case, β_2_GPI) begins to exceed the amount of antibody, the dose–response curve will plateau and, with further increase, may, paradoxically, become negatively sloped. In some cases, this phenomenon has also been attributed to the coating density of the capture antibody (the capture protein in this case being VWF).

#### Thiol dependence of reduced β_2_GPI binding to VWF

The increased binding of reduced β_2_GPI to immobilized VWF was abrogated when the thiol-reactive molecule MPB was added to reduced β_2_GPI (reduced by TRX-1/TRX-R/NADPH) before addition to VWF-coated wells. This indicated that the binding of reduced β_2_GPI to VWF was dependent on disulfide bond formation between the two molecules, which was prevented in the presence of MPB (Fig. 1A).

Inhibition of TRX-R activity by DNCB (before the inclusion of TRX-R in the TRX-1/TRX-R/NADPH mixture used to reduce β_2_GPI) decreased the binding of β_2_GPI treated with TRX-1/TRX-R/NADPH to immobilized VWF, and the binding was comparable to that of untreated β_2_GPI (Fig. 1C).

#### Reduction of VWF by TRX-1 is not necessary for interaction with β_2_GPI

There is evidence from the literature that exposure of VWF to TRX-1/DTT reduces disulfide bonds in the molecule, decreasing the binding affinity of collagen [21]. To exclude the effect of TRX-1/TRX-R/NADPH on vWF in our experiments, we used two different approaches. First, we treated VWF coated on the wells with TRX-1/TRX-R/NADPH or buffer alone, and compared binding affinities for β_2_GPI. Treatment of VWF with the reducing mixture before addition of β_2_GPI did not affect β_2_GPI binding: non-reduced β_2_GPI showed low binding to VWF, whether or not VWF had been pretreated with TRX-1/TRX-R/NADPH. Also, reduced β_2_GPI showed a high level of binding to VWF, whether or not VWF had been pretreated with TRX-1/TRX-R/NADPH (Fig. 1D).

In the second approach, we utilized the TRX-R inhibitor DNCB to inactivate the residual reducing activity of TRX-1/TRX-R/NADPH after it had already reduced β_2_GPI. When DNCB had been incubated with TRX-1/TRX-R/NADPH before it was used to reduce β_2_GPI, β_2_GPI could not be reduced, and therefore the mixture of β_2_GPI and inactive TRX-1/TRX-R/NADPH showed the same low level of binding to VWF as β_2_GPI alone (Fig. 1C). However, when DNCB was added to TRX-1/TRX-R/NADPH after it had already reduced β_2_GPI, β_2_GPI/inactivated TRX-1/TRX-R/NADPH showed the same high level of binding as β_2_GPI/active TRX-1/TRX-R/NADPH, thus showing that the critical event in VWF–β_2_GPI binding is the reduction of β_2_GPI (Fig. 1D).

#### β_2_GPI reduced by TRX-1 promotes binding of GPIba to immobilized VWF

The presence of reduced β_2_GPI (reduced by TRX-1/TRX-R/NADPH) increased the binding of GPIba to immobilized VWF in comparison with untreated β_2_GPI or BSA treated with TRX-1/TRX-R/NADPH as control protein (Fig. 1E).

#### Assays for binding of VWF to immobilized β_2_GPI

##### Dependence of VWF binding to reduced β_2_GPI on ristocetin activation of VWF

**Fig. 2:**
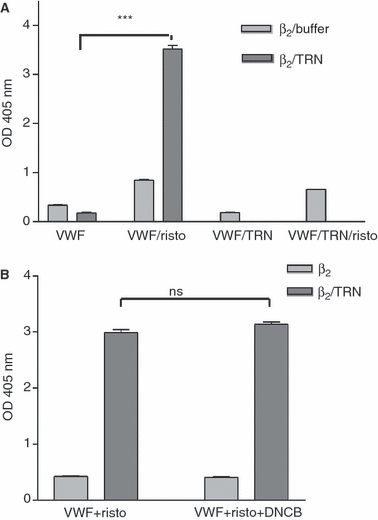
Assays for binding of von Willebrand factor (VWF) in solution to immobilized β_2_-glycoprotein I (β_2_GPI). Ristocetin-activated VWF has increased affinity for reduced β_2_GPI. Dependence of VWF–β_2_GPI binding on the reduction state of β_2_GPI. (A) Ristocetin activation of VWF increases binding to immobilized reduced recombinant β_2_GPI (rβ_2_GPI). Non-reduced and reduced rβ_2_GPI [reduced by thioredoxin-1 (TRX-1)/thioredoxin reductase (TRX-R)/NADPH] were coated on enzyme-linked immunosorbent assay (ELISA) plates (under argon) at a concentration of 10 μg mL^−1^ for the β_2_GPI component. After washing, VWF (10 μg mL^−1^) was added alone or after incubation with TRX-1/TRX-R/NADPH (at the same concentration and with the same method as used for the reduction of β_2_GPI). Half of the VWF solution was further incubated with ristocetin before addition to the wells. The amount of bound VWF was assessed with an anti-VWF monoclonal antibody. Ristocetin-activated VWF showed a marked increase in binding to reduced β_2_GPI as compared with non-activated VWF.Treatment of VWF in solution with the reducing mixture TRX-1/TRX-R/NADPH did not affect its binding to β_2_GPI in the presence or absence of ristocetin. (B) Binding of VWF to coated β_2_GPI in the presence of 1-chloro-2,4-dinitrobenzene (DNCB). Reduced and non-reduced β_2_GPI (reduced by TRX-1/TRX-R/NADPH) was coated onto 96-well plates under argon. Preparations of VWF or VWF activated by ristocetin were prepared, and half of these were incubated with DNCB. The VWF mixtures were added to the wells and incubated for 1 h at room temperature. Detection with an anti-VWF antibody showed that the presence of DNCB did not affect the binding of VWF to already reduced β_2_GPI on the plate. OD, optical density; β_2_, β_2_GPI; TRN, TRX-1/TRX-R/NADPH; risto, ristocetin; NS, not significant. For all ELISAs, values are the mean ± standard error of the mean (*n* = 3). ***P < 0.001.

Ristocetin-activated VWF bound more than non-activated VWF to coated, non-reduced β_2_GPI. Furthermore, there was a significant increase in the binding of ristocetin-activated VWF to coated, reduced β_2_GPI as compared with non-activated VWF (Fig. 2A). This shows that the affinity of reduced β_2_GPI for VWF is high when VWF is activated either by coating on microtiter wells (Fig. 1A) or being treated in solution with ristocetin (Fig. 2A). Incubating TRX-1/TRX-R/NADPH with VWF in solution did not affect its capacity to bind to β_2_GPI (Fig. 2A).

The binding of VWF (activated or non-activated by ristocetin) to immobilized (reduced or non-reduced) β_2_GPI was not affected by the presence or not of DNCB in the VWF solution (Fig. 2B).

### Potential role of reduced β_2_GPI and TRX-1 in platelet adhesion

#### TRX-1 and TRX-R are detected by western blot in platelet lysates

**Fig. 3:**
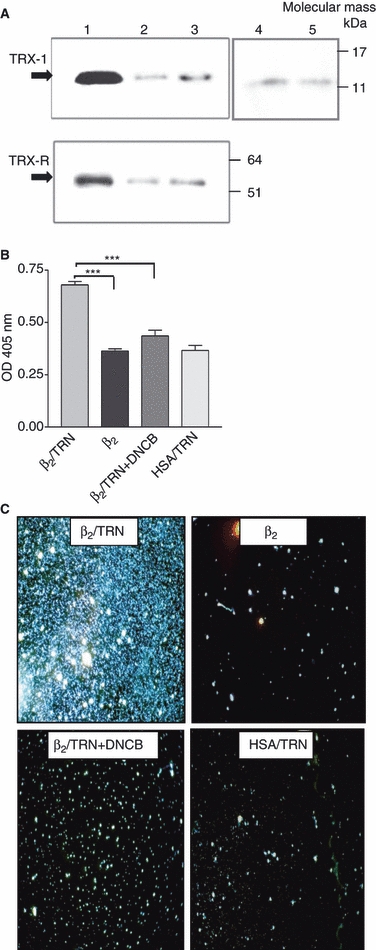
Detection of thioredoxin-1 (TRX-1) and thioredoxin reductase (TRX-R) in platelets. β_2_-glycoprotein I (β_2_GPI) reduced by TRX-1 increases platelet adhesion to activated von Willebrand factor (VWF). (A) Platelets as a source of TRX-1 and TRX-R. Western blotting of TRX-1 under reducing conditions in lysates of resting (lane 2) and thrombin-activated (lane 3) platelets, and releasates of resting (lane 4) and thrombin-activated (lane 5) platelets. Detection of TRX-R was confirmed on non-reduced proteins of platelet lysate of thrombin-activated platelets (lane 2) and resting platelets (lane 3). Lane 1 shows the band for recombinant human TRX-1 (400 ng) and recombinant rat TRX-R (250 ng), respectively. Platelet lysates (1.5 μg) were loaded in lanes 2 and 3, and platelet releasate (5 μg) in lanes 4 and 5. (B) Platelet adhesion to reduced β_2_GPI is enhanced in the presence of activated VWF. Recombinant β_2_GPI (rβ_2_GPI) was treated or not treated with TRX-1/TRX-R/NADPH and coated onto 96-well plates. After blocking, wells were incubated with VWF activated with ristocetin. Platelets in adhesion buffer with or without TRX-1/TRX-R/NADPH or 1-chloro-2,4-dinitrobenzene (DNCB) were aliquoted into the corresponding (± TRX-1/TRX-R/NADPH) wells and incubated at room temperature for 1 h. After extensive washing with phosphate-buffered saline, platelets that had adhered were quantified with lysis buffer for 1 h. The reaction was terminated by the addition of 2 m NaOH, and absorbance was read at 405 nm. OD, optical density; β_2_, β_2_GPI; TRN, TRX-1/TRX-R/NADPH; HSA, human serum albumin. Values are the mean ± standard error of the mean (*n* = 3, in triplicate). ***P < 0.001. (C) Representative images of platelet adhesion obtained with a Leica DC200 camera at × 10 magnification. β_2_GPI reduced by TRX-1/TRX-R/NADPH significantly enhanced platelet adhesion to vWF activated by ristocetin, whereas non-reduced β_2_GPI or TRX-1/TRX-R/NADPH-treated HSA did not. Platelet adhesion was partially inhibited when TRX-1/TRX-R/NADPH was treated with DNCB.

As VWF tethers platelets, we were interested to determine whether the source of β_2_GPI’s reducing agent, TRX-1, can be found in platelets. Both TRX-1 and TRX-R were detected in platelet lysates of resting and thrombin-activated platelets. TRX-1 was also detected in the releasates from resting and thrombin-activated platelets (Fig. 3A).

#### β_2_GPI reduced by TRX-1 increases platelet adhesion to activated VWF

The VWF–platelet GPIbα receptor interaction is important for the initial step of platelet adhesion. Ristocetin promotes VWF binding to GPIbα in solution. We demonstrated that ristocetin-activated VWF had a greater affinity for reduced β_2_GPI (Fig. 2A), so we tested whether reduced β_2_GPI could support platelet adhesion. The method was based on one previously published, which uses an ELISA to measure acid phosphatase in adherent platelets [8]. In this system, reduced β_2_GPI caused a significant increase, by 41%, in platelet adhesion as compared with non-reduced β_2_GPI when VWF was activated with ristocetin (OD 0.68 ± 0.04 vs. 0.40 ± 0.02, respectively, mean ± standard deviation, *n* = 3 in triplicate, *P* < 0.0001). Platelet adhesion was partially inhibited by addition of the TRX-R inhibitor DNCB, supporting the notion that reduction of β_2_GPI by TRX-1 was partially responsible for the adhesion of the platelets (Fig. 3B). Representative images of the platelet adhesion assay are shown in Fig. 3C. Platelets displayed low adhesion to VWF not treated with ristocetin, with no differences being seen between coated β_2_GPI, HSA, TRX-1-treated β_2_GPI or TRX-1-treated HSA (data not shown).

## Discussion

In the current study, we have shown that β_2_GPI reduced by TRX-1 demonstrates thiol-dependent increased binding to VWF and platelet adhesion to activated VWF. TRX-1 is ubiquitously expressed and is secreted to the cell surface [26]. TRX-1 is also present in platelet lysates [27] and releasates. We have previously shown that β_2_GPI is partially reduced on the platelet surface by TRX-1/TRX-R/NADPH [6]. We now demonstrate that the predominant cysteine reduced by TRX-1 is Cys326 in domain V. This finding is in agreement with the majority of biological functions of β_2_GPI being attributed to domain V including phospholipid [1], thrombin [28] and GPIbα binding [4,5], and FXIa cleavage [29]. The fifth domain is predicted to be anchored to the plasma membrane, providing the appropriate interface to react with cell surface proteins such as platelet oxidoreductases.

It is interesting that β_2_GPI has been shown to bind both with GPIba and VWF [3–5]. Hulstein *et al.* [3] showed that β_2_GPI bound to the A1 domain of VWF with low affinity and inhibited platelet adhesion to immobilized VWF. However, when free thiols are introduced into β_2_GPI by TRX-1, the binding affinity for VWF increases significantly and promotes adhesion of GPIbα and platelets to activated VWF. The GPIba–VWF interaction is crucial for hemostasis. Disulfide exchange may be an important feature of platelet tethering to exposed VWF, as shear has been shown to promote disulfide formation between VWF subunits and VWF binding to platelets [24]. Furthermore, GPIbα shows physical proximity to PDI on the platelet surface [20]. Platelets may also provide the surface for reduction of β_2_GPI, which could be a modifier of disulfide reactions of VWF and/or GPIbα.

Multiple interactions of β_2_GPI have been shown with various components of the coagulation and fibrinolysis system *in vitro*, often with conflicting results and interpretations. Although β_2_GPI deficiency does not lead to gross hemostatic abnormalities, we believe that reduction of circulating β_2_GPI can promote thrombus formation under specific conditions. This idea is supported by the facts that β_2_GPI^−/−^ mice have impaired thrombin generation [30] and that β_2_GPI inhibits thrombin inactivation by heparin cofactor II [31]. Our laboratory has shown that β_2_GPI is predominantly in the reduced form *in vivo* [7]. Hence, it is possible that *in vivo* reduced β_2_GPI supports platelet adhesion, a function not apparent in *in vitro* studies, where the purified protein has been significantly oxidized. The design of *in vivo* studies would delineate the role of reduced vs. non-reduced β_2_GPI in platelet adhesion.

The presence of the reduced form of β_2_GPI in the circulation may be relevant to disease development. There are a number of publications addressing the relationship of β_2_GPI levels to thrombotic and atherothrombotic disease, some in the context of antiphospholipid syndrome. In a review by Inanc *et al.* [32], the levels of circulating β_2_GPI was not associated with thrombotic risk. High levels of circulating β_2_GPI have been reported to decrease the risk of myocardial infarction in elderly men [33]. With regard to the risk of thrombosis, the amount of reduced β_2_GPI in the circulation may be more important than the total levels of circulating β_2_GPI.

The finding for the first time that β_2_GPI can be involved in thiol exchange reactions with TRX-1 is of considerable importance, given β_2_GPI’s high concentration in plasma makes it easily available for reactions where thiols are needed. There is increased scientific interest in the role of thiol-acting enzymes such as PDI in thrombus formation. PDI has been shown *in vivo* to be required for thrombus formation [34] and tissue factor activation [35]. Inhibition of PDI action by an inhibitory antibody or the thiol blocker bacitracin abrogated thrombus formation. Although the substrate of PDI has not been definitely established, existing data support integrin α_IIb_β_3_ or GPIba. The demonstration that β_2_GPI is a substrate of TRX-1 and PDI has implications for a role of β_2_GPI in thrombus formation. Our results also provide considerable insight into the participation of extracellular TRX-1 in the regulation of platelet adhesion.

## Addendum

F. H. Passam., S. Rahgozar and M. Qi performed experiments and wrote the manuscript; K. Tanaka and J. C. Qi prepared recombinant β_2_GPI and anti-β_2_GPI antibodies; M. J. Raftery, J. W. H. Wong and P. J. Hogg performed the mass spectrometry and the disulfide bond structural analysis; R. Gemmell and W. E. Hughes provided experimental materials and analytic tools; B. Giannakopoulos, Y. Ioannou and J. Y. Zhang were involved in the interpretation of data and revision of the manuscript; P. J. Hogg and S. A. Krilis made important intellectual contributions; and S. A. Krilis provided the scientific hypothesis, interpreted the data, revised the manuscript and coordinated the whole study.

## Supporting Information

**Data S1.** Reduction of β_2_GPI by TRX-1 for mass spectrometry.

**Table S1.** Results from X!Tandem search for tryptic peptides produced from the three samples: nB2GPI, nB2GPI/TRX-1/TRX-R/NADPH and nB2GPI/TRX-1/TRX-R/NADPH + MPB.

**Table S2.** Results from Mascot search for tryptic peptides produced from the three samples: nB2GPI (2A), nB2GPI/TRX-1/TRX-R/NADPH+MPB (2B) and nB2GPI/TRX-1/TRX-R/NADPH (2C).

**Table S3.** MPB/IA labeled abundance ratios of cysteine containing peptides in nβ2GPI treated with TRX-1/TRX-R/NADPH/MPB.
