# Supplementary material for: Redox control of β2-glycoprotein I–von Willebrand factor interaction by thioredoxin-1
Source: J Thromb Haemost. 2010 Aug;8(8):1754–62. doi: 10.1111/j.1538-7836.2010.03944.x (PMC3017748; doi:10.1111/j.1538-7836.2010.03944.x)
Supplement: Supplementary file 1 [file jth0008-1754-SD1.doc]

**JOURNAL OF THROMBOSIS AND HAEMOSTASIS**

**SUPPLEMENTARY INFORMATION**

**Title:**

**Redox control of β2-glycoprotein I-von Willebrand factor interaction by thioredoxin-1.**

**Authors:**

F.H. Passam,*^1^ S. Rahgozar,*^1^ M. Qi,* M.J Raftery, ‡ J.W.H. Wong,§ K. Tanaka,* Y. Ioannou,* J.Y. Zhang,* R. Gemmell,† J.C. Qi,* B. Giannakopoulos,* W.E. Hughes, ¶ P.J. Hogg, § and S.A. Krilis *

^1^ equal contribution to the manuscript

*Reduction of β2GPI by TRX-1 for mass spectrometry*

All reactions were performed in 20 mM HEPES buffer containing 0.14 M NaCl, pH 7.4 (HBS) and are based on the method described by Burgess *et al.* [1]

TRX-1 (5 µM) was reduced by incubation for 1 h at 37ºC with TRX-R (10 nM) and NADPH (200 µM) in a total volume of 300 µl HBS. Native (n) β2GPI or recombinant (r) β2GPI were individually added at a concentration of 0.2 µM to the TRX-1/TRX-R/NADPH mixture and incubated for 1 h at 37ºC.

To label free thiols, MPB at a concentration of 100 µM was added to the β2GPI/TRX-1/TRX-R/NADPH and incubated for 10 min at 37ºC. The reaction was quenched by the addition of glutathione (GSH) at a concentration of 200 µM for 10 min at 37ºC.

Samples were separated by SDS-PAGE (4-12%) under non-reducing conditions and

stained with Coomassie Blue stain. Bands were excised and subjected to mass spectrometry.

**Mass spectrometry**

In order to determine the cysteine residue(s) in the β2GPI molecule involved in the thiol exchange reactions mass spectrometry was performed on nβ2GPI treated with TRX- 1/TRX-R/NADPH±MPB (as described above) separated by SDS-PAGE and stained with Coomassie Blue stain. The bands were excised, destained with NH_4_HCO_3_ (25 mM, 50% CH_3_CN) for 90 min, reduced with DTT (10 mM, 37ºC, 30 min) treated with iodoacetamide (20 mM, 37ºC, 30 min) then washed with acetonitrile (x 3 with 100 μl). The samples were rehydrated with NH_4_HCO_3_ (30 μl, 10 mM) containing trypsin (5 ng/μl) and left overnight at 37ºC.

Digested peptides were separated by nano-LC using an Ultimate 3000 HPLC and autosampler system (Dionex, Amsterdam, Netherlands). Samples (5 µl) were concentrated and desalted onto a micro C18 precolumn (500 µm x 2 mm, Michrom Bioresources, Auburn, CA) with H_2_O:CH_3_CN (98:2, 0.05 % TFA) at 20 µl/min. After a 4 min wash the pre-column was switched (Valco 10 port valve, Dionex) into line with a fritless nano column (75µ x ~10cm) containing C18 media (5µ, 200 Å Magic, Michrom) manufactured according to Gatlin [2]. Peptides were eluted using a linear gradient of H_2_O:CH_3_CN (98:2, 0.1 % formic acid) to H_2_O:CH_3_CN (64:36, 0.1 % formic acid) at 250 nl/min over 60 min. High voltage (1800 V) was applied to low volume tee (Upchurch Scientific) and the column tip positioned ~ 0.5 cm from the heated capillary (T=200°C) of a LTQ FT Ultra (Thermo Electron, Bremen, Germany) mass spectrometer. Positive ions were generated by electrospray and the LTQ FT Ultra operated in data dependent acquisition mode (DDA) [3].

A survey scan m/z 350-1750 was acquired in the FT ICR cell (Resolution = 100,000 at m/z 400, with an accumulation target value of 1,000,000 ions). Up to the 6 most abundant ions (>2500 counts) with charge states of +2, +3 or +4 were sequentially isolated and fragmented within the linear ion trap using collisionally induced dissociation with an activation q = 0.25 and activation time of 30 ms at a target value of 30,000 ions. M/z ratios selected for MS/ MS were dynamically excluded for 30 seconds.

Mass spectral data were searched using Mascot (V2.2, Matrix Science) or converted to MzXML file format using ReAdW (version 4.0.2) [4] using default parameters, and submitted to the database search program X!Tandem (Release 2008.12.01) [5]. Search parameters were: Precursor tolerance 10 ppm and product ion tolerances ± 0.4 Da. For the first search stage of Mascot and X!Tandem, Met-O, Cys-carboxyamidomethyl, Cys-MPB, Cys-MPB+O, Cys-MPB+H_2_O and Cys-MPB+H_2_O_2_ were specified as variable modifications with full tryptic cleavage and up to 1 missed cleavage. For the X!Tandem refinement stages, additional Met-2O, Trp-O, Trp-2O, Glu/Gln-deamination were specified as variable modifications with semi-tryptic cleavage and up to 3 missed cleavages. The acceptance threshold selected was log(e) value of < -1 for both peptides and proteins. All searches were performed against the non redundant database from NCBI (13^th^ January 2009) concatenated with the reverse compliment of the same database to determine false discovery rate ***(Supplementary Tables 1 and 2).***

To determine the extent of biotinylation of cysteine residues in β2GPI, the ion abundance ratio of Cys-carboxyamidomethyl and Cys-MPB+H_2_O_2_ was used. The other modifications of MPB represented a minority in comparison to the MPB+ H_2_O_2_ modification. The ratio was calculated for all cysteine containing peptides where there was evidence of both carboxylamidomethylation and succinimidyl biotinylation present based on results from X!Tandem. To calculate ion abundance of peptides, extracted ion chromatograms (XIC) were generated using the XCalibur Qual Browser software (version 2.0.7, Thermo). For each peptide, the mono-isotopic 2+ and 3+ ion masses were used to generate the XIC. A mass tolerance of ±0.01 Da was permitted for each ion. The area was calculated using the automated peak detection function built into the software. For each peptide ion, the correct peak was verified by manually cross-referencing with the scan number of the peak maximum against the scan number of the corresponding tandem mass spectrum [6,7] ***(Supplementary Table 3).***

The configuration of the total disulfide bonds of β2GPI are shown in **Table 1 in main text.**

**Supplementary Information References**

1. Burgess JK, Hotchkiss KA, Suter C, Dudman NP, Szöllösi J, Chesterman CN, Chong BH, Hogg PJ. [Physical proximity and functional association of glycoprotein 1balpha and protein-disulfide isomerase on the platelet plasma membrane.](http://www.ncbi.nlm.nih.gov/pubmed/10734129?ordinalpos=37&itool=EntrezSystem2.PEntrez.Pubmed.Pubmed_ResultsPanel.Pubmed_DefaultReportPanel.Pubmed_RVDocSum) *J Biol Chem* 2000; **275**: 9758-66.

2. Gatlin CL, Kleemann GR, Hays LG, Link AJ, Yates JR 3rd. Protein identification at the low femtomole level from silver-stained gels using a new fritless electrospray interface for liquid chromatography-microspray and nanospray mass spectrometry. *Anal Biochem* 1998; **263**: 93-101.

3. Couttas TA, Raftery MJ, Bernardini G, Wilkins MR. Immonium ion scanning for the discovery of post-translational modifications and its application to histones. *J Proteome Res* 2008; **7**: 2632-41.

4. Keller A, Eng J, Zhang N, Li XJ, Aebersold R. A uniform proteomics MS/MS analysis platform utilizing open XML file formats. *Mol Syst Biol* 2005; **1**: 2005.0017.

5. Craig R, Beavis RC. TANDEM: matching proteins with tandem mass spectra. *Bioinformatics* 2004; **20**: 1466-7.

6. Kalia J, Raines RT. Catalysis of imido group hydrolysis in a maleimide conjugate. *Bioorg Med Chem Lett* 2007; **17**: 6286-9.

7. Froelich JM, Reid GE. The origin and control of ex vivo oxidative peptide modifications prior to mass spectrometry analysis. *Proteomics* 2008; **8**:1334-45.
