# Supplementary material for: Redox control of β2-glycoprotein I–von Willebrand factor interaction by thioredoxin-1
Source: J Thromb Haemost. 2010 Aug;8(8):1754–62. doi: 10.1111/j.1538-7836.2010.03944.x (PMC3017748; doi:10.1111/j.1538-7836.2010.03944.x)
Supplement: Supplementary file 2 [file jth0008-1754-SD2.pdf]

Supplementary Information

Table 1. Results from X!Tandem search for tryptic peptides produced from the three samples: nB2GPI, nB2GPI/TRX-1/TRX-R/NADPH and nB2GPI/TRX-1/TRX-R/NADPH+MPB.

Sample Beta-2-GPI

False discovery rat 0.46%

Proteins

rank	log(e)	log(I)	% (measured	% (corrected	unique	total	Mr	Accession	Description
1	-885.1	7.84	72	99	79	612	38.3	P02749	APOH, Beta-2-glycoprotein 1 Precursor (Beta-2-glycoprotein I)(Beta(2)GPI)(B2GPI)(Apolipoprotein H)(Apo-H)(Activated protein C-binding protein)(APC inhibitor)(Anticardiolipin cofactor) [Source: UniProt P02749]
2	-73	5.35	19	32	10	19	66	P04264	KRT1, Keratin, type II cytoskeletal 1 (Cytokeratin-1) (CK-1) (Keratin-1) (K1) (67 kDa cytokeratin) (Hair alpha protein). Source: Uniprot/SWISSPROT P04264
3	-33.5	5.32	36	42	5	13	11.7	P10599	TXN, Thioredoxin (Trx)(ATL-derived factor)(ADF)(Surface-associated sulphydryl protein)(SASP) [Source: UniProt P10599]

Peptides

Spectrum	log(e)	log(I)	m+h	delta	z	retention	pre	start	sequence	end	post	modifications	Protein	Description
799.1.1	-1.2	4.46	1162.5674	0.0017	3	16.74	iqek	286	FKNGMLHGDK	295	vsff	M [290] 15.9949	ENSP00000205948	Beta-2-glycoprotein 1 Precursor (Beta-2-glycoprotein I)(Beta(2)GPI)(B2GPI)(Apolipoprotein H)(Apo-H)(Activated protein C-binding protein)(APC inhibitor)(Anticardiolipin cofactor) [Source: UniProt P02749]
805.1.1	-1.6	4.03	1162.5674	-0.0002	2	16.74	iqek	286	FKNGMLHGDK	295	vsff	M [290] 15.9949	ENSP00000205948	Beta-2-glycoprotein 1 Precursor (Beta-2-glycoprotein I)(Beta(2)GPI)(B2GPI)(Apolipoprotein H)(Apo-H)(Activated protein C-binding protein)(APC inhibitor)(Anticardiolipin cofactor) [Source: UniProt P02749]
859.1.1	-2.4	4.69	1163.5522	0.0007	3	16.74	iqek	286	FKNGMLHGDK	295	vsff	M [290] 15.9949, N [288] 0.9848	ENSP00000205948	Beta-2-glycoprotein 1 Precursor (Beta-2-glycoprotein I)(Beta(2)GPI)(B2GPI)(Apolipoprotein H)(Apo-H)(Activated protein C-binding protein)(APC inhibitor)(Anticardiolipin cofactor) [Source: UniProt P02749]
898.1.1	-6.6	4.19	992.4353	0.0009	2	6.52	afwk	337	TDASDVKPC	345	]	C [345] 57.0215	ENSP00000205948	Beta-2-glycoprotein 1 Precursor (Beta-2-glycoprotein I)(Beta(2)GPI)(B2GPI)(Apolipoprotein H)(Apo-H)(Activated protein C-binding protein)(APC inhibitor)(Anticardiolipin cofactor) [Source: UniProt P02749]
900.1.1	-5.9	4.56	992.4353	0.0006	2	6.52	afwk	337	TDASDVKPC	345	]	C [345] 57.0215	ENSP00000205948	Beta-2-glycoprotein 1 Precursor (Beta-2-glycoprotein I)(Beta(2)GPI)(B2GPI)(Apolipoprotein H)(Apo-H)(Activated protein C-binding protein)(APC inhibitor)(Anticardiolipin cofactor) [Source: UniProt P02749]
903.1.1	-6.7	4.85	992.4353	0.0006	2	6.52	afwk	337	TDASDVKPC	345	]	C [345] 57.0215	ENSP00000205948	Beta-2-glycoprotein 1 Precursor (Beta-2-glycoprotein I)(Beta(2)GPI)(B2GPI)(Apolipoprotein H)(Apo-H)(Activated protein C-binding protein)(APC inhibitor)(Anticardiolipin cofactor) [Source: UniProt P02749]
968.1.1	-7.1	4.2	992.4353	0.0008	2	6.52	afwk	337	TDASDVKPC	345	]	C [345] 57.0215	ENSP00000205948	Beta-2-glycoprotein 1 Precursor (Beta-2-glycoprotein I)(Beta(2)GPI)(B2GPI)(Apolipoprotein H)(Apo-H)(Activated protein C-binding protein)(APC inhibitor)(Anticardiolipin cofactor) [Source: UniProt P02749]
973.1.1	-6.9	4.19	992.4353	0.0009	2	6.52	afwk	337	TDASDVKPC	345	]	C [345] 57.0215	ENSP00000205948	Beta-2-glycoprotein 1 Precursor (Beta-2-glycoprotein I)(Beta(2)GPI)(B2GPI)(Apolipoprotein H)(Apo-H)(Activated protein C-binding protein)(APC inhibitor)(Anticardiolipin cofactor) [Source: UniProt P02749]
977.1.1	-6.5	4.13	992.4353	0.0008	2	6.52	afwk	337	TDASDVKPC	345	]	C [345] 57.0215	ENSP00000205948	Beta-2-glycoprotein 1 Precursor (Beta-2-glycoprotein I)(Beta(2)GPI)(B2GPI)(Apolipoprotein H)(Apo-H)(Activated protein C-binding protein)(APC inhibitor)(Anticardiolipin cofactor) [Source: UniProt P02749]
988.1.1	-2.8	3.94	1165.5848	-0.0001	2	11.83	kgqk	86	VGEFSGANKEK	96	leat		ENSP00000363641	Thioredoxin (Trx)(ATL-derived factor)(ADF)(Surface-associated sulphydryl protein)(SASP) [Source: UniProt P10599]
991.1.1	-3.8	3.9	1165.5848	0.0006	2	11.83	kgqk	86	VGEFSGANKEK	96	leat		ENSP00000363641	Thioredoxin (Trx)(ATL-derived factor)(ADF)(Surface-associated sulphydryl protein)(SASP) [Source: UniProt P10599]
1031.1.1	-1.7	4.08	902.5128	0.0009	2	8	psck	262	ASCKVPVK	269	katv	C [264] 57.0215, V [266] 14.0157	ENSP00000205948	Beta-2-glycoprotein 1 Precursor (Beta-2-glycoprotein I)(Beta(2)GPI)(B2GPI)(Apolipoprotein H)(Apo-H)(Activated protein C-binding protein)(APC inhibitor)(Anticardiolipin cofactor) [Source: UniProt P02749]
1035.1.1	-1.4	4.24	902.5128	0.0009	2	8	psck	262	ASCKVPVK	269	katv	C [264] 57.0215, V [266] 14.0157	ENSP00000205948	Beta-2-glycoprotein 1 Precursor (Beta-2-glycoprotein I)(Beta(2)GPI)(B2GPI)(Apolipoprotein H)(Apo-H)(Activated protein C-binding protein)(APC inhibitor)(Anticardiolipin cofactor) [Source: UniProt P02749]
1054.1.1	-4.8	4.48	1150.6215	0.001	2	12.63	vpvk	270	KATVVYQGER	279	vkiq		ENSP00000205948	Beta-2-glycoprotein 1 Precursor (Beta-2-glycoprotein I)(Beta(2)GPI)(B2GPI)(Apolipoprotein H)(Apo-H)(Activated protein C-binding protein)(APC inhibitor)(Anticardiolipin cofactor) [Source: UniProt P02749]
1055.1.1	-4.5	4.68	1150.6215	0.0025	3	12.63	vpvk	270	KATVVYQGER	279	vkiq		ENSP00000205948	Beta-2-glycoprotein 1 Precursor (Beta-2-glycoprotein I)(Beta(2)GPI)(B2GPI)(Apolipoprotein H)(Apo-H)(Activated protein C-binding protein)(APC inhibitor)(Anticardiolipin cofactor) [Source: UniProt P02749]
1058.1.1	-4.2	4.92	1150.6215	0.001	2	12.63	vpvk	270	KATVVYQGER	279	vkiq		ENSP00000205948	Beta-2-glycoprotein 1 Precursor (Beta-2-glycoprotein I)(Beta(2)GPI)(B2GPI)(Apolipoprotein H)(Apo-H)(Activated protein C-binding protein)(APC inhibitor)(Anticardiolipin cofactor) [Source: UniProt P02749]
1059.1.1	-2.8	5.2	1150.6215	0.0015	3	12.63	vpvk	270	KATVVYQGER	279	vkiq		ENSP00000205948	Beta-2-glycoprotein 1 Precursor (Beta-2-glycoprotein I)(Beta(2)GPI)(B2GPI)(Apolipoprotein H)(Apo-H)(Activated protein C-binding protein)(APC inhibitor)(Anticardiolipin cofactor) [Source: UniProt P02749]
1065.1.1	-5.3	5.42	1150.6215	0.0004	2	12.63	vpvk	270	KATVVYQGER	279	vkiq		ENSP00000205948	Beta-2-glycoprotein 1 Precursor (Beta-2-glycoprotein I)(Beta(2)GPI)(B2GPI)(Apolipoprotein H)(Apo-H)(Activated protein C-binding protein)(APC inhibitor)(Anticardiolipin cofactor) [Source: UniProt P02749]
1066.1.1	-2.4	5.6	1150.6215	0.0015	3	12.63	vpvk	270	KATVVYQGER	279	vkiq		ENSP00000205948	Beta-2-glycoprotein 1 Precursor (Beta-2-glycoprotein I)(Beta(2)GPI)(B2GPI)(Apolipoprotein H)(Apo-H)(Activated protein C-binding protein)(APC inhibitor)(Anticardiolipin cofactor) [Source: UniProt P02749]
1101.1.1	-2.9	4.05	908.4472	0.001	2	12.82	kgqk	86	VGEFSGANK	94	ekle		ENSP00000363641	Thioredoxin (Trx)(ATL-derived factor)(ADF)(Surface-associated sulphydryl protein)(SASP) [Source: UniProt P10599]
1104.1.1	-3.9	4.2	908.4472	0.0006	2	12.82	kgqk	86	VGEFSGANK	94	ekle		ENSP00000363641	Thioredoxin (Trx)(ATL-derived factor)(ADF)(Surface-associated sulphydryl protein)(SASP) [Source: UniProt P10599]
1106.1.1	-2.7	4.07	908.4472	0.0014	2	12.82	kgqk	86	VGEFSGANK	94	ekle		ENSP00000363641	Thioredoxin (Trx)(ATL-derived factor)(ADF)(Surface-associated sulphydryl protein)(SASP) [Source: UniProt P10599]
1118.1.1	-3.2	3.9	1150.6215	0.001	2	12.63	vpvk	270	KATVVYQGER	279	vkiq		ENSP00000205948	Beta-2-glycoprotein 1 Precursor (Beta-2-glycoprotein I)(Beta(2)GPI)(B2GPI)(Apolipoprotein H)(Apo-H)(Activated protein C-binding protein)(APC inhibitor)(Anticardiolipin cofactor) [Source: UniProt P02749]
1119.1.1	-1.1	4.14	1150.6215	0.0017	3	12.63	vpvk	270	KATVVYQGER	279	vkiq		ENSP00000205948	Beta-2-glycoprotein 1 Precursor (Beta-2-glycoprotein I)(Beta(2)GPI)(B2GPI)(Apolipoprotein H)(Apo-H)(Activated protein C-binding protein)(APC inhibitor)(Anticardiolipin cofactor) [Source: UniProt P02749]
1121.1.1	-4.3	3.88	1150.6215	0.0007	2	12.63	vpvk	270	KATVVYQGER	279	vkiq		ENSP00000205948	Beta-2-glycoprotein 1 Precursor (Beta-2-glycoprotein I)(Beta(2)GPI)(B2GPI)(Apolipoprotein H)(Apo-H)(Activated protein C-binding protein)(APC inhibitor)(Anticardiolipin cofactor) [Source: UniProt P02749]
1122.1.1	-1.1	4.17	1150.6215	0.0014	3	12.63	vpvk	270	KATVVYQGER	279	vkiq		ENSP00000205948	Beta-2-glycoprotein 1 Precursor (Beta-2-glycoprotein I)(Beta(2)GPI)(B2GPI)(Apolipoprotein H)(Apo-H)(Activated protein C-binding protein)(APC inhibitor)(Anticardiolipin cofactor) [Source: UniProt P02749]
1124.1.1	-3.7	3.96	1150.6215	0.001	2	12.63	vpvk	270	KATVVYQGER	279	vkiq		ENSP00000205948	Beta-2-glycoprotein 1 Precursor (Beta-2-glycoprotein I)(Beta(2)GPI)(B2GPI)(Apolipoprotein H)(Apo-H)(Activated protein C-binding protein)(APC inhibitor)(Anticardiolipin cofactor) [Source: UniProt P02749]
1125.1.1	-1.7	4.15	1150.6215	0.0004	3	12.63	vpvk	270	KATVVYQGER	279	vkiq		ENSP00000205948	Beta-2-glycoprotein 1 Precursor (Beta-2-glycoprotein I)(Beta(2)GPI)(B2GPI)(Apolipoprotein H)(Apo-H)(Activated protein C-binding protein)(APC inhibitor)(Anticardiolipin cofactor) [Source: UniProt P02749]
1131.1.1	-3.7	3.85	1062.4772	0.0023	2	10.56	ysld	242	GPEEIECTK	250	lgnw	C [248] 57.0215	ENSP00000205948	Beta-2-glycoprotein 1 Precursor (Beta-2-glycoprotein I)(Beta(2)GPI)(B2GPI)(Apolipoprotein H)(Apo-H)(Activated protein C-binding protein)(APC inhibitor)(Anticardiolipin cofactor) [Source: UniProt P02749]
1133.1.1	-5.4	4.07	1062.4772	0.0007	2	10.56	ysld	242	GPEEIECTK	250	lgnw	C [248] 57.0215	ENSP00000205948	Beta-2-glycoprotein 1 Precursor (Beta-2-glycoprotein I)(Beta(2)GPI)(B2GPI)(Apolipoprotein H)(Apo-H)(Activated protein C-binding protein)(APC inhibitor)(Anticardiolipin cofactor) [Source: UniProt P02749]
1136.1.1	-3.4	4.15	1062.4772	0.0007	2	10.56	ysld	242	GPEEIECTK	250	lgnw	C [248] 57.0215	ENSP00000205948	Beta-2-glycoprotein 1 Precursor (Beta-2-glycoprotein I)(Beta(2)GPI)(B2GPI)(Apolipoprotein H)(Apo-H)(Activated protein C-binding protein)(APC inhibitor)(Anticardiolipin cofactor) [Source: UniProt P02749]
1146.1.1	-1.3	3.77	1084.455	0.0056	2	20.16	rsik	235	NFCEVMER	242	wfvk	C [237] 57.0215	ENSP00000221418:reversed	
1177.1.1	-2.9	3.72	1150.6215	-0.0013	2	12.63	vpvk	270	KATVVYQGER	279	vkiq		ENSP00000205948	Beta-2-glycoprotein 1 Precursor (Beta-2-glycoprotein I)(Beta(2)GPI)(B2GPI)(Apolipoprotein H)(Apo-H)(Activated protein C-binding protein)(APC inhibitor)(Anticardiolipin cofactor) [Source: UniProt P02749]
1179.1.1	-2.3	3.68	1150.6215	0.0014	2	12.63	vpvk	270	KATVVYQGER	279	vkiq		ENSP00000205948	Beta-2-glycoprotein 1 Precursor (Beta-2-glycoprotein I)(Beta(2)GPI)(B2GPI)(Apolipoprotein H)(Apo-H)(Activated protein C-binding protein)(APC inhibitor)(Anticardiolipin cofactor) [Source: UniProt P02749]
1181.1.1	-2	3.69	1150.6215	0	2	12.63	vpvk	270	KATVVYQGER	279	vkiq		ENSP00000205948	Beta-2-glycoprotein 1 Precursor (Beta-2-glycoprotein I)(Beta(2)GPI)(B2GPI)(Apolipoprotein H)(Apo-H)(Activated protein C-binding protein)(APC inhibitor)(Anticardiolipin cofactor) [Source: UniProt P02749]
1204.1.1	-5	4.33	1022.5265	0.0001	2	12.58	pvkk	271	ATVVYQGER	279	vkiq		ENSP00000205948	Beta-2-glycoprotein 1 Precursor (Beta-2-glycoprotein I)(Beta(2)GPI)(B2GPI)(Apolipoprotein H)(Apo-H)(Activated protein C-binding protein)(APC inhibitor)(Anticardiolipin cofactor) [Source: UniProt P02749]
1206.1.1	-5.5	4.92	1022.5265	0.0006	2	12.58	pvkk	271	ATVVYQGER	279	vkiq		ENSP00000205948	Beta-2-glycoprotein 1 Precursor (Beta-2-glycoprotein I)(Beta(2)GPI)(B2GPI)(Apolipoprotein H)(Apo-H)(Activated protein C-binding protein)(APC inhibitor)(Anticardiolipin cofactor) [Source: UniProt P02749]
1262.1.1	-2.3	3.93	1249.6899	-0.0014	2	16.13	pvkk	271	ATVVYQGERVK	281	iqek		ENSP00000205948	Beta-2-glycoprotein 1 Precursor (Beta-2-glycoprotein I)(Beta(2)GPI)(B2GPI)(Apolipoprotein H)(Apo-H)(Activated protein C-binding protein)(APC inhibitor)(Anticardiolipin cofactor) [Source: UniProt P02749]
1267.1.1	-1.4	3.99	1249.6899	-0.0004	2	16.13	pvkk	271	ATVVYQGERVK	281	iqek		ENSP00000205948	Beta-2-glycoprotein 1 Precursor (Beta-2-glycoprotein I)(Beta(2)GPI)(B2GPI)(Apolipoprotein H)(Apo-H)(Activated protein C-binding protein)(APC inhibitor)(Anticardiolipin cofactor) [Source: UniProt P02749]
1279.1.1	-5.8	5.28	1022.5265	0.0005	2	12.58	pvkk	271	ATVVYQGER	279	vkiq		ENSP00000205948	Beta-2-glycoprotein 1 Precursor (Beta-2-glycoprotein I)(Beta(2)GPI)(B2GPI)(Apolipoprotein H)(Apo-H)(Activated protein C-binding protein)(APC inhibitor)(Anticardiolipin cofactor) [Source: UniProt P02749]
1281.1.1	-5.2	5.27	1022.5265	0.0004	2	12.58	pvkk	271	ATVVYQGER	279	vkiq		ENSP00000205948	Beta-2-glycoprotein 1 Precursor (Beta-2-glycoprotein I)(Beta(2)GPI)(B2GPI)(Apolipoprotein H)(Apo-H)(Activated protein C-binding protein)(APC inhibitor)(Anticardiolipin cofactor) [Source: UniProt P02749]
1284.1.1	-5.5	5.25	1022.5265	0.001	2	12.58	pvkk	271	ATVVYQGER	279	vkiq		ENSP00000205948	Beta-2-glycoprotein 1 Precursor (Beta-2-glycoprotein I)(Beta(2)GPI)(B2GPI)(Apolipoprotein H)(Apo-H)(Activated protein C-binding protein)(APC inhibitor)(Anticardiolipin cofactor) [Source: UniProt P02749]
1310.1.1	-4.4	4.09	1065.5211	0.0015	2	11.26	aevk	356	AQYEDIAQK	364	skae		ENSP00000252244	Keratin, type II cytoskeletal 1 (Cytokeratin-1) (CK-1) (Keratin-1) (K1) (67 kDa cytokeratin) (Hair alpha protein). Source: Uniprot/SWISSPROT P04264
1313.1.1	-4.4	4.08	1065.5211	0.0016	2	11.26	aevk	356	AQYEDIAQK	364	skae		ENSP00000252244	Keratin, type II cytoskeletal 1 (Cytokeratin-1) (CK-1) (Keratin-1) (K1) (67 kDa cytokeratin) (Hair alpha protein). Source: Uniprot/SWISSPROT P04264
1317.1.1	-4.1	4.06	1065.5211	-0.0001	2	11.26	aevk	356	AQYEDIAQK	364	skae		ENSP00000252244	Keratin, type II cytoskeletal 1 (Cytokeratin-1) (CK-1) (Keratin-1) (K1) (67 kDa cytokeratin) (Hair alpha protein). Source: Uniprot/SWISSPROT P04264
1334.1.1	-5.4	4.72	1022.5265	0.0008	2	12.58	pvkk	271	ATVVYQGER	279	vkiq		ENSP00000205948	Beta-2-glycoprotein 1 Precursor (Beta-2-glycoprotein I)(Beta(2)GPI)(B2GPI)(Apolipoprotein H)(Apo-H)(Activated protein C-binding protein)(APC inhibitor)(Anticardiolipin cofactor) [Source: UniProt P02749]
1336.1.1	-4.3	4.73	1022.5265	0.0001	2	12.58	pvkk	271	ATVVYQGER	279	vkiq		ENSP00000205948	Beta-2-glycoprotein 1 Precursor (Beta-2-glycoprotein I)(Beta(2)GPI)(B2GPI)(Apolipoprotein H)(Apo-H)(Activated protein C-binding protein)(APC inhibitor)(Anticardiolipin cofactor) [Source: UniProt P02749]
1338.1.1	-4.6	4.7	1022.5265	0.0006	2	12.58	pvkk	271	ATVVYQGER	279	vkiq		ENSP00000205948	Beta-2-glycoprotein 1 Precursor (Beta-2-glycoprotein I)(Beta(2)GPI)(B2GPI)(Apolipoprotein H)(Apo-H)(Activated protein C-binding protein)(APC inhibitor)(Anticardiolipin cofactor) [Source: UniProt P02749]
1373.1.1	-1.1	4.02	1131.5793	-0.001	2	4.89	tldr	1125	SPPPPPPTGER	1135	kllk		ENSP00000385618:reversed	
1381.1.1	-2	4.09	1131.5793	0.0019	2	4.89	tldr	1125	SPPPPPPTGER	1135	kllk		ENSP00000385618:reversed	
1406.1.1	-6.2	4.48	1022.5265	0.0005	2	12.58	pvkk	271	ATVVYQGER	279	vkiq		ENSP00000205948	Beta-2-glycoprotein 1 Precursor (Beta-2-glycoprotein I)(Beta(2)GPI)(B2GPI)(Apolipoprotein H)(Apo-H)(Activated protein C-binding protein)(APC inhibitor)(Anticardiolipin cofactor) [Source: UniProt P02749]
1409.1.1	-5.5	4.46	1022.5265	0.0009	2	12.58	pvkk	271	ATVVYQGER	279	vkiq		ENSP00000205948	Beta-2-glycoprotein 1 Precursor (Beta-2-glycoprotein I)(Beta(2)GPI)(B2GPI)(Apolipoprotein H)(Apo-H)(Activated protein C-binding protein)(APC inhibitor)(Anticardiolipin cofactor) [Source: UniProt P02749]
1410.1.1	-2	4.25	1089.4782	0.0014	2	13.96	revk	205	CPFPSRPDN	213	gfvn	C [205] 57.0215	ENSP00000205948	Beta-2-glycoprotein 1 Precursor (Beta-2-glycoprotein I)(Beta(2)GPI)(B2GPI)(Apolipoprotein H)(Apo-H)(Activated protein C-binding protein)(APC inhibitor)(Anticardiolipin cofactor) [Source: UniProt P02749]
1413.1.1	-5.1	4.46	1022.5265	0.0012	2	12.58	pvkk	271	ATVVYQGER	279	vkiq		ENSP00000205948	Beta-2-glycoprotein 1 Precursor (Beta-2-glycoprotein I)(Beta(2)GPI)(B2GPI)(Apolipoprotein H)(Apo-H)(Activated protein C-binding protein)(APC inhibitor)(Anticardiolipin cofactor) [Source: UniProt P02749]
1414.1.1	-2.8	4.29	1089.4782	0.0032	2	13.96	revk	205	CPFPSRPDN	213	gfvn	C [205] 57.0215	ENSP00000205948	Beta-2-glycoprotein 1 Precursor (Beta-2-glycoprotein I)(Beta(2)GPI)(B2GPI)(Apolipoprotein H)(Apo-H)(Activated protein C-binding protein)(APC inhibitor)(Anticardiolipin cofactor) [Source: UniProt P02749]
1432.1.1	-2.4	4.4	1146.4996	0.0017	2	13.67	revk	205	CPFPSRPDNG	214	fvny	C [205] 57.0215	ENSP00000205948	Beta-2-glycoprotein 1 Precursor (Beta-2-glycoprotein I)(Beta(2)GPI)(B2GPI)(Apolipoprotein H)(Apo-H)(Activated protein C-binding protein)(APC inhibitor)(Anticardiolipin cofactor) [Source: UniProt P02749]
1434.1.1	-2.3	4.34	1146.4996	0.0013	2	13.67	revk	205	CPFPSRPDNG	214	fvny	C [205] 57.0215	ENSP00000205948	Beta-2-glycoprotein 1 Precursor (Beta-2-glycoprotein I)(Beta(2)GPI)(B2GPI)(Apolipoprotein H)(Apo-H)(Activated protein C-binding protein)(APC inhibitor)(Anticardiolipin cofactor) [Source: UniProt P02749]
1448.1.1	-1.7	3.96	1048.5422	0.0018	2	19.65	askk	157	ESVVYGGISR	166	lqad	E [157] -18.0106	ENSP00000387110	
1452.1.1	-4.2	3.96	1033.516	-0.001	2	17.66	atyr	484	TLLEGEESR	492	msge		ENSP00000252244	Keratin, type II cytoskeletal 1 (Cytokeratin-1) (CK-1) (Keratin-1) (K1) (67 kDa cytokeratin) (Hair alpha protein). Source: Uniprot/SWISSPROT P04264
1453.1.1	-1.5	3.94	1048.5422	0.0005	2	18.24	tper	527	ESVVDFSKR	535	ymdy	E [527] -18.0106	ENSP00000330148	Glutamate receptor delta-1 subunit precursor (GluR delta-1). [Source: SWISSPROT (Q9ULK0)]
1458.1.1	-2.1	3.98	1033.516	0.0009	2	17.66	atyr	484	TLLEGEESR	492	msge		ENSP00000252244	Keratin, type II cytoskeletal 1 (Cytokeratin-1) (CK-1) (Keratin-1) (K1) (67 kDa cytokeratin) (Hair alpha protein). Source: Uniprot/SWISSPROT P04264
1459.1.1	-1.1	3.85	1048.5422	0.0024	2	18.24	tper	527	ESVVDFSKR	535	ymdy	E [527] -18.0106	ENSP00000330148	Glutamate receptor delta-1 subunit precursor (GluR delta-1). [Source: SWISSPROT (Q9ULK0)]
1462.1.1	-2.1	4.08	1033.516	0.0004	2	17.66	atyr	484	TLLEGEESR	492	msge		ENSP00000252244	Keratin, type II cytoskeletal 1 (Cytokeratin-1) (CK-1) (Keratin-1) (K1) (67 kDa cytokeratin) (Hair alpha protein). Source: Uniprot/SWISSPROT P04264
1481.1.1	-4.3	4.28	1022.5265	0	2	12.58	pvkk	271	ATVVYQGER	279	vkiq		ENSP00000205948	Beta-2-glycoprotein 1 Precursor (Beta-2-glycoprotein I)(Beta(2)GPI)(B2GPI)(Apolipoprotein H)(Apo-H)(Activated protein C-binding protein)(APC inhibitor)(Anticardiolipin cofactor) [Source: UniProt P02749]
1484.1.1	-4.3	4.3	1022.5265	0.0009	2	12.58	pvkk	271	ATVVYQGER	279	vkiq		ENSP00000205948	Beta-2-glycoprotein 1 Precursor (Beta-2-glycoprotein I)(Beta(2)GPI)(B2GPI)(Apolipoprotein H)(Apo-H)(Activated protein C-binding protein)(APC inhibitor)(Anticardiolipin cofactor) [Source: UniProt P02749]
1492.1.1	-4.3	4.26	1022.5265	0.001	2	12.58	pvkk	271	ATVVYQGER	279	vkiq		ENSP00000205948	Beta-2-glycoprotein 1 Precursor (Beta-2-glycoprotein I)(Beta(2)GPI)(B2GPI)(Apolipoprotein H)(Apo-H)(Activated protein C-binding protein)(APC inhibitor)(Anticardiolipin cofactor) [Source: UniProt P02749]
1501.1.1	-2.3	4.25	975.4353	-0.0007	2	14.95	revk	205	CPFPSRPD	212	ngfv	C [205] 57.0215	ENSP00000205948	Beta-2-glycoprotein 1 Precursor (Beta-2-glycoprotein I)(Beta(2)GPI)(B2GPI)(Apolipoprotein H)(Apo-H)(Activated protein C-binding protein)(APC inhibitor)(Anticardiolipin cofactor) [Source: UniProt P02749]
1506.1.1	-2	4.37	975.4353	0.0008	2	14.95	revk	205	CPFPSRPD	212	ngfv	C [205] 57.0215	ENSP00000205948	Beta-2-glycoprotein 1 Precursor (Beta-2-glycoprotein I)(Beta(2)GPI)(B2GPI)(Apolipoprotein H)(Apo-H)(Activated protein C-binding protein)(APC inhibitor)(Anticardiolipin cofactor) [Source: UniProt P02749]
1508.1.1	-6.7	4.27	1489.5933	-0.0013	2	14.47	nkek	306	KCSYTEDAQCID	317	gtie	C [307] 57.0215, C [315] 57.0215	ENSP00000205948	Beta-2-glycoprotein 1 Precursor (Beta-2-glycoprotein I)(Beta(2)GPI)(B2GPI)(Apolipoprotein H)(Apo-H)(Activated protein C-binding protein)(APC inhibitor)(Anticardiolipin cofactor) [Source: UniProt P02749]
1510.1.1	-3.7	4.36	1489.5933	0.0009	2	14.47	nkek	306	KCSYTEDAQCID	317	gtie	C [307] 57.0215, C [315] 57.0215	ENSP00000205948	Beta-2-glycoprotein 1 Precursor (Beta-2-glycoprotein I)(Beta(2)GPI)(B2GPI)(Apolipoprotein H)(Apo-H)(Activated protein C-binding protein)(APC inhibitor)(Anticardiolipin cofactor) [Source: UniProt P02749]
1512.1.1	-2.1	4.28	975.4353	0.0001	2	14.95	revk	205	CPFPSRPD	212	ngfv	C [205] 57.0215	ENSP00000205948	Beta-2-glycoprotein 1 Precursor (Beta-2-glycoprotein I)(Beta(2)GPI)(B2GPI)(Apolipoprotein H)(Apo-H)(Activated protein C-binding protein)(APC inhibitor)(Anticardiolipin cofactor) [Source: UniProt P02749]
1517.1.1	-4.9	4.46	1489.5933	0.0006	2	14.47	nkek	306	KCSYTEDAQCID	317	gtie	C [307] 57.0215, C [315] 57.0215	ENSP00000205948	Beta-2-glycoprotein 1 Precursor (Beta-2-glycoprotein I)(Beta(2)GPI)(B2GPI)(Apolipoprotein H)(Apo-H)(Activated protein C-binding protein)(APC inhibitor)(Anticardiolipin cofactor) [Source: UniProt P02749]
1520.1.1	-2	4.14	1131.5793	0.0014	2	4.89	tldr	1125	SPPPPPPTGER	1135	kllk		ENSP00000385618:reversed	
1527.1.1	-1.3	4.05	1131.5793	-0.0009	2	4.89	tldr	1125	SPPPPPPTGER	1135	kllk		ENSP00000385618:reversed	
1541.1.1	-2	4.04	1027.3938	0.0009	2	12.75	ykdk	230	ATFGCHDGY	238	sldg	C [234] 57.0215	ENSP00000205948	Beta-2-glycoprotein 1 Precursor (Beta-2-glycoprotein I)(Beta(2)GPI)(B2GPI)(Apolipoprotein H)(Apo-H)(Activated protein C-binding protein)(APC inhibitor)(Anticardiolipin cofactor) [Source: UniProt P02749]
1565.1.1	-4.7	4.15	1022.5265	0.0007	2	12.58	pvkk	271	ATVVYQGER	279	vkiq		ENSP00000205948	Beta-2-glycoprotein 1 Precursor (Beta-2-glycoprotein I)(Beta(2)GPI)(B2GPI)(Apolipoprotein H)(Apo-H)(Activated protein C-binding protein)(APC inhibitor)(Anticardiolipin cofactor) [Source: UniProt P02749]
1567.1.1	-4.8	4.1	1022.5265	0.0009	2	12.58	pvkk	271	ATVVYQGER	279	vkiq		ENSP00000205948	Beta-2-glycoprotein 1 Precursor (Beta-2-glycoprotein I)(Beta(2)GPI)(B2GPI)(Apolipoprotein H)(Apo-H)(Activated protein C-binding protein)(APC inhibitor)(Anticardiolipin cofactor) [Source: UniProt P02749]
1569.1.1	-4.7	4.08	1022.5265	0.0004	2	12.58	pvkk	271	ATVVYQGER	279	vkiq		ENSP00000205948	Beta-2-glycoprotein 1 Precursor (Beta-2-glycoprotein I)(Beta(2)GPI)(B2GPI)(Apolipoprotein H)(Apo-H)(Activated protein C-binding protein)(APC inhibitor)(Anticardiolipin cofactor) [Source: UniProt P02749]
1628.1.1	-5.3	4.12	1022.5265	0.0003	2	12.58	pvkk	271	ATVVYQGER	279	vkiq		ENSP00000205948	Beta-2-glycoprotein 1 Precursor (Beta-2-glycoprotein I)(Beta(2)GPI)(B2GPI)(Apolipoprotein H)(Apo-H)(Activated protein C-binding protein)(APC inhibitor)(Anticardiolipin cofactor) [Source: UniProt P02749]
1631.1.1	-3.2	4.07	1022.5265	0.0013	2	12.58	pvkk	271	ATVVYQGER	279	vkiq		ENSP00000205948	Beta-2-glycoprotein 1 Precursor (Beta-2-glycoprotein I)(Beta(2)GPI)(B2GPI)(Apolipoprotein H)(Apo-H)(Activated protein C-binding protein)(APC inhibitor)(Anticardiolipin cofactor) [Source: UniProt P02749]
1636.1.1	-3.9	4.07	1022.5265	0.0006	2	12.58	pvkk	271	ATVVYQGER	279	vkiq		ENSP00000205948	Beta-2-glycoprotein 1 Precursor (Beta-2-glycoprotein I)(Beta(2)GPI)(B2GPI)(Apolipoprotein H)(Apo-H)(Activated protein C-binding protein)(APC inhibitor)(Anticardiolipin cofactor) [Source: UniProt P02749]
1657.1.1	-3	4.24	999.5582	0.0011	2	20.12	vcpf	87	AGILENGAVR	96	yttf		ENSP00000205948	Beta-2-glycoprotein 1 Precursor (Beta-2-glycoprotein I)(Beta(2)GPI)(B2GPI)(Apolipoprotein H)(Apo-H)(Activated protein C-binding protein)(APC inhibitor)(Anticardiolipin cofactor) [Source: UniProt P02749]
1661.1.1	-3.4	4.57	999.5582	0.0011	2	20.12	vcpf	87	AGILENGAVR	96	yttf		ENSP00000205948	Beta-2-glycoprotein 1 Precursor (Beta-2-glycoprotein I)(Beta(2)GPI)(B2GPI)(Apolipoprotein H)(Apo-H)(Activated protein C-binding protein)(APC inhibitor)(Anticardiolipin cofactor) [Source: UniProt P02749]
1664.1.1	-3.4	4.74	999.5582	0.0006	2	20.12	vcpf	87	AGILENGAVR	96	yttf		ENSP00000205948	Beta-2-glycoprotein 1 Precursor (Beta-2-glycoprotein I)(Beta(2)GPI)(B2GPI)(Apolipoprotein H)(Apo-H)(Activated protein C-binding protein)(APC inhibitor)(Anticardiolipin cofactor) [Source: UniProt P02749]

1687.1.1	-2.1	3.83	1243.6721	-0.0028	2	23.46	gfvn	218	YPAKPTLYYK	227	dkat		ENSP00000205948	Beta-2-glycoprotein 1 Precursor (Beta-2-glycoprotein I)(Beta(2)GPI)(B2GPI)(Apolipoprotein H)(Apo-H)(Activated protein C-binding protein)(APC inhibitor)(Anticardiolipin cofactor) [Source: UniProt P02749]
1690.1.1	-3.4	3.91	1243.6721	-0.0003	2	23.46	gfvn	218	YPAKPTLYYK	227	dkat		ENSP00000205948	Beta-2-glycoprotein 1 Precursor (Beta-2-glycoprotein I)(Beta(2)GPI)(B2GPI)(Apolipoprotein H)(Apo-H)(Activated protein C-binding protein)(APC inhibitor)(Anticardiolipin cofactor) [Source: UniProt P02749]
1693.1.1	-1.7	4.31	1393.7322	0.0025	3	22.63	inkr	278	TNAENEFVTIKK	289	dvdg		ENSP00000252244	Keratin, type II cytoskeletal 1 (Cytokeratin-1) (CK-1) (Keratin-1) (K1) (67 kDa cytokeratin) (Hair alpha protein). Source: Uniprot/SWISSPROT P04264
1697.1.1	-6.1	3.71	1377.6204	-0.0023	2	19.79	hdgy	239	SLDGPEEIECTK	250	lgnw	C [248] 57.0215	ENSP00000205948	Beta-2-glycoprotein 1 Precursor (Beta-2-glycoprotein I)(Beta(2)GPI)(B2GPI)(Apolipoprotein H)(Apo-H)(Activated protein C-binding protein)(APC inhibitor)(Anticardiolipin cofactor) [Source: UniProt P02749]
1701.1.1	-5.3	3.93	1377.6204	-0.0009	2	19.79	hdgy	239	SLDGPEEIECTK	250	lgnw	C [248] 57.0215	ENSP00000205948	Beta-2-glycoprotein 1 Precursor (Beta-2-glycoprotein I)(Beta(2)GPI)(B2GPI)(Apolipoprotein H)(Apo-H)(Activated protein C-binding protein)(APC inhibitor)(Anticardiolipin cofactor) [Source: UniProt P02749]
1702.1.1	-3.3	3.94	1022.5265	0.0013	2	12.58	pvkk	271	ATVVYQGER	279	vkiq		ENSP00000205948	Beta-2-glycoprotein 1 Precursor (Beta-2-glycoprotein I)(Beta(2)GPI)(B2GPI)(Apolipoprotein H)(Apo-H)(Activated protein C-binding protein)(APC inhibitor)(Anticardiolipin cofactor) [Source: UniProt P02749]
1703.1.1	-5	4.12	1361.4985	0.0055	2	15.77	kekk	307	CSYTEDAQCID	317	gtie	C [307] 57.0215, C [315] 57.0215	ENSP00000205948	Beta-2-glycoprotein 1 Precursor (Beta-2-glycoprotein I)(Beta(2)GPI)(B2GPI)(Apolipoprotein H)(Apo-H)(Activated protein C-binding protein)(APC inhibitor)(Anticardiolipin cofactor) [Source: UniProt P02749]
1709.1.1	-8.8	4.07	1377.6204	-0.0007	2	19.79	hdgy	239	SLDGPEEIECTK	250	lgnw	C [248] 57.0215	ENSP00000205948	Beta-2-glycoprotein 1 Precursor (Beta-2-glycoprotein I)(Beta(2)GPI)(B2GPI)(Apolipoprotein H)(Apo-H)(Activated protein C-binding protein)(APC inhibitor)(Anticardiolipin cofactor) [Source: UniProt P02749]
1710.1.1	-5.5	4.05	1361.4985	0.004	2	15.77	kekk	307	CSYTEDAQCID	317	gtie	C [307] 57.0215, C [315] 57.0215	ENSP00000205948	Beta-2-glycoprotein 1 Precursor (Beta-2-glycoprotein I)(Beta(2)GPI)(B2GPI)(Apolipoprotein H)(Apo-H)(Activated protein C-binding protein)(APC inhibitor)(Anticardiolipin cofactor) [Source: UniProt P02749]
1711.1.1	-4.1	4.05	1022.5265	0.0017	2	12.58	pvkk	271	ATVVYQGER	279	vkiq		ENSP00000205948	Beta-2-glycoprotein 1 Precursor (Beta-2-glycoprotein I)(Beta(2)GPI)(B2GPI)(Apolipoprotein H)(Apo-H)(Activated protein C-binding protein)(APC inhibitor)(Anticardiolipin cofactor) [Source: UniProt P02749]
1716.1.1	-4	4.04	1022.5265	-0.0004	2	12.58	pvkk	271	ATVVYQGER	279	vkiq		ENSP00000205948	Beta-2-glycoprotein 1 Precursor (Beta-2-glycoprotein I)(Beta(2)GPI)(B2GPI)(Apolipoprotein H)(Apo-H)(Activated protein C-binding protein)(APC inhibitor)(Anticardiolipin cofactor) [Source: UniProt P02749]
1719.1.1	-2.8	3.85	2915.1597	-0.0062	3	5.08	ygsg	543	GGGGGGRGSYGSGGSS	579	sgss	G [553] -57.0215	ENSP00000252244	Keratin, type II cytoskeletal 1 (Cytokeratin-1) (CK-1) (Keratin-1) (K1) (67 kDa cytokeratin) (Hair alpha protein). Source: Uniprot/SWISSPROT P04264
1724.1.1	-3.7	3.98	1381.6482	-0.004	2	21.44	dkvr	166	ALEESNYELEGK	177	ikew		ENSP00000269576	Keratin, type I cytoskeletal 10 (Cytokeratin-10) (CK-10) (Keratin-10) (K10). Source: Uniprot/SWISSPROT P13645
1734.1.1	-1.3	4.31	832.4887	0.0009	2	19.2	ggsk	75	SISISVAR	82	gggr		ENSP00000252244	Keratin, type II cytoskeletal 1 (Cytokeratin-1) (CK-1) (Keratin-1) (K1) (67 kDa cytokeratin) (Hair alpha protein). Source: Uniprot/SWISSPROT P04264
1739.1.1	-1.1	3.94	874.4993	0.0009	2	18.24	fgsr	66	SLVNLGGSK	74	sisi		ENSP00000252244	Keratin, type II cytoskeletal 1 (Cytokeratin-1) (CK-1) (Keratin-1) (K1) (67 kDa cytokeratin) (Hair alpha protein). Source: Uniprot/SWISSPROT P04264
1784.1.1	-4	3.93	1022.5265	-0.001	2	12.58	pvkk	271	ATVVYQGER	279	vkiq		ENSP00000205948	Beta-2-glycoprotein 1 Precursor (Beta-2-glycoprotein I)(Beta(2)GPI)(B2GPI)(Apolipoprotein H)(Apo-H)(Activated protein C-binding protein)(APC inhibitor)(Anticardiolipin cofactor) [Source: UniProt P02749]
1788.1.1	-4	3.79	1022.5265	0.001	2	12.58	pvkk	271	ATVVYQGER	279	vkiq		ENSP00000205948	Beta-2-glycoprotein 1 Precursor (Beta-2-glycoprotein I)(Beta(2)GPI)(B2GPI)(Apolipoprotein H)(Apo-H)(Activated protein C-binding protein)(APC inhibitor)(Anticardiolipin cofactor) [Source: UniProt P02749]
1791.1.1	-1.9	3.75	1022.5265	0.002	2	12.58	pvkk	271	ATVVYQGER	279	vkiq		ENSP00000205948	Beta-2-glycoprotein 1 Precursor (Beta-2-glycoprotein I)(Beta(2)GPI)(B2GPI)(Apolipoprotein H)(Apo-H)(Activated protein C-binding protein)(APC inhibitor)(Anticardiolipin cofactor) [Source: UniProt P02749]
1793.1.1	-4.6	4.13	2109.9546	-0.0039	3	32.78	kcfk	328	EHSSLAFWKTDASDVKF	345	]	C [345] 57.0215, W [335] 31.9898	ENSP00000205948	Beta-2-glycoprotein 1 Precursor (Beta-2-glycoprotein I)(Beta(2)GPI)(B2GPI)(Apolipoprotein H)(Apo-H)(Activated protein C-binding protein)(APC inhibitor)(Anticardiolipin cofactor) [Source: UniProt P02749]
1794.1.1	-1.6	4.35	2109.9546	0.0029	4	32.78	kcfk	328	EHSSLAFWKTDASDVKF	345	]	C [345] 57.0215, W [335] 31.9898	ENSP00000205948	Beta-2-glycoprotein 1 Precursor (Beta-2-glycoprotein I)(Beta(2)GPI)(B2GPI)(Apolipoprotein H)(Apo-H)(Activated protein C-binding protein)(APC inhibitor)(Anticardiolipin cofactor) [Source: UniProt P02749]
1797.1.1	-3.7	4.22	2109.9546	-0.005	3	32.78	kcfk	328	EHSSLAFWKTDASDVKF	345	]	C [345] 57.0215, W [335] 31.9898	ENSP00000205948	Beta-2-glycoprotein 1 Precursor (Beta-2-glycoprotein I)(Beta(2)GPI)(B2GPI)(Apolipoprotein H)(Apo-H)(Activated protein C-binding protein)(APC inhibitor)(Anticardiolipin cofactor) [Source: UniProt P02749]
1799.1.1	-5.5	4.3	2109.9546	-0.0026	3	32.78	kcfk	328	EHSSLAFWKTDASDVKF	345	]	C [345] 57.0215, W [335] 31.9898	ENSP00000205948	Beta-2-glycoprotein 1 Precursor (Beta-2-glycoprotein I)(Beta(2)GPI)(B2GPI)(Apolipoprotein H)(Apo-H)(Activated protein C-binding protein)(APC inhibitor)(Anticardiolipin cofactor) [Source: UniProt P02749]
1800.1.1	-3.8	4.42	2109.9546	0.0004	4	32.78	kcfk	328	EHSSLAFWKTDASDVKF	345	]	C [345] 57.0215, W [335] 31.9898	ENSP00000205948	Beta-2-glycoprotein 1 Precursor (Beta-2-glycoprotein I)(Beta(2)GPI)(B2GPI)(Apolipoprotein H)(Apo-H)(Activated protein C-binding protein)(APC inhibitor)(Anticardiolipin cofactor) [Source: UniProt P02749]
1806.1.1	-2.4	4.17	2109.9546	0.0011	4	32.78	kcfk	328	EHSSLAFWKTDASDVKF	345	]	C [345] 57.0215, W [335] 31.9898	ENSP00000205948	Beta-2-glycoprotein 1 Precursor (Beta-2-glycoprotein I)(Beta(2)GPI)(B2GPI)(Apolipoprotein H)(Apo-H)(Activated protein C-binding protein)(APC inhibitor)(Anticardiolipin cofactor) [Source: UniProt P02749]
1818.1.1	-3.3	4.59	1655.7669	0.0015	3	31.46	ekfk	288	NGMLHGDKVSFFCK	301	nkek	C [300] 57.0215, M [290] 15.9949	ENSP00000205948	Beta-2-glycoprotein 1 Precursor (Beta-2-glycoprotein I)(Beta(2)GPI)(B2GPI)(Apolipoprotein H)(Apo-H)(Activated protein C-binding protein)(APC inhibitor)(Anticardiolipin cofactor) [Source: UniProt P02749]
1824.1.1	-2.5	5.16	1655.7669	0.0019	3	31.46	ekfk	288	NGMLHGDKVSFFCK	301	nkek	C [300] 57.0215, M [290] 15.9949	ENSP00000205948	Beta-2-glycoprotein 1 Precursor (Beta-2-glycoprotein I)(Beta(2)GPI)(B2GPI)(Apolipoprotein H)(Apo-H)(Activated protein C-binding protein)(APC inhibitor)(Anticardiolipin cofactor) [Source: UniProt P02749]
1828.1.1	-3.3	3.78	1670.7841	-0.0013	2	21.08	yepg	45	EEITYSCKPGYVSR	58	ggmr	C [51] 57.0215, E [45] -18.0106	ENSP00000205948	Beta-2-glycoprotein 1 Precursor (Beta-2-glycoprotein I)(Beta(2)GPI)(B2GPI)(Apolipoprotein H)(Apo-H)(Activated protein C-binding protein)(APC inhibitor)(Anticardiolipin cofactor) [Source: UniProt P02749]
1831.1.1	-4.3	5.62	1655.7669	0.0006	3	31.46	ekfk	288	NGMLHGDKVSFFCK	301	nkek	C [300] 57.0215, M [290] 15.9949	ENSP00000205948	Beta-2-glycoprotein 1 Precursor (Beta-2-glycoprotein I)(Beta(2)GPI)(B2GPI)(Apolipoprotein H)(Apo-H)(Activated protein C-binding protein)(APC inhibitor)(Anticardiolipin cofactor) [Source: UniProt P02749]
1835.1.1	-7.2	4.28	1655.7669	-0.0033	2	31.46	ekfk	288	NGMLHGDKVSFFCK	301	nkek	C [300] 57.0215, M [290] 15.9949	ENSP00000205948	Beta-2-glycoprotein 1 Precursor (Beta-2-glycoprotein I)(Beta(2)GPI)(B2GPI)(Apolipoprotein H)(Apo-H)(Activated protein C-binding protein)(APC inhibitor)(Anticardiolipin cofactor) [Source: UniProt P02749]
1837.1.1	-9.6	4.28	1655.7669	0.0013	2	31.46	ekfk	288	NGMLHGDKVSFFCK	301	nkek	C [300] 57.0215, M [290] 15.9949	ENSP00000205948	Beta-2-glycoprotein 1 Precursor (Beta-2-glycoprotein I)(Beta(2)GPI)(B2GPI)(Apolipoprotein H)(Apo-H)(Activated protein C-binding protein)(APC inhibitor)(Anticardiolipin cofactor) [Source: UniProt P02749]
1840.1.1	-2.2	4.59	1087.5241	0.0042	3	22.69	gmlh	293	GDKVSFFCK	301	nkek	C [300] 57.0215	ENSP00000205948	Beta-2-glycoprotein 1 Precursor (Beta-2-glycoprotein I)(Beta(2)GPI)(B2GPI)(Apolipoprotein H)(Apo-H)(Activated protein C-binding protein)(APC inhibitor)(Anticardiolipin cofactor) [Source: UniProt P02749]
1845.1.1	-2.7	4.78	1087.5241	0.0009	3	22.69	gmlh	293	GDKVSFFCK	301	nkek	C [300] 57.0215	ENSP00000205948	Beta-2-glycoprotein 1 Precursor (Beta-2-glycoprotein I)(Beta(2)GPI)(B2GPI)(Apolipoprotein H)(Apo-H)(Activated protein C-binding protein)(APC inhibitor)(Anticardiolipin cofactor) [Source: UniProt P02749]
1847.1.1	-5.5	4.27	1655.7669	-0.0024	2	31.46	ekfk	288	NGMLHGDKVSFFCK	301	nkek	C [300] 57.0215, M [290] 15.9949	ENSP00000205948	Beta-2-glycoprotein 1 Precursor (Beta-2-glycoprotein I)(Beta(2)GPI)(B2GPI)(Apolipoprotein H)(Apo-H)(Activated protein C-binding protein)(APC inhibitor)(Anticardiolipin cofactor) [Source: UniProt P02749]
1848.1.1	-5.1	3.9	1475.7853	0.0032	2	24.75	dkvr	200	FLEQQNQVLQTK	211	well		ENSP00000252244	Keratin, type II cytoskeletal 1 (Cytokeratin-1) (CK-1) (Keratin-1) (K1) (67 kDa cytokeratin) (Hair alpha protein). Source: Uniprot/SWISSPROT P04264
1856.1.1	-4.2	3.79	1475.7853	-0.0028	2	24.75	dkvr	200	FLEQQNQVLQTK	211	well		ENSP00000252244	Keratin, type II cytoskeletal 1 (Cytokeratin-1) (CK-1) (Keratin-1) (K1) (67 kDa cytokeratin) (Hair alpha protein). Source: Uniprot/SWISSPROT P04264
1859.1.1	-3.5	3.79	1456.7834	-0.0023	2	26.5	dngf	216	VNYPAKPTLYYK	227	dkat		ENSP00000205948	Beta-2-glycoprotein 1 Precursor (Beta-2-glycoprotein I)(Beta(2)GPI)(B2GPI)(Apolipoprotein H)(Apo-H)(Activated protein C-binding protein)(APC inhibitor)(Anticardiolipin cofactor) [Source: UniProt P02749]
1860.1.1	-4.6	3.63	1475.7853	0.0015	2	24.75	dkvr	200	FLEQQNQVLQTK	211	well		ENSP00000252244	Keratin, type II cytoskeletal 1 (Cytokeratin-1) (CK-1) (Keratin-1) (K1) (67 kDa cytokeratin) (Hair alpha protein). Source: Uniprot/SWISSPROT P04264
1861.1.1	-4	3.71	1585.6588	0.003	2	22.82	lyyk	228	DKATFGCHDGYSLD	241	gpee	C [234] 57.0215	ENSP00000205948	Beta-2-glycoprotein 1 Precursor (Beta-2-glycoprotein I)(Beta(2)GPI)(B2GPI)(Apolipoprotein H)(Apo-H)(Activated protein C-binding protein)(APC inhibitor)(Anticardiolipin cofactor) [Source: UniProt P02749]
1863.1.1	-1.6	4.62	1930.9302	-0.0012	4	34.25	iqek	286	FKNGMLHGDKVSFFCK	301	nkek	C [300] 57.0215, M [290] 15.9949	ENSP00000205948	Beta-2-glycoprotein 1 Precursor (Beta-2-glycoprotein I)(Beta(2)GPI)(B2GPI)(Apolipoprotein H)(Apo-H)(Activated protein C-binding protein)(APC inhibitor)(Anticardiolipin cofactor) [Source: UniProt P02749]
1868.1.1	-5.4	3.9	1022.5265	0.0006	2	12.58	pvkk	271	ATVVYQGER	279	vkiq		ENSP00000205948	Beta-2-glycoprotein 1 Precursor (Beta-2-glycoprotein I)(Beta(2)GPI)(B2GPI)(Apolipoprotein H)(Apo-H)(Activated protein C-binding protein)(APC inhibitor)(Anticardiolipin cofactor) [Source: UniProt P02749]
1873.1.1	-2.3	4.89	1930.9302	0.0015	4	34.25	iqek	286	FKNGMLHGDKVSFFCK	301	nkek	C [300] 57.0215, M [290] 15.9949	ENSP00000205948	Beta-2-glycoprotein 1 Precursor (Beta-2-glycoprotein I)(Beta(2)GPI)(B2GPI)(Apolipoprotein H)(Apo-H)(Activated protein C-binding protein)(APC inhibitor)(Anticardiolipin cofactor) [Source: UniProt P02749]
1874.1.1	-1.5	4.15	1930.9302	0.0056	3	34.25	iqek	286	FKNGMLHGDKVSFFCK	301	nkek	C [300] 57.0215, M [290] 15.9949	ENSP00000205948	Beta-2-glycoprotein 1 Precursor (Beta-2-glycoprotein I)(Beta(2)GPI)(B2GPI)(Apolipoprotein H)(Apo-H)(Activated protein C-binding protein)(APC inhibitor)(Anticardiolipin cofactor) [Source: UniProt P02749]
1876.1.1	-3.3	3.88	1022.5265	0.0024	2	12.58	pvkk	271	ATVVYQGER	279	vkiq		ENSP00000205948	Beta-2-glycoprotein 1 Precursor (Beta-2-glycoprotein I)(Beta(2)GPI)(B2GPI)(Apolipoprotein H)(Apo-H)(Activated protein C-binding protein)(APC inhibitor)(Anticardiolipin cofactor) [Source: UniProt P02749]
1879.1.1	-1.6	4.07	1930.9302	-0.0016	3	34.25	iqek	286	FKNGMLHGDKVSFFCK	301	nkek	C [300] 57.0215, M [290] 15.9949	ENSP00000205948	Beta-2-glycoprotein 1 Precursor (Beta-2-glycoprotein I)(Beta(2)GPI)(B2GPI)(Apolipoprotein H)(Apo-H)(Activated protein C-binding protein)(APC inhibitor)(Anticardiolipin cofactor) [Source: UniProt P02749]
1881.1.1	-5.5	3.88	1022.5265	0.0004	2	12.58	pvkk	271	ATVVYQGER	279	vkiq		ENSP00000205948	Beta-2-glycoprotein 1 Precursor (Beta-2-glycoprotein I)(Beta(2)GPI)(B2GPI)(Apolipoprotein H)(Apo-H)(Activated protein C-binding protein)(APC inhibitor)(Anticardiolipin cofactor) [Source: UniProt P02749]
1884.1.1	-1.5	4.06	1930.9302	-0.0028	3	34.25	iqek	286	FKNGMLHGDKVSFFCK	301	nkek	C [300] 57.0215, M [290] 15.9949	ENSP00000205948	Beta-2-glycoprotein 1 Precursor (Beta-2-glycoprotein I)(Beta(2)GPI)(B2GPI)(Apolipoprotein H)(Apo-H)(Activated protein C-binding protein)(APC inhibitor)(Anticardiolipin cofactor) [Source: UniProt P02749]
1907.1.1	-5.9	4.22	1671.7618	-0.0019	3	31.46	ekfk	288	NGMLHGDKVSFFCK	301	nkek	C [300] 57.0215, M [290] 31.9898	ENSP00000205948	Beta-2-glycoprotein 1 Precursor (Beta-2-glycoprotein I)(Beta(2)GPI)(B2GPI)(Apolipoprotein H)(Apo-H)(Activated protein C-binding protein)(APC inhibitor)(Anticardiolipin cofactor) [Source: UniProt P02749]
1912.1.1	-6.4	4.27	1671.7618	-0.0001	3	31.46	ekfk	288	NGMLHGDKVSFFCK	301	nkek	C [300] 57.0215, M [290] 31.9898	ENSP00000205948	Beta-2-glycoprotein 1 Precursor (Beta-2-glycoprotein I)(Beta(2)GPI)(B2GPI)(Apolipoprotein H)(Apo-H)(Activated protein C-binding protein)(APC inhibitor)(Anticardiolipin cofactor) [Source: UniProt P02749]
1914.1.1	-2.8	4.24	1655.7669	-0.0031	3	31.46	ekfk	288	NGMLHGDKVSFFCK	301	nkek	C [300] 57.0215, M [290] 15.9949	ENSP00000205948	Beta-2-glycoprotein 1 Precursor (Beta-2-glycoprotein I)(Beta(2)GPI)(B2GPI)(Apolipoprotein H)(Apo-H)(Activated protein C-binding protein)(APC inhibitor)(Anticardiolipin cofactor) [Source: UniProt P02749]
1917.1.1	-4.5	4.16	1330.6671	0.0014	2	21.18	yted	313	AQCIDGTIEVPK	324	cfke	C [315] 57.0215	ENSP00000205948	Beta-2-glycoprotein 1 Precursor (Beta-2-glycoprotein I)(Beta(2)GPI)(B2GPI)(Apolipoprotein H)(Apo-H)(Activated protein C-binding protein)(APC inhibitor)(Anticardiolipin cofactor) [Source: UniProt P02749]
1918.1.1	-2.9	4.1	1671.7618	0.001	3	31.46	ekfk	288	NGMLHGDKVSFFCK	301	nkek	C [300] 57.0215, M [290] 31.9898	ENSP00000205948	Beta-2-glycoprotein 1 Precursor (Beta-2-glycoprotein I)(Beta(2)GPI)(B2GPI)(Apolipoprotein H)(Apo-H)(Activated protein C-binding protein)(APC inhibitor)(Anticardiolipin cofactor) [Source: UniProt P02749]
1919.1.1	-2.8	4.21	1655.7669	0	3	31.46	ekfk	288	NGMLHGDKVSFFCK	301	nkek	C [300] 57.0215, M [290] 15.9949	ENSP00000205948	Beta-2-glycoprotein 1 Precursor (Beta-2-glycoprotein I)(Beta(2)GPI)(B2GPI)(Apolipoprotein H)(Apo-H)(Activated protein C-binding protein)(APC inhibitor)(Anticardiolipin cofactor) [Source: UniProt P02749]
1921.1.1	-6	4.45	1330.6671	0.0003	2	21.18	yted	313	AQCIDGTIEVPK	324	cfke	C [315] 57.0215	ENSP00000205948	Beta-2-glycoprotein 1 Precursor (Beta-2-glycoprotein I)(Beta(2)GPI)(B2GPI)(Apolipoprotein H)(Apo-H)(Activated protein C-binding protein)(APC inhibitor)(Anticardiolipin cofactor) [Source: UniProt P02749]
1923.1.1	-3.3	4.11	1655.7669	0.0028	3	31.46	ekfk	288	NGMLHGDKVSFFCK	301	nkek	C [300] 57.0215, M [290] 15.9949	ENSP00000205948	Beta-2-glycoprotein 1 Precursor (Beta-2-glycoprotein I)(Beta(2)GPI)(B2GPI)(Apolipoprotein H)(Apo-H)(Activated protein C-binding protein)(APC inhibitor)(Anticardiolipin cofactor) [Source: UniProt P02749]
1927.1.1	-6.3	4.88	1330.6671	-0.0009	2	21.18	yted	313	AQCIDGTIEVPK	324	cfke	C [315] 57.0215	ENSP00000205948	Beta-2-glycoprotein 1 Precursor (Beta-2-glycoprotein I)(Beta(2)GPI)(B2GPI)(Apolipoprotein H)(Apo-H)(Activated protein C-binding protein)(APC inhibitor)(Anticardiolipin cofactor) [Source: UniProt P02749]
1929.1.1	-4.1	3.92	2066.843	0.0046	3	22.46	katf	233	GCHDGYSLDGPEEIECT	250	lgnw	C [234] 57.0215, C [248] 57.0215	ENSP00000205948	Beta-2-glycoprotein 1 Precursor (Beta-2-glycoprotein I)(Beta(2)GPI)(B2GPI)(Apolipoprotein H)(Apo-H)(Activated protein C-binding protein)(APC inhibitor)(Anticardiolipin cofactor) [Source: UniProt P02749]
1930.1.1	-5.6	3.78	1179.6004	0.0009	2	21.27	yqsk	377	YEELQITAGR	386	hgds		ENSP00000252244	Keratin, type II cytoskeletal 1 (Cytokeratin-1) (CK-1) (Keratin-1) (K1) (67 kDa cytokeratin) (Hair alpha protein). Source: Uniprot/SWISSPROT P04264
1935.1.1	-9.3	3.97	2066.843	-0.0016	3	22.46	katf	233	GCHDGYSLDGPEEIECT	250	lgnw	C [234] 57.0215, C [248] 57.0215	ENSP00000205948	Beta-2-glycoprotein 1 Precursor (Beta-2-glycoprotein I)(Beta(2)GPI)(B2GPI)(Apolipoprotein H)(Apo-H)(Activated protein C-binding protein)(APC inhibitor)(Anticardiolipin cofactor) [Source: UniProt P02749]
1941.1.1	-5.2	3.65	1179.6004	-0.0004	2	21.27	yqsk	377	YEELQITAGR	386	hgds		ENSP00000252244	Keratin, type II cytoskeletal 1 (Cytokeratin-1) (CK-1) (Keratin-1) (K1) (67 kDa cytokeratin) (Hair alpha protein). Source: Uniprot/SWISSPROT P04264
1946.1.1	-6.7	3.71	2066.843	-0.0034	3	22.46	katf	233	GCHDGYSLDGPEEIECT	250	lgnw	C [234] 57.0215, C [248] 57.0215	ENSP00000205948	Beta-2-glycoprotein 1 Precursor (Beta-2-glycoprotein I)(Beta(2)GPI)(B2GPI)(Apolipoprotein H)(Apo-H)(Activated protein C-binding protein)(APC inhibitor)(Anticardiolipin cofactor) [Source: UniProt P02749]
1953.1.1	-3.8	3.66	1953.901	-0.0035	2	23.56	ktfy	42	EPGEEITYSCKPGYVSR	58	ggmr	C [51] 57.0215, E [42] -18.0106	ENSP00000205948	Beta-2-glycoprotein 1 Precursor (Beta-2-glycoprotein I)(Beta(2)GPI)(B2GPI)(Apolipoprotein H)(Apo-H)(Activated protein C-binding protein)(APC inhibitor)(Anticardiolipin cofactor) [Source: UniProt P02749]
1955.1.1	-1.8	5.52	1931.915	0.0009	4	34.25	iqek	286	FKNGMLHGDKVSFFCK	301	nkek	C [300] 57.0215, M [290] 15.9949, N [28	ENSP00000205948	Beta-2-glycoprotein 1 Precursor (Beta-2-glycoprotein I)(Beta(2)GPI)(B2GPI)(Apolipoprotein H)(Apo-H)(Activated protein C-binding protein)(APC inhibitor)(Anticardiolipin cofactor) [Source: UniProt P02749]
1968.1.1	-1.8	5.21	1931.915	-0.002	3	34.25	iqek	286	FKNGMLHGDKVSFFCK	301	nkek	C [300] 57.0215, M [290] 15.9949, N [28	ENSP00000205948	Beta-2-glycoprotein 1 Precursor (Beta-2-glycoprotein I)(Beta(2)GPI)(B2GPI)(Apolipoprotein H)(Apo-H)(Activated protein C-binding protein)(APC inhibitor)(Anticardiolipin cofactor) [Source: UniProt P02749]
1970.1.1	-4	4.01	1931.915	-0.0066	2	34.25	iqek	286	FKNGMLHGDKVSFFCK	301	nkek	C [300] 57.0215, M [290] 15.9949, N [28	ENSP00000205948	Beta-2-glycoprotein 1 Precursor (Beta-2-glycoprotein I)(Beta(2)GPI)(B2GPI)(Apolipoprotein H)(Apo-H)(Activated protein C-binding protein)(APC inhibitor)(Anticardiolipin cofactor) [Source: UniProt P02749]
1972.1.1	-3.3	5.22	1931.915	-0.0013	3	34.25	iqek	286	FKNGMLHGDKVSFFCK	301	nkek	C [300] 57.0215, M [290] 15.9949, N [28	ENSP00000205948	Beta-2-glycoprotein 1 Precursor (Beta-2-glycoprotein I)(Beta(2)GPI)(B2GPI)(Apolipoprotein H)(Apo-H)(Activated protein C-binding protein)(APC inhibitor)(Anticardiolipin cofactor) [Source: UniProt P02749]
1974.1.1	-1.1	3.89	1931.915	-0.0042	2	34.25	iqek	286	FKNGMLHGDKVSFFCK	301	nkek	C [300] 57.0215, M [290] 15.9949, N [28	ENSP00000205948	Beta-2-glycoprotein 1 Precursor (Beta-2-glycoprotein I)(Beta(2)GPI)(B2GPI)(Apolipoprotein H)(Apo-H)(Activated protein C-binding protein)(APC inhibitor)(Anticardiolipin cofactor) [Source: UniProt P02749]
1975.1.1	-1.4	3.65	1656.7517	0.0084	2	31.46	ekfk	288	NGMLHGDKVSFFCK	301	nkek	C [300] 57.0215, M [290] 15.9949, N [28	ENSP00000205948	Beta-2-glycoprotein 1 Precursor (Beta-2-glycoprotein I)(Beta(2)GPI)(B2GPI)(Apolipoprotein H)(Apo-H)(Activated protein C-binding protein)(APC inhibitor)(Anticardiolipin cofactor) [Source: UniProt P02749]
1976.1.1	-3	3.9	1022.5265	0.0041	2	12.58	pvkk	271	ATVVYQGER	279	vkiq		ENSP00000205948	Beta-2-glycoprotein 1 Precursor (Beta-2-glycoprotein I)(Beta(2)GPI)(B2GPI)(Apolipoprotein H)(Apo-H)(Activated protein C-binding protein)(APC inhibitor)(Anticardiolipin cofactor) [Source: UniProt P02749]
1983.1.1	-4.1	4.26	1342.5368	0.0002	2	22.14	ykdk	230	ATFGCHDGYSLD	241	gpee	C [234] 57.0215	ENSP00000205948	Beta-2-glycoprotein 1 Precursor (Beta-2-glycoprotein I)(Beta(2)GPI)(B2GPI)(Apolipoprotein H)(Apo-H)(Activated protein C-binding protein)(APC inhibitor)(Anticardiolipin cofactor) [Source: UniProt P02749]
1987.1.1	-3.4	4.61	1342.5368	0.0006	2	22.14	ykdk	230	ATFGCHDGYSLD	241	gpee	C [234] 57.0215	ENSP00000205948	Beta-2-glycoprotein 1 Precursor (Beta-2-glycoprotein I)(Beta(2)GPI)(B2GPI)(Apolipoprotein H)(Apo-H)(Activated protein C-binding protein)(APC inhibitor)(Anticardiolipin cofactor) [Source: UniProt P02749]
1991.1.1	-2.8	3.97	1129.4731	0.0005	2	13.67	revk	205	CPFPSRPDNG	214	fvny	C [205] 57.0215, C [205] -17.0265	ENSP00000205948	Beta-2-glycoprotein 1 Precursor (Beta-2-glycoprotein I)(Beta(2)GPI)(B2GPI)(Apolipoprotein H)(Apo-H)(Activated protein C-binding protein)(APC inhibitor)(Anticardiolipin cofactor) [Source: UniProt P02749]
1993.1.1	-3.6	4.76	1342.5368	-0.0002	2	22.14	ykdk	230	ATFGCHDGYSLD	241	gpee	C [234] 57.0215	ENSP00000205948	Beta-2-glycoprotein 1 Precursor (Beta-2-glycoprotein I)(Beta(2)GPI)(B2GPI)(Apolipoprotein H)(Apo-H)(Activated protein C-binding protein)(APC inhibitor)(Anticardiolipin cofactor) [Source: UniProt P02749]
1998.1.1	-1.5	3.7	1022.5265	0.0021	2	12.58	pvkk	271	ATVVYQGER	279	vkiq		ENSP00000205948	Beta-2-glycoprotein 1 Precursor (Beta-2-glycoprotein I)(Beta(2)GPI)(B2GPI)(Apolipoprotein H)(Apo-H)(Activated protein C-binding protein)(APC inhibitor)(Anticardiolipin cofactor) [Source: UniProt P02749]
2001.1.1	-1.9	4.12	1656.7517	-0.0052	3	31.46	ekfk	288	NGMLHGDKVSFFCK	301	nkek	C [300] 57.0215, M [290] 15.9949, N [28	ENSP00000205948	Beta-2-glycoprotein 1 Precursor (Beta-2-glycoprotein I)(Beta(2)GPI)(B2GPI)(Apolipoprotein H)(Apo-H)(Activated protein C-binding protein)(APC inhibitor)(Anticardiolipin cofactor) [Source: UniProt P02749]
2003.1.1	-2.5	3.73	1022.5265	0.0008	2	12.58	pvkk	271	ATVVYQGER	279	vkiq		ENSP00000205948	Beta-2-glycoprotein 1 Precursor (Beta-2-glycoprotein I)(Beta(2)GPI)(B2GPI)(Apolipoprotein H)(Apo-H)(Activated protein C-binding protein)(APC inhibitor)(Anticardiolipin cofactor) [Source: UniProt P02749]
2008.1.1	-2.2	3.42	3514.3897	-0.0091	3	2.79	lleg	456	EGSSGGGGRGGGSFGG	500	ggss	H [487] 26.0044	ENSP00000269576	Keratin, type I cytoskeletal 10 (Cytokeratin-10) (CK-10) (Keratin-10) (K10). Source: Uniprot/SWISSPROT P13645
2009.1.1	-4.4	4.27	2214.0053	0.0024	3	26.94	nkek	306	KCSYTEDAQCIDGTIEVI	324	cfke	C [307] 57.0215, C [315] 57.0215	ENSP00000205948	Beta-2-glycoprotein 1 Precursor (Beta-2-glycoprotein I)(Beta(2)GPI)(B2GPI)(Apolipoprotein H)(Apo-H)(Activated protein C-binding protein)(APC inhibitor)(Anticardiolipin cofactor) [Source: UniProt P02749]
2014.1.1	-6.5	4.56	2214.0053	-0.0019	3	26.94	nkek	306	KCSYTEDAQCIDGTIEVI	324	cfke	C [307] 57.0215, C [315] 57.0215	ENSP00000205948	Beta-2-glycoprotein 1 Precursor (Beta-2-glycoprotein I)(Beta(2)GPI)(B2GPI)(Apolipoprotein H)(Apo-H)(Activated protein C-binding protein)(APC inhibitor)(Anticardiolipin cofactor) [Source: UniProt P02749]
2018.1.1	-10	4.8	2214.0053	-0.0022	3	26.94	nkek	306	KCSYTEDAQCIDGTIEVI	324	cfke	C [307] 57.0215, C [315] 57.0215	ENSP00000205948	Beta-2-glycoprotein 1 Precursor (Beta-2-glycoprotein I)(Beta(2)GPI)(B2GPI)(Apolipoprotein H)(Apo-H)(Activated protein C-binding protein)(APC inhibitor)(Anticardiolipin cofactor) [Source: UniProt P02749]
2019.1.1	-2	4.67	1656.7517	-0.0002	3	31.46	ekfk	288	NGMLHGDKVSFFCK	301	nkek	C [300] 57.0215, M [290] 15.9949, N [28	ENSP00000205948	Beta-2-glycoprotein 1 Precursor (Beta-2-glycoprotein I)(Beta(2)GPI)(B2GPI)(Apolipoprotein H)(Apo-H)(Activated protein C-binding protein)(APC inhibitor)(Anticardiolipin cofactor) [Source: UniProt P02749]
2025.1.1	-5.2	5.25	1656.7517	-0.0008	3	31.46	ekfk	288	NGMLHGDKVSFFCK	301	nkek	C [300] 57.0215, M [290] 15.9949, N [28	ENSP00000205948	Beta-2-glycoprotein 1 Precursor (Beta-2-glycoprotein I)(Beta(2)GPI)(B2GPI)(Apolipoprotein H)(Apo-H)(Activated protein C-binding protein)(APC inhibitor)(Anticardiolipin cofactor) [Source: UniProt P02749]
2030.1.1	-4.4	3.85	2214.0053	-0.0092	2	26.94	nkek	306	KCSYTEDAQCIDGTIEVI	324	cfke	C [307] 57.0215, C [315] 57.0215	ENSP00000205948	Beta-2-glycoprotein 1 Precursor (Beta-2-glycoprotein I)(Beta(2)GPI)(B2GPI)(Apolipoprotein H)(Apo-H)(Activated protein C-binding protein)(APC inhibitor)(Anticardiolipin cofactor) [Source: UniProt P02749]
2032.1.1	-1.1	5.46	787.3807	0.0012	2	19.77	hgdk	296	VSFFCK	301	nkek	C [300] 57.0215	ENSP00000205948	Beta-2-glycoprotein 1 Precursor (Beta-2-glycoprotein I)(Beta(2)GPI)(B2GPI)(Apolipoprotein H)(Apo-H)(Activated protein C-binding protein)(APC inhibitor)(Anticardiolipin cofactor) [Source: UniProt P02749]
2034.1.1	-7	4.32	1656.7517	0.0008	2	31.46	ekfk	288	NGMLHGDKVSFFCK	301	nkek	C [300] 57.0215, M [290] 15.9949, N [28	ENSP00000205948	Beta-2-glycoprotein 1 Precursor (Beta-2-glycoprotein I)(Beta(2)GPI)(B2GPI)(Apolipoprotein H)(Apo-H)(Activated protein C-binding protein)(APC inhibitor)(Anticardiolipin cofactor) [Source: UniProt P02749]
2035.1.1	-1.5	4.61	1479.7664	0.0003	3	32.78	gpck	37	MIKPFFHSLSEK	48	ysnv	M [37] 15.9949	ENSP00000363641	Thioredoxin (Trx)(ATL-derived factor)(ADF)(Surface-associated sulphydryl protein)(SASP) [Source: UniProt P10599]
2041.1.1	-7.5	4.52	1656.7517	-0.0001	2	31.46	ekfk	288	NGMLHGDKVSFFCK	301	nkek	C [300] 57.0215, M [290] 15.9949, N [28	ENSP00000205948	Beta-2-glycoprotein 1 Precursor (Beta-2-glycoprotein I)(Beta(2)GPI)(B2GPI)(Apolipoprotein H)(Apo-H)(Activated protein C-binding protein)(APC inhibitor)(Anticardiolipin cofactor) [Source: UniProt P02749]
2042.1.1	-2.1	4.66	1479.7664	-0.0012	3	32.78	gpck	37	MIKPFFHSLSEK	48	ysnv	M [37] 15.9949	ENSP00000363641	Thioredoxin (Trx)(ATL-derived factor)(ADF)(Surface-associated sulphydryl protein)(SASP) [Source: UniProt P10599]
2046.1.1	-3.7	4.61	1656.7517	-0.0025	2	31.46	ekfk	288	NGMLHGDKVSFFCK	301	nkek	C [300] 57.0215, M [290] 15.9949, N [28	ENSP00000205948	Beta-2-glycoprotein 1 Precursor (Beta-2-glycoprotein I)(Beta(2)GPI)(B2GPI)(Apolipoprotein H)(Apo-H)(Activated protein C-binding protein)(APC inhibitor)(Anticardiolipin cofactor) [Source: UniProt P02749]
2049.1.1	-1.3	4.57	1479.7664	0.0007	3	32.78	gpck	37	MIKPFFHSLSEK	48	ysnv	M [37] 15.9949	ENSP00000363641	Thioredoxin (Trx)(ATL-derived factor)(ADF)(Surface-associated sulphydryl protein)(SASP) [Source: UniProt P10599]
2062.1.1	-4	3.96	1540.6836	-0.0048	2	23.42	chdg	238	YSLDGPEEIECTK	250	lgnw	C [248] 57.0215	ENSP00000205948	Beta-2-glycoprotein 1 Precursor (Beta-2-glycoprotein I)(Beta(2)GPI)(B2GPI)(Apolipoprotein H)(Apo-H)(Activated protein C-binding protein)(APC inhibitor)(Anticardiolipin cofactor) [Source: UniProt P02749]
2063.1.1	-1	4.37	1931.915	-0.0047	4	34.25	iqek	286	FKNGMLHGDKVSFFCK	301	nkek	C [300] 57.0215, M [290] 15.9949, N [28	ENSP00000205948	Beta-2-glycoprotein 1 Precursor (Beta-2-glycoprotein I)(Beta(2)GPI)(B2GPI)(Apolipoprotein H)(Apo-H)(Activated protein C-binding protein)(APC inhibitor)(Anticardiolipin cofactor) [Source: UniProt P02749]
2068.1.1	-3.7	4.09	1540.6836	0.0014	2	23.42	chdg	238	YSLDGPEEIECTK	250	lgnw	C [248] 57.0215	ENSP00000205948	Beta-2-glycoprotein 1 Precursor (Beta-2-glycoprotein I)(Beta(2)GPI)(B2GPI)(Apolipoprotein H)(Apo-H)(Activated protein C-binding protein)(APC inhibitor)(Anticardiolipin cofactor) [Source: UniProt P02749]
2073.1.1	-8.2	4.07	1540.6836	-0.0031	2	23.42	chdg	238	YSLDGPEEIECTK	250	lgnw	C [248] 57.0215	ENSP00000205948	Beta-2-glycoprotein 1 Precursor (Beta-2-glycoprotein I)(Beta(2)GPI)(B2GPI)(Apolipoprotein H)(Apo-H)(Activated protein C-binding protein)(APC inhibitor)(Anticardiolipin cofactor) [Source: UniProt P02749]
2075.1.1	-2.1	4.1	1571.7311	0.0011	3	33.42	evpk	325	CFKEHSSLAFWK	336	tdas	C [325] 57.0215, W [335] 31.9898	ENSP00000205948	Beta-2-glycoprotein 1 Precursor (Beta-2-glycoprotein I)(Beta(2)GPI)(B2GPI)(Apolipoprotein H)(Apo-H)(Activated protein C-binding protein)(APC inhibitor)(Anticardiolipin cofactor) [Source: UniProt P02749]
2077.1.1	-6.2	4.13	1597.7051	-0.0012	2	23.66	gchd	237	GYSLDGPEEIECTK	250	lgnw	C [248] 57.0215	ENSP00000205948	Beta-2-glycoprotein 1 Precursor (Beta-2-glycoprotein I)(Beta(2)GPI)(B2GPI)(Apolipoprotein H)(Apo-H)(Activated protein C-binding protein)(APC inhibitor)(Anticardiolipin cofactor) [Source: UniProt P02749]
2079.1.1	-5.2	4.21	1597.7051	-0.001	2	23.66	gchd	237	GYSLDGPEEIECTK	250	lgnw	C [248] 57.0215	ENSP00000205948	Beta-2-glycoprotein 1 Precursor (Beta-2-glycoprotein I)(Beta(2)GPI)(B2GPI)(Apolipoprotein H)(Apo-H)(Activated protein C-binding protein)(APC inhibitor)(Anticardiolipin cofactor) [Source: UniProt P02749]
2082.1.1	-7.9	4.4	1597.7051	-0.0009	2	23.66	gchd	237	GYSLDGPEEIECTK	250	lgnw	C [248] 57.0215	ENSP00000205948	Beta-2-glycoprotein 1 Precursor (Beta-2-glycoprotein I)(Beta(2)GPI)(B2GPI)(Apolipoprotein H)(Apo-H)(Activated protein C-binding protein)(APC inhibitor)(Anticardiolipin cofactor) [Source: UniProt P02749]
2092.1.1	-1.8	4.12	2629.1181	0.0009	4	28.85	lyyk	228	DKATFGCHDGYSLDGPI	250	lgnw	C [234] 57.0215, C [248] 57.0215	ENSP00000205948	Beta-2-glycoprotein 1 Precursor (Beta-2-glycoprotein I)(Beta(2)GPI)(B2GPI)(Apolipoprotein H)(Apo-H)(Activated protein C-binding protein)(APC inhibitor)(Anticardiolipin cofactor) [Source: UniProt P02749]
2098.1.1	-2.4	4.11	1506.6793	0.0008	2	26.18	revk	205	CPFPSRPDNGFVN	217	ypak	C [205] 57.0215	ENSP00000205948	Beta-2-glycoprotein 1 Precursor (Beta-2-glycoprotein I)(Beta(2)GPI)(B2GPI)(Apolipoprotein H)(Apo-H)(Activated protein C-binding protein)(APC inhibitor)(Anticardiolipin cofactor) [Source: UniProt P02749]
2102.1.1	-2.8	4.86	2214.0053	-0.0026	3	26.94	nkek	306	KCSYTEDAQCIDGTIEVI	324	cfke	C [307] 57.0215, C [315] 57.0215	ENSP00000205948	Beta-2-glycoprotein 1 Precursor (Beta-2-glycoprotein I)(Beta(2)GPI)(B2GPI)(Apolipoprotein H)(Apo-H)(Activated protein C-binding protein)(APC inhibitor)(Anticardiolipin cofactor) [Source: UniProt P02749]
2103.1.1	-3.9	4.67	2629.1181	0.0002	4	28.85	lyyk	228	DKATFGCHDGYSLDGPI	250	lgnw	C [234] 57.0215, C [248] 57.0215	ENSP00000205948	Beta-2-glycoprotein 1 Precursor (Beta-2-glycoprotein I)(Beta(2)GPI)(B2GPI)(Apolipoprotein H)(Apo-H)(Activated protein C-binding protein)(APC inhibitor)(Anticardiolipin cofactor) [Source: UniProt P02749]

2104.1.1	-9.5	4.3	2629.1181	-0.0025	3	28.85	lyyk	228	DKATFGCHDGYSLDGPI	250	lgnw	C [234] 57.0215, C [248] 57.0215	ENSP00000205948	Beta-2-glycoprotein 1 Precursor (Beta-2-glycoprotein I)(Beta(2)GPI)(B2GPI)(Apolipoprotein H)(Apo-H)(Activated protein C-binding protein)(APC inhibitor)(Anticardiolipin cofactor) [Source: UniProt P02749]
2105.1.1	-2.6	4.15	1506.6793	-0.0026	2	26.18	revk	205	CPFPSRPDNGFVN	217	ypak	C [205] 57.0215	ENSP00000205948	Beta-2-glycoprotein 1 Precursor (Beta-2-glycoprotein I)(Beta(2)GPI)(B2GPI)(Apolipoprotein H)(Apo-H)(Activated protein C-binding protein)(APC inhibitor)(Anticardiolipin cofactor) [Source: UniProt P02749]
2107.1.1	-1.4	3.92	2644.125	0.986	3	14.18	hghr	125	EDSSLSENSDEHDHTVG	148	nrie		ENSP00000276369:reversed	
2110.1.1	-3.3	5.11	2629.1181	-0.0035	4	28.85	lyyk	228	DKATFGCHDGYSLDGPI	250	lgnw	C [234] 57.0215, C [248] 57.0215	ENSP00000205948	Beta-2-glycoprotein 1 Precursor (Beta-2-glycoprotein I)(Beta(2)GPI)(B2GPI)(Apolipoprotein H)(Apo-H)(Activated protein C-binding protein)(APC inhibitor)(Anticardiolipin cofactor) [Source: UniProt P02749]
2111.1.1	-10.1	4.66	2629.1181	0.0027	3	28.85	lyyk	228	DKATFGCHDGYSLDGPI	250	lgnw	C [234] 57.0215, C [248] 57.0215	ENSP00000205948	Beta-2-glycoprotein 1 Precursor (Beta-2-glycoprotein I)(Beta(2)GPI)(B2GPI)(Apolipoprotein H)(Apo-H)(Activated protein C-binding protein)(APC inhibitor)(Anticardiolipin cofactor) [Source: UniProt P02749]
2114.1.1	-9.2	4.93	2629.1181	0.003	3	28.85	lyyk	228	DKATFGCHDGYSLDGPI	250	lgnw	C [234] 57.0215, C [248] 57.0215	ENSP00000205948	Beta-2-glycoprotein 1 Precursor (Beta-2-glycoprotein I)(Beta(2)GPI)(B2GPI)(Apolipoprotein H)(Apo-H)(Activated protein C-binding protein)(APC inhibitor)(Anticardiolipin cofactor) [Source: UniProt P02749]
2121.1.1	-1.2	4.54	787.3807	0.001	2	19.77	hgdk	296	VSFFCK	301	nkek	C [300] 57.0215	ENSP00000205948	Beta-2-glycoprotein 1 Precursor (Beta-2-glycoprotein I)(Beta(2)GPI)(B2GPI)(Apolipoprotein H)(Apo-H)(Activated protein C-binding protein)(APC inhibitor)(Anticardiolipin cofactor) [Source: UniProt P02749]
2122.1.1	-4	4.53	1965.9275	-0.0015	3	30.03	revk	205	CPFPSRPDNGFVNYPAK	221	ptly	C [205] 57.0215	ENSP00000205948	Beta-2-glycoprotein 1 Precursor (Beta-2-glycoprotein I)(Beta(2)GPI)(B2GPI)(Apolipoprotein H)(Apo-H)(Activated protein C-binding protein)(APC inhibitor)(Anticardiolipin cofactor) [Source: UniProt P02749]
2142.1.1	-2.9	4.79	1965.9275	-0.0017	3	30.03	revk	205	CPFPSRPDNGFVNYPAK	221	ptly	C [205] 57.0215	ENSP00000205948	Beta-2-glycoprotein 1 Precursor (Beta-2-glycoprotein I)(Beta(2)GPI)(B2GPI)(Apolipoprotein H)(Apo-H)(Activated protein C-binding protein)(APC inhibitor)(Anticardiolipin cofactor) [Source: UniProt P02749]
2148.1.1	-5.1	4.95	1965.9275	-0.0032	3	30.03	revk	205	CPFPSRPDNGFVNYPAK	221	ptly	C [205] 57.0215	ENSP00000205948	Beta-2-glycoprotein 1 Precursor (Beta-2-glycoprotein I)(Beta(2)GPI)(B2GPI)(Apolipoprotein H)(Apo-H)(Activated protein C-binding protein)(APC inhibitor)(Anticardiolipin cofactor) [Source: UniProt P02749]
2150.1.1	-10.1	4.72	2214.0053	0.0055	2	26.94	nkek	306	KCSYTEDAQCIDGTIEVI	324	cfke	C [307] 57.0215, C [315] 57.0215	ENSP00000205948	Beta-2-glycoprotein 1 Precursor (Beta-2-glycoprotein I)(Beta(2)GPI)(B2GPI)(Apolipoprotein H)(Apo-H)(Activated protein C-binding protein)(APC inhibitor)(Anticardiolipin cofactor) [Source: UniProt P02749]
2154.1.1	-10.2	5.06	2214.0053	-0.0014	2	26.94	nkek	306	KCSYTEDAQCIDGTIEVI	324	cfke	C [307] 57.0215, C [315] 57.0215	ENSP00000205948	Beta-2-glycoprotein 1 Precursor (Beta-2-glycoprotein I)(Beta(2)GPI)(B2GPI)(Apolipoprotein H)(Apo-H)(Activated protein C-binding protein)(APC inhibitor)(Anticardiolipin cofactor) [Source: UniProt P02749]
2155.1.1	-2.1	4.6	1639.7719	0.0016	3	31.46	ekfk	288	NGMLHGDKVSFFCK	301	nkek	C [300] 57.0215	ENSP00000205948	Beta-2-glycoprotein 1 Precursor (Beta-2-glycoprotein I)(Beta(2)GPI)(B2GPI)(Apolipoprotein H)(Apo-H)(Activated protein C-binding protein)(APC inhibitor)(Anticardiolipin cofactor) [Source: UniProt P02749]
2158.1.1	-7.3	5.25	2214.0053	-0.0036	2	26.94	nkek	306	KCSYTEDAQCIDGTIEVI	324	cfke	C [307] 57.0215, C [315] 57.0215	ENSP00000205948	Beta-2-glycoprotein 1 Precursor (Beta-2-glycoprotein I)(Beta(2)GPI)(B2GPI)(Apolipoprotein H)(Apo-H)(Activated protein C-binding protein)(APC inhibitor)(Anticardiolipin cofactor) [Source: UniProt P02749]
2159.1.1	-2.4	4.62	1639.7719	0.0018	3	31.46	ekfk	288	NGMLHGDKVSFFCK	301	nkek	C [300] 57.0215	ENSP00000205948	Beta-2-glycoprotein 1 Precursor (Beta-2-glycoprotein I)(Beta(2)GPI)(B2GPI)(Apolipoprotein H)(Apo-H)(Activated protein C-binding protein)(APC inhibitor)(Anticardiolipin cofactor) [Source: UniProt P02749]
2165.1.1	-1.4	4.38	1639.7719	-0.0004	3	31.46	ekfk	288	NGMLHGDKVSFFCK	301	nkek	C [300] 57.0215	ENSP00000205948	Beta-2-glycoprotein 1 Precursor (Beta-2-glycoprotein I)(Beta(2)GPI)(B2GPI)(Apolipoprotein H)(Apo-H)(Activated protein C-binding protein)(APC inhibitor)(Anticardiolipin cofactor) [Source: UniProt P02749]
2171.1.1	-5.7	4.29	1712.732	0.0036	2	24.13	fgch	236	DGYSLDGPEEIECTK	250	lgnw	C [248] 57.0215	ENSP00000205948	Beta-2-glycoprotein 1 Precursor (Beta-2-glycoprotein I)(Beta(2)GPI)(B2GPI)(Apolipoprotein H)(Apo-H)(Activated protein C-binding protein)(APC inhibitor)(Anticardiolipin cofactor) [Source: UniProt P02749]
2175.1.1	-10	4.42	1712.732	0.0024	2	24.13	fgch	236	DGYSLDGPEEIECTK	250	lgnw	C [248] 57.0215	ENSP00000205948	Beta-2-glycoprotein 1 Precursor (Beta-2-glycoprotein I)(Beta(2)GPI)(B2GPI)(Apolipoprotein H)(Apo-H)(Activated protein C-binding protein)(APC inhibitor)(Anticardiolipin cofactor) [Source: UniProt P02749]
2181.1.1	-8.4	4.47	1712.732	0.0002	2	24.13	fgch	236	DGYSLDGPEEIECTK	250	lgnw	C [248] 57.0215	ENSP00000205948	Beta-2-glycoprotein 1 Precursor (Beta-2-glycoprotein I)(Beta(2)GPI)(B2GPI)(Apolipoprotein H)(Apo-H)(Activated protein C-binding protein)(APC inhibitor)(Anticardiolipin cofactor) [Source: UniProt P02749]
2186.1.1	-8.2	4.16	2385.9962	-0.0005	3	29.48	ykdk	230	ATFGCHDGYSLDGPEEI	250	lgnw	C [234] 57.0215, C [248] 57.0215	ENSP00000205948	Beta-2-glycoprotein 1 Precursor (Beta-2-glycoprotein I)(Beta(2)GPI)(B2GPI)(Apolipoprotein H)(Apo-H)(Activated protein C-binding protein)(APC inhibitor)(Anticardiolipin cofactor) [Source: UniProt P02749]
2195.1.1	-5.4	4.94	2214.0053	-0.0041	3	26.94	nkek	306	KCSYTEDAQCIDGTIEVI	324	cfke	C [307] 57.0215, C [315] 57.0215	ENSP00000205948	Beta-2-glycoprotein 1 Precursor (Beta-2-glycoprotein I)(Beta(2)GPI)(B2GPI)(Apolipoprotein H)(Apo-H)(Activated protein C-binding protein)(APC inhibitor)(Anticardiolipin cofactor) [Source: UniProt P02749]
2197.1.1	-7.5	4.84	2385.9962	-0.0018	3	29.48	ykdk	230	ATFGCHDGYSLDGPEEI	250	lgnw	C [234] 57.0215, C [248] 57.0215	ENSP00000205948	Beta-2-glycoprotein 1 Precursor (Beta-2-glycoprotein I)(Beta(2)GPI)(B2GPI)(Apolipoprotein H)(Apo-H)(Activated protein C-binding protein)(APC inhibitor)(Anticardiolipin cofactor) [Source: UniProt P02749]
2202.1.1	-8	5.08	2385.9962	-0.0009	3	29.48	ykdk	230	ATFGCHDGYSLDGPEEI	250	lgnw	C [234] 57.0215, C [248] 57.0215	ENSP00000205948	Beta-2-glycoprotein 1 Precursor (Beta-2-glycoprotein I)(Beta(2)GPI)(B2GPI)(Apolipoprotein H)(Apo-H)(Activated protein C-binding protein)(APC inhibitor)(Anticardiolipin cofactor) [Source: UniProt P02749]
2204.1.1	-4.9	4.74	2214.0053	-0.0037	3	26.94	nkek	306	KCSYTEDAQCIDGTIEVI	324	cfke	C [307] 57.0215, C [315] 57.0215	ENSP00000205948	Beta-2-glycoprotein 1 Precursor (Beta-2-glycoprotein I)(Beta(2)GPI)(B2GPI)(Apolipoprotein H)(Apo-H)(Activated protein C-binding protein)(APC inhibitor)(Anticardiolipin cofactor) [Source: UniProt P02749]
2210.1.1	-5.4	4.67	2214.0053	-0.0052	3	26.94	nkek	306	KCSYTEDAQCIDGTIEVI	324	cfke	C [307] 57.0215, C [315] 57.0215	ENSP00000205948	Beta-2-glycoprotein 1 Precursor (Beta-2-glycoprotein I)(Beta(2)GPI)(B2GPI)(Apolipoprotein H)(Apo-H)(Activated protein C-binding protein)(APC inhibitor)(Anticardiolipin cofactor) [Source: UniProt P02749]
2217.1.1	-15.9	4.66	2385.9962	0.0043	2	29.48	ykdk	230	ATFGCHDGYSLDGPEEI	250	lgnw	C [234] 57.0215, C [248] 57.0215	ENSP00000205948	Beta-2-glycoprotein 1 Precursor (Beta-2-glycoprotein I)(Beta(2)GPI)(B2GPI)(Apolipoprotein H)(Apo-H)(Activated protein C-binding protein)(APC inhibitor)(Anticardiolipin cofactor) [Source: UniProt P02749]
2223.1.1	-12	5.07	2385.9962	0.0012	2	29.48	ykdk	230	ATFGCHDGYSLDGPEEI	250	lgnw	C [234] 57.0215, C [248] 57.0215	ENSP00000205948	Beta-2-glycoprotein 1 Precursor (Beta-2-glycoprotein I)(Beta(2)GPI)(B2GPI)(Apolipoprotein H)(Apo-H)(Activated protein C-binding protein)(APC inhibitor)(Anticardiolipin cofactor) [Source: UniProt P02749]
2225.1.1	-12.9	4.47	2085.9103	-0.0021	2	27.19	kekk	307	CSYTEDAQCIDGTIEVPI	324	cfke	C [307] 57.0215, C [315] 57.0215	ENSP00000205948	Beta-2-glycoprotein 1 Precursor (Beta-2-glycoprotein I)(Beta(2)GPI)(B2GPI)(Apolipoprotein H)(Apo-H)(Activated protein C-binding protein)(APC inhibitor)(Anticardiolipin cofactor) [Source: UniProt P02749]
2230.1.1	-10.4	5.22	2385.9962	0.0036	2	29.48	ykdk	230	ATFGCHDGYSLDGPEEI	250	lgnw	C [234] 57.0215, C [248] 57.0215	ENSP00000205948	Beta-2-glycoprotein 1 Precursor (Beta-2-glycoprotein I)(Beta(2)GPI)(B2GPI)(Apolipoprotein H)(Apo-H)(Activated protein C-binding protein)(APC inhibitor)(Anticardiolipin cofactor) [Source: UniProt P02749]
2231.1.1	-13.2	4.65	2085.9103	0.0023	2	27.19	kekk	307	CSYTEDAQCIDGTIEVPI	324	cfke	C [307] 57.0215, C [315] 57.0215	ENSP00000205948	Beta-2-glycoprotein 1 Precursor (Beta-2-glycoprotein I)(Beta(2)GPI)(B2GPI)(Apolipoprotein H)(Apo-H)(Activated protein C-binding protein)(APC inhibitor)(Anticardiolipin cofactor) [Source: UniProt P02749]
2238.1.1	-9.6	4.53	2085.9103	0.0067	2	27.19	kekk	307	CSYTEDAQCIDGTIEVPI	324	cfke	C [307] 57.0215, C [315] 57.0215	ENSP00000205948	Beta-2-glycoprotein 1 Precursor (Beta-2-glycoprotein I)(Beta(2)GPI)(B2GPI)(Apolipoprotein H)(Apo-H)(Activated protein C-binding protein)(APC inhibitor)(Anticardiolipin cofactor) [Source: UniProt P02749]
2239.1.1	-2.2	5.02	1539.7413	0.0032	3	33.42	evpk	325	CFKEHSSLAFWK	336	tdas	C [325] 57.0215	ENSP00000205948	Beta-2-glycoprotein 1 Precursor (Beta-2-glycoprotein I)(Beta(2)GPI)(B2GPI)(Apolipoprotein H)(Apo-H)(Activated protein C-binding protein)(APC inhibitor)(Anticardiolipin cofactor) [Source: UniProt P02749]
2241.1.1	-2.1	5.34	1539.7413	0.0003	3	33.42	evpk	325	CFKEHSSLAFWK	336	tdas	C [325] 57.0215	ENSP00000205948	Beta-2-glycoprotein 1 Precursor (Beta-2-glycoprotein I)(Beta(2)GPI)(B2GPI)(Apolipoprotein H)(Apo-H)(Activated protein C-binding protein)(APC inhibitor)(Anticardiolipin cofactor) [Source: UniProt P02749]
2243.1.1	-14.8	4.55	1723.7521	0.0026	2	27.37	vplk	39	TFYEPGEEITYSCK	52	pgyv	C [51] 57.0215	ENSP00000205948	Beta-2-glycoprotein 1 Precursor (Beta-2-glycoprotein I)(Beta(2)GPI)(B2GPI)(Apolipoprotein H)(Apo-H)(Activated protein C-binding protein)(APC inhibitor)(Anticardiolipin cofactor) [Source: UniProt P02749]
2244.1.1	-3.9	4.47	1316.5939	0.0008	2	26.44	slyr	168	DTAVFECLPQH	178	amfg	C [174] 57.0215	ENSP00000205948	Beta-2-glycoprotein 1 Precursor (Beta-2-glycoprotein I)(Beta(2)GPI)(B2GPI)(Apolipoprotein H)(Apo-H)(Activated protein C-binding protein)(APC inhibitor)(Anticardiolipin cofactor) [Source: UniProt P02749]
2246.1.1	-2.1	5.69	1539.7413	0.0015	3	33.42	evpk	325	CFKEHSSLAFWK	336	tdas	C [325] 57.0215	ENSP00000205948	Beta-2-glycoprotein 1 Precursor (Beta-2-glycoprotein I)(Beta(2)GPI)(B2GPI)(Apolipoprotein H)(Apo-H)(Activated protein C-binding protein)(APC inhibitor)(Anticardiolipin cofactor) [Source: UniProt P02749]
2247.1.1	-2.1	4.95	2425.1017	-0.0005	3	33.69	vplk	39	TFYEPGEEITYSCKPGYV	58	ggmr	C [51] 57.0215, T [39] 42.0106	ENSP00000205948	Beta-2-glycoprotein 1 Precursor (Beta-2-glycoprotein I)(Beta(2)GPI)(B2GPI)(Apolipoprotein H)(Apo-H)(Activated protein C-binding protein)(APC inhibitor)(Anticardiolipin cofactor) [Source: UniProt P02749]
2252.1.1	-13.7	5.27	1723.7521	0.0019	2	27.37	vplk	39	TFYEPGEEITYSCK	52	pgyv	C [51] 57.0215	ENSP00000205948	Beta-2-glycoprotein 1 Precursor (Beta-2-glycoprotein I)(Beta(2)GPI)(B2GPI)(Apolipoprotein H)(Apo-H)(Activated protein C-binding protein)(APC inhibitor)(Anticardiolipin cofactor) [Source: UniProt P02749]
2254.1.1	-5.6	4.47	1539.7413	-0.0006	2	33.42	evpk	325	CFKEHSSLAFWK	336	tdas	C [325] 57.0215	ENSP00000205948	Beta-2-glycoprotein 1 Precursor (Beta-2-glycoprotein I)(Beta(2)GPI)(B2GPI)(Apolipoprotein H)(Apo-H)(Activated protein C-binding protein)(APC inhibitor)(Anticardiolipin cofactor) [Source: UniProt P02749]
2261.1.1	-6.7	4.48	1539.7413	-0.006	2	33.42	evpk	325	CFKEHSSLAFWK	336	tdas	C [325] 57.0215	ENSP00000205948	Beta-2-glycoprotein 1 Precursor (Beta-2-glycoprotein I)(Beta(2)GPI)(B2GPI)(Apolipoprotein H)(Apo-H)(Activated protein C-binding protein)(APC inhibitor)(Anticardiolipin cofactor) [Source: UniProt P02749]
2278.1.1	-1.4	4.82	1104.5473	0.0022	3	27.81	kcfk	328	EHSSLAFWK	336	tdas		ENSP00000205948	Beta-2-glycoprotein 1 Precursor (Beta-2-glycoprotein I)(Beta(2)GPI)(B2GPI)(Apolipoprotein H)(Apo-H)(Activated protein C-binding protein)(APC inhibitor)(Anticardiolipin cofactor) [Source: UniProt P02749]
2280.1.1	-1.4	5.31	1104.5473	0.001	3	27.81	kcfk	328	EHSSLAFWK	336	tdas		ENSP00000205948	Beta-2-glycoprotein 1 Precursor (Beta-2-glycoprotein I)(Beta(2)GPI)(B2GPI)(Apolipoprotein H)(Apo-H)(Activated protein C-binding protein)(APC inhibitor)(Anticardiolipin cofactor) [Source: UniProt P02749]
2285.1.1	-2.1	5.8	1104.5473	0.0017	3	27.81	kcfk	328	EHSSLAFWK	336	tdas		ENSP00000205948	Beta-2-glycoprotein 1 Precursor (Beta-2-glycoprotein I)(Beta(2)GPI)(B2GPI)(Apolipoprotein H)(Apo-H)(Activated protein C-binding protein)(APC inhibitor)(Anticardiolipin cofactor) [Source: UniProt P02749]
2306.1.1	-2.2	5.03	2255.988	0.022	3	38.69	deir	687	EMVDNDLGFQQAPLM	705	tktl	C [702] 57.0215, E [687] -18.0106	ENSP00000269214	No description
2315.1.1	-5.2	4.32	1925.8798	0.0071	2	27.14	ekkc	308	SYTEDAQCIDGTIEVPK	324	cfke	C [315] 57.0215	ENSP00000205948	Beta-2-glycoprotein 1 Precursor (Beta-2-glycoprotein I)(Beta(2)GPI)(B2GPI)(Apolipoprotein H)(Apo-H)(Activated protein C-binding protein)(APC inhibitor)(Anticardiolipin cofactor) [Source: UniProt P02749]
2316.1.1	-2.6	3.91	1660.8734	0.0029	2	32.76	rpdn	214	GFVNYPAKPTLYYK	227	dkat		ENSP00000205948	Beta-2-glycoprotein 1 Precursor (Beta-2-glycoprotein I)(Beta(2)GPI)(B2GPI)(Apolipoprotein H)(Apo-H)(Activated protein C-binding protein)(APC inhibitor)(Anticardiolipin cofactor) [Source: UniProt P02749]
2322.1.1	-1.4	4.43	2256.0218	-0.0037	2	21.04	drar	419	CQPLHHQEPAALSER	433	srev	C [419] 500.199, C [419] 57.0215, C [419	ENSP00000078545	Receptor-type protein-tyrosine phosphatase U precursor (EC 3.1.3.48) (R-PTP-U) (Protein-tyrosine phosphatase J) (PTP-J) (Pancreatic carcinoma phosphatase 2) (PCP-2). [Source: SWISSPROT (Q92729)]
2323.1.1	-6.5	4.23	1925.8798	0.0012	2	27.14	ekkc	308	SYTEDAQCIDGTIEVPK	324	cfke	C [315] 57.0215	ENSP00000205948	Beta-2-glycoprotein 1 Precursor (Beta-2-glycoprotein I)(Beta(2)GPI)(B2GPI)(Apolipoprotein H)(Apo-H)(Activated protein C-binding protein)(APC inhibitor)(Anticardiolipin cofactor) [Source: UniProt P02749]
2330.1.1	-7.2	4.3	2383.0911	-0.0032	2	33.69	vplk	39	TFYEPGEEITYSCKPGYV	58	ggmr	C [51] 57.0215	ENSP00000205948	Beta-2-glycoprotein 1 Precursor (Beta-2-glycoprotein I)(Beta(2)GPI)(B2GPI)(Apolipoprotein H)(Apo-H)(Activated protein C-binding protein)(APC inhibitor)(Anticardiolipin cofactor) [Source: UniProt P02749]
2333.1.1	-3	4.59	2528.1221	-0.0018	4	36.49	evpk	325	CFKEHSSLAFWKTDASD	345	]	C [325] 57.0215, C [345] 57.0215, W [33	ENSP00000205948	Beta-2-glycoprotein 1 Precursor (Beta-2-glycoprotein I)(Beta(2)GPI)(B2GPI)(Apolipoprotein H)(Apo-H)(Activated protein C-binding protein)(APC inhibitor)(Anticardiolipin cofactor) [Source: UniProt P02749]
2340.1.1	-4.9	4.41	2528.1221	0.0001	4	36.49	evpk	325	CFKEHSSLAFWKTDASD	345	]	C [325] 57.0215, C [345] 57.0215, W [33	ENSP00000205948	Beta-2-glycoprotein 1 Precursor (Beta-2-glycoprotein I)(Beta(2)GPI)(B2GPI)(Apolipoprotein H)(Apo-H)(Activated protein C-binding protein)(APC inhibitor)(Anticardiolipin cofactor) [Source: UniProt P02749]
2342.1.1	-6.1	3.94	2085.9103	0.0033	2	27.19	kekk	307	CSYTEDAQCIDGTIEVPI	324	cfke	C [307] 57.0215, C [315] 57.0215	ENSP00000205948	Beta-2-glycoprotein 1 Precursor (Beta-2-glycoprotein I)(Beta(2)GPI)(B2GPI)(Apolipoprotein H)(Apo-H)(Activated protein C-binding protein)(APC inhibitor)(Anticardiolipin cofactor) [Source: UniProt P02749]
2348.1.1	-2.9	4.38	1539.7413	0.0048	3	33.42	evpk	325	CFKEHSSLAFWK	336	tdas	C [325] 57.0215	ENSP00000205948	Beta-2-glycoprotein 1 Precursor (Beta-2-glycoprotein I)(Beta(2)GPI)(B2GPI)(Apolipoprotein H)(Apo-H)(Activated protein C-binding protein)(APC inhibitor)(Anticardiolipin cofactor) [Source: UniProt P02749]
2349.1.1	-6.7	3.82	1723.7521	0.0057	2	27.37	vplk	39	TFYEPGEEITYSCK	52	pgyv	C [51] 57.0215	ENSP00000205948	Beta-2-glycoprotein 1 Precursor (Beta-2-glycoprotein I)(Beta(2)GPI)(B2GPI)(Apolipoprotein H)(Apo-H)(Activated protein C-binding protein)(APC inhibitor)(Anticardiolipin cofactor) [Source: UniProt P02749]
2357.1.1	-3.1	4.16	1986.9961	0.0018	3	34.04	fpsr	211	PDNGFVNYPAKPTLYYK	227	dkat		ENSP00000205948	Beta-2-glycoprotein 1 Precursor (Beta-2-glycoprotein I)(Beta(2)GPI)(B2GPI)(Apolipoprotein H)(Apo-H)(Activated protein C-binding protein)(APC inhibitor)(Anticardiolipin cofactor) [Source: UniProt P02749]
2358.1.1	-7.4	3.99	2383.091	0.021	2	33.69	vplk	39	TFYEPGEEITYSCKPGYV	58	ggmr	C [51] 57.0215	ENSP00000205948	Beta-2-glycoprotein 1 Precursor (Beta-2-glycoprotein I)(Beta(2)GPI)(B2GPI)(Apolipoprotein H)(Apo-H)(Activated protein C-binding protein)(APC inhibitor)(Anticardiolipin cofactor) [Source: UniProt P02749]
2370.1.1	-11.9	4.53	2085.9103	-0.0014	2	27.19	kekk	307	CSYTEDAQCIDGTIEVPI	324	cfke	C [307] 57.0215, C [315] 57.0215	ENSP00000205948	Beta-2-glycoprotein 1 Precursor (Beta-2-glycoprotein I)(Beta(2)GPI)(B2GPI)(Apolipoprotein H)(Apo-H)(Activated protein C-binding protein)(APC inhibitor)(Anticardiolipin cofactor) [Source: UniProt P02749]
2371.1.1	-2	4.06	2383.0911	0.0042	2	33.69	vplk	39	TFYEPGEEITYSCKPGYV	58	ggmr	C [51] 57.0215	ENSP00000205948	Beta-2-glycoprotein 1 Precursor (Beta-2-glycoprotein I)(Beta(2)GPI)(B2GPI)(Apolipoprotein H)(Apo-H)(Activated protein C-binding protein)(APC inhibitor)(Anticardiolipin cofactor) [Source: UniProt P02749]
2373.1.1	-10.4	5	2085.9103	-0.0004	2	27.19	kekk	307	CSYTEDAQCIDGTIEVPI	324	cfke	C [307] 57.0215, C [315] 57.0215	ENSP00000205948	Beta-2-glycoprotein 1 Precursor (Beta-2-glycoprotein I)(Beta(2)GPI)(B2GPI)(Apolipoprotein H)(Apo-H)(Activated protein C-binding protein)(APC inhibitor)(Anticardiolipin cofactor) [Source: UniProt P02749]
2380.1.1	-10.5	5.25	2085.9103	-0.0022	3	27.19	kekk	307	CSYTEDAQCIDGTIEVPI	324	cfke	C [307] 57.0215, C [315] 57.0215	ENSP00000205948	Beta-2-glycoprotein 1 Precursor (Beta-2-glycoprotein I)(Beta(2)GPI)(B2GPI)(Apolipoprotein H)(Apo-H)(Activated protein C-binding protein)(APC inhibitor)(Anticardiolipin cofactor) [Source: UniProt P02749]
2383.1.1	-5.7	4.69	1640.7567	-0.0001	3	31.46	ekfk	288	NGMLHGDKVSFFCK	301	nkek	C [300] 57.0215, N [288] 0.9848	ENSP00000205948	Beta-2-glycoprotein 1 Precursor (Beta-2-glycoprotein I)(Beta(2)GPI)(B2GPI)(Apolipoprotein H)(Apo-H)(Activated protein C-binding protein)(APC inhibitor)(Anticardiolipin cofactor) [Source: UniProt P02749]
2386.1.1	-8.2	5.44	2085.9103	-0.0018	3	27.19	kekk	307	CSYTEDAQCIDGTIEVPI	324	cfke	C [307] 57.0215, C [315] 57.0215	ENSP00000205948	Beta-2-glycoprotein 1 Precursor (Beta-2-glycoprotein I)(Beta(2)GPI)(B2GPI)(Apolipoprotein H)(Apo-H)(Activated protein C-binding protein)(APC inhibitor)(Anticardiolipin cofactor) [Source: UniProt P02749]
2388.1.1	-2.2	4.76	1640.7567	0.0004	3	31.46	ekfk	288	NGMLHGDKVSFFCK	301	nkek	C [300] 57.0215, N [288] 0.9848	ENSP00000205948	Beta-2-glycoprotein 1 Precursor (Beta-2-glycoprotein I)(Beta(2)GPI)(B2GPI)(Apolipoprotein H)(Apo-H)(Activated protein C-binding protein)(APC inhibitor)(Anticardiolipin cofactor) [Source: UniProt P02749]
2392.1.1	-8.6	5.45	2085.9103	-0.0013	3	27.19	kekk	307	CSYTEDAQCIDGTIEVPI	324	cfke	C [307] 57.0215, C [315] 57.0215	ENSP00000205948	Beta-2-glycoprotein 1 Precursor (Beta-2-glycoprotein I)(Beta(2)GPI)(B2GPI)(Apolipoprotein H)(Apo-H)(Activated protein C-binding protein)(APC inhibitor)(Anticardiolipin cofactor) [Source: UniProt P02749]
2395.1.1	-3.7	4.5	1640.7567	-0.0014	3	31.46	ekfk	288	NGMLHGDKVSFFCK	301	nkek	C [300] 57.0215, N [288] 0.9848	ENSP00000205948	Beta-2-glycoprotein 1 Precursor (Beta-2-glycoprotein I)(Beta(2)GPI)(B2GPI)(Apolipoprotein H)(Apo-H)(Activated protein C-binding protein)(APC inhibitor)(Anticardiolipin cofactor) [Source: UniProt P02749]
2397.1.1	-1.4	4.35	813.3989	-0.0037	2	9.81	qcrk	418	ETEDLPK	424	flsc	E [418] -18.0106	ENSP00000284690:reversed	
2411.1.1	-1.6	4.26	1104.5473	0.0014	3	27.81	kcfk	328	EHSSLAFWK	336	tdas		ENSP00000205948	Beta-2-glycoprotein 1 Precursor (Beta-2-glycoprotein I)(Beta(2)GPI)(B2GPI)(Apolipoprotein H)(Apo-H)(Activated protein C-binding protein)(APC inhibitor)(Anticardiolipin cofactor) [Source: UniProt P02749]
2425.1.1	-2	4.26	1104.5473	0.0001	3	27.81	kcfk	328	EHSSLAFWK	336	tdas		ENSP00000205948	Beta-2-glycoprotein 1 Precursor (Beta-2-glycoprotein I)(Beta(2)GPI)(B2GPI)(Apolipoprotein H)(Apo-H)(Activated protein C-binding protein)(APC inhibitor)(Anticardiolipin cofactor) [Source: UniProt P02749]
2455.1.1	-3.4	5.31	2731.3337	-0.0013	4	38.86	revk	205	CPFPSRPDNGFVNYPAK	227	dkat	C [205] 57.0215	ENSP00000205948	Beta-2-glycoprotein 1 Precursor (Beta-2-glycoprotein I)(Beta(2)GPI)(B2GPI)(Apolipoprotein H)(Apo-H)(Activated protein C-binding protein)(APC inhibitor)(Anticardiolipin cofactor) [Source: UniProt P02749]
2457.1.1	-7.8	4.71	2731.3337	0.0053	3	38.86	revk	205	CPFPSRPDNGFVNYPAK	227	dkat	C [205] 57.0215	ENSP00000205948	Beta-2-glycoprotein 1 Precursor (Beta-2-glycoprotein I)(Beta(2)GPI)(B2GPI)(Apolipoprotein H)(Apo-H)(Activated protein C-binding protein)(APC inhibitor)(Anticardiolipin cofactor) [Source: UniProt P02749]
2460.1.1	-3.7	4.05	2085.9103	-0.0065	2	27.19	kekk	307	CSYTEDAQCIDGTIEVPI	324	cfke	C [307] 57.0215, C [315] 57.0215	ENSP00000205948	Beta-2-glycoprotein 1 Precursor (Beta-2-glycoprotein I)(Beta(2)GPI)(B2GPI)(Apolipoprotein H)(Apo-H)(Activated protein C-binding protein)(APC inhibitor)(Anticardiolipin cofactor) [Source: UniProt P02749]
2462.1.1	-2	5.9	2731.3337	-0.0006	4	38.86	revk	205	CPFPSRPDNGFVNYPAK	227	dkat	C [205] 57.0215	ENSP00000205948	Beta-2-glycoprotein 1 Precursor (Beta-2-glycoprotein I)(Beta(2)GPI)(B2GPI)(Apolipoprotein H)(Apo-H)(Activated protein C-binding protein)(APC inhibitor)(Anticardiolipin cofactor) [Source: UniProt P02749]
2463.1.1	-5.5	5.26	2731.3337	0.0051	3	38.86	revk	205	CPFPSRPDNGFVNYPAK	227	dkat	C [205] 57.0215	ENSP00000205948	Beta-2-glycoprotein 1 Precursor (Beta-2-glycoprotein I)(Beta(2)GPI)(B2GPI)(Apolipoprotein H)(Apo-H)(Activated protein C-binding protein)(APC inhibitor)(Anticardiolipin cofactor) [Source: UniProt P02749]
2468.1.1	-1.1	6.28	2731.3337	0.0021	4	38.86	revk	205	CPFPSRPDNGFVNYPAK	227	dkat	C [205] 57.0215	ENSP00000205948	Beta-2-glycoprotein 1 Precursor (Beta-2-glycoprotein I)(Beta(2)GPI)(B2GPI)(Apolipoprotein H)(Apo-H)(Activated protein C-binding protein)(APC inhibitor)(Anticardiolipin cofactor) [Source: UniProt P02749]
2469.1.1	-5.4	5.4	2731.3337	0.0075	3	38.86	revk	205	CPFPSRPDNGFVNYPAK	227	dkat	C [205] 57.0215	ENSP00000205948	Beta-2-glycoprotein 1 Precursor (Beta-2-glycoprotein I)(Beta(2)GPI)(B2GPI)(Apolipoprotein H)(Apo-H)(Activated protein C-binding protein)(APC inhibitor)(Anticardiolipin cofactor) [Source: UniProt P02749]
2472.1.1	-7.8	4.31	2086.895	0.011	2	27.19	kekk	307	CSYTEDAQCIDGTIEVPI	324	cfke	C [307] 57.0215, C [315] 57.0215, Q [31	ENSP00000205948	Beta-2-glycoprotein 1 Precursor (Beta-2-glycoprotein I)(Beta(2)GPI)(B2GPI)(Apolipoprotein H)(Apo-H)(Activated protein C-binding protein)(APC inhibitor)(Anticardiolipin cofactor) [Source: UniProt P02749]
2473.1.1	-2.7	4.64	2425.102	0.021	2	33.69	vplk	39	TFYEPGEEITYSCKPGYV	58	ggmr	C [51] 57.0215, T [39] 42.0106	ENSP00000205948	Beta-2-glycoprotein 1 Precursor (Beta-2-glycoprotein I)(Beta(2)GPI)(B2GPI)(Apolipoprotein H)(Apo-H)(Activated protein C-binding protein)(APC inhibitor)(Anticardiolipin cofactor) [Source: UniProt P02749]
2476.1.1	-9	4.46	2086.8951	0.0048	2	27.19	kekk	307	CSYTEDAQCIDGTIEVPI	324	cfke	C [307] 57.0215, C [315] 57.0215, Q [31	ENSP00000205948	Beta-2-glycoprotein 1 Precursor (Beta-2-glycoprotein I)(Beta(2)GPI)(B2GPI)(Apolipoprotein H)(Apo-H)(Activated protein C-binding protein)(APC inhibitor)(Anticardiolipin cofactor) [Source: UniProt P02749]
2480.1.1	-2.7	4.58	2425.102	0.014	2	33.69	vplk	39	TFYEPGEEITYSCKPGYV	58	ggmr	C [51] 57.0215, T [39] 42.0106	ENSP00000205948	Beta-2-glycoprotein 1 Precursor (Beta-2-glycoprotein I)(Beta(2)GPI)(B2GPI)(Apolipoprotein H)(Apo-H)(Activated protein C-binding protein)(APC inhibitor)(Anticardiolipin cofactor) [Source: UniProt P02749]
2489.1.1	-6.1	3.99	1765.7626	0.0033	2	27.37	vplk	39	TFYEPGEEITYSCK	52	pgyv	C [51] 57.0215, T [39] 42.0106	ENSP00000205948	Beta-2-glycoprotein 1 Precursor (Beta-2-glycoprotein I)(Beta(2)GPI)(B2GPI)(Apolipoprotein H)(Apo-H)(Activated protein C-binding protein)(APC inhibitor)(Anticardiolipin cofactor) [Source: UniProt P02749]
2496.1.1	-7.1	4.09	1765.7626	-0.0006	2	27.37	vplk	39	TFYEPGEEITYSCK	52	pgyv	C [51] 57.0215, T [39] 42.0106	ENSP00000205948	Beta-2-glycoprotein 1 Precursor (Beta-2-glycoprotein I)(Beta(2)GPI)(B2GPI)(Apolipoprotein H)(Apo-H)(Activated protein C-binding protein)(APC inhibitor)(Anticardiolipin cofactor) [Source: UniProt P02749]
2498.1.1	-2	5.44	2425.1017	0.0032	3	33.69	vplk	39	TFYEPGEEITYSCKPGYV	58	ggmr	C [51] 57.0215, T [39] 42.0106	ENSP00000205948	Beta-2-glycoprotein 1 Precursor (Beta-2-glycoprotein I)(Beta(2)GPI)(B2GPI)(Apolipoprotein H)(Apo-H)(Activated protein C-binding protein)(APC inhibitor)(Anticardiolipin cofactor) [Source: UniProt P02749]
2501.1.1	-5.5	4.07	1765.7626	0.0037	2	27.37	vplk	39	TFYEPGEEITYSCK	52	pgyv	C [51] 57.0215, T [39] 42.0106	ENSP00000205948	Beta-2-glycoprotein 1 Precursor (Beta-2-glycoprotein I)(Beta(2)GPI)(B2GPI)(Apolipoprotein H)(Apo-H)(Activated protein C-binding protein)(APC inhibitor)(Anticardiolipin cofactor) [Source: UniProt P02749]
2550.1.1	-2.8	6.06	2732.3185	0.001	4	38.86	revk	205	CPFPSRPDNGFVNYPAK	227	dkat	C [205] 57.0215, N [213] 0.9848	ENSP00000205948	Beta-2-glycoprotein 1 Precursor (Beta-2-glycoprotein I)(Beta(2)GPI)(B2GPI)(Apolipoprotein H)(Apo-H)(Activated protein C-binding protein)(APC inhibitor)(Anticardiolipin cofactor) [Source: UniProt P02749]
2552.1.1	-10.8	5.13	2732.3185	0.0062	3	38.86	revk	205	CPFPSRPDNGFVNYPAK	227	dkat	C [205] 57.0215, N [213] 0.9848	ENSP00000205948	Beta-2-glycoprotein 1 Precursor (Beta-2-glycoprotein I)(Beta(2)GPI)(B2GPI)(Apolipoprotein H)(Apo-H)(Activated protein C-binding protein)(APC inhibitor)(Anticardiolipin cofactor) [Source: UniProt P02749]
2555.1.1	-1.1	5.16	2714.3072	0.0003	4	38.86	revk	205	CPFPSRPDNGFVNYPAK	227	dkat	C [205] 57.0215, C [205] -17.0265	ENSP00000205948	Beta-2-glycoprotein 1 Precursor (Beta-2-glycoprotein I)(Beta(2)GPI)(B2GPI)(Apolipoprotein H)(Apo-H)(Activated protein C-binding protein)(APC inhibitor)(Anticardiolipin cofactor) [Source: UniProt P02749]
2557.1.1	-1.4	5.92	2732.3185	-0.0027	4	38.86	revk	205	CPFPSRPDNGFVNYPAK	227	dkat	C [205] 57.0215, N [217] 0.9848	ENSP00000205948	Beta-2-glycoprotein 1 Precursor (Beta-2-glycoprotein I)(Beta(2)GPI)(B2GPI)(Apolipoprotein H)(Apo-H)(Activated protein C-binding protein)(APC inhibitor)(Anticardiolipin cofactor) [Source: UniProt P02749]
2561.1.1	-11.1	5.01	2732.3185	0.003	3	38.86	revk	205	CPFPSRPDNGFVNYPAK	227	dkat	C [205] 57.0215, N [213] 0.9848	ENSP00000205948	Beta-2-glycoprotein 1 Precursor (Beta-2-glycoprotein I)(Beta(2)GPI)(B2GPI)(Apolipoprotein H)(Apo-H)(Activated protein C-binding protein)(APC inhibitor)(Anticardiolipin cofactor) [Source: UniProt P02749]
2564.1.1	-1.3	5.8	2731.334	0.984	4	38.86	revk	205	CPFPSRPDNGFVNYPAK	227	dkat	C [205] 57.0215	ENSP00000205948	Beta-2-glycoprotein 1 Precursor (Beta-2-glycoprotein I)(Beta(2)GPI)(B2GPI)(Apolipoprotein H)(Apo-H)(Activated protein C-binding protein)(APC inhibitor)(Anticardiolipin cofactor) [Source: UniProt P02749]
2565.1.1	-11.3	4.98	2732.3185	0.002	3	38.86	revk	205	CPFPSRPDNGFVNYPAK	227	dkat	C [205] 57.0215, N [213] 0.9848	ENSP00000205948	Beta-2-glycoprotein 1 Precursor (Beta-2-glycoprotein I)(Beta(2)GPI)(B2GPI)(Apolipoprotein H)(Apo-H)(Activated protein C-binding protein)(APC inhibitor)(Anticardiolipin cofactor) [Source: UniProt P02749]
2569.1.1	-2.7	4.15	2714.3072	0.0025	3	38.86	revk	205	CPFPSRPDNGFVNYPAK	227	dkat	C [205] 57.0215, C [205] -17.0265	ENSP00000205948	Beta-2-glycoprotein 1 Precursor (Beta-2-glycoprotein I)(Beta(2)GPI)(B2GPI)(Apolipoprotein H)(Apo-H)(Activated protein C-binding protein)(APC inhibitor)(Anticardiolipin cofactor) [Source: UniProt P02749]
2580.1.1	-4.7	3.98	2714.3072	0.0063	3	38.86	revk	205	CPFPSRPDNGFVNYPAK	227	dkat	C [205] 57.0215, C [205] -17.0265	ENSP00000205948	Beta-2-glycoprotein 1 Precursor (Beta-2-glycoprotein I)(Beta(2)GPI)(B2GPI)(Apolipoprotein H)(Apo-H)(Activated protein C-binding protein)(APC inhibitor)(Anticardiolipin cofactor) [Source: UniProt P02749]
2585.1.1	-14.1	4.18	2068.8838	0.0049	2	27.19	kekk	307	CSYTEDAQCIDGTIEVPI	324	cfke	C [307] 57.0215, C [315] 57.0215, C [307	ENSP00000205948	Beta-2-glycoprotein 1 Precursor (Beta-2-glycoprotein I)(Beta(2)GPI)(B2GPI)(Apolipoprotein H)(Apo-H)(Activated protein C-binding protein)(APC inhibitor)(Anticardiolipin cofactor) [Source: UniProt P02749]
2588.1.1	-4.9	3.72	2085.9103	-0.0092	2	27.19	kekk	307	CSYTEDAQCIDGTIEVPI	324	cfke	C [307] 57.0215, C [315] 57.0215	ENSP00000205948	Beta-2-glycoprotein 1 Precursor (Beta-2-glycoprotein I)(Beta(2)GPI)(B2GPI)(Apolipoprotein H)(Apo-H)(Activated protein C-binding protein)(APC inhibitor)(Anticardiolipin cofactor) [Source: UniProt P02749]
2593.1.1	-11.4	4.55	2068.8838	-0.0029	2	27.19	kekk	307	CSYTEDAQCIDGTIEVPI	324	cfke	C [307] 57.0215, C [315] 57.0215, C [307	ENSP00000205948	Beta-2-glycoprotein 1 Precursor (Beta-2-glycoprotein I)(Beta(2)GPI)(B2GPI)(Apolipoprotein H)(Apo-H)(Activated protein C-binding protein)(APC inhibitor)(Anticardiolipin cofactor) [Source: UniProt P02749]
2594.1.1	-5.6	4.44	1948.9009	0.0044	2	30.03	revk	205	CPFPSRPDNGFVNYPAK	221	ptly	C [205] 57.0215, C [205] -17.0265	ENSP00000205948	Beta-2-glycoprotein 1 Precursor (Beta-2-glycoprotein I)(Beta(2)GPI)(B2GPI)(Apolipoprotein H)(Apo-H)(Activated protein C-binding protein)(APC inhibitor)(Anticardiolipin cofactor) [Source: UniProt P02749]
2595.1.1	-2.5	4.52	838.4457	0.0012	2	27.22	fkeh	330	SSLAFWK	336	tdas		ENSP00000205948	Beta-2-glycoprotein 1 Precursor (Beta-2-glycoprotein I)(Beta(2)GPI)(B2GPI)(Apolipoprotein H)(Apo-H)(Activated protein C-binding protein)(APC inhibitor)(Anticardiolipin cofactor) [Source: UniProt P02749]
2596.1.1	-4.6	3.66	2085.9103	0.0021	2	27.19	kekk	307	CSYTEDAQCIDGTIEVPI	324	cfke	C [307] 57.0215, C [315] 57.0215	ENSP00000205948	Beta-2-glycoprotein 1 Precursor (Beta-2-glycoprotein I)(Beta(2)GPI)(B2GPI)(Apolipoprotein H)(Apo-H)(Activated protein C-binding protein)(APC inhibitor)(Anticardiolipin cofactor) [Source: UniProt P02749]
2599.1.1	-11.3	4.7	2068.8838	-0.0005	2	27.19	kekk	307	CSYTEDAQCIDGTIEVPI	324	cfke	C [307] 57.0215, C [315] 57.0215, C [307	ENSP00000205948	Beta-2-glycoprotein 1 Precursor (Beta-2-glycoprotein I)(Beta(2)GPI)(B2GPI)(Apolipoprotein H)(Apo-H)(Activated protein C-binding protein)(APC inhibitor)(Anticardiolipin cofactor) [Source: UniProt P02749]
2600.1.1	-7.6	4.49	1948.9009	0.0006	2	30.03	revk	205	CPFPSRPDNGFVNYPAK	221	ptly	C [205] 57.0215, C [205] -17.0265	ENSP00000205948	Beta-2-glycoprotein 1 Precursor (Beta-2-glycoprotein I)(Beta(2)GPI)(B2GPI)(Apolipoprotein H)(Apo-H)(Activated protein C-binding protein)(APC inhibitor)(Anticardiolipin cofactor) [Source: UniProt P02749]
2601.1.1	-5.2	3.6	2085.9103	0.0057	2	27.19	kekk	307	CSYTEDAQCIDGTIEVPI	324	cfke	C [307] 57.0215, C [315] 57.0215	ENSP00000205948	Beta-2-glycoprotein 1 Precursor (Beta-2-glycoprotein I)(Beta(2)GPI)(B2GPI)(Apolipoprotein H)(Apo-H)(Activated protein C-binding protein)(APC inhibitor)(Anticardiolipin cofactor) [Source: UniProt P02749]

2603.1.1	-2.3	4.83	2957.4291	-0.0056	4	38.61	revk	205	CPFPSRPDNGFVNYPAK	229	atfg	C [205] 57.0215, C [205] -17.0265	ENSP00000205948	Beta-2-glycoprotein 1 Precursor (Beta-2-glycoprotein I)(Beta(2)GPI)(B2GPI)(Apolipoprotein H)(Apo-H)(Activated protein C-binding protein)(APC inhibitor)(Anticardiolipin cofactor) [Source: UniProt P02749]
2606.1.1	-2.6	5.01	2957.4291	0.0012	4	38.61	revk	205	CPFPSRPDNGFVNYPAK	229	atfg	C [205] 57.0215, C [205] -17.0265	ENSP00000205948	Beta-2-glycoprotein 1 Precursor (Beta-2-glycoprotein I)(Beta(2)GPI)(B2GPI)(Apolipoprotein H)(Apo-H)(Activated protein C-binding protein)(APC inhibitor)(Anticardiolipin cofactor) [Source: UniProt P02749]
2607.1.1	-6	4.62	1948.9009	0.0037	2	30.03	revk	205	CPFPSRPDNGFVNYPAK	221	ptly	C [205] 57.0215, C [205] -17.0265	ENSP00000205948	Beta-2-glycoprotein 1 Precursor (Beta-2-glycoprotein I)(Beta(2)GPI)(B2GPI)(Apolipoprotein H)(Apo-H)(Activated protein C-binding protein)(APC inhibitor)(Anticardiolipin cofactor) [Source: UniProt P02749]
2608.1.1	-1.1	4.6	2383.0911	0.0028	3	33.69	vplk	39	TFYEPGEEITYSCKPGYV	58	ggmr	C [51] 57.0215	ENSP00000205948	Beta-2-glycoprotein 1 Precursor (Beta-2-glycoprotein I)(Beta(2)GPI)(B2GPI)(Apolipoprotein H)(Apo-H)(Activated protein C-binding protein)(APC inhibitor)(Anticardiolipin cofactor) [Source: UniProt P02749]
2611.1.1	-3.5	4.26	2957.4291	0.0095	3	38.61	revk	205	CPFPSRPDNGFVNYPAK	229	atfg	C [205] 57.0215, C [205] -17.0265	ENSP00000205948	Beta-2-glycoprotein 1 Precursor (Beta-2-glycoprotein I)(Beta(2)GPI)(B2GPI)(Apolipoprotein H)(Apo-H)(Activated protein C-binding protein)(APC inhibitor)(Anticardiolipin cofactor) [Source: UniProt P02749]
2613.1.1	-4.2	5.21	2957.4291	-0.0046	4	38.61	revk	205	CPFPSRPDNGFVNYPAK	229	atfg	C [205] 57.0215, C [205] -17.0265	ENSP00000205948	Beta-2-glycoprotein 1 Precursor (Beta-2-glycoprotein I)(Beta(2)GPI)(B2GPI)(Apolipoprotein H)(Apo-H)(Activated protein C-binding protein)(APC inhibitor)(Anticardiolipin cofactor) [Source: UniProt P02749]
2615.1.1	-4.2	4.39	2957.4291	0.0073	3	38.61	revk	205	CPFPSRPDNGFVNYPAK	229	atfg	C [205] 57.0215, C [205] -17.0265	ENSP00000205948	Beta-2-glycoprotein 1 Precursor (Beta-2-glycoprotein I)(Beta(2)GPI)(B2GPI)(Apolipoprotein H)(Apo-H)(Activated protein C-binding protein)(APC inhibitor)(Anticardiolipin cofactor) [Source: UniProt P02749]
2618.1.1	-4.6	4.48	1086.5367	0.0003	2	27.81	kcfk	328	EHSSLAFWK	336	tdas	E [328] -18.0106	ENSP00000205948	Beta-2-glycoprotein 1 Precursor (Beta-2-glycoprotein I)(Beta(2)GPI)(B2GPI)(Apolipoprotein H)(Apo-H)(Activated protein C-binding protein)(APC inhibitor)(Anticardiolipin cofactor) [Source: UniProt P02749]
2622.1.1	-5.4	4.27	2957.4291	0.0029	3	38.61	revk	205	CPFPSRPDNGFVNYPAK	229	atfg	C [205] 57.0215, C [205] -17.0265	ENSP00000205948	Beta-2-glycoprotein 1 Precursor (Beta-2-glycoprotein I)(Beta(2)GPI)(B2GPI)(Apolipoprotein H)(Apo-H)(Activated protein C-binding protein)(APC inhibitor)(Anticardiolipin cofactor) [Source: UniProt P02749]
2623.1.1	-4.4	4.52	1086.5367	0.0011	2	27.81	kcfk	328	EHSSLAFWK	336	tdas	E [328] -18.0106	ENSP00000205948	Beta-2-glycoprotein 1 Precursor (Beta-2-glycoprotein I)(Beta(2)GPI)(B2GPI)(Apolipoprotein H)(Apo-H)(Activated protein C-binding protein)(APC inhibitor)(Anticardiolipin cofactor) [Source: UniProt P02749]
2629.1.1	-4.5	4.49	1086.5367	0.0009	2	27.81	kcfk	328	EHSSLAFWK	336	tdas	E [328] -18.0106	ENSP00000205948	Beta-2-glycoprotein 1 Precursor (Beta-2-glycoprotein I)(Beta(2)GPI)(B2GPI)(Apolipoprotein H)(Apo-H)(Activated protein C-binding protein)(APC inhibitor)(Anticardiolipin cofactor) [Source: UniProt P02749]
2634.1.1	-9	5.6	2127.1265	0.0004	3	36.23	vaia	20	GRTCPKPDDLPFSTVVP	38	tfye	C [23] 57.0215	ENSP00000205948	Beta-2-glycoprotein 1 Precursor (Beta-2-glycoprotein I)(Beta(2)GPI)(B2GPI)(Apolipoprotein H)(Apo-H)(Activated protein C-binding protein)(APC inhibitor)(Anticardiolipin cofactor) [Source: UniProt P02749]
2641.1.1	-8.1	5.84	2127.1265	0.001	3	36.23	vaia	20	GRTCPKPDDLPFSTVVP	38	tfye	C [23] 57.0215	ENSP00000205948	Beta-2-glycoprotein 1 Precursor (Beta-2-glycoprotein I)(Beta(2)GPI)(B2GPI)(Apolipoprotein H)(Apo-H)(Activated protein C-binding protein)(APC inhibitor)(Anticardiolipin cofactor) [Source: UniProt P02749]
2648.1.1	-9.4	5.98	2127.1265	0	3	36.23	vaia	20	GRTCPKPDDLPFSTVVP	38	tfye	C [23] 57.0215	ENSP00000205948	Beta-2-glycoprotein 1 Precursor (Beta-2-glycoprotein I)(Beta(2)GPI)(B2GPI)(Apolipoprotein H)(Apo-H)(Activated protein C-binding protein)(APC inhibitor)(Anticardiolipin cofactor) [Source: UniProt P02749]
2655.1.1	-2.2	6.23	2127.1265	0.0028	4	36.23	vaia	20	GRTCPKPDDLPFSTVVP	38	tfye	C [23] 57.0215	ENSP00000205948	Beta-2-glycoprotein 1 Precursor (Beta-2-glycoprotein I)(Beta(2)GPI)(B2GPI)(Apolipoprotein H)(Apo-H)(Activated protein C-binding protein)(APC inhibitor)(Anticardiolipin cofactor) [Source: UniProt P02749]
2658.1.1	-6.6	4.59	2211.104	0.0038	3	36.26	iqvr	58	LGEHNIDVLEGNEQFIN	77	iith		sp|TRYP_PIG|	Trypsin; EC 3.4.21.4; Flags: Precursor;
2660.1.1	-5.4	4.64	2440.1751	0.0037	3	38.48	revk	205	CPFPSRPDNGFVNYPAK	225	ykdk	C [205] 57.0215	ENSP00000205948	Beta-2-glycoprotein 1 Precursor (Beta-2-glycoprotein I)(Beta(2)GPI)(B2GPI)(Apolipoprotein H)(Apo-H)(Activated protein C-binding protein)(APC inhibitor)(Anticardiolipin cofactor) [Source: UniProt P02749]
2663.1.1	-6.8	5.1	2070.105	-0.0043	3	37.03	aiag	21	RTCPKPDDLPFSTVVPLI	38	tfye	C [23] 57.0215	ENSP00000205948	Beta-2-glycoprotein 1 Precursor (Beta-2-glycoprotein I)(Beta(2)GPI)(B2GPI)(Apolipoprotein H)(Apo-H)(Activated protein C-binding protein)(APC inhibitor)(Anticardiolipin cofactor) [Source: UniProt P02749]
2664.1.1	-5.9	4.68	2211.104	-0.0017	3	36.26	iqvr	58	LGEHNIDVLEGNEQFIN	77	iith		sp|TRYP_PIG|	Trypsin; EC 3.4.21.4; Flags: Precursor;
2665.1.1	-5.6	4.71	2440.1751	0.0013	3	38.48	revk	205	CPFPSRPDNGFVNYPAK	225	ykdk	C [205] 57.0215	ENSP00000205948	Beta-2-glycoprotein 1 Precursor (Beta-2-glycoprotein I)(Beta(2)GPI)(B2GPI)(Apolipoprotein H)(Apo-H)(Activated protein C-binding protein)(APC inhibitor)(Anticardiolipin cofactor) [Source: UniProt P02749]
2666.1.1	-9.4	4.67	1179.535	0.0006	2	27.68	slyr	168	DTAVFECLPQ	177	hamf	C [174] 57.0215	ENSP00000205948	Beta-2-glycoprotein 1 Precursor (Beta-2-glycoprotein I)(Beta(2)GPI)(B2GPI)(Apolipoprotein H)(Apo-H)(Activated protein C-binding protein)(APC inhibitor)(Anticardiolipin cofactor) [Source: UniProt P02749]
2669.1.1	-7.1	4.77	2440.1751	0.0033	3	38.48	revk	205	CPFPSRPDNGFVNYPAK	225	ykdk	C [205] 57.0215	ENSP00000205948	Beta-2-glycoprotein 1 Precursor (Beta-2-glycoprotein I)(Beta(2)GPI)(B2GPI)(Apolipoprotein H)(Apo-H)(Activated protein C-binding protein)(APC inhibitor)(Anticardiolipin cofactor) [Source: UniProt P02749]
2670.1.1	-7.7	4.89	2070.105	-0.0054	3	37.03	aiag	21	RTCPKPDDLPFSTVVPLI	38	tfye	C [23] 57.0215	ENSP00000205948	Beta-2-glycoprotein 1 Precursor (Beta-2-glycoprotein I)(Beta(2)GPI)(B2GPI)(Apolipoprotein H)(Apo-H)(Activated protein C-binding protein)(APC inhibitor)(Anticardiolipin cofactor) [Source: UniProt P02749]
2671.1.1	-5.8	4.78	1179.535	-0.0005	2	27.68	slyr	168	DTAVFECLPQ	177	hamf	C [174] 57.0215	ENSP00000205948	Beta-2-glycoprotein 1 Precursor (Beta-2-glycoprotein I)(Beta(2)GPI)(B2GPI)(Apolipoprotein H)(Apo-H)(Activated protein C-binding protein)(APC inhibitor)(Anticardiolipin cofactor) [Source: UniProt P02749]
2672.1.1	-5.7	4.44	2211.104	-0.0028	3	36.26	iqvr	58	LGEHNIDVLEGNEQFIN	77	iith		sp|TRYP_PIG|	Trypsin; EC 3.4.21.4; Flags: Precursor;
2673.1.1	-1.4	4.92	2732.3185	0	4	38.86	revk	205	CPFPSRPDNGFVNYPAK	227	dkat	C [205] 57.0215, N [217] 0.9848	ENSP00000205948	Beta-2-glycoprotein 1 Precursor (Beta-2-glycoprotein I)(Beta(2)GPI)(B2GPI)(Apolipoprotein H)(Apo-H)(Activated protein C-binding protein)(APC inhibitor)(Anticardiolipin cofactor) [Source: UniProt P02749]
2676.1.1	-8.4	4.67	1179.535	0.0003	2	27.68	slyr	168	DTAVFECLPQ	177	hamf	C [174] 57.0215	ENSP00000205948	Beta-2-glycoprotein 1 Precursor (Beta-2-glycoprotein I)(Beta(2)GPI)(B2GPI)(Apolipoprotein H)(Apo-H)(Activated protein C-binding protein)(APC inhibitor)(Anticardiolipin cofactor) [Source: UniProt P02749]
2677.1.1	-8	4.61	2070.105	-0.0001	3	37.03	aiag	21	RTCPKPDDLPFSTVVPLI	38	tfye	C [23] 57.0215	ENSP00000205948	Beta-2-glycoprotein 1 Precursor (Beta-2-glycoprotein I)(Beta(2)GPI)(B2GPI)(Apolipoprotein H)(Apo-H)(Activated protein C-binding protein)(APC inhibitor)(Anticardiolipin cofactor) [Source: UniProt P02749]
2678.1.1	-1.7	4.82	2732.3185	0.0032	4	38.86	revk	205	CPFPSRPDNGFVNYPAK	227	dkat	C [205] 57.0215, N [213] 0.9848	ENSP00000205948	Beta-2-glycoprotein 1 Precursor (Beta-2-glycoprotein I)(Beta(2)GPI)(B2GPI)(Apolipoprotein H)(Apo-H)(Activated protein C-binding protein)(APC inhibitor)(Anticardiolipin cofactor) [Source: UniProt P02749]
2684.1.1	-4.3	4.14	2731.334	-0.016	3	38.86	revk	205	CPFPSRPDNGFVNYPAK	227	dkat	C [205] 57.0215	ENSP00000205948	Beta-2-glycoprotein 1 Precursor (Beta-2-glycoprotein I)(Beta(2)GPI)(B2GPI)(Apolipoprotein H)(Apo-H)(Activated protein C-binding protein)(APC inhibitor)(Anticardiolipin cofactor) [Source: UniProt P02749]
2692.1.1	-3.2	4.31	2731.333	-0.027	3	38.86	revk	205	CPFPSRPDNGFVNYPAK	227	dkat	C [205] 57.0215	ENSP00000205948	Beta-2-glycoprotein 1 Precursor (Beta-2-glycoprotein I)(Beta(2)GPI)(B2GPI)(Apolipoprotein H)(Apo-H)(Activated protein C-binding protein)(APC inhibitor)(Anticardiolipin cofactor) [Source: UniProt P02749]
2693.1.1	-4.2	4.43	2425.1017	0.0066	3	33.69	vplk	39	TFYEPGEEITYSCKPGYV	58	ggmr	C [51] 57.0215, T [39] 42.0106	ENSP00000205948	Beta-2-glycoprotein 1 Precursor (Beta-2-glycoprotein I)(Beta(2)GPI)(B2GPI)(Apolipoprotein H)(Apo-H)(Activated protein C-binding protein)(APC inhibitor)(Anticardiolipin cofactor) [Source: UniProt P02749]
2700.1.1	-4.9	4.58	1914.0041	0.0026	3	36.7	iagr	22	TCPKPDDLPFSTVVPLK	38	tfye	C [23] 57.0215	ENSP00000205948	Beta-2-glycoprotein 1 Precursor (Beta-2-glycoprotein I)(Beta(2)GPI)(B2GPI)(Apolipoprotein H)(Apo-H)(Activated protein C-binding protein)(APC inhibitor)(Anticardiolipin cofactor) [Source: UniProt P02749]
2704.1.1	-4.3	5.06	2958.4139	-0.0036	4	38.61	revk	205	CPFPSRPDNGFVNYPAK	229	atfg	C [205] 57.0215, N [213] 0.9848, C [205]	ENSP00000205948	Beta-2-glycoprotein 1 Precursor (Beta-2-glycoprotein I)(Beta(2)GPI)(B2GPI)(Apolipoprotein H)(Apo-H)(Activated protein C-binding protein)(APC inhibitor)(Anticardiolipin cofactor) [Source: UniProt P02749]
2706.1.1	-4.8	4.59	1914.0041	-0.001	3	36.7	iagr	22	TCPKPDDLPFSTVVPLK	38	tfye	C [23] 57.0215	ENSP00000205948	Beta-2-glycoprotein 1 Precursor (Beta-2-glycoprotein I)(Beta(2)GPI)(B2GPI)(Apolipoprotein H)(Apo-H)(Activated protein C-binding protein)(APC inhibitor)(Anticardiolipin cofactor) [Source: UniProt P02749]
2711.1.1	-3.5	4.94	2958.4139	-0.006	4	38.61	revk	205	CPFPSRPDNGFVNYPAK	229	atfg	C [205] 57.0215, N [213] 0.9848, C [205]	ENSP00000205948	Beta-2-glycoprotein 1 Precursor (Beta-2-glycoprotein I)(Beta(2)GPI)(B2GPI)(Apolipoprotein H)(Apo-H)(Activated protein C-binding protein)(APC inhibitor)(Anticardiolipin cofactor) [Source: UniProt P02749]
2714.1.1	-4.7	4.44	1914.0041	0.001	3	36.7	iagr	22	TCPKPDDLPFSTVVPLK	38	tfye	C [23] 57.0215	ENSP00000205948	Beta-2-glycoprotein 1 Precursor (Beta-2-glycoprotein I)(Beta(2)GPI)(B2GPI)(Apolipoprotein H)(Apo-H)(Activated protein C-binding protein)(APC inhibitor)(Anticardiolipin cofactor) [Source: UniProt P02749]
2716.1.1	-5.9	4.07	2958.4139	-0.0025	3	38.61	revk	205	CPFPSRPDNGFVNYPAK	229	atfg	C [205] 57.0215, N [213] 0.9848, C [205]	ENSP00000205948	Beta-2-glycoprotein 1 Precursor (Beta-2-glycoprotein I)(Beta(2)GPI)(B2GPI)(Apolipoprotein H)(Apo-H)(Activated protein C-binding protein)(APC inhibitor)(Anticardiolipin cofactor) [Source: UniProt P02749]
2718.1.1	-6	4.81	2958.4139	-0.0014	4	38.61	revk	205	CPFPSRPDNGFVNYPAK	229	atfg	C [205] 57.0215, N [213] 0.9848, C [205]	ENSP00000205948	Beta-2-glycoprotein 1 Precursor (Beta-2-glycoprotein I)(Beta(2)GPI)(B2GPI)(Apolipoprotein H)(Apo-H)(Activated protein C-binding protein)(APC inhibitor)(Anticardiolipin cofactor) [Source: UniProt P02749]
2722.1.1	-3.7	3.89	2958.4139	0.0026	3	38.61	revk	205	CPFPSRPDNGFVNYPAK	229	atfg	C [205] 57.0215, N [213] 0.9848, C [205]	ENSP00000205948	Beta-2-glycoprotein 1 Precursor (Beta-2-glycoprotein I)(Beta(2)GPI)(B2GPI)(Apolipoprotein H)(Apo-H)(Activated protein C-binding protein)(APC inhibitor)(Anticardiolipin cofactor) [Source: UniProt P02749]
2725.1.1	-2.9	4.5	2169.137	-0.0047	3	36.23	vaia	20	GRTCPKPDDLPFSTVVP	38	tfye	C [23] 57.0215, G [20] 42.0106	ENSP00000205948	Beta-2-glycoprotein 1 Precursor (Beta-2-glycoprotein I)(Beta(2)GPI)(B2GPI)(Apolipoprotein H)(Apo-H)(Activated protein C-binding protein)(APC inhibitor)(Anticardiolipin cofactor) [Source: UniProt P02749]
2728.1.1	-3	4.25	1102.6255	0.0008	2	28.61	icpp	145	PSIPTFATLR	154	vykp		ENSP00000205948	Beta-2-glycoprotein 1 Precursor (Beta-2-glycoprotein I)(Beta(2)GPI)(B2GPI)(Apolipoprotein H)(Apo-H)(Activated protein C-binding protein)(APC inhibitor)(Anticardiolipin cofactor) [Source: UniProt P02749]
2735.1.1	-3.8	4.1	1102.6255	-0.0004	2	28.61	icpp	145	PSIPTFATLR	154	vykp		ENSP00000205948	Beta-2-glycoprotein 1 Precursor (Beta-2-glycoprotein I)(Beta(2)GPI)(B2GPI)(Apolipoprotein H)(Apo-H)(Activated protein C-binding protein)(APC inhibitor)(Anticardiolipin cofactor) [Source: UniProt P02749]
2746.1.1	-11.4	4.11	2068.8838	-0.0017	2	27.19	kekk	307	CSYTEDAQCIDGTIEVPI	324	cfke	C [307] 57.0215, C [315] 57.0215, C [307	ENSP00000205948	Beta-2-glycoprotein 1 Precursor (Beta-2-glycoprotein I)(Beta(2)GPI)(B2GPI)(Apolipoprotein H)(Apo-H)(Activated protein C-binding protein)(APC inhibitor)(Anticardiolipin cofactor) [Source: UniProt P02749]
2748.1.1	-2.2	4.22	2169.137	0.0033	4	36.23	vaia	20	GRTCPKPDDLPFSTVVP	38	tfye	C [23] 57.0215, G [20] 42.0106	ENSP00000205948	Beta-2-glycoprotein 1 Precursor (Beta-2-glycoprotein I)(Beta(2)GPI)(B2GPI)(Apolipoprotein H)(Apo-H)(Activated protein C-binding protein)(APC inhibitor)(Anticardiolipin cofactor) [Source: UniProt P02749]
2753.1.1	-13	5.09	2068.8838	0.0008	2	27.19	kekk	307	CSYTEDAQCIDGTIEVPI	324	cfke	C [307] 57.0215, C [315] 57.0215, C [307	ENSP00000205948	Beta-2-glycoprotein 1 Precursor (Beta-2-glycoprotein I)(Beta(2)GPI)(B2GPI)(Apolipoprotein H)(Apo-H)(Activated protein C-binding protein)(APC inhibitor)(Anticardiolipin cofactor) [Source: UniProt P02749]
2754.1.1	-3.7	3.62	2085.9103	0.0018	2	27.19	kekk	307	CSYTEDAQCIDGTIEVPI	324	cfke	C [307] 57.0215, C [315] 57.0215	ENSP00000205948	Beta-2-glycoprotein 1 Precursor (Beta-2-glycoprotein I)(Beta(2)GPI)(B2GPI)(Apolipoprotein H)(Apo-H)(Activated protein C-binding protein)(APC inhibitor)(Anticardiolipin cofactor) [Source: UniProt P02749]
2758.1.1	-3.3	3.81	1348.5945	-0.0002	2	28.33	vplk	39	TFYEPGEEITY	49	sckp		ENSP00000205948	Beta-2-glycoprotein 1 Precursor (Beta-2-glycoprotein I)(Beta(2)GPI)(B2GPI)(Apolipoprotein H)(Apo-H)(Activated protein C-binding protein)(APC inhibitor)(Anticardiolipin cofactor) [Source: UniProt P02749]
2760.1.1	-16.2	5.7	2068.8838	-0.0017	2	27.19	kekk	307	CSYTEDAQCIDGTIEVPI	324	cfke	C [307] 57.0215, C [315] 57.0215, C [307	ENSP00000205948	Beta-2-glycoprotein 1 Precursor (Beta-2-glycoprotein I)(Beta(2)GPI)(B2GPI)(Apolipoprotein H)(Apo-H)(Activated protein C-binding protein)(APC inhibitor)(Anticardiolipin cofactor) [Source: UniProt P02749]
2767.1.1	-4.4	3.63	2085.9103	0.0016	2	27.19	kekk	307	CSYTEDAQCIDGTIEVPI	324	cfke	C [307] 57.0215, C [315] 57.0215	ENSP00000205948	Beta-2-glycoprotein 1 Precursor (Beta-2-glycoprotein I)(Beta(2)GPI)(B2GPI)(Apolipoprotein H)(Apo-H)(Activated protein C-binding protein)(APC inhibitor)(Anticardiolipin cofactor) [Source: UniProt P02749]
2771.1.1	-13	4.48	2068.8838	0.0034	3	27.19	kekk	307	CSYTEDAQCIDGTIEVPI	324	cfke	C [307] 57.0215, C [315] 57.0215, C [307	ENSP00000205948	Beta-2-glycoprotein 1 Precursor (Beta-2-glycoprotein I)(Beta(2)GPI)(B2GPI)(Apolipoprotein H)(Apo-H)(Activated protein C-binding protein)(APC inhibitor)(Anticardiolipin cofactor) [Source: UniProt P02749]
2777.1.1	-10.6	4.34	2068.8838	0.0033	3	27.19	kekk	307	CSYTEDAQCIDGTIEVPI	324	cfke	C [307] 57.0215, C [315] 57.0215, C [307	ENSP00000205948	Beta-2-glycoprotein 1 Precursor (Beta-2-glycoprotein I)(Beta(2)GPI)(B2GPI)(Apolipoprotein H)(Apo-H)(Activated protein C-binding protein)(APC inhibitor)(Anticardiolipin cofactor) [Source: UniProt P02749]
2783.1.1	-10.1	4.29	2068.8838	0.004	3	27.19	kekk	307	CSYTEDAQCIDGTIEVPI	324	cfke	C [307] 57.0215, C [315] 57.0215, C [307	ENSP00000205948	Beta-2-glycoprotein 1 Precursor (Beta-2-glycoprotein I)(Beta(2)GPI)(B2GPI)(Apolipoprotein H)(Apo-H)(Activated protein C-binding protein)(APC inhibitor)(Anticardiolipin cofactor) [Source: UniProt P02749]
2790.1.1	-3.1	4.24	1208.5405	0.0015	2	25.72	evpk	325	CFKEHSSLAF	334	wktd	C [325] 57.0215, C [325] -17.0265	ENSP00000205948	Beta-2-glycoprotein 1 Precursor (Beta-2-glycoprotein I)(Beta(2)GPI)(B2GPI)(Apolipoprotein H)(Apo-H)(Activated protein C-binding protein)(APC inhibitor)(Anticardiolipin cofactor) [Source: UniProt P02749]
2792.1.1	-4.6	4.01	2112.1155	0.0016	3	37.03	aiag	21	RTCPKPDDLPFSTVVPLI	38	tfye	C [23] 57.0215, R [21] 42.0106	ENSP00000205948	Beta-2-glycoprotein 1 Precursor (Beta-2-glycoprotein I)(Beta(2)GPI)(B2GPI)(Apolipoprotein H)(Apo-H)(Activated protein C-binding protein)(APC inhibitor)(Anticardiolipin cofactor) [Source: UniProt P02749]
2797.1.1	-2.1	4.43	2732.3185	0.0068	4	38.86	revk	205	CPFPSRPDNGFVNYPAK	227	dkat	C [205] 57.0215, N [213] 0.9848	ENSP00000205948	Beta-2-glycoprotein 1 Precursor (Beta-2-glycoprotein I)(Beta(2)GPI)(B2GPI)(Apolipoprotein H)(Apo-H)(Activated protein C-binding protein)(APC inhibitor)(Anticardiolipin cofactor) [Source: UniProt P02749]
2800.1.1	-6.5	4.03	2112.1155	-0.0035	3	37.03	aiag	21	RTCPKPDDLPFSTVVPLI	38	tfye	C [23] 57.0215, R [21] 42.0106	ENSP00000205948	Beta-2-glycoprotein 1 Precursor (Beta-2-glycoprotein I)(Beta(2)GPI)(B2GPI)(Apolipoprotein H)(Apo-H)(Activated protein C-binding protein)(APC inhibitor)(Anticardiolipin cofactor) [Source: UniProt P02749]
2805.1.1	-7	4.44	2714.3072	0.0034	3	38.86	revk	205	CPFPSRPDNGFVNYPAK	227	dkat	C [205] 57.0215, C [205] -17.0265	ENSP00000205948	Beta-2-glycoprotein 1 Precursor (Beta-2-glycoprotein I)(Beta(2)GPI)(B2GPI)(Apolipoprotein H)(Apo-H)(Activated protein C-binding protein)(APC inhibitor)(Anticardiolipin cofactor) [Source: UniProt P02749]
2809.1.1	-5.6	3.79	2112.1155	-0.0009	3	37.03	aiag	21	RTCPKPDDLPFSTVVPLI	38	tfye	C [23] 57.0215, R [21] 42.0106	ENSP00000205948	Beta-2-glycoprotein 1 Precursor (Beta-2-glycoprotein I)(Beta(2)GPI)(B2GPI)(Apolipoprotein H)(Apo-H)(Activated protein C-binding protein)(APC inhibitor)(Anticardiolipin cofactor) [Source: UniProt P02749]
2812.1.1	-6.9	5.43	2714.3072	0.0018	3	38.86	revk	205	CPFPSRPDNGFVNYPAK	227	dkat	C [205] 57.0215, C [205] -17.0265	ENSP00000205948	Beta-2-glycoprotein 1 Precursor (Beta-2-glycoprotein I)(Beta(2)GPI)(B2GPI)(Apolipoprotein H)(Apo-H)(Activated protein C-binding protein)(APC inhibitor)(Anticardiolipin cofactor) [Source: UniProt P02749]
2814.1.1	-4.3	4.73	1914.0041	0.0008	3	36.7	iagr	22	TCPKPDDLPFSTVVPLK	38	tfye	C [23] 57.0215	ENSP00000205948	Beta-2-glycoprotein 1 Precursor (Beta-2-glycoprotein I)(Beta(2)GPI)(B2GPI)(Apolipoprotein H)(Apo-H)(Activated protein C-binding protein)(APC inhibitor)(Anticardiolipin cofactor) [Source: UniProt P02749]
2816.1.1	-8.1	4.28	1199.6783	-0.0015	2	28.12	iicp	144	PPSIPTFATLR	154	vykp		ENSP00000205948	Beta-2-glycoprotein 1 Precursor (Beta-2-glycoprotein I)(Beta(2)GPI)(B2GPI)(Apolipoprotein H)(Apo-H)(Activated protein C-binding protein)(APC inhibitor)(Anticardiolipin cofactor) [Source: UniProt P02749]
2819.1.1	-8.6	5.95	2714.3072	0.0003	3	38.86	revk	205	CPFPSRPDNGFVNYPAK	227	dkat	C [205] 57.0215, C [205] -17.0265	ENSP00000205948	Beta-2-glycoprotein 1 Precursor (Beta-2-glycoprotein I)(Beta(2)GPI)(B2GPI)(Apolipoprotein H)(Apo-H)(Activated protein C-binding protein)(APC inhibitor)(Anticardiolipin cofactor) [Source: UniProt P02749]
2821.1.1	-6.1	4.95	1914.0041	0.0019	3	36.7	iagr	22	TCPKPDDLPFSTVVPLK	38	tfye	C [23] 57.0215	ENSP00000205948	Beta-2-glycoprotein 1 Precursor (Beta-2-glycoprotein I)(Beta(2)GPI)(B2GPI)(Apolipoprotein H)(Apo-H)(Activated protein C-binding protein)(APC inhibitor)(Anticardiolipin cofactor) [Source: UniProt P02749]
2823.1.1	-6	4.35	1199.6783	-0.0006	2	28.12	iicp	144	PPSIPTFATLR	154	vykp		ENSP00000205948	Beta-2-glycoprotein 1 Precursor (Beta-2-glycoprotein I)(Beta(2)GPI)(B2GPI)(Apolipoprotein H)(Apo-H)(Activated protein C-binding protein)(APC inhibitor)(Anticardiolipin cofactor) [Source: UniProt P02749]
2826.1.1	-4.9	5.09	1914.0041	0.0019	3	36.7	iagr	22	TCPKPDDLPFSTVVPLK	38	tfye	C [23] 57.0215	ENSP00000205948	Beta-2-glycoprotein 1 Precursor (Beta-2-glycoprotein I)(Beta(2)GPI)(B2GPI)(Apolipoprotein H)(Apo-H)(Activated protein C-binding protein)(APC inhibitor)(Anticardiolipin cofactor) [Source: UniProt P02749]
2829.1.1	-7.8	4.32	1199.6783	0.0005	2	28.12	iicp	144	PPSIPTFATLR	154	vykp		ENSP00000205948	Beta-2-glycoprotein 1 Precursor (Beta-2-glycoprotein I)(Beta(2)GPI)(B2GPI)(Apolipoprotein H)(Apo-H)(Activated protein C-binding protein)(APC inhibitor)(Anticardiolipin cofactor) [Source: UniProt P02749]
2835.1.1	-4.6	4.6	1914.0041	0.0034	2	36.7	iagr	22	TCPKPDDLPFSTVVPLK	38	tfye	C [23] 57.0215	ENSP00000205948	Beta-2-glycoprotein 1 Precursor (Beta-2-glycoprotein I)(Beta(2)GPI)(B2GPI)(Apolipoprotein H)(Apo-H)(Activated protein C-binding protein)(APC inhibitor)(Anticardiolipin cofactor) [Source: UniProt P02749]
2840.1.1	-9.4	5.09	1914.0041	0.0017	2	36.7	iagr	22	TCPKPDDLPFSTVVPLK	38	tfye	C [23] 57.0215	ENSP00000205948	Beta-2-glycoprotein 1 Precursor (Beta-2-glycoprotein I)(Beta(2)GPI)(B2GPI)(Apolipoprotein H)(Apo-H)(Activated protein C-binding protein)(APC inhibitor)(Anticardiolipin cofactor) [Source: UniProt P02749]
2847.1.1	-5.4	5.53	1914.0041	0.0019	2	36.7	iagr	22	TCPKPDDLPFSTVVPLK	38	tfye	C [23] 57.0215	ENSP00000205948	Beta-2-glycoprotein 1 Precursor (Beta-2-glycoprotein I)(Beta(2)GPI)(B2GPI)(Apolipoprotein H)(Apo-H)(Activated protein C-binding protein)(APC inhibitor)(Anticardiolipin cofactor) [Source: UniProt P02749]
2866.1.1	-9.8	4.76	1522.7147	0	2	33.42	evpk	325	CFKEHSSLAFWK	336	tdas	C [325] 57.0215, C [325] -17.0265	ENSP00000205948	Beta-2-glycoprotein 1 Precursor (Beta-2-glycoprotein I)(Beta(2)GPI)(B2GPI)(Apolipoprotein H)(Apo-H)(Activated protein C-binding protein)(APC inhibitor)(Anticardiolipin cofactor) [Source: UniProt P02749]
2868.1.1	-2.5	5.81	1522.7147	0.0003	3	33.42	evpk	325	CFKEHSSLAFWK	336	tdas	C [325] 57.0215, C [325] -17.0265	ENSP00000205948	Beta-2-glycoprotein 1 Precursor (Beta-2-glycoprotein I)(Beta(2)GPI)(B2GPI)(Apolipoprotein H)(Apo-H)(Activated protein C-binding protein)(APC inhibitor)(Anticardiolipin cofactor) [Source: UniProt P02749]
2869.1.1	-7.1	4.96	1522.7147	-0.0006	2	33.42	evpk	325	CFKEHSSLAFWK	336	tdas	C [325] 57.0215, C [325] -17.0265	ENSP00000205948	Beta-2-glycoprotein 1 Precursor (Beta-2-glycoprotein I)(Beta(2)GPI)(B2GPI)(Apolipoprotein H)(Apo-H)(Activated protein C-binding protein)(APC inhibitor)(Anticardiolipin cofactor) [Source: UniProt P02749]
2870.1.1	-2.2	4.61	1100.6714	-0.0014	2	32.27	kpdd	29	LPFSTVVPLK	38	tfye		ENSP00000205948	Beta-2-glycoprotein 1 Precursor (Beta-2-glycoprotein I)(Beta(2)GPI)(B2GPI)(Apolipoprotein H)(Apo-H)(Activated protein C-binding protein)(APC inhibitor)(Anticardiolipin cofactor) [Source: UniProt P02749]
2875.1.1	-2.5	5.98	1522.7147	0.0013	3	33.42	evpk	325	CFKEHSSLAFWK	336	tdas	C [325] 57.0215, C [325] -17.0265	ENSP00000205948	Beta-2-glycoprotein 1 Precursor (Beta-2-glycoprotein I)(Beta(2)GPI)(B2GPI)(Apolipoprotein H)(Apo-H)(Activated protein C-binding protein)(APC inhibitor)(Anticardiolipin cofactor) [Source: UniProt P02749]
2876.1.1	-6.8	5.05	1522.7147	0.0005	2	33.42	evpk	325	CFKEHSSLAFWK	336	tdas	C [325] 57.0215, C [325] -17.0265	ENSP00000205948	Beta-2-glycoprotein 1 Precursor (Beta-2-glycoprotein I)(Beta(2)GPI)(B2GPI)(Apolipoprotein H)(Apo-H)(Activated protein C-binding protein)(APC inhibitor)(Anticardiolipin cofactor) [Source: UniProt P02749]
2878.1.1	-2.4	4.5	1100.6714	0.0001	2	32.27	kpdd	29	LPFSTVVPLK	38	tfye		ENSP00000205948	Beta-2-glycoprotein 1 Precursor (Beta-2-glycoprotein I)(Beta(2)GPI)(B2GPI)(Apolipoprotein H)(Apo-H)(Activated protein C-binding protein)(APC inhibitor)(Anticardiolipin cofactor) [Source: UniProt P02749]
2881.1.1	-2.4	6.13	1522.7147	0.0017	3	33.42	evpk	325	CFKEHSSLAFWK	336	tdas	C [325] 57.0215, C [325] -17.0265	ENSP00000205948	Beta-2-glycoprotein 1 Precursor (Beta-2-glycoprotein I)(Beta(2)GPI)(B2GPI)(Apolipoprotein H)(Apo-H)(Activated protein C-binding protein)(APC inhibitor)(Anticardiolipin cofactor) [Source: UniProt P02749]
2883.1.1	-2.7	3.71	1258.6889	-0.0016	2	29.92	gank	95	EKLEATINELV	105	]		ENSP00000363641	Thioredoxin (Trx)(ATL-derived factor)(ADF)(Surface-associated sulphydryl protein)(SASP) [Source: UniProt P10599]
2889.1.1	-3.6	3.82	1258.6889	0.0002	2	29.92	gank	95	EKLEATINELV	105	]		ENSP00000363641	Thioredoxin (Trx)(ATL-derived factor)(ADF)(Surface-associated sulphydryl protein)(SASP) [Source: UniProt P10599]
2907.1.1	-5.7	5.91	2715.292	0.0008	3	38.86	revk	205	CPFPSRPDNGFVNYPAK	227	dkat	C [205] 57.0215, N [213] 0.9848, C [205]	ENSP00000205948	Beta-2-glycoprotein 1 Precursor (Beta-2-glycoprotein I)(Beta(2)GPI)(B2GPI)(Apolipoprotein H)(Apo-H)(Activated protein C-binding protein)(APC inhibitor)(Anticardiolipin cofactor) [Source: UniProt P02749]
2910.1.1	-5.5	5.87	2715.292	0.0023	3	38.86	revk	205	CPFPSRPDNGFVNYPAK	227	dkat	C [205] 57.0215, N [213] 0.9848, C [205]	ENSP00000205948	Beta-2-glycoprotein 1 Precursor (Beta-2-glycoprotein I)(Beta(2)GPI)(B2GPI)(Apolipoprotein H)(Apo-H)(Activated protein C-binding protein)(APC inhibitor)(Anticardiolipin cofactor) [Source: UniProt P02749]
2911.1.1	-4.4	5.66	1914.0041	0.0003	3	36.7	iagr	22	TCPKPDDLPFSTVVPLK	38	tfye	C [23] 57.0215	ENSP00000205948	Beta-2-glycoprotein 1 Precursor (Beta-2-glycoprotein I)(Beta(2)GPI)(B2GPI)(Apolipoprotein H)(Apo-H)(Activated protein C-binding protein)(APC inhibitor)(Anticardiolipin cofactor) [Source: UniProt P02749]
2912.1.1	-2.2	4.02	1048.586	-0.0027	2	32	ggmr	63	KFICPLTGL	71	wpin	C [66] 57.0215	ENSP00000205948	Beta-2-glycoprotein 1 Precursor (Beta-2-glycoprotein I)(Beta(2)GPI)(B2GPI)(Apolipoprotein H)(Apo-H)(Activated protein C-binding protein)(APC inhibitor)(Anticardiolipin cofactor) [Source: UniProt P02749]
2916.1.1	-9.9	5.83	2715.292	0.0017	3	38.86	revk	205	CPFPSRPDNGFVNYPAK	227	dkat	C [205] 57.0215, N [213] 0.9848, C [205]	ENSP00000205948	Beta-2-glycoprotein 1 Precursor (Beta-2-glycoprotein I)(Beta(2)GPI)(B2GPI)(Apolipoprotein H)(Apo-H)(Activated protein C-binding protein)(APC inhibitor)(Anticardiolipin cofactor) [Source: UniProt P02749]
2917.1.1	-4.4	5.5	1914.0041	-0.0001	3	36.7	iagr	22	TCPKPDDLPFSTVVPLK	38	tfye	C [23] 57.0215	ENSP00000205948	Beta-2-glycoprotein 1 Precursor (Beta-2-glycoprotein I)(Beta(2)GPI)(B2GPI)(Apolipoprotein H)(Apo-H)(Activated protein C-binding protein)(APC inhibitor)(Anticardiolipin cofactor) [Source: UniProt P02749]
2921.1.1	-3.8	5.48	1914.0041	0.0006	3	36.7	iagr	22	TCPKPDDLPFSTVVPLK	38	tfye	C [23] 57.0215	ENSP00000205948	Beta-2-glycoprotein 1 Precursor (Beta-2-glycoprotein I)(Beta(2)GPI)(B2GPI)(Apolipoprotein H)(Apo-H)(Activated protein C-binding protein)(APC inhibitor)(Anticardiolipin cofactor) [Source: UniProt P02749]
2933.1.1	-8.2	3.91	2068.8838	-0.0026	2	27.19	kekk	307	CSYTEDAQCIDGTIEVPI	324	cfke	C [307] 57.0215, C [315] 57.0215, C [307	ENSP00000205948	Beta-2-glycoprotein 1 Precursor (Beta-2-glycoprotein I)(Beta(2)GPI)(B2GPI)(Apolipoprotein H)(Apo-H)(Activated protein C-binding protein)(APC inhibitor)(Anticardiolipin cofactor) [Source: UniProt P02749]
2936.1.1	-1.2	4.63	2114.0976	-0.0038	3	38.4	qkir	344	SLLDQASIPSDTISETPIA	363	eelg		ENSP00000262085:reversed	
2938.1.1	-3.6	4.3	1914.0041	-0.0004	2	36.7	iagr	22	TCPKPDDLPFSTVVPLK	38	tfye	C [23] 57.0215	ENSP00000205948	Beta-2-glycoprotein 1 Precursor (Beta-2-glycoprotein I)(Beta(2)GPI)(B2GPI)(Apolipoprotein H)(Apo-H)(Activated protein C-binding protein)(APC inhibitor)(Anticardiolipin cofactor) [Source: UniProt P02749]
2940.1.1	-11.1	3.9	2068.8838	0.0003	2	27.19	kekk	307	CSYTEDAQCIDGTIEVPI	324	cfke	C [307] 57.0215, C [315] 57.0215, C [307	ENSP00000205948	Beta-2-glycoprotein 1 Precursor (Beta-2-glycoprotein I)(Beta(2)GPI)(B2GPI)(Apolipoprotein H)(Apo-H)(Activated protein C-binding protein)(APC inhibitor)(Anticardiolipin cofactor) [Source: UniProt P02749]
2946.1.1	-7.2	3.78	2068.8838	0.0003	2	27.19	kekk	307	CSYTEDAQCIDGTIEVPI	324	cfke	C [307] 57.0215, C [315] 57.0215, C [307	ENSP00000205948	Beta-2-glycoprotein 1 Precursor (Beta-2-glycoprotein I)(Beta(2)GPI)(B2GPI)(Apolipoprotein H)(Apo-H)(Activated protein C-binding protein)(APC inhibitor)(Anticardiolipin cofactor) [Source: UniProt P02749]
2948.1.1	-1.6	4.36	2114.113	-0.015	3	38.57	tfak	863	GNSETKLHFLSLSDLPEV	881	vkee		ENSP00000362748:reversed	
2960.1.1	-2.1	4.6	1522.7147	0	3	33.42	evpk	325	CFKEHSSLAFWK	336	tdas	C [325] 57.0215, C [325] -17.0265	ENSP00000205948	Beta-2-glycoprotein 1 Precursor (Beta-2-glycoprotein I)(Beta(2)GPI)(B2GPI)(Apolipoprotein H)(Apo-H)(Activated protein C-binding protein)(APC inhibitor)(Anticardiolipin cofactor) [Source: UniProt P02749]
2962.1.1	-7.2	3.9	1502.7784	0.0081	2	32.15	ctpr	83	VCPFAGILENGAVR	96	yttf	C [84] 57.0215	ENSP00000205948	Beta-2-glycoprotein 1 Precursor (Beta-2-glycoprotein I)(Beta(2)GPI)(B2GPI)(Apolipoprotein H)(Apo-H)(Activated protein C-binding protein)(APC inhibitor)(Anticardiolipin cofactor) [Source: UniProt P02749]
2971.1.1	-2.3	3.95	1502.7784	0.0031	2	32.15	ctpr	83	VCPFAGILENGAVR	96	yttf	C [84] 57.0215	ENSP00000205948	Beta-2-glycoprotein 1 Precursor (Beta-2-glycoprotein I)(Beta(2)GPI)(B2GPI)(Apolipoprotein H)(Apo-H)(Activated protein C-binding protein)(APC inhibitor)(Anticardiolipin cofactor) [Source: UniProt P02749]
2975.1.1	-2	4.55	1522.7147	0.0019	3	33.42	evpk	325	CFKEHSSLAFWK	336	tdas	C [325] 57.0215, C [325] -17.0265	ENSP00000205948	Beta-2-glycoprotein 1 Precursor (Beta-2-glycoprotein I)(Beta(2)GPI)(B2GPI)(Apolipoprotein H)(Apo-H)(Activated protein C-binding protein)(APC inhibitor)(Anticardiolipin cofactor) [Source: UniProt P02749]
2979.1.1	-2.5	3.87	1914.0041	0.0042	2	36.7	iagr	22	TCPKPDDLPFSTVVPLK	38	tfye	C [23] 57.0215	ENSP00000205948	Beta-2-glycoprotein 1 Precursor (Beta-2-glycoprotein I)(Beta(2)GPI)(B2GPI)(Apolipoprotein H)(Apo-H)(Activated protein C-binding protein)(APC inhibitor)(Anticardiolipin cofactor) [Source: UniProt P02749]
2985.1.1	-2.3	3.8	1956.0145	0.0024	3	36.7	iagr	22	TCPKPDDLPFSTVVPLK	38	tfye	C [23] 57.0215, T [22] 42.0106	ENSP00000205948	Beta-2-glycoprotein 1 Precursor (Beta-2-glycoprotein I)(Beta(2)GPI)(B2GPI)(Apolipoprotein H)(Apo-H)(Activated protein C-binding protein)(APC inhibitor)(Anticardiolipin cofactor) [Source: UniProt P02749]
2987.1.1	-8	4.4	1503.7632	-0.0012	2	32.15	ctpr	83	VCPFAGILENGAVR	96	yttf	C [84] 57.0215, N [92] 0.9848	ENSP00000205948	Beta-2-glycoprotein 1 Precursor (Beta-2-glycoprotein I)(Beta(2)GPI)(B2GPI)(Apolipoprotein H)(Apo-H)(Activated protein C-binding protein)(APC inhibitor)(Anticardiolipin cofactor) [Source: UniProt P02749]
2995.1.1	-11.4	4.72	2715.292	0.0092	3	38.86	revk	205	CPFPSRPDNGFVNYPAK	227	dkat	C [205] 57.0215, N [213] 0.9848, C [205]	ENSP00000205948	Beta-2-glycoprotein 1 Precursor (Beta-2-glycoprotein I)(Beta(2)GPI)(B2GPI)(Apolipoprotein H)(Apo-H)(Activated protein C-binding protein)(APC inhibitor)(Anticardiolipin cofactor) [Source: UniProt P02749]

3000.1.1	-5.1	4.81	1914.0041	-0.0008	3	36.7	iagr	22	TCPKPDDLPFSTVVPLK	38	tfye	C [23] 57.0215	ENSP00000205948	Beta-2-glycoprotein 1 Precursor (Beta-2-glycoprotein I)(Beta(2)GPI)(B2GPI)(Apolipoprotein H)(Apo-H)(Activated protein C-binding protein)(APC inhibitor)(Anticardiolipin cofactor) [Source: UniProt P02749]
3001.1.1	-2.9	4.86	1956.0145	0.0029	3	36.7	iagr	22	TCPKPDDLPFSTVVPLK	38	tfye	C [23] 57.0215, T [22] 42.0106	ENSP00000205948	Beta-2-glycoprotein 1 Precursor (Beta-2-glycoprotein I)(Beta(2)GPI)(B2GPI)(Apolipoprotein H)(Apo-H)(Activated protein C-binding protein)(APC inhibitor)(Anticardiolipin cofactor) [Source: UniProt P02749]
3003.1.1	-3.9	4.6	1503.7632	0.0021	3	32.15	ctpr	83	VCPFAGILENGAVR	96	yttf	C [84] 57.0215, N [92] 0.9848	ENSP00000205948	Beta-2-glycoprotein 1 Precursor (Beta-2-glycoprotein I)(Beta(2)GPI)(B2GPI)(Apolipoprotein H)(Apo-H)(Activated protein C-binding protein)(APC inhibitor)(Anticardiolipin cofactor) [Source: UniProt P02749]
3007.1.1	-3.3	5.25	1956.0145	0.0009	3	36.7	iagr	22	TCPKPDDLPFSTVVPLK	38	tfye	C [23] 57.0215, T [22] 42.0106	ENSP00000205948	Beta-2-glycoprotein 1 Precursor (Beta-2-glycoprotein I)(Beta(2)GPI)(B2GPI)(Apolipoprotein H)(Apo-H)(Activated protein C-binding protein)(APC inhibitor)(Anticardiolipin cofactor) [Source: UniProt P02749]
3008.1.1	-5.5	4.63	1914.0041	0.0008	3	36.7	iagr	22	TCPKPDDLPFSTVVPLK	38	tfye	C [23] 57.0215	ENSP00000205948	Beta-2-glycoprotein 1 Precursor (Beta-2-glycoprotein I)(Beta(2)GPI)(B2GPI)(Apolipoprotein H)(Apo-H)(Activated protein C-binding protein)(APC inhibitor)(Anticardiolipin cofactor) [Source: UniProt P02749]
3009.1.1	-4.9	4.89	1503.7632	0.001	3	32.15	ctpr	83	VCPFAGILENGAVR	96	yttf	C [84] 57.0215, N [92] 0.9848	ENSP00000205948	Beta-2-glycoprotein 1 Precursor (Beta-2-glycoprotein I)(Beta(2)GPI)(B2GPI)(Apolipoprotein H)(Apo-H)(Activated protein C-binding protein)(APC inhibitor)(Anticardiolipin cofactor) [Source: UniProt P02749]
3010.1.1	-2.6	4.49	1956.0145	0.0024	2	36.7	iagr	22	TCPKPDDLPFSTVVPLK	38	tfye	C [23] 57.0215, T [22] 42.0106	ENSP00000205948	Beta-2-glycoprotein 1 Precursor (Beta-2-glycoprotein I)(Beta(2)GPI)(B2GPI)(Apolipoprotein H)(Apo-H)(Activated protein C-binding protein)(APC inhibitor)(Anticardiolipin cofactor) [Source: UniProt P02749]
3015.1.1	-4.3	5.33	1503.7632	0.0007	3	32.15	ctpr	83	VCPFAGILENGAVR	96	yttf	C [84] 57.0215, N [92] 0.9848	ENSP00000205948	Beta-2-glycoprotein 1 Precursor (Beta-2-glycoprotein I)(Beta(2)GPI)(B2GPI)(Apolipoprotein H)(Apo-H)(Activated protein C-binding protein)(APC inhibitor)(Anticardiolipin cofactor) [Source: UniProt P02749]
3017.1.1	-5.2	4.77	1914.0041	0.0015	3	36.7	iagr	22	TCPKPDDLPFSTVVPLK	38	tfye	C [23] 57.0215	ENSP00000205948	Beta-2-glycoprotein 1 Precursor (Beta-2-glycoprotein I)(Beta(2)GPI)(B2GPI)(Apolipoprotein H)(Apo-H)(Activated protein C-binding protein)(APC inhibitor)(Anticardiolipin cofactor) [Source: UniProt P02749]
3021.1.1	-4.4	4.83	1956.0145	0.0024	2	36.7	iagr	22	TCPKPDDLPFSTVVPLK	38	tfye	C [23] 57.0215, T [22] 42.0106	ENSP00000205948	Beta-2-glycoprotein 1 Precursor (Beta-2-glycoprotein I)(Beta(2)GPI)(B2GPI)(Apolipoprotein H)(Apo-H)(Activated protein C-binding protein)(APC inhibitor)(Anticardiolipin cofactor) [Source: UniProt P02749]
3032.1.1	-1.2	4.41	1525.7428	0.0058	2	26.08	selr	1279	RTVYLCTDDNLR	1290	gadm	C [1284] 57.0215	ENSP00000359630	
3036.1.1	-6.9	4.39	2715.292	0.01	3	38.86	revk	205	CPFPSRPDNGFVNYPAK	227	dkat	C [205] 57.0215, N [213] 0.9848, C [205]	ENSP00000205948	Beta-2-glycoprotein 1 Precursor (Beta-2-glycoprotein I)(Beta(2)GPI)(B2GPI)(Apolipoprotein H)(Apo-H)(Activated protein C-binding protein)(APC inhibitor)(Anticardiolipin cofactor) [Source: UniProt P02749]
3044.1.1	-7.4	4.34	2715.292	0.014	3	38.86	revk	205	CPFPSRPDNGFVNYPAK	227	dkat	C [205] 57.0215, N [213] 0.9848, C [205]	ENSP00000205948	Beta-2-glycoprotein 1 Precursor (Beta-2-glycoprotein I)(Beta(2)GPI)(B2GPI)(Apolipoprotein H)(Apo-H)(Activated protein C-binding protein)(APC inhibitor)(Anticardiolipin cofactor) [Source: UniProt P02749]
3049.1.1	-8.3	4.15	1843.7843	0.0034	2	34.21	gavr	97	YTTFEYPNTISFSCN	111	tgfy	C [110] 57.0215	ENSP00000205948	Beta-2-glycoprotein 1 Precursor (Beta-2-glycoprotein I)(Beta(2)GPI)(B2GPI)(Apolipoprotein H)(Apo-H)(Activated protein C-binding protein)(APC inhibitor)(Anticardiolipin cofactor) [Source: UniProt P02749]
3053.1.1	-10.6	6.84	1502.7784	-0.0005	2	32.15	ctpr	83	VCPFAGILENGAVR	96	yttf	C [84] 57.0215	ENSP00000205948	Beta-2-glycoprotein 1 Precursor (Beta-2-glycoprotein I)(Beta(2)GPI)(B2GPI)(Apolipoprotein H)(Apo-H)(Activated protein C-binding protein)(APC inhibitor)(Anticardiolipin cofactor) [Source: UniProt P02749]
3057.1.1	-10.3	6.71	1502.7784	-0.001	2	32.15	ctpr	83	VCPFAGILENGAVR	96	yttf	C [84] 57.0215	ENSP00000205948	Beta-2-glycoprotein 1 Precursor (Beta-2-glycoprotein I)(Beta(2)GPI)(B2GPI)(Apolipoprotein H)(Apo-H)(Activated protein C-binding protein)(APC inhibitor)(Anticardiolipin cofactor) [Source: UniProt P02749]
3061.1.1	-9.4	6.74	1502.7784	0.0003	2	32.15	ctpr	83	VCPFAGILENGAVR	96	yttf	C [84] 57.0215	ENSP00000205948	Beta-2-glycoprotein 1 Precursor (Beta-2-glycoprotein I)(Beta(2)GPI)(B2GPI)(Apolipoprotein H)(Apo-H)(Activated protein C-binding protein)(APC inhibitor)(Anticardiolipin cofactor) [Source: UniProt P02749]
3072.1.1	-2.4	3.85	2383.0911	0.0022	3	33.69	vplk	39	TFYEPGEEITYSCKPGYV	58	ggmr	C [51] 57.0215	ENSP00000205948	Beta-2-glycoprotein 1 Precursor (Beta-2-glycoprotein I)(Beta(2)GPI)(B2GPI)(Apolipoprotein H)(Apo-H)(Activated protein C-binding protein)(APC inhibitor)(Anticardiolipin cofactor) [Source: UniProt P02749]
3075.1.1	-8.5	4.29	2715.292	0.0057	3	38.86	revk	205	CPFPSRPDNGFVNYPAK	227	dkat	C [205] 57.0215, N [213] 0.9848, C [205]	ENSP00000205948	Beta-2-glycoprotein 1 Precursor (Beta-2-glycoprotein I)(Beta(2)GPI)(B2GPI)(Apolipoprotein H)(Apo-H)(Activated protein C-binding protein)(APC inhibitor)(Anticardiolipin cofactor) [Source: UniProt P02749]
3076.1.1	-7.7	4.45	2423.1486	0.0033	2	38.48	revk	205	CPFPSRPDNGFVNYPAK	225	ykdk	C [205] 57.0215, C [205] -17.0265	ENSP00000205948	Beta-2-glycoprotein 1 Precursor (Beta-2-glycoprotein I)(Beta(2)GPI)(B2GPI)(Apolipoprotein H)(Apo-H)(Activated protein C-binding protein)(APC inhibitor)(Anticardiolipin cofactor) [Source: UniProt P02749]
3078.1.1	-7.1	5.69	1502.7784	0.0024	3	32.15	ctpr	83	VCPFAGILENGAVR	96	yttf	C [84] 57.0215	ENSP00000205948	Beta-2-glycoprotein 1 Precursor (Beta-2-glycoprotein I)(Beta(2)GPI)(B2GPI)(Apolipoprotein H)(Apo-H)(Activated protein C-binding protein)(APC inhibitor)(Anticardiolipin cofactor) [Source: UniProt P02749]
3079.1.1	-7.6	4.26	2715.292	0.014	3	38.86	revk	205	CPFPSRPDNGFVNYPAK	227	dkat	C [205] 57.0215, N [213] 0.9848, C [205]	ENSP00000205948	Beta-2-glycoprotein 1 Precursor (Beta-2-glycoprotein I)(Beta(2)GPI)(B2GPI)(Apolipoprotein H)(Apo-H)(Activated protein C-binding protein)(APC inhibitor)(Anticardiolipin cofactor) [Source: UniProt P02749]
3080.1.1	-9.4	4.11	2423.1486	0.0071	3	38.48	revk	205	CPFPSRPDNGFVNYPAK	225	ykdk	C [205] 57.0215, C [205] -17.0265	ENSP00000205948	Beta-2-glycoprotein 1 Precursor (Beta-2-glycoprotein I)(Beta(2)GPI)(B2GPI)(Apolipoprotein H)(Apo-H)(Activated protein C-binding protein)(APC inhibitor)(Anticardiolipin cofactor) [Source: UniProt P02749]
3081.1.1	-9.7	4.48	2423.1486	0.0079	2	38.48	revk	205	CPFPSRPDNGFVNYPAK	225	ykdk	C [205] 57.0215, C [205] -17.0265	ENSP00000205948	Beta-2-glycoprotein 1 Precursor (Beta-2-glycoprotein I)(Beta(2)GPI)(B2GPI)(Apolipoprotein H)(Apo-H)(Activated protein C-binding protein)(APC inhibitor)(Anticardiolipin cofactor) [Source: UniProt P02749]
3082.1.1	-5	4.42	1914.0041	-0.0063	3	36.7	iagr	22	TCPKPDDLPFSTVVPLK	38	tfye	C [23] 57.0215	ENSP00000205948	Beta-2-glycoprotein 1 Precursor (Beta-2-glycoprotein I)(Beta(2)GPI)(B2GPI)(Apolipoprotein H)(Apo-H)(Activated protein C-binding protein)(APC inhibitor)(Anticardiolipin cofactor) [Source: UniProt P02749]
3085.1.1	-5.4	5.65	1502.7784	0.0017	3	32.15	ctpr	83	VCPFAGILENGAVR	96	yttf	C [84] 57.0215	ENSP00000205948	Beta-2-glycoprotein 1 Precursor (Beta-2-glycoprotein I)(Beta(2)GPI)(B2GPI)(Apolipoprotein H)(Apo-H)(Activated protein C-binding protein)(APC inhibitor)(Anticardiolipin cofactor) [Source: UniProt P02749]
3086.1.1	-8	4.27	2715.292	0.012	3	38.86	revk	205	CPFPSRPDNGFVNYPAK	227	dkat	C [205] 57.0215, N [213] 0.9848, C [205]	ENSP00000205948	Beta-2-glycoprotein 1 Precursor (Beta-2-glycoprotein I)(Beta(2)GPI)(B2GPI)(Apolipoprotein H)(Apo-H)(Activated protein C-binding protein)(APC inhibitor)(Anticardiolipin cofactor) [Source: UniProt P02749]
3087.1.1	-8	4.4	2423.1486	-0.0008	3	38.48	revk	205	CPFPSRPDNGFVNYPAK	225	ykdk	C [205] 57.0215, C [205] -17.0265	ENSP00000205948	Beta-2-glycoprotein 1 Precursor (Beta-2-glycoprotein I)(Beta(2)GPI)(B2GPI)(Apolipoprotein H)(Apo-H)(Activated protein C-binding protein)(APC inhibitor)(Anticardiolipin cofactor) [Source: UniProt P02749]
3088.1.1	-5.4	4.34	2423.1486	0.0011	2	38.48	revk	205	CPFPSRPDNGFVNYPAK	225	ykdk	C [205] 57.0215, C [205] -17.0265	ENSP00000205948	Beta-2-glycoprotein 1 Precursor (Beta-2-glycoprotein I)(Beta(2)GPI)(B2GPI)(Apolipoprotein H)(Apo-H)(Activated protein C-binding protein)(APC inhibitor)(Anticardiolipin cofactor) [Source: UniProt P02749]
3092.1.1	-5.9	5.4	1502.7784	0.0021	3	32.15	ctpr	83	VCPFAGILENGAVR	96	yttf	C [84] 57.0215	ENSP00000205948	Beta-2-glycoprotein 1 Precursor (Beta-2-glycoprotein I)(Beta(2)GPI)(B2GPI)(Apolipoprotein H)(Apo-H)(Activated protein C-binding protein)(APC inhibitor)(Anticardiolipin cofactor) [Source: UniProt P02749]
3093.1.1	-7	4.3	2423.1486	0.0009	3	38.48	revk	205	CPFPSRPDNGFVNYPAK	225	ykdk	C [205] 57.0215, C [205] -17.0265	ENSP00000205948	Beta-2-glycoprotein 1 Precursor (Beta-2-glycoprotein I)(Beta(2)GPI)(B2GPI)(Apolipoprotein H)(Apo-H)(Activated protein C-binding protein)(APC inhibitor)(Anticardiolipin cofactor) [Source: UniProt P02749]
3099.1.1	-1.1	4.28	1560.7985	-0.0056	2	28.39	enir	34	LECGDQLMRVAIR	46	aink	C [36] 57.0215	ENSP00000250263:reversed	
3100.1.1	-3.8	4.43	1914.0041	0.001	3	36.7	iagr	22	TCPKPDDLPFSTVVPLK	38	tfye	C [23] 57.0215	ENSP00000205948	Beta-2-glycoprotein 1 Precursor (Beta-2-glycoprotein I)(Beta(2)GPI)(B2GPI)(Apolipoprotein H)(Apo-H)(Activated protein C-binding protein)(APC inhibitor)(Anticardiolipin cofactor) [Source: UniProt P02749]
3102.1.1	-2.3	3.92	1142.6567	-0.001	2	33.03	icpl	69	TGLWPINTLK	78	ctpr		ENSP00000205948	Beta-2-glycoprotein 1 Precursor (Beta-2-glycoprotein I)(Beta(2)GPI)(B2GPI)(Apolipoprotein H)(Apo-H)(Activated protein C-binding protein)(APC inhibitor)(Anticardiolipin cofactor) [Source: UniProt P02749]
3106.1.1	-4.3	4.42	1914.0041	0.003	3	36.7	iagr	22	TCPKPDDLPFSTVVPLK	38	tfye	C [23] 57.0215	ENSP00000205948	Beta-2-glycoprotein 1 Precursor (Beta-2-glycoprotein I)(Beta(2)GPI)(B2GPI)(Apolipoprotein H)(Apo-H)(Activated protein C-binding protein)(APC inhibitor)(Anticardiolipin cofactor) [Source: UniProt P02749]
3107.1.1	-1	4.26	1560.7985	-0.0099	2	28.39	enir	34	LECGDQLMRVAIR	46	aink	C [36] 57.0215	ENSP00000250263:reversed	
3108.1.1	-4.4	4.38	1956.0145	0.0013	3	36.7	iagr	22	TCPKPDDLPFSTVVPLK	38	tfye	C [23] 57.0215, T [22] 42.0106	ENSP00000205948	Beta-2-glycoprotein 1 Precursor (Beta-2-glycoprotein I)(Beta(2)GPI)(B2GPI)(Apolipoprotein H)(Apo-H)(Activated protein C-binding protein)(APC inhibitor)(Anticardiolipin cofactor) [Source: UniProt P02749]
3114.1.1	-3.2	4.43	1956.0145	0.0005	3	36.7	iagr	22	TCPKPDDLPFSTVVPLK	38	tfye	C [23] 57.0215, T [22] 42.0106	ENSP00000205948	Beta-2-glycoprotein 1 Precursor (Beta-2-glycoprotein I)(Beta(2)GPI)(B2GPI)(Apolipoprotein H)(Apo-H)(Activated protein C-binding protein)(APC inhibitor)(Anticardiolipin cofactor) [Source: UniProt P02749]
3120.1.1	-3.4	4.43	1956.0145	-0.0006	3	36.7	iagr	22	TCPKPDDLPFSTVVPLK	38	tfye	C [23] 57.0215, T [22] 42.0106	ENSP00000205948	Beta-2-glycoprotein 1 Precursor (Beta-2-glycoprotein I)(Beta(2)GPI)(B2GPI)(Apolipoprotein H)(Apo-H)(Activated protein C-binding protein)(APC inhibitor)(Anticardiolipin cofactor) [Source: UniProt P02749]
3130.1.1	-2.7	4.62	1215.6984	-0.0002	2	33.72	pkpd	28	DLPFSTVVPLK	38	tfye		ENSP00000205948	Beta-2-glycoprotein 1 Precursor (Beta-2-glycoprotein I)(Beta(2)GPI)(B2GPI)(Apolipoprotein H)(Apo-H)(Activated protein C-binding protein)(APC inhibitor)(Anticardiolipin cofactor) [Source: UniProt P02749]
3132.1.1	-2.9	3.74	1956.0145	0.0072	2	36.7	iagr	22	TCPKPDDLPFSTVVPLK	38	tfye	C [23] 57.0215, T [22] 42.0106	ENSP00000205948	Beta-2-glycoprotein 1 Precursor (Beta-2-glycoprotein I)(Beta(2)GPI)(B2GPI)(Apolipoprotein H)(Apo-H)(Activated protein C-binding protein)(APC inhibitor)(Anticardiolipin cofactor) [Source: UniProt P02749]
3144.1.1	-8	5.99	1503.7632	-0.0011	2	32.15	ctpr	83	VCPFAGILENGAVR	96	yttf	C [84] 57.0215, N [92] 0.9848	ENSP00000205948	Beta-2-glycoprotein 1 Precursor (Beta-2-glycoprotein I)(Beta(2)GPI)(B2GPI)(Apolipoprotein H)(Apo-H)(Activated protein C-binding protein)(APC inhibitor)(Anticardiolipin cofactor) [Source: UniProt P02749]
3146.1.1	-4.3	3.79	1682.9298	0.0039	2	36.4	vcap	140	IICPPPSIPTFATLR	154	vykp	C [142] 57.0215	ENSP00000205948	Beta-2-glycoprotein 1 Precursor (Beta-2-glycoprotein I)(Beta(2)GPI)(B2GPI)(Apolipoprotein H)(Apo-H)(Activated protein C-binding protein)(APC inhibitor)(Anticardiolipin cofactor) [Source: UniProt P02749]
3148.1.1	-11.5	5.9	1503.7632	-0.0007	2	32.15	ctpr	83	VCPFAGILENGAVR	96	yttf	C [84] 57.0215, N [92] 0.9848	ENSP00000205948	Beta-2-glycoprotein 1 Precursor (Beta-2-glycoprotein I)(Beta(2)GPI)(B2GPI)(Apolipoprotein H)(Apo-H)(Activated protein C-binding protein)(APC inhibitor)(Anticardiolipin cofactor) [Source: UniProt P02749]
3155.1.1	-9	5.92	1503.7632	-0.0014	2	32.15	ctpr	83	VCPFAGILENGAVR	96	yttf	C [84] 57.0215, N [92] 0.9848	ENSP00000205948	Beta-2-glycoprotein 1 Precursor (Beta-2-glycoprotein I)(Beta(2)GPI)(B2GPI)(Apolipoprotein H)(Apo-H)(Activated protein C-binding protein)(APC inhibitor)(Anticardiolipin cofactor) [Source: UniProt P02749]
3156.1.1	-11.7	4.61	1427.7781	0.0002	2	36.34	tcpk	26	PDDLPFSTVVPLK	38	tfye		ENSP00000205948	Beta-2-glycoprotein 1 Precursor (Beta-2-glycoprotein I)(Beta(2)GPI)(B2GPI)(Apolipoprotein H)(Apo-H)(Activated protein C-binding protein)(APC inhibitor)(Anticardiolipin cofactor) [Source: UniProt P02749]
3159.1.1	-7.6	4.59	1427.7781	0.001	2	36.34	tcpk	26	PDDLPFSTVVPLK	38	tfye		ENSP00000205948	Beta-2-glycoprotein 1 Precursor (Beta-2-glycoprotein I)(Beta(2)GPI)(B2GPI)(Apolipoprotein H)(Apo-H)(Activated protein C-binding protein)(APC inhibitor)(Anticardiolipin cofactor) [Source: UniProt P02749]
3160.1.1	-2.8	4.26	1933.0616	0.0019	3	47.69	ggmr	63	KFICPLTGLWPINTLK	78	ctpr	C [66] 57.0215, W [72] 31.9898	ENSP00000205948	Beta-2-glycoprotein 1 Precursor (Beta-2-glycoprotein I)(Beta(2)GPI)(B2GPI)(Apolipoprotein H)(Apo-H)(Activated protein C-binding protein)(APC inhibitor)(Anticardiolipin cofactor) [Source: UniProt P02749]
3163.1.1	-4.5	4.46	1569.7107	0.0016	2	34.45	gavr	97	YTTFEYPNTISFS	109	cntg		ENSP00000205948	Beta-2-glycoprotein 1 Precursor (Beta-2-glycoprotein I)(Beta(2)GPI)(B2GPI)(Apolipoprotein H)(Apo-H)(Activated protein C-binding protein)(APC inhibitor)(Anticardiolipin cofactor) [Source: UniProt P02749]
3164.1.1	-7.6	4.39	1427.7781	0.001	2	36.34	tcpk	26	PDDLPFSTVVPLK	38	tfye		ENSP00000205948	Beta-2-glycoprotein 1 Precursor (Beta-2-glycoprotein I)(Beta(2)GPI)(B2GPI)(Apolipoprotein H)(Apo-H)(Activated protein C-binding protein)(APC inhibitor)(Anticardiolipin cofactor) [Source: UniProt P02749]
3165.1.1	-4.2	4.45	1933.0616	0.0017	3	47.69	ggmr	63	KFICPLTGLWPINTLK	78	ctpr	C [66] 57.0215, W [72] 31.9898	ENSP00000205948	Beta-2-glycoprotein 1 Precursor (Beta-2-glycoprotein I)(Beta(2)GPI)(B2GPI)(Apolipoprotein H)(Apo-H)(Activated protein C-binding protein)(APC inhibitor)(Anticardiolipin cofactor) [Source: UniProt P02749]
3170.1.1	-6.1	4.44	1569.7107	0	2	34.45	gavr	97	YTTFEYPNTISFS	109	cntg		ENSP00000205948	Beta-2-glycoprotein 1 Precursor (Beta-2-glycoprotein I)(Beta(2)GPI)(B2GPI)(Apolipoprotein H)(Apo-H)(Activated protein C-binding protein)(APC inhibitor)(Anticardiolipin cofactor) [Source: UniProt P02749]
3172.1.1	-4.4	4.56	1933.0616	-0.0007	3	47.69	ggmr	63	KFICPLTGLWPINTLK	78	ctpr	C [66] 57.0215, W [72] 31.9898	ENSP00000205948	Beta-2-glycoprotein 1 Precursor (Beta-2-glycoprotein I)(Beta(2)GPI)(B2GPI)(Apolipoprotein H)(Apo-H)(Activated protein C-binding protein)(APC inhibitor)(Anticardiolipin cofactor) [Source: UniProt P02749]
3178.1.1	-2.7	4.31	1569.7107	0.0004	2	34.45	gavr	97	YTTFEYPNTISFS	109	cntg		ENSP00000205948	Beta-2-glycoprotein 1 Precursor (Beta-2-glycoprotein I)(Beta(2)GPI)(B2GPI)(Apolipoprotein H)(Apo-H)(Activated protein C-binding protein)(APC inhibitor)(Anticardiolipin cofactor) [Source: UniProt P02749]
3179.1.1	-4.9	4.58	1503.7632	0.0006	3	32.15	ctpr	83	VCPFAGILENGAVR	96	yttf	C [84] 57.0215, N [92] 0.9848	ENSP00000205948	Beta-2-glycoprotein 1 Precursor (Beta-2-glycoprotein I)(Beta(2)GPI)(B2GPI)(Apolipoprotein H)(Apo-H)(Activated protein C-binding protein)(APC inhibitor)(Anticardiolipin cofactor) [Source: UniProt P02749]
3184.1.1	-8.1	4.16	2715.292	0.011	3	38.86	revk	205	CPFPSRPDNGFVNYPAK	227	dkat	C [205] 57.0215, N [213] 0.9848, C [205]	ENSP00000205948	Beta-2-glycoprotein 1 Precursor (Beta-2-glycoprotein I)(Beta(2)GPI)(B2GPI)(Apolipoprotein H)(Apo-H)(Activated protein C-binding protein)(APC inhibitor)(Anticardiolipin cofactor) [Source: UniProt P02749]
3185.1.1	-7.1	4.37	1503.7632	0.0004	3	32.15	ctpr	83	VCPFAGILENGAVR	96	yttf	C [84] 57.0215, N [92] 0.9848	ENSP00000205948	Beta-2-glycoprotein 1 Precursor (Beta-2-glycoprotein I)(Beta(2)GPI)(B2GPI)(Apolipoprotein H)(Apo-H)(Activated protein C-binding protein)(APC inhibitor)(Anticardiolipin cofactor) [Source: UniProt P02749]
3186.1.1	-5.6	3.76	2246.029	0.014	2	38.81	dsak	124	CTEEGKWSPELPVCAPI	142	ppps	C [124] 57.0215, C [137] 57.0215, C [142	ENSP00000205948	Beta-2-glycoprotein 1 Precursor (Beta-2-glycoprotein I)(Beta(2)GPI)(B2GPI)(Apolipoprotein H)(Apo-H)(Activated protein C-binding protein)(APC inhibitor)(Anticardiolipin cofactor) [Source: UniProt P02749]
3189.1.1	-6.1	4.26	1914.0041	0.0021	3	36.7	iagr	22	TCPKPDDLPFSTVVPLK	38	tfye	C [23] 57.0215	ENSP00000205948	Beta-2-glycoprotein 1 Precursor (Beta-2-glycoprotein I)(Beta(2)GPI)(B2GPI)(Apolipoprotein H)(Apo-H)(Activated protein C-binding protein)(APC inhibitor)(Anticardiolipin cofactor) [Source: UniProt P02749]
3190.1.1	-4	4.4	1503.7632	0.0081	3	32.15	ctpr	83	VCPFAGILENGAVR	96	yttf	C [84] 57.0215, N [92] 0.9848	ENSP00000205948	Beta-2-glycoprotein 1 Precursor (Beta-2-glycoprotein I)(Beta(2)GPI)(B2GPI)(Apolipoprotein H)(Apo-H)(Activated protein C-binding protein)(APC inhibitor)(Anticardiolipin cofactor) [Source: UniProt P02749]
3194.1.1	-3.6	4.32	1914.0041	-0.0007	3	36.7	iagr	22	TCPKPDDLPFSTVVPLK	38	tfye	C [23] 57.0215	ENSP00000205948	Beta-2-glycoprotein 1 Precursor (Beta-2-glycoprotein I)(Beta(2)GPI)(B2GPI)(Apolipoprotein H)(Apo-H)(Activated protein C-binding protein)(APC inhibitor)(Anticardiolipin cofactor) [Source: UniProt P02749]
3195.1.1	-3.6	3.98	1119.5503	0.001	2	29.1	ctpr	83	VCPFAGILEN	92	gavr	C [84] 57.0215	ENSP00000205948	Beta-2-glycoprotein 1 Precursor (Beta-2-glycoprotein I)(Beta(2)GPI)(B2GPI)(Apolipoprotein H)(Apo-H)(Activated protein C-binding protein)(APC inhibitor)(Anticardiolipin cofactor) [Source: UniProt P02749]
3198.1.1	-4.6	4.31	1914.0041	0.0025	3	36.7	iagr	22	TCPKPDDLPFSTVVPLK	38	tfye	C [23] 57.0215	ENSP00000205948	Beta-2-glycoprotein 1 Precursor (Beta-2-glycoprotein I)(Beta(2)GPI)(B2GPI)(Apolipoprotein H)(Apo-H)(Activated protein C-binding protein)(APC inhibitor)(Anticardiolipin cofactor) [Source: UniProt P02749]
3205.1.1	-8.7	4.12	2715.292	0.013	3	38.86	revk	205	CPFPSRPDNGFVNYPAK	227	dkat	C [205] 57.0215, N [213] 0.9848, C [205]	ENSP00000205948	Beta-2-glycoprotein 1 Precursor (Beta-2-glycoprotein I)(Beta(2)GPI)(B2GPI)(Apolipoprotein H)(Apo-H)(Activated protein C-binding protein)(APC inhibitor)(Anticardiolipin cofactor) [Source: UniProt P02749]
3206.1.1	-1.4	4.38	1485.7431	0.0061	2	25.65	ygrk	1470	GIQAEEIGLPESDK	1483	qgat		ENSP00000263857:reversed	
3223.1.1	-2.3	4.56	1968.0185	0.0002	2	26.14	mlrk	20	EVPKSPHDYTVSSGIVPF	37	dvft		ENSP00000382333:reversed	
3224.1.1	-4.1	4.81	4278.0774	0.0024	4	45.33	iagr	22	TCPKPDDLPFSTVVPLKT	58	ggmr	C [23] 57.0215, C [51] 57.0215	ENSP00000205948	Beta-2-glycoprotein 1 Precursor (Beta-2-glycoprotein I)(Beta(2)GPI)(B2GPI)(Apolipoprotein H)(Apo-H)(Activated protein C-binding protein)(APC inhibitor)(Anticardiolipin cofactor) [Source: UniProt P02749]
3229.1.1	-3.1	4.87	4278.0774	0.0058	4	45.33	iagr	22	TCPKPDDLPFSTVVPLKT	58	ggmr	C [23] 57.0215, C [51] 57.0215	ENSP00000205948	Beta-2-glycoprotein 1 Precursor (Beta-2-glycoprotein I)(Beta(2)GPI)(B2GPI)(Apolipoprotein H)(Apo-H)(Activated protein C-binding protein)(APC inhibitor)(Anticardiolipin cofactor) [Source: UniProt P02749]
3237.1.1	-2.8	4.75	4278.0774	0.0087	4	45.33	iagr	22	TCPKPDDLPFSTVVPLKT	58	ggmr	C [23] 57.0215, C [51] 57.0215	ENSP00000205948	Beta-2-glycoprotein 1 Precursor (Beta-2-glycoprotein I)(Beta(2)GPI)(B2GPI)(Apolipoprotein H)(Apo-H)(Activated protein C-binding protein)(APC inhibitor)(Anticardiolipin cofactor) [Source: UniProt P02749]
3238.1.1	-2.8	4.69	1503.7632	0.0019	2	32.15	ctpr	83	VCPFAGILENGAVR	96	yttf	C [84] 57.0215, N [92] 0.9848	ENSP00000205948	Beta-2-glycoprotein 1 Precursor (Beta-2-glycoprotein I)(Beta(2)GPI)(B2GPI)(Apolipoprotein H)(Apo-H)(Activated protein C-binding protein)(APC inhibitor)(Anticardiolipin cofactor) [Source: UniProt P02749]
3241.1.1	-5.3	4.14	2715.292	0.017	3	38.86	revk	205	CPFPSRPDNGFVNYPAK	227	dkat	C [205] 57.0215, N [213] 0.9848, C [205]	ENSP00000205948	Beta-2-glycoprotein 1 Precursor (Beta-2-glycoprotein I)(Beta(2)GPI)(B2GPI)(Apolipoprotein H)(Apo-H)(Activated protein C-binding protein)(APC inhibitor)(Anticardiolipin cofactor) [Source: UniProt P02749]
3243.1.1	-1.9	4.62	1503.7632	-0.0005	2	32.15	ctpr	83	VCPFAGILENGAVR	96	yttf	C [84] 57.0215, N [92] 0.9848	ENSP00000205948	Beta-2-glycoprotein 1 Precursor (Beta-2-glycoprotein I)(Beta(2)GPI)(B2GPI)(Apolipoprotein H)(Apo-H)(Activated protein C-binding protein)(APC inhibitor)(Anticardiolipin cofactor) [Source: UniProt P02749]
3250.1.1	-7.2	4.53	1503.7632	0.0038	2	32.15	ctpr	83	VCPFAGILENGAVR	96	yttf	C [84] 57.0215, N [92] 0.9848	ENSP00000205948	Beta-2-glycoprotein 1 Precursor (Beta-2-glycoprotein I)(Beta(2)GPI)(B2GPI)(Apolipoprotein H)(Apo-H)(Activated protein C-binding protein)(APC inhibitor)(Anticardiolipin cofactor) [Source: UniProt P02749]
3271.1.1	-4	3.86	1502.7784	0.0086	3	32.15	ctpr	83	VCPFAGILENGAVR	96	yttf	C [84] 57.0215	ENSP00000205948	Beta-2-glycoprotein 1 Precursor (Beta-2-glycoprotein I)(Beta(2)GPI)(B2GPI)(Apolipoprotein H)(Apo-H)(Activated protein C-binding protein)(APC inhibitor)(Anticardiolipin cofactor) [Source: UniProt P02749]
3273.1.1	-3.2	3.67	1851.02	-0.011	2	38.17	lpvc	138	APIICPPPSIPTFATLR	154	vykp	C [142] 57.0215	ENSP00000205948	Beta-2-glycoprotein 1 Precursor (Beta-2-glycoprotein I)(Beta(2)GPI)(B2GPI)(Apolipoprotein H)(Apo-H)(Activated protein C-binding protein)(APC inhibitor)(Anticardiolipin cofactor) [Source: UniProt P02749]
3276.1.1	-2.3	4.05	1914.0041	-0.0001	3	36.7	iagr	22	TCPKPDDLPFSTVVPLK	38	tfye	C [23] 57.0215	ENSP00000205948	Beta-2-glycoprotein 1 Precursor (Beta-2-glycoprotein I)(Beta(2)GPI)(B2GPI)(Apolipoprotein H)(Apo-H)(Activated protein C-binding protein)(APC inhibitor)(Anticardiolipin cofactor) [Source: UniProt P02749]
3279.1.1	-3.5	3.99	1502.7784	0.0009	3	32.15	ctpr	83	VCPFAGILENGAVR	96	yttf	C [84] 57.0215	ENSP00000205948	Beta-2-glycoprotein 1 Precursor (Beta-2-glycoprotein I)(Beta(2)GPI)(B2GPI)(Apolipoprotein H)(Apo-H)(Activated protein C-binding protein)(APC inhibitor)(Anticardiolipin cofactor) [Source: UniProt P02749]
3282.1.1	-3.7	4.18	1914.0041	-0.0041	3	36.7	iagr	22	TCPKPDDLPFSTVVPLK	38	tfye	C [23] 57.0215	ENSP00000205948	Beta-2-glycoprotein 1 Precursor (Beta-2-glycoprotein I)(Beta(2)GPI)(B2GPI)(Apolipoprotein H)(Apo-H)(Activated protein C-binding protein)(APC inhibitor)(Anticardiolipin cofactor) [Source: UniProt P02749]
3291.1.1	-4.1	4.2	1914.0041	0.001	3	36.7	iagr	22	TCPKPDDLPFSTVVPLK	38	tfye	C [23] 57.0215	ENSP00000205948	Beta-2-glycoprotein 1 Precursor (Beta-2-glycoprotein I)(Beta(2)GPI)(B2GPI)(Apolipoprotein H)(Apo-H)(Activated protein C-binding protein)(APC inhibitor)(Anticardiolipin cofactor) [Source: UniProt P02749]
3300.1.1	-7.2	3.99	2715.292	0.013	3	38.86	revk	205	CPFPSRPDNGFVNYPAK	227	dkat	C [205] 57.0215, N [213] 0.9848, C [205]	ENSP00000205948	Beta-2-glycoprotein 1 Precursor (Beta-2-glycoprotein I)(Beta(2)GPI)(B2GPI)(Apolipoprotein H)(Apo-H)(Activated protein C-binding protein)(APC inhibitor)(Anticardiolipin cofactor) [Source: UniProt P02749]
3303.1.1	-3	3.76	1469.7886	-0.005	2	36.34	tcpk	26	PDDLPFSTVVPLK	38	tfye	P [26] 42.0106	ENSP00000205948	Beta-2-glycoprotein 1 Precursor (Beta-2-glycoprotein I)(Beta(2)GPI)(B2GPI)(Apolipoprotein H)(Apo-H)(Activated protein C-binding protein)(APC inhibitor)(Anticardiolipin cofactor) [Source: UniProt P02749]
3305.1.1	-6	3.43	2068.8838	0.0064	2	27.19	kekk	307	CSYTEDAQCIDGTIEVPI	324	cfke	C [307] 57.0215, C [315] 57.0215, C [307	ENSP00000205948	Beta-2-glycoprotein 1 Precursor (Beta-2-glycoprotein I)(Beta(2)GPI)(B2GPI)(Apolipoprotein H)(Apo-H)(Activated protein C-binding protein)(APC inhibitor)(Anticardiolipin cofactor) [Source: UniProt P02749]
3313.1.1	-2	4.63	1534.674	2.02	2	3.8	iggr	617	GSSSGGVKSSGGSSSVK	633	fvst	S [626] 79.9663	ENSP00000252244	Keratin, type II cytoskeletal 1 (Cytokeratin-1) (CK-1) (Keratin-1) (K1) (67 kDa cytokeratin) (Hair alpha protein). Source: Uniprot/SWISSPROT P04264
3336.1.1	-1.6	4.39	1502.778	0.99	2	32.15	ctpr	83	VCPFAGILENGAVR	96	yttf	C [84] 57.0215	ENSP00000205948	Beta-2-glycoprotein 1 Precursor (Beta-2-glycoprotein I)(Beta(2)GPI)(B2GPI)(Apolipoprotein H)(Apo-H)(Activated protein C-binding protein)(APC inhibitor)(Anticardiolipin cofactor) [Source: UniProt P02749]
3340.1.1	-6.2	4.28	1503.7632	0.0059	2	32.15	ctpr	83	VCPFAGILENGAVR	96	yttf	C [84] 57.0215, N [92] 0.9848	ENSP00000205948	Beta-2-glycoprotein 1 Precursor (Beta-2-glycoprotein I)(Beta(2)GPI)(B2GPI)(Apolipoprotein H)(Apo-H)(Activated protein C-binding protein)(APC inhibitor)(Anticardiolipin cofactor) [Source: UniProt P02749]
3354.1.1	-5.9	3.99	2715.292	0.007	3	38.86	revk	205	CPFPSRPDNGFVNYPAK	227	dkat	C [205] 57.0215, N [213] 0.9848, C [205]	ENSP00000205948	Beta-2-glycoprotein 1 Precursor (Beta-2-glycoprotein I)(Beta(2)GPI)(B2GPI)(Apolipoprotein H)(Apo-H)(Activated protein C-binding protein)(APC inhibitor)(Anticardiolipin cofactor) [Source: UniProt P02749]
3357.1.1	-1.2	4.01	2732.3185	-0.001	4	38.86	revk	205	CPFPSRPDNGFVNYPAK	227	dkat	C [205] 57.0215, N [213] 0.9848	ENSP00000205948	Beta-2-glycoprotein 1 Precursor (Beta-2-glycoprotein I)(Beta(2)GPI)(B2GPI)(Apolipoprotein H)(Apo-H)(Activated protein C-binding protein)(APC inhibitor)(Anticardiolipin cofactor) [Source: UniProt P02749]
3359.1.1	-3.4	4.12	1503.7632	0.006	2	32.15	ctpr	83	VCPFAGILENGAVR	96	yttf	C [84] 57.0215, N [92] 0.9848	ENSP00000205948	Beta-2-glycoprotein 1 Precursor (Beta-2-glycoprotein I)(Beta(2)GPI)(B2GPI)(Apolipoprotein H)(Apo-H)(Activated protein C-binding protein)(APC inhibitor)(Anticardiolipin cofactor) [Source: UniProt P02749]
3372.1.1	-2.2	4.21	1482.6788	-0.0016	2	34.42	gavr	97	YTTFEYPNTISF	108	scnt		ENSP00000205948	Beta-2-glycoprotein 1 Precursor (Beta-2-glycoprotein I)(Beta(2)GPI)(B2GPI)(Apolipoprotein H)(Apo-H)(Activated protein C-binding protein)(APC inhibitor)(Anticardiolipin cofactor) [Source: UniProt P02749]
3375.1.1	-1.8	4.19	1914.0041	-0.0025	3	36.7	iagr	22	TCPKPDDLPFSTVVPLK	38	tfye	C [23] 57.0215	ENSP00000205948	Beta-2-glycoprotein 1 Precursor (Beta-2-glycoprotein I)(Beta(2)GPI)(B2GPI)(Apolipoprotein H)(Apo-H)(Activated protein C-binding protein)(APC inhibitor)(Anticardiolipin cofactor) [Source: UniProt P02749]
3379.1.1	-3.2	4.13	1914.0041	0.001	3	36.7	iagr	22	TCPKPDDLPFSTVVPLK	38	tfye	C [23] 57.0215	ENSP00000205948	Beta-2-glycoprotein 1 Precursor (Beta-2-glycoprotein I)(Beta(2)GPI)(B2GPI)(Apolipoprotein H)(Apo-H)(Activated protein C-binding protein)(APC inhibitor)(Anticardiolipin cofactor) [Source: UniProt P02749]
3381.1.1	-3.3	4.13	1914.0041	-0.001	3	36.7	iagr	22	TCPKPDDLPFSTVVPLK	38	tfye	C [23] 57.0215	ENSP00000205948	Beta-2-glycoprotein 1 Precursor (Beta-2-glycoprotein I)(Beta(2)GPI)(B2GPI)(Apolipoprotein H)(Apo-H)(Activated protein C-binding protein)(APC inhibitor)(Anticardiolipin cofactor) [Source: UniProt P02749]
3382.1.1	-1.6	3.78	3068.362	-0.019	3	41.58	gavr	97	YTTFEYPNTISFSCNTGF	123	ctee	C [110] 57.0215	ENSP00000205948	Beta-2-glycoprotein 1 Precursor (Beta-2-glycoprotein I)(Beta(2)GPI)(B2GPI)(Apolipoprotein H)(Apo-H)(Activated protein C-binding protein)(APC inhibitor)(Anticardiolipin cofactor) [Source: UniProt P02749]
3389.1.1	-3.7	3.94	2715.292	0.013	3	38.86	revk	205	CPFPSRPDNGFVNYPAK	227	dkat	C [205] 57.0215, N [213] 0.9848, C [205]	ENSP00000205948	Beta-2-glycoprotein 1 Precursor (Beta-2-glycoprotein I)(Beta(2)GPI)(B2GPI)(Apolipoprotein H)(Apo-H)(Activated protein C-binding protein)(APC inhibitor)(Anticardiolipin cofactor) [Source: UniProt P02749]
3395.1.1	-4.7	3.89	2715.292	0.012	3	38.86	revk	205	CPFPSRPDNGFVNYPAK	227	dkat	C [205] 57.0215, N [217] 0.9848, C [205]	ENSP00000205948	Beta-2-glycoprotein 1 Precursor (Beta-2-glycoprotein I)(Beta(2)GPI)(B2GPI)(Apolipoprotein H)(Apo-H)(Activated protein C-binding protein)(APC inhibitor)(Anticardiolipin cofactor) [Source: UniProt P02749]
3405.1.1	-4.7	3.82	1204.5166	0	2	32.64	cevk	73	CMPTFQFFK	81	kgqk	C [73] 57.0215, M [74] 15.9949, C [73] -1	ENSP00000363641	Thioredoxin (Trx)(ATL-derived factor)(ADF)(Surface-associated sulphydryl protein)(SASP) [Source: UniProt P10599]
3408.1.1	-4.1	3.86	1204.5166	0.0009	2	32.64	cevk	73	CMPTFQFFK	81	kgqk	C [73] 57.0215, M [74] 15.9949, C [73] -1	ENSP00000363641	Thioredoxin (Trx)(ATL-derived factor)(ADF)(Surface-associated sulphydryl protein)(SASP) [Source: UniProt P10599]
3412.1.1	-3.6	3.82	1204.5166	-0.0005	2	32.64	cevk	73	CMPTFQFFK	81	kgqk	C [73] 57.0215, M [74] 15.9949, C [73] -1	ENSP00000363641	Thioredoxin (Trx)(ATL-derived factor)(ADF)(Surface-associated sulphydryl protein)(SASP) [Source: UniProt P10599]
3418.1.1	-4.3	4.02	1503.7632	0.008	2	32.15	ctpr	83	VCPFAGILENGAVR	96	yttf	C [84] 57.0215, N [92] 0.9848	ENSP00000205948	Beta-2-glycoprotein 1 Precursor (Beta-2-glycoprotein I)(Beta(2)GPI)(B2GPI)(Apolipoprotein H)(Apo-H)(Activated protein C-binding protein)(APC inhibitor)(Anticardiolipin cofactor) [Source: UniProt P02749]
3423.1.1	-1.2	3.97	2732.3185	0.0014	4	38.86	revk	205	CPFPSRPDNGFVNYPAK	227	dkat	C [205] 57.0215, N [213] 0.9848	ENSP00000205948	Beta-2-glycoprotein 1 Precursor (Beta-2-glycoprotein I)(Beta(2)GPI)(B2GPI)(Apolipoprotein H)(Apo-H)(Activated protein C-binding protein)(APC inhibitor)(Anticardiolipin cofactor) [Source: UniProt P02749]
3430.1.1	-3.1	4.06	1503.7632	0.0072	2	32.15	ctpr	83	VCPFAGILENGAVR	96	yttf	C [84] 57.0215, N [92] 0.9848	ENSP00000205948	Beta-2-glycoprotein 1 Precursor (Beta-2-glycoprotein I)(Beta(2)GPI)(B2GPI)(Apolipoprotein H)(Apo-H)(Activated protein C-binding protein)(APC inhibitor)(Anticardiolipin cofactor) [Source: UniProt P02749]

3442.1.1	-1.7	4.11	1502.7784	-0.006	2	32.15	ctpr	83	VCPFAGILENGAVR	96	yttf	C [84] 57.0215	ENSP00000205948	Beta-2-glycoprotein 1 Precursor (Beta-2-glycoprotein I)(Beta(2)GPI)(B2GPI)(Apolipoprotein H)(Apo-H)(Activated protein C-binding protein)(APC inhibitor)(Anticardiolipin cofactor) [Source: UniProt P02749]
3443.1.1	-3.2	4.1	1914.0041	-0.004	3	36.7	iagr	22	TCPKPDDLPFSTVVPLK	38	tfye	C [23] 57.0215	ENSP00000205948	Beta-2-glycoprotein 1 Precursor (Beta-2-glycoprotein I)(Beta(2)GPI)(B2GPI)(Apolipoprotein H)(Apo-H)(Activated protein C-binding protein)(APC inhibitor)(Anticardiolipin cofactor) [Source: UniProt P02749]
3449.1.1	-2.9	3.98	1914.0041	0.001	3	36.7	iagr	22	TCPKPDDLPFSTVVPLK	38	tfye	C [23] 57.0215	ENSP00000205948	Beta-2-glycoprotein 1 Precursor (Beta-2-glycoprotein I)(Beta(2)GPI)(B2GPI)(Apolipoprotein H)(Apo-H)(Activated protein C-binding protein)(APC inhibitor)(Anticardiolipin cofactor) [Source: UniProt P02749]
3456.1.1	-4.1	4.02	1914.0041	-0.0003	3	36.7	iagr	22	TCPKPDDLPFSTVVPLK	38	tfye	C [23] 57.0215	ENSP00000205948	Beta-2-glycoprotein 1 Precursor (Beta-2-glycoprotein I)(Beta(2)GPI)(B2GPI)(Apolipoprotein H)(Apo-H)(Activated protein C-binding protein)(APC inhibitor)(Anticardiolipin cofactor) [Source: UniProt P02749]
3458.1.1	-3	4.07	1804.9666	0.0054	2	46.54	gmrk	64	FICPLTGLWPINTLK	78	ctpr	C [66] 57.0215, W [72] 31.9898	ENSP00000205948	Beta-2-glycoprotein 1 Precursor (Beta-2-glycoprotein I)(Beta(2)GPI)(B2GPI)(Apolipoprotein H)(Apo-H)(Activated protein C-binding protein)(APC inhibitor)(Anticardiolipin cofactor) [Source: UniProt P02749]
3460.1.1	-5.4	4.07	1804.9666	-0.0006	2	46.54	gmrk	64	FICPLTGLWPINTLK	78	ctpr	C [66] 57.0215, W [72] 31.9898	ENSP00000205948	Beta-2-glycoprotein 1 Precursor (Beta-2-glycoprotein I)(Beta(2)GPI)(B2GPI)(Apolipoprotein H)(Apo-H)(Activated protein C-binding protein)(APC inhibitor)(Anticardiolipin cofactor) [Source: UniProt P02749]
3461.1.1	-4.7	3.69	1933.0616	0.007	3	47.69	ggmr	63	KFICPLTGLWPINTLK	78	ctpr	C [66] 57.0215, W [72] 31.9898	ENSP00000205948	Beta-2-glycoprotein 1 Precursor (Beta-2-glycoprotein I)(Beta(2)GPI)(B2GPI)(Apolipoprotein H)(Apo-H)(Activated protein C-binding protein)(APC inhibitor)(Anticardiolipin cofactor) [Source: UniProt P02749]
3465.1.1	-4.5	4.44	1804.9666	0.0037	2	46.54	gmrk	64	FICPLTGLWPINTLK	78	ctpr	C [66] 57.0215, W [72] 31.9898	ENSP00000205948	Beta-2-glycoprotein 1 Precursor (Beta-2-glycoprotein I)(Beta(2)GPI)(B2GPI)(Apolipoprotein H)(Apo-H)(Activated protein C-binding protein)(APC inhibitor)(Anticardiolipin cofactor) [Source: UniProt P02749]
3466.1.1	-7.4	3.85	1933.0616	-0.0027	3	47.69	ggmr	63	KFICPLTGLWPINTLK	78	ctpr	C [66] 57.0215, W [72] 31.9898	ENSP00000205948	Beta-2-glycoprotein 1 Precursor (Beta-2-glycoprotein I)(Beta(2)GPI)(B2GPI)(Apolipoprotein H)(Apo-H)(Activated protein C-binding protein)(APC inhibitor)(Anticardiolipin cofactor) [Source: UniProt P02749]
3470.1.1	-3.6	3.89	1933.0616	0.0006	3	47.69	ggmr	63	KFICPLTGLWPINTLK	78	ctpr	C [66] 57.0215, W [72] 31.9898	ENSP00000205948	Beta-2-glycoprotein 1 Precursor (Beta-2-glycoprotein I)(Beta(2)GPI)(B2GPI)(Apolipoprotein H)(Apo-H)(Activated protein C-binding protein)(APC inhibitor)(Anticardiolipin cofactor) [Source: UniProt P02749]
3474.1.1	-10.7	4.76	3555.7321	-0.0008	3	48.28	dsak	124	CTEEGKWSPELPVCAPI	154	vykp	C [124] 57.0215, C [137] 57.0215, C [142	ENSP00000205948	Beta-2-glycoprotein 1 Precursor (Beta-2-glycoprotein I)(Beta(2)GPI)(B2GPI)(Apolipoprotein H)(Apo-H)(Activated protein C-binding protein)(APC inhibitor)(Anticardiolipin cofactor) [Source: UniProt P02749]
3477.1.1	-10.7	4.72	3555.7321	0.0022	3	48.28	dsak	124	CTEEGKWSPELPVCAPI	154	vykp	C [124] 57.0215, C [137] 57.0215, C [142	ENSP00000205948	Beta-2-glycoprotein 1 Precursor (Beta-2-glycoprotein I)(Beta(2)GPI)(B2GPI)(Apolipoprotein H)(Apo-H)(Activated protein C-binding protein)(APC inhibitor)(Anticardiolipin cofactor) [Source: UniProt P02749]
3479.1.1	-4.5	4.13	1804.9666	-0.0015	3	46.54	gmrk	64	FICPLTGLWPINTLK	78	ctpr	C [66] 57.0215, W [72] 31.9898	ENSP00000205948	Beta-2-glycoprotein 1 Precursor (Beta-2-glycoprotein I)(Beta(2)GPI)(B2GPI)(Apolipoprotein H)(Apo-H)(Activated protein C-binding protein)(APC inhibitor)(Anticardiolipin cofactor) [Source: UniProt P02749]
3481.1.1	-7.9	4.71	3555.7321	0	3	48.28	dsak	124	CTEEGKWSPELPVCAPI	154	vykp	C [124] 57.0215, C [137] 57.0215, C [142	ENSP00000205948	Beta-2-glycoprotein 1 Precursor (Beta-2-glycoprotein I)(Beta(2)GPI)(B2GPI)(Apolipoprotein H)(Apo-H)(Activated protein C-binding protein)(APC inhibitor)(Anticardiolipin cofactor) [Source: UniProt P02749]
3482.1.1	-2	4.15	3555.7321	0.0095	4	48.28	dsak	124	CTEEGKWSPELPVCAPI	154	vykp	C [124] 57.0215, C [137] 57.0215, C [142	ENSP00000205948	Beta-2-glycoprotein 1 Precursor (Beta-2-glycoprotein I)(Beta(2)GPI)(B2GPI)(Apolipoprotein H)(Apo-H)(Activated protein C-binding protein)(APC inhibitor)(Anticardiolipin cofactor) [Source: UniProt P02749]
3483.1.1	-7	4.17	1804.9666	0.003	3	46.54	gmrk	64	FICPLTGLWPINTLK	78	ctpr	C [66] 57.0215, W [72] 31.9898	ENSP00000205948	Beta-2-glycoprotein 1 Precursor (Beta-2-glycoprotein I)(Beta(2)GPI)(B2GPI)(Apolipoprotein H)(Apo-H)(Activated protein C-binding protein)(APC inhibitor)(Anticardiolipin cofactor) [Source: UniProt P02749]
3487.1.1	-5.7	4.12	1804.9666	-0.0004	3	46.54	gmrk	64	FICPLTGLWPINTLK	78	ctpr	C [66] 57.0215, W [72] 31.9898	ENSP00000205948	Beta-2-glycoprotein 1 Precursor (Beta-2-glycoprotein I)(Beta(2)GPI)(B2GPI)(Apolipoprotein H)(Apo-H)(Activated protein C-binding protein)(APC inhibitor)(Anticardiolipin cofactor) [Source: UniProt P02749]
3496.1.1	-4.1	4.23	3555.7321	-0.0037	4	48.28	dsak	124	CTEEGKWSPELPVCAPI	154	vykp	C [124] 57.0215, C [137] 57.0215, C [142	ENSP00000205948	Beta-2-glycoprotein 1 Precursor (Beta-2-glycoprotein I)(Beta(2)GPI)(B2GPI)(Apolipoprotein H)(Apo-H)(Activated protein C-binding protein)(APC inhibitor)(Anticardiolipin cofactor) [Source: UniProt P02749]
3506.1.1	-2.3	4.1	3539.7372	-0.0014	4	48.28	dsak	124	CTEEGKWSPELPVCAPI	154	vykp	C [124] 57.0215, C [137] 57.0215, C [142	ENSP00000205948	Beta-2-glycoprotein 1 Precursor (Beta-2-glycoprotein I)(Beta(2)GPI)(B2GPI)(Apolipoprotein H)(Apo-H)(Activated protein C-binding protein)(APC inhibitor)(Anticardiolipin cofactor) [Source: UniProt P02749]
3516.1.1	-2.8	3.92	1503.7632	0.0059	2	32.15	ctpr	83	VCPFAGILENGAVR	96	yttf	C [84] 57.0215, N [92] 0.9848	ENSP00000205948	Beta-2-glycoprotein 1 Precursor (Beta-2-glycoprotein I)(Beta(2)GPI)(B2GPI)(Apolipoprotein H)(Apo-H)(Activated protein C-binding protein)(APC inhibitor)(Anticardiolipin cofactor) [Source: UniProt P02749]
3518.1.1	-2.7	4.68	3539.7372	0.0018	4	48.28	dsak	124	CTEEGKWSPELPVCAPI	154	vykp	C [124] 57.0215, C [137] 57.0215, C [142	ENSP00000205948	Beta-2-glycoprotein 1 Precursor (Beta-2-glycoprotein I)(Beta(2)GPI)(B2GPI)(Apolipoprotein H)(Apo-H)(Activated protein C-binding protein)(APC inhibitor)(Anticardiolipin cofactor) [Source: UniProt P02749]
3520.1.1	-5.5	4.96	3539.7372	0.0002	3	48.28	dsak	124	CTEEGKWSPELPVCAPI	154	vykp	C [124] 57.0215, C [137] 57.0215, C [142	ENSP00000205948	Beta-2-glycoprotein 1 Precursor (Beta-2-glycoprotein I)(Beta(2)GPI)(B2GPI)(Apolipoprotein H)(Apo-H)(Activated protein C-binding protein)(APC inhibitor)(Anticardiolipin cofactor) [Source: UniProt P02749]
3521.1.1	-6.4	3.94	1302.7151	0.0003	2	33.09	dnnr	344	SLDLDSIIAEVK	355	aqye		ENSP00000252244	Keratin, type II cytoskeletal 1 (Cytokeratin-1) (CK-1) (Keratin-1) (K1) (67 kDa cytokeratin) (Hair alpha protein). Source: Uniprot/SWISSPROT P04264
3523.1.1	-9.7	4.98	3539.7372	-0.0027	3	48.28	dsak	124	CTEEGKWSPELPVCAPI	154	vykp	C [124] 57.0215, C [137] 57.0215, C [142	ENSP00000205948	Beta-2-glycoprotein 1 Precursor (Beta-2-glycoprotein I)(Beta(2)GPI)(B2GPI)(Apolipoprotein H)(Apo-H)(Activated protein C-binding protein)(APC inhibitor)(Anticardiolipin cofactor) [Source: UniProt P02749]
3524.1.1	-5.7	3.98	1302.7151	0.001	2	33.09	dnnr	344	SLDLDSIIAEVK	355	aqye		ENSP00000252244	Keratin, type II cytoskeletal 1 (Cytokeratin-1) (CK-1) (Keratin-1) (K1) (67 kDa cytokeratin) (Hair alpha protein). Source: Uniprot/SWISSPROT P04264
3526.1.1	-6.9	4.96	3539.7372	-0.0056	3	48.28	dsak	124	CTEEGKWSPELPVCAPI	154	vykp	C [124] 57.0215, C [137] 57.0215, C [142	ENSP00000205948	Beta-2-glycoprotein 1 Precursor (Beta-2-glycoprotein I)(Beta(2)GPI)(B2GPI)(Apolipoprotein H)(Apo-H)(Activated protein C-binding protein)(APC inhibitor)(Anticardiolipin cofactor) [Source: UniProt P02749]
3527.1.1	-6.4	4.01	1302.7151	0.0004	2	33.09	dnnr	344	SLDLDSIIAEVK	355	aqye		ENSP00000252244	Keratin, type II cytoskeletal 1 (Cytokeratin-1) (CK-1) (Keratin-1) (K1) (67 kDa cytokeratin) (Hair alpha protein). Source: Uniprot/SWISSPROT P04264
3528.1.1	-4.9	4.07	2715.292	0.017	3	38.86	revk	205	CPFPSRPDNGFVNYPAK	227	dkat	C [205] 57.0215, N [213] 0.9848, C [205]	ENSP00000205948	Beta-2-glycoprotein 1 Precursor (Beta-2-glycoprotein I)(Beta(2)GPI)(B2GPI)(Apolipoprotein H)(Apo-H)(Activated protein C-binding protein)(APC inhibitor)(Anticardiolipin cofactor) [Source: UniProt P02749]
3529.1.1	-6.3	3.95	1503.7632	0.0071	2	32.15	ctpr	83	VCPFAGILENGAVR	96	yttf	C [84] 57.0215, N [92] 0.9848	ENSP00000205948	Beta-2-glycoprotein 1 Precursor (Beta-2-glycoprotein I)(Beta(2)GPI)(B2GPI)(Apolipoprotein H)(Apo-H)(Activated protein C-binding protein)(APC inhibitor)(Anticardiolipin cofactor) [Source: UniProt P02749]
3536.1.1	-1.4	4.04	2383.0911	-0.0075	3	33.69	vplk	39	TFYEPGEEITYSCKPGYV	58	ggmr	C [51] 57.0215	ENSP00000205948	Beta-2-glycoprotein 1 Precursor (Beta-2-glycoprotein I)(Beta(2)GPI)(B2GPI)(Apolipoprotein H)(Apo-H)(Activated protein C-binding protein)(APC inhibitor)(Anticardiolipin cofactor) [Source: UniProt P02749]
3537.1.1	-2.6	4.02	1914.0041	0.0028	3	36.7	iagr	22	TCPKPDDLPFSTVVPLK	38	tfye	C [23] 57.0215	ENSP00000205948	Beta-2-glycoprotein 1 Precursor (Beta-2-glycoprotein I)(Beta(2)GPI)(B2GPI)(Apolipoprotein H)(Apo-H)(Activated protein C-binding protein)(APC inhibitor)(Anticardiolipin cofactor) [Source: UniProt P02749]
3540.1.1	-4	4.02	1804.9666	0.0034	2	46.54	gmrk	64	FICPLTGLWPINTLK	78	ctpr	C [66] 57.0215, W [72] 31.9898	ENSP00000205948	Beta-2-glycoprotein 1 Precursor (Beta-2-glycoprotein I)(Beta(2)GPI)(B2GPI)(Apolipoprotein H)(Apo-H)(Activated protein C-binding protein)(APC inhibitor)(Anticardiolipin cofactor) [Source: UniProt P02749]
3542.1.1	-3.2	3.92	1503.7632	0.0039	2	32.15	ctpr	83	VCPFAGILENGAVR	96	yttf	C [84] 57.0215, N [92] 0.9848	ENSP00000205948	Beta-2-glycoprotein 1 Precursor (Beta-2-glycoprotein I)(Beta(2)GPI)(B2GPI)(Apolipoprotein H)(Apo-H)(Activated protein C-binding protein)(APC inhibitor)(Anticardiolipin cofactor) [Source: UniProt P02749]
3548.1.1	-4.5	3.94	1804.9666	0.006	2	46.54	gmrk	64	FICPLTGLWPINTLK	78	ctpr	C [66] 57.0215, W [72] 31.9898	ENSP00000205948	Beta-2-glycoprotein 1 Precursor (Beta-2-glycoprotein I)(Beta(2)GPI)(B2GPI)(Apolipoprotein H)(Apo-H)(Activated protein C-binding protein)(APC inhibitor)(Anticardiolipin cofactor) [Source: UniProt P02749]
3551.1.1	-1.9	3.93	1804.967	0.012	2	46.54	gmrk	64	FICPLTGLWPINTLK	78	ctpr	C [66] 57.0215, W [72] 31.9898	ENSP00000205948	Beta-2-glycoprotein 1 Precursor (Beta-2-glycoprotein I)(Beta(2)GPI)(B2GPI)(Apolipoprotein H)(Apo-H)(Activated protein C-binding protein)(APC inhibitor)(Anticardiolipin cofactor) [Source: UniProt P02749]
3552.1.1	-2.7	3.99	1914.0041	0.0004	3	36.7	iagr	22	TCPKPDDLPFSTVVPLK	38	tfye	C [23] 57.0215	ENSP00000205948	Beta-2-glycoprotein 1 Precursor (Beta-2-glycoprotein I)(Beta(2)GPI)(B2GPI)(Apolipoprotein H)(Apo-H)(Activated protein C-binding protein)(APC inhibitor)(Anticardiolipin cofactor) [Source: UniProt P02749]
3555.1.1	-3.4	4.43	3555.7321	-0.0015	3	48.28	dsak	124	CTEEGKWSPELPVCAPI	154	vykp	C [124] 57.0215, C [137] 57.0215, C [142	ENSP00000205948	Beta-2-glycoprotein 1 Precursor (Beta-2-glycoprotein I)(Beta(2)GPI)(B2GPI)(Apolipoprotein H)(Apo-H)(Activated protein C-binding protein)(APC inhibitor)(Anticardiolipin cofactor) [Source: UniProt P02749]
3558.1.1	-9.1	4.47	3555.7321	-0.0026	3	48.28	dsak	124	CTEEGKWSPELPVCAPI	154	vykp	C [124] 57.0215, C [137] 57.0215, C [142	ENSP00000205948	Beta-2-glycoprotein 1 Precursor (Beta-2-glycoprotein I)(Beta(2)GPI)(B2GPI)(Apolipoprotein H)(Apo-H)(Activated protein C-binding protein)(APC inhibitor)(Anticardiolipin cofactor) [Source: UniProt P02749]
3560.1.1	-8.8	4.6	3555.7321	0.0047	3	48.28	dsak	124	CTEEGKWSPELPVCAPI	154	vykp	C [124] 57.0215, C [137] 57.0215, C [142	ENSP00000205948	Beta-2-glycoprotein 1 Precursor (Beta-2-glycoprotein I)(Beta(2)GPI)(B2GPI)(Apolipoprotein H)(Apo-H)(Activated protein C-binding protein)(APC inhibitor)(Anticardiolipin cofactor) [Source: UniProt P02749]
3565.1.1	-3	3.97	1914.0041	-0.0027	3	36.7	iagr	22	TCPKPDDLPFSTVVPLK	38	tfye	C [23] 57.0215	ENSP00000205948	Beta-2-glycoprotein 1 Precursor (Beta-2-glycoprotein I)(Beta(2)GPI)(B2GPI)(Apolipoprotein H)(Apo-H)(Activated protein C-binding protein)(APC inhibitor)(Anticardiolipin cofactor) [Source: UniProt P02749]
3569.1.1	-1.4	4.67	3527.701	0.03	3	48.28	dsak	124	CTEEGKWSPELPVCAPI	154	vykp	C [124] 57.0215, C [137] 57.0215, C [142	ENSP00000205948	Beta-2-glycoprotein 1 Precursor (Beta-2-glycoprotein I)(Beta(2)GPI)(B2GPI)(Apolipoprotein H)(Apo-H)(Activated protein C-binding protein)(APC inhibitor)(Anticardiolipin cofactor) [Source: UniProt P02749]
3575.1.1	-2.1	4.7	3527.701	0.03	3	48.28	dsak	124	CTEEGKWSPELPVCAPI	154	vykp	C [124] 57.0215, C [137] 57.0215, C [142	ENSP00000205948	Beta-2-glycoprotein 1 Precursor (Beta-2-glycoprotein I)(Beta(2)GPI)(B2GPI)(Apolipoprotein H)(Apo-H)(Activated protein C-binding protein)(APC inhibitor)(Anticardiolipin cofactor) [Source: UniProt P02749]
3580.1.1	-2.8	4.42	1901.0717	0.0008	3	47.69	ggmr	63	KFICPLTGLWPINTLK	78	ctpr	C [66] 57.0215	ENSP00000205948	Beta-2-glycoprotein 1 Precursor (Beta-2-glycoprotein I)(Beta(2)GPI)(B2GPI)(Apolipoprotein H)(Apo-H)(Activated protein C-binding protein)(APC inhibitor)(Anticardiolipin cofactor) [Source: UniProt P02749]
3582.1.1	-3	4.4	1901.0717	0.0025	3	47.69	ggmr	63	KFICPLTGLWPINTLK	78	ctpr	C [66] 57.0215	ENSP00000205948	Beta-2-glycoprotein 1 Precursor (Beta-2-glycoprotein I)(Beta(2)GPI)(B2GPI)(Apolipoprotein H)(Apo-H)(Activated protein C-binding protein)(APC inhibitor)(Anticardiolipin cofactor) [Source: UniProt P02749]
3584.1.1	-3	3.91	1788.9717	-0.0014	2	46.54	gmrk	64	FICPLTGLWPINTLK	78	ctpr	C [66] 57.0215, W [72] 15.9949	ENSP00000205948	Beta-2-glycoprotein 1 Precursor (Beta-2-glycoprotein I)(Beta(2)GPI)(B2GPI)(Apolipoprotein H)(Apo-H)(Activated protein C-binding protein)(APC inhibitor)(Anticardiolipin cofactor) [Source: UniProt P02749]
3589.1.1	-6.5	4.05	1788.9717	0.0017	2	46.54	gmrk	64	FICPLTGLWPINTLK	78	ctpr	C [66] 57.0215, W [72] 15.9949	ENSP00000205948	Beta-2-glycoprotein 1 Precursor (Beta-2-glycoprotein I)(Beta(2)GPI)(B2GPI)(Apolipoprotein H)(Apo-H)(Activated protein C-binding protein)(APC inhibitor)(Anticardiolipin cofactor) [Source: UniProt P02749]
3591.1.1	-4.2	3.7	1901.0717	0.003	2	47.69	ggmr	63	KFICPLTGLWPINTLK	78	ctpr	C [66] 57.0215	ENSP00000205948	Beta-2-glycoprotein 1 Precursor (Beta-2-glycoprotein I)(Beta(2)GPI)(B2GPI)(Apolipoprotein H)(Apo-H)(Activated protein C-binding protein)(APC inhibitor)(Anticardiolipin cofactor) [Source: UniProt P02749]
3593.1.1	-2.7	4.6	3539.7372	-0.0052	3	48.28	dsak	124	CTEEGKWSPELPVCAPI	154	vykp	C [124] 57.0215, C [137] 57.0215, C [142	ENSP00000205948	Beta-2-glycoprotein 1 Precursor (Beta-2-glycoprotein I)(Beta(2)GPI)(B2GPI)(Apolipoprotein H)(Apo-H)(Activated protein C-binding protein)(APC inhibitor)(Anticardiolipin cofactor) [Source: UniProt P02749]
3595.1.1	-5.4	3.84	2715.292	0.017	3	38.86	revk	205	CPFPSRPDNGFVNYPAK	227	dkat	C [205] 57.0215, N [213] 0.9848, C [205]	ENSP00000205948	Beta-2-glycoprotein 1 Precursor (Beta-2-glycoprotein I)(Beta(2)GPI)(B2GPI)(Apolipoprotein H)(Apo-H)(Activated protein C-binding protein)(APC inhibitor)(Anticardiolipin cofactor) [Source: UniProt P02749]
3596.1.1	-2.8	3.68	1901.0717	-0.0028	2	47.69	ggmr	63	KFICPLTGLWPINTLK	78	ctpr	C [66] 57.0215	ENSP00000205948	Beta-2-glycoprotein 1 Precursor (Beta-2-glycoprotein I)(Beta(2)GPI)(B2GPI)(Apolipoprotein H)(Apo-H)(Activated protein C-binding protein)(APC inhibitor)(Anticardiolipin cofactor) [Source: UniProt P02749]
3598.1.1	-3.2	4.4	3539.7372	-0.0071	3	48.28	dsak	124	CTEEGKWSPELPVCAPI	154	vykp	C [124] 57.0215, C [137] 57.0215, C [142	ENSP00000205948	Beta-2-glycoprotein 1 Precursor (Beta-2-glycoprotein I)(Beta(2)GPI)(B2GPI)(Apolipoprotein H)(Apo-H)(Activated protein C-binding protein)(APC inhibitor)(Anticardiolipin cofactor) [Source: UniProt P02749]
3600.1.1	-4.6	4.08	1788.9717	0.0019	3	46.54	gmrk	64	FICPLTGLWPINTLK	78	ctpr	C [66] 57.0215, W [72] 15.9949	ENSP00000205948	Beta-2-glycoprotein 1 Precursor (Beta-2-glycoprotein I)(Beta(2)GPI)(B2GPI)(Apolipoprotein H)(Apo-H)(Activated protein C-binding protein)(APC inhibitor)(Anticardiolipin cofactor) [Source: UniProt P02749]
3602.1.1	-2.6	4.68	3539.7372	0.0017	3	48.28	dsak	124	CTEEGKWSPELPVCAPI	154	vykp	C [124] 57.0215, C [137] 57.0215, C [142	ENSP00000205948	Beta-2-glycoprotein 1 Precursor (Beta-2-glycoprotein I)(Beta(2)GPI)(B2GPI)(Apolipoprotein H)(Apo-H)(Activated protein C-binding protein)(APC inhibitor)(Anticardiolipin cofactor) [Source: UniProt P02749]
3603.1.1	-5.1	4.15	1788.9717	0.0015	3	46.54	gmrk	64	FICPLTGLWPINTLK	78	ctpr	C [66] 57.0215, W [72] 15.9949	ENSP00000205948	Beta-2-glycoprotein 1 Precursor (Beta-2-glycoprotein I)(Beta(2)GPI)(B2GPI)(Apolipoprotein H)(Apo-H)(Activated protein C-binding protein)(APC inhibitor)(Anticardiolipin cofactor) [Source: UniProt P02749]
3604.1.1	-2.7	4.12	1958.107	-0.0072	3	43.63	tcat	2829	AHIGGVNIVLLEGITPNI	2847	ledf		ENSP00000264501	No description
3605.1.1	-6.1	3.9	1901.0717	0.0019	2	47.69	ggmr	63	KFICPLTGLWPINTLK	78	ctpr	C [66] 57.0215	ENSP00000205948	Beta-2-glycoprotein 1 Precursor (Beta-2-glycoprotein I)(Beta(2)GPI)(B2GPI)(Apolipoprotein H)(Apo-H)(Activated protein C-binding protein)(APC inhibitor)(Anticardiolipin cofactor) [Source: UniProt P02749]
3607.1.1	-2.3	4.1	1958.107	-0.017	3	43.63	tcat	2829	AHIGGVNIVLLEGITPNI	2847	ledf		ENSP00000264501	No description
3609.1.1	-2.9	4.23	1788.9717	0.0009	3	46.54	gmrk	64	FICPLTGLWPINTLK	78	ctpr	C [66] 57.0215, W [72] 15.9949	ENSP00000205948	Beta-2-glycoprotein 1 Precursor (Beta-2-glycoprotein I)(Beta(2)GPI)(B2GPI)(Apolipoprotein H)(Apo-H)(Activated protein C-binding protein)(APC inhibitor)(Anticardiolipin cofactor) [Source: UniProt P02749]
3622.1.1	-7.7	4.39	2633.3828	0.0002	3	47.72	egkw	131	SPELPVCAPIICPPPSIPTI	154	vykp	C [137] 57.0215, C [142] 57.0215	ENSP00000205948	Beta-2-glycoprotein 1 Precursor (Beta-2-glycoprotein I)(Beta(2)GPI)(B2GPI)(Apolipoprotein H)(Apo-H)(Activated protein C-binding protein)(APC inhibitor)(Anticardiolipin cofactor) [Source: UniProt P02749]
3625.1.1	-7	4.56	2633.3828	-0.0034	3	47.72	egkw	131	SPELPVCAPIICPPPSIPTI	154	vykp	C [137] 57.0215, C [142] 57.0215	ENSP00000205948	Beta-2-glycoprotein 1 Precursor (Beta-2-glycoprotein I)(Beta(2)GPI)(B2GPI)(Apolipoprotein H)(Apo-H)(Activated protein C-binding protein)(APC inhibitor)(Anticardiolipin cofactor) [Source: UniProt P02749]
3627.1.1	-7.4	4.8	2633.3828	0.0079	3	47.72	egkw	131	SPELPVCAPIICPPPSIPTI	154	vykp	C [137] 57.0215, C [142] 57.0215	ENSP00000205948	Beta-2-glycoprotein 1 Precursor (Beta-2-glycoprotein I)(Beta(2)GPI)(B2GPI)(Apolipoprotein H)(Apo-H)(Activated protein C-binding protein)(APC inhibitor)(Anticardiolipin cofactor) [Source: UniProt P02749]
3628.1.1	-5.9	3.8	1503.7632	-0.0014	2	32.15	ctpr	83	VCPFAGILENGAVR	96	yttf	C [84] 57.0215, N [92] 0.9848	ENSP00000205948	Beta-2-glycoprotein 1 Precursor (Beta-2-glycoprotein I)(Beta(2)GPI)(B2GPI)(Apolipoprotein H)(Apo-H)(Activated protein C-binding protein)(APC inhibitor)(Anticardiolipin cofactor) [Source: UniProt P02749]
3630.1.1	-10.4	4.28	2633.383	0.011	2	47.72	egkw	131	SPELPVCAPIICPPPSIPTI	154	vykp	C [137] 57.0215, C [142] 57.0215	ENSP00000205948	Beta-2-glycoprotein 1 Precursor (Beta-2-glycoprotein I)(Beta(2)GPI)(B2GPI)(Apolipoprotein H)(Apo-H)(Activated protein C-binding protein)(APC inhibitor)(Anticardiolipin cofactor) [Source: UniProt P02749]
3632.1.1	-1.8	3.8	1816.98	-0.012	2	34.95	sher	119	GSFEIGVEQLAGSLGKPI	136	rryf		ENSP00000300005:reversed	
3635.1.1	-8.6	4.29	2633.3828	-0.0024	2	47.72	egkw	131	SPELPVCAPIICPPPSIPTI	154	vykp	C [137] 57.0215, C [142] 57.0215	ENSP00000205948	Beta-2-glycoprotein 1 Precursor (Beta-2-glycoprotein I)(Beta(2)GPI)(B2GPI)(Apolipoprotein H)(Apo-H)(Activated protein C-binding protein)(APC inhibitor)(Anticardiolipin cofactor) [Source: UniProt P02749]
3637.1.1	-10.4	4.46	2633.3828	0.0051	2	47.72	egkw	131	SPELPVCAPIICPPPSIPTI	154	vykp	C [137] 57.0215, C [142] 57.0215	ENSP00000205948	Beta-2-glycoprotein 1 Precursor (Beta-2-glycoprotein I)(Beta(2)GPI)(B2GPI)(Apolipoprotein H)(Apo-H)(Activated protein C-binding protein)(APC inhibitor)(Anticardiolipin cofactor) [Source: UniProt P02749]
3639.1.1	-3.2	3.99	1914.0041	-0.0036	3	36.7	iagr	22	TCPKPDDLPFSTVVPLK	38	tfye	C [23] 57.0215	ENSP00000205948	Beta-2-glycoprotein 1 Precursor (Beta-2-glycoprotein I)(Beta(2)GPI)(B2GPI)(Apolipoprotein H)(Apo-H)(Activated protein C-binding protein)(APC inhibitor)(Anticardiolipin cofactor) [Source: UniProt P02749]
3646.1.1	-9.3	5.46	3523.7422	-0.0013	3	48.28	dsak	124	CTEEGKWSPELPVCAPI	154	vykp	C [124] 57.0215, C [137] 57.0215, C [142	ENSP00000205948	Beta-2-glycoprotein 1 Precursor (Beta-2-glycoprotein I)(Beta(2)GPI)(B2GPI)(Apolipoprotein H)(Apo-H)(Activated protein C-binding protein)(APC inhibitor)(Anticardiolipin cofactor) [Source: UniProt P02749]
3647.1.1	-2.7	4.78	3523.7422	0.0038	4	48.28	dsak	124	CTEEGKWSPELPVCAPI	154	vykp	C [124] 57.0215, C [137] 57.0215, C [142	ENSP00000205948	Beta-2-glycoprotein 1 Precursor (Beta-2-glycoprotein I)(Beta(2)GPI)(B2GPI)(Apolipoprotein H)(Apo-H)(Activated protein C-binding protein)(APC inhibitor)(Anticardiolipin cofactor) [Source: UniProt P02749]
3651.1.1	-9.4	5.32	3523.7422	0.0005	3	48.28	dsak	124	CTEEGKWSPELPVCAPI	154	vykp	C [124] 57.0215, C [137] 57.0215, C [142	ENSP00000205948	Beta-2-glycoprotein 1 Precursor (Beta-2-glycoprotein I)(Beta(2)GPI)(B2GPI)(Apolipoprotein H)(Apo-H)(Activated protein C-binding protein)(APC inhibitor)(Anticardiolipin cofactor) [Source: UniProt P02749]
3652.1.1	-4.5	5.23	1901.0717	0.0004	3	47.69	ggmr	63	KFICPLTGLWPINTLK	78	ctpr	C [66] 57.0215	ENSP00000205948	Beta-2-glycoprotein 1 Precursor (Beta-2-glycoprotein I)(Beta(2)GPI)(B2GPI)(Apolipoprotein H)(Apo-H)(Activated protein C-binding protein)(APC inhibitor)(Anticardiolipin cofactor) [Source: UniProt P02749]
3658.1.1	-7.2	5.23	3523.7422	-0.0002	3	48.28	dsak	124	CTEEGKWSPELPVCAPI	154	vykp	C [124] 57.0215, C [137] 57.0215, C [142	ENSP00000205948	Beta-2-glycoprotein 1 Precursor (Beta-2-glycoprotein I)(Beta(2)GPI)(B2GPI)(Apolipoprotein H)(Apo-H)(Activated protein C-binding protein)(APC inhibitor)(Anticardiolipin cofactor) [Source: UniProt P02749]
3659.1.1	-6.9	5.11	1901.0717	0.0015	3	47.69	ggmr	63	KFICPLTGLWPINTLK	78	ctpr	C [66] 57.0215	ENSP00000205948	Beta-2-glycoprotein 1 Precursor (Beta-2-glycoprotein I)(Beta(2)GPI)(B2GPI)(Apolipoprotein H)(Apo-H)(Activated protein C-binding protein)(APC inhibitor)(Anticardiolipin cofactor) [Source: UniProt P02749]
3660.1.1	-5.7	4.49	1788.9717	0.0016	2	46.54	gmrk	64	FICPLTGLWPINTLK	78	ctpr	C [66] 57.0215, W [72] 15.9949	ENSP00000205948	Beta-2-glycoprotein 1 Precursor (Beta-2-glycoprotein I)(Beta(2)GPI)(B2GPI)(Apolipoprotein H)(Apo-H)(Activated protein C-binding protein)(APC inhibitor)(Anticardiolipin cofactor) [Source: UniProt P02749]
3661.1.1	-5	4.58	3523.7422	0.0069	4	48.28	dsak	124	CTEEGKWSPELPVCAPI	154	vykp	C [124] 57.0215, C [137] 57.0215, C [142	ENSP00000205948	Beta-2-glycoprotein 1 Precursor (Beta-2-glycoprotein I)(Beta(2)GPI)(B2GPI)(Apolipoprotein H)(Apo-H)(Activated protein C-binding protein)(APC inhibitor)(Anticardiolipin cofactor) [Source: UniProt P02749]
3662.1.1	-7.2	4.06	1384.73	0.0013	2	39.55	nllr	38	FFVAPFPEVFGK	49	ekvn		sp|CAS1_BOVIN|	Alpha-S1-casein; Contains: Antioxidant peptide; Flags: Precursor;
3665.1.1	-6.4	5.02	1901.0717	0.0025	3	47.69	ggmr	63	KFICPLTGLWPINTLK	78	ctpr	C [66] 57.0215	ENSP00000205948	Beta-2-glycoprotein 1 Precursor (Beta-2-glycoprotein I)(Beta(2)GPI)(B2GPI)(Apolipoprotein H)(Apo-H)(Activated protein C-binding protein)(APC inhibitor)(Anticardiolipin cofactor) [Source: UniProt P02749]
3666.1.1	-6.5	4.42	1788.9717	0.0043	2	46.54	gmrk	64	FICPLTGLWPINTLK	78	ctpr	C [66] 57.0215, W [72] 15.9949	ENSP00000205948	Beta-2-glycoprotein 1 Precursor (Beta-2-glycoprotein I)(Beta(2)GPI)(B2GPI)(Apolipoprotein H)(Apo-H)(Activated protein C-binding protein)(APC inhibitor)(Anticardiolipin cofactor) [Source: UniProt P02749]
3667.1.1	-3.9	4.4	3523.7422	0.0052	4	48.28	dsak	124	CTEEGKWSPELPVCAPI	154	vykp	C [124] 57.0215, C [137] 57.0215, C [142	ENSP00000205948	Beta-2-glycoprotein 1 Precursor (Beta-2-glycoprotein I)(Beta(2)GPI)(B2GPI)(Apolipoprotein H)(Apo-H)(Activated protein C-binding protein)(APC inhibitor)(Anticardiolipin cofactor) [Source: UniProt P02749]
3668.1.1	-5	4.12	1384.73	0.0022	2	39.55	nllr	38	FFVAPFPEVFGK	49	ekvn		sp|CAS1_BOVIN|	Alpha-S1-casein; Contains: Antioxidant peptide; Flags: Precursor;
3671.1.1	-7	4.38	1788.9717	0.0017	2	46.54	gmrk	64	FICPLTGLWPINTLK	78	ctpr	C [66] 57.0215, W [72] 15.9949	ENSP00000205948	Beta-2-glycoprotein 1 Precursor (Beta-2-glycoprotein I)(Beta(2)GPI)(B2GPI)(Apolipoprotein H)(Apo-H)(Activated protein C-binding protein)(APC inhibitor)(Anticardiolipin cofactor) [Source: UniProt P02749]
3672.1.1	-6.2	4.09	1384.73	-0.0007	2	39.55	nllr	38	FFVAPFPEVFGK	49	ekvn		sp|CAS1_BOVIN|	Alpha-S1-casein; Contains: Antioxidant peptide; Flags: Precursor;
3675.1.1	-3.3	3.97	1901.0717	0.0045	2	47.69	ggmr	63	KFICPLTGLWPINTLK	78	ctpr	C [66] 57.0215	ENSP00000205948	Beta-2-glycoprotein 1 Precursor (Beta-2-glycoprotein I)(Beta(2)GPI)(B2GPI)(Apolipoprotein H)(Apo-H)(Activated protein C-binding protein)(APC inhibitor)(Anticardiolipin cofactor) [Source: UniProt P02749]
3677.1.1	-2.1	3.99	2383.0911	-0.0031	3	33.69	vplk	39	TFYEPGEEITYSCKPGYV	58	ggmr	C [51] 57.0215	ENSP00000205948	Beta-2-glycoprotein 1 Precursor (Beta-2-glycoprotein I)(Beta(2)GPI)(B2GPI)(Apolipoprotein H)(Apo-H)(Activated protein C-binding protein)(APC inhibitor)(Anticardiolipin cofactor) [Source: UniProt P02749]
3679.1.1	-2.7	4.05	1788.9717	0.0013	3	46.54	gmrk	64	FICPLTGLWPINTLK	78	ctpr	C [66] 57.0215, W [72] 15.9949	ENSP00000205948	Beta-2-glycoprotein 1 Precursor (Beta-2-glycoprotein I)(Beta(2)GPI)(B2GPI)(Apolipoprotein H)(Apo-H)(Activated protein C-binding protein)(APC inhibitor)(Anticardiolipin cofactor) [Source: UniProt P02749]
3684.1.1	-3.1	3.86	1914.0041	-0.0078	3	36.7	iagr	22	TCPKPDDLPFSTVVPLK	38	tfye	C [23] 57.0215	ENSP00000205948	Beta-2-glycoprotein 1 Precursor (Beta-2-glycoprotein I)(Beta(2)GPI)(B2GPI)(Apolipoprotein H)(Apo-H)(Activated protein C-binding protein)(APC inhibitor)(Anticardiolipin cofactor) [Source: UniProt P02749]
3689.1.1	-3.4	3.91	1914.0041	-0.0067	3	36.7	iagr	22	TCPKPDDLPFSTVVPLK	38	tfye	C [23] 57.0215	ENSP00000205948	Beta-2-glycoprotein 1 Precursor (Beta-2-glycoprotein I)(Beta(2)GPI)(B2GPI)(Apolipoprotein H)(Apo-H)(Activated protein C-binding protein)(APC inhibitor)(Anticardiolipin cofactor) [Source: UniProt P02749]
3691.1.1	-7.4	4.61	3538.7055	0.0054	3	48.28	dsak	124	CTEEGKWSPELPVCAPI	154	vykp	C [124] 57.0215, C [137] 57.0215, C [142	ENSP00000205948	Beta-2-glycoprotein 1 Precursor (Beta-2-glycoprotein I)(Beta(2)GPI)(B2GPI)(Apolipoprotein H)(Apo-H)(Activated protein C-binding protein)(APC inhibitor)(Anticardiolipin cofactor) [Source: UniProt P02749]
3694.1.1	-8.1	4.68	3538.7055	0.0076	3	48.28	dsak	124	CTEEGKWSPELPVCAPI	154	vykp	C [124] 57.0215, C [137] 57.0215, C [142	ENSP00000205948	Beta-2-glycoprotein 1 Precursor (Beta-2-glycoprotein I)(Beta(2)GPI)(B2GPI)(Apolipoprotein H)(Apo-H)(Activated protein C-binding protein)(APC inhibitor)(Anticardiolipin cofactor) [Source: UniProt P02749]
3695.1.1	-2.9	4	3555.7321	-0.0081	3	48.28	dsak	124	CTEEGKWSPELPVCAPI	154	vykp	C [124] 57.0215, C [137] 57.0215, C [142	ENSP00000205948	Beta-2-glycoprotein 1 Precursor (Beta-2-glycoprotein I)(Beta(2)GPI)(B2GPI)(Apolipoprotein H)(Apo-H)(Activated protein C-binding protein)(APC inhibitor)(Anticardiolipin cofactor) [Source: UniProt P02749]
3697.1.1	-5.8	3.93	1914.0041	-0.0041	3	36.7	iagr	22	TCPKPDDLPFSTVVPLK	38	tfye	C [23] 57.0215	ENSP00000205948	Beta-2-glycoprotein 1 Precursor (Beta-2-glycoprotein I)(Beta(2)GPI)(B2GPI)(Apolipoprotein H)(Apo-H)(Activated protein C-binding protein)(APC inhibitor)(Anticardiolipin cofactor) [Source: UniProt P02749]
3699.1.1	-5.4	4.64	3538.7055	-0.0038	3	48.28	dsak	124	CTEEGKWSPELPVCAPI	154	vykp	C [124] 57.0215, C [137] 57.0215, C [142	ENSP00000205948	Beta-2-glycoprotein 1 Precursor (Beta-2-glycoprotein I)(Beta(2)GPI)(B2GPI)(Apolipoprotein H)(Apo-H)(Activated protein C-binding protein)(APC inhibitor)(Anticardiolipin cofactor) [Source: UniProt P02749]
3708.1.1	-3.3	3.73	1503.7632	0.0044	2	32.15	ctpr	83	VCPFAGILENGAVR	96	yttf	C [84] 57.0215, N [92] 0.9848	ENSP00000205948	Beta-2-glycoprotein 1 Precursor (Beta-2-glycoprotein I)(Beta(2)GPI)(B2GPI)(Apolipoprotein H)(Apo-H)(Activated protein C-binding protein)(APC inhibitor)(Anticardiolipin cofactor) [Source: UniProt P02749]
3711.1.1	-1.5	3.89	2833.47	0.976	3	30.27	tlsk	49	LQVNGELPRPNYRPSTF	73	stsa		ENSP00000382139:reversed	
3714.1.1	-5	4.01	3555.7321	-0.0015	3	48.28	dsak	124	CTEEGKWSPELPVCAPI	154	vykp	C [124] 57.0215, C [137] 57.0215, C [142	ENSP00000205948	Beta-2-glycoprotein 1 Precursor (Beta-2-glycoprotein I)(Beta(2)GPI)(B2GPI)(Apolipoprotein H)(Apo-H)(Activated protein C-binding protein)(APC inhibitor)(Anticardiolipin cofactor) [Source: UniProt P02749]
3717.1.1	-6.4	4.23	2851.4521	-0.0036	3	53.03	eegk	130	WSPELPVCAPIICPPPSII	154	vykp	C [137] 57.0215, C [142] 57.0215, W [13	ENSP00000205948	Beta-2-glycoprotein 1 Precursor (Beta-2-glycoprotein I)(Beta(2)GPI)(B2GPI)(Apolipoprotein H)(Apo-H)(Activated protein C-binding protein)(APC inhibitor)(Anticardiolipin cofactor) [Source: UniProt P02749]
3719.1.1	-5.5	3.8	2633.3828	0.009	3	47.72	egkw	131	SPELPVCAPIICPPPSIPTI	154	vykp	C [137] 57.0215, C [142] 57.0215	ENSP00000205948	Beta-2-glycoprotein 1 Precursor (Beta-2-glycoprotein I)(Beta(2)GPI)(B2GPI)(Apolipoprotein H)(Apo-H)(Activated protein C-binding protein)(APC inhibitor)(Anticardiolipin cofactor) [Source: UniProt P02749]
3721.1.1	-5.5	4.39	2851.4521	0.0043	3	53.03	eegk	130	WSPELPVCAPIICPPPSII	154	vykp	C [137] 57.0215, C [142] 57.0215, W [13	ENSP00000205948	Beta-2-glycoprotein 1 Precursor (Beta-2-glycoprotein I)(Beta(2)GPI)(B2GPI)(Apolipoprotein H)(Apo-H)(Activated protein C-binding protein)(APC inhibitor)(Anticardiolipin cofactor) [Source: UniProt P02749]
3726.1.1	-6.8	4.43	2851.4521	0.0034	3	53.03	eegk	130	WSPELPVCAPIICPPPSII	154	vykp	C [137] 57.0215, C [142] 57.0215, W [13	ENSP00000205948	Beta-2-glycoprotein 1 Precursor (Beta-2-glycoprotein I)(Beta(2)GPI)(B2GPI)(Apolipoprotein H)(Apo-H)(Activated protein C-binding protein)(APC inhibitor)(Anticardiolipin cofactor) [Source: UniProt P02749]
3728.1.1	-4	3.85	2633.3828	-0.0047	3	47.72	egkw	131	SPELPVCAPIICPPPSIPTI	154	vykp	C [137] 57.0215, C [142] 57.0215	ENSP00000205948	Beta-2-glycoprotein 1 Precursor (Beta-2-glycoprotein I)(Beta(2)GPI)(B2GPI)(Apolipoprotein H)(Apo-H)(Activated protein C-binding protein)(APC inhibitor)(Anticardiolipin cofactor) [Source: UniProt P02749]
3729.1.1	-2.9	3.93	1788.9717	0.0017	3	46.54	gmrk	64	FICPLTGLWPINTLK	78	ctpr	C [66] 57.0215, W [72] 15.9949	ENSP00000205948	Beta-2-glycoprotein 1 Precursor (Beta-2-glycoprotein I)(Beta(2)GPI)(B2GPI)(Apolipoprotein H)(Apo-H)(Activated protein C-binding protein)(APC inhibitor)(Anticardiolipin cofactor) [Source: UniProt P02749]

3733.1.1	-1.5	3.72	3538.7055	0.0039	4	48.28	dsak	124	CTEEGKWSPELPVCAPI	154	vykp	C [124] 57.0215, C [137] 57.0215, C [142	ENSP00000205948	Beta-2-glycoprotein 1 Precursor (Beta-2-glycoprotein I)(Beta(2)GPI)(B2GPI)(Apolipoprotein H)(Apo-H)(Activated protein C-binding protein)(APC inhibitor)(Anticardiolipin cofactor) [Source: UniProt P02749]
3735.1.1	-3.5	4.45	1901.0717	-0.0021	3	47.69	ggmr	63	KFICPLTGLWPINTLK	78	ctpr	C [66] 57.0215	ENSP00000205948	Beta-2-glycoprotein 1 Precursor (Beta-2-glycoprotein I)(Beta(2)GPI)(B2GPI)(Apolipoprotein H)(Apo-H)(Activated protein C-binding protein)(APC inhibitor)(Anticardiolipin cofactor) [Source: UniProt P02749]
3741.1.1	-4.3	4.21	1788.9717	-0.0007	2	46.54	gmrk	64	FICPLTGLWPINTLK	78	ctpr	C [66] 57.0215, W [72] 15.9949	ENSP00000205948	Beta-2-glycoprotein 1 Precursor (Beta-2-glycoprotein I)(Beta(2)GPI)(B2GPI)(Apolipoprotein H)(Apo-H)(Activated protein C-binding protein)(APC inhibitor)(Anticardiolipin cofactor) [Source: UniProt P02749]
3742.1.1	-5.7	4.34	1901.0717	-0.0001	3	47.69	ggmr	63	KFICPLTGLWPINTLK	78	ctpr	C [66] 57.0215	ENSP00000205948	Beta-2-glycoprotein 1 Precursor (Beta-2-glycoprotein I)(Beta(2)GPI)(B2GPI)(Apolipoprotein H)(Apo-H)(Activated protein C-binding protein)(APC inhibitor)(Anticardiolipin cofactor) [Source: UniProt P02749]
3745.1.1	-5.6	4.24	1901.0717	0.0012	3	47.69	ggmr	63	KFICPLTGLWPINTLK	78	ctpr	C [66] 57.0215	ENSP00000205948	Beta-2-glycoprotein 1 Precursor (Beta-2-glycoprotein I)(Beta(2)GPI)(B2GPI)(Apolipoprotein H)(Apo-H)(Activated protein C-binding protein)(APC inhibitor)(Anticardiolipin cofactor) [Source: UniProt P02749]
3746.1.1	-5.6	4.26	1788.9717	0.0031	2	46.54	gmrk	64	FICPLTGLWPINTLK	78	ctpr	C [66] 57.0215, W [72] 15.9949	ENSP00000205948	Beta-2-glycoprotein 1 Precursor (Beta-2-glycoprotein I)(Beta(2)GPI)(B2GPI)(Apolipoprotein H)(Apo-H)(Activated protein C-binding protein)(APC inhibitor)(Anticardiolipin cofactor) [Source: UniProt P02749]
3747.1.1	-2.8	3.77	1502.778	0.991	2	32.15	ctpr	83	VCPFAGILENGAVR	96	yttf	C [84] 57.0215	ENSP00000205948	Beta-2-glycoprotein 1 Precursor (Beta-2-glycoprotein I)(Beta(2)GPI)(B2GPI)(Apolipoprotein H)(Apo-H)(Activated protein C-binding protein)(APC inhibitor)(Anticardiolipin cofactor) [Source: UniProt P02749]
3750.1.1	-6	4.11	1788.9717	-0.0015	2	46.54	gmrk	64	FICPLTGLWPINTLK	78	ctpr	C [66] 57.0215, W [72] 15.9949	ENSP00000205948	Beta-2-glycoprotein 1 Precursor (Beta-2-glycoprotein I)(Beta(2)GPI)(B2GPI)(Apolipoprotein H)(Apo-H)(Activated protein C-binding protein)(APC inhibitor)(Anticardiolipin cofactor) [Source: UniProt P02749]
3752.1.1	-2.7	3.83	1804.9666	0.0056	2	46.54	gmrk	64	FICPLTGLWPINTLK	78	ctpr	C [66] 57.0215, W [72] 31.9898	ENSP00000205948	Beta-2-glycoprotein 1 Precursor (Beta-2-glycoprotein I)(Beta(2)GPI)(B2GPI)(Apolipoprotein H)(Apo-H)(Activated protein C-binding protein)(APC inhibitor)(Anticardiolipin cofactor) [Source: UniProt P02749]
3755.1.1	-2.9	4	1804.9666	0.0051	2	46.54	gmrk	64	FICPLTGLWPINTLK	78	ctpr	C [66] 57.0215, W [72] 31.9898	ENSP00000205948	Beta-2-glycoprotein 1 Precursor (Beta-2-glycoprotein I)(Beta(2)GPI)(B2GPI)(Apolipoprotein H)(Apo-H)(Activated protein C-binding protein)(APC inhibitor)(Anticardiolipin cofactor) [Source: UniProt P02749]
3757.1.1	-5.9	4.26	1804.9666	0.0013	2	46.54	gmrk	64	FICPLTGLWPINTLK	78	ctpr	C [66] 57.0215, W [72] 31.9898	ENSP00000205948	Beta-2-glycoprotein 1 Precursor (Beta-2-glycoprotein I)(Beta(2)GPI)(B2GPI)(Apolipoprotein H)(Apo-H)(Activated protein C-binding protein)(APC inhibitor)(Anticardiolipin cofactor) [Source: UniProt P02749]
3759.1.1	-3.2	3.62	1503.7632	0.0044	2	32.15	ctpr	83	VCPFAGILENGAVR	96	yttf	C [84] 57.0215, N [92] 0.9848	ENSP00000205948	Beta-2-glycoprotein 1 Precursor (Beta-2-glycoprotein I)(Beta(2)GPI)(B2GPI)(Apolipoprotein H)(Apo-H)(Activated protein C-binding protein)(APC inhibitor)(Anticardiolipin cofactor) [Source: UniProt P02749]
3766.1.1	-2.5	3.63	1502.7784	-0.0033	2	32.15	ctpr	83	VCPFAGILENGAVR	96	yttf	C [84] 57.0215	ENSP00000205948	Beta-2-glycoprotein 1 Precursor (Beta-2-glycoprotein I)(Beta(2)GPI)(B2GPI)(Apolipoprotein H)(Apo-H)(Activated protein C-binding protein)(APC inhibitor)(Anticardiolipin cofactor) [Source: UniProt P02749]
3770.1.1	-3.2	3.91	1914.0041	0.0043	3	36.7	iagr	22	TCPKPDDLPFSTVVPLK	38	tfye	C [23] 57.0215	ENSP00000205948	Beta-2-glycoprotein 1 Precursor (Beta-2-glycoprotein I)(Beta(2)GPI)(B2GPI)(Apolipoprotein H)(Apo-H)(Activated protein C-binding protein)(APC inhibitor)(Anticardiolipin cofactor) [Source: UniProt P02749]
3774.1.1	-3.7	3.99	1788.9717	0.0008	3	46.54	gmrk	64	FICPLTGLWPINTLK	78	ctpr	C [66] 57.0215, W [72] 15.9949	ENSP00000205948	Beta-2-glycoprotein 1 Precursor (Beta-2-glycoprotein I)(Beta(2)GPI)(B2GPI)(Apolipoprotein H)(Apo-H)(Activated protein C-binding protein)(APC inhibitor)(Anticardiolipin cofactor) [Source: UniProt P02749]
3777.1.1	-2.4	4.43	2835.4572	0.0024	3	53.03	eegk	130	WSPELPVCAPIICPPPSI	154	vykp	C [137] 57.0215, C [142] 57.0215, W [13	ENSP00000205948	Beta-2-glycoprotein 1 Precursor (Beta-2-glycoprotein I)(Beta(2)GPI)(B2GPI)(Apolipoprotein H)(Apo-H)(Activated protein C-binding protein)(APC inhibitor)(Anticardiolipin cofactor) [Source: UniProt P02749]
3783.1.1	-3.6	4.45	2835.4572	0.0027	3	53.03	eegk	130	WSPELPVCAPIICPPPSI	154	vykp	C [137] 57.0215, C [142] 57.0215, W [13	ENSP00000205948	Beta-2-glycoprotein 1 Precursor (Beta-2-glycoprotein I)(Beta(2)GPI)(B2GPI)(Apolipoprotein H)(Apo-H)(Activated protein C-binding protein)(APC inhibitor)(Anticardiolipin cofactor) [Source: UniProt P02749]
3789.1.1	-1.2	4.3	3510.674	0.026	3	48.28	dsak	124	CTEEGKWSPELPVCAPI	154	vykp	C [124] 57.0215, C [137] 57.0215, C [142	ENSP00000205948	Beta-2-glycoprotein 1 Precursor (Beta-2-glycoprotein I)(Beta(2)GPI)(B2GPI)(Apolipoprotein H)(Apo-H)(Activated protein C-binding protein)(APC inhibitor)(Anticardiolipin cofactor) [Source: UniProt P02749]
3790.1.1	-3.9	3.98	2851.452	1.02	3	53.03	eegk	130	WSPELPVCAPIICPPPSI	154	vykp	C [137] 57.0215, C [142] 57.0215, W [13	ENSP00000205948	Beta-2-glycoprotein 1 Precursor (Beta-2-glycoprotein I)(Beta(2)GPI)(B2GPI)(Apolipoprotein H)(Apo-H)(Activated protein C-binding protein)(APC inhibitor)(Anticardiolipin cofactor) [Source: UniProt P02749]
3791.1.1	-2.4	3.87	1914.0041	-0.0019	3	36.7	iagr	22	TCPKPDDLPFSTVVPLK	38	tfye	C [23] 57.0215	ENSP00000205948	Beta-2-glycoprotein 1 Precursor (Beta-2-glycoprotein I)(Beta(2)GPI)(B2GPI)(Apolipoprotein H)(Apo-H)(Activated protein C-binding protein)(APC inhibitor)(Anticardiolipin cofactor) [Source: UniProt P02749]
3794.1.1	-3.3	3.79	1914.0041	0.0001	3	36.7	iagr	22	TCPKPDDLPFSTVVPLK	38	tfye	C [23] 57.0215	ENSP00000205948	Beta-2-glycoprotein 1 Precursor (Beta-2-glycoprotein I)(Beta(2)GPI)(B2GPI)(Apolipoprotein H)(Apo-H)(Activated protein C-binding protein)(APC inhibitor)(Anticardiolipin cofactor) [Source: UniProt P02749]
3801.1.1	-1.2	4.16	3510.674	0.021	4	48.28	dsak	124	CTEEGKWSPELPVCAPI	154	vykp	C [124] 57.0215, C [137] 57.0215, C [142	ENSP00000205948	Beta-2-glycoprotein 1 Precursor (Beta-2-glycoprotein I)(Beta(2)GPI)(B2GPI)(Apolipoprotein H)(Apo-H)(Activated protein C-binding protein)(APC inhibitor)(Anticardiolipin cofactor) [Source: UniProt P02749]
3806.1.1	-3.2	4.1	1788.9717	-0.0015	2	46.54	gmrk	64	FICPLTGLWPINTLK	78	ctpr	C [66] 57.0215, W [72] 15.9949	ENSP00000205948	Beta-2-glycoprotein 1 Precursor (Beta-2-glycoprotein I)(Beta(2)GPI)(B2GPI)(Apolipoprotein H)(Apo-H)(Activated protein C-binding protein)(APC inhibitor)(Anticardiolipin cofactor) [Source: UniProt P02749]
3807.1.1	-3.8	3.69	1772.9768	0.0056	2	46.54	gmrk	64	FICPLTGLWPINTLK	78	ctpr	C [66] 57.0215	ENSP00000205948	Beta-2-glycoprotein 1 Precursor (Beta-2-glycoprotein I)(Beta(2)GPI)(B2GPI)(Apolipoprotein H)(Apo-H)(Activated protein C-binding protein)(APC inhibitor)(Anticardiolipin cofactor) [Source: UniProt P02749]
3810.1.1	-5.3	4.08	1788.9717	-0.0017	2	46.54	gmrk	64	FICPLTGLWPINTLK	78	ctpr	C [66] 57.0215, W [72] 15.9949	ENSP00000205948	Beta-2-glycoprotein 1 Precursor (Beta-2-glycoprotein I)(Beta(2)GPI)(B2GPI)(Apolipoprotein H)(Apo-H)(Activated protein C-binding protein)(APC inhibitor)(Anticardiolipin cofactor) [Source: UniProt P02749]
3812.1.1	-3.7	3.85	1804.9666	0.0055	2	46.54	gmrk	64	FICPLTGLWPINTLK	78	ctpr	C [66] 57.0215, W [72] 31.9898	ENSP00000205948	Beta-2-glycoprotein 1 Precursor (Beta-2-glycoprotein I)(Beta(2)GPI)(B2GPI)(Apolipoprotein H)(Apo-H)(Activated protein C-binding protein)(APC inhibitor)(Anticardiolipin cofactor) [Source: UniProt P02749]
3813.1.1	-4.7	3.92	1772.9768	-0.0046	2	46.54	gmrk	64	FICPLTGLWPINTLK	78	ctpr	C [66] 57.0215	ENSP00000205948	Beta-2-glycoprotein 1 Precursor (Beta-2-glycoprotein I)(Beta(2)GPI)(B2GPI)(Apolipoprotein H)(Apo-H)(Activated protein C-binding protein)(APC inhibitor)(Anticardiolipin cofactor) [Source: UniProt P02749]
3816.1.1	-4.6	4.02	1788.9717	-0.0012	2	46.54	gmrk	64	FICPLTGLWPINTLK	78	ctpr	C [66] 57.0215, W [72] 15.9949	ENSP00000205948	Beta-2-glycoprotein 1 Precursor (Beta-2-glycoprotein I)(Beta(2)GPI)(B2GPI)(Apolipoprotein H)(Apo-H)(Activated protein C-binding protein)(APC inhibitor)(Anticardiolipin cofactor) [Source: UniProt P02749]
3817.1.1	-4.9	3.98	1772.9768	-0.0004	2	46.54	gmrk	64	FICPLTGLWPINTLK	78	ctpr	C [66] 57.0215	ENSP00000205948	Beta-2-glycoprotein 1 Precursor (Beta-2-glycoprotein I)(Beta(2)GPI)(B2GPI)(Apolipoprotein H)(Apo-H)(Activated protein C-binding protein)(APC inhibitor)(Anticardiolipin cofactor) [Source: UniProt P02749]
3818.1.1	-2.9	3.83	1804.9666	-0.0005	2	46.54	gmrk	64	FICPLTGLWPINTLK	78	ctpr	C [66] 57.0215, W [72] 31.9898	ENSP00000205948	Beta-2-glycoprotein 1 Precursor (Beta-2-glycoprotein I)(Beta(2)GPI)(B2GPI)(Apolipoprotein H)(Apo-H)(Activated protein C-binding protein)(APC inhibitor)(Anticardiolipin cofactor) [Source: UniProt P02749]
3820.1.1	-5	4.31	2819.4623	-0.0011	2	53.03	eegk	130	WSPELPVCAPIICPPPSI	154	vykp	C [137] 57.0215, C [142] 57.0215	ENSP00000205948	Beta-2-glycoprotein 1 Precursor (Beta-2-glycoprotein I)(Beta(2)GPI)(B2GPI)(Apolipoprotein H)(Apo-H)(Activated protein C-binding protein)(APC inhibitor)(Anticardiolipin cofactor) [Source: UniProt P02749]
3821.1.1	-1.9	4.01	1901.0717	-0.0036	3	47.69	ggmr	63	KFICPLTGLWPINTLK	78	ctpr	C [66] 57.0215	ENSP00000205948	Beta-2-glycoprotein 1 Precursor (Beta-2-glycoprotein I)(Beta(2)GPI)(B2GPI)(Apolipoprotein H)(Apo-H)(Activated protein C-binding protein)(APC inhibitor)(Anticardiolipin cofactor) [Source: UniProt P02749]
3823.1.1	-9.9	4.39	3523.7422	0.0002	3	48.28	dsak	124	CTEEGKWSPELPVCAPI	154	vykp	C [124] 57.0215, C [137] 57.0215, C [142	ENSP00000205948	Beta-2-glycoprotein 1 Precursor (Beta-2-glycoprotein I)(Beta(2)GPI)(B2GPI)(Apolipoprotein H)(Apo-H)(Activated protein C-binding protein)(APC inhibitor)(Anticardiolipin cofactor) [Source: UniProt P02749]
3824.1.1	-1.2	3.68	3523.7422	-0.0062	4	48.28	dsak	124	CTEEGKWSPELPVCAPI	154	vykp	C [124] 57.0215, C [137] 57.0215, C [142	ENSP00000205948	Beta-2-glycoprotein 1 Precursor (Beta-2-glycoprotein I)(Beta(2)GPI)(B2GPI)(Apolipoprotein H)(Apo-H)(Activated protein C-binding protein)(APC inhibitor)(Anticardiolipin cofactor) [Source: UniProt P02749]
3825.1.1	-2.2	3.99	1901.0717	0.0026	3	47.69	ggmr	63	KFICPLTGLWPINTLK	78	ctpr	C [66] 57.0215	ENSP00000205948	Beta-2-glycoprotein 1 Precursor (Beta-2-glycoprotein I)(Beta(2)GPI)(B2GPI)(Apolipoprotein H)(Apo-H)(Activated protein C-binding protein)(APC inhibitor)(Anticardiolipin cofactor) [Source: UniProt P02749]
3827.1.1	-8.4	4.51	3523.7422	-0.002	3	48.28	dsak	124	CTEEGKWSPELPVCAPI	154	vykp	C [124] 57.0215, C [137] 57.0215, C [142	ENSP00000205948	Beta-2-glycoprotein 1 Precursor (Beta-2-glycoprotein I)(Beta(2)GPI)(B2GPI)(Apolipoprotein H)(Apo-H)(Activated protein C-binding protein)(APC inhibitor)(Anticardiolipin cofactor) [Source: UniProt P02749]
3828.1.1	-7.8	4.77	2819.4623	0.0072	2	53.03	eegk	130	WSPELPVCAPIICPPPSI	154	vykp	C [137] 57.0215, C [142] 57.0215	ENSP00000205948	Beta-2-glycoprotein 1 Precursor (Beta-2-glycoprotein I)(Beta(2)GPI)(B2GPI)(Apolipoprotein H)(Apo-H)(Activated protein C-binding protein)(APC inhibitor)(Anticardiolipin cofactor) [Source: UniProt P02749]
3830.1.1	-1.7	3.95	1901.0717	0.0006	3	47.69	ggmr	63	KFICPLTGLWPINTLK	78	ctpr	C [66] 57.0215	ENSP00000205948	Beta-2-glycoprotein 1 Precursor (Beta-2-glycoprotein I)(Beta(2)GPI)(B2GPI)(Apolipoprotein H)(Apo-H)(Activated protein C-binding protein)(APC inhibitor)(Anticardiolipin cofactor) [Source: UniProt P02749]
3831.1.1	-2.4	3.59	1502.7784	0.0008	2	32.15	ctpr	83	VCPFAGILENGAVR	96	yttf	C [84] 57.0215	ENSP00000205948	Beta-2-glycoprotein 1 Precursor (Beta-2-glycoprotein I)(Beta(2)GPI)(B2GPI)(Apolipoprotein H)(Apo-H)(Activated protein C-binding protein)(APC inhibitor)(Anticardiolipin cofactor) [Source: UniProt P02749]
3833.1.1	-7.1	4.32	3523.7422	-0.002	3	48.28	dsak	124	CTEEGKWSPELPVCAPI	154	vykp	C [124] 57.0215, C [137] 57.0215, C [142	ENSP00000205948	Beta-2-glycoprotein 1 Precursor (Beta-2-glycoprotein I)(Beta(2)GPI)(B2GPI)(Apolipoprotein H)(Apo-H)(Activated protein C-binding protein)(APC inhibitor)(Anticardiolipin cofactor) [Source: UniProt P02749]
3834.1.1	-5.7	4.57	2819.4623	0.0026	2	53.03	eegk	130	WSPELPVCAPIICPPPSI	154	vykp	C [137] 57.0215, C [142] 57.0215	ENSP00000205948	Beta-2-glycoprotein 1 Precursor (Beta-2-glycoprotein I)(Beta(2)GPI)(B2GPI)(Apolipoprotein H)(Apo-H)(Activated protein C-binding protein)(APC inhibitor)(Anticardiolipin cofactor) [Source: UniProt P02749]
3837.1.1	-9.5	5.33	2819.4623	0.0001	3	53.03	eegk	130	WSPELPVCAPIICPPPSI	154	vykp	C [137] 57.0215, C [142] 57.0215	ENSP00000205948	Beta-2-glycoprotein 1 Precursor (Beta-2-glycoprotein I)(Beta(2)GPI)(B2GPI)(Apolipoprotein H)(Apo-H)(Activated protein C-binding protein)(APC inhibitor)(Anticardiolipin cofactor) [Source: UniProt P02749]
3838.1.1	-3.1	3.66	1503.7632	0.0087	2	32.15	ctpr	83	VCPFAGILENGAVR	96	yttf	C [84] 57.0215, N [92] 0.9848	ENSP00000205948	Beta-2-glycoprotein 1 Precursor (Beta-2-glycoprotein I)(Beta(2)GPI)(B2GPI)(Apolipoprotein H)(Apo-H)(Activated protein C-binding protein)(APC inhibitor)(Anticardiolipin cofactor) [Source: UniProt P02749]
3840.1.1	-9.2	5.27	2819.4623	0.001	3	53.03	eegk	130	WSPELPVCAPIICPPPSI	154	vykp	C [137] 57.0215, C [142] 57.0215	ENSP00000205948	Beta-2-glycoprotein 1 Precursor (Beta-2-glycoprotein I)(Beta(2)GPI)(B2GPI)(Apolipoprotein H)(Apo-H)(Activated protein C-binding protein)(APC inhibitor)(Anticardiolipin cofactor) [Source: UniProt P02749]
3843.1.1	-6.9	5.26	2819.4623	0.0043	3	53.03	eegk	130	WSPELPVCAPIICPPPSI	154	vykp	C [137] 57.0215, C [142] 57.0215	ENSP00000205948	Beta-2-glycoprotein 1 Precursor (Beta-2-glycoprotein I)(Beta(2)GPI)(B2GPI)(Apolipoprotein H)(Apo-H)(Activated protein C-binding protein)(APC inhibitor)(Anticardiolipin cofactor) [Source: UniProt P02749]
3848.1.1	-1.6	4.06	2834.502	1.988	3	9.56	nqkk	179	LPKPSSAPSHSHKPSAEP	205	yfhp		ENSP00000344488:reversed	
3856.1.1	-1.8	3.64	1804.967	-0.011	2	46.54	gmrk	64	FICPLTGLWPINTLK	78	ctpr	C [66] 57.0215, W [72] 31.9898	ENSP00000205948	Beta-2-glycoprotein 1 Precursor (Beta-2-glycoprotein I)(Beta(2)GPI)(B2GPI)(Apolipoprotein H)(Apo-H)(Activated protein C-binding protein)(APC inhibitor)(Anticardiolipin cofactor) [Source: UniProt P02749]
3860.1.1	-10.2	4.58	3506.7157	0.0082	3	48.28	dsak	124	CTEEGKWSPELPVCAPI	154	vykp	C [124] 57.0215, C [137] 57.0215, C [142	ENSP00000205948	Beta-2-glycoprotein 1 Precursor (Beta-2-glycoprotein I)(Beta(2)GPI)(B2GPI)(Apolipoprotein H)(Apo-H)(Activated protein C-binding protein)(APC inhibitor)(Anticardiolipin cofactor) [Source: UniProt P02749]
3862.1.1	-1.7	3.72	1914.0041	-0.0021	3	36.7	iagr	22	TCPKPDDLPFSTVVPLK	38	tfye	C [23] 57.0215	ENSP00000205948	Beta-2-glycoprotein 1 Precursor (Beta-2-glycoprotein I)(Beta(2)GPI)(B2GPI)(Apolipoprotein H)(Apo-H)(Activated protein C-binding protein)(APC inhibitor)(Anticardiolipin cofactor) [Source: UniProt P02749]
3865.1.1	-6.3	4.62	3506.7157	0.0093	3	48.28	dsak	124	CTEEGKWSPELPVCAPI	154	vykp	C [124] 57.0215, C [137] 57.0215, C [142	ENSP00000205948	Beta-2-glycoprotein 1 Precursor (Beta-2-glycoprotein I)(Beta(2)GPI)(B2GPI)(Apolipoprotein H)(Apo-H)(Activated protein C-binding protein)(APC inhibitor)(Anticardiolipin cofactor) [Source: UniProt P02749]
3868.1.1	-3.2	3.53	1804.9666	-0.0035	2	46.54	gmrk	64	FICPLTGLWPINTLK	78	ctpr	C [66] 57.0215, W [72] 31.9898	ENSP00000205948	Beta-2-glycoprotein 1 Precursor (Beta-2-glycoprotein I)(Beta(2)GPI)(B2GPI)(Apolipoprotein H)(Apo-H)(Activated protein C-binding protein)(APC inhibitor)(Anticardiolipin cofactor) [Source: UniProt P02749]
3871.1.1	-6.4	4.61	3506.7157	0.0034	3	48.28	dsak	124	CTEEGKWSPELPVCAPI	154	vykp	C [124] 57.0215, C [137] 57.0215, C [142	ENSP00000205948	Beta-2-glycoprotein 1 Precursor (Beta-2-glycoprotein I)(Beta(2)GPI)(B2GPI)(Apolipoprotein H)(Apo-H)(Activated protein C-binding protein)(APC inhibitor)(Anticardiolipin cofactor) [Source: UniProt P02749]
3874.1.1	-4.2	3.95	1788.9717	-0.0016	3	46.54	gmrk	64	FICPLTGLWPINTLK	78	ctpr	C [66] 57.0215, W [72] 15.9949	ENSP00000205948	Beta-2-glycoprotein 1 Precursor (Beta-2-glycoprotein I)(Beta(2)GPI)(B2GPI)(Apolipoprotein H)(Apo-H)(Activated protein C-binding protein)(APC inhibitor)(Anticardiolipin cofactor) [Source: UniProt P02749]
3875.1.1	-1.6	3.53	1804.9666	-0.0026	2	46.54	gmrk	64	FICPLTGLWPINTLK	78	ctpr	C [66] 57.0215, W [72] 31.9898	ENSP00000205948	Beta-2-glycoprotein 1 Precursor (Beta-2-glycoprotein I)(Beta(2)GPI)(B2GPI)(Apolipoprotein H)(Apo-H)(Activated protein C-binding protein)(APC inhibitor)(Anticardiolipin cofactor) [Source: UniProt P02749]
3876.1.1	-2.9	3.74	1914.0041	-0.0091	3	36.7	iagr	22	TCPKPDDLPFSTVVPLK	38	tfye	C [23] 57.0215	ENSP00000205948	Beta-2-glycoprotein 1 Precursor (Beta-2-glycoprotein I)(Beta(2)GPI)(B2GPI)(Apolipoprotein H)(Apo-H)(Activated protein C-binding protein)(APC inhibitor)(Anticardiolipin cofactor) [Source: UniProt P02749]
3878.1.1	-5.6	4.63	1772.9768	0.0029	3	46.54	gmrk	64	FICPLTGLWPINTLK	78	ctpr	C [66] 57.0215	ENSP00000205948	Beta-2-glycoprotein 1 Precursor (Beta-2-glycoprotein I)(Beta(2)GPI)(B2GPI)(Apolipoprotein H)(Apo-H)(Activated protein C-binding protein)(APC inhibitor)(Anticardiolipin cofactor) [Source: UniProt P02749]
3884.1.1	-7	4.78	1772.9768	0.0001	3	46.54	gmrk	64	FICPLTGLWPINTLK	78	ctpr	C [66] 57.0215	ENSP00000205948	Beta-2-glycoprotein 1 Precursor (Beta-2-glycoprotein I)(Beta(2)GPI)(B2GPI)(Apolipoprotein H)(Apo-H)(Activated protein C-binding protein)(APC inhibitor)(Anticardiolipin cofactor) [Source: UniProt P02749]
3886.1.1	-6.3	4.87	1772.9768	0.001	3	46.54	gmrk	64	FICPLTGLWPINTLK	78	ctpr	C [66] 57.0215	ENSP00000205948	Beta-2-glycoprotein 1 Precursor (Beta-2-glycoprotein I)(Beta(2)GPI)(B2GPI)(Apolipoprotein H)(Apo-H)(Activated protein C-binding protein)(APC inhibitor)(Anticardiolipin cofactor) [Source: UniProt P02749]
3888.1.1	-8	6.06	1772.9768	-0.0019	2	46.54	gmrk	64	FICPLTGLWPINTLK	78	ctpr	C [66] 57.0215	ENSP00000205948	Beta-2-glycoprotein 1 Precursor (Beta-2-glycoprotein I)(Beta(2)GPI)(B2GPI)(Apolipoprotein H)(Apo-H)(Activated protein C-binding protein)(APC inhibitor)(Anticardiolipin cofactor) [Source: UniProt P02749]
3889.1.1	-1.2	3.99	1573.782	-0.014	2	20.68	etar	235	AAEEALSSLGQSGTPR	250	lwar		ENSP00000377334:reversed	
3891.1.1	-7.5	6.2	1772.9768	0.0007	2	46.54	gmrk	64	FICPLTGLWPINTLK	78	ctpr	C [66] 57.0215	ENSP00000205948	Beta-2-glycoprotein 1 Precursor (Beta-2-glycoprotein I)(Beta(2)GPI)(B2GPI)(Apolipoprotein H)(Apo-H)(Activated protein C-binding protein)(APC inhibitor)(Anticardiolipin cofactor) [Source: UniProt P02749]
3892.1.1	-4.4	4.97	1788.9717	-0.0008	2	46.54	gmrk	64	FICPLTGLWPINTLK	78	ctpr	C [66] 57.0215, W [72] 15.9949	ENSP00000205948	Beta-2-glycoprotein 1 Precursor (Beta-2-glycoprotein I)(Beta(2)GPI)(B2GPI)(Apolipoprotein H)(Apo-H)(Activated protein C-binding protein)(APC inhibitor)(Anticardiolipin cofactor) [Source: UniProt P02749]
3895.1.1	-6.7	6.11	1772.9768	0.0014	2	46.54	gmrk	64	FICPLTGLWPINTLK	78	ctpr	C [66] 57.0215	ENSP00000205948	Beta-2-glycoprotein 1 Precursor (Beta-2-glycoprotein I)(Beta(2)GPI)(B2GPI)(Apolipoprotein H)(Apo-H)(Activated protein C-binding protein)(APC inhibitor)(Anticardiolipin cofactor) [Source: UniProt P02749]
3896.1.1	-4.5	4.86	1788.9717	0.0006	2	46.54	gmrk	64	FICPLTGLWPINTLK	78	ctpr	C [66] 57.0215, W [72] 15.9949	ENSP00000205948	Beta-2-glycoprotein 1 Precursor (Beta-2-glycoprotein I)(Beta(2)GPI)(B2GPI)(Apolipoprotein H)(Apo-H)(Activated protein C-binding protein)(APC inhibitor)(Anticardiolipin cofactor) [Source: UniProt P02749]
3934.1.1	-4.2	4.92	1772.9768	0.0014	3	46.54	gmrk	64	FICPLTGLWPINTLK	78	ctpr	C [66] 57.0215	ENSP00000205948	Beta-2-glycoprotein 1 Precursor (Beta-2-glycoprotein I)(Beta(2)GPI)(B2GPI)(Apolipoprotein H)(Apo-H)(Activated protein C-binding protein)(APC inhibitor)(Anticardiolipin cofactor) [Source: UniProt P02749]
3936.1.1	-7.2	5.05	1772.9768	-0.0004	3	46.54	gmrk	64	FICPLTGLWPINTLK	78	ctpr	C [66] 57.0215	ENSP00000205948	Beta-2-glycoprotein 1 Precursor (Beta-2-glycoprotein I)(Beta(2)GPI)(B2GPI)(Apolipoprotein H)(Apo-H)(Activated protein C-binding protein)(APC inhibitor)(Anticardiolipin cofactor) [Source: UniProt P02749]
3938.1.1	-2.9	5.04	1772.9768	0.002	3	46.54	gmrk	64	FICPLTGLWPINTLK	78	ctpr	C [66] 57.0215	ENSP00000205948	Beta-2-glycoprotein 1 Precursor (Beta-2-glycoprotein I)(Beta(2)GPI)(B2GPI)(Apolipoprotein H)(Apo-H)(Activated protein C-binding protein)(APC inhibitor)(Anticardiolipin cofactor) [Source: UniProt P02749]
3940.1.1	-6.3	5.97	1772.9768	-0.001	2	46.54	gmrk	64	FICPLTGLWPINTLK	78	ctpr	C [66] 57.0215	ENSP00000205948	Beta-2-glycoprotein 1 Precursor (Beta-2-glycoprotein I)(Beta(2)GPI)(B2GPI)(Apolipoprotein H)(Apo-H)(Activated protein C-binding protein)(APC inhibitor)(Anticardiolipin cofactor) [Source: UniProt P02749]
3943.1.1	-12	5.98	1772.9768	0.0009	2	46.54	gmrk	64	FICPLTGLWPINTLK	78	ctpr	C [66] 57.0215	ENSP00000205948	Beta-2-glycoprotein 1 Precursor (Beta-2-glycoprotein I)(Beta(2)GPI)(B2GPI)(Apolipoprotein H)(Apo-H)(Activated protein C-binding protein)(APC inhibitor)(Anticardiolipin cofactor) [Source: UniProt P02749]
3945.1.1	-1.9	3.85	1788.9717	0.0058	2	46.54	gmrk	64	FICPLTGLWPINTLK	78	ctpr	C [66] 57.0215, W [72] 15.9949	ENSP00000205948	Beta-2-glycoprotein 1 Precursor (Beta-2-glycoprotein I)(Beta(2)GPI)(B2GPI)(Apolipoprotein H)(Apo-H)(Activated protein C-binding protein)(APC inhibitor)(Anticardiolipin cofactor) [Source: UniProt P02749]
3947.1.1	-8.1	6.01	1772.9768	0.0011	2	46.54	gmrk	64	FICPLTGLWPINTLK	78	ctpr	C [66] 57.0215	ENSP00000205948	Beta-2-glycoprotein 1 Precursor (Beta-2-glycoprotein I)(Beta(2)GPI)(B2GPI)(Apolipoprotein H)(Apo-H)(Activated protein C-binding protein)(APC inhibitor)(Anticardiolipin cofactor) [Source: UniProt P02749]
3949.1.1	-1.6	4.12	1788.9717	0.0006	2	46.54	gmrk	64	FICPLTGLWPINTLK	78	ctpr	C [66] 57.0215, W [72] 15.9949	ENSP00000205948	Beta-2-glycoprotein 1 Precursor (Beta-2-glycoprotein I)(Beta(2)GPI)(B2GPI)(Apolipoprotein H)(Apo-H)(Activated protein C-binding protein)(APC inhibitor)(Anticardiolipin cofactor) [Source: UniProt P02749]
3951.1.1	-1.5	3.72	1914.0041	-0.0003	3	36.7	iagr	22	TCPKPDDLPFSTVVPLK	38	tfye	C [23] 57.0215	ENSP00000205948	Beta-2-glycoprotein 1 Precursor (Beta-2-glycoprotein I)(Beta(2)GPI)(B2GPI)(Apolipoprotein H)(Apo-H)(Activated protein C-binding protein)(APC inhibitor)(Anticardiolipin cofactor) [Source: UniProt P02749]
3954.1.1	-2.6	3.71	1788.9717	-0.0036	2	46.54	gmrk	64	FICPLTGLWPINTLK	78	ctpr	C [66] 57.0215, W [72] 15.9949	ENSP00000205948	Beta-2-glycoprotein 1 Precursor (Beta-2-glycoprotein I)(Beta(2)GPI)(B2GPI)(Apolipoprotein H)(Apo-H)(Activated protein C-binding protein)(APC inhibitor)(Anticardiolipin cofactor) [Source: UniProt P02749]
3975.1.1	-3.8	3.79	1914.0041	-0.0045	3	36.7	iagr	22	TCPKPDDLPFSTVVPLK	38	tfye	C [23] 57.0215	ENSP00000205948	Beta-2-glycoprotein 1 Precursor (Beta-2-glycoprotein I)(Beta(2)GPI)(B2GPI)(Apolipoprotein H)(Apo-H)(Activated protein C-binding protein)(APC inhibitor)(Anticardiolipin cofactor) [Source: UniProt P02749]
3993.1.1	-7.7	4.97	1772.9768	-0.0002	2	46.54	gmrk	64	FICPLTGLWPINTLK	78	ctpr	C [66] 57.0215	ENSP00000205948	Beta-2-glycoprotein 1 Precursor (Beta-2-glycoprotein I)(Beta(2)GPI)(B2GPI)(Apolipoprotein H)(Apo-H)(Activated protein C-binding protein)(APC inhibitor)(Anticardiolipin cofactor) [Source: UniProt P02749]
3996.1.1	-8.7	4.91	1772.9768	0.002	2	46.54	gmrk	64	FICPLTGLWPINTLK	78	ctpr	C [66] 57.0215	ENSP00000205948	Beta-2-glycoprotein 1 Precursor (Beta-2-glycoprotein I)(Beta(2)GPI)(B2GPI)(Apolipoprotein H)(Apo-H)(Activated protein C-binding protein)(APC inhibitor)(Anticardiolipin cofactor) [Source: UniProt P02749]
3998.1.1	-5.5	4.97	1772.9768	0.0006	2	46.54	gmrk	64	FICPLTGLWPINTLK	78	ctpr	C [66] 57.0215	ENSP00000205948	Beta-2-glycoprotein 1 Precursor (Beta-2-glycoprotein I)(Beta(2)GPI)(B2GPI)(Apolipoprotein H)(Apo-H)(Activated protein C-binding protein)(APC inhibitor)(Anticardiolipin cofactor) [Source: UniProt P02749]
4005.1.1	-2.2	3.78	1430.7501	0.002	2	41.82	gmrk	64	FICPLTGLWPIN	75	tlkc	C [66] 57.0215	ENSP00000205948	Beta-2-glycoprotein 1 Precursor (Beta-2-glycoprotein I)(Beta(2)GPI)(B2GPI)(Apolipoprotein H)(Apo-H)(Activated protein C-binding protein)(APC inhibitor)(Anticardiolipin cofactor) [Source: UniProt P02749]
4015.1.1	-2.8	3.61	2715.292	0.0098	3	38.86	revk	205	CPFFPSRPDNGFVNYPAK	227	dkat	C [205] 57.0215, N [213] 0.9848, C [205]	ENSP00000205948	Beta-2-glycoprotein 1 Precursor (Beta-2-glycoprotein I)(Beta(2)GPI)(B2GPI)(Apolipoprotein H)(Apo-H)(Activated protein C-binding protein)(APC inhibitor)(Anticardiolipin cofactor) [Source: UniProt P02749]
4033.1.1	-3.6	3.58	1914.0041	-0.0016	3	36.7	iagr	22	TCPKPDDLPFSTVVPLK	38	tfye	C [23] 57.0215	ENSP00000205948	Beta-2-glycoprotein 1 Precursor (Beta-2-glycoprotein I)(Beta(2)GPI)(B2GPI)(Apolipoprotein H)(Apo-H)(Activated protein C-binding protein)(APC inhibitor)(Anticardiolipin cofactor) [Source: UniProt P02749]
4038.1.1	-5.9	4.59	1772.9768	0.0004	2	46.54	gmrk	64	FICPLTGLWPINTLK	78	ctpr	C [66] 57.0215	ENSP00000205948	Beta-2-glycoprotein 1 Precursor (Beta-2-glycoprotein I)(Beta(2)GPI)(B2GPI)(Apolipoprotein H)(Apo-H)(Activated protein C-binding protein)(APC inhibitor)(Anticardiolipin cofactor) [Source: UniProt P02749]
4040.1.1	-6	4.58	1772.9768	0.0003	2	46.54	gmrk	64	FICPLTGLWPINTLK	78	ctpr	C [66] 57.0215	ENSP00000205948	Beta-2-glycoprotein 1 Precursor (Beta-2-glycoprotein I)(Beta(2)GPI)(B2GPI)(Apolipoprotein H)(Apo-H)(Activated protein C-binding protein)(APC inhibitor)(Anticardiolipin cofactor) [Source: UniProt P02749]
4042.1.1	-6.1	4.55	1772.9768	0.0004	2	46.54	gmrk	64	FICPLTGLWPINTLK	78	ctpr	C [66] 57.0215	ENSP00000205948	Beta-2-glycoprotein 1 Precursor (Beta-2-glycoprotein I)(Beta(2)GPI)(B2GPI)(Apolipoprotein H)(Apo-H)(Activated protein C-binding protein)(APC inhibitor)(Anticardiolipin cofactor) [Source: UniProt P02749]
4047.1.1	-2.2	3.54	2714.3072	-0.0072	3	38.86	revk	205	CPFFPSRPDNGFVNYPAK	227	dkat	C [205] 57.0215, C [205] -17.0265	ENSP00000205948	Beta-2-glycoprotein 1 Precursor (Beta-2-glycoprotein I)(Beta(2)GPI)(B2GPI)(Apolipoprotein H)(Apo-H)(Activated protein C-binding protein)(APC inhibitor)(Anticardiolipin cofactor) [Source: UniProt P02749]
4072.1.1	-8	4.32	1772.9768	0.0012	2	46.54	gmrk	64	FICPLTGLWPINTLK	78	ctpr	C [66] 57.0215	ENSP00000205948	Beta-2-glycoprotein 1 Precursor (Beta-2-glycoprotein I)(Beta(2)GPI)(B2GPI)(Apolipoprotein H)(Apo-H)(Activated protein C-binding protein)(APC inhibitor)(Anticardiolipin cofactor) [Source: UniProt P02749]
4074.1.1	-5.6	4.34	1772.9768	0.0011	2	46.54	gmrk	64	FICPLTGLWPINTLK	78	ctpr	C [66] 57.0215	ENSP00000205948	Beta-2-glycoprotein 1 Precursor (Beta-2-glycoprotein I)(Beta(2)GPI)(B2GPI)(Apolipoprotein H)(Apo-H)(Activated protein C-binding protein)(APC inhibitor)(Anticardiolipin cofactor) [Source: UniProt P02749]
4076.1.1	-6.7	4.34	1772.9768	0.0009	2	46.54	gmrk	64	FICPLTGLWPINTLK	78	ctpr	C [66] 57.0215	ENSP00000205948	Beta-2-glycoprotein 1 Precursor (Beta-2-glycoprotein I)(Beta(2)GPI)(B2GPI)(Apolipoprotein H)(Apo-H)(Activated protein C-binding protein)(APC inhibitor)(Anticardiolipin cofactor) [Source: UniProt P02749]

Sample Beta-2-GPI/TRX-1/TRX-R/NADPH

False discovery rate 0.71%

Proteins

rank	log(e)	log(I)	% (measured	% (corrected	unique	total	Mr	Accession	Description
1	-1103.9	8.35	81	100+	98	534	38.3	P02749	APOH, Beta-2-glycoprotein 1 Precursor (Beta-2-glycoprotein I)(Beta(2)GPI)(B2GPI)(Apolipoprotein H)(Apo-H)(Activated protein C-binding protein)(APC inhibitor)(Anticardiolipin cofactor) [Source: UniProt P02749]
2	-101.9	6.12	25	38	12	27	51.2	P27918	CFP, Properdin precursor (Factor P). Source: Uniprot/SWISSPROT P27918
3	-80.2	5.75	14	23	9	19	66	P04264	KRT1, Keratin, type II cytoskeletal 1 (Cytokeratin-1) (CK-1) (Keratin-1) (K1) (67 kDa cytokeratin) (Hair alpha protein). Source: Uniprot/SWISSPROT P04264
4	-69.4	5.51	11	14	7	12	65.4	P35908	KRT2, Keratin, type II cytoskeletal 2 epidermal (Cytokeratin-2e) (K2e) (CK 2e) (keratin-2). Source: Uniprot/SWISSPROT P35908
5	-67.2	6.09	20	24	7	22	54.5	ENSP0000	TXNRD1, Thioredoxin reductase 1, cytoplasmic (TR)(EC 1.8.1.9)(Thioredoxin reductase TR1)(KM-102-derived reductase-like factor)(Gene associated with retinoid-IFN-induced mortality 12 protein)(GRIM-12) [Source: UniProt Q16881] Source: 1.8.1.9
6	-61.6	6.43	48	55	8	23	11.7	P10599	TXN, Thioredoxin (Trx)(ATL-derived factor)(ADF)(Surface-associated sulphydryl protein)(SASP) [Source: UniProt P10599]
7	-56.2	5.41	10	19	6	12	58.8	P13645	KRT10, Keratin, type I cytoskeletal 10 (Cytokeratin-10) (CK-10) (Keratin-10) (K10). Source: Uniprot/SWISSPROT P13645
9	-24	4.68	7.6	11	3	4	45.6	P05154	SERPINA5, Plasma serine protease inhibitor precursor (PCI) (Protein C inhibitor) (Serpin A5) (Plasminogen activator inhibitor 3) (PAI-3) (PAI3) (Acrosomal serine protease inhibitor). Source: Uniprot/SWISSPROT P05154

Peptides

Spectrum	log(e)	log(I)	m+h	delta	z	retention	pre	start	sequence	end	post	modifications	Protein	Description
710.1.1	-1.3	3.82	1016.5921	0.0005	2	10	psck	262	ASCKVPVKK	270	atvv	C [264] 57.0215	ENSP00000205948	Beta-2-glycoprotein 1 Precursor (Beta-2-glycoprotein I)(Beta(2)GPI)(B2GPI)(Apolipoprotein H)(Apo-H)(Activated protein C-binding protein)(APC inhibitor)(Anticardiolipin cofactor) [Source: UniProt P027
714.1.1	-2.2	3.9	1016.5921	0.0014	2	10	psck	262	ASCKVPVKK	270	atvv	C [264] 57.0215	ENSP00000205948	Beta-2-glycoprotein 1 Precursor (Beta-2-glycoprotein I)(Beta(2)GPI)(B2GPI)(Apolipoprotein H)(Apo-H)(Activated protein C-binding protein)(APC inhibitor)(Anticardiolipin cofactor) [Source: UniProt P027
801.1.1	-3.3	3.73	871.4091	0.0022	2	14.39	ekfk	288	NGMLHGDK	295	vsff		ENSP00000205948	Beta-2-glycoprotein 1 Precursor (Beta-2-glycoprotein I)(Beta(2)GPI)(B2GPI)(Apolipoprotein H)(Apo-H)(Activated protein C-binding protein)(APC inhibitor)(Anticardiolipin cofactor) [Source: UniProt P027
803.1.1	-1	3.85	871.4091	0.0008	2	14.39	ekfk	288	NGMLHGDK	295	vsff		ENSP00000205948	Beta-2-glycoprotein 1 Precursor (Beta-2-glycoprotein I)(Beta(2)GPI)(B2GPI)(Apolipoprotein H)(Apo-H)(Activated protein C-binding protein)(APC inhibitor)(Anticardiolipin cofactor) [Source: UniProt P027
827.1.1	-1.4	4.22	1162.5674	0.0008	2	19.74	iqek	286	FKNGMLHGDK	295	vsff	M [290] 15.9949	ENSP00000205948	Beta-2-glycoprotein 1 Precursor (Beta-2-glycoprotein I)(Beta(2)GPI)(B2GPI)(Apolipoprotein H)(Apo-H)(Activated protein C-binding protein)(APC inhibitor)(Anticardiolipin cofactor) [Source: UniProt P027
829.1.1	-1.7	4.25	1162.5674	0.0019	2	19.74	iqek	286	FKNGMLHGDK	295	vsff	M [290] 15.9949	ENSP00000205948	Beta-2-glycoprotein 1 Precursor (Beta-2-glycoprotein I)(Beta(2)GPI)(B2GPI)(Apolipoprotein H)(Apo-H)(Activated protein C-binding protein)(APC inhibitor)(Anticardiolipin cofactor) [Source: UniProt P027
831.1.1	-1.8	4.38	1162.5674	0.0002	2	19.74	iqek	286	FKNGMLHGDK	295	vsff	M [290] 15.9949	ENSP00000205948	Beta-2-glycoprotein 1 Precursor (Beta-2-glycoprotein I)(Beta(2)GPI)(B2GPI)(Apolipoprotein H)(Apo-H)(Activated protein C-binding protein)(APC inhibitor)(Anticardiolipin cofactor) [Source: UniProt P027
842.1.1	-4.3	3.69	1120.5303	0.0016	2	8.43	lafw	336	KTDASDVKPC	345	]	C [345] 57.0215	ENSP00000205948	Beta-2-glycoprotein 1 Precursor (Beta-2-glycoprotein I)(Beta(2)GPI)(B2GPI)(Apolipoprotein H)(Apo-H)(Activated protein C-binding protein)(APC inhibitor)(Anticardiolipin cofactor) [Source: UniProt P027
844.1.1	-3.5	3.7	1120.5303	0.0019	2	8.43	lafw	336	KTDASDVKPC	345	]	C [345] 57.0215	ENSP00000205948	Beta-2-glycoprotein 1 Precursor (Beta-2-glycoprotein I)(Beta(2)GPI)(B2GPI)(Apolipoprotein H)(Apo-H)(Activated protein C-binding protein)(APC inhibitor)(Anticardiolipin cofactor) [Source: UniProt P027
846.1.1	-3.8	3.81	1120.5303	-0.0012	2	8.43	lafw	336	KTDASDVKPC	345	]	C [345] 57.0215	ENSP00000205948	Beta-2-glycoprotein 1 Precursor (Beta-2-glycoprotein I)(Beta(2)GPI)(B2GPI)(Apolipoprotein H)(Apo-H)(Activated protein C-binding protein)(APC inhibitor)(Anticardiolipin cofactor) [Source: UniProt P027
892.1.1	-1.9	3.9	1030.6078	-0.0003	2	10	psck	262	ASCKVPVKK	270	atvv	C [264] 57.0215, V [266] 14.0157	ENSP00000205948	Beta-2-glycoprotein 1 Precursor (Beta-2-glycoprotein I)(Beta(2)GPI)(B2GPI)(Apolipoprotein H)(Apo-H)(Activated protein C-binding protein)(APC inhibitor)(Anticardiolipin cofactor) [Source: UniProt P027
894.1.1	-4.1	3.94	1030.6078	0	2	10	psck	262	ASCKVPVKK	270	atvv	C [264] 57.0215, V [266] 14.0157	ENSP00000205948	Beta-2-glycoprotein 1 Precursor (Beta-2-glycoprotein I)(Beta(2)GPI)(B2GPI)(Apolipoprotein H)(Apo-H)(Activated protein C-binding protein)(APC inhibitor)(Anticardiolipin cofactor) [Source: UniProt P027
929.1.1	-2.2	3.95	1053.5146	0.0003	2	11.46	eity	50	SCKPGYVSR	58	ggmr	C [51] 57.0215	ENSP00000205948	Beta-2-glycoprotein 1 Precursor (Beta-2-glycoprotein I)(Beta(2)GPI)(B2GPI)(Apolipoprotein H)(Apo-H)(Activated protein C-binding protein)(APC inhibitor)(Anticardiolipin cofactor) [Source: UniProt P027
931.1.1	-3.2	4.31	1053.5146	0.003	3	11.46	eity	50	SCKPGYVSR	58	ggmr	C [51] 57.0215	ENSP00000205948	Beta-2-glycoprotein 1 Precursor (Beta-2-glycoprotein I)(Beta(2)GPI)(B2GPI)(Apolipoprotein H)(Apo-H)(Activated protein C-binding protein)(APC inhibitor)(Anticardiolipin cofactor) [Source: UniProt P027
932.1.1	-1.5	4.17	1163.5522	-0.0014	2	19.74	iqek	286	FKNGMLHGDK	295	vsff	M [290] 15.9949, N [288] 0.9848	ENSP00000205948	Beta-2-glycoprotein 1 Precursor (Beta-2-glycoprotein I)(Beta(2)GPI)(B2GPI)(Apolipoprotein H)(Apo-H)(Activated protein C-binding protein)(APC inhibitor)(Anticardiolipin cofactor) [Source: UniProt P027
935.1.1	-1.8	4.32	1163.5522	-0.0003	2	19.74	iqek	286	FKNGMLHGDK	295	vsff	M [290] 15.9949, N [288] 0.9848	ENSP00000205948	Beta-2-glycoprotein 1 Precursor (Beta-2-glycoprotein I)(Beta(2)GPI)(B2GPI)(Apolipoprotein H)(Apo-H)(Activated protein C-binding protein)(APC inhibitor)(Anticardiolipin cofactor) [Source: UniProt P027
936.1.1	-3.5	4.34	1053.5146	0.0022	3	11.46	eity	50	SCKPGYVSR	58	ggmr	C [51] 57.0215	ENSP00000205948	Beta-2-glycoprotein 1 Precursor (Beta-2-glycoprotein I)(Beta(2)GPI)(B2GPI)(Apolipoprotein H)(Apo-H)(Activated protein C-binding protein)(APC inhibitor)(Anticardiolipin cofactor) [Source: UniProt P027
942.1.1	-1.8	4.5	1163.5522	-0.0008	2	19.74	iqek	286	FKNGMLHGDK	295	vsff	M [290] 15.9949, N [288] 0.9848	ENSP00000205948	Beta-2-glycoprotein 1 Precursor (Beta-2-glycoprotein I)(Beta(2)GPI)(B2GPI)(Apolipoprotein H)(Apo-H)(Activated protein C-binding protein)(APC inhibitor)(Anticardiolipin cofactor) [Source: UniProt P027
963.1.1	-1.4	4.1	806.3275	-0.002	2	0.12	gkdr	411	GDEDQSR	417	eqma		ENSP00000328547	DNA (cytosine-5)-methyltransferase 3B (EC 2.1.1.37) (Dnmt3b) (DNA methyltransferase HsaIIIB) (DNA MTase HsaIIIB) (M.HsaIIIB). [Source: SWISSPROT (Q9UBC3)]
989.1.1	-3	3.91	992.4353	0.0022	2	9.52	afwk	337	TDASDVKPC	345	]	C [345] 57.0215	ENSP00000205948	Beta-2-glycoprotein 1 Precursor (Beta-2-glycoprotein I)(Beta(2)GPI)(B2GPI)(Apolipoprotein H)(Apo-H)(Activated protein C-binding protein)(APC inhibitor)(Anticardiolipin cofactor) [Source: UniProt P027
991.1.1	-6.6	4.29	992.4353	0.0006	2	9.52	afwk	337	TDASDVKPC	345	]	C [345] 57.0215	ENSP00000205948	Beta-2-glycoprotein 1 Precursor (Beta-2-glycoprotein I)(Beta(2)GPI)(B2GPI)(Apolipoprotein H)(Apo-H)(Activated protein C-binding protein)(APC inhibitor)(Anticardiolipin cofactor) [Source: UniProt P027
994.1.1	-7.3	4.67	992.4353	0.0011	2	9.52	afwk	337	TDASDVKPC	345	]	C [345] 57.0215	ENSP00000205948	Beta-2-glycoprotein 1 Precursor (Beta-2-glycoprotein I)(Beta(2)GPI)(B2GPI)(Apolipoprotein H)(Apo-H)(Activated protein C-binding protein)(APC inhibitor)(Anticardiolipin cofactor) [Source: UniProt P027
1017.1.1	-1.2	4.09	772.4563	0.0022	2	10.89	tlgr	11	GPTLKEK	17	slga		ENSP00000386178	
1030.1.1	-2.5	4.15	1190.5517	-0.0004	3	12.3	ayqk	67	RSGGLCQPCR	76	sprw	C [72] 57.0215, C [75] 57.0215	ENSP00000380189	Properdin precursor (Factor P). Source: Uniprot/SWISSPROT P27918
1034.1.1	-3	3.72	872.3939	0.0014	2	14.39	ekfk	288	NGMLHGDK	295	vsff	N [288] 0.9848	ENSP00000205948	Beta-2-glycoprotein 1 Precursor (Beta-2-glycoprotein I)(Beta(2)GPI)(B2GPI)(Apolipoprotein H)(Apo-H)(Activated protein C-binding protein)(APC inhibitor)(Anticardiolipin cofactor) [Source: UniProt P027
1080.1.1	-2.1	4.42	1050.4309	0	2	12.5	lyyk	228	DKATFGCHD	236	gysl	C [234] 57.0215	ENSP00000205948	Beta-2-glycoprotein 1 Precursor (Beta-2-glycoprotein I)(Beta(2)GPI)(B2GPI)(Apolipoprotein H)(Apo-H)(Activated protein C-binding protein)(APC inhibitor)(Anticardiolipin cofactor) [Source: UniProt P027
1084.1.1	-3.2	4.48	1050.4309	0.0006	2	12.5	lyyk	228	DKATFGCHD	236	gysl	C [234] 57.0215	ENSP00000205948	Beta-2-glycoprotein 1 Precursor (Beta-2-glycoprotein I)(Beta(2)GPI)(B2GPI)(Apolipoprotein H)(Apo-H)(Activated protein C-binding protein)(APC inhibitor)(Anticardiolipin cofactor) [Source: UniProt P027
1085.1.1	-6.4	4.33	992.4353	0.0013	2	9.52	afwk	337	TDASDVKPC	345	]	C [345] 57.0215	ENSP00000205948	Beta-2-glycoprotein 1 Precursor (Beta-2-glycoprotein I)(Beta(2)GPI)(B2GPI)(Apolipoprotein H)(Apo-H)(Activated protein C-binding protein)(APC inhibitor)(Anticardiolipin cofactor) [Source: UniProt P027
1090.1.1	-7.5	4.26	992.4353	0.0011	2	9.52	afwk	337	TDASDVKPC	345	]	C [345] 57.0215	ENSP00000205948	Beta-2-glycoprotein 1 Precursor (Beta-2-glycoprotein I)(Beta(2)GPI)(B2GPI)(Apolipoprotein H)(Apo-H)(Activated protein C-binding protein)(APC inhibitor)(Anticardiolipin cofactor) [Source: UniProt P027
1093.1.1	-2	4.06	1106.5259	-0.0015	2	17.79	gnek	148	VTMQNLNDR	156	lasy	M [150] 15.9949	ENSP00000269576	Keratin, type I cytoskeletal 10 (Cytokeratin-10) (CK-10) (Keratin-10) (K10). Source: Uniprot/SWISSPROT P13645
1097.1.1	-7.6	4.05	992.4353	0.0012	2	9.52	afwk	337	TDASDVKPC	345	]	C [345] 57.0215	ENSP00000205948	Beta-2-glycoprotein 1 Precursor (Beta-2-glycoprotein I)(Beta(2)GPI)(B2GPI)(Apolipoprotein H)(Apo-H)(Activated protein C-binding protein)(APC inhibitor)(Anticardiolipin cofactor) [Source: UniProt P027
1106.1.1	-4.1	4.41	1165.5848	-0.0009	2	14.83	kgqk	86	VGEFSGANKEK	96	leat		ENSP00000363641	Thioredoxin (Trx)(ATL-derived factor)(ADF)(Surface-associated sulphydryl protein)(SASP) [Source: UniProt P10599]
1110.1.1	-1.5	3.76	1106.5259	0.0004	2	17.79	gnek	148	VTMQNLNDR	156	lasy	M [150] 15.9949	ENSP00000269576	Keratin, type I cytoskeletal 10 (Cytokeratin-10) (CK-10) (Keratin-10) (K10). Source: Uniprot/SWISSPROT P13645
1153.1.1	-1	4.5	1194.6114	0.0025	3	12.64	dykk	267	KYEDEINKR	275	taae		ENSP00000310861	Keratin, type II cytoskeletal 2 epidermal (Cytokeratin-2e) (K2e) (CK 2e) (keratin-2). Source: Uniprot/SWISSPROT P35908
1174.1.1	-1.7	4.09	902.5128	0.0016	2	11	psck	262	ASCKVPVK	269	katv	C [264] 57.0215, V [266] 14.0157	ENSP00000205948	Beta-2-glycoprotein 1 Precursor (Beta-2-glycoprotein I)(Beta(2)GPI)(B2GPI)(Apolipoprotein H)(Apo-H)(Activated protein C-binding protein)(APC inhibitor)(Anticardiolipin cofactor) [Source: UniProt P027
1177.1.1	-2	4.32	902.5128	0.0013	2	11	psck	262	ASCKVPVK	269	katv	C [264] 57.0215, V [266] 14.0157	ENSP00000205948	Beta-2-glycoprotein 1 Precursor (Beta-2-glycoprotein I)(Beta(2)GPI)(B2GPI)(Apolipoprotein H)(Apo-H)(Activated protein C-binding protein)(APC inhibitor)(Anticardiolipin cofactor) [Source: UniProt P027
1180.1.1	-2.4	3.82	992.4353	0.0005	2	9.52	afwk	337	TDASDVKPC	345	]	C [345] 57.0215	ENSP00000205948	Beta-2-glycoprotein 1 Precursor (Beta-2-glycoprotein I)(Beta(2)GPI)(B2GPI)(Apolipoprotein H)(Apo-H)(Activated protein C-binding protein)(APC inhibitor)(Anticardiolipin cofactor) [Source: UniProt P027
1207.1.1	-1.8	4.69	1150.6215	0.0009	3	15.63	vpvk	270	KATVVYQGER	279	vkiq		ENSP00000205948	Beta-2-glycoprotein 1 Precursor (Beta-2-glycoprotein I)(Beta(2)GPI)(B2GPI)(Apolipoprotein H)(Apo-H)(Activated protein C-binding protein)(APC inhibitor)(Anticardiolipin cofactor) [Source: UniProt P027
1211.1.1	-3.7	5.39	1150.6215	0.0012	3	15.63	vpvk	270	KATVVYQGER	279	vkiq		ENSP00000205948	Beta-2-glycoprotein 1 Precursor (Beta-2-glycoprotein I)(Beta(2)GPI)(B2GPI)(Apolipoprotein H)(Apo-H)(Activated protein C-binding protein)(APC inhibitor)(Anticardiolipin cofactor) [Source: UniProt P027
1213.1.1	-8.7	4.01	1015.4401	-0.0043	2	16.35	tikk	290	DVDGAYMTK	298	vdlq	M [296] 15.9949	ENSP00000252244	Keratin, type II cytoskeletal 1 (Cytokeratin-1) (CK-1) (Keratin-1) (K1) (67 kDa cytokeratin) (Hair alpha protein). Source: Uniprot/SWISSPROT P04264
1215.1.1	-6.4	4.01	1015.4401	0.0001	2	16.35	tikk	290	DVDGAYMTK	298	vdlq	M [296] 15.9949	ENSP00000252244	Keratin, type II cytoskeletal 1 (Cytokeratin-1) (CK-1) (Keratin-1) (K1) (67 kDa cytokeratin) (Hair alpha protein). Source: Uniprot/SWISSPROT P04264
1223.1.1	-7.2	6.23	1150.6215	-0.001	2	15.63	vpvk	270	KATVVYQGER	279	vkiq		ENSP00000205948	Beta-2-glycoprotein 1 Precursor (Beta-2-glycoprotein I)(Beta(2)GPI)(B2GPI)(Apolipoprotein H)(Apo-H)(Activated protein C-binding protein)(APC inhibitor)(Anticardiolipin cofactor) [Source: UniProt P027
1228.1.1	-4.9	6.36	1150.6215	-0.0005	2	15.63	vpvk	270	KATVVYQGER	279	vkiq		ENSP00000205948	Beta-2-glycoprotein 1 Precursor (Beta-2-glycoprotein I)(Beta(2)GPI)(B2GPI)(Apolipoprotein H)(Apo-H)(Activated protein C-binding protein)(APC inhibitor)(Anticardiolipin cofactor) [Source: UniProt P027
1229.1.1	-1.8	4.6	716.4189	0.0006	2	13.81	qgqk	447	LVVEEK	452	rpcl		ENSP00000380189	Properdin precursor (Factor P). Source: Uniprot/SWISSPROT P27918
1232.1.1	-4.8	6.12	1150.6215	-0.001	2	15.63	vpvk	270	KATVVYQGER	279	vkiq		ENSP00000205948	Beta-2-glycoprotein 1 Precursor (Beta-2-glycoprotein I)(Beta(2)GPI)(B2GPI)(Apolipoprotein H)(Apo-H)(Activated protein C-binding protein)(APC inhibitor)(Anticardiolipin cofactor) [Source: UniProt P027
1233.1.1	-1.6	4.7	716.4189	-0.0002	2	13.81	qgqk	447	LVVEEK	452	rpcl		ENSP00000380189	Properdin precursor (Factor P). Source: Uniprot/SWISSPROT P27918
1239.1.1	-1.3	4.77	716.4189	0.0006	2	13.81	qgqk	447	LVVEEK	452	rpcl		ENSP00000380189	Properdin precursor (Factor P). Source: Uniprot/SWISSPROT P27918
1266.1.1	-4.4	4.64	908.4472	0.0003	2	15.82	kgqk	86	VGEFSGANK	94	ekle		ENSP00000363641	Thioredoxin (Trx)(ATL-derived factor)(ADF)(Surface-associated sulphydryl protein)(SASP) [Source: UniProt P10599]
1270.1.1	-4	4.82	908.4472	0.0008	2	15.82	kgqk	86	VGEFSGANK	94	ekle		ENSP00000363641	Thioredoxin (Trx)(ATL-derived factor)(ADF)(Surface-associated sulphydryl protein)(SASP) [Source: UniProt P10599]
1272.1.1	-4.6	5.06	908.4472	0.0006	2	15.82	kgqk	86	VGEFSGANK	94	ekle		ENSP00000363641	Thioredoxin (Trx)(ATL-derived factor)(ADF)(Surface-associated sulphydryl protein)(SASP) [Source: UniProt P10599]
1276.1.1	-5.6	4.15	1235.5287	-0.0024	2	11.6	gggr	47	FSSSSGYGGGSSR	59	vcgr		ENSP00000246662	Keratin, type I cytoskeletal 9 (Cytokeratin-9) (CK-9) (Keratin-9) (K9). Source: Uniprot/SWISSPROT P35527
1280.1.1	-4.6	4.29	1235.5287	0.001	2	11.6	gggr	47	FSSSSGYGGGSSR	59	vcgr		ENSP00000246662	Keratin, type I cytoskeletal 9 (Cytokeratin-9) (CK-9) (Keratin-9) (K9). Source: Uniprot/SWISSPROT P35527
1284.1.1	-6.9	4.25	1235.5287	-0.0002	2	11.6	gggr	47	FSSSSGYGGGSSR	59	vcgr		ENSP00000246662	Keratin, type I cytoskeletal 9 (Cytokeratin-9) (CK-9) (Keratin-9) (K9). Source: Uniprot/SWISSPROT P35527
1291.1.1	-1.6	4.67	1150.6215	0.0005	3	15.63	vpvk	270	KATVVYQGER	279	vkiq		ENSP00000205948	Beta-2-glycoprotein 1 Precursor (Beta-2-glycoprotein I)(Beta(2)GPI)(B2GPI)(Apolipoprotein H)(Apo-H)(Activated protein C-binding protein)(APC inhibitor)(Anticardiolipin cofactor) [Source: UniProt P027
1295.1.1	-1.9	4.57	1150.6215	0.0007	3	15.63	vpvk	270	KATVVYQGER	279	vkiq		ENSP00000205948	Beta-2-glycoprotein 1 Precursor (Beta-2-glycoprotein I)(Beta(2)GPI)(B2GPI)(Apolipoprotein H)(Apo-H)(Activated protein C-binding protein)(APC inhibitor)(Anticardiolipin cofactor) [Source: UniProt P027
1314.1.1	-1.3	4.22	1079.4939	-0.0002	2	22.55	ankr	428	CQLPDGSFR	436	rqrk	C [428] 57.0215	ENSP00000317123	U5 small nuclear ribonucleoprotein 200 kDa helicase (EC 3.6.1.-) (U5 snRNP-specific 200 kDa protein) (U5-200KD) (Activating signal cointegrator 1 complex subunit 3-like 1) (BRR2 homolog). Source: Unipr
1323.1.1	-1.2	4.34	853.4778	-0.0053	2	3.8	vtpk	25	SPPPEAKK	32	ears		ENSP00000300006:reversed	
1324.1.1	-3	4.3	1300.595	0.0024	3	18.78	ylkk	286	NHEEEMKDLR	295	nvst		ENSP00000269576	Keratin, type I cytoskeletal 10 (Cytokeratin-10) (CK-10) (Keratin-10) (K10). Source: Uniprot/SWISSPROT P13645
1329.1.1	-4.9	4.46	1151.6063	-0.0022	2	15.63	vpvk	270	KATVVYQGER	279	vkiq	Q [276] 0.9848	ENSP00000205948	Beta-2-glycoprotein 1 Precursor (Beta-2-glycoprotein I)(Beta(2)GPI)(B2GPI)(Apolipoprotein H)(Apo-H)(Activated protein C-binding protein)(APC inhibitor)(Anticardiolipin cofactor) [Source: UniProt P027
1330.1.1	-2.2	4.38	853.4778	-0.0053	2	3.8	vtpk	25	SPPPEAKK	32	ears		ENSP00000300006:reversed	
1334.1.1	-5.6	4.29	1151.6063	-0.0002	2	15.63	vpvk	270	KATVVYQGER	279	vkiq	Q [276] 0.9848	ENSP00000205948	Beta-2-glycoprotein 1 Precursor (Beta-2-glycoprotein I)(Beta(2)GPI)(B2GPI)(Apolipoprotein H)(Apo-H)(Activated protein C-binding protein)(APC inhibitor)(Anticardiolipin cofactor) [Source: UniProt P027
1346.1.1	-4.7	5.3	1062.4772	0.0004	2	13.56	ysld	242	GPEEIECTK	250	lgnw	C [248] 57.0215	ENSP00000205948	Beta-2-glycoprotein 1 Precursor (Beta-2-glycoprotein I)(Beta(2)GPI)(B2GPI)(Apolipoprotein H)(Apo-H)(Activated protein C-binding protein)(APC inhibitor)(Anticardiolipin cofactor) [Source: UniProt P027
1353.1.1	-3.7	5.5	1062.4772	0.0005	2	13.56	ysld	242	GPEEIECTK	250	lgnw	C [248] 57.0215	ENSP00000205948	Beta-2-glycoprotein 1 Precursor (Beta-2-glycoprotein I)(Beta(2)GPI)(B2GPI)(Apolipoprotein H)(Apo-H)(Activated protein C-binding protein)(APC inhibitor)(Anticardiolipin cofactor) [Source: UniProt P027
1356.1.1	-4.6	4.46	951.4895	-0.0005	2	14.63	vkka	272	TVVYQGER	279	vkiq		ENSP00000205948	Beta-2-glycoprotein 1 Precursor (Beta-2-glycoprotein I)(Beta(2)GPI)(B2GPI)(Apolipoprotein H)(Apo-H)(Activated protein C-binding protein)(APC inhibitor)(Anticardiolipin cofactor) [Source: UniProt P027
1359.1.1	-4.8	5.34	1062.4772	0.0005	2	13.56	ysld	242	GPEEIECTK	250	lgnw	C [248] 57.0215	ENSP00000205948	Beta-2-glycoprotein 1 Precursor (Beta-2-glycoprotein I)(Beta(2)GPI)(B2GPI)(Apolipoprotein H)(Apo-H)(Activated protein C-binding protein)(APC inhibitor)(Anticardiolipin cofactor) [Source: UniProt P027
1361.1.1	-4.7	4.65	951.4895	0.0006	2	14.63	vkka	272	TVVYQGER	279	vkiq		ENSP00000205948	Beta-2-glycoprotein 1 Precursor (Beta-2-glycoprotein I)(Beta(2)GPI)(B2GPI)(Apolipoprotein H)(Apo-H)(Activated protein C-binding protein)(APC inhibitor)(Anticardiolipin cofactor) [Source: UniProt P027
1366.1.1	-3.9	4.67	951.4895	0.0006	2	14.63	vkka	272	TVVYQGER	279	vkiq		ENSP00000205948	Beta-2-glycoprotein 1 Precursor (Beta-2-glycoprotein I)(Beta(2)GPI)(B2GPI)(Apolipoprotein H)(Apo-H)(Activated protein C-binding protein)(APC inhibitor)(Anticardiolipin cofactor) [Source: UniProt P027
1389.1.1	-2.7	3.82	1278.5994	-0.0022	2	19.21	segk	275	MQQVENGLSEK	285	tlrk	M [275] 15.9949	ENSP00000333203	Plasma serine protease inhibitor precursor (PCI) (Protein C inhibitor) (Serpin A5) (Plasminogen activator inhibitor 3) (PAI-3) (PAI3) (Acrosomal serine protease inhibitor). Source: Uniprot/SWISSPROT P0515
1401.1.1	-1.2	4.33	1019.6248	0.0012	2	20.3	qger	280	VKIQEKFK	287	ngml		ENSP00000205948	Beta-2-glycoprotein 1 Precursor (Beta-2-glycoprotein I)(Beta(2)GPI)(B2GPI)(Apolipoprotein H)(Apo-H)(Activated protein C-binding protein)(APC inhibitor)(Anticardiolipin cofactor) [Source: UniProt P027
1409.1.1	-1.3	4.32	1019.6248	0.0004	2	20.3	qger	280	VKIQEKFK	287	ngml		ENSP00000205948	Beta-2-glycoprotein 1 Precursor (Beta-2-glycoprotein I)(Beta(2)GPI)(B2GPI)(Apolipoprotein H)(Apo-H)(Activated protein C-binding protein)(APC inhibitor)(Anticardiolipin cofactor) [Source: UniProt P027
1423.1.1	-1.9	4.46	1147.5572	-0.0013	3	19.74	iqek	286	FKNGMLHGDK	295	vsff	N [288] 0.9848	ENSP00000205948	Beta-2-glycoprotein 1 Precursor (Beta-2-glycoprotein I)(Beta(2)GPI)(B2GPI)(Apolipoprotein H)(Apo-H)(Activated protein C-binding protein)(APC inhibitor)(Anticardiolipin cofactor) [Source: UniProt P027
1428.1.1	-1.1	4.36	1147.5572	0.0006	3	19.74	iqek	286	FKNGMLHGDK	295	vsff	N [288] 0.9848	ENSP00000205948	Beta-2-glycoprotein 1 Precursor (Beta-2-glycoprotein I)(Beta(2)GPI)(B2GPI)(Apolipoprotein H)(Apo-H)(Activated protein C-binding protein)(APC inhibitor)(Anticardiolipin cofactor) [Source: UniProt P027
1436.1.1	-2.9	4.5	1573.906	-0.0001	3	23.67	asck	266	VPVKKATVVYQGER	279	vkiq		ENSP00000205948	Beta-2-glycoprotein 1 Precursor (Beta-2-glycoprotein I)(Beta(2)GPI)(B2GPI)(Apolipoprotein H)(Apo-H)(Activated protein C-binding protein)(APC inhibitor)(Anticardiolipin cofactor) [Source: UniProt P027
1437.1.1	-2.9	4.41	1308.6542	0.001	3	12.69	edyr	268	NKYEDEINKR	277	tnae		ENSP00000252244	Keratin, type II cytoskeletal 1 (Cytokeratin-1) (CK-1) (Keratin-1) (K1) (67 kDa cytokeratin) (Hair alpha protein). Source: Uniprot/SWISSPROT P04264
1441.1.1	-5.6	4.57	1573.906	-0.0016	3	23.67	asck	266	VPVKKATVVYQGER	279	vkiq		ENSP00000205948	Beta-2-glycoprotein 1 Precursor (Beta-2-glycoprotein I)(Beta(2)GPI)(B2GPI)(Apolipoprotein H)(Apo-H)(Activated protein C-binding protein)(APC inhibitor)(Anticardiolipin cofactor) [Source: UniProt P027
1490.1.1	-5.3	6.64	1022.5265	0.0001	2	15.58	pvkk	271	ATVVYQGER	279	vkiq		ENSP00000205948	Beta-2-glycoprotein 1 Precursor (Beta-2-glycoprotein I)(Beta(2)GPI)(B2GPI)(Apolipoprotein H)(Apo-H)(Activated protein C-binding protein)(APC inhibitor)(Anticardiolipin cofactor) [Source: UniProt P027
1496.1.1	-6.1	6.46	1022.5265	0.0001	2	15.58	pvkk	271	ATVVYQGER	279	vkiq		ENSP00000205948	Beta-2-glycoprotein 1 Precursor (Beta-2-glycoprotein I)(Beta(2)GPI)(B2GPI)(Apolipoprotein H)(Apo-H)(Activated protein C-binding protein)(APC inhibitor)(Anticardiolipin cofactor) [Source: UniProt P027
1499.1.1	-6.4	6.3	1022.5265	0.0002	2	15.58	pvkk	271	ATVVYQGER	279	vkiq		ENSP00000205948	Beta-2-glycoprotein 1 Precursor (Beta-2-glycoprotein I)(Beta(2)GPI)(B2GPI)(Apolipoprotein H)(Apo-H)(Activated protein C-binding protein)(APC inhibitor)(Anticardiolipin cofactor) [Source: UniProt P027
1501.1.1	-1	4.24	1249.6899	0.002	2	19.13	pvkk	271	ATVVYQGERVK	281	iqek		ENSP00000205948	Beta-2-glycoprotein 1 Precursor (Beta-2-glycoprotein I)(Beta(2)GPI)(B2GPI)(Apolipoprotein H)(Apo-H)(Activated protein C-binding protein)(APC inhibitor)(Anticardiolipin cofactor) [Source: UniProt P027
1532.1.1	-3.9	4.2	1065.4993	0.0022	2	16.79	anek	155	STMQELNSR	163	lasy		ENSP00000246662	Keratin, type I cytoskeletal 9 (Cytokeratin-9) (CK-9) (Keratin-9) (K9). Source: Uniprot/SWISSPROT P35527

1542.1.1	-2.3	4.19	1065.4993	0.0011	2	16.79	anek	155	STMQELNSR	163	lasy		ENSP00000246662	Keratin, type I cytoskeletal 9 (Cytokeratin-9) (CK-9) (Keratin-9) (K9). Source: Uniprot/SWISSPROT P35527
1547.1.1	-2.1	4.07	1065.4993	0.0024	2	16.79	anek	155	STMQELNSR	163	lasy		ENSP00000246662	Keratin, type I cytoskeletal 9 (Cytokeratin-9) (CK-9) (Keratin-9) (K9). Source: Uniprot/SWISSPROT P35527
1578.1.1	-1.5	4.1	1493.7343	-0.0076	2	16.4	nnmr	323	SQYEQLAEQNRK	334	daea		ENSP00000269576	Keratin, type I cytoskeletal 10 (Cytokeratin-10) (CK-10) (Keratin-10) (K10). Source: Uniprot/SWISSPROT P13645
1583.1.1	-3.3	5.55	1023.5113	-0.0027	2	15.58	pvkk	271	ATVVYQGER	279	vkiq	Q [276] 0.9848	ENSP00000205948	Beta-2-glycoprotein 1 Precursor (Beta-2-glycoprotein I)(Beta(2)GPI)(B2GPI)(Apolipoprotein H)(Apo-H)(Activated protein C-binding protein)(APC inhibitor)(Anticardiolipin cofactor) [Source: UniProt P027
1585.1.1	-6.1	4.46	1177.5041	0.0002	2	14.35	gysl	241	DGPEEIECTK	250	lgnw	C [248] 57.0215	ENSP00000205948	Beta-2-glycoprotein 1 Precursor (Beta-2-glycoprotein I)(Beta(2)GPI)(B2GPI)(Apolipoprotein H)(Apo-H)(Activated protein C-binding protein)(APC inhibitor)(Anticardiolipin cofactor) [Source: UniProt P027
1586.1.1	-2	4.02	1493.7343	-0.0046	2	16.4	nnmr	323	SQYEQLAEQNRK	334	daea		ENSP00000269576	Keratin, type I cytoskeletal 10 (Cytokeratin-10) (CK-10) (Keratin-10) (K10). Source: Uniprot/SWISSPROT P13645
1590.1.1	-3	5.44	1023.5113	-0.0031	2	15.58	pvkk	271	ATVVYQGER	279	vkiq	Q [276] 0.9848	ENSP00000205948	Beta-2-glycoprotein 1 Precursor (Beta-2-glycoprotein I)(Beta(2)GPI)(B2GPI)(Apolipoprotein H)(Apo-H)(Activated protein C-binding protein)(APC inhibitor)(Anticardiolipin cofactor) [Source: UniProt P027
1591.1.1	-4.3	4.46	1177.5041	0.0001	2	14.35	gysl	241	DGPEEIECTK	250	lgnw	C [248] 57.0215	ENSP00000205948	Beta-2-glycoprotein 1 Precursor (Beta-2-glycoprotein I)(Beta(2)GPI)(B2GPI)(Apolipoprotein H)(Apo-H)(Activated protein C-binding protein)(APC inhibitor)(Anticardiolipin cofactor) [Source: UniProt P027
1594.1.1	-1.7	4.06	1058.4684	0.0042	2	8.45	dghr	360	CAGQQQDIR	368	hcys	C [360] 57.0215, C [360] -17.0265	ENSP00000380189	Properdin precursor (Factor P). Source: Uniprot/SWISSPROT P27918
1597.1.1	-2.8	5.3	1023.5113	-0.0048	2	15.58	pvkk	271	ATVVYQGER	279	vkiq	Q [276] 0.9848	ENSP00000205948	Beta-2-glycoprotein 1 Precursor (Beta-2-glycoprotein I)(Beta(2)GPI)(B2GPI)(Apolipoprotein H)(Apo-H)(Activated protein C-binding protein)(APC inhibitor)(Anticardiolipin cofactor) [Source: UniProt P027
1598.1.1	-8.2	4.15	1161.5746	0.0032	2	15.79	mvhk	351	AVVEVDESGTR	361	aaaa		ENSP00000333203	Plasma serine protease inhibitor precursor (PCI) (Protein C inhibitor) (Serpin A5) (Plasminogen activator inhibitor 3) (PAI-3) (PAI3) (Acrosomal serine protease inhibitor). Source: Uniprot/SWISSPROT P0515
1605.1.1	-2.4	4.4	1058.4684	0.0008	2	8.45	dghr	360	CAGQQQDIR	368	hcys	C [360] 57.0215, C [360] -17.0265	ENSP00000380189	Properdin precursor (Factor P). Source: Uniprot/SWISSPROT P27918
1607.1.1	-3	4.4	1442.6668	-0.0006	3	23.38	qdir	369	HCYSIQHCPLK	379	gsws	C [370] 57.0215, C [376] 57.0215	ENSP00000380189	Properdin precursor (Factor P). Source: Uniprot/SWISSPROT P27918
1608.1.1	-6.1	3.89	1161.5746	0.0023	2	15.79	mvhk	351	AVVEVDESGTR	361	aaaa		ENSP00000333203	Plasma serine protease inhibitor precursor (PCI) (Protein C inhibitor) (Serpin A5) (Plasminogen activator inhibitor 3) (PAI-3) (PAI3) (Acrosomal serine protease inhibitor). Source: Uniprot/SWISSPROT P0515
1611.1.1	-1.7	4.43	1442.6668	0.0024	3	23.38	qdir	369	HCYSIQHCPLK	379	gsws	C [370] 57.0215, C [376] 57.0215	ENSP00000380189	Properdin precursor (Factor P). Source: Uniprot/SWISSPROT P27918
1619.1.1	-1.1	4.32	1442.6668	0.0025	3	23.38	qdir	369	HCYSIQHCPLK	379	gsws	C [370] 57.0215, C [376] 57.0215	ENSP00000380189	Properdin precursor (Factor P). Source: Uniprot/SWISSPROT P27918
1648.1.1	-3.6	4.14	1252.5198	0.0017	2	14.4	ryrr	104	CVGWNGQCSGK	114	vapg	C [104] 57.0215, C [111] 57.0215	ENSP00000380189	Properdin precursor (Factor P). Source: Uniprot/SWISSPROT P27918
1653.1.1	-5.4	4.21	1252.5198	0.0002	2	14.4	ryrr	104	CVGWNGQCSGK	114	vapg	C [104] 57.0215, C [111] 57.0215	ENSP00000380189	Properdin precursor (Factor P). Source: Uniprot/SWISSPROT P27918
1659.1.1	-4.2	4.1	1252.5198	0.0004	2	14.4	ryrr	104	CVGWNGQCSGK	114	vapg	C [104] 57.0215, C [111] 57.0215	ENSP00000380189	Properdin precursor (Factor P). Source: Uniprot/SWISSPROT P27918
1667.1.1	-1.2	4.76	1131.5793	0.0012	2	7.89	tldr	1125	SPPPPPPTGER	1135	kllk		ENSP00000385618:reversed	
1670.1.1	-1.3	4.76	1131.5793	0.0007	2	7.89	tldr	1125	SPPPPPPTGER	1135	kllk		ENSP00000385618:reversed	
1677.1.1	-2.3	4.53	1131.5793	0.0008	2	7.89	tldr	1125	SPPPPPPTGER	1135	kllk		ENSP00000385618:reversed	
1684.1.1	-4.6	4.86	1022.5265	0.001	2	15.58	pvkk	271	ATVVYQGER	279	vkiq		ENSP00000205948	Beta-2-glycoprotein 1 Precursor (Beta-2-glycoprotein I)(Beta(2)GPI)(B2GPI)(Apolipoprotein H)(Apo-H)(Activated protein C-binding protein)(APC inhibitor)(Anticardiolipin cofactor) [Source: UniProt P027
1687.1.1	-5	4.34	1107.5429	0.0012	2	16.01	aevk	354	AQYEEIAQR	362	skee		ENSP00000310861	Keratin, type II cytoskeletal 2 epidermal (Cytokeratin-2e) (K2e) (CK 2e) (keratin-2). Source: Uniprot/SWISSPROT P35908
1690.1.1	-4.5	4.84	1022.5265	0.0007	2	15.58	pvkk	271	ATVVYQGER	279	vkiq		ENSP00000205948	Beta-2-glycoprotein 1 Precursor (Beta-2-glycoprotein I)(Beta(2)GPI)(B2GPI)(Apolipoprotein H)(Apo-H)(Activated protein C-binding protein)(APC inhibitor)(Anticardiolipin cofactor) [Source: UniProt P027
1691.1.1	-2.3	4.7	2682.3127	-0.0034	4	22.8	trsr	223	KCSAPEPSQKPPGKPCPGLAYEQR	246	rctg	C [224] 57.0215, C [238] 57.0215	ENSP00000380189	Properdin precursor (Factor P). Source: Uniprot/SWISSPROT P27918
1694.1.1	-4.5	4.37	1107.5429	-0.0003	2	16.01	aevk	354	AQYEEIAQR	362	skee		ENSP00000310861	Keratin, type II cytoskeletal 2 epidermal (Cytokeratin-2e) (K2e) (CK 2e) (keratin-2). Source: Uniprot/SWISSPROT P35908
1697.1.1	-5.2	4.81	1022.5265	0.0007	2	15.58	pvkk	271	ATVVYQGER	279	vkiq		ENSP00000205948	Beta-2-glycoprotein 1 Precursor (Beta-2-glycoprotein I)(Beta(2)GPI)(B2GPI)(Apolipoprotein H)(Apo-H)(Activated protein C-binding protein)(APC inhibitor)(Anticardiolipin cofactor) [Source: UniProt P027
1701.1.1	-3.3	4.17	1107.5429	0.0002	2	16.01	aevk	354	AQYEEIAQR	362	skee		ENSP00000310861	Keratin, type II cytoskeletal 2 epidermal (Cytokeratin-2e) (K2e) (CK 2e) (keratin-2). Source: Uniprot/SWISSPROT P35908
1732.1.1	-8.5	4.28	1262.5971	-0.0025	2	18.28	qtyr	451	SLLEGEGSSGGGGR	464	gggs		ENSP00000269576	Keratin, type I cytoskeletal 10 (Cytokeratin-10) (CK-10) (Keratin-10) (K10). Source: Uniprot/SWISSPROT P13645
1733.1.1	-1.2	4.41	1147.5776	-0.0026	2	23.94	gqvr	2378	ISMIVDQGER	2387	vsqd		ENSP00000311684:reversed	
1743.1.1	-7	4.42	1254.6073	-0.0015	2	19.95	ggfr	21	GFSSGSAVVSGGSR	34	rsts		ENSP00000310861	Keratin, type II cytoskeletal 2 epidermal (Cytokeratin-2e) (K2e) (CK 2e) (keratin-2). Source: Uniprot/SWISSPROT P35908
1750.1.1	-1.1	4.32	1048.5422	0.0005	2	22.65	askk	157	ESVVYGGISR	166	lqad	E [157] -18.0106	ENSP00000387110	
1752.1.1	-3.3	3.87	1468.7543	-0.0014	3	22.59	atlr	155	VYKPSAGNNSLYR	167	dtav		ENSP00000205948	Beta-2-glycoprotein 1 Precursor (Beta-2-glycoprotein I)(Beta(2)GPI)(B2GPI)(Apolipoprotein H)(Apo-H)(Activated protein C-binding protein)(APC inhibitor)(Anticardiolipin cofactor) [Source: UniProt P027
1753.1.1	-7.3	4.14	1254.6073	0.0022	2	19.95	ggfr	21	GFSSGSAVVSGGSR	34	rsts		ENSP00000310861	Keratin, type II cytoskeletal 2 epidermal (Cytokeratin-2e) (K2e) (CK 2e) (keratin-2). Source: Uniprot/SWISSPROT P35908
1759.1.1	-2	4.27	1468.7543	0.0021	3	22.59	atlr	155	VYKPSAGNNSLYR	167	dtav		ENSP00000205948	Beta-2-glycoprotein 1 Precursor (Beta-2-glycoprotein I)(Beta(2)GPI)(B2GPI)(Apolipoprotein H)(Apo-H)(Activated protein C-binding protein)(APC inhibitor)(Anticardiolipin cofactor) [Source: UniProt P027
1760.1.1	-1.9	4.65	1033.516	0.0021	2	20.66	atyr	484	TLLEGEESR	492	msge		ENSP00000252244	Keratin, type II cytoskeletal 1 (Cytokeratin-1) (CK-1) (Keratin-1) (K1) (67 kDa cytokeratin) (Hair alpha protein). Source: Uniprot/SWISSPROT P04264
1761.1.1	-1.7	4.29	1090.531	-0.0021	2	17.79	gnek	148	VTMQNLNDR	156	lasy		ENSP00000269576	Keratin, type I cytoskeletal 10 (Cytokeratin-10) (CK-10) (Keratin-10) (K10). Source: Uniprot/SWISSPROT P13645
1765.1.1	-3	4.58	1033.516	0.0011	2	20.66	atyr	484	TLLEGEESR	492	msge		ENSP00000252244	Keratin, type II cytoskeletal 1 (Cytokeratin-1) (CK-1) (Keratin-1) (K1) (67 kDa cytokeratin) (Hair alpha protein). Source: Uniprot/SWISSPROT P04264
1768.1.1	-1.1	3.98	1468.7543	0.0027	3	22.59	atlr	155	VYKPSAGNNSLYR	167	dtav		ENSP00000205948	Beta-2-glycoprotein 1 Precursor (Beta-2-glycoprotein I)(Beta(2)GPI)(B2GPI)(Apolipoprotein H)(Apo-H)(Activated protein C-binding protein)(APC inhibitor)(Anticardiolipin cofactor) [Source: UniProt P027
1772.1.1	-2.7	4.54	1033.516	0.0006	2	20.66	atyr	484	TLLEGEESR	492	msge		ENSP00000252244	Keratin, type II cytoskeletal 1 (Cytokeratin-1) (CK-1) (Keratin-1) (K1) (67 kDa cytokeratin) (Hair alpha protein). Source: Uniprot/SWISSPROT P04264
1787.1.1	-5.6	4.64	1022.5265	0.0007	2	15.58	pvkk	271	ATVVYQGER	279	vkiq		ENSP00000205948	Beta-2-glycoprotein 1 Precursor (Beta-2-glycoprotein I)(Beta(2)GPI)(B2GPI)(Apolipoprotein H)(Apo-H)(Activated protein C-binding protein)(APC inhibitor)(Anticardiolipin cofactor) [Source: UniProt P027
1791.1.1	-5.3	4.59	1022.5265	0.0007	2	15.58	pvkk	271	ATVVYQGER	279	vkiq		ENSP00000205948	Beta-2-glycoprotein 1 Precursor (Beta-2-glycoprotein I)(Beta(2)GPI)(B2GPI)(Apolipoprotein H)(Apo-H)(Activated protein C-binding protein)(APC inhibitor)(Anticardiolipin cofactor) [Source: UniProt P027
1795.1.1	-8	4.29	1320.5828	0.0005	3	18.9	clsr	46	HGGGGGGFGGGGFGSR	61	slvg		ENSP00000310861	Keratin, type II cytoskeletal 2 epidermal (Cytokeratin-2e) (K2e) (CK 2e) (keratin-2). Source: Uniprot/SWISSPROT P35908
1800.1.1	-6.8	4.57	1022.5265	0.0007	2	15.58	pvkk	271	ATVVYQGER	279	vkiq		ENSP00000205948	Beta-2-glycoprotein 1 Precursor (Beta-2-glycoprotein I)(Beta(2)GPI)(B2GPI)(Apolipoprotein H)(Apo-H)(Activated protein C-binding protein)(APC inhibitor)(Anticardiolipin cofactor) [Source: UniProt P027
1827.1.1	-2.6	5.2	3672.547	0.967	3	28.56	scrt	17	SCSSRPCVPPSCHSCTLPGACNIPA	45	cnwf	C [18] 57.0215, C [23] 57.0215, C	ENSP00000377567	Keratin, type I cuticular Ha1 (Hair keratin, type I Ha1) (Keratin-31). Source: Uniprot/SWISSPROT Q15323
1833.1.1	-2.1	4.89	1489.5933	0.0044	2	17.47	nkek	306	KCSYTEDAQCID	317	gtie	C [307] 57.0215, C [315] 57.0215	ENSP00000205948	Beta-2-glycoprotein 1 Precursor (Beta-2-glycoprotein I)(Beta(2)GPI)(B2GPI)(Apolipoprotein H)(Apo-H)(Activated protein C-binding protein)(APC inhibitor)(Anticardiolipin cofactor) [Source: UniProt P027
1839.1.1	-2.1	5.31	1489.7064	-0.006	2	17.79	rnmk	334	SISCQEIPGQQSR	346	grtc	C [337] 57.0215	ENSP00000380189	Properdin precursor (Factor P). Source: Uniprot/SWISSPROT P27918
1840.1.1	-1.5	4.67	1131.5793	0.0011	2	7.89	tldr	1125	SPPPPPPTGER	1135	kllk		ENSP00000385618:reversed	
1842.1.1	-3.2	4.46	975.4353	0.0007	2	17.95	revk	205	CPFPSRPD	212	ngfv	C [205] 57.0215	ENSP00000205948	Beta-2-glycoprotein 1 Precursor (Beta-2-glycoprotein I)(Beta(2)GPI)(B2GPI)(Apolipoprotein H)(Apo-H)(Activated protein C-binding protein)(APC inhibitor)(Anticardiolipin cofactor) [Source: UniProt P027
1845.1.1	-4	5.39	1489.7064	-0.0025	2	17.79	rnmk	334	SISCQEIPGQQSR	346	grtc	C [337] 57.0215	ENSP00000380189	Properdin precursor (Factor P). Source: Uniprot/SWISSPROT P27918
1859.1.1	-2.7	4.44	1027.3938	0.0008	2	15.75	ykdk	230	ATFGCHDGY	238	sldg	C [234] 57.0215	ENSP00000205948	Beta-2-glycoprotein 1 Precursor (Beta-2-glycoprotein I)(Beta(2)GPI)(B2GPI)(Apolipoprotein H)(Apo-H)(Activated protein C-binding protein)(APC inhibitor)(Anticardiolipin cofactor) [Source: UniProt P027
1865.1.1	-2.2	4.45	1027.3938	0.0006	2	15.75	ykdk	230	ATFGCHDGY	238	sldg	C [234] 57.0215	ENSP00000205948	Beta-2-glycoprotein 1 Precursor (Beta-2-glycoprotein I)(Beta(2)GPI)(B2GPI)(Apolipoprotein H)(Apo-H)(Activated protein C-binding protein)(APC inhibitor)(Anticardiolipin cofactor) [Source: UniProt P027
1872.1.1	-2.5	4.27	1027.3938	0.0006	2	15.75	ykdk	230	ATFGCHDGY	238	sldg	C [234] 57.0215	ENSP00000205948	Beta-2-glycoprotein 1 Precursor (Beta-2-glycoprotein I)(Beta(2)GPI)(B2GPI)(Apolipoprotein H)(Apo-H)(Activated protein C-binding protein)(APC inhibitor)(Anticardiolipin cofactor) [Source: UniProt P027
1873.1.1	-1.1	3.97	838.5033	0.0023	2	21.97	vsnk	210	ALPAPIEK	217	tisk		ENSP00000374990	Immunoglobulin heavy chain C gene segment Source: IMGT/GENE_DB IGHG1
1882.1.1	-4.5	4.45	1022.5265	0.0006	2	15.58	pvkk	271	ATVVYQGER	279	vkiq		ENSP00000205948	Beta-2-glycoprotein 1 Precursor (Beta-2-glycoprotein I)(Beta(2)GPI)(B2GPI)(Apolipoprotein H)(Apo-H)(Activated protein C-binding protein)(APC inhibitor)(Anticardiolipin cofactor) [Source: UniProt P027
1890.1.1	-4.9	4.54	1022.5265	0.0004	2	15.58	pvkk	271	ATVVYQGER	279	vkiq		ENSP00000205948	Beta-2-glycoprotein 1 Precursor (Beta-2-glycoprotein I)(Beta(2)GPI)(B2GPI)(Apolipoprotein H)(Apo-H)(Activated protein C-binding protein)(APC inhibitor)(Anticardiolipin cofactor) [Source: UniProt P027
1893.1.1	-3.9	4.61	1022.5265	0.0009	2	15.58	pvkk	271	ATVVYQGER	279	vkiq		ENSP00000205948	Beta-2-glycoprotein 1 Precursor (Beta-2-glycoprotein I)(Beta(2)GPI)(B2GPI)(Apolipoprotein H)(Apo-H)(Activated protein C-binding protein)(APC inhibitor)(Anticardiolipin cofactor) [Source: UniProt P027
1898.1.1	-1.5	4.2	1747.9701	0.0004	3	25.15	pvkk	271	ATVVYQGERVKIQEK	285	fkng		ENSP00000205948	Beta-2-glycoprotein 1 Precursor (Beta-2-glycoprotein I)(Beta(2)GPI)(B2GPI)(Apolipoprotein H)(Apo-H)(Activated protein C-binding protein)(APC inhibitor)(Anticardiolipin cofactor) [Source: UniProt P027
1932.1.1	-2.7	4.54	2693.2923	0.0067	4	23.3	rsrk	224	CSAPEPSQKPPGKPCPGLAYEQRR	247	ctgl	C [224] 57.0215, C [238] 57.0215,	ENSP00000380189	Properdin precursor (Factor P). Source: Uniprot/SWISSPROT P27918
1954.1.1	-2.6	4.4	928.5211	0.0012	2	22.91	cpfa	88	GILENGAVR	96	yttf		ENSP00000205948	Beta-2-glycoprotein 1 Precursor (Beta-2-glycoprotein I)(Beta(2)GPI)(B2GPI)(Apolipoprotein H)(Apo-H)(Activated protein C-binding protein)(APC inhibitor)(Anticardiolipin cofactor) [Source: UniProt P027
1956.1.1	-3	4.73	928.5211	0.0003	2	22.91	cpfa	88	GILENGAVR	96	yttf		ENSP00000205948	Beta-2-glycoprotein 1 Precursor (Beta-2-glycoprotein I)(Beta(2)GPI)(B2GPI)(Apolipoprotein H)(Apo-H)(Activated protein C-binding protein)(APC inhibitor)(Anticardiolipin cofactor) [Source: UniProt P027
1957.1.1	-2.9	4.25	1266.5606	-0.0001	2	25.37	ectk	251	LGNWSAMPSCK	261	asck	C [260] 57.0215, M [257] 15.9949	ENSP00000205948	Beta-2-glycoprotein 1 Precursor (Beta-2-glycoprotein I)(Beta(2)GPI)(B2GPI)(Apolipoprotein H)(Apo-H)(Activated protein C-binding protein)(APC inhibitor)(Anticardiolipin cofactor) [Source: UniProt P027
1959.1.1	-3.1	4.71	928.5211	0.0007	2	22.91	cpfa	88	GILENGAVR	96	yttf		ENSP00000205948	Beta-2-glycoprotein 1 Precursor (Beta-2-glycoprotein I)(Beta(2)GPI)(B2GPI)(Apolipoprotein H)(Apo-H)(Activated protein C-binding protein)(APC inhibitor)(Anticardiolipin cofactor) [Source: UniProt P027
1961.1.1	-3.5	4.37	1266.5606	0	2	25.37	ectk	251	LGNWSAMPSCK	261	asck	C [260] 57.0215, M [257] 15.9949	ENSP00000205948	Beta-2-glycoprotein 1 Precursor (Beta-2-glycoprotein I)(Beta(2)GPI)(B2GPI)(Apolipoprotein H)(Apo-H)(Activated protein C-binding protein)(APC inhibitor)(Anticardiolipin cofactor) [Source: UniProt P027
1970.1.1	-4.9	4.39	1266.5606	-0.0003	2	25.37	ectk	251	LGNWSAMPSCK	261	asck	C [260] 57.0215, M [257] 15.9949	ENSP00000205948	Beta-2-glycoprotein 1 Precursor (Beta-2-glycoprotein I)(Beta(2)GPI)(B2GPI)(Apolipoprotein H)(Apo-H)(Activated protein C-binding protein)(APC inhibitor)(Anticardiolipin cofactor) [Source: UniProt P027
1981.1.1	-3.7	4.48	1022.5265	0.0034	2	15.58	pvkk	271	ATVVYQGER	279	vkiq		ENSP00000205948	Beta-2-glycoprotein 1 Precursor (Beta-2-glycoprotein I)(Beta(2)GPI)(B2GPI)(Apolipoprotein H)(Apo-H)(Activated protein C-binding protein)(APC inhibitor)(Anticardiolipin cofactor) [Source: UniProt P027
1987.1.1	-4.8	4.54	1022.5265	0.0021	2	15.58	pvkk	271	ATVVYQGER	279	vkiq		ENSP00000205948	Beta-2-glycoprotein 1 Precursor (Beta-2-glycoprotein I)(Beta(2)GPI)(B2GPI)(Apolipoprotein H)(Apo-H)(Activated protein C-binding protein)(APC inhibitor)(Anticardiolipin cofactor) [Source: UniProt P027
1990.1.1	-2.9	4.55	1022.5265	0.0042	2	15.58	pvkk	271	ATVVYQGER	279	vkiq		ENSP00000205948	Beta-2-glycoprotein 1 Precursor (Beta-2-glycoprotein I)(Beta(2)GPI)(B2GPI)(Apolipoprotein H)(Apo-H)(Activated protein C-binding protein)(APC inhibitor)(Anticardiolipin cofactor) [Source: UniProt P027
1999.1.1	-1.3	4.3	842.5094	-0.0007	2	21.3	lnsr	108	VATVSLPR	115	scaa		sp|TRYP_PIG|	Trypsin; EC 3.4.21.4; Flags: Precursor;
2002.1.1	-2.8	4.36	1842.8691	0.0027	3	26.11	tfye	43	PGEEITYSCKPGYVSR	58	ggmr	C [51] 57.0215	ENSP00000205948	Beta-2-glycoprotein 1 Precursor (Beta-2-glycoprotein I)(Beta(2)GPI)(B2GPI)(Apolipoprotein H)(Apo-H)(Activated protein C-binding protein)(APC inhibitor)(Anticardiolipin cofactor) [Source: UniProt P027
2003.1.1	-4	4.06	1290.5882	0.0036	2	20.11	dgys	240	LDGPEEIECTK	250	lgnw	C [248] 57.0215	ENSP00000205948	Beta-2-glycoprotein 1 Precursor (Beta-2-glycoprotein I)(Beta(2)GPI)(B2GPI)(Apolipoprotein H)(Apo-H)(Activated protein C-binding protein)(APC inhibitor)(Anticardiolipin cofactor) [Source: UniProt P027
2004.1.1	-2.1	4.15	1131.5099	0.0071	2	22.2	gsrr	36	STSSFSCLSR	45	hggg	C [42] 57.0215	ENSP00000310861	Keratin, type II cytoskeletal 2 epidermal (Cytokeratin-2e) (K2e) (CK 2e) (keratin-2). Source: Uniprot/SWISSPROT P35908
2011.1.1	-2.1	4.19	1290.5882	-0.0023	2	20.11	dgys	240	LDGPEEIECTK	250	lgnw	C [248] 57.0215	ENSP00000205948	Beta-2-glycoprotein 1 Precursor (Beta-2-glycoprotein I)(Beta(2)GPI)(B2GPI)(Apolipoprotein H)(Apo-H)(Activated protein C-binding protein)(APC inhibitor)(Anticardiolipin cofactor) [Source: UniProt P027
2014.1.1	-5.5	4.72	999.5582	0.0004	2	23.12	vcpf	87	AGILENGAVR	96	yttf		ENSP00000205948	Beta-2-glycoprotein 1 Precursor (Beta-2-glycoprotein I)(Beta(2)GPI)(B2GPI)(Apolipoprotein H)(Apo-H)(Activated protein C-binding protein)(APC inhibitor)(Anticardiolipin cofactor) [Source: UniProt P027
2018.1.1	-4	4.2	1290.5882	-0.0018	2	20.11	dgys	240	LDGPEEIECTK	250	lgnw	C [248] 57.0215	ENSP00000205948	Beta-2-glycoprotein 1 Precursor (Beta-2-glycoprotein I)(Beta(2)GPI)(B2GPI)(Apolipoprotein H)(Apo-H)(Activated protein C-binding protein)(APC inhibitor)(Anticardiolipin cofactor) [Source: UniProt P027
2021.1.1	-2.9	5.15	999.5582	0.0008	2	23.12	vcpf	87	AGILENGAVR	96	yttf		ENSP00000205948	Beta-2-glycoprotein 1 Precursor (Beta-2-glycoprotein I)(Beta(2)GPI)(B2GPI)(Apolipoprotein H)(Apo-H)(Activated protein C-binding protein)(APC inhibitor)(Anticardiolipin cofactor) [Source: UniProt P027
2024.1.1	-2	4.45	831.4934	0.0009	2	22.84	fgsr	62	SLVGLGGTK	70	sisi		ENSP00000310861	Keratin, type II cytoskeletal 2 epidermal (Cytokeratin-2e) (K2e) (CK 2e) (keratin-2). Source: Uniprot/SWISSPROT P35908
2028.1.1	-3.5	5.28	999.5582	0.001	2	23.12	vcpf	87	AGILENGAVR	96	yttf		ENSP00000205948	Beta-2-glycoprotein 1 Precursor (Beta-2-glycoprotein I)(Beta(2)GPI)(B2GPI)(Apolipoprotein H)(Apo-H)(Activated protein C-binding protein)(APC inhibitor)(Anticardiolipin cofactor) [Source: UniProt P027
2056.1.1	-5.1	4.2	1243.6721	0.001	2	26.46	gfvn	218	YPAKPTLYYK	227	dkat		ENSP00000205948	Beta-2-glycoprotein 1 Precursor (Beta-2-glycoprotein I)(Beta(2)GPI)(B2GPI)(Apolipoprotein H)(Apo-H)(Activated protein C-binding protein)(APC inhibitor)(Anticardiolipin cofactor) [Source: UniProt P027
2060.1.1	-6.1	4.46	2537.1912	0.0039	3	24.11	rsrk	224	CSAPEPSQKPPGKPCPGLAYEQR	246	rctg	C [224] 57.0215, C [238] 57.0215,	ENSP00000380189	Properdin precursor (Factor P). Source: Uniprot/SWISSPROT P27918
2061.1.1	-4.3	4.07	1243.6721	0.0031	2	26.46	gfvn	218	YPAKPTLYYK	227	dkat		ENSP00000205948	Beta-2-glycoprotein 1 Precursor (Beta-2-glycoprotein I)(Beta(2)GPI)(B2GPI)(Apolipoprotein H)(Apo-H)(Activated protein C-binding protein)(APC inhibitor)(Anticardiolipin cofactor) [Source: UniProt P027
2062.1.1	-4.9	4.48	1971.9115	0.0021	3	26.56	ktfy	42	EPGEEITYSCKPGYVSR	58	ggmr	C [51] 57.0215	ENSP00000205948	Beta-2-glycoprotein 1 Precursor (Beta-2-glycoprotein I)(Beta(2)GPI)(B2GPI)(Apolipoprotein H)(Apo-H)(Activated protein C-binding protein)(APC inhibitor)(Anticardiolipin cofactor) [Source: UniProt P027
2063.1.1	-5.2	3.98	1393.7322	-0.0008	2	25.63	inkr	278	TNAENEFVTIKK	289	dvdg		ENSP00000252244	Keratin, type II cytoskeletal 1 (Cytokeratin-1) (CK-1) (Keratin-1) (K1) (67 kDa cytokeratin) (Hair alpha protein). Source: Uniprot/SWISSPROT P04264
2064.1.1	-2.2	4.13	1234.679	0.0027	2	26.36	ddfr	236	LKYENEVALR	245	qsve		ENSP00000269576	Keratin, type I cytoskeletal 10 (Cytokeratin-10) (CK-10) (Keratin-10) (K10). Source: Uniprot/SWISSPROT P13645
2066.1.1	-6.8	4.44	2537.1912	0.0041	3	24.11	rsrk	224	CSAPEPSQKPPGKPCPGLAYEQR	246	rctg	C [224] 57.0215, C [238] 57.0215,	ENSP00000380189	Properdin precursor (Factor P). Source: Uniprot/SWISSPROT P27918
2067.1.1	-3.1	4.61	1971.9115	0.0018	3	26.56	ktfy	42	EPGEEITYSCKPGYVSR	58	ggmr	C [51] 57.0215	ENSP00000205948	Beta-2-glycoprotein 1 Precursor (Beta-2-glycoprotein I)(Beta(2)GPI)(B2GPI)(Apolipoprotein H)(Apo-H)(Activated protein C-binding protein)(APC inhibitor)(Anticardiolipin cofactor) [Source: UniProt P027
2068.1.1	-1.4	4.73	1393.7322	-0.0028	3	25.63	inkr	278	TNAENEFVTIKK	289	dvdg		ENSP00000252244	Keratin, type II cytoskeletal 1 (Cytokeratin-1) (CK-1) (Keratin-1) (K1) (67 kDa cytokeratin) (Hair alpha protein). Source: Uniprot/SWISSPROT P04264
2069.1.1	-6.8	4.36	1377.6204	0.0001	2	22.79	hdgy	239	SLDGPEEIECTK	250	lgnw	C [248] 57.0215	ENSP00000205948	Beta-2-glycoprotein 1 Precursor (Beta-2-glycoprotein I)(Beta(2)GPI)(B2GPI)(Apolipoprotein H)(Apo-H)(Activated protein C-binding protein)(APC inhibitor)(Anticardiolipin cofactor) [Source: UniProt P027
2070.1.1	-2.6	4.64	1234.679	-0.0008	3	26.36	ddfr	236	LKYENEVALR	245	qsve		ENSP00000269576	Keratin, type I cytoskeletal 10 (Cytokeratin-10) (CK-10) (Keratin-10) (K10). Source: Uniprot/SWISSPROT P13645
2073.1.1	-7.1	4.43	1377.6204	-0.001	2	22.79	hdgy	239	SLDGPEEIECTK	250	lgnw	C [248] 57.0215	ENSP00000205948	Beta-2-glycoprotein 1 Precursor (Beta-2-glycoprotein I)(Beta(2)GPI)(B2GPI)(Apolipoprotein H)(Apo-H)(Activated protein C-binding protein)(APC inhibitor)(Anticardiolipin cofactor) [Source: UniProt P027
2075.1.1	-5.6	4.59	1971.9115	-0.0001	3	26.56	ktfy	42	EPGEEITYSCKPGYVSR	58	ggmr	C [51] 57.0215	ENSP00000205948	Beta-2-glycoprotein 1 Precursor (Beta-2-glycoprotein I)(Beta(2)GPI)(B2GPI)(Apolipoprotein H)(Apo-H)(Activated protein C-binding protein)(APC inhibitor)(Anticardiolipin cofactor) [Source: UniProt P027
2077.1.1	-2.2	4.54	1393.7322	-0.0006	3	25.63	inkr	278	TNAENEFVTIKK	289	dvdg		ENSP00000252244	Keratin, type II cytoskeletal 1 (Cytokeratin-1) (CK-1) (Keratin-1) (K1) (67 kDa cytokeratin) (Hair alpha protein). Source: Uniprot/SWISSPROT P04264
2082.1.1	-8.5	4.58	1377.6204	-0.0002	2	22.79	hdgy	239	SLDGPEEIECTK	250	lgnw	C [248] 57.0215	ENSP00000205948	Beta-2-glycoprotein 1 Precursor (Beta-2-glycoprotein I)(Beta(2)GPI)(B2GPI)(Apolipoprotein H)(Apo-H)(Activated protein C-binding protein)(APC inhibitor)(Anticardiolipin cofactor) [Source: UniProt P027
2089.1.1	-3.9	4.56	1361.4985	0.0023	2	18.77	kekk	307	CSYTEDAQCID	317	gtie	C [307] 57.0215, C [315] 57.0215	ENSP00000205948	Beta-2-glycoprotein 1 Precursor (Beta-2-glycoprotein I)(Beta(2)GPI)(B2GPI)(Apolipoprotein H)(Apo-H)(Activated protein C-binding protein)(APC inhibitor)(Anticardiolipin cofactor) [Source: UniProt P027
2090.1.1	-5.2	4.34	1022.5265	0.001	2	15.58	pvkk	271	ATVVYQGER	279	vkiq		ENSP00000205948	Beta-2-glycoprotein 1 Precursor (Beta-2-glycoprotein I)(Beta(2)GPI)(B2GPI)(Apolipoprotein H)(Apo-H)(Activated protein C-binding protein)(APC inhibitor)(Anticardiolipin cofactor) [Source: UniProt P027

2094.1.1	-10.7	4.61	1381.6482	0.0008	2	24.44	dkvr	166	ALEESNYELEGK	177	ikew		ENSP00000269576	Keratin, type I cytoskeletal 10 (Cytokeratin-10) (CK-10) (Keratin-10) (K10). Source: Uniprot/SWISSPROT P13645
2101.1.1	-7.3	4.61	1381.6482	-0.0014	2	24.44	dkvr	166	ALEESNYELEGK	177	ikew		ENSP00000269576	Keratin, type I cytoskeletal 10 (Cytokeratin-10) (CK-10) (Keratin-10) (K10). Source: Uniprot/SWISSPROT P13645
2109.1.1	-7.5	4.32	1381.6482	0	2	24.44	dkvr	166	ALEESNYELEGK	177	ikew		ENSP00000269576	Keratin, type I cytoskeletal 10 (Cytokeratin-10) (CK-10) (Keratin-10) (K10). Source: Uniprot/SWISSPROT P13645
2110.1.1	-1.4	4.47	874.4993	0.0009	2	21.24	fgsr	66	SLVNLGGSK	74	sisi		ENSP00000252244	Keratin, type II cytoskeletal 1 (Cytokeratin-1) (CK-1) (Keratin-1) (K1) (67 kDa cytokeratin) (Hair alpha protein). Source: Uniprot/SWISSPROT P04264
2111.1.1	-5	4.3	1022.5265	0.0004	2	15.58	pvkk	271	ATVVYQGER	279	vkiq		ENSP00000205948	Beta-2-glycoprotein 1 Precursor (Beta-2-glycoprotein I)(Beta(2)GPI)(B2GPI)(Apolipoprotein H)(Apo-H)(Activated protein C-binding protein)(APC inhibitor)(Anticardiolipin cofactor) [Source: UniProt P027
2115.1.1	-3.5	4.31	1022.5265	0.0021	2	15.58	pvkk	271	ATVVYQGER	279	vkiq		ENSP00000205948	Beta-2-glycoprotein 1 Precursor (Beta-2-glycoprotein I)(Beta(2)GPI)(B2GPI)(Apolipoprotein H)(Apo-H)(Activated protein C-binding protein)(APC inhibitor)(Anticardiolipin cofactor) [Source: UniProt P027
2117.1.1	-3.1	4.24	1085.5262	0.001	2	22.88	cntg	114	FYLNGADSAK	123	ctee		ENSP00000205948	Beta-2-glycoprotein 1 Precursor (Beta-2-glycoprotein I)(Beta(2)GPI)(B2GPI)(Apolipoprotein H)(Apo-H)(Activated protein C-binding protein)(APC inhibitor)(Anticardiolipin cofactor) [Source: UniProt P027
2120.1.1	-4.1	4.81	971.5408	0.0007	2	22.57	daqc	316	IDGTIEVPK	324	cfke		ENSP00000205948	Beta-2-glycoprotein 1 Precursor (Beta-2-glycoprotein I)(Beta(2)GPI)(B2GPI)(Apolipoprotein H)(Apo-H)(Activated protein C-binding protein)(APC inhibitor)(Anticardiolipin cofactor) [Source: UniProt P027
2126.1.1	-4.9	5.14	971.5408	0.0013	2	22.57	daqc	316	IDGTIEVPK	324	cfke		ENSP00000205948	Beta-2-glycoprotein 1 Precursor (Beta-2-glycoprotein I)(Beta(2)GPI)(B2GPI)(Apolipoprotein H)(Apo-H)(Activated protein C-binding protein)(APC inhibitor)(Anticardiolipin cofactor) [Source: UniProt P027
2129.1.1	-4.3	4.37	1159.6582	0.0003	2	26.91	saer	157	FLIATGERPR	166	ylgi		ENSP00000373506	Thioredoxin reductase 1, cytoplasmic (TR)(EC 1.8.1.9)(Thioredoxin reductase TR1)(KM-102-derived reductase-like factor)(Gene associated with retinoid-IFN-induced mortality 12 protein)(GRIM-12) [Sc
2134.1.1	-4.3	5.11	971.5408	0.0013	2	22.57	daqc	316	IDGTIEVPK	324	cfke		ENSP00000205948	Beta-2-glycoprotein 1 Precursor (Beta-2-glycoprotein I)(Beta(2)GPI)(B2GPI)(Apolipoprotein H)(Apo-H)(Activated protein C-binding protein)(APC inhibitor)(Anticardiolipin cofactor) [Source: UniProt P027
2136.1.1	-4.7	4.7	1159.6582	0.0011	2	26.91	saer	157	FLIATGERPR	166	ylgi		ENSP00000373506	Thioredoxin reductase 1, cytoplasmic (TR)(EC 1.8.1.9)(Thioredoxin reductase TR1)(KM-102-derived reductase-like factor)(Gene associated with retinoid-IFN-induced mortality 12 protein)(GRIM-12) [Sc
2142.1.1	-4.2	4.6	1159.6582	0.0009	2	26.91	saer	157	FLIATGERPR	166	ylgi		ENSP00000373506	Thioredoxin reductase 1, cytoplasmic (TR)(EC 1.8.1.9)(Thioredoxin reductase TR1)(KM-102-derived reductase-like factor)(Gene associated with retinoid-IFN-induced mortality 12 protein)(GRIM-12) [Sc
2164.1.1	-2.1	4.25	1221.5898	0.0054	2	23.22	fpsr	211	PDNGFVNYPAK	221	ptly		ENSP00000205948	Beta-2-glycoprotein 1 Precursor (Beta-2-glycoprotein I)(Beta(2)GPI)(B2GPI)(Apolipoprotein H)(Apo-H)(Activated protein C-binding protein)(APC inhibitor)(Anticardiolipin cofactor) [Source: UniProt P027
2181.1.1	-6	4.54	2109.9546	0.0023	3	35.78	kcfk	328	EHSSLAFWKTDASDVKPC	345	]	C [345] 57.0215, W [335] 31.9898	ENSP00000205948	Beta-2-glycoprotein 1 Precursor (Beta-2-glycoprotein I)(Beta(2)GPI)(B2GPI)(Apolipoprotein H)(Apo-H)(Activated protein C-binding protein)(APC inhibitor)(Anticardiolipin cofactor) [Source: UniProt P027
2186.1.1	-3.1	4.68	2109.9546	-0.0004	3	35.78	kcfk	328	EHSSLAFWKTDASDVKPC	345	]	C [345] 57.0215, W [335] 31.9898	ENSP00000205948	Beta-2-glycoprotein 1 Precursor (Beta-2-glycoprotein I)(Beta(2)GPI)(B2GPI)(Apolipoprotein H)(Apo-H)(Activated protein C-binding protein)(APC inhibitor)(Anticardiolipin cofactor) [Source: UniProt P027
2192.1.1	-5.7	4.53	2109.9546	-0.0028	3	35.78	kcfk	328	EHSSLAFWKTDASDVKPC	345	]	C [345] 57.0215, W [335] 31.9898	ENSP00000205948	Beta-2-glycoprotein 1 Precursor (Beta-2-glycoprotein I)(Beta(2)GPI)(B2GPI)(Apolipoprotein H)(Apo-H)(Activated protein C-binding protein)(APC inhibitor)(Anticardiolipin cofactor) [Source: UniProt P027
2194.1.1	-3.4	4.53	2109.9546	0.0004	4	35.78	kcfk	328	EHSSLAFWKTDASDVKPC	345	]	C [345] 57.0215, W [335] 31.9898	ENSP00000205948	Beta-2-glycoprotein 1 Precursor (Beta-2-glycoprotein I)(Beta(2)GPI)(B2GPI)(Apolipoprotein H)(Apo-H)(Activated protein C-binding protein)(APC inhibitor)(Anticardiolipin cofactor) [Source: UniProt P027
2202.1.1	-3.9	4.07	1541.7415	0.0015	2	23.15	epge	46	EITYSCKPGYVSR	58	ggmr	C [51] 57.0215, E [46] -18.0106	ENSP00000205948	Beta-2-glycoprotein 1 Precursor (Beta-2-glycoprotein I)(Beta(2)GPI)(B2GPI)(Apolipoprotein H)(Apo-H)(Activated protein C-binding protein)(APC inhibitor)(Anticardiolipin cofactor) [Source: UniProt P027
2212.1.1	-4	5.44	1655.7669	0.0013	3	34.46	ekfk	288	NGMLHGDKVSFFCK	301	nkek	C [300] 57.0215, M [290] 15.9949	ENSP00000205948	Beta-2-glycoprotein 1 Precursor (Beta-2-glycoprotein I)(Beta(2)GPI)(B2GPI)(Apolipoprotein H)(Apo-H)(Activated protein C-binding protein)(APC inhibitor)(Anticardiolipin cofactor) [Source: UniProt P027
2215.1.1	-6.3	4.39	1670.7841	0.0026	2	24.08	yepg	45	EEITYSCKPGYVSR	58	ggmr	C [51] 57.0215, E [45] -18.0106	ENSP00000205948	Beta-2-glycoprotein 1 Precursor (Beta-2-glycoprotein I)(Beta(2)GPI)(B2GPI)(Apolipoprotein H)(Apo-H)(Activated protein C-binding protein)(APC inhibitor)(Anticardiolipin cofactor) [Source: UniProt P027
2219.1.1	-2.7	6.03	1655.7669	0.003	3	34.46	ekfk	288	NGMLHGDKVSFFCK	301	nkek	C [300] 57.0215, M [290] 15.9949	ENSP00000205948	Beta-2-glycoprotein 1 Precursor (Beta-2-glycoprotein I)(Beta(2)GPI)(B2GPI)(Apolipoprotein H)(Apo-H)(Activated protein C-binding protein)(APC inhibitor)(Anticardiolipin cofactor) [Source: UniProt P027
2222.1.1	-6.6	4.42	1670.7841	0.0048	2	24.08	yepg	45	EEITYSCKPGYVSR	58	ggmr	C [51] 57.0215, E [45] -18.0106	ENSP00000205948	Beta-2-glycoprotein 1 Precursor (Beta-2-glycoprotein I)(Beta(2)GPI)(B2GPI)(Apolipoprotein H)(Apo-H)(Activated protein C-binding protein)(APC inhibitor)(Anticardiolipin cofactor) [Source: UniProt P027
2226.1.1	-3.7	6.4	1655.7669	-0.0007	3	34.46	ekfk	288	NGMLHGDKVSFFCK	301	nkek	C [300] 57.0215, M [290] 15.9949	ENSP00000205948	Beta-2-glycoprotein 1 Precursor (Beta-2-glycoprotein I)(Beta(2)GPI)(B2GPI)(Apolipoprotein H)(Apo-H)(Activated protein C-binding protein)(APC inhibitor)(Anticardiolipin cofactor) [Source: UniProt P027
2227.1.1	-12.7	4.87	1655.7669	0.0022	2	34.46	ekfk	288	NGMLHGDKVSFFCK	301	nkek	C [300] 57.0215, M [290] 15.9949	ENSP00000205948	Beta-2-glycoprotein 1 Precursor (Beta-2-glycoprotein I)(Beta(2)GPI)(B2GPI)(Apolipoprotein H)(Apo-H)(Activated protein C-binding protein)(APC inhibitor)(Anticardiolipin cofactor) [Source: UniProt P027
2236.1.1	-9.9	4.7	1655.7669	0.0005	2	34.46	ekfk	288	NGMLHGDKVSFFCK	301	nkek	C [300] 57.0215, M [290] 15.9949	ENSP00000205948	Beta-2-glycoprotein 1 Precursor (Beta-2-glycoprotein I)(Beta(2)GPI)(B2GPI)(Apolipoprotein H)(Apo-H)(Activated protein C-binding protein)(APC inhibitor)(Anticardiolipin cofactor) [Source: UniProt P027
2243.1.1	-12	4.38	1655.7669	0.0008	2	34.46	ekfk	288	NGMLHGDKVSFFCK	301	nkek	C [300] 57.0215, M [290] 15.9949	ENSP00000205948	Beta-2-glycoprotein 1 Precursor (Beta-2-glycoprotein I)(Beta(2)GPI)(B2GPI)(Apolipoprotein H)(Apo-H)(Activated protein C-binding protein)(APC inhibitor)(Anticardiolipin cofactor) [Source: UniProt P027
2244.1.1	-4.6	4.54	1475.7853	-0.0018	2	27.75	dkvr	200	FLEQQNQVLQTK	211	well		ENSP00000252244	Keratin, type II cytoskeletal 1 (Cytokeratin-1) (CK-1) (Keratin-1) (K1) (67 kDa cytokeratin) (Hair alpha protein). Source: Uniprot/SWISSPROT P04264
2246.1.1	-1	5.5	1930.9302	0.0015	4	37.25	iqek	286	FKNGMLHGDKVSFFCK	301	nkek	C [300] 57.0215, M [290] 15.9949	ENSP00000205948	Beta-2-glycoprotein 1 Precursor (Beta-2-glycoprotein I)(Beta(2)GPI)(B2GPI)(Apolipoprotein H)(Apo-H)(Activated protein C-binding protein)(APC inhibitor)(Anticardiolipin cofactor) [Source: UniProt P027
2249.1.1	-9.3	4.5	1475.7853	-0.0008	2	27.75	dkvr	200	FLEQQNQVLQTK	211	well		ENSP00000252244	Keratin, type II cytoskeletal 1 (Cytokeratin-1) (CK-1) (Keratin-1) (K1) (67 kDa cytokeratin) (Hair alpha protein). Source: Uniprot/SWISSPROT P04264
2253.1.1	-1.7	5.86	1930.9302	0.0007	4	37.25	iqek	286	FKNGMLHGDKVSFFCK	301	nkek	C [300] 57.0215, M [290] 15.9949	ENSP00000205948	Beta-2-glycoprotein 1 Precursor (Beta-2-glycoprotein I)(Beta(2)GPI)(B2GPI)(Apolipoprotein H)(Apo-H)(Activated protein C-binding protein)(APC inhibitor)(Anticardiolipin cofactor) [Source: UniProt P027
2255.1.1	-4.2	5.16	1930.9302	-0.0008	3	37.25	iqek	286	FKNGMLHGDKVSFFCK	301	nkek	C [300] 57.0215, M [290] 15.9949	ENSP00000205948	Beta-2-glycoprotein 1 Precursor (Beta-2-glycoprotein I)(Beta(2)GPI)(B2GPI)(Apolipoprotein H)(Apo-H)(Activated protein C-binding protein)(APC inhibitor)(Anticardiolipin cofactor) [Source: UniProt P027
2256.1.1	-2	5.02	1087.5241	0.0013	3	25.69	gmlh	293	GDKVSFFCK	301	nkek	C [300] 57.0215	ENSP00000205948	Beta-2-glycoprotein 1 Precursor (Beta-2-glycoprotein I)(Beta(2)GPI)(B2GPI)(Apolipoprotein H)(Apo-H)(Activated protein C-binding protein)(APC inhibitor)(Anticardiolipin cofactor) [Source: UniProt P027
2260.1.1	-2.8	5.37	1930.9302	-0.0005	3	37.25	iqek	286	FKNGMLHGDKVSFFCK	301	nkek	C [300] 57.0215, M [290] 15.9949	ENSP00000205948	Beta-2-glycoprotein 1 Precursor (Beta-2-glycoprotein I)(Beta(2)GPI)(B2GPI)(Apolipoprotein H)(Apo-H)(Activated protein C-binding protein)(APC inhibitor)(Anticardiolipin cofactor) [Source: UniProt P027
2263.1.1	-2.7	4.74	1087.5241	-0.0009	2	25.69	gmlh	293	GDKVSFFCK	301	nkek	C [300] 57.0215	ENSP00000205948	Beta-2-glycoprotein 1 Precursor (Beta-2-glycoprotein I)(Beta(2)GPI)(B2GPI)(Apolipoprotein H)(Apo-H)(Activated protein C-binding protein)(APC inhibitor)(Anticardiolipin cofactor) [Source: UniProt P027
2266.1.1	-4	5.35	1930.9302	0.0003	3	37.25	iqek	286	FKNGMLHGDKVSFFCK	301	nkek	C [300] 57.0215, M [290] 15.9949	ENSP00000205948	Beta-2-glycoprotein 1 Precursor (Beta-2-glycoprotein I)(Beta(2)GPI)(B2GPI)(Apolipoprotein H)(Apo-H)(Activated protein C-binding protein)(APC inhibitor)(Anticardiolipin cofactor) [Source: UniProt P027
2274.1.1	-3.4	4.64	2078.9634	-0.0008	3	31.37	veek	453	RPCLHVPACKDPEEEEL	469	]	C [455] 57.0215, C [461] 57.0215	ENSP00000380189	Properdin precursor (Factor P). Source: Uniprot/SWISSPROT P27918
2282.1.1	-1.8	4.57	2078.9634	-0.0015	3	31.37	veek	453	RPCLHVPACKDPEEEEL	469	]	C [455] 57.0215, C [461] 57.0215	ENSP00000380189	Properdin precursor (Factor P). Source: Uniprot/SWISSPROT P27918
2284.1.1	-1.5	4.62	1888.864	0.99	4	15.01	spar	200	STWGGGGGAAGGRGGSGPAESR	221	nelk		ENSP00000353200:reversed	
2289.1.1	-2.9	4.32	2078.9634	0.0011	3	31.37	veek	453	RPCLHVPACKDPEEEEL	469	]	C [455] 57.0215, C [461] 57.0215	ENSP00000380189	Properdin precursor (Factor P). Source: Uniprot/SWISSPROT P27918
2294.1.1	-1.8	4.47	862.4669	0.0021	2	21.8	erpr	167	YLGIPGDK	174	eyci		ENSP00000373506	Thioredoxin reductase 1, cytoplasmic (TR)(EC 1.8.1.9)(Thioredoxin reductase TR1)(KM-102-derived reductase-like factor)(Gene associated with retinoid-IFN-induced mortality 12 protein)(GRIM-12) [Sc
2301.1.1	-2.7	4.44	862.4669	0.0006	2	21.8	erpr	167	YLGIPGDK	174	eyci		ENSP00000373506	Thioredoxin reductase 1, cytoplasmic (TR)(EC 1.8.1.9)(Thioredoxin reductase TR1)(KM-102-derived reductase-like factor)(Gene associated with retinoid-IFN-induced mortality 12 protein)(GRIM-12) [Sc
2304.1.1	-3.5	4.66	1671.7618	0.0001	3	34.46	ekfk	288	NGMLHGDKVSFFCK	301	nkek	C [300] 57.0215, M [290] 31.9898	ENSP00000205948	Beta-2-glycoprotein 1 Precursor (Beta-2-glycoprotein I)(Beta(2)GPI)(B2GPI)(Apolipoprotein H)(Apo-H)(Activated protein C-binding protein)(APC inhibitor)(Anticardiolipin cofactor) [Source: UniProt P027
2306.1.1	-1.9	4.32	862.4669	0.0012	2	21.8	erpr	167	YLGIPGDK	174	eyci		ENSP00000373506	Thioredoxin reductase 1, cytoplasmic (TR)(EC 1.8.1.9)(Thioredoxin reductase TR1)(KM-102-derived reductase-like factor)(Gene associated with retinoid-IFN-induced mortality 12 protein)(GRIM-12) [Sc
2312.1.1	-4.3	4.84	1671.7618	0.0012	3	34.46	ekfk	288	NGMLHGDKVSFFCK	301	nkek	C [300] 57.0215, M [290] 31.9898	ENSP00000205948	Beta-2-glycoprotein 1 Precursor (Beta-2-glycoprotein I)(Beta(2)GPI)(B2GPI)(Apolipoprotein H)(Apo-H)(Activated protein C-binding protein)(APC inhibitor)(Anticardiolipin cofactor) [Source: UniProt P027
2325.1.1	-7.1	4.49	1330.6671	-0.0006	2	24.18	yted	313	AQCIDGTIEVPK	324	cfke	C [315] 57.0215	ENSP00000205948	Beta-2-glycoprotein 1 Precursor (Beta-2-glycoprotein I)(Beta(2)GPI)(B2GPI)(Apolipoprotein H)(Apo-H)(Activated protein C-binding protein)(APC inhibitor)(Anticardiolipin cofactor) [Source: UniProt P027
2331.1.1	-6.7	4.93	1330.6671	-0.0009	2	24.18	yted	313	AQCIDGTIEVPK	324	cfke	C [315] 57.0215	ENSP00000205948	Beta-2-glycoprotein 1 Precursor (Beta-2-glycoprotein I)(Beta(2)GPI)(B2GPI)(Apolipoprotein H)(Apo-H)(Activated protein C-binding protein)(APC inhibitor)(Anticardiolipin cofactor) [Source: UniProt P027
2338.1.1	-7.3	5.33	1330.6671	0.0002	2	24.18	yted	313	AQCIDGTIEVPK	324	cfke	C [315] 57.0215	ENSP00000205948	Beta-2-glycoprotein 1 Precursor (Beta-2-glycoprotein I)(Beta(2)GPI)(B2GPI)(Apolipoprotein H)(Apo-H)(Activated protein C-binding protein)(APC inhibitor)(Anticardiolipin cofactor) [Source: UniProt P027
2342.1.1	-3.1	4.61	1849.7909	-0.0051	3	27.1	tfgc	235	HDGYSLDGPEEIECTK	250	lgnw	C [248] 57.0215	ENSP00000205948	Beta-2-glycoprotein 1 Precursor (Beta-2-glycoprotein I)(Beta(2)GPI)(B2GPI)(Apolipoprotein H)(Apo-H)(Activated protein C-binding protein)(APC inhibitor)(Anticardiolipin cofactor) [Source: UniProt P027
2343.1.1	-3	4.45	1179.6004	0.0002	2	24.27	yqsk	377	YEELQITAGR	386	hgds		ENSP00000252244	Keratin, type II cytoskeletal 1 (Cytokeratin-1) (CK-1) (Keratin-1) (K1) (67 kDa cytokeratin) (Hair alpha protein). Source: Uniprot/SWISSPROT P04264
2348.1.1	-3.8	4.41	1179.6004	-0.0002	2	24.27	yqsk	377	YEELQITAGR	386	hgds		ENSP00000252244	Keratin, type II cytoskeletal 1 (Cytokeratin-1) (CK-1) (Keratin-1) (K1) (67 kDa cytokeratin) (Hair alpha protein). Source: Uniprot/SWISSPROT P04264
2349.1.1	-6	4.41	2066.843	0.0037	3	25.46	katf	233	GCHDGYSLDGPEEIECTK	250	lgnw	C [234] 57.0215, C [248] 57.0215	ENSP00000205948	Beta-2-glycoprotein 1 Precursor (Beta-2-glycoprotein I)(Beta(2)GPI)(B2GPI)(Apolipoprotein H)(Apo-H)(Activated protein C-binding protein)(APC inhibitor)(Anticardiolipin cofactor) [Source: UniProt P027
2350.1.1	-3.2	4.29	1357.6382	0.0036	2	25.3	sfsc	111	NTGFYLNGADSAK	123	ctee		ENSP00000205948	Beta-2-glycoprotein 1 Precursor (Beta-2-glycoprotein I)(Beta(2)GPI)(B2GPI)(Apolipoprotein H)(Apo-H)(Activated protein C-binding protein)(APC inhibitor)(Anticardiolipin cofactor) [Source: UniProt P027
2352.1.1	-1.1	6.25	1931.915	0.0007	4	37.25	iqek	286	FKNGMLHGDKVSFFCK	301	nkek	C [300] 57.0215, M [290] 15.9949	ENSP00000205948	Beta-2-glycoprotein 1 Precursor (Beta-2-glycoprotein I)(Beta(2)GPI)(B2GPI)(Apolipoprotein H)(Apo-H)(Activated protein C-binding protein)(APC inhibitor)(Anticardiolipin cofactor) [Source: UniProt P027
2364.1.1	-1.5	5.88	1931.915	0.0004	3	37.25	iqek	286	FKNGMLHGDKVSFFCK	301	nkek	C [300] 57.0215, M [290] 15.9949	ENSP00000205948	Beta-2-glycoprotein 1 Precursor (Beta-2-glycoprotein I)(Beta(2)GPI)(B2GPI)(Apolipoprotein H)(Apo-H)(Activated protein C-binding protein)(APC inhibitor)(Anticardiolipin cofactor) [Source: UniProt P027
2367.1.1	-4.5	5.51	1656.7517	-0.0002	3	34.46	ekfk	288	NGMLHGDKVSFFCK	301	nkek	C [300] 57.0215, M [290] 15.9949	ENSP00000205948	Beta-2-glycoprotein 1 Precursor (Beta-2-glycoprotein I)(Beta(2)GPI)(B2GPI)(Apolipoprotein H)(Apo-H)(Activated protein C-binding protein)(APC inhibitor)(Anticardiolipin cofactor) [Source: UniProt P027
2373.1.1	-3.6	5.7	1656.7517	-0.0017	3	34.46	ekfk	288	NGMLHGDKVSFFCK	301	nkek	C [300] 57.0215, M [290] 15.9949	ENSP00000205948	Beta-2-glycoprotein 1 Precursor (Beta-2-glycoprotein I)(Beta(2)GPI)(B2GPI)(Apolipoprotein H)(Apo-H)(Activated protein C-binding protein)(APC inhibitor)(Anticardiolipin cofactor) [Source: UniProt P027
2382.1.1	-5.6	4.27	1243.5953	0.0008	2	25.86	fscn	112	TGFYLNGADSAK	123	ctee		ENSP00000205948	Beta-2-glycoprotein 1 Precursor (Beta-2-glycoprotein I)(Beta(2)GPI)(B2GPI)(Apolipoprotein H)(Apo-H)(Activated protein C-binding protein)(APC inhibitor)(Anticardiolipin cofactor) [Source: UniProt P027
2395.1.1	-4.7	4.59	1399.5583	-0.0003	2	24.27	ykdk	230	ATFGCHDGYSLDG	242	peei	C [234] 57.0215	ENSP00000205948	Beta-2-glycoprotein 1 Precursor (Beta-2-glycoprotein I)(Beta(2)GPI)(B2GPI)(Apolipoprotein H)(Apo-H)(Activated protein C-binding protein)(APC inhibitor)(Anticardiolipin cofactor) [Source: UniProt P027
2398.1.1	-4.1	4.52	1399.5583	-0.0004	2	24.27	ykdk	230	ATFGCHDGYSLDG	242	peei	C [234] 57.0215	ENSP00000205948	Beta-2-glycoprotein 1 Precursor (Beta-2-glycoprotein I)(Beta(2)GPI)(B2GPI)(Apolipoprotein H)(Apo-H)(Activated protein C-binding protein)(APC inhibitor)(Anticardiolipin cofactor) [Source: UniProt P027
2399.1.1	-5.1	4.78	1342.5368	0.0006	2	25.14	ykdk	230	ATFGCHDGYSLD	241	gpee	C [234] 57.0215	ENSP00000205948	Beta-2-glycoprotein 1 Precursor (Beta-2-glycoprotein I)(Beta(2)GPI)(B2GPI)(Apolipoprotein H)(Apo-H)(Activated protein C-binding protein)(APC inhibitor)(Anticardiolipin cofactor) [Source: UniProt P027
2404.1.1	-5.4	4.98	1342.5368	-0.0008	2	25.14	ykdk	230	ATFGCHDGYSLD	241	gpee	C [234] 57.0215	ENSP00000205948	Beta-2-glycoprotein 1 Precursor (Beta-2-glycoprotein I)(Beta(2)GPI)(B2GPI)(Apolipoprotein H)(Apo-H)(Activated protein C-binding protein)(APC inhibitor)(Anticardiolipin cofactor) [Source: UniProt P027
2410.1.1	-4.8	5.29	1342.5368	-0.0006	2	25.14	ykdk	230	ATFGCHDGYSLD	241	gpee	C [234] 57.0215	ENSP00000205948	Beta-2-glycoprotein 1 Precursor (Beta-2-glycoprotein I)(Beta(2)GPI)(B2GPI)(Apolipoprotein H)(Apo-H)(Activated protein C-binding protein)(APC inhibitor)(Anticardiolipin cofactor) [Source: UniProt P027
2422.1.1	-6.3	4.53	2214.0053	-0.0041	3	29.94	nkek	306	KCSYTEDAQCIDGTIEVPK	324	cfke	C [307] 57.0215, C [315] 57.0215	ENSP00000205948	Beta-2-glycoprotein 1 Precursor (Beta-2-glycoprotein I)(Beta(2)GPI)(B2GPI)(Apolipoprotein H)(Apo-H)(Activated protein C-binding protein)(APC inhibitor)(Anticardiolipin cofactor) [Source: UniProt P027
2427.1.1	-5.7	4.84	2214.0053	0.0009	3	29.94	nkek	306	KCSYTEDAQCIDGTIEVPK	324	cfke	C [307] 57.0215, C [315] 57.0215	ENSP00000205948	Beta-2-glycoprotein 1 Precursor (Beta-2-glycoprotein I)(Beta(2)GPI)(B2GPI)(Apolipoprotein H)(Apo-H)(Activated protein C-binding protein)(APC inhibitor)(Anticardiolipin cofactor) [Source: UniProt P027
2433.1.1	-1	5.44	1479.7664	0.0017	3	35.78	gpck	37	MIKPFFHSLSEK	48	ysnv	M [37] 15.9949	ENSP00000363641	Thioredoxin (Trx)(ATL-derived factor)(ADF)(Surface-associated sulphydryl protein)(SASP) [Source: UniProt P10599]
2434.1.1	-6.6	5.04	2214.0053	-0.003	3	29.94	nkek	306	KCSYTEDAQCIDGTIEVPK	324	cfke	C [307] 57.0215, C [315] 57.0215	ENSP00000205948	Beta-2-glycoprotein 1 Precursor (Beta-2-glycoprotein I)(Beta(2)GPI)(B2GPI)(Apolipoprotein H)(Apo-H)(Activated protein C-binding protein)(APC inhibitor)(Anticardiolipin cofactor) [Source: UniProt P027
2440.1.1	-1	5.72	1479.7664	0.0011	3	35.78	gpck	37	MIKPFFHSLSEK	48	ysnv	M [37] 15.9949	ENSP00000363641	Thioredoxin (Trx)(ATL-derived factor)(ADF)(Surface-associated sulphydryl protein)(SASP) [Source: UniProt P10599]
2442.1.1	-1.1	4.97	787.3807	0.0016	2	22.77	hgdk	296	VSFFCK	301	nkek	C [300] 57.0215	ENSP00000205948	Beta-2-glycoprotein 1 Precursor (Beta-2-glycoprotein I)(Beta(2)GPI)(B2GPI)(Apolipoprotein H)(Apo-H)(Activated protein C-binding protein)(APC inhibitor)(Anticardiolipin cofactor) [Source: UniProt P027
2443.1.1	-6.5	4.79	1656.7517	0.0005	2	34.46	ekfk	288	NGMLHGDKVSFFCK	301	nkek	C [300] 57.0215, M [290] 15.9949	ENSP00000205948	Beta-2-glycoprotein 1 Precursor (Beta-2-glycoprotein I)(Beta(2)GPI)(B2GPI)(Apolipoprotein H)(Apo-H)(Activated protein C-binding protein)(APC inhibitor)(Anticardiolipin cofactor) [Source: UniProt P027
2449.1.1	-7.8	5.07	1656.7517	0.001	2	34.46	ekfk	288	NGMLHGDKVSFFCK	301	nkek	C [300] 57.0215, M [290] 15.9949	ENSP00000205948	Beta-2-glycoprotein 1 Precursor (Beta-2-glycoprotein I)(Beta(2)GPI)(B2GPI)(Apolipoprotein H)(Apo-H)(Activated protein C-binding protein)(APC inhibitor)(Anticardiolipin cofactor) [Source: UniProt P027
2454.1.1	-2	6.04	787.3807	0.0011	2	22.77	hgdk	296	VSFFCK	301	nkek	C [300] 57.0215	ENSP00000205948	Beta-2-glycoprotein 1 Precursor (Beta-2-glycoprotein I)(Beta(2)GPI)(B2GPI)(Apolipoprotein H)(Apo-H)(Activated protein C-binding protein)(APC inhibitor)(Anticardiolipin cofactor) [Source: UniProt P027
2455.1.1	-5.1	5.04	1656.7517	0.0005	2	34.46	ekfk	288	NGMLHGDKVSFFCK	301	nkek	C [300] 57.0215, M [290] 15.9949	ENSP00000205948	Beta-2-glycoprotein 1 Precursor (Beta-2-glycoprotein I)(Beta(2)GPI)(B2GPI)(Apolipoprotein H)(Apo-H)(Activated protein C-binding protein)(APC inhibitor)(Anticardiolipin cofactor) [Source: UniProt P027
2468.1.1	-4.8	5.96	1656.7517	-0.0006	3	34.46	ekfk	288	NGMLHGDKVSFFCK	301	nkek	C [300] 57.0215, M [290] 15.9949	ENSP00000205948	Beta-2-glycoprotein 1 Precursor (Beta-2-glycoprotein I)(Beta(2)GPI)(B2GPI)(Apolipoprotein H)(Apo-H)(Activated protein C-binding protein)(APC inhibitor)(Anticardiolipin cofactor) [Source: UniProt P027
2475.1.1	-5.2	5.62	1656.7517	-0.0002	3	34.46	ekfk	288	NGMLHGDKVSFFCK	301	nkek	C [300] 57.0215, M [290] 15.9949	ENSP00000205948	Beta-2-glycoprotein 1 Precursor (Beta-2-glycoprotein I)(Beta(2)GPI)(B2GPI)(Apolipoprotein H)(Apo-H)(Activated protein C-binding protein)(APC inhibitor)(Anticardiolipin cofactor) [Source: UniProt P027
2479.1.1	-1.6	4.86	1571.7311	0.0019	3	36.42	evpk	325	CFKEHSSLAFWK	336	tdas	C [325] 57.0215, W [335] 31.9898	ENSP00000205948	Beta-2-glycoprotein 1 Precursor (Beta-2-glycoprotein I)(Beta(2)GPI)(B2GPI)(Apolipoprotein H)(Apo-H)(Activated protein C-binding protein)(APC inhibitor)(Anticardiolipin cofactor) [Source: UniProt P027
2481.1.1	-1.7	5.67	1486.631	-0.0057	2	25.76	sqsr	191	CCVPVCYQCKI	201	]	C [191] 57.0215, C [192] 57.0215,	ENSP00000355351	Keratin, ultra high-sulfur matrix protein A (UHS keratin A) (UHS KerA). [Source: UniProt P26371]
2482.1.1	-5.3	5.21	1656.7517	-0.0011	3	34.46	ekfk	288	NGMLHGDKVSFFCK	301	nkek	C [300] 57.0215, M [290] 15.9949	ENSP00000205948	Beta-2-glycoprotein 1 Precursor (Beta-2-glycoprotein I)(Beta(2)GPI)(B2GPI)(Apolipoprotein H)(Apo-H)(Activated protein C-binding protein)(APC inhibitor)(Anticardiolipin cofactor) [Source: UniProt P027
2486.1.1	-1.6	4.78	1571.7311	0.0022	3	36.42	evpk	325	CFKEHSSLAFWK	336	tdas	C [325] 57.0215, W [335] 31.9898	ENSP00000205948	Beta-2-glycoprotein 1 Precursor (Beta-2-glycoprotein I)(Beta(2)GPI)(B2GPI)(Apolipoprotein H)(Apo-H)(Activated protein C-binding protein)(APC inhibitor)(Anticardiolipin cofactor) [Source: UniProt P027
2488.1.1	-1.4	5.46	1486.631	-0.0063	2	25.76	sqsr	191	CCVPVCYQCKI	201	]	C [191] 57.0215, C [192] 57.0215,	ENSP00000355351	Keratin, ultra high-sulfur matrix protein A (UHS keratin A) (UHS KerA). [Source: UniProt P26371]
2490.1.1	-8.8	4.71	1540.6836	0.0002	2	26.42	chdg	238	YSLDGPEEIECTK	250	lgnw	C [248] 57.0215	ENSP00000205948	Beta-2-glycoprotein 1 Precursor (Beta-2-glycoprotein I)(Beta(2)GPI)(B2GPI)(Apolipoprotein H)(Apo-H)(Activated protein C-binding protein)(APC inhibitor)(Anticardiolipin cofactor) [Source: UniProt P027
2495.1.1	-7.5	4.57	1540.6836	-0.0008	2	26.42	chdg	238	YSLDGPEEIECTK	250	lgnw	C [248] 57.0215	ENSP00000205948	Beta-2-glycoprotein 1 Precursor (Beta-2-glycoprotein I)(Beta(2)GPI)(B2GPI)(Apolipoprotein H)(Apo-H)(Activated protein C-binding protein)(APC inhibitor)(Anticardiolipin cofactor) [Source: UniProt P027
2502.1.1	-5.5	4.34	1540.6836	-0.0007	2	26.42	chdg	238	YSLDGPEEIECTK	250	lgnw	C [248] 57.0215	ENSP00000205948	Beta-2-glycoprotein 1 Precursor (Beta-2-glycoprotein I)(Beta(2)GPI)(B2GPI)(Apolipoprotein H)(Apo-H)(Activated protein C-binding protein)(APC inhibitor)(Anticardiolipin cofactor) [Source: UniProt P027
2503.1.1	-9.7	4.64	1597.7051	0.0013	2	26.66	gchd	237	GYSLDGPEEIECTK	250	lgnw	C [248] 57.0215	ENSP00000205948	Beta-2-glycoprotein 1 Precursor (Beta-2-glycoprotein I)(Beta(2)GPI)(B2GPI)(Apolipoprotein H)(Apo-H)(Activated protein C-binding protein)(APC inhibitor)(Anticardiolipin cofactor) [Source: UniProt P027
2507.1.1	-8.5	4.91	1597.7051	0.0027	2	26.66	gchd	237	GYSLDGPEEIECTK	250	lgnw	C [248] 57.0215	ENSP00000205948	Beta-2-glycoprotein 1 Precursor (Beta-2-glycoprotein I)(Beta(2)GPI)(B2GPI)(Apolipoprotein H)(Apo-H)(Activated protein C-binding protein)(APC inhibitor)(Anticardiolipin cofactor) [Source: UniProt P027
2512.1.1	-8.1	4.54	2629.1181	0.0019	3	31.85	lyyk	228	DKATFGCHDGYSLDGPEEIECTK	250	lgnw	C [234] 57.0215, C [248] 57.0215	ENSP00000205948	Beta-2-glycoprotein 1 Precursor (Beta-2-glycoprotein I)(Beta(2)GPI)(B2GPI)(Apolipoprotein H)(Apo-H)(Activated protein C-binding protein)(APC inhibitor)(Anticardiolipin cofactor) [Source: UniProt P027
2514.1.1	-10	5.13	1597.7051	0.0062	2	26.66	gchd	237	GYSLDGPEEIECTK	250	lgnw	C [248] 57.0215	ENSP00000205948	Beta-2-glycoprotein 1 Precursor (Beta-2-glycoprotein I)(Beta(2)GPI)(B2GPI)(Apolipoprotein H)(Apo-H)(Activated protein C-binding protein)(APC inhibitor)(Anticardiolipin cofactor) [Source: UniProt P027
2515.1.1	-4.1	5.04	2629.1181	0.0078	4	31.85	lyyk	228	DKATFGCHDGYSLDGPEEIECTK	250	lgnw	C [234] 57.0215, C [248] 57.0215	ENSP00000205948	Beta-2-glycoprotein 1 Precursor (Beta-2-glycoprotein I)(Beta(2)GPI)(B2GPI)(Apolipoprotein H)(Apo-H)(Activated protein C-binding protein)(APC inhibitor)(Anticardiolipin cofactor) [Source: UniProt P027
2516.1.1	-10	4.81	2629.118	0.015	3	31.85	lyyk	228	DKATFGCHDGYSLDGPEEIECTK	250	lgnw	C [234] 57.0215, C [248] 57.0215	ENSP00000205948	Beta-2-glycoprotein 1 Precursor (Beta-2-glycoprotein I)(Beta(2)GPI)(B2GPI)(Apolipoprotein H)(Apo-H)(Activated protein C-binding protein)(APC inhibitor)(Anticardiolipin cofactor) [Source: UniProt P027
2521.1.1	-5.1	5.4	2629.1181	-0.0018	4	31.85	lyyk	228	DKATFGCHDGYSLDGPEEIECTK	250	lgnw	C [234] 57.0215, C [248] 57.0215	ENSP00000205948	Beta-2-glycoprotein 1 Precursor (Beta-2-glycoprotein I)(Beta(2)GPI)(B2GPI)(Apolipoprotein H)(Apo-H)(Activated protein C-binding protein)(APC inhibitor)(Anticardiolipin cofactor) [Source: UniProt P027
2522.1.1	-9.6	5.09	2629.1181	0.0043	3	31.85	lyyk	228	DKATFGCHDGYSLDGPEEIECTK	250	lgnw	C [234] 57.0215, C [248] 57.0215	ENSP00000205948	Beta-2-glycoprotein 1 Precursor (Beta-2-glycoprotein I)(Beta(2)GPI)(B2GPI)(Apolipoprotein H)(Apo-H)(Activated protein C-binding protein)(APC inhibitor)(Anticardiolipin cofactor) [Source: UniProt P027
2528.1.1	-2.6	5.72	2629.1181	-0.0015	4	31.85	lyyk	228	DKATFGCHDGYSLDGPEEIECTK	250	lgnw	C [234] 57.0215, C [248] 57.0215	ENSP00000205948	Beta-2-glycoprotein 1 Precursor (Beta-2-glycoprotein I)(Beta(2)GPI)(B2GPI)(Apolipoprotein H)(Apo-H)(Activated protein C-binding protein)(APC inhibitor)(Anticardiolipin cofactor) [Source: UniProt P027
2531.1.1	-4.4	4.59	1506.6793	-0.0013	2	29.18	revk	205	CPFPSRPDNGFVN	217	ypak	C [205] 57.0215	ENSP00000205948	Beta-2-glycoprotein 1 Precursor (Beta-2-glycoprotein I)(Beta(2)GPI)(B2GPI)(Apolipoprotein H)(Apo-H)(Activated protein C-binding protein)(APC inhibitor)(Anticardiolipin cofactor) [Source: UniProt P027
2569.1.1	-4.5	5.63	2214.0053	-0.0008	3	29.94	nkek	306	KCSYTEDAQCIDGTIEVPK	324	cfke	C [307] 57.0215, C [315] 57.0215	ENSP00000205948	Beta-2-glycoprotein 1 Precursor (Beta-2-glycoprotein I)(Beta(2)GPI)(B2GPI)(Apolipoprotein H)(Apo-H)(Activated protein C-binding protein)(APC inhibitor)(Anticardiolipin cofactor) [Source: UniProt P027
2571.1.1	-2.1	5.21	1965.9275	-0.0017	3	33.03	revk	205	CPFPSRPDNGFVNYPAK	221	ptly	C [205] 57.0215	ENSP00000205948	Beta-2-glycoprotein 1 Precursor (Beta-2-glycoprotein I)(Beta(2)GPI)(B2GPI)(Apolipoprotein H)(Apo-H)(Activated protein C-binding protein)(APC inhibitor)(Anticardiolipin cofactor) [Source: UniProt P027
2572.1.1	-1.7	5.32	1639.7719	0.0021	3	34.46	ekfk	288	NGMLHGDKVSFFCK	301	nkek	C [300] 57.0215	ENSP00000205948	Beta-2-glycoprotein 1 Precursor (Beta-2-glycoprotein I)(Beta(2)GPI)(B2GPI)(Apolipoprotein H)(Apo-H)(Activated protein C-binding protein)(APC inhibitor)(Anticardiolipin cofactor) [Source: UniProt P027

2576.1.1	-5.6	6.42	2214.0053	0.0009	3	29.94	nkek	306	KCSYTEDAQCIDGTIEVPK	324	cfke	C [307] 57.0215, C [315] 57.0215	ENSP00000205948	Beta-2-glycoprotein 1 Precursor (Beta-2-glycoprotein I)(Beta(2)GPI)(B2GPI)(Apolipoprotein H)(Apo-H)(Activated protein C-binding protein)(APC inhibitor)(Anticardiolipin cofactor) [Source: UniProt P027
2577.1.1	-2.1	5.31	1639.7719	0.0021	3	34.46	ekfk	288	NGMLHGDKVSFFCK	301	nkek	C [300] 57.0215	ENSP00000205948	Beta-2-glycoprotein 1 Precursor (Beta-2-glycoprotein I)(Beta(2)GPI)(B2GPI)(Apolipoprotein H)(Apo-H)(Activated protein C-binding protein)(APC inhibitor)(Anticardiolipin cofactor) [Source: UniProt P027
2582.1.1	-11.8	5.61	2214.0053	-0.0001	2	29.94	nkek	306	KCSYTEDAQCIDGTIEVPK	324	cfke	C [307] 57.0215, C [315] 57.0215	ENSP00000205948	Beta-2-glycoprotein 1 Precursor (Beta-2-glycoprotein I)(Beta(2)GPI)(B2GPI)(Apolipoprotein H)(Apo-H)(Activated protein C-binding protein)(APC inhibitor)(Anticardiolipin cofactor) [Source: UniProt P027
2589.1.1	-10.8	5.53	2214.0053	-0.0004	2	29.94	nkek	306	KCSYTEDAQCIDGTIEVPK	324	cfke	C [307] 57.0215, C [315] 57.0215	ENSP00000205948	Beta-2-glycoprotein 1 Precursor (Beta-2-glycoprotein I)(Beta(2)GPI)(B2GPI)(Apolipoprotein H)(Apo-H)(Activated protein C-binding protein)(APC inhibitor)(Anticardiolipin cofactor) [Source: UniProt P027
2594.1.1	-1.3	4.08	941.5489	0.0011	2	27.49	arqr	406	LCTPLLPK	413	yppt	C [407] 57.0215	ENSP00000380189	Properdin precursor (Factor P). Source: Uniprot/SWISSPROT P27918
2604.1.1	-9.2	4.84	1712.732	0.0015	2	27.13	fgch	236	DGYSLDGPEEIECTK	250	lgnw	C [248] 57.0215	ENSP00000205948	Beta-2-glycoprotein 1 Precursor (Beta-2-glycoprotein I)(Beta(2)GPI)(B2GPI)(Apolipoprotein H)(Apo-H)(Activated protein C-binding protein)(APC inhibitor)(Anticardiolipin cofactor) [Source: UniProt P027
2606.1.1	-3	4.51	941.5489	0.0015	2	27.49	arqr	406	LCTPLLPK	413	yppt	C [407] 57.0215	ENSP00000380189	Properdin precursor (Factor P). Source: Uniprot/SWISSPROT P27918
2611.1.1	-9.6	5.03	1712.732	0.0008	2	27.13	fgch	236	DGYSLDGPEEIECTK	250	lgnw	C [248] 57.0215	ENSP00000205948	Beta-2-glycoprotein 1 Precursor (Beta-2-glycoprotein I)(Beta(2)GPI)(B2GPI)(Apolipoprotein H)(Apo-H)(Activated protein C-binding protein)(APC inhibitor)(Anticardiolipin cofactor) [Source: UniProt P027
2614.1.1	-2.7	4.83	941.5489	0.0006	2	27.49	arqr	406	LCTPLLPK	413	yppt	C [407] 57.0215	ENSP00000380189	Properdin precursor (Factor P). Source: Uniprot/SWISSPROT P27918
2617.1.1	-11.3	5.04	1712.732	0.0008	2	27.13	fgch	236	DGYSLDGPEEIECTK	250	lgnw	C [248] 57.0215	ENSP00000205948	Beta-2-glycoprotein 1 Precursor (Beta-2-glycoprotein I)(Beta(2)GPI)(B2GPI)(Apolipoprotein H)(Apo-H)(Activated protein C-binding protein)(APC inhibitor)(Anticardiolipin cofactor) [Source: UniProt P027
2618.1.1	-11.6	5.01	2629.1181	0.0028	3	31.85	lyyk	228	DKATFGCHDGYSLDGPEEIECTK	250	lgnw	C [234] 57.0215, C [248] 57.0215	ENSP00000205948	Beta-2-glycoprotein 1 Precursor (Beta-2-glycoprotein I)(Beta(2)GPI)(B2GPI)(Apolipoprotein H)(Apo-H)(Activated protein C-binding protein)(APC inhibitor)(Anticardiolipin cofactor) [Source: UniProt P027
2622.1.1	-11.8	4.94	2629.1181	-0.0008	3	31.85	lyyk	228	DKATFGCHDGYSLDGPEEIECTK	250	lgnw	C [234] 57.0215, C [248] 57.0215	ENSP00000205948	Beta-2-glycoprotein 1 Precursor (Beta-2-glycoprotein I)(Beta(2)GPI)(B2GPI)(Apolipoprotein H)(Apo-H)(Activated protein C-binding protein)(APC inhibitor)(Anticardiolipin cofactor) [Source: UniProt P027
2623.1.1	-4.1	5.45	2629.1181	0.0012	4	31.85	lyyk	228	DKATFGCHDGYSLDGPEEIECTK	250	lgnw	C [234] 57.0215, C [248] 57.0215	ENSP00000205948	Beta-2-glycoprotein 1 Precursor (Beta-2-glycoprotein I)(Beta(2)GPI)(B2GPI)(Apolipoprotein H)(Apo-H)(Activated protein C-binding protein)(APC inhibitor)(Anticardiolipin cofactor) [Source: UniProt P027
2629.1.1	-5.9	5.37	2385.9962	0.0026	3	32.48	ykdk	230	ATFGCHDGYSLDGPEEIECTK	250	lgnw	C [234] 57.0215, C [248] 57.0215	ENSP00000205948	Beta-2-glycoprotein 1 Precursor (Beta-2-glycoprotein I)(Beta(2)GPI)(B2GPI)(Apolipoprotein H)(Apo-H)(Activated protein C-binding protein)(APC inhibitor)(Anticardiolipin cofactor) [Source: UniProt P027
2633.1.1	-6.7	4.65	2629.1181	0.0043	3	31.85	lyyk	228	DKATFGCHDGYSLDGPEEIECTK	250	lgnw	C [234] 57.0215, C [248] 57.0215	ENSP00000205948	Beta-2-glycoprotein 1 Precursor (Beta-2-glycoprotein I)(Beta(2)GPI)(B2GPI)(Apolipoprotein H)(Apo-H)(Activated protein C-binding protein)(APC inhibitor)(Anticardiolipin cofactor) [Source: UniProt P027
2636.1.1	-7.6	5.79	2385.9962	0.0011	3	32.48	ykdk	230	ATFGCHDGYSLDGPEEIECTK	250	lgnw	C [234] 57.0215, C [248] 57.0215	ENSP00000205948	Beta-2-glycoprotein 1 Precursor (Beta-2-glycoprotein I)(Beta(2)GPI)(B2GPI)(Apolipoprotein H)(Apo-H)(Activated protein C-binding protein)(APC inhibitor)(Anticardiolipin cofactor) [Source: UniProt P027
2643.1.1	-9.6	6.1	2385.9962	-0.0001	3	32.48	ykdk	230	ATFGCHDGYSLDGPEEIECTK	250	lgnw	C [234] 57.0215, C [248] 57.0215	ENSP00000205948	Beta-2-glycoprotein 1 Precursor (Beta-2-glycoprotein I)(Beta(2)GPI)(B2GPI)(Apolipoprotein H)(Apo-H)(Activated protein C-binding protein)(APC inhibitor)(Anticardiolipin cofactor) [Source: UniProt P027
2648.1.1	-9.6	5.17	2385.9962	0.0024	2	32.48	ykdk	230	ATFGCHDGYSLDGPEEIECTK	250	lgnw	C [234] 57.0215, C [248] 57.0215	ENSP00000205948	Beta-2-glycoprotein 1 Precursor (Beta-2-glycoprotein I)(Beta(2)GPI)(B2GPI)(Apolipoprotein H)(Apo-H)(Activated protein C-binding protein)(APC inhibitor)(Anticardiolipin cofactor) [Source: UniProt P027
2651.1.1	-9.3	5.33	2385.9962	0.0041	2	32.48	ykdk	230	ATFGCHDGYSLDGPEEIECTK	250	lgnw	C [234] 57.0215, C [248] 57.0215	ENSP00000205948	Beta-2-glycoprotein 1 Precursor (Beta-2-glycoprotein I)(Beta(2)GPI)(B2GPI)(Apolipoprotein H)(Apo-H)(Activated protein C-binding protein)(APC inhibitor)(Anticardiolipin cofactor) [Source: UniProt P027
2657.1.1	-11.5	5.65	2385.9962	0.0029	2	32.48	ykdk	230	ATFGCHDGYSLDGPEEIECTK	250	lgnw	C [234] 57.0215, C [248] 57.0215	ENSP00000205948	Beta-2-glycoprotein 1 Precursor (Beta-2-glycoprotein I)(Beta(2)GPI)(B2GPI)(Apolipoprotein H)(Apo-H)(Activated protein C-binding protein)(APC inhibitor)(Anticardiolipin cofactor) [Source: UniProt P027
2659.1.1	-9.9	4.84	2085.9103	0.0069	2	30.19	kekk	307	CSYTEDAQCIDGTIEVPK	324	cfke	C [307] 57.0215, C [315] 57.0215	ENSP00000205948	Beta-2-glycoprotein 1 Precursor (Beta-2-glycoprotein I)(Beta(2)GPI)(B2GPI)(Apolipoprotein H)(Apo-H)(Activated protein C-binding protein)(APC inhibitor)(Anticardiolipin cofactor) [Source: UniProt P027
2660.1.1	-2.2	5.4	1539.7413	0.0003	3	36.42	evpk	325	CFKEHSSLAFWK	336	tdas	C [325] 57.0215	ENSP00000205948	Beta-2-glycoprotein 1 Precursor (Beta-2-glycoprotein I)(Beta(2)GPI)(B2GPI)(Apolipoprotein H)(Apo-H)(Activated protein C-binding protein)(APC inhibitor)(Anticardiolipin cofactor) [Source: UniProt P027
2665.1.1	-6.2	5	2085.9103	0.003	2	30.19	kekk	307	CSYTEDAQCIDGTIEVPK	324	cfke	C [307] 57.0215, C [315] 57.0215	ENSP00000205948	Beta-2-glycoprotein 1 Precursor (Beta-2-glycoprotein I)(Beta(2)GPI)(B2GPI)(Apolipoprotein H)(Apo-H)(Activated protein C-binding protein)(APC inhibitor)(Anticardiolipin cofactor) [Source: UniProt P027
2670.1.1	-1.3	6.44	1539.7413	0.0021	3	36.42	evpk	325	CFKEHSSLAFWK	336	tdas	C [325] 57.0215	ENSP00000205948	Beta-2-glycoprotein 1 Precursor (Beta-2-glycoprotein I)(Beta(2)GPI)(B2GPI)(Apolipoprotein H)(Apo-H)(Activated protein C-binding protein)(APC inhibitor)(Anticardiolipin cofactor) [Source: UniProt P027
2672.1.1	-12.3	4.79	2085.9103	0.0055	2	30.19	kekk	307	CSYTEDAQCIDGTIEVPK	324	cfke	C [307] 57.0215, C [315] 57.0215	ENSP00000205948	Beta-2-glycoprotein 1 Precursor (Beta-2-glycoprotein I)(Beta(2)GPI)(B2GPI)(Apolipoprotein H)(Apo-H)(Activated protein C-binding protein)(APC inhibitor)(Anticardiolipin cofactor) [Source: UniProt P027
2674.1.1	-8.8	5.29	1539.7413	-0.002	2	36.42	evpk	325	CFKEHSSLAFWK	336	tdas	C [325] 57.0215	ENSP00000205948	Beta-2-glycoprotein 1 Precursor (Beta-2-glycoprotein I)(Beta(2)GPI)(B2GPI)(Apolipoprotein H)(Apo-H)(Activated protein C-binding protein)(APC inhibitor)(Anticardiolipin cofactor) [Source: UniProt P027
2678.1.1	-1.5	5.66	1915.9201	0.0013	4	37.25	iqek	286	FKNGMLHGDKVSFFCK	301	nkek	C [300] 57.0215, N [288] 0.9848	ENSP00000205948	Beta-2-glycoprotein 1 Precursor (Beta-2-glycoprotein I)(Beta(2)GPI)(B2GPI)(Apolipoprotein H)(Apo-H)(Activated protein C-binding protein)(APC inhibitor)(Anticardiolipin cofactor) [Source: UniProt P027
2679.1.1	-5.5	5.42	1539.7413	0.0014	2	36.42	evpk	325	CFKEHSSLAFWK	336	tdas	C [325] 57.0215	ENSP00000205948	Beta-2-glycoprotein 1 Precursor (Beta-2-glycoprotein I)(Beta(2)GPI)(B2GPI)(Apolipoprotein H)(Apo-H)(Activated protein C-binding protein)(APC inhibitor)(Anticardiolipin cofactor) [Source: UniProt P027
2681.1.1	-1.8	4.63	2214.9901	-0.0074	3	29.94	nkek	306	KCSYTEDAQCIDGTIEVPK	324	cfke	C [307] 57.0215, C [315] 57.0215,	ENSP00000205948	Beta-2-glycoprotein 1 Precursor (Beta-2-glycoprotein I)(Beta(2)GPI)(B2GPI)(Apolipoprotein H)(Apo-H)(Activated protein C-binding protein)(APC inhibitor)(Anticardiolipin cofactor) [Source: UniProt P027
2685.1.1	-6	5.29	1539.7413	-0.0066	2	36.42	evpk	325	CFKEHSSLAFWK	336	tdas	C [325] 57.0215	ENSP00000205948	Beta-2-glycoprotein 1 Precursor (Beta-2-glycoprotein I)(Beta(2)GPI)(B2GPI)(Apolipoprotein H)(Apo-H)(Activated protein C-binding protein)(APC inhibitor)(Anticardiolipin cofactor) [Source: UniProt P027
2689.1.1	-22.1	5.78	1723.7521	0.0018	2	30.37	vplk	39	TFYEPGEEITYSCK	52	pgyv	C [51] 57.0215	ENSP00000205948	Beta-2-glycoprotein 1 Precursor (Beta-2-glycoprotein I)(Beta(2)GPI)(B2GPI)(Apolipoprotein H)(Apo-H)(Activated protein C-binding protein)(APC inhibitor)(Anticardiolipin cofactor) [Source: UniProt P027
2695.1.1	-9.3	5.74	1723.7521	0.003	2	30.37	vplk	39	TFYEPGEEITYSCK	52	pgyv	C [51] 57.0215	ENSP00000205948	Beta-2-glycoprotein 1 Precursor (Beta-2-glycoprotein I)(Beta(2)GPI)(B2GPI)(Apolipoprotein H)(Apo-H)(Activated protein C-binding protein)(APC inhibitor)(Anticardiolipin cofactor) [Source: UniProt P027
2708.1.1	-1.3	5.43	1104.5473	0.0024	3	30.81	kcfk	328	EHSSLAFWK	336	tdas		ENSP00000205948	Beta-2-glycoprotein 1 Precursor (Beta-2-glycoprotein I)(Beta(2)GPI)(B2GPI)(Apolipoprotein H)(Apo-H)(Activated protein C-binding protein)(APC inhibitor)(Anticardiolipin cofactor) [Source: UniProt P027
2711.1.1	-2.2	5.92	1104.5473	0.0015	3	30.81	kcfk	328	EHSSLAFWK	336	tdas		ENSP00000205948	Beta-2-glycoprotein 1 Precursor (Beta-2-glycoprotein I)(Beta(2)GPI)(B2GPI)(Apolipoprotein H)(Apo-H)(Activated protein C-binding protein)(APC inhibitor)(Anticardiolipin cofactor) [Source: UniProt P027
2715.1.1	-1.4	6.23	1104.5473	0.0014	3	30.81	kcfk	328	EHSSLAFWK	336	tdas		ENSP00000205948	Beta-2-glycoprotein 1 Precursor (Beta-2-glycoprotein I)(Beta(2)GPI)(B2GPI)(Apolipoprotein H)(Apo-H)(Activated protein C-binding protein)(APC inhibitor)(Anticardiolipin cofactor) [Source: UniProt P027
2730.1.1	-3.2	5.06	2974.4556	0.0072	3	41.61	revk	205	CPFPSRPDNGFVNYPAKPTLYYKDI	229	atfg	C [205] 57.0215	ENSP00000205948	Beta-2-glycoprotein 1 Precursor (Beta-2-glycoprotein I)(Beta(2)GPI)(B2GPI)(Apolipoprotein H)(Apo-H)(Activated protein C-binding protein)(APC inhibitor)(Anticardiolipin cofactor) [Source: UniProt P027
2736.1.1	-3.1	5.07	2974.4556	0.0055	3	41.61	revk	205	CPFPSRPDNGFVNYPAKPTLYYKDI	229	atfg	C [205] 57.0215	ENSP00000205948	Beta-2-glycoprotein 1 Precursor (Beta-2-glycoprotein I)(Beta(2)GPI)(B2GPI)(Apolipoprotein H)(Apo-H)(Activated protein C-binding protein)(APC inhibitor)(Anticardiolipin cofactor) [Source: UniProt P027
2739.1.1	-4.4	4.44	1660.8734	-0.0008	2	35.76	rpdn	214	GFVNYPAKPTLYYK	227	dkat		ENSP00000205948	Beta-2-glycoprotein 1 Precursor (Beta-2-glycoprotein I)(Beta(2)GPI)(B2GPI)(Apolipoprotein H)(Apo-H)(Activated protein C-binding protein)(APC inhibitor)(Anticardiolipin cofactor) [Source: UniProt P027
2743.1.1	-2.1	4.78	2974.4556	0.0046	3	41.61	revk	205	CPFPSRPDNGFVNYPAKPTLYYKDI	229	atfg	C [205] 57.0215	ENSP00000205948	Beta-2-glycoprotein 1 Precursor (Beta-2-glycoprotein I)(Beta(2)GPI)(B2GPI)(Apolipoprotein H)(Apo-H)(Activated protein C-binding protein)(APC inhibitor)(Anticardiolipin cofactor) [Source: UniProt P027
2752.1.1	-3.7	5.21	1925.8798	0.0038	2	30.14	ekkc	308	SYTEDAQCIDGTIEVPK	324	cfke	C [315] 57.0215	ENSP00000205948	Beta-2-glycoprotein 1 Precursor (Beta-2-glycoprotein I)(Beta(2)GPI)(B2GPI)(Apolipoprotein H)(Apo-H)(Activated protein C-binding protein)(APC inhibitor)(Anticardiolipin cofactor) [Source: UniProt P027
2756.1.1	-7	5.19	1925.8798	0.004	2	30.14	ekkc	308	SYTEDAQCIDGTIEVPK	324	cfke	C [315] 57.0215	ENSP00000205948	Beta-2-glycoprotein 1 Precursor (Beta-2-glycoprotein I)(Beta(2)GPI)(B2GPI)(Apolipoprotein H)(Apo-H)(Activated protein C-binding protein)(APC inhibitor)(Anticardiolipin cofactor) [Source: UniProt P027
2762.1.1	-7	5.08	1925.8798	0.0031	2	30.14	ekkc	308	SYTEDAQCIDGTIEVPK	324	cfke	C [315] 57.0215	ENSP00000205948	Beta-2-glycoprotein 1 Precursor (Beta-2-glycoprotein I)(Beta(2)GPI)(B2GPI)(Apolipoprotein H)(Apo-H)(Activated protein C-binding protein)(APC inhibitor)(Anticardiolipin cofactor) [Source: UniProt P027
2776.1.1	-4.7	4.73	2528.1221	-0.0018	3	39.49	evpk	325	CFKEHSSLAFWKTDASDVKPC	345	]	C [325] 57.0215, C [345] 57.0215,	ENSP00000205948	Beta-2-glycoprotein 1 Precursor (Beta-2-glycoprotein I)(Beta(2)GPI)(B2GPI)(Apolipoprotein H)(Apo-H)(Activated protein C-binding protein)(APC inhibitor)(Anticardiolipin cofactor) [Source: UniProt P027
2827.1.1	-3.3	5.28	1640.7567	-0.0012	3	34.46	ekfk	288	NGMLHGDKVSFFCK	301	nkek	C [300] 57.0215, N [288] 0.9848	ENSP00000205948	Beta-2-glycoprotein 1 Precursor (Beta-2-glycoprotein I)(Beta(2)GPI)(B2GPI)(Apolipoprotein H)(Apo-H)(Activated protein C-binding protein)(APC inhibitor)(Anticardiolipin cofactor) [Source: UniProt P027
2828.1.1	-6.9	5.27	2085.9103	-0.0029	3	30.19	kekk	307	CSYTEDAQCIDGTIEVPK	324	cfke	C [307] 57.0215, C [315] 57.0215	ENSP00000205948	Beta-2-glycoprotein 1 Precursor (Beta-2-glycoprotein I)(Beta(2)GPI)(B2GPI)(Apolipoprotein H)(Apo-H)(Activated protein C-binding protein)(APC inhibitor)(Anticardiolipin cofactor) [Source: UniProt P027
2832.1.1	-6.7	5.36	2085.9103	-0.0031	3	30.19	kekk	307	CSYTEDAQCIDGTIEVPK	324	cfke	C [307] 57.0215, C [315] 57.0215	ENSP00000205948	Beta-2-glycoprotein 1 Precursor (Beta-2-glycoprotein I)(Beta(2)GPI)(B2GPI)(Apolipoprotein H)(Apo-H)(Activated protein C-binding protein)(APC inhibitor)(Anticardiolipin cofactor) [Source: UniProt P027
2835.1.1	-1.4	5.06	1640.7567	-0.0008	3	34.46	ekfk	288	NGMLHGDKVSFFCK	301	nkek	C [300] 57.0215, N [288] 0.9848	ENSP00000205948	Beta-2-glycoprotein 1 Precursor (Beta-2-glycoprotein I)(Beta(2)GPI)(B2GPI)(Apolipoprotein H)(Apo-H)(Activated protein C-binding protein)(APC inhibitor)(Anticardiolipin cofactor) [Source: UniProt P027
2838.1.1	-6	5.55	2085.9103	-0.0002	3	30.19	kekk	307	CSYTEDAQCIDGTIEVPK	324	cfke	C [307] 57.0215, C [315] 57.0215	ENSP00000205948	Beta-2-glycoprotein 1 Precursor (Beta-2-glycoprotein I)(Beta(2)GPI)(B2GPI)(Apolipoprotein H)(Apo-H)(Activated protein C-binding protein)(APC inhibitor)(Anticardiolipin cofactor) [Source: UniProt P027
2841.1.1	-4.2	4.91	1640.7567	-0.0012	3	34.46	ekfk	288	NGMLHGDKVSFFCK	301	nkek	C [300] 57.0215, N [288] 0.9848	ENSP00000205948	Beta-2-glycoprotein 1 Precursor (Beta-2-glycoprotein I)(Beta(2)GPI)(B2GPI)(Apolipoprotein H)(Apo-H)(Activated protein C-binding protein)(APC inhibitor)(Anticardiolipin cofactor) [Source: UniProt P027
2874.1.1	-7.4	5.08	1336.6379	0.002	2	25.27	iesk	9	TAFQEALDAAGDK	21	lvvv		ENSP00000363641	Thioredoxin (Trx)(ATL-derived factor)(ADF)(Surface-associated sulphydryl protein)(SASP) [Source: UniProt P10599]
2875.1.1	-9.4	4.54	2085.9103	0.0045	2	30.19	kekk	307	CSYTEDAQCIDGTIEVPK	324	cfke	C [307] 57.0215, C [315] 57.0215	ENSP00000205948	Beta-2-glycoprotein 1 Precursor (Beta-2-glycoprotein I)(Beta(2)GPI)(B2GPI)(Apolipoprotein H)(Apo-H)(Activated protein C-binding protein)(APC inhibitor)(Anticardiolipin cofactor) [Source: UniProt P027
2878.1.1	-6.6	5.08	1336.6379	-0.0008	2	25.27	iesk	9	TAFQEALDAAGDK	21	lvvv		ENSP00000363641	Thioredoxin (Trx)(ATL-derived factor)(ADF)(Surface-associated sulphydryl protein)(SASP) [Source: UniProt P10599]
2884.1.1	-5.1	4.86	1336.6379	-0.0017	2	25.27	iesk	9	TAFQEALDAAGDK	21	lvvv		ENSP00000363641	Thioredoxin (Trx)(ATL-derived factor)(ADF)(Surface-associated sulphydryl protein)(SASP) [Source: UniProt P10599]
2904.1.1	-1.7	5.65	2731.3337	-0.0003	4	41.86	revk	205	CPFPSRPDNGFVNYPAKPTLYYK	227	dkat	C [205] 57.0215	ENSP00000205948	Beta-2-glycoprotein 1 Precursor (Beta-2-glycoprotein I)(Beta(2)GPI)(B2GPI)(Apolipoprotein H)(Apo-H)(Activated protein C-binding protein)(APC inhibitor)(Anticardiolipin cofactor) [Source: UniProt P027
2906.1.1	-7.1	5.07	2731.3337	-0.0007	3	41.86	revk	205	CPFPSRPDNGFVNYPAKPTLYYK	227	dkat	C [205] 57.0215	ENSP00000205948	Beta-2-glycoprotein 1 Precursor (Beta-2-glycoprotein I)(Beta(2)GPI)(B2GPI)(Apolipoprotein H)(Apo-H)(Activated protein C-binding protein)(APC inhibitor)(Anticardiolipin cofactor) [Source: UniProt P027
2911.1.1	-1.3	6.39	2731.3337	-0.0023	4	41.86	revk	205	CPFPSRPDNGFVNYPAKPTLYYK	227	dkat	C [205] 57.0215	ENSP00000205948	Beta-2-glycoprotein 1 Precursor (Beta-2-glycoprotein I)(Beta(2)GPI)(B2GPI)(Apolipoprotein H)(Apo-H)(Activated protein C-binding protein)(APC inhibitor)(Anticardiolipin cofactor) [Source: UniProt P027
2912.1.1	-9	5.79	2731.3337	0.0035	3	41.86	revk	205	CPFPSRPDNGFVNYPAKPTLYYK	227	dkat	C [205] 57.0215	ENSP00000205948	Beta-2-glycoprotein 1 Precursor (Beta-2-glycoprotein I)(Beta(2)GPI)(B2GPI)(Apolipoprotein H)(Apo-H)(Activated protein C-binding protein)(APC inhibitor)(Anticardiolipin cofactor) [Source: UniProt P027
2918.1.1	-1.3	6.9	2731.3337	-0.0001	4	41.86	revk	205	CPFPSRPDNGFVNYPAKPTLYYK	227	dkat	C [205] 57.0215	ENSP00000205948	Beta-2-glycoprotein 1 Precursor (Beta-2-glycoprotein I)(Beta(2)GPI)(B2GPI)(Apolipoprotein H)(Apo-H)(Activated protein C-binding protein)(APC inhibitor)(Anticardiolipin cofactor) [Source: UniProt P027
2928.1.1	-7.7	4.56	2086.8951	-0.0003	2	30.19	kekk	307	CSYTEDAQCIDGTIEVPK	324	cfke	C [307] 57.0215, C [315] 57.0215,	ENSP00000205948	Beta-2-glycoprotein 1 Precursor (Beta-2-glycoprotein I)(Beta(2)GPI)(B2GPI)(Apolipoprotein H)(Apo-H)(Activated protein C-binding protein)(APC inhibitor)(Anticardiolipin cofactor) [Source: UniProt P027
2933.1.1	-4.3	5.22	2425.1017	-0.0013	2	36.69	vplk	39	TFYEPGEEITYSCKPGYVSR	58	ggmr	C [51] 57.0215, T [39] 42.0106	ENSP00000205948	Beta-2-glycoprotein 1 Precursor (Beta-2-glycoprotein I)(Beta(2)GPI)(B2GPI)(Apolipoprotein H)(Apo-H)(Activated protein C-binding protein)(APC inhibitor)(Anticardiolipin cofactor) [Source: UniProt P027
2937.1.1	-6.4	5.25	2425.1017	0.0053	2	36.69	vplk	39	TFYEPGEEITYSCKPGYVSR	58	ggmr	C [51] 57.0215, T [39] 42.0106	ENSP00000205948	Beta-2-glycoprotein 1 Precursor (Beta-2-glycoprotein I)(Beta(2)GPI)(B2GPI)(Apolipoprotein H)(Apo-H)(Activated protein C-binding protein)(APC inhibitor)(Anticardiolipin cofactor) [Source: UniProt P027
2938.1.1	-6.6	4.77	2086.8951	0.0019	2	30.19	kekk	307	CSYTEDAQCIDGTIEVPK	324	cfke	C [307] 57.0215, C [315] 57.0215,	ENSP00000205948	Beta-2-glycoprotein 1 Precursor (Beta-2-glycoprotein I)(Beta(2)GPI)(B2GPI)(Apolipoprotein H)(Apo-H)(Activated protein C-binding protein)(APC inhibitor)(Anticardiolipin cofactor) [Source: UniProt P027
2942.1.1	-6.5	4.85	2086.8951	0.0068	2	30.19	kekk	307	CSYTEDAQCIDGTIEVPK	324	cfke	C [307] 57.0215, C [315] 57.0215,	ENSP00000205948	Beta-2-glycoprotein 1 Precursor (Beta-2-glycoprotein I)(Beta(2)GPI)(B2GPI)(Apolipoprotein H)(Apo-H)(Activated protein C-binding protein)(APC inhibitor)(Anticardiolipin cofactor) [Source: UniProt P027
2946.1.1	-5.2	4.83	2425.102	0.011	2	36.69	vplk	39	TFYEPGEEITYSCKPGYVSR	58	ggmr	C [51] 57.0215, T [39] 42.0106	ENSP00000205948	Beta-2-glycoprotein 1 Precursor (Beta-2-glycoprotein I)(Beta(2)GPI)(B2GPI)(Apolipoprotein H)(Apo-H)(Activated protein C-binding protein)(APC inhibitor)(Anticardiolipin cofactor) [Source: UniProt P027
2961.1.1	-2.4	5.49	2425.1017	0.0035	3	36.69	vplk	39	TFYEPGEEITYSCKPGYVSR	58	ggmr	C [51] 57.0215, T [39] 42.0106	ENSP00000205948	Beta-2-glycoprotein 1 Precursor (Beta-2-glycoprotein I)(Beta(2)GPI)(B2GPI)(Apolipoprotein H)(Apo-H)(Activated protein C-binding protein)(APC inhibitor)(Anticardiolipin cofactor) [Source: UniProt P027
2970.1.1	-5.4	4.08	1765.7626	0.0038	2	30.37	vplk	39	TFYEPGEEITYSCK	52	pgyv	C [51] 57.0215, T [39] 42.0106	ENSP00000205948	Beta-2-glycoprotein 1 Precursor (Beta-2-glycoprotein I)(Beta(2)GPI)(B2GPI)(Apolipoprotein H)(Apo-H)(Activated protein C-binding protein)(APC inhibitor)(Anticardiolipin cofactor) [Source: UniProt P027
2976.1.1	-8	4.96	1243.6794	-0.0002	2	31.31	prvc	85	PFAGILENGAVR	96	yttf		ENSP00000205948	Beta-2-glycoprotein 1 Precursor (Beta-2-glycoprotein I)(Beta(2)GPI)(B2GPI)(Apolipoprotein H)(Apo-H)(Activated protein C-binding protein)(APC inhibitor)(Anticardiolipin cofactor) [Source: UniProt P027
2981.1.1	-6.4	5.2	1243.6794	-0.0002	2	31.31	prvc	85	PFAGILENGAVR	96	yttf		ENSP00000205948	Beta-2-glycoprotein 1 Precursor (Beta-2-glycoprotein I)(Beta(2)GPI)(B2GPI)(Apolipoprotein H)(Apo-H)(Activated protein C-binding protein)(APC inhibitor)(Anticardiolipin cofactor) [Source: UniProt P027
2986.1.1	-7.2	5.23	1243.6794	0.0005	2	31.31	prvc	85	PFAGILENGAVR	96	yttf		ENSP00000205948	Beta-2-glycoprotein 1 Precursor (Beta-2-glycoprotein I)(Beta(2)GPI)(B2GPI)(Apolipoprotein H)(Apo-H)(Activated protein C-binding protein)(APC inhibitor)(Anticardiolipin cofactor) [Source: UniProt P027
3004.1.1	-1.6	5	2127.938	-0.018	2	30.16	rkrk	411	YFHHDRLDWDEHSSAGR	427	yvsr		ENSP00000326830	
3008.1.1	-2	6.3	2732.3185	-0.0042	4	41.86	revk	205	CPFPSRPDNGFVNYPAKPTLYYK	227	dkat	C [205] 57.0215, N [213] 0.9848	ENSP00000205948	Beta-2-glycoprotein 1 Precursor (Beta-2-glycoprotein I)(Beta(2)GPI)(B2GPI)(Apolipoprotein H)(Apo-H)(Activated protein C-binding protein)(APC inhibitor)(Anticardiolipin cofactor) [Source: UniProt P027
3010.1.1	-6.5	5.34	2732.3185	0.0009	3	41.86	revk	205	CPFPSRPDNGFVNYPAKPTLYYK	227	dkat	C [205] 57.0215, N [213] 0.9848	ENSP00000205948	Beta-2-glycoprotein 1 Precursor (Beta-2-glycoprotein I)(Beta(2)GPI)(B2GPI)(Apolipoprotein H)(Apo-H)(Activated protein C-binding protein)(APC inhibitor)(Anticardiolipin cofactor) [Source: UniProt P027
3015.1.1	-1.5	6.08	2732.3185	-0.0044	4	41.86	revk	205	CPFPSRPDNGFVNYPAKPTLYYK	227	dkat	C [205] 57.0215, N [217] 0.9848	ENSP00000205948	Beta-2-glycoprotein 1 Precursor (Beta-2-glycoprotein I)(Beta(2)GPI)(B2GPI)(Apolipoprotein H)(Apo-H)(Activated protein C-binding protein)(APC inhibitor)(Anticardiolipin cofactor) [Source: UniProt P027
3017.1.1	-7.7	5.16	2732.3185	0.0051	3	41.86	revk	205	CPFPSRPDNGFVNYPAKPTLYYK	227	dkat	C [205] 57.0215, N [213] 0.9848	ENSP00000205948	Beta-2-glycoprotein 1 Precursor (Beta-2-glycoprotein I)(Beta(2)GPI)(B2GPI)(Apolipoprotein H)(Apo-H)(Activated protein C-binding protein)(APC inhibitor)(Anticardiolipin cofactor) [Source: UniProt P027
3022.1.1	-2	6	2732.3185	-0.0037	4	41.86	revk	205	CPFPSRPDNGFVNYPAKPTLYYK	227	dkat	C [205] 57.0215, N [217] 0.9848	ENSP00000205948	Beta-2-glycoprotein 1 Precursor (Beta-2-glycoprotein I)(Beta(2)GPI)(B2GPI)(Apolipoprotein H)(Apo-H)(Activated protein C-binding protein)(APC inhibitor)(Anticardiolipin cofactor) [Source: UniProt P027
3025.1.1	-7.6	5.11	2732.3185	0.0027	3	41.86	revk	205	CPFPSRPDNGFVNYPAKPTLYYK	227	dkat	C [205] 57.0215, N [213] 0.9848	ENSP00000205948	Beta-2-glycoprotein 1 Precursor (Beta-2-glycoprotein I)(Beta(2)GPI)(B2GPI)(Apolipoprotein H)(Apo-H)(Activated protein C-binding protein)(APC inhibitor)(Anticardiolipin cofactor) [Source: UniProt P027
3053.1.1	-5.8	4.52	1948.901	0.011	2	33.03	revk	205	CPFPSRPDNGFVNYPAK	221	ptly	C [205] 57.0215, C [205] -17.0265	ENSP00000205948	Beta-2-glycoprotein 1 Precursor (Beta-2-glycoprotein I)(Beta(2)GPI)(B2GPI)(Apolipoprotein H)(Apo-H)(Activated protein C-binding protein)(APC inhibitor)(Anticardiolipin cofactor) [Source: UniProt P027
3058.1.1	-5.1	5.47	2957.4291	-0.0056	4	41.61	revk	205	CPFPSRPDNGFVNYPAKPTLYYKDI	229	atfg	C [205] 57.0215, C [205] -17.0265	ENSP00000205948	Beta-2-glycoprotein 1 Precursor (Beta-2-glycoprotein I)(Beta(2)GPI)(B2GPI)(Apolipoprotein H)(Apo-H)(Activated protein C-binding protein)(APC inhibitor)(Anticardiolipin cofactor) [Source: UniProt P027
3060.1.1	-1.5	4.67	2383.0911	-0.0035	3	36.69	vplk	39	TFYEPGEEITYSCKPGYVSR	58	ggmr	C [51] 57.0215	ENSP00000205948	Beta-2-glycoprotein 1 Precursor (Beta-2-glycoprotein I)(Beta(2)GPI)(B2GPI)(Apolipoprotein H)(Apo-H)(Activated protein C-binding protein)(APC inhibitor)(Anticardiolipin cofactor) [Source: UniProt P027
3061.1.1	-8.1	4.7	1948.9009	0.0006	2	33.03	revk	205	CPFPSRPDNGFVNYPAK	221	ptly	C [205] 57.0215, C [205] -17.0265	ENSP00000205948	Beta-2-glycoprotein 1 Precursor (Beta-2-glycoprotein I)(Beta(2)GPI)(B2GPI)(Apolipoprotein H)(Apo-H)(Activated protein C-binding protein)(APC inhibitor)(Anticardiolipin cofactor) [Source: UniProt P027
3062.1.1	-5.2	4.66	1435.6265	-0.0024	2	31.46	vplk	39	TFYEPGEEITYS	50	ckpg		ENSP00000205948	Beta-2-glycoprotein 1 Precursor (Beta-2-glycoprotein I)(Beta(2)GPI)(B2GPI)(Apolipoprotein H)(Apo-H)(Activated protein C-binding protein)(APC inhibitor)(Anticardiolipin cofactor) [Source: UniProt P027
3064.1.1	-3.5	5.94	2957.4291	0.0005	4	41.61	revk	205	CPFPSRPDNGFVNYPAKPTLYYKDI	229	atfg	C [205] 57.0215, C [205] -17.0265	ENSP00000205948	Beta-2-glycoprotein 1 Precursor (Beta-2-glycoprotein I)(Beta(2)GPI)(B2GPI)(Apolipoprotein H)(Apo-H)(Activated protein C-binding protein)(APC inhibitor)(Anticardiolipin cofactor) [Source: UniProt P027
3066.1.1	-7	5.23	2957.429	0.014	3	41.61	revk	205	CPFPSRPDNGFVNYPAKPTLYYKDI	229	atfg	C [205] 57.0215, C [205] -17.0265	ENSP00000205948	Beta-2-glycoprotein 1 Precursor (Beta-2-glycoprotein I)(Beta(2)GPI)(B2GPI)(Apolipoprotein H)(Apo-H)(Activated protein C-binding protein)(APC inhibitor)(Anticardiolipin cofactor) [Source: UniProt P027
3068.1.1	-8.1	4.77	1948.9009	0.003	2	33.03	revk	205	CPFPSRPDNGFVNYPAK	221	ptly	C [205] 57.0215, C [205] -17.0265	ENSP00000205948	Beta-2-glycoprotein 1 Precursor (Beta-2-glycoprotein I)(Beta(2)GPI)(B2GPI)(Apolipoprotein H)(Apo-H)(Activated protein C-binding protein)(APC inhibitor)(Anticardiolipin cofactor) [Source: UniProt P027
3069.1.1	-14.8	4.79	2068.8838	0.0047	2	30.19	kekk	307	CSYTEDAQCIDGTIEVPK	324	cfke	C [307] 57.0215, C [315] 57.0215,	ENSP00000205948	Beta-2-glycoprotein 1 Precursor (Beta-2-glycoprotein I)(Beta(2)GPI)(B2GPI)(Apolipoprotein H)(Apo-H)(Activated protein C-binding protein)(APC inhibitor)(Anticardiolipin cofactor) [Source: UniProt P027
3071.1.1	-3.9	6.26	2957.4291	-0.0032	4	41.61	revk	205	CPFPSRPDNGFVNYPAKPTLYYKDI	229	atfg	C [205] 57.0215, C [205] -17.0265	ENSP00000205948	Beta-2-glycoprotein 1 Precursor (Beta-2-glycoprotein I)(Beta(2)GPI)(B2GPI)(Apolipoprotein H)(Apo-H)(Activated protein C-binding protein)(APC inhibitor)(Anticardiolipin cofactor) [Source: UniProt P027
3072.1.1	-7	5.45	2957.4291	0.0022	3	41.61	revk	205	CPFPSRPDNGFVNYPAKPTLYYKDI	229	atfg	C [205] 57.0215, C [205] -17.0265	ENSP00000205948	Beta-2-glycoprotein 1 Precursor (Beta-2-glycoprotein I)(Beta(2)GPI)(B2GPI)(Apolipoprotein H)(Apo-H)(Activated protein C-binding protein)(APC inhibitor)(Anticardiolipin cofactor) [Source: UniProt P027
3073.1.1	-11.8	4.83	2068.8838	0	2	30.19	kekk	307	CSYTEDAQCIDGTIEVPK	324	cfke	C [307] 57.0215, C [315] 57.0215,	ENSP00000205948	Beta-2-glycoprotein 1 Precursor (Beta-2-glycoprotein I)(Beta(2)GPI)(B2GPI)(Apolipoprotein H)(Apo-H)(Activated protein C-binding protein)(APC inhibitor)(Anticardiolipin cofactor) [Source: UniProt P027
3078.1.1	-6.9	5.6	2957.4291	0.0071	3	41.61	revk	205	CPFPSRPDNGFVNYPAKPTLYYKDI	229	atfg	C [205] 57.0215, C [205] -17.0265	ENSP00000205948	Beta-2-glycoprotein 1 Precursor (Beta-2-glycoprotein I)(Beta(2)GPI)(B2GPI)(Apolipoprotein H)(Apo-H)(Activated protein C-binding protein)(APC inhibitor)(Anticardiolipin cofactor) [Source: UniProt P027
3079.1.1	-11.4	4.91	2068.8838	0.0037	2	30.19	kekk	307	CSYTEDAQCIDGTIEVPK	324	cfke	C [307] 57.0215, C [315] 57.0215,	ENSP00000205948	Beta-2-glycoprotein 1 Precursor (Beta-2-glycoprotein I)(Beta(2)GPI)(B2GPI)(Apolipoprotein H)(Apo-H)(Activated protein C-binding protein)(APC inhibitor)(Anticardiolipin cofactor) [Source: UniProt P027
3082.1.1	-7.2	5.09	2070.105	0.0007	3	40.03	aiag	21	RTCPKPDDLPFSTVVPLK	38	tfye	C [23] 57.0215	ENSP00000205948	Beta-2-glycoprotein 1 Precursor (Beta-2-glycoprotein I)(Beta(2)GPI)(B2GPI)(Apolipoprotein H)(Apo-H)(Activated protein C-binding protein)(APC inhibitor)(Anticardiolipin cofactor) [Source: UniProt P027
3085.1.1	-7	5.28	2070.105	0.0007	3	40.03	aiag	21	RTCPKPDDLPFSTVVPLK	38	tfye	C [23] 57.0215	ENSP00000205948	Beta-2-glycoprotein 1 Precursor (Beta-2-glycoprotein I)(Beta(2)GPI)(B2GPI)(Apolipoprotein H)(Apo-H)(Activated protein C-binding protein)(APC inhibitor)(Anticardiolipin cofactor) [Source: UniProt P027
3092.1.1	-7.6	5.67	2070.105	-0.0041	3	40.03	aiag	21	RTCPKPDDLPFSTVVPLK	38	tfye	C [23] 57.0215	ENSP00000205948	Beta-2-glycoprotein 1 Precursor (Beta-2-glycoprotein I)(Beta(2)GPI)(B2GPI)(Apolipoprotein H)(Apo-H)(Activated protein C-binding protein)(APC inhibitor)(Anticardiolipin cofactor) [Source: UniProt P027
3098.1.1	-3.3	6.65	2127.1265	0.0023	4	39.23	vaia	20	GRTCPKPDDLPFSTVVPLK	38	tfye	C [23] 57.0215	ENSP00000205948	Beta-2-glycoprotein 1 Precursor (Beta-2-glycoprotein I)(Beta(2)GPI)(B2GPI)(Apolipoprotein H)(Apo-H)(Activated protein C-binding protein)(APC inhibitor)(Anticardiolipin cofactor) [Source: UniProt P027
3112.1.1	-2.2	6.6	2127.1265	0.0014	4	39.23	vaia	20	GRTCPKPDDLPFSTVVPLK	38	tfye	C [23] 57.0215	ENSP00000205948	Beta-2-glycoprotein 1 Precursor (Beta-2-glycoprotein I)(Beta(2)GPI)(B2GPI)(Apolipoprotein H)(Apo-H)(Activated protein C-binding protein)(APC inhibitor)(Anticardiolipin cofactor) [Source: UniProt P027
3117.1.1	-3.9	4.73	2127.1265	0.0003	2	39.23	vaia	20	GRTCPKPDDLPFSTVVPLK	38	tfye	C [23] 57.0215	ENSP00000205948	Beta-2-glycoprotein 1 Precursor (Beta-2-glycoprotein I)(Beta(2)GPI)(B2GPI)(Apolipoprotein H)(Apo-H)(Activated protein C-binding protein)(APC inhibitor)(Anticardiolipin cofactor) [Source: UniProt P027

3119.1.1	-7.3	6.18	2127.1265	-0.004	3	39.23	vaia	20	GRTCPKPDDLPFSTVVPLK	38	tfye	C [23] 57.0215	ENSP00000205948	Beta-2-glycoprotein 1 Precursor (Beta-2-glycoprotein I)(Beta(2)GPI)(B2GPI)(Apolipoprotein H)(Apo-H)(Activated protein C-binding protein)(APC inhibitor)(Anticardiolipin cofactor) [Source: UniProt P027
3125.1.1	-8.5	6.04	2127.1265	-0.0001	3	39.23	vaia	20	GRTCPKPDDLPFSTVVPLK	38	tfye	C [23] 57.0215	ENSP00000205948	Beta-2-glycoprotein 1 Precursor (Beta-2-glycoprotein I)(Beta(2)GPI)(B2GPI)(Apolipoprotein H)(Apo-H)(Activated protein C-binding protein)(APC inhibitor)(Anticardiolipin cofactor) [Source: UniProt P027
3129.1.1	-7.9	4.97	2211.104	-0.0048	3	39.26	iqvr	58	LGEHNIDVLEGNEQFINAAK	77	iith		sp|TRYP_PIG|	Trypsin; EC 3.4.21.4; Flags: Precursor;
3132.1.1	-8.4	6.04	2127.1265	-0.0053	3	39.23	vaia	20	GRTCPKPDDLPFSTVVPLK	38	tfye	C [23] 57.0215	ENSP00000205948	Beta-2-glycoprotein 1 Precursor (Beta-2-glycoprotein I)(Beta(2)GPI)(B2GPI)(Apolipoprotein H)(Apo-H)(Activated protein C-binding protein)(APC inhibitor)(Anticardiolipin cofactor) [Source: UniProt P027
3134.1.1	-9.4	5.29	2211.104	-0.0074	3	39.26	iqvr	58	LGEHNIDVLEGNEQFINAAK	77	iith		sp|TRYP_PIG|	Trypsin; EC 3.4.21.4; Flags: Precursor;
3136.1.1	-6.3	4.98	2440.1751	-0.0097	3	41.48	revk	205	CPFPSRPDNGFVNYPAKPTLY	225	ykdk	C [205] 57.0215	ENSP00000205948	Beta-2-glycoprotein 1 Precursor (Beta-2-glycoprotein I)(Beta(2)GPI)(B2GPI)(Apolipoprotein H)(Apo-H)(Activated protein C-binding protein)(APC inhibitor)(Anticardiolipin cofactor) [Source: UniProt P027
3139.1.1	-3.4	5.14	2211.104	0.0003	3	39.26	iqvr	58	LGEHNIDVLEGNEQFINAAK	77	iith		sp|TRYP_PIG|	Trypsin; EC 3.4.21.4; Flags: Precursor;
3140.1.1	-5.5	4.95	2440.1751	0.0079	3	41.48	revk	205	CPFPSRPDNGFVNYPAKPTLY	225	ykdk	C [205] 57.0215	ENSP00000205948	Beta-2-glycoprotein 1 Precursor (Beta-2-glycoprotein I)(Beta(2)GPI)(B2GPI)(Apolipoprotein H)(Apo-H)(Activated protein C-binding protein)(APC inhibitor)(Anticardiolipin cofactor) [Source: UniProt P027
3141.1.1	-5.5	4.52	1179.535	0.0013	2	30.68	slyr	168	DTAVFECLPQ	177	hamf	C [174] 57.0215	ENSP00000205948	Beta-2-glycoprotein 1 Precursor (Beta-2-glycoprotein I)(Beta(2)GPI)(B2GPI)(Apolipoprotein H)(Apo-H)(Activated protein C-binding protein)(APC inhibitor)(Anticardiolipin cofactor) [Source: UniProt P027
3147.1.1	-4.8	5.02	2440.1751	0.002	3	41.48	revk	205	CPFPSRPDNGFVNYPAKPTLY	225	ykdk	C [205] 57.0215	ENSP00000205948	Beta-2-glycoprotein 1 Precursor (Beta-2-glycoprotein I)(Beta(2)GPI)(B2GPI)(Apolipoprotein H)(Apo-H)(Activated protein C-binding protein)(APC inhibitor)(Anticardiolipin cofactor) [Source: UniProt P027
3148.1.1	-7.7	5.02	1179.535	-0.0003	2	30.68	slyr	168	DTAVFECLPQ	177	hamf	C [174] 57.0215	ENSP00000205948	Beta-2-glycoprotein 1 Precursor (Beta-2-glycoprotein I)(Beta(2)GPI)(B2GPI)(Apolipoprotein H)(Apo-H)(Activated protein C-binding protein)(APC inhibitor)(Anticardiolipin cofactor) [Source: UniProt P027
3165.1.1	-2.8	5.86	2958.4139	-0.0043	4	41.61	revk	205	CPFPSRPDNGFVNYPAKPTLYYKDI	229	atfg	C [205] 57.0215, N [213] 0.9848, (	ENSP00000205948	Beta-2-glycoprotein 1 Precursor (Beta-2-glycoprotein I)(Beta(2)GPI)(B2GPI)(Apolipoprotein H)(Apo-H)(Activated protein C-binding protein)(APC inhibitor)(Anticardiolipin cofactor) [Source: UniProt P027
3166.1.1	-2.2	4.74	2425.1017	0.0081	3	36.69	vplk	39	TFYEPGEEITYSCKPGYVSR	58	ggmr	C [51] 57.0215, T [39] 42.0106	ENSP00000205948	Beta-2-glycoprotein 1 Precursor (Beta-2-glycoprotein I)(Beta(2)GPI)(B2GPI)(Apolipoprotein H)(Apo-H)(Activated protein C-binding protein)(APC inhibitor)(Anticardiolipin cofactor) [Source: UniProt P027
3169.1.1	-13.6	4.69	1759.7843	0.0055	2	31.14	atgs	28	DPVLCFTQYEESSGK	42	ckgl	C [32] 57.0215	ENSP00000380189	Properdin precursor (Factor P). Source: Uniprot/SWISSPROT P27918
3172.1.1	-4.7	5.67	2958.4139	-0.0026	4	41.61	revk	205	CPFPSRPDNGFVNYPAKPTLYYKDI	229	atfg	C [205] 57.0215, N [213] 0.9848, (	ENSP00000205948	Beta-2-glycoprotein 1 Precursor (Beta-2-glycoprotein I)(Beta(2)GPI)(B2GPI)(Apolipoprotein H)(Apo-H)(Activated protein C-binding protein)(APC inhibitor)(Anticardiolipin cofactor) [Source: UniProt P027
3173.1.1	-6.9	4.78	2958.4139	0.0048	3	41.61	revk	205	CPFPSRPDNGFVNYPAKPTLYYKDI	229	atfg	C [205] 57.0215, N [213] 0.9848, (	ENSP00000205948	Beta-2-glycoprotein 1 Precursor (Beta-2-glycoprotein I)(Beta(2)GPI)(B2GPI)(Apolipoprotein H)(Apo-H)(Activated protein C-binding protein)(APC inhibitor)(Anticardiolipin cofactor) [Source: UniProt P027
3174.1.1	-14.6	4.95	1759.7843	0.0008	2	31.14	atgs	28	DPVLCFTQYEESSGK	42	ckgl	C [32] 57.0215	ENSP00000380189	Properdin precursor (Factor P). Source: Uniprot/SWISSPROT P27918
3176.1.1	-1.2	4.61	1174.5561	-0.0027	2	30.06	tspk	3287	YFLSIECSR	3295	ksss	C [3293] 57.0215	ENSP00000261800	Protocadherin Fat 2 precursor (hFat2) (Multiple epidermal growth factor-like domains 1). [Source: SWISSPROT (Q9NYQ8)]
3179.1.1	-3.6	5.4	2958.4139	-0.0051	4	41.61	revk	205	CPFPSRPDNGFVNYPAKPTLYYKDI	229	atfg	C [205] 57.0215, N [213] 0.9848, (	ENSP00000205948	Beta-2-glycoprotein 1 Precursor (Beta-2-glycoprotein I)(Beta(2)GPI)(B2GPI)(Apolipoprotein H)(Apo-H)(Activated protein C-binding protein)(APC inhibitor)(Anticardiolipin cofactor) [Source: UniProt P027
3180.1.1	-13	4.88	1759.7843	-0.0001	2	31.14	atgs	28	DPVLCFTQYEESSGK	42	ckgl	C [32] 57.0215	ENSP00000380189	Properdin precursor (Factor P). Source: Uniprot/SWISSPROT P27918
3189.1.1	-4	4.51	2958.414	0.013	3	41.61	revk	205	CPFPSRPDNGFVNYPAKPTLYYKDI	229	atfg	C [205] 57.0215, N [217] 0.9848, (	ENSP00000205948	Beta-2-glycoprotein 1 Precursor (Beta-2-glycoprotein I)(Beta(2)GPI)(B2GPI)(Apolipoprotein H)(Apo-H)(Activated protein C-binding protein)(APC inhibitor)(Anticardiolipin cofactor) [Source: UniProt P027
3204.1.1	-2.3	4.38	1102.6255	0.0015	2	31.61	icpp	145	PSIPTFATLR	154	vykp		ENSP00000205948	Beta-2-glycoprotein 1 Precursor (Beta-2-glycoprotein I)(Beta(2)GPI)(B2GPI)(Apolipoprotein H)(Apo-H)(Activated protein C-binding protein)(APC inhibitor)(Anticardiolipin cofactor) [Source: UniProt P027
3205.1.1	-5.9	4.29	1226.6528	0.0015	2	30.81	sgtr	362	AAAATGTIFTFR	373	sarl		ENSP00000333203	Plasma serine protease inhibitor precursor (PCI) (Protein C inhibitor) (Serpin A5) (Plasminogen activator inhibitor 3) (PAI-3) (PAI3) (Acrosomal serine protease inhibitor). Source: Uniprot/SWISSPROT P0515
3214.1.1	-5.1	5.03	2169.137	-0.0037	3	39.23	vaia	20	GRTCPKPDDLPFSTVVPLK	38	tfye	C [23] 57.0215, G [20] 42.0106	ENSP00000205948	Beta-2-glycoprotein 1 Precursor (Beta-2-glycoprotein I)(Beta(2)GPI)(B2GPI)(Apolipoprotein H)(Apo-H)(Activated protein C-binding protein)(APC inhibitor)(Anticardiolipin cofactor) [Source: UniProt P027
3220.1.1	-5.6	4.97	2169.137	0.0021	3	39.23	vaia	20	GRTCPKPDDLPFSTVVPLK	38	tfye	C [23] 57.0215, G [20] 42.0106	ENSP00000205948	Beta-2-glycoprotein 1 Precursor (Beta-2-glycoprotein I)(Beta(2)GPI)(B2GPI)(Apolipoprotein H)(Apo-H)(Activated protein C-binding protein)(APC inhibitor)(Anticardiolipin cofactor) [Source: UniProt P027
3221.1.1	-3.3	4.75	2127.126	0.993	3	39.23	vaia	20	GRTCPKPDDLPFSTVVPLK	38	tfye	C [23] 57.0215	ENSP00000205948	Beta-2-glycoprotein 1 Precursor (Beta-2-glycoprotein I)(Beta(2)GPI)(B2GPI)(Apolipoprotein H)(Apo-H)(Activated protein C-binding protein)(APC inhibitor)(Anticardiolipin cofactor) [Source: UniProt P027
3226.1.1	-6.6	4.87	2169.137	0.0008	3	39.23	vaia	20	GRTCPKPDDLPFSTVVPLK	38	tfye	C [23] 57.0215, G [20] 42.0106	ENSP00000205948	Beta-2-glycoprotein 1 Precursor (Beta-2-glycoprotein I)(Beta(2)GPI)(B2GPI)(Apolipoprotein H)(Apo-H)(Activated protein C-binding protein)(APC inhibitor)(Anticardiolipin cofactor) [Source: UniProt P027
3230.1.1	-2.4	4.3	2169.137	0.0045	4	39.23	vaia	20	GRTCPKPDDLPFSTVVPLK	38	tfye	C [23] 57.0215, G [20] 42.0106	ENSP00000205948	Beta-2-glycoprotein 1 Precursor (Beta-2-glycoprotein I)(Beta(2)GPI)(B2GPI)(Apolipoprotein H)(Apo-H)(Activated protein C-binding protein)(APC inhibitor)(Anticardiolipin cofactor) [Source: UniProt P027
3235.1.1	-2.1	4.64	2127.126	0.986	4	39.23	vaia	20	GRTCPKPDDLPFSTVVPLK	38	tfye	C [23] 57.0215	ENSP00000205948	Beta-2-glycoprotein 1 Precursor (Beta-2-glycoprotein I)(Beta(2)GPI)(B2GPI)(Apolipoprotein H)(Apo-H)(Activated protein C-binding protein)(APC inhibitor)(Anticardiolipin cofactor) [Source: UniProt P027
3236.1.1	-4.6	4.55	1348.5945	0.0002	2	31.33	vplk	39	TFYEPGEEITY	49	sckp		ENSP00000205948	Beta-2-glycoprotein 1 Precursor (Beta-2-glycoprotein I)(Beta(2)GPI)(B2GPI)(Apolipoprotein H)(Apo-H)(Activated protein C-binding protein)(APC inhibitor)(Anticardiolipin cofactor) [Source: UniProt P027
3237.1.1	-3.5	4.55	1255.6753	0.0023	2	10.6	mgsr	43	QKAATEGPAQVR	54	aarp		ENSP00000321601	No description
3240.1.1	-3.9	4.62	1348.5945	-0.0002	2	31.33	vplk	39	TFYEPGEEITY	49	sckp		ENSP00000205948	Beta-2-glycoprotein 1 Precursor (Beta-2-glycoprotein I)(Beta(2)GPI)(B2GPI)(Apolipoprotein H)(Apo-H)(Activated protein C-binding protein)(APC inhibitor)(Anticardiolipin cofactor) [Source: UniProt P027
3243.1.1	-2.9	4.67	1255.6753	0.0021	2	10.6	mgsr	43	QKAATEGPAQVR	54	aarp		ENSP00000321601	No description
3249.1.1	-4.9	4.27	1348.5945	-0.0005	2	31.33	vplk	39	TFYEPGEEITY	49	sckp		ENSP00000205948	Beta-2-glycoprotein 1 Precursor (Beta-2-glycoprotein I)(Beta(2)GPI)(B2GPI)(Apolipoprotein H)(Apo-H)(Activated protein C-binding protein)(APC inhibitor)(Anticardiolipin cofactor) [Source: UniProt P027
3264.1.1	-5.1	4.8	1617.7876	0.0015	2	35.41	lgtr	53	WGLGGTCVNVGCIPK	67	klmh	C [59] 57.0215, C [64] 57.0215	ENSP00000373506	Thioredoxin reductase 1, cytoplasmic (TR)(EC 1.8.1.9)(Thioredoxin reductase TR1)(KM-102-derived reductase-like factor)(Gene associated with retinoid-IFN-induced mortality 12 protein)(GRIM-12) [Sc
3268.1.1	-5.9	4.8	1617.7876	0.0046	2	35.41	lgtr	53	WGLGGTCVNVGCIPK	67	klmh	C [59] 57.0215, C [64] 57.0215	ENSP00000373506	Thioredoxin reductase 1, cytoplasmic (TR)(EC 1.8.1.9)(Thioredoxin reductase TR1)(KM-102-derived reductase-like factor)(Gene associated with retinoid-IFN-induced mortality 12 protein)(GRIM-12) [Sc
3274.1.1	-4.2	4.7	1617.7876	0.0058	2	35.41	lgtr	53	WGLGGTCVNVGCIPK	67	klmh	C [59] 57.0215, C [64] 57.0215	ENSP00000373506	Thioredoxin reductase 1, cytoplasmic (TR)(EC 1.8.1.9)(Thioredoxin reductase TR1)(KM-102-derived reductase-like factor)(Gene associated with retinoid-IFN-induced mortality 12 protein)(GRIM-12) [Sc
3284.1.1	-5.7	4.87	2714.3072	0.006	3	41.86	revk	205	CPFPSRPDNGFVNYPAKPTLYYK	227	dkat	C [205] 57.0215, C [205] -17.0265	ENSP00000205948	Beta-2-glycoprotein 1 Precursor (Beta-2-glycoprotein I)(Beta(2)GPI)(B2GPI)(Apolipoprotein H)(Apo-H)(Activated protein C-binding protein)(APC inhibitor)(Anticardiolipin cofactor) [Source: UniProt P027
3288.1.1	-2.4	4.02	1208.5405	-0.0043	2	28.72	evpk	325	CFKEHSSLAF	334	wktd	C [325] 57.0215, C [325] -17.0265	ENSP00000205948	Beta-2-glycoprotein 1 Precursor (Beta-2-glycoprotein I)(Beta(2)GPI)(B2GPI)(Apolipoprotein H)(Apo-H)(Activated protein C-binding protein)(APC inhibitor)(Anticardiolipin cofactor) [Source: UniProt P027
3290.1.1	-5.8	5.67	2714.3072	0.0045	3	41.86	revk	205	CPFPSRPDNGFVNYPAKPTLYYK	227	dkat	C [205] 57.0215, C [205] -17.0265	ENSP00000205948	Beta-2-glycoprotein 1 Precursor (Beta-2-glycoprotein I)(Beta(2)GPI)(B2GPI)(Apolipoprotein H)(Apo-H)(Activated protein C-binding protein)(APC inhibitor)(Anticardiolipin cofactor) [Source: UniProt P027
3296.1.1	-5.7	6.34	2714.3072	-0.0001	3	41.86	revk	205	CPFPSRPDNGFVNYPAKPTLYYK	227	dkat	C [205] 57.0215, C [205] -17.0265	ENSP00000205948	Beta-2-glycoprotein 1 Precursor (Beta-2-glycoprotein I)(Beta(2)GPI)(B2GPI)(Apolipoprotein H)(Apo-H)(Activated protein C-binding protein)(APC inhibitor)(Anticardiolipin cofactor) [Source: UniProt P027
3297.1.1	-8	5.11	2068.8838	-0.0019	2	30.19	kekk	307	CSYTEDAQCIDGTIEVPK	324	cfke	C [307] 57.0215, C [315] 57.0215,	ENSP00000205948	Beta-2-glycoprotein 1 Precursor (Beta-2-glycoprotein I)(Beta(2)GPI)(B2GPI)(Apolipoprotein H)(Apo-H)(Activated protein C-binding protein)(APC inhibitor)(Anticardiolipin cofactor) [Source: UniProt P027
3306.1.1	-12.2	4.75	2068.8838	0.0076	2	30.19	kekk	307	CSYTEDAQCIDGTIEVPK	324	cfke	C [307] 57.0215, C [315] 57.0215,	ENSP00000205948	Beta-2-glycoprotein 1 Precursor (Beta-2-glycoprotein I)(Beta(2)GPI)(B2GPI)(Apolipoprotein H)(Apo-H)(Activated protein C-binding protein)(APC inhibitor)(Anticardiolipin cofactor) [Source: UniProt P027
3313.1.1	-6.6	5.52	1914.0041	-0.0015	2	39.7	iagr	22	TCPKPDDLPFSTVVPLK	38	tfye	C [23] 57.0215	ENSP00000205948	Beta-2-glycoprotein 1 Precursor (Beta-2-glycoprotein I)(Beta(2)GPI)(B2GPI)(Apolipoprotein H)(Apo-H)(Activated protein C-binding protein)(APC inhibitor)(Anticardiolipin cofactor) [Source: UniProt P027
3315.1.1	-4.1	6.69	1914.0041	-0.0027	3	39.7	iagr	22	TCPKPDDLPFSTVVPLK	38	tfye	C [23] 57.0215	ENSP00000205948	Beta-2-glycoprotein 1 Precursor (Beta-2-glycoprotein I)(Beta(2)GPI)(B2GPI)(Apolipoprotein H)(Apo-H)(Activated protein C-binding protein)(APC inhibitor)(Anticardiolipin cofactor) [Source: UniProt P027
3316.1.1	-7	5.61	1914.0041	-0.0026	2	39.7	iagr	22	TCPKPDDLPFSTVVPLK	38	tfye	C [23] 57.0215	ENSP00000205948	Beta-2-glycoprotein 1 Precursor (Beta-2-glycoprotein I)(Beta(2)GPI)(B2GPI)(Apolipoprotein H)(Apo-H)(Activated protein C-binding protein)(APC inhibitor)(Anticardiolipin cofactor) [Source: UniProt P027
3322.1.1	-7.7	6.1	1914.0041	0.0002	2	39.7	iagr	22	TCPKPDDLPFSTVVPLK	38	tfye	C [23] 57.0215	ENSP00000205948	Beta-2-glycoprotein 1 Precursor (Beta-2-glycoprotein I)(Beta(2)GPI)(B2GPI)(Apolipoprotein H)(Apo-H)(Activated protein C-binding protein)(APC inhibitor)(Anticardiolipin cofactor) [Source: UniProt P027
3324.1.1	-5.1	5.59	1652.9257	0.0011	3	38.49	grtc	24	PKPDDLPFSTVVPLK	38	tfye		ENSP00000205948	Beta-2-glycoprotein 1 Precursor (Beta-2-glycoprotein I)(Beta(2)GPI)(B2GPI)(Apolipoprotein H)(Apo-H)(Activated protein C-binding protein)(APC inhibitor)(Anticardiolipin cofactor) [Source: UniProt P027
3330.1.1	-2.7	5.66	1652.9257	0.0007	3	38.49	grtc	24	PKPDDLPFSTVVPLK	38	tfye		ENSP00000205948	Beta-2-glycoprotein 1 Precursor (Beta-2-glycoprotein I)(Beta(2)GPI)(B2GPI)(Apolipoprotein H)(Apo-H)(Activated protein C-binding protein)(APC inhibitor)(Anticardiolipin cofactor) [Source: UniProt P027
3333.1.1	-4.5	4.38	1258.6889	-0.0011	2	32.92	gank	95	EKLEATINELV	105	]		ENSP00000363641	Thioredoxin (Trx)(ATL-derived factor)(ADF)(Surface-associated sulphydryl protein)(SASP) [Source: UniProt P10599]
3337.1.1	-4.7	5.73	1652.9257	0.0013	3	38.49	grtc	24	PKPDDLPFSTVVPLK	38	tfye		ENSP00000205948	Beta-2-glycoprotein 1 Precursor (Beta-2-glycoprotein I)(Beta(2)GPI)(B2GPI)(Apolipoprotein H)(Apo-H)(Activated protein C-binding protein)(APC inhibitor)(Anticardiolipin cofactor) [Source: UniProt P027
3338.1.1	-2.8	5.04	1258.6889	0.0025	2	32.92	gank	95	EKLEATINELV	105	]		ENSP00000363641	Thioredoxin (Trx)(ATL-derived factor)(ADF)(Surface-associated sulphydryl protein)(SASP) [Source: UniProt P10599]
3342.1.1	-5.7	5.4	1522.7147	0.0022	2	36.42	evpk	325	CFKEHSSLAFWK	336	tdas	C [325] 57.0215, C [325] -17.0265	ENSP00000205948	Beta-2-glycoprotein 1 Precursor (Beta-2-glycoprotein I)(Beta(2)GPI)(B2GPI)(Apolipoprotein H)(Apo-H)(Activated protein C-binding protein)(APC inhibitor)(Anticardiolipin cofactor) [Source: UniProt P027
3348.1.1	-1.5	6.47	1522.7147	0.0017	3	36.42	evpk	325	CFKEHSSLAFWK	336	tdas	C [325] 57.0215, C [325] -17.0265	ENSP00000205948	Beta-2-glycoprotein 1 Precursor (Beta-2-glycoprotein I)(Beta(2)GPI)(B2GPI)(Apolipoprotein H)(Apo-H)(Activated protein C-binding protein)(APC inhibitor)(Anticardiolipin cofactor) [Source: UniProt P027
3349.1.1	-7.1	5.64	1522.7147	0.0014	2	36.42	evpk	325	CFKEHSSLAFWK	336	tdas	C [325] 57.0215, C [325] -17.0265	ENSP00000205948	Beta-2-glycoprotein 1 Precursor (Beta-2-glycoprotein I)(Beta(2)GPI)(B2GPI)(Apolipoprotein H)(Apo-H)(Activated protein C-binding protein)(APC inhibitor)(Anticardiolipin cofactor) [Source: UniProt P027
3352.1.1	-4.3	4.71	1221.5431	-0.0022	2	35.64	cevk	73	CMPTFQFFK	81	kgqk	C [73] 57.0215, M [74] 15.9949	ENSP00000363641	Thioredoxin (Trx)(ATL-derived factor)(ADF)(Surface-associated sulphydryl protein)(SASP) [Source: UniProt P10599]
3354.1.1	-2	6.41	1522.7147	-0.0007	3	36.42	evpk	325	CFKEHSSLAFWK	336	tdas	C [325] 57.0215, C [325] -17.0265	ENSP00000205948	Beta-2-glycoprotein 1 Precursor (Beta-2-glycoprotein I)(Beta(2)GPI)(B2GPI)(Apolipoprotein H)(Apo-H)(Activated protein C-binding protein)(APC inhibitor)(Anticardiolipin cofactor) [Source: UniProt P027
3355.1.1	-7.8	5.77	1522.7147	-0.0019	2	36.42	evpk	325	CFKEHSSLAFWK	336	tdas	C [325] 57.0215, C [325] -17.0265	ENSP00000205948	Beta-2-glycoprotein 1 Precursor (Beta-2-glycoprotein I)(Beta(2)GPI)(B2GPI)(Apolipoprotein H)(Apo-H)(Activated protein C-binding protein)(APC inhibitor)(Anticardiolipin cofactor) [Source: UniProt P027
3358.1.1	-5	4.8	1221.5431	-0.0022	2	35.64	cevk	73	CMPTFQFFK	81	kgqk	C [73] 57.0215, M [74] 15.9949	ENSP00000363641	Thioredoxin (Trx)(ATL-derived factor)(ADF)(Surface-associated sulphydryl protein)(SASP) [Source: UniProt P10599]
3360.1.1	-1.3	6.5	1522.7147	-0.0006	3	36.42	evpk	325	CFKEHSSLAFWK	336	tdas	C [325] 57.0215, C [325] -17.0265	ENSP00000205948	Beta-2-glycoprotein 1 Precursor (Beta-2-glycoprotein I)(Beta(2)GPI)(B2GPI)(Apolipoprotein H)(Apo-H)(Activated protein C-binding protein)(APC inhibitor)(Anticardiolipin cofactor) [Source: UniProt P027
3365.1.1	-3.4	4.74	1221.5431	-0.0001	2	35.64	cevk	73	CMPTFQFFK	81	kgqk	C [73] 57.0215, M [74] 15.9949	ENSP00000363641	Thioredoxin (Trx)(ATL-derived factor)(ADF)(Surface-associated sulphydryl protein)(SASP) [Source: UniProt P10599]
3368.1.1	-2.1	4.84	1853.966	0.014	2	35.58	kfir	250	QFVPIKVEQIEAGTPGR	266	lrvv	Q [250] 0.9848, Q [258] 0.9848, Q	ENSP00000373506	Thioredoxin reductase 1, cytoplasmic (TR)(EC 1.8.1.9)(Thioredoxin reductase TR1)(KM-102-derived reductase-like factor)(Gene associated with retinoid-IFN-induced mortality 12 protein)(GRIM-12) [Sc
3374.1.1	-1.1	4.87	2195.146	1.989	3	42.45	tpvk	134	WQDVQLKLANVEDIDGPVR	152	fige		ENSP00000366522:reversed	
3385.1.1	-6.7	6.24	2715.292	0.0063	3	41.86	revk	205	CPFPSRPDNGFVNYPAKPTLYYK	227	dkat	C [205] 57.0215, N [213] 0.9848, (	ENSP00000205948	Beta-2-glycoprotein 1 Precursor (Beta-2-glycoprotein I)(Beta(2)GPI)(B2GPI)(Apolipoprotein H)(Apo-H)(Activated protein C-binding protein)(APC inhibitor)(Anticardiolipin cofactor) [Source: UniProt P027
3392.1.1	-7.7	6.27	2715.292	0.0004	3	41.86	revk	205	CPFPSRPDNGFVNYPAKPTLYYK	227	dkat	C [205] 57.0215, N [213] 0.9848, (	ENSP00000205948	Beta-2-glycoprotein 1 Precursor (Beta-2-glycoprotein I)(Beta(2)GPI)(B2GPI)(Apolipoprotein H)(Apo-H)(Activated protein C-binding protein)(APC inhibitor)(Anticardiolipin cofactor) [Source: UniProt P027
3395.1.1	-2.6	4.57	1048.586	-0.0005	2	35	ggmr	63	KFICPLTGL	71	wpin	C [66] 57.0215	ENSP00000205948	Beta-2-glycoprotein 1 Precursor (Beta-2-glycoprotein I)(Beta(2)GPI)(B2GPI)(Apolipoprotein H)(Apo-H)(Activated protein C-binding protein)(APC inhibitor)(Anticardiolipin cofactor) [Source: UniProt P027
3398.1.1	-6.8	6.13	2715.292	0.0023	3	41.86	revk	205	CPFPSRPDNGFVNYPAKPTLYYK	227	dkat	C [205] 57.0215, N [213] 0.9848, (	ENSP00000205948	Beta-2-glycoprotein 1 Precursor (Beta-2-glycoprotein I)(Beta(2)GPI)(B2GPI)(Apolipoprotein H)(Apo-H)(Activated protein C-binding protein)(APC inhibitor)(Anticardiolipin cofactor) [Source: UniProt P027
3413.1.1	-4.6	5.32	1914.0041	-0.003	3	39.7	iagr	22	TCPKPDDLPFSTVVPLK	38	tfye	C [23] 57.0215	ENSP00000205948	Beta-2-glycoprotein 1 Precursor (Beta-2-glycoprotein I)(Beta(2)GPI)(B2GPI)(Apolipoprotein H)(Apo-H)(Activated protein C-binding protein)(APC inhibitor)(Anticardiolipin cofactor) [Source: UniProt P027
3420.1.1	-5.1	4.98	1914.0041	0.0004	3	39.7	iagr	22	TCPKPDDLPFSTVVPLK	38	tfye	C [23] 57.0215	ENSP00000205948	Beta-2-glycoprotein 1 Precursor (Beta-2-glycoprotein I)(Beta(2)GPI)(B2GPI)(Apolipoprotein H)(Apo-H)(Activated protein C-binding protein)(APC inhibitor)(Anticardiolipin cofactor) [Source: UniProt P027
3433.1.1	-1.1	4.65	2765.317	0.991	3	32.22	gagr	564	GLHTQGVLDTAALPPDPSSTCK	585	rqlg	C [584] 500.199, C [584] 57.0215	ENSP00000313670	ALS2 C-terminal like isoform 1 Source: RefSeq_peptide NP_667340
3435.1.1	-1.9	4.53	1001.5514	0.0021	2	30.16	nkek	97	LEATINELV	105	]		ENSP00000363641	Thioredoxin (Trx)(ATL-derived factor)(ADF)(Surface-associated sulphydryl protein)(SASP) [Source: UniProt P10599]
3463.1.1	-3.8	4.42	1502.7784	0.0012	2	35.15	ctpr	83	VCPFAGILENGAVR	96	yttf	C [84] 57.0215	ENSP00000205948	Beta-2-glycoprotein 1 Precursor (Beta-2-glycoprotein I)(Beta(2)GPI)(B2GPI)(Apolipoprotein H)(Apo-H)(Activated protein C-binding protein)(APC inhibitor)(Anticardiolipin cofactor) [Source: UniProt P027
3464.1.1	-8.1	4.09	2068.8838	-0.0056	2	30.19	kekk	307	CSYTEDAQCIDGTIEVPK	324	cfke	C [307] 57.0215, C [315] 57.0215,	ENSP00000205948	Beta-2-glycoprotein 1 Precursor (Beta-2-glycoprotein I)(Beta(2)GPI)(B2GPI)(Apolipoprotein H)(Apo-H)(Activated protein C-binding protein)(APC inhibitor)(Anticardiolipin cofactor) [Source: UniProt P027
3483.1.1	-8.7	4.9	1503.7632	0.0032	2	35.15	ctpr	83	VCPFAGILENGAVR	96	yttf	C [84] 57.0215, N [92] 0.9848	ENSP00000205948	Beta-2-glycoprotein 1 Precursor (Beta-2-glycoprotein I)(Beta(2)GPI)(B2GPI)(Apolipoprotein H)(Apo-H)(Activated protein C-binding protein)(APC inhibitor)(Anticardiolipin cofactor) [Source: UniProt P027
3489.1.1	-7.4	5.22	1503.7632	-0.0007	2	35.15	ctpr	83	VCPFAGILENGAVR	96	yttf	C [84] 57.0215, N [92] 0.9848	ENSP00000205948	Beta-2-glycoprotein 1 Precursor (Beta-2-glycoprotein I)(Beta(2)GPI)(B2GPI)(Apolipoprotein H)(Apo-H)(Activated protein C-binding protein)(APC inhibitor)(Anticardiolipin cofactor) [Source: UniProt P027
3491.1.1	-8.2	4.91	2715.292	0.011	3	41.86	revk	205	CPFPSRPDNGFVNYPAKPTLYYK	227	dkat	C [205] 57.0215, N [213] 0.9848, (	ENSP00000205948	Beta-2-glycoprotein 1 Precursor (Beta-2-glycoprotein I)(Beta(2)GPI)(B2GPI)(Apolipoprotein H)(Apo-H)(Activated protein C-binding protein)(APC inhibitor)(Anticardiolipin cofactor) [Source: UniProt P027
3497.1.1	-2.6	5.05	1956.0145	0	3	39.7	iagr	22	TCPKPDDLPFSTVVPLK	38	tfye	C [23] 57.0215, T [22] 42.0106	ENSP00000205948	Beta-2-glycoprotein 1 Precursor (Beta-2-glycoprotein I)(Beta(2)GPI)(B2GPI)(Apolipoprotein H)(Apo-H)(Activated protein C-binding protein)(APC inhibitor)(Anticardiolipin cofactor) [Source: UniProt P027
3499.1.1	-3.4	4.74	1503.7632	-0.001	3	35.15	ctpr	83	VCPFAGILENGAVR	96	yttf	C [84] 57.0215, N [92] 0.9848	ENSP00000205948	Beta-2-glycoprotein 1 Precursor (Beta-2-glycoprotein I)(Beta(2)GPI)(B2GPI)(Apolipoprotein H)(Apo-H)(Activated protein C-binding protein)(APC inhibitor)(Anticardiolipin cofactor) [Source: UniProt P027
3501.1.1	-4.2	5.25	1956.0145	-0.0009	3	39.7	iagr	22	TCPKPDDLPFSTVVPLK	38	tfye	C [23] 57.0215, T [22] 42.0106	ENSP00000205948	Beta-2-glycoprotein 1 Precursor (Beta-2-glycoprotein I)(Beta(2)GPI)(B2GPI)(Apolipoprotein H)(Apo-H)(Activated protein C-binding protein)(APC inhibitor)(Anticardiolipin cofactor) [Source: UniProt P027
3506.1.1	-4.1	5.06	1503.7632	0.001	3	35.15	ctpr	83	VCPFAGILENGAVR	96	yttf	C [84] 57.0215, N [92] 0.9848	ENSP00000205948	Beta-2-glycoprotein 1 Precursor (Beta-2-glycoprotein I)(Beta(2)GPI)(B2GPI)(Apolipoprotein H)(Apo-H)(Activated protein C-binding protein)(APC inhibitor)(Anticardiolipin cofactor) [Source: UniProt P027
3508.1.1	-4	5.64	1956.0145	0.0025	3	39.7	iagr	22	TCPKPDDLPFSTVVPLK	38	tfye	C [23] 57.0215, T [22] 42.0106	ENSP00000205948	Beta-2-glycoprotein 1 Precursor (Beta-2-glycoprotein I)(Beta(2)GPI)(B2GPI)(Apolipoprotein H)(Apo-H)(Activated protein C-binding protein)(APC inhibitor)(Anticardiolipin cofactor) [Source: UniProt P027
3509.1.1	-4.7	5.31	1503.7632	0.0017	3	35.15	ctpr	83	VCPFAGILENGAVR	96	yttf	C [84] 57.0215, N [92] 0.9848	ENSP00000205948	Beta-2-glycoprotein 1 Precursor (Beta-2-glycoprotein I)(Beta(2)GPI)(B2GPI)(Apolipoprotein H)(Apo-H)(Activated protein C-binding protein)(APC inhibitor)(Anticardiolipin cofactor) [Source: UniProt P027
3512.1.1	-4.9	4.95	1956.0145	0.0045	2	39.7	iagr	22	TCPKPDDLPFSTVVPLK	38	tfye	C [23] 57.0215, T [22] 42.0106	ENSP00000205948	Beta-2-glycoprotein 1 Precursor (Beta-2-glycoprotein I)(Beta(2)GPI)(B2GPI)(Apolipoprotein H)(Apo-H)(Activated protein C-binding protein)(APC inhibitor)(Anticardiolipin cofactor) [Source: UniProt P027
3515.1.1	-2.7	5.01	1956.0145	0.0002	2	39.7	iagr	22	TCPKPDDLPFSTVVPLK	38	tfye	C [23] 57.0215, T [22] 42.0106	ENSP00000205948	Beta-2-glycoprotein 1 Precursor (Beta-2-glycoprotein I)(Beta(2)GPI)(B2GPI)(Apolipoprotein H)(Apo-H)(Activated protein C-binding protein)(APC inhibitor)(Anticardiolipin cofactor) [Source: UniProt P027
3519.1.1	-5.8	4.35	1914.0041	0.0019	3	39.7	iagr	22	TCPKPDDLPFSTVVPLK	38	tfye	C [23] 57.0215	ENSP00000205948	Beta-2-glycoprotein 1 Precursor (Beta-2-glycoprotein I)(Beta(2)GPI)(B2GPI)(Apolipoprotein H)(Apo-H)(Activated protein C-binding protein)(APC inhibitor)(Anticardiolipin cofactor) [Source: UniProt P027
3522.1.1	-3.8	5.02	1956.0145	0.0019	2	39.7	iagr	22	TCPKPDDLPFSTVVPLK	38	tfye	C [23] 57.0215, T [22] 42.0106	ENSP00000205948	Beta-2-glycoprotein 1 Precursor (Beta-2-glycoprotein I)(Beta(2)GPI)(B2GPI)(Apolipoprotein H)(Apo-H)(Activated protein C-binding protein)(APC inhibitor)(Anticardiolipin cofactor) [Source: UniProt P027
3531.1.1	-1.5	4.59	1525.7428	-0.0002	2	29.08	selr	1279	RTVYLCTDDNLR	1290	gadm	C [1284] 57.0215	ENSP00000359630	
3540.1.1	-2.2	4.68	2714.307	1.012	3	41.86	revk	205	CPFPSRPDNGFVNYPAKPTLYYK	227	dkat	C [205] 57.0215, C [205] -17.0265	ENSP00000205948	Beta-2-glycoprotein 1 Precursor (Beta-2-glycoprotein I)(Beta(2)GPI)(B2GPI)(Apolipoprotein H)(Apo-H)(Activated protein C-binding protein)(APC inhibitor)(Anticardiolipin cofactor) [Source: UniProt P027
3542.1.1	-3.5	4.2	1914.0041	0.0003	3	39.7	iagr	22	TCPKPDDLPFSTVVPLK	38	tfye	C [23] 57.0215	ENSP00000205948	Beta-2-glycoprotein 1 Precursor (Beta-2-glycoprotein I)(Beta(2)GPI)(B2GPI)(Apolipoprotein H)(Apo-H)(Activated protein C-binding protein)(APC inhibitor)(Anticardiolipin cofactor) [Source: UniProt P027
3571.1.1	-8.5	7.25	1502.7784	-0.0008	2	35.15	ctpr	83	VCPFAGILENGAVR	96	yttf	C [84] 57.0215	ENSP00000205948	Beta-2-glycoprotein 1 Precursor (Beta-2-glycoprotein I)(Beta(2)GPI)(B2GPI)(Apolipoprotein H)(Apo-H)(Activated protein C-binding protein)(APC inhibitor)(Anticardiolipin cofactor) [Source: UniProt P027
3574.1.1	-10.5	7.26	1502.7784	0.0023	2	35.15	ctpr	83	VCPFAGILENGAVR	96	yttf	C [84] 57.0215	ENSP00000205948	Beta-2-glycoprotein 1 Precursor (Beta-2-glycoprotein I)(Beta(2)GPI)(B2GPI)(Apolipoprotein H)(Apo-H)(Activated protein C-binding protein)(APC inhibitor)(Anticardiolipin cofactor) [Source: UniProt P027
3579.1.1	-7.4	7.07	1502.7784	-0.0014	2	35.15	ctpr	83	VCPFAGILENGAVR	96	yttf	C [84] 57.0215	ENSP00000205948	Beta-2-glycoprotein 1 Precursor (Beta-2-glycoprotein I)(Beta(2)GPI)(B2GPI)(Apolipoprotein H)(Apo-H)(Activated protein C-binding protein)(APC inhibitor)(Anticardiolipin cofactor) [Source: UniProt P027
3580.1.1	-1.6	5.16	2134.046	1.022	2	41.6	eilr	259	EAYALCHCLPVLSTDKFK	276	tdfy	C [264] 57.0215, C [266] 57.0215,	ENSP00000304102	COP9 signalosome subunit 6; COP9 subunit 6; MOV34 homolog, 34 kD; H_NH0506M12.12. [Source: RefSeq (NM_006833)]
3588.1.1	-6.8	6.45	1502.7784	0.0025	3	35.15	ctpr	83	VCPFAGILENGAVR	96	yttf	C [84] 57.0215	ENSP00000205948	Beta-2-glycoprotein 1 Precursor (Beta-2-glycoprotein I)(Beta(2)GPI)(B2GPI)(Apolipoprotein H)(Apo-H)(Activated protein C-binding protein)(APC inhibitor)(Anticardiolipin cofactor) [Source: UniProt P027
3593.1.1	-5.7	6.35	1502.7784	0.0013	3	35.15	ctpr	83	VCPFAGILENGAVR	96	yttf	C [84] 57.0215	ENSP00000205948	Beta-2-glycoprotein 1 Precursor (Beta-2-glycoprotein I)(Beta(2)GPI)(B2GPI)(Apolipoprotein H)(Apo-H)(Activated protein C-binding protein)(APC inhibitor)(Anticardiolipin cofactor) [Source: UniProt P027
3598.1.1	-6.1	6.23	1502.7784	0.001	3	35.15	ctpr	83	VCPFAGILENGAVR	96	yttf	C [84] 57.0215	ENSP00000205948	Beta-2-glycoprotein 1 Precursor (Beta-2-glycoprotein I)(Beta(2)GPI)(B2GPI)(Apolipoprotein H)(Apo-H)(Activated protein C-binding protein)(APC inhibitor)(Anticardiolipin cofactor) [Source: UniProt P027

3614.1.1	-5.8	4.65	2423.1486	0.005	2	41.48	revk	205	CPFPSRPDNGFVNYPAKPTLY	225	ykdk	C [205] 57.0215, C [205] -17.0265	ENSP00000205948	Beta-2-glycoprotein 1 Precursor (Beta-2-glycoprotein I)(Beta(2)GPI)(B2GPI)(Apolipoprotein H)(Apo-H)(Activated protein C-binding protein)(APC inhibitor)(Anticardiolipin cofactor) [Source: UniProt P027
3622.1.1	-4.5	4.64	2423.1486	0.004	2	41.48	revk	205	CPFPSRPDNGFVNYPAKPTLY	225	ykdk	C [205] 57.0215, C [205] -17.0265	ENSP00000205948	Beta-2-glycoprotein 1 Precursor (Beta-2-glycoprotein I)(Beta(2)GPI)(B2GPI)(Apolipoprotein H)(Apo-H)(Activated protein C-binding protein)(APC inhibitor)(Anticardiolipin cofactor) [Source: UniProt P027
3635.1.1	-3	4.24	1142.6567	0.006	2	36.03	icpl	69	TGLWPINTLK	78	ctpr		ENSP00000205948	Beta-2-glycoprotein 1 Precursor (Beta-2-glycoprotein I)(Beta(2)GPI)(B2GPI)(Apolipoprotein H)(Apo-H)(Activated protein C-binding protein)(APC inhibitor)(Anticardiolipin cofactor) [Source: UniProt P027
3640.1.1	-2.3	4.33	1251.7096	-0.0026	2	34.59	kiak	46	YIPIQYVLSR	55	ypsy		sp|CASK_BOVIN|	Kappa-casein precursor
3648.1.1	-2	4.23	1251.7096	0.0009	2	34.59	kiak	46	YIPIQYVLSR	55	ypsy		sp|CASK_BOVIN|	Kappa-casein precursor
3652.1.1	-2.6	4.9	1215.6984	0.0003	2	36.72	pkpd	28	DLPFSTVVPLK	38	tfye		ENSP00000205948	Beta-2-glycoprotein 1 Precursor (Beta-2-glycoprotein I)(Beta(2)GPI)(B2GPI)(Apolipoprotein H)(Apo-H)(Activated protein C-binding protein)(APC inhibitor)(Anticardiolipin cofactor) [Source: UniProt P027
3656.1.1	-3.1	4.31	1956.0145	0.0053	3	39.7	iagr	22	TCPKPDDLPFSTVVPLK	38	tfye	C [23] 57.0215, T [22] 42.0106	ENSP00000205948	Beta-2-glycoprotein 1 Precursor (Beta-2-glycoprotein I)(Beta(2)GPI)(B2GPI)(Apolipoprotein H)(Apo-H)(Activated protein C-binding protein)(APC inhibitor)(Anticardiolipin cofactor) [Source: UniProt P027
3658.1.1	-3.2	5.17	1215.6984	-0.0002	2	36.72	pkpd	28	DLPFSTVVPLK	38	tfye		ENSP00000205948	Beta-2-glycoprotein 1 Precursor (Beta-2-glycoprotein I)(Beta(2)GPI)(B2GPI)(Apolipoprotein H)(Apo-H)(Activated protein C-binding protein)(APC inhibitor)(Anticardiolipin cofactor) [Source: UniProt P027
3669.1.1	-3.4	4.12	1330.7253	0.0004	2	38.76	cpkp	27	DDLPFSTVVPLK	38	tfye		ENSP00000205948	Beta-2-glycoprotein 1 Precursor (Beta-2-glycoprotein I)(Beta(2)GPI)(B2GPI)(Apolipoprotein H)(Apo-H)(Activated protein C-binding protein)(APC inhibitor)(Anticardiolipin cofactor) [Source: UniProt P027
3672.1.1	-9.3	6.22	1503.7632	0.0015	2	35.15	ctpr	83	VCPFAGILENGAVR	96	yttf	C [84] 57.0215, N [92] 0.9848	ENSP00000205948	Beta-2-glycoprotein 1 Precursor (Beta-2-glycoprotein I)(Beta(2)GPI)(B2GPI)(Apolipoprotein H)(Apo-H)(Activated protein C-binding protein)(APC inhibitor)(Anticardiolipin cofactor) [Source: UniProt P027
3677.1.1	-3.2	4.08	1330.7253	0.0037	2	38.76	cpkp	27	DDLPFSTVVPLK	38	tfye		ENSP00000205948	Beta-2-glycoprotein 1 Precursor (Beta-2-glycoprotein I)(Beta(2)GPI)(B2GPI)(Apolipoprotein H)(Apo-H)(Activated protein C-binding protein)(APC inhibitor)(Anticardiolipin cofactor) [Source: UniProt P027
3679.1.1	-10.4	6.38	1503.7632	-0.0014	2	35.15	ctpr	83	VCPFAGILENGAVR	96	yttf	C [84] 57.0215, N [92] 0.9848	ENSP00000205948	Beta-2-glycoprotein 1 Precursor (Beta-2-glycoprotein I)(Beta(2)GPI)(B2GPI)(Apolipoprotein H)(Apo-H)(Activated protein C-binding protein)(APC inhibitor)(Anticardiolipin cofactor) [Source: UniProt P027
3681.1.1	-1	4.4	1933.0616	-0.0016	3	50.69	ggmr	63	KFICPLTGLWPINTLK	78	ctpr	C [66] 57.0215, W [72] 31.9898	ENSP00000205948	Beta-2-glycoprotein 1 Precursor (Beta-2-glycoprotein I)(Beta(2)GPI)(B2GPI)(Apolipoprotein H)(Apo-H)(Activated protein C-binding protein)(APC inhibitor)(Anticardiolipin cofactor) [Source: UniProt P027
3685.1.1	-8.9	6.46	1503.7632	0.0006	2	35.15	ctpr	83	VCPFAGILENGAVR	96	yttf	C [84] 57.0215, N [92] 0.9848	ENSP00000205948	Beta-2-glycoprotein 1 Precursor (Beta-2-glycoprotein I)(Beta(2)GPI)(B2GPI)(Apolipoprotein H)(Apo-H)(Activated protein C-binding protein)(APC inhibitor)(Anticardiolipin cofactor) [Source: UniProt P027
3686.1.1	-3.7	5.65	1503.7632	0.0021	3	35.15	ctpr	83	VCPFAGILENGAVR	96	yttf	C [84] 57.0215, N [92] 0.9848	ENSP00000205948	Beta-2-glycoprotein 1 Precursor (Beta-2-glycoprotein I)(Beta(2)GPI)(B2GPI)(Apolipoprotein H)(Apo-H)(Activated protein C-binding protein)(APC inhibitor)(Anticardiolipin cofactor) [Source: UniProt P027
3687.1.1	-7.7	5.05	1427.7781	-0.0002	2	39.34	tcpk	26	PDDLPFSTVVPLK	38	tfye		ENSP00000205948	Beta-2-glycoprotein 1 Precursor (Beta-2-glycoprotein I)(Beta(2)GPI)(B2GPI)(Apolipoprotein H)(Apo-H)(Activated protein C-binding protein)(APC inhibitor)(Anticardiolipin cofactor) [Source: UniProt P027
3688.1.1	-5.1	5.18	1569.7107	0.0006	2	37.45	gavr	97	YTTFEYPNTISFS	109	cntg		ENSP00000205948	Beta-2-glycoprotein 1 Precursor (Beta-2-glycoprotein I)(Beta(2)GPI)(B2GPI)(Apolipoprotein H)(Apo-H)(Activated protein C-binding protein)(APC inhibitor)(Anticardiolipin cofactor) [Source: UniProt P027
3689.1.1	-5.1	5.04	1933.0616	0.0026	3	50.69	ggmr	63	KFICPLTGLWPINTLK	78	ctpr	C [66] 57.0215, W [72] 31.9898	ENSP00000205948	Beta-2-glycoprotein 1 Precursor (Beta-2-glycoprotein I)(Beta(2)GPI)(B2GPI)(Apolipoprotein H)(Apo-H)(Activated protein C-binding protein)(APC inhibitor)(Anticardiolipin cofactor) [Source: UniProt P027
3692.1.1	-5.7	5.42	1569.7107	0.0005	2	37.45	gavr	97	YTTFEYPNTISFS	109	cntg		ENSP00000205948	Beta-2-glycoprotein 1 Precursor (Beta-2-glycoprotein I)(Beta(2)GPI)(B2GPI)(Apolipoprotein H)(Apo-H)(Activated protein C-binding protein)(APC inhibitor)(Anticardiolipin cofactor) [Source: UniProt P027
3693.1.1	-4.8	5.56	1503.7632	0.0008	3	35.15	ctpr	83	VCPFAGILENGAVR	96	yttf	C [84] 57.0215, N [92] 0.9848	ENSP00000205948	Beta-2-glycoprotein 1 Precursor (Beta-2-glycoprotein I)(Beta(2)GPI)(B2GPI)(Apolipoprotein H)(Apo-H)(Activated protein C-binding protein)(APC inhibitor)(Anticardiolipin cofactor) [Source: UniProt P027
3695.1.1	-5.3	5.09	1933.0616	0.0006	3	50.69	ggmr	63	KFICPLTGLWPINTLK	78	ctpr	C [66] 57.0215, W [72] 31.9898	ENSP00000205948	Beta-2-glycoprotein 1 Precursor (Beta-2-glycoprotein I)(Beta(2)GPI)(B2GPI)(Apolipoprotein H)(Apo-H)(Activated protein C-binding protein)(APC inhibitor)(Anticardiolipin cofactor) [Source: UniProt P027
3699.1.1	-6.3	5.59	1569.7107	0.0005	2	37.45	gavr	97	YTTFEYPNTISFS	109	cntg		ENSP00000205948	Beta-2-glycoprotein 1 Precursor (Beta-2-glycoprotein I)(Beta(2)GPI)(B2GPI)(Apolipoprotein H)(Apo-H)(Activated protein C-binding protein)(APC inhibitor)(Anticardiolipin cofactor) [Source: UniProt P027
3700.1.1	-5.3	5.32	1503.7632	0.0011	3	35.15	ctpr	83	VCPFAGILENGAVR	96	yttf	C [84] 57.0215, N [92] 0.9848	ENSP00000205948	Beta-2-glycoprotein 1 Precursor (Beta-2-glycoprotein I)(Beta(2)GPI)(B2GPI)(Apolipoprotein H)(Apo-H)(Activated protein C-binding protein)(APC inhibitor)(Anticardiolipin cofactor) [Source: UniProt P027
3702.1.1	-7.3	4.68	1427.7781	-0.0004	2	39.34	tcpk	26	PDDLPFSTVVPLK	38	tfye		ENSP00000205948	Beta-2-glycoprotein 1 Precursor (Beta-2-glycoprotein I)(Beta(2)GPI)(B2GPI)(Apolipoprotein H)(Apo-H)(Activated protein C-binding protein)(APC inhibitor)(Anticardiolipin cofactor) [Source: UniProt P027
3728.1.1	-2.2	4.69	1411.7764	-0.0069	2	11.7	amgs	42	RQKAATEGPAQVR	54	aarp		ENSP00000321601	No description
3735.1.1	-2.3	4.94	1411.7764	-0.0077	2	11.7	amgs	42	RQKAATEGPAQVR	54	aarp		ENSP00000321601	No description
3737.1.1	-8.4	4.66	1661.8931	0.0057	2	41.5	ygkk	38	VMVLDFVTPTPLGTR	52	wglg	M [39] 15.9949	ENSP00000373506	Thioredoxin reductase 1, cytoplasmic (TR)(EC 1.8.1.9)(Thioredoxin reductase TR1)(KM-102-derived reductase-like factor)(Gene associated with retinoid-IFN-induced mortality 12 protein)(GRIM-12) [Sc
3739.1.1	-3.3	4.18	1119.5503	-0.0001	2	32.1	ctpr	83	VCPFAGILEN	92	gavr	C [84] 57.0215	ENSP00000205948	Beta-2-glycoprotein 1 Precursor (Beta-2-glycoprotein I)(Beta(2)GPI)(B2GPI)(Apolipoprotein H)(Apo-H)(Activated protein C-binding protein)(APC inhibitor)(Anticardiolipin cofactor) [Source: UniProt P027
3745.1.1	-8.3	4.83	1661.8931	0.0017	2	41.5	ygkk	38	VMVLDFVTPTPLGTR	52	wglg	M [39] 15.9949	ENSP00000373506	Thioredoxin reductase 1, cytoplasmic (TR)(EC 1.8.1.9)(Thioredoxin reductase TR1)(KM-102-derived reductase-like factor)(Gene associated with retinoid-IFN-induced mortality 12 protein)(GRIM-12) [Sc
3751.1.1	-2.1	5.29	4278.0774	0.0073	4	48.33	iagr	22	TCPKPDDLPFSTVVPLKTFYEPGEEI	58	ggmr	C [23] 57.0215, C [51] 57.0215	ENSP00000205948	Beta-2-glycoprotein 1 Precursor (Beta-2-glycoprotein I)(Beta(2)GPI)(B2GPI)(Apolipoprotein H)(Apo-H)(Activated protein C-binding protein)(APC inhibitor)(Anticardiolipin cofactor) [Source: UniProt P027
3752.1.1	-6.3	5	1661.8931	0.0017	2	41.5	ygkk	38	VMVLDFVTPTPLGTR	52	wglg	M [39] 15.9949	ENSP00000373506	Thioredoxin reductase 1, cytoplasmic (TR)(EC 1.8.1.9)(Thioredoxin reductase TR1)(KM-102-derived reductase-like factor)(Gene associated with retinoid-IFN-induced mortality 12 protein)(GRIM-12) [Sc
3758.1.1	-2.1	5.55	4278.0774	0.0024	4	48.33	iagr	22	TCPKPDDLPFSTVVPLKTFYEPGEEI	58	ggmr	C [23] 57.0215, C [51] 57.0215	ENSP00000205948	Beta-2-glycoprotein 1 Precursor (Beta-2-glycoprotein I)(Beta(2)GPI)(B2GPI)(Apolipoprotein H)(Apo-H)(Activated protein C-binding protein)(APC inhibitor)(Anticardiolipin cofactor) [Source: UniProt P027
3763.1.1	-2.8	5.66	4278.0774	0.0043	4	48.33	iagr	22	TCPKPDDLPFSTVVPLKTFYEPGEEI	58	ggmr	C [23] 57.0215, C [51] 57.0215	ENSP00000205948	Beta-2-glycoprotein 1 Precursor (Beta-2-glycoprotein I)(Beta(2)GPI)(B2GPI)(Apolipoprotein H)(Apo-H)(Activated protein C-binding protein)(APC inhibitor)(Anticardiolipin cofactor) [Source: UniProt P027
3781.1.1	-1.4	5.19	1503.7632	0.0008	2	35.15	ctpr	83	VCPFAGILENGAVR	96	yttf	C [84] 57.0215, N [92] 0.9848	ENSP00000205948	Beta-2-glycoprotein 1 Precursor (Beta-2-glycoprotein I)(Beta(2)GPI)(B2GPI)(Apolipoprotein H)(Apo-H)(Activated protein C-binding protein)(APC inhibitor)(Anticardiolipin cofactor) [Source: UniProt P027
3782.1.1	-2.7	5.47	2148.1182	-0.0018	3	41.88	kgqk	86	VGEFSGANKEKLEATINELV	105	]		ENSP00000363641	Thioredoxin (Trx)(ATL-derived factor)(ADF)(Surface-associated sulphydryl protein)(SASP) [Source: UniProt P10599]
3788.1.1	-1.6	5.61	2148.1182	-0.0042	3	41.88	kgqk	86	VGEFSGANKEKLEATINELV	105	]		ENSP00000363641	Thioredoxin (Trx)(ATL-derived factor)(ADF)(Surface-associated sulphydryl protein)(SASP) [Source: UniProt P10599]
3790.1.1	-2.2	4.55	2148.1182	0.0051	2	41.88	kgqk	86	VGEFSGANKEKLEATINELV	105	]		ENSP00000363641	Thioredoxin (Trx)(ATL-derived factor)(ADF)(Surface-associated sulphydryl protein)(SASP) [Source: UniProt P10599]
3791.1.1	-3	4.66	1851.0197	-0.0023	2	41.17	lpvc	138	APIICPPPSIPTFATLR	154	vykp	C [142] 57.0215	ENSP00000205948	Beta-2-glycoprotein 1 Precursor (Beta-2-glycoprotein I)(Beta(2)GPI)(B2GPI)(Apolipoprotein H)(Apo-H)(Activated protein C-binding protein)(APC inhibitor)(Anticardiolipin cofactor) [Source: UniProt P027
3795.1.1	-4.2	4.81	1851.0197	0.0005	2	41.17	lpvc	138	APIICPPPSIPTFATLR	154	vykp	C [142] 57.0215	ENSP00000205948	Beta-2-glycoprotein 1 Precursor (Beta-2-glycoprotein I)(Beta(2)GPI)(B2GPI)(Apolipoprotein H)(Apo-H)(Activated protein C-binding protein)(APC inhibitor)(Anticardiolipin cofactor) [Source: UniProt P027
3797.1.1	-2.1	4.49	2148.118	0.01	2	41.88	kgqk	86	VGEFSGANKEKLEATINELV	105	]		ENSP00000363641	Thioredoxin (Trx)(ATL-derived factor)(ADF)(Surface-associated sulphydryl protein)(SASP) [Source: UniProt P10599]
3801.1.1	-5.9	5.02	1851.0197	0.0032	2	41.17	lpvc	138	APIICPPPSIPTFATLR	154	vykp	C [142] 57.0215	ENSP00000205948	Beta-2-glycoprotein 1 Precursor (Beta-2-glycoprotein I)(Beta(2)GPI)(B2GPI)(Apolipoprotein H)(Apo-H)(Activated protein C-binding protein)(APC inhibitor)(Anticardiolipin cofactor) [Source: UniProt P027
3803.1.1	-2.1	4.24	2148.1182	0.01	2	41.88	kgqk	86	VGEFSGANKEKLEATINELV	105	]		ENSP00000363641	Thioredoxin (Trx)(ATL-derived factor)(ADF)(Surface-associated sulphydryl protein)(SASP) [Source: UniProt P10599]
3809.1.1	-2.5	4.51	1851.0197	0.0035	3	41.17	lpvc	138	APIICPPPSIPTFATLR	154	vykp	C [142] 57.0215	ENSP00000205948	Beta-2-glycoprotein 1 Precursor (Beta-2-glycoprotein I)(Beta(2)GPI)(B2GPI)(Apolipoprotein H)(Apo-H)(Activated protein C-binding protein)(APC inhibitor)(Anticardiolipin cofactor) [Source: UniProt P027
3815.1.1	-3.6	4.57	1851.0197	0.0002	3	41.17	lpvc	138	APIICPPPSIPTFATLR	154	vykp	C [142] 57.0215	ENSP00000205948	Beta-2-glycoprotein 1 Precursor (Beta-2-glycoprotein I)(Beta(2)GPI)(B2GPI)(Apolipoprotein H)(Apo-H)(Activated protein C-binding protein)(APC inhibitor)(Anticardiolipin cofactor) [Source: UniProt P027
3817.1.1	-5.4	4.04	1493.7425	0.0029	2	35.15	ctpr	83	VCPFAGILENGAVR	96	yttf	C [84] 57.0215, N [92] 0.9848, P [8	ENSP00000205948	Beta-2-glycoprotein 1 Precursor (Beta-2-glycoprotein I)(Beta(2)GPI)(B2GPI)(Apolipoprotein H)(Apo-H)(Activated protein C-binding protein)(APC inhibitor)(Anticardiolipin cofactor) [Source: UniProt P027
3821.1.1	-3.6	4.91	1851.0197	0.001	3	41.17	lpvc	138	APIICPPPSIPTFATLR	154	vykp	C [142] 57.0215	ENSP00000205948	Beta-2-glycoprotein 1 Precursor (Beta-2-glycoprotein I)(Beta(2)GPI)(B2GPI)(Apolipoprotein H)(Apo-H)(Activated protein C-binding protein)(APC inhibitor)(Anticardiolipin cofactor) [Source: UniProt P027
3829.1.1	-2.7	4.61	1779.9826	-0.0011	2	42.22	pvca	139	PIICPPPSIPTFATLR	154	vykp	C [142] 57.0215	ENSP00000205948	Beta-2-glycoprotein 1 Precursor (Beta-2-glycoprotein I)(Beta(2)GPI)(B2GPI)(Apolipoprotein H)(Apo-H)(Activated protein C-binding protein)(APC inhibitor)(Anticardiolipin cofactor) [Source: UniProt P027
3836.1.1	-2.9	4.69	1779.9826	0.0015	2	42.22	pvca	139	PIICPPPSIPTFATLR	154	vykp	C [142] 57.0215	ENSP00000205948	Beta-2-glycoprotein 1 Precursor (Beta-2-glycoprotein I)(Beta(2)GPI)(B2GPI)(Apolipoprotein H)(Apo-H)(Activated protein C-binding protein)(APC inhibitor)(Anticardiolipin cofactor) [Source: UniProt P027
3841.1.1	-8.9	5.09	2282.2292	-0.0012	3	44.25	dner	435	VVGFHVLGPNAGEVTQGFAAALK	457	cglt		ENSP00000373506	Thioredoxin reductase 1, cytoplasmic (TR)(EC 1.8.1.9)(Thioredoxin reductase TR1)(KM-102-derived reductase-like factor)(Gene associated with retinoid-IFN-induced mortality 12 protein)(GRIM-12) [Sc
3847.1.1	-11.4	5.19	2282.2292	-0.0026	3	44.25	dner	435	VVGFHVLGPNAGEVTQGFAAALK	457	cglt		ENSP00000373506	Thioredoxin reductase 1, cytoplasmic (TR)(EC 1.8.1.9)(Thioredoxin reductase TR1)(KM-102-derived reductase-like factor)(Gene associated with retinoid-IFN-induced mortality 12 protein)(GRIM-12) [Sc
3855.1.1	-9.3	5.06	2282.2292	-0.0019	3	44.25	dner	435	VVGFHVLGPNAGEVTQGFAAALK	457	cglt		ENSP00000373506	Thioredoxin reductase 1, cytoplasmic (TR)(EC 1.8.1.9)(Thioredoxin reductase TR1)(KM-102-derived reductase-like factor)(Gene associated with retinoid-IFN-induced mortality 12 protein)(GRIM-12) [Sc
3871.1.1	-2.2	4.74	1917.0667	0.0049	3	50.69	ggmr	63	KFICPLTGLWPINTLK	78	ctpr	C [66] 57.0215, W [72] 15.9949	ENSP00000205948	Beta-2-glycoprotein 1 Precursor (Beta-2-glycoprotein I)(Beta(2)GPI)(B2GPI)(Apolipoprotein H)(Apo-H)(Activated protein C-binding protein)(APC inhibitor)(Anticardiolipin cofactor) [Source: UniProt P027
3877.1.1	-3.9	4.64	1917.0667	0.0035	3	50.69	ggmr	63	KFICPLTGLWPINTLK	78	ctpr	C [66] 57.0215, W [72] 15.9949	ENSP00000205948	Beta-2-glycoprotein 1 Precursor (Beta-2-glycoprotein I)(Beta(2)GPI)(B2GPI)(Apolipoprotein H)(Apo-H)(Activated protein C-binding protein)(APC inhibitor)(Anticardiolipin cofactor) [Source: UniProt P027
3882.1.1	-1.2	5.17	1979.0219	0.0038	2	36.42	mkqk	1500	LLDEVIPPPDEEEVTVGK	1517	fyat		ENSP00000365427	
3885.1.1	-3.3	4.65	1917.0667	0.0014	3	50.69	ggmr	63	KFICPLTGLWPINTLK	78	ctpr	C [66] 57.0215, W [72] 15.9949	ENSP00000205948	Beta-2-glycoprotein 1 Precursor (Beta-2-glycoprotein I)(Beta(2)GPI)(B2GPI)(Apolipoprotein H)(Apo-H)(Activated protein C-binding protein)(APC inhibitor)(Anticardiolipin cofactor) [Source: UniProt P027
3892.1.1	-1.9	4.88	1503.7632	0.0045	2	35.15	ctpr	83	VCPFAGILENGAVR	96	yttf	C [84] 57.0215, N [92] 0.9848	ENSP00000205948	Beta-2-glycoprotein 1 Precursor (Beta-2-glycoprotein I)(Beta(2)GPI)(B2GPI)(Apolipoprotein H)(Apo-H)(Activated protein C-binding protein)(APC inhibitor)(Anticardiolipin cofactor) [Source: UniProt P027
3899.1.1	-2.8	4.85	1503.7632	0.0062	2	35.15	ctpr	83	VCPFAGILENGAVR	96	yttf	C [84] 57.0215, N [92] 0.9848	ENSP00000205948	Beta-2-glycoprotein 1 Precursor (Beta-2-glycoprotein I)(Beta(2)GPI)(B2GPI)(Apolipoprotein H)(Apo-H)(Activated protein C-binding protein)(APC inhibitor)(Anticardiolipin cofactor) [Source: UniProt P027
3904.1.1	-6.1	4.55	3068.3618	-0.0088	3	44.58	gavr	97	YTTFEYPNTISFSCNTGFYLNGADS/	123	ctee	C [110] 57.0215	ENSP00000205948	Beta-2-glycoprotein 1 Precursor (Beta-2-glycoprotein I)(Beta(2)GPI)(B2GPI)(Apolipoprotein H)(Apo-H)(Activated protein C-binding protein)(APC inhibitor)(Anticardiolipin cofactor) [Source: UniProt P027
3909.1.1	-7.3	4.7	3068.3618	0.0017	3	44.58	gavr	97	YTTFEYPNTISFSCNTGFYLNGADS/	123	ctee	C [110] 57.0215	ENSP00000205948	Beta-2-glycoprotein 1 Precursor (Beta-2-glycoprotein I)(Beta(2)GPI)(B2GPI)(Apolipoprotein H)(Apo-H)(Activated protein C-binding protein)(APC inhibitor)(Anticardiolipin cofactor) [Source: UniProt P027
3912.1.1	-3.6	4.28	1851.0197	0.0018	2	41.17	lpvc	138	APIICPPPSIPTFATLR	154	vykp	C [142] 57.0215	ENSP00000205948	Beta-2-glycoprotein 1 Precursor (Beta-2-glycoprotein I)(Beta(2)GPI)(B2GPI)(Apolipoprotein H)(Apo-H)(Activated protein C-binding protein)(APC inhibitor)(Anticardiolipin cofactor) [Source: UniProt P027
3914.1.1	-8.6	5.02	3068.3618	0.005	3	44.58	gavr	97	YTTFEYPNTISFSCNTGFYLNGADS/	123	ctee	C [110] 57.0215	ENSP00000205948	Beta-2-glycoprotein 1 Precursor (Beta-2-glycoprotein I)(Beta(2)GPI)(B2GPI)(Apolipoprotein H)(Apo-H)(Activated protein C-binding protein)(APC inhibitor)(Anticardiolipin cofactor) [Source: UniProt P027
3933.1.1	-13.4	4.86	3068.3618	0.0064	2	44.58	gavr	97	YTTFEYPNTISFSCNTGFYLNGADS/	123	ctee	C [110] 57.0215	ENSP00000205948	Beta-2-glycoprotein 1 Precursor (Beta-2-glycoprotein I)(Beta(2)GPI)(B2GPI)(Apolipoprotein H)(Apo-H)(Activated protein C-binding protein)(APC inhibitor)(Anticardiolipin cofactor) [Source: UniProt P027
3940.1.1	-12.8	4.48	3068.362	0.021	2	44.58	gavr	97	YTTFEYPNTISFSCNTGFYLNGADS/	123	ctee	C [110] 57.0215	ENSP00000205948	Beta-2-glycoprotein 1 Precursor (Beta-2-glycoprotein I)(Beta(2)GPI)(B2GPI)(Apolipoprotein H)(Apo-H)(Activated protein C-binding protein)(APC inhibitor)(Anticardiolipin cofactor) [Source: UniProt P027
3943.1.1	-2.5	5.08	1482.6788	-0.0021	2	37.42	gavr	97	YTTFEYPNTISF	108	scnt		ENSP00000205948	Beta-2-glycoprotein 1 Precursor (Beta-2-glycoprotein I)(Beta(2)GPI)(B2GPI)(Apolipoprotein H)(Apo-H)(Activated protein C-binding protein)(APC inhibitor)(Anticardiolipin cofactor) [Source: UniProt P027
3948.1.1	-3.4	5.01	1482.6788	-0.0009	2	37.42	gavr	97	YTTFEYPNTISF	108	scnt		ENSP00000205948	Beta-2-glycoprotein 1 Precursor (Beta-2-glycoprotein I)(Beta(2)GPI)(B2GPI)(Apolipoprotein H)(Apo-H)(Activated protein C-binding protein)(APC inhibitor)(Anticardiolipin cofactor) [Source: UniProt P027
3971.1.1	-3.7	4.64	1204.5166	0.0014	2	35.64	cevk	73	CMPTFQFFK	81	kgqk	C [73] 57.0215, M [74] 15.9949, C	ENSP00000363641	Thioredoxin (Trx)(ATL-derived factor)(ADF)(Surface-associated sulphydryl protein)(SASP) [Source: UniProt P10599]
3978.1.1	-4.6	4.8	1204.5166	0.0012	2	35.64	cevk	73	CMPTFQFFK	81	kgqk	C [73] 57.0215, M [74] 15.9949, C	ENSP00000363641	Thioredoxin (Trx)(ATL-derived factor)(ADF)(Surface-associated sulphydryl protein)(SASP) [Source: UniProt P10599]
3983.1.1	-4.1	4.96	1204.5166	-0.0028	2	35.64	cevk	73	CMPTFQFFK	81	kgqk	C [73] 57.0215, M [74] 15.9949, C	ENSP00000363641	Thioredoxin (Trx)(ATL-derived factor)(ADF)(Surface-associated sulphydryl protein)(SASP) [Source: UniProt P10599]
3988.1.1	-3.4	4.17	1352.7936	-0.0049	2	40.67	kfic	67	PLTGLWPINTLK	78	ctpr		ENSP00000205948	Beta-2-glycoprotein 1 Precursor (Beta-2-glycoprotein I)(Beta(2)GPI)(B2GPI)(Apolipoprotein H)(Apo-H)(Activated protein C-binding protein)(APC inhibitor)(Anticardiolipin cofactor) [Source: UniProt P027
3994.1.1	-3	4.19	1352.7936	0.0014	2	40.67	kfic	67	PLTGLWPINTLK	78	ctpr		ENSP00000205948	Beta-2-glycoprotein 1 Precursor (Beta-2-glycoprotein I)(Beta(2)GPI)(B2GPI)(Apolipoprotein H)(Apo-H)(Activated protein C-binding protein)(APC inhibitor)(Anticardiolipin cofactor) [Source: UniProt P027
3997.1.1	-7.4	5.32	3069.3466	-0.0033	3	44.58	gavr	97	YTTFEYPNTISFSCNTGFYLNGADS/	123	ctee	C [110] 57.0215, N [117] 0.9848	ENSP00000205948	Beta-2-glycoprotein 1 Precursor (Beta-2-glycoprotein I)(Beta(2)GPI)(B2GPI)(Apolipoprotein H)(Apo-H)(Activated protein C-binding protein)(APC inhibitor)(Anticardiolipin cofactor) [Source: UniProt P027
3998.1.1	-4.4	4.2	1352.7936	-0.0004	2	40.67	kfic	67	PLTGLWPINTLK	78	ctpr		ENSP00000205948	Beta-2-glycoprotein 1 Precursor (Beta-2-glycoprotein I)(Beta(2)GPI)(B2GPI)(Apolipoprotein H)(Apo-H)(Activated protein C-binding protein)(APC inhibitor)(Anticardiolipin cofactor) [Source: UniProt P027
4000.1.1	-10	5.28	3069.3466	0.0003	3	44.58	gavr	97	YTTFEYPNTISFSCNTGFYLNGADS/	123	ctee	C [110] 57.0215, N [117] 0.9848	ENSP00000205948	Beta-2-glycoprotein 1 Precursor (Beta-2-glycoprotein I)(Beta(2)GPI)(B2GPI)(Apolipoprotein H)(Apo-H)(Activated protein C-binding protein)(APC inhibitor)(Anticardiolipin cofactor) [Source: UniProt P027
4002.1.1	-1.1	4.34	1502.7784	-0.0046	2	35.15	ctpr	83	VCPFAGILENGAVR	96	yttf	C [84] 57.0215	ENSP00000205948	Beta-2-glycoprotein 1 Precursor (Beta-2-glycoprotein I)(Beta(2)GPI)(B2GPI)(Apolipoprotein H)(Apo-H)(Activated protein C-binding protein)(APC inhibitor)(Anticardiolipin cofactor) [Source: UniProt P027
4004.1.1	-4.5	5.17	3069.3466	-0.0028	3	44.58	gavr	97	YTTFEYPNTISFSCNTGFYLNGADS/	123	ctee	C [110] 57.0215, N [117] 0.9848	ENSP00000205948	Beta-2-glycoprotein 1 Precursor (Beta-2-glycoprotein I)(Beta(2)GPI)(B2GPI)(Apolipoprotein H)(Apo-H)(Activated protein C-binding protein)(APC inhibitor)(Anticardiolipin cofactor) [Source: UniProt P027
4007.1.1	-2	4.36	1502.7784	-0.0009	2	35.15	ctpr	83	VCPFAGILENGAVR	96	yttf	C [84] 57.0215	ENSP00000205948	Beta-2-glycoprotein 1 Precursor (Beta-2-glycoprotein I)(Beta(2)GPI)(B2GPI)(Apolipoprotein H)(Apo-H)(Activated protein C-binding protein)(APC inhibitor)(Anticardiolipin cofactor) [Source: UniProt P027
4020.1.1	-8.6	4.38	1645.8982	0.0015	2	41.5	ygkk	38	VMVLDFVTPTPLGTR	52	wglg		ENSP00000373506	Thioredoxin reductase 1, cytoplasmic (TR)(EC 1.8.1.9)(Thioredoxin reductase TR1)(KM-102-derived reductase-like factor)(Gene associated with retinoid-IFN-induced mortality 12 protein)(GRIM-12) [Sc
4027.1.1	-8.3	4.41	1645.8982	0.0033	2	41.5	ygkk	38	VMVLDFVTPTPLGTR	52	wglg		ENSP00000373506	Thioredoxin reductase 1, cytoplasmic (TR)(EC 1.8.1.9)(Thioredoxin reductase TR1)(KM-102-derived reductase-like factor)(Gene associated with retinoid-IFN-induced mortality 12 protein)(GRIM-12) [Sc
4034.1.1	-2.4	4.57	2994.39	0.0031	3	47.67	erpr	167	YLGIPGDKEYCISSDDLFSLPYCPGK	192	tlvv	C [177] 57.0215, C [189] 57.0215	ENSP00000373506	Thioredoxin reductase 1, cytoplasmic (TR)(EC 1.8.1.9)(Thioredoxin reductase TR1)(KM-102-derived reductase-like factor)(Gene associated with retinoid-IFN-induced mortality 12 protein)(GRIM-12) [Sc
4035.1.1	-5.6	4.05	1645.8982	0.0049	2	41.5	ygkk	38	VMVLDFVTPTPLGTR	52	wglg		ENSP00000373506	Thioredoxin reductase 1, cytoplasmic (TR)(EC 1.8.1.9)(Thioredoxin reductase TR1)(KM-102-derived reductase-like factor)(Gene associated with retinoid-IFN-induced mortality 12 protein)(GRIM-12) [Sc
4038.1.1	-5.1	4.69	2994.39	0.002	3	47.67	erpr	167	YLGIPGDKEYCISSDDLFSLPYCPGK	192	tlvv	C [177] 57.0215, C [189] 57.0215	ENSP00000373506	Thioredoxin reductase 1, cytoplasmic (TR)(EC 1.8.1.9)(Thioredoxin reductase TR1)(KM-102-derived reductase-like factor)(Gene associated with retinoid-IFN-induced mortality 12 protein)(GRIM-12) [Sc
4043.1.1	-9.2	5.1	3555.7321	0.0069	3	51.28	dsak	124	CTEEGKWSPELPVCAPIICPPPSIPT	154	vykp	C [124] 57.0215, C [137] 57.0215,	ENSP00000205948	Beta-2-glycoprotein 1 Precursor (Beta-2-glycoprotein I)(Beta(2)GPI)(B2GPI)(Apolipoprotein H)(Apo-H)(Activated protein C-binding protein)(APC inhibitor)(Anticardiolipin cofactor) [Source: UniProt P027
4044.1.1	-6.2	4.68	2994.39	0.0013	3	47.67	erpr	167	YLGIPGDKEYCISSDDLFSLPYCPGK	192	tlvv	C [177] 57.0215, C [189] 57.0215	ENSP00000373506	Thioredoxin reductase 1, cytoplasmic (TR)(EC 1.8.1.9)(Thioredoxin reductase TR1)(KM-102-derived reductase-like factor)(Gene associated with retinoid-IFN-induced mortality 12 protein)(GRIM-12) [Sc
4046.1.1	-7	4.44	1933.0616	0.0068	3	50.69	ggmr	63	KFICPLTGLWPINTLK	78	ctpr	C [66] 57.0215, W [72] 31.9898	ENSP00000205948	Beta-2-glycoprotein 1 Precursor (Beta-2-glycoprotein I)(Beta(2)GPI)(B2GPI)(Apolipoprotein H)(Apo-H)(Activated protein C-binding protein)(APC inhibitor)(Anticardiolipin cofactor) [Source: UniProt P027
4048.1.1	-2.9	4.14	1917.0667	0.0078	3	50.69	ggmr	63	KFICPLTGLWPINTLK	78	ctpr	C [66] 57.0215, W [72] 15.9949	ENSP00000205948	Beta-2-glycoprotein 1 Precursor (Beta-2-glycoprotein I)(Beta(2)GPI)(B2GPI)(Apolipoprotein H)(Apo-H)(Activated protein C-binding protein)(APC inhibitor)(Anticardiolipin cofactor) [Source: UniProt P027
4052.1.1	-8.1	4.56	1933.0616	0.0044	3	50.69	ggmr	63	KFICPLTGLWPINTLK	78	ctpr	C [66] 57.0215, W [72] 31.9898	ENSP00000205948	Beta-2-glycoprotein 1 Precursor (Beta-2-glycoprotein I)(Beta(2)GPI)(B2GPI)(Apolipoprotein H)(Apo-H)(Activated protein C-binding protein)(APC inhibitor)(Anticardiolipin cofactor) [Source: UniProt P027
4058.1.1	-8.3	5.11	3555.7321	0.0007	3	51.28	dsak	124	CTEEGKWSPELPVCAPIICPPPSIPT	154	vykp	C [124] 57.0215, C [137] 57.0215,	ENSP00000205948	Beta-2-glycoprotein 1 Precursor (Beta-2-glycoprotein I)(Beta(2)GPI)(B2GPI)(Apolipoprotein H)(Apo-H)(Activated protein C-binding protein)(APC inhibitor)(Anticardiolipin cofactor) [Source: UniProt P027
4061.1.1	-5.8	4.62	1933.0616	0.0024	3	50.69	ggmr	63	KFICPLTGLWPINTLK	78	ctpr	C [66] 57.0215, W [72] 31.9898	ENSP00000205948	Beta-2-glycoprotein 1 Precursor (Beta-2-glycoprotein I)(Beta(2)GPI)(B2GPI)(Apolipoprotein H)(Apo-H)(Activated protein C-binding protein)(APC inhibitor)(Anticardiolipin cofactor) [Source: UniProt P027
4067.1.1	-1.2	4.75	3555.7321	0.0037	4	51.28	dsak	124	CTEEGKWSPELPVCAPIICPPPSIPT	154	vykp	C [124] 57.0215, C [137] 57.0215,	ENSP00000205948	Beta-2-glycoprotein 1 Precursor (Beta-2-glycoprotein I)(Beta(2)GPI)(B2GPI)(Apolipoprotein H)(Apo-H)(Activated protein C-binding protein)(APC inhibitor)(Anticardiolipin cofactor) [Source: UniProt P027
4072.1.1	-9.8	5.2	3555.732	0.017	3	51.28	dsak	124	CTEEGKWSPELPVCAPIICPPPSIPT	154	vykp	C [124] 57.0215, C [137] 57.0215,	ENSP00000205948	Beta-2-glycoprotein 1 Precursor (Beta-2-glycoprotein I)(Beta(2)GPI)(B2GPI)(Apolipoprotein H)(Apo-H)(Activated protein C-binding protein)(APC inhibitor)(Anticardiolipin cofactor) [Source: UniProt P027
4074.1.1	-1.6	4.62	3555.7321	0.0032	4	51.28	dsak	124	CTEEGKWSPELPVCAPIICPPPSIPT	154	vykp	C [124] 57.0215, C [137] 57.0215,	ENSP00000205948	Beta-2-glycoprotein 1 Precursor (Beta-2-glycoprotein I)(Beta(2)GPI)(B2GPI)(Apolipoprotein H)(Apo-H)(Activated protein C-binding protein)(APC inhibitor)(Anticardiolipin cofactor) [Source: UniProt P027
4080.1.1	-4.7	4.76	3555.7321	-0.0059	4	51.28	dsak	124	CTEEGKWSPELPVCAPIICPPPSIPT	154	vykp	C [124] 57.0215, C [137] 57.0215,	ENSP00000205948	Beta-2-glycoprotein 1 Precursor (Beta-2-glycoprotein I)(Beta(2)GPI)(B2GPI)(Apolipoprotein H)(Apo-H)(Activated protein C-binding protein)(APC inhibitor)(Anticardiolipin cofactor) [Source: UniProt P027
4081.1.1	-4.4	4.42	1804.9666	0.0043	3	49.54	gmrk	64	FICPLTGLWPINTLK	78	ctpr	C [66] 57.0215, W [72] 31.9898	ENSP00000205948	Beta-2-glycoprotein 1 Precursor (Beta-2-glycoprotein I)(Beta(2)GPI)(B2GPI)(Apolipoprotein H)(Apo-H)(Activated protein C-binding protein)(APC inhibitor)(Anticardiolipin cofactor) [Source: UniProt P027
4086.1.1	-4.9	5.06	1804.9666	0.0034	3	49.54	gmrk	64	FICPLTGLWPINTLK	78	ctpr	C [66] 57.0215, W [72] 31.9898	ENSP00000205948	Beta-2-glycoprotein 1 Precursor (Beta-2-glycoprotein I)(Beta(2)GPI)(B2GPI)(Apolipoprotein H)(Apo-H)(Activated protein C-binding protein)(APC inhibitor)(Anticardiolipin cofactor) [Source: UniProt P027
4094.1.1	-6.1	4.4	1804.9666	0.0047	3	49.54	gmrk	64	FICPLTGLWPINTLK	78	ctpr	C [66] 57.0215, W [72] 31.9898	ENSP00000205948	Beta-2-glycoprotein 1 Precursor (Beta-2-glycoprotein I)(Beta(2)GPI)(B2GPI)(Apolipoprotein H)(Apo-H)(Activated protein C-binding protein)(APC inhibitor)(Anticardiolipin cofactor) [Source: UniProt P027
4110.1.1	-1.9	5.17	3539.7372	-0.0029	4	51.28	dsak	124	CTEEGKWSPELPVCAPIICPPPSIPT	154	vykp	C [124] 57.0215, C [137] 57.0215,	ENSP00000205948	Beta-2-glycoprotein 1 Precursor (Beta-2-glycoprotein I)(Beta(2)GPI)(B2GPI)(Apolipoprotein H)(Apo-H)(Activated protein C-binding protein)(APC inhibitor)(Anticardiolipin cofactor) [Source: UniProt P027

4117.1.1	-6.6	5.57	3539.7372	-0.0034	3	51.28	dsak	124	CTEEGKWSPELPVCAPIICPPPSIPT	154	vykp	C [124] 57.0215, C [137] 57.0215,	ENSP00000205948	Beta-2-glycoprotein 1 Precursor (Beta-2-glycoprotein I)(Beta(2)GPI)(B2GPI)(Apolipoprotein H)(Apo-H)(Activated protein C-binding protein)(APC inhibitor)(Anticardiolipin cofactor) [Source: UniProt P027
4118.1.1	-2.9	5.35	3539.7372	0.0015	4	51.28	dsak	124	CTEEGKWSPELPVCAPIICPPPSIPT	154	vykp	C [124] 57.0215, C [137] 57.0215,	ENSP00000205948	Beta-2-glycoprotein 1 Precursor (Beta-2-glycoprotein I)(Beta(2)GPI)(B2GPI)(Apolipoprotein H)(Apo-H)(Activated protein C-binding protein)(APC inhibitor)(Anticardiolipin cofactor) [Source: UniProt P027
4119.1.1	-4.3	4.9	1804.9666	0.0048	2	49.54	gmrk	64	FICPLTGLWPINTLK	78	ctpr	C [66] 57.0215, W [72] 31.9898	ENSP00000205948	Beta-2-glycoprotein 1 Precursor (Beta-2-glycoprotein I)(Beta(2)GPI)(B2GPI)(Apolipoprotein H)(Apo-H)(Activated protein C-binding protein)(APC inhibitor)(Anticardiolipin cofactor) [Source: UniProt P027
4120.1.1	-4.1	4.32	1329.726	-0.0003	2	35.62	dnsr	342	NLDLDSIIAEVK	353	aqye		ENSP00000310861	Keratin, type II cytoskeletal 2 epidermal (Cytokeratin-2e) (K2e) (CK 2e) (keratin-2). Source: Uniprot/SWISSPROT P35908
4121.1.1	-5.3	4.39	1302.7151	0.0006	2	36.09	dnnr	344	SLDLDSIIAEVK	355	aqye		ENSP00000252244	Keratin, type II cytoskeletal 1 (Cytokeratin-1) (CK-1) (Keratin-1) (K1) (67 kDa cytokeratin) (Hair alpha protein). Source: Uniprot/SWISSPROT P04264
4124.1.1	-5	5.48	3539.7372	-0.0056	3	51.28	dsak	124	CTEEGKWSPELPVCAPIICPPPSIPT	154	vykp	C [124] 57.0215, C [137] 57.0215,	ENSP00000205948	Beta-2-glycoprotein 1 Precursor (Beta-2-glycoprotein I)(Beta(2)GPI)(B2GPI)(Apolipoprotein H)(Apo-H)(Activated protein C-binding protein)(APC inhibitor)(Anticardiolipin cofactor) [Source: UniProt P027
4125.1.1	-4	4.77	1804.9666	0.0048	2	49.54	gmrk	64	FICPLTGLWPINTLK	78	ctpr	C [66] 57.0215, W [72] 31.9898	ENSP00000205948	Beta-2-glycoprotein 1 Precursor (Beta-2-glycoprotein I)(Beta(2)GPI)(B2GPI)(Apolipoprotein H)(Apo-H)(Activated protein C-binding protein)(APC inhibitor)(Anticardiolipin cofactor) [Source: UniProt P027
4126.1.1	-5.2	4.69	1302.7151	0.0001	2	36.09	dnnr	344	SLDLDSIIAEVK	355	aqye		ENSP00000252244	Keratin, type II cytoskeletal 1 (Cytokeratin-1) (CK-1) (Keratin-1) (K1) (67 kDa cytokeratin) (Hair alpha protein). Source: Uniprot/SWISSPROT P04264
4128.1.1	-4.1	4.47	1329.726	-0.0003	2	35.62	dnsr	342	NLDLDSIIAEVK	353	aqye		ENSP00000310861	Keratin, type II cytoskeletal 2 epidermal (Cytokeratin-2e) (K2e) (CK 2e) (keratin-2). Source: Uniprot/SWISSPROT P35908
4130.1.1	-9.6	5.36	3539.7372	0.0039	3	51.28	dsak	124	CTEEGKWSPELPVCAPIICPPPSIPT	154	vykp	C [124] 57.0215, C [137] 57.0215,	ENSP00000205948	Beta-2-glycoprotein 1 Precursor (Beta-2-glycoprotein I)(Beta(2)GPI)(B2GPI)(Apolipoprotein H)(Apo-H)(Activated protein C-binding protein)(APC inhibitor)(Anticardiolipin cofactor) [Source: UniProt P027
4131.1.1	-6.3	4.61	1302.7151	0.0025	2	36.09	dnnr	344	SLDLDSIIAEVK	355	aqye		ENSP00000252244	Keratin, type II cytoskeletal 1 (Cytokeratin-1) (CK-1) (Keratin-1) (K1) (67 kDa cytokeratin) (Hair alpha protein). Source: Uniprot/SWISSPROT P04264
4132.1.1	-4	4.57	1804.967	0.011	2	49.54	gmrk	64	FICPLTGLWPINTLK	78	ctpr	C [66] 57.0215, W [72] 31.9898	ENSP00000205948	Beta-2-glycoprotein 1 Precursor (Beta-2-glycoprotein I)(Beta(2)GPI)(B2GPI)(Apolipoprotein H)(Apo-H)(Activated protein C-binding protein)(APC inhibitor)(Anticardiolipin cofactor) [Source: UniProt P027
4133.1.1	-2.9	4.59	3506.716	1.008	3	51.28	dsak	124	CTEEGKWSPELPVCAPIICPPPSIPT	154	vykp	C [124] 57.0215, C [137] 57.0215,	ENSP00000205948	Beta-2-glycoprotein 1 Precursor (Beta-2-glycoprotein I)(Beta(2)GPI)(B2GPI)(Apolipoprotein H)(Apo-H)(Activated protein C-binding protein)(APC inhibitor)(Anticardiolipin cofactor) [Source: UniProt P027
4134.1.1	-3.7	3.95	1329.726	0.0007	2	35.62	dnsr	342	NLDLDSIIAEVK	353	aqye		ENSP00000310861	Keratin, type II cytoskeletal 2 epidermal (Cytokeratin-2e) (K2e) (CK 2e) (keratin-2). Source: Uniprot/SWISSPROT P35908
4135.1.1	-2.4	4.24	1502.7784	0.0024	2	35.15	ctpr	83	VCPFAGILENGAVR	96	yttf	C [84] 57.0215	ENSP00000205948	Beta-2-glycoprotein 1 Precursor (Beta-2-glycoprotein I)(Beta(2)GPI)(B2GPI)(Apolipoprotein H)(Apo-H)(Activated protein C-binding protein)(APC inhibitor)(Anticardiolipin cofactor) [Source: UniProt P027
4155.1.1	-2.3	4.19	1503.7632	0.0033	2	35.15	ctpr	83	VCPFAGILENGAVR	96	yttf	C [84] 57.0215, N [92] 0.9848	ENSP00000205948	Beta-2-glycoprotein 1 Precursor (Beta-2-glycoprotein I)(Beta(2)GPI)(B2GPI)(Apolipoprotein H)(Apo-H)(Activated protein C-binding protein)(APC inhibitor)(Anticardiolipin cofactor) [Source: UniProt P027
4164.1.1	-4.6	5.22	3555.7321	0.0095	3	51.28	dsak	124	CTEEGKWSPELPVCAPIICPPPSIPT	154	vykp	C [124] 57.0215, C [137] 57.0215,	ENSP00000205948	Beta-2-glycoprotein 1 Precursor (Beta-2-glycoprotein I)(Beta(2)GPI)(B2GPI)(Apolipoprotein H)(Apo-H)(Activated protein C-binding protein)(APC inhibitor)(Anticardiolipin cofactor) [Source: UniProt P027
4166.1.1	-1.4	4.85	3555.732	1.006	4	51.28	dsak	124	CTEEGKWSPELPVCAPIICPPPSIPT	154	vykp	C [124] 57.0215, C [137] 57.0215,	ENSP00000205948	Beta-2-glycoprotein 1 Precursor (Beta-2-glycoprotein I)(Beta(2)GPI)(B2GPI)(Apolipoprotein H)(Apo-H)(Activated protein C-binding protein)(APC inhibitor)(Anticardiolipin cofactor) [Source: UniProt P027
4171.1.1	-2.6	5.06	3555.7321	0.0047	3	51.28	dsak	124	CTEEGKWSPELPVCAPIICPPPSIPT	154	vykp	C [124] 57.0215, C [137] 57.0215,	ENSP00000205948	Beta-2-glycoprotein 1 Precursor (Beta-2-glycoprotein I)(Beta(2)GPI)(B2GPI)(Apolipoprotein H)(Apo-H)(Activated protein C-binding protein)(APC inhibitor)(Anticardiolipin cofactor) [Source: UniProt P027
4172.1.1	-2.6	5.37	3527.701	0.034	3	51.28	dsak	124	CTEEGKWSPELPVCAPIICPPPSIPT	154	vykp	C [124] 57.0215, C [137] 57.0215,	ENSP00000205948	Beta-2-glycoprotein 1 Precursor (Beta-2-glycoprotein I)(Beta(2)GPI)(B2GPI)(Apolipoprotein H)(Apo-H)(Activated protein C-binding protein)(APC inhibitor)(Anticardiolipin cofactor) [Source: UniProt P027
4178.1.1	-1.5	4.85	1901.0717	-0.0012	3	50.69	ggmr	63	KFICPLTGLWPINTLK	78	ctpr	C [66] 57.0215	ENSP00000205948	Beta-2-glycoprotein 1 Precursor (Beta-2-glycoprotein I)(Beta(2)GPI)(B2GPI)(Apolipoprotein H)(Apo-H)(Activated protein C-binding protein)(APC inhibitor)(Anticardiolipin cofactor) [Source: UniProt P027
4184.1.1	-4.1	5.23	1901.0717	0.0017	3	50.69	ggmr	63	KFICPLTGLWPINTLK	78	ctpr	C [66] 57.0215	ENSP00000205948	Beta-2-glycoprotein 1 Precursor (Beta-2-glycoprotein I)(Beta(2)GPI)(B2GPI)(Apolipoprotein H)(Apo-H)(Activated protein C-binding protein)(APC inhibitor)(Anticardiolipin cofactor) [Source: UniProt P027
4187.1.1	-2.2	4.45	1788.9717	0.006	2	49.54	gmrk	64	FICPLTGLWPINTLK	78	ctpr	C [66] 57.0215, W [72] 15.9949	ENSP00000205948	Beta-2-glycoprotein 1 Precursor (Beta-2-glycoprotein I)(Beta(2)GPI)(B2GPI)(Apolipoprotein H)(Apo-H)(Activated protein C-binding protein)(APC inhibitor)(Anticardiolipin cofactor) [Source: UniProt P027
4192.1.1	-1.8	4.04	1917.0667	0.0067	3	50.69	ggmr	63	KFICPLTGLWPINTLK	78	ctpr	C [66] 57.0215, W [72] 15.9949	ENSP00000205948	Beta-2-glycoprotein 1 Precursor (Beta-2-glycoprotein I)(Beta(2)GPI)(B2GPI)(Apolipoprotein H)(Apo-H)(Activated protein C-binding protein)(APC inhibitor)(Anticardiolipin cofactor) [Source: UniProt P027
4198.1.1	-4.7	4.64	1788.9717	0.0098	2	49.54	gmrk	64	FICPLTGLWPINTLK	78	ctpr	C [66] 57.0215, W [72] 15.9949	ENSP00000205948	Beta-2-glycoprotein 1 Precursor (Beta-2-glycoprotein I)(Beta(2)GPI)(B2GPI)(Apolipoprotein H)(Apo-H)(Activated protein C-binding protein)(APC inhibitor)(Anticardiolipin cofactor) [Source: UniProt P027
4201.1.1	-7.6	4.81	1788.9717	0.0027	2	49.54	gmrk	64	FICPLTGLWPINTLK	78	ctpr	C [66] 57.0215, W [72] 15.9949	ENSP00000205948	Beta-2-glycoprotein 1 Precursor (Beta-2-glycoprotein I)(Beta(2)GPI)(B2GPI)(Apolipoprotein H)(Apo-H)(Activated protein C-binding protein)(APC inhibitor)(Anticardiolipin cofactor) [Source: UniProt P027
4202.1.1	-6.4	4.44	1901.0717	0.0047	2	50.69	ggmr	63	KFICPLTGLWPINTLK	78	ctpr	C [66] 57.0215	ENSP00000205948	Beta-2-glycoprotein 1 Precursor (Beta-2-glycoprotein I)(Beta(2)GPI)(B2GPI)(Apolipoprotein H)(Apo-H)(Activated protein C-binding protein)(APC inhibitor)(Anticardiolipin cofactor) [Source: UniProt P027
4203.1.1	-4	4.2	1788.9717	-0.0009	3	49.54	gmrk	64	FICPLTGLWPINTLK	78	ctpr	C [66] 57.0215, W [72] 15.9949	ENSP00000205948	Beta-2-glycoprotein 1 Precursor (Beta-2-glycoprotein I)(Beta(2)GPI)(B2GPI)(Apolipoprotein H)(Apo-H)(Activated protein C-binding protein)(APC inhibitor)(Anticardiolipin cofactor) [Source: UniProt P027
4205.1.1	-6.5	4.56	1901.0717	0.0009	2	50.69	ggmr	63	KFICPLTGLWPINTLK	78	ctpr	C [66] 57.0215	ENSP00000205948	Beta-2-glycoprotein 1 Precursor (Beta-2-glycoprotein I)(Beta(2)GPI)(B2GPI)(Apolipoprotein H)(Apo-H)(Activated protein C-binding protein)(APC inhibitor)(Anticardiolipin cofactor) [Source: UniProt P027
4207.1.1	-3.9	4.39	1788.9717	-0.0016	3	49.54	gmrk	64	FICPLTGLWPINTLK	78	ctpr	C [66] 57.0215, W [72] 15.9949	ENSP00000205948	Beta-2-glycoprotein 1 Precursor (Beta-2-glycoprotein I)(Beta(2)GPI)(B2GPI)(Apolipoprotein H)(Apo-H)(Activated protein C-binding protein)(APC inhibitor)(Anticardiolipin cofactor) [Source: UniProt P027
4209.1.1	-3.7	4.29	1503.7632	0.0087	2	35.15	ctpr	83	VCPFAGILENGAVR	96	yttf	C [84] 57.0215, N [92] 0.9848	ENSP00000205948	Beta-2-glycoprotein 1 Precursor (Beta-2-glycoprotein I)(Beta(2)GPI)(B2GPI)(Apolipoprotein H)(Apo-H)(Activated protein C-binding protein)(APC inhibitor)(Anticardiolipin cofactor) [Source: UniProt P027
4212.1.1	-3.2	4.51	1901.0717	0.0069	2	50.69	ggmr	63	KFICPLTGLWPINTLK	78	ctpr	C [66] 57.0215	ENSP00000205948	Beta-2-glycoprotein 1 Precursor (Beta-2-glycoprotein I)(Beta(2)GPI)(B2GPI)(Apolipoprotein H)(Apo-H)(Activated protein C-binding protein)(APC inhibitor)(Anticardiolipin cofactor) [Source: UniProt P027
4214.1.1	-4	4.29	1788.9717	0.0044	3	49.54	gmrk	64	FICPLTGLWPINTLK	78	ctpr	C [66] 57.0215, W [72] 15.9949	ENSP00000205948	Beta-2-glycoprotein 1 Precursor (Beta-2-glycoprotein I)(Beta(2)GPI)(B2GPI)(Apolipoprotein H)(Apo-H)(Activated protein C-binding protein)(APC inhibitor)(Anticardiolipin cofactor) [Source: UniProt P027
4225.1.1	-4.2	3.99	1502.7784	-0.0011	2	35.15	ctpr	83	VCPFAGILENGAVR	96	yttf	C [84] 57.0215	ENSP00000205948	Beta-2-glycoprotein 1 Precursor (Beta-2-glycoprotein I)(Beta(2)GPI)(B2GPI)(Apolipoprotein H)(Apo-H)(Activated protein C-binding protein)(APC inhibitor)(Anticardiolipin cofactor) [Source: UniProt P027
4238.1.1	-6	5.32	2633.3828	0.0034	3	50.72	egkw	131	SPELPVCAPIICPPPSIPTFATLR	154	vykp	C [137] 57.0215, C [142] 57.0215	ENSP00000205948	Beta-2-glycoprotein 1 Precursor (Beta-2-glycoprotein I)(Beta(2)GPI)(B2GPI)(Apolipoprotein H)(Apo-H)(Activated protein C-binding protein)(APC inhibitor)(Anticardiolipin cofactor) [Source: UniProt P027
4241.1.1	-2.4	4.17	1502.7784	0.0048	2	35.15	ctpr	83	VCPFAGILENGAVR	96	yttf	C [84] 57.0215	ENSP00000205948	Beta-2-glycoprotein 1 Precursor (Beta-2-glycoprotein I)(Beta(2)GPI)(B2GPI)(Apolipoprotein H)(Apo-H)(Activated protein C-binding protein)(APC inhibitor)(Anticardiolipin cofactor) [Source: UniProt P027
4243.1.1	-4.9	5.48	2633.3828	0.0021	3	50.72	egkw	131	SPELPVCAPIICPPPSIPTFATLR	154	vykp	C [137] 57.0215, C [142] 57.0215	ENSP00000205948	Beta-2-glycoprotein 1 Precursor (Beta-2-glycoprotein I)(Beta(2)GPI)(B2GPI)(Apolipoprotein H)(Apo-H)(Activated protein C-binding protein)(APC inhibitor)(Anticardiolipin cofactor) [Source: UniProt P027
4244.1.1	-6.2	4.88	2633.3828	0.0064	2	50.72	egkw	131	SPELPVCAPIICPPPSIPTFATLR	154	vykp	C [137] 57.0215, C [142] 57.0215	ENSP00000205948	Beta-2-glycoprotein 1 Precursor (Beta-2-glycoprotein I)(Beta(2)GPI)(B2GPI)(Apolipoprotein H)(Apo-H)(Activated protein C-binding protein)(APC inhibitor)(Anticardiolipin cofactor) [Source: UniProt P027
4246.1.1	-2.1	4.93	2287.209	-0.0036	3	50.73	gmrk	64	FICPLTGLWPINTLKCTPR	82	vcpf	C [66] 57.0215, C [79] 57.0215	ENSP00000205948	Beta-2-glycoprotein 1 Precursor (Beta-2-glycoprotein I)(Beta(2)GPI)(B2GPI)(Apolipoprotein H)(Apo-H)(Activated protein C-binding protein)(APC inhibitor)(Anticardiolipin cofactor) [Source: UniProt P027
4249.1.1	-6.1	5.71	2633.3828	0.0035	3	50.72	egkw	131	SPELPVCAPIICPPPSIPTFATLR	154	vykp	C [137] 57.0215, C [142] 57.0215	ENSP00000205948	Beta-2-glycoprotein 1 Precursor (Beta-2-glycoprotein I)(Beta(2)GPI)(B2GPI)(Apolipoprotein H)(Apo-H)(Activated protein C-binding protein)(APC inhibitor)(Anticardiolipin cofactor) [Source: UniProt P027
4250.1.1	-3.6	5.59	3523.7422	0.004	4	51.28	dsak	124	CTEEGKWSPELPVCAPIICPPPSIPT	154	vykp	C [124] 57.0215, C [137] 57.0215,	ENSP00000205948	Beta-2-glycoprotein 1 Precursor (Beta-2-glycoprotein I)(Beta(2)GPI)(B2GPI)(Apolipoprotein H)(Apo-H)(Activated protein C-binding protein)(APC inhibitor)(Anticardiolipin cofactor) [Source: UniProt P027
4252.1.1	-7.1	5.15	2633.3828	0.0071	2	50.72	egkw	131	SPELPVCAPIICPPPSIPTFATLR	154	vykp	C [137] 57.0215, C [142] 57.0215	ENSP00000205948	Beta-2-glycoprotein 1 Precursor (Beta-2-glycoprotein I)(Beta(2)GPI)(B2GPI)(Apolipoprotein H)(Apo-H)(Activated protein C-binding protein)(APC inhibitor)(Anticardiolipin cofactor) [Source: UniProt P027
4258.1.1	-7.2	5.08	2633.3828	0.0066	2	50.72	egkw	131	SPELPVCAPIICPPPSIPTFATLR	154	vykp	C [137] 57.0215, C [142] 57.0215	ENSP00000205948	Beta-2-glycoprotein 1 Precursor (Beta-2-glycoprotein I)(Beta(2)GPI)(B2GPI)(Apolipoprotein H)(Apo-H)(Activated protein C-binding protein)(APC inhibitor)(Anticardiolipin cofactor) [Source: UniProt P027
4263.1.1	-5.8	5.83	3523.7422	-0.0013	3	51.28	dsak	124	CTEEGKWSPELPVCAPIICPPPSIPT	154	vykp	C [124] 57.0215, C [137] 57.0215,	ENSP00000205948	Beta-2-glycoprotein 1 Precursor (Beta-2-glycoprotein I)(Beta(2)GPI)(B2GPI)(Apolipoprotein H)(Apo-H)(Activated protein C-binding protein)(APC inhibitor)(Anticardiolipin cofactor) [Source: UniProt P027
4269.1.1	-4.7	5.84	1901.0717	0.0004	3	50.69	ggmr	63	KFICPLTGLWPINTLK	78	ctpr	C [66] 57.0215	ENSP00000205948	Beta-2-glycoprotein 1 Precursor (Beta-2-glycoprotein I)(Beta(2)GPI)(B2GPI)(Apolipoprotein H)(Apo-H)(Activated protein C-binding protein)(APC inhibitor)(Anticardiolipin cofactor) [Source: UniProt P027
4275.1.1	-6.7	5.86	1901.0717	0.0012	3	50.69	ggmr	63	KFICPLTGLWPINTLK	78	ctpr	C [66] 57.0215	ENSP00000205948	Beta-2-glycoprotein 1 Precursor (Beta-2-glycoprotein I)(Beta(2)GPI)(B2GPI)(Apolipoprotein H)(Apo-H)(Activated protein C-binding protein)(APC inhibitor)(Anticardiolipin cofactor) [Source: UniProt P027
4276.1.1	-5	5.68	3523.7422	-0.0024	3	51.28	dsak	124	CTEEGKWSPELPVCAPIICPPPSIPT	154	vykp	C [124] 57.0215, C [137] 57.0215,	ENSP00000205948	Beta-2-glycoprotein 1 Precursor (Beta-2-glycoprotein I)(Beta(2)GPI)(B2GPI)(Apolipoprotein H)(Apo-H)(Activated protein C-binding protein)(APC inhibitor)(Anticardiolipin cofactor) [Source: UniProt P027
4279.1.1	-2.7	4.93	2287.209	-0.0023	3	50.73	gmrk	64	FICPLTGLWPINTLKCTPR	82	vcpf	C [66] 57.0215, C [79] 57.0215	ENSP00000205948	Beta-2-glycoprotein 1 Precursor (Beta-2-glycoprotein I)(Beta(2)GPI)(B2GPI)(Apolipoprotein H)(Apo-H)(Activated protein C-binding protein)(APC inhibitor)(Anticardiolipin cofactor) [Source: UniProt P027
4280.1.1	-8.8	4.87	1384.73	0.0004	2	42.55	nllr	38	FFVAPFPEVFGK	49	ekvn		sp|CAS1_BOVIN|	Alpha-S1-casein; Contains: Antioxidant peptide; Flags: Precursor;
4282.1.1	-7.3	4.94	1384.73	0.0005	2	42.55	nllr	38	FFVAPFPEVFGK	49	ekvn		sp|CAS1_BOVIN|	Alpha-S1-casein; Contains: Antioxidant peptide; Flags: Precursor;
4284.1.1	-3	4.97	2287.209	-0.0022	3	50.73	gmrk	64	FICPLTGLWPINTLKCTPR	82	vcpf	C [66] 57.0215, C [79] 57.0215	ENSP00000205948	Beta-2-glycoprotein 1 Precursor (Beta-2-glycoprotein I)(Beta(2)GPI)(B2GPI)(Apolipoprotein H)(Apo-H)(Activated protein C-binding protein)(APC inhibitor)(Anticardiolipin cofactor) [Source: UniProt P027
4285.1.1	-4.8	4.04	2546.3506	-0.0067	3	50.34	gkws	132	PELPVCAPIICPPPSIPTFATLR	154	vykp	C [137] 57.0215, C [142] 57.0215	ENSP00000205948	Beta-2-glycoprotein 1 Precursor (Beta-2-glycoprotein I)(Beta(2)GPI)(B2GPI)(Apolipoprotein H)(Apo-H)(Activated protein C-binding protein)(APC inhibitor)(Anticardiolipin cofactor) [Source: UniProt P027
4289.1.1	-6.2	5.1	1788.9717	0.003	2	49.54	gmrk	64	FICPLTGLWPINTLK	78	ctpr	C [66] 57.0215, W [72] 15.9949	ENSP00000205948	Beta-2-glycoprotein 1 Precursor (Beta-2-glycoprotein I)(Beta(2)GPI)(B2GPI)(Apolipoprotein H)(Apo-H)(Activated protein C-binding protein)(APC inhibitor)(Anticardiolipin cofactor) [Source: UniProt P027
4290.1.1	-8	5.12	1384.73	-0.0003	2	42.55	nllr	38	FFVAPFPEVFGK	49	ekvn		sp|CAS1_BOVIN|	Alpha-S1-casein; Contains: Antioxidant peptide; Flags: Precursor;
4294.1.1	-6.5	4.98	1788.9717	0.0002	2	49.54	gmrk	64	FICPLTGLWPINTLK	78	ctpr	C [66] 57.0215, W [72] 15.9949	ENSP00000205948	Beta-2-glycoprotein 1 Precursor (Beta-2-glycoprotein I)(Beta(2)GPI)(B2GPI)(Apolipoprotein H)(Apo-H)(Activated protein C-binding protein)(APC inhibitor)(Anticardiolipin cofactor) [Source: UniProt P027
4295.1.1	-5.9	4.62	1901.0717	0.0034	2	50.69	ggmr	63	KFICPLTGLWPINTLK	78	ctpr	C [66] 57.0215	ENSP00000205948	Beta-2-glycoprotein 1 Precursor (Beta-2-glycoprotein I)(Beta(2)GPI)(B2GPI)(Apolipoprotein H)(Apo-H)(Activated protein C-binding protein)(APC inhibitor)(Anticardiolipin cofactor) [Source: UniProt P027
4296.1.1	-5	4.81	2287.209	0.0006	3	50.73	gmrk	64	FICPLTGLWPINTLKCTPR	82	vcpf	C [66] 57.0215, C [79] 57.0215	ENSP00000205948	Beta-2-glycoprotein 1 Precursor (Beta-2-glycoprotein I)(Beta(2)GPI)(B2GPI)(Apolipoprotein H)(Apo-H)(Activated protein C-binding protein)(APC inhibitor)(Anticardiolipin cofactor) [Source: UniProt P027
4297.1.1	-3.5	4.48	1788.9717	-0.0007	3	49.54	gmrk	64	FICPLTGLWPINTLK	78	ctpr	C [66] 57.0215, W [72] 15.9949	ENSP00000205948	Beta-2-glycoprotein 1 Precursor (Beta-2-glycoprotein I)(Beta(2)GPI)(B2GPI)(Apolipoprotein H)(Apo-H)(Activated protein C-binding protein)(APC inhibitor)(Anticardiolipin cofactor) [Source: UniProt P027
4300.1.1	-4.6	4.95	1788.9717	0.0013	2	49.54	gmrk	64	FICPLTGLWPINTLK	78	ctpr	C [66] 57.0215, W [72] 15.9949	ENSP00000205948	Beta-2-glycoprotein 1 Precursor (Beta-2-glycoprotein I)(Beta(2)GPI)(B2GPI)(Apolipoprotein H)(Apo-H)(Activated protein C-binding protein)(APC inhibitor)(Anticardiolipin cofactor) [Source: UniProt P027
4301.1.1	-5.3	4.52	1901.0717	0.0027	2	50.69	ggmr	63	KFICPLTGLWPINTLK	78	ctpr	C [66] 57.0215	ENSP00000205948	Beta-2-glycoprotein 1 Precursor (Beta-2-glycoprotein I)(Beta(2)GPI)(B2GPI)(Apolipoprotein H)(Apo-H)(Activated protein C-binding protein)(APC inhibitor)(Anticardiolipin cofactor) [Source: UniProt P027
4302.1.1	-4	4.57	1788.9717	-0.0027	3	49.54	gmrk	64	FICPLTGLWPINTLK	78	ctpr	C [66] 57.0215, W [72] 15.9949	ENSP00000205948	Beta-2-glycoprotein 1 Precursor (Beta-2-glycoprotein I)(Beta(2)GPI)(B2GPI)(Apolipoprotein H)(Apo-H)(Activated protein C-binding protein)(APC inhibitor)(Anticardiolipin cofactor) [Source: UniProt P027
4304.1.1	-4.3	4.32	1993.9767	-0.0043	3	43.17	tstr	224	THNLEPYFESFINNLR	239	rrvd		ENSP00000252244	Keratin, type II cytoskeletal 1 (Cytokeratin-1) (CK-1) (Keratin-1) (K1) (67 kDa cytokeratin) (Hair alpha protein). Source: Uniprot/SWISSPROT P04264
4306.1.1	-6.7	4.53	1901.0717	0.0025	2	50.69	ggmr	63	KFICPLTGLWPINTLK	78	ctpr	C [66] 57.0215	ENSP00000205948	Beta-2-glycoprotein 1 Precursor (Beta-2-glycoprotein I)(Beta(2)GPI)(B2GPI)(Apolipoprotein H)(Apo-H)(Activated protein C-binding protein)(APC inhibitor)(Anticardiolipin cofactor) [Source: UniProt P027
4311.1.1	-5.1	4.4	1788.9717	0.0026	3	49.54	gmrk	64	FICPLTGLWPINTLK	78	ctpr	C [66] 57.0215, W [72] 15.9949	ENSP00000205948	Beta-2-glycoprotein 1 Precursor (Beta-2-glycoprotein I)(Beta(2)GPI)(B2GPI)(Apolipoprotein H)(Apo-H)(Activated protein C-binding protein)(APC inhibitor)(Anticardiolipin cofactor) [Source: UniProt P027
4312.1.1	-4	4.3	1993.9767	0.0047	3	43.17	tstr	224	THNLEPYFESFINNLR	239	rrvd		ENSP00000252244	Keratin, type II cytoskeletal 1 (Cytokeratin-1) (CK-1) (Keratin-1) (K1) (67 kDa cytokeratin) (Hair alpha protein). Source: Uniprot/SWISSPROT P04264
4317.1.1	-9.4	5.41	3538.706	0.011	3	51.28	dsak	124	CTEEGKWSPELPVCAPIICPPPSIPT	154	vykp	C [124] 57.0215, C [137] 57.0215,	ENSP00000205948	Beta-2-glycoprotein 1 Precursor (Beta-2-glycoprotein I)(Beta(2)GPI)(B2GPI)(Apolipoprotein H)(Apo-H)(Activated protein C-binding protein)(APC inhibitor)(Anticardiolipin cofactor) [Source: UniProt P027
4322.1.1	-7.4	5.38	3538.7055	0.0043	3	51.28	dsak	124	CTEEGKWSPELPVCAPIICPPPSIPT	154	vykp	C [124] 57.0215, C [137] 57.0215,	ENSP00000205948	Beta-2-glycoprotein 1 Precursor (Beta-2-glycoprotein I)(Beta(2)GPI)(B2GPI)(Apolipoprotein H)(Apo-H)(Activated protein C-binding protein)(APC inhibitor)(Anticardiolipin cofactor) [Source: UniProt P027
4326.1.1	-9.1	5.52	3538.7055	0.0017	3	51.28	dsak	124	CTEEGKWSPELPVCAPIICPPPSIPT	154	vykp	C [124] 57.0215, C [137] 57.0215,	ENSP00000205948	Beta-2-glycoprotein 1 Precursor (Beta-2-glycoprotein I)(Beta(2)GPI)(B2GPI)(Apolipoprotein H)(Apo-H)(Activated protein C-binding protein)(APC inhibitor)(Anticardiolipin cofactor) [Source: UniProt P027
4351.1.1	-5.5	3.92	2068.9718	0.0063	2	42.62	dsak	124	CTEEGKWSPELPVCAPII	141	cppp	C [124] 57.0215, C [137] 57.0215,	ENSP00000205948	Beta-2-glycoprotein 1 Precursor (Beta-2-glycoprotein I)(Beta(2)GPI)(B2GPI)(Apolipoprotein H)(Apo-H)(Activated protein C-binding protein)(APC inhibitor)(Anticardiolipin cofactor) [Source: UniProt P027
4354.1.1	-3.5	4.74	1901.0717	0.0039	3	50.69	ggmr	63	KFICPLTGLWPINTLK	78	ctpr	C [66] 57.0215	ENSP00000205948	Beta-2-glycoprotein 1 Precursor (Beta-2-glycoprotein I)(Beta(2)GPI)(B2GPI)(Apolipoprotein H)(Apo-H)(Activated protein C-binding protein)(APC inhibitor)(Anticardiolipin cofactor) [Source: UniProt P027
4371.1.1	-1.8	4.1	1804.9666	0.0068	2	49.54	gmrk	64	FICPLTGLWPINTLK	78	ctpr	C [66] 57.0215, W [72] 31.9898	ENSP00000205948	Beta-2-glycoprotein 1 Precursor (Beta-2-glycoprotein I)(Beta(2)GPI)(B2GPI)(Apolipoprotein H)(Apo-H)(Activated protein C-binding protein)(APC inhibitor)(Anticardiolipin cofactor) [Source: UniProt P027
4373.1.1	-6.7	5.63	3522.7106	0.0009	3	51.28	dsak	124	CTEEGKWSPELPVCAPIICPPPSIPT	154	vykp	C [124] 57.0215, C [137] 57.0215,	ENSP00000205948	Beta-2-glycoprotein 1 Precursor (Beta-2-glycoprotein I)(Beta(2)GPI)(B2GPI)(Apolipoprotein H)(Apo-H)(Activated protein C-binding protein)(APC inhibitor)(Anticardiolipin cofactor) [Source: UniProt P027
4378.1.1	-4.7	4.79	1788.9717	-0.0003	2	49.54	gmrk	64	FICPLTGLWPINTLK	78	ctpr	C [66] 57.0215, W [72] 15.9949	ENSP00000205948	Beta-2-glycoprotein 1 Precursor (Beta-2-glycoprotein I)(Beta(2)GPI)(B2GPI)(Apolipoprotein H)(Apo-H)(Activated protein C-binding protein)(APC inhibitor)(Anticardiolipin cofactor) [Source: UniProt P027
4379.1.1	-4.8	4.43	1804.9666	0.0023	2	49.54	gmrk	64	FICPLTGLWPINTLK	78	ctpr	C [66] 57.0215, W [72] 31.9898	ENSP00000205948	Beta-2-glycoprotein 1 Precursor (Beta-2-glycoprotein I)(Beta(2)GPI)(B2GPI)(Apolipoprotein H)(Apo-H)(Activated protein C-binding protein)(APC inhibitor)(Anticardiolipin cofactor) [Source: UniProt P027
4381.1.1	-3.1	4.39	1902.057	0.011	3	50.69	ggmr	63	KFICPLTGLWPINTLK	78	ctpr	C [66] 57.0215, N [75] 0.9848	ENSP00000205948	Beta-2-glycoprotein 1 Precursor (Beta-2-glycoprotein I)(Beta(2)GPI)(B2GPI)(Apolipoprotein H)(Apo-H)(Activated protein C-binding protein)(APC inhibitor)(Anticardiolipin cofactor) [Source: UniProt P027
4382.1.1	-1.3	4.13	2624.364	-0.014	2	40.36	gthr	45	EKGPPTMLPINVDPDSKPGEYVLK	68	slfv		ENSP00000369607	furry homolog Source: RefSeq_peptide NP_075463
4384.1.1	-9.3	5.57	3522.7106	0.002	3	51.28	dsak	124	CTEEGKWSPELPVCAPIICPPPSIPT	154	vykp	C [124] 57.0215, C [137] 57.0215,	ENSP00000205948	Beta-2-glycoprotein 1 Precursor (Beta-2-glycoprotein I)(Beta(2)GPI)(B2GPI)(Apolipoprotein H)(Apo-H)(Activated protein C-binding protein)(APC inhibitor)(Anticardiolipin cofactor) [Source: UniProt P027
4385.1.1	-4.5	4.8	1788.9717	0.0022	2	49.54	gmrk	64	FICPLTGLWPINTLK	78	ctpr	C [66] 57.0215, W [72] 15.9949	ENSP00000205948	Beta-2-glycoprotein 1 Precursor (Beta-2-glycoprotein I)(Beta(2)GPI)(B2GPI)(Apolipoprotein H)(Apo-H)(Activated protein C-binding protein)(APC inhibitor)(Anticardiolipin cofactor) [Source: UniProt P027
4386.1.1	-6.8	4.95	1804.9666	0.0039	2	49.54	gmrk	64	FICPLTGLWPINTLK	78	ctpr	C [66] 57.0215, W [72] 31.9898	ENSP00000205948	Beta-2-glycoprotein 1 Precursor (Beta-2-glycoprotein I)(Beta(2)GPI)(B2GPI)(Apolipoprotein H)(Apo-H)(Activated protein C-binding protein)(APC inhibitor)(Anticardiolipin cofactor) [Source: UniProt P027
4388.1.1	-7.1	4.51	2851.4521	0.0056	3	56.03	eegk	130	WSPELPVCAPIICPPPSIPTFATLR	154	vykp	C [137] 57.0215, C [142] 57.0215,	ENSP00000205948	Beta-2-glycoprotein 1 Precursor (Beta-2-glycoprotein I)(Beta(2)GPI)(B2GPI)(Apolipoprotein H)(Apo-H)(Activated protein C-binding protein)(APC inhibitor)(Anticardiolipin cofactor) [Source: UniProt P027
4390.1.1	-8.5	5.67	3522.7106	0.0013	3	51.28	dsak	124	CTEEGKWSPELPVCAPIICPPPSIPT	154	vykp	C [124] 57.0215, C [137] 57.0215,	ENSP00000205948	Beta-2-glycoprotein 1 Precursor (Beta-2-glycoprotein I)(Beta(2)GPI)(B2GPI)(Apolipoprotein H)(Apo-H)(Activated protein C-binding protein)(APC inhibitor)(Anticardiolipin cofactor) [Source: UniProt P027
4391.1.1	-4.3	4.78	1788.9717	0	2	49.54	gmrk	64	FICPLTGLWPINTLK	78	ctpr	C [66] 57.0215, W [72] 15.9949	ENSP00000205948	Beta-2-glycoprotein 1 Precursor (Beta-2-glycoprotein I)(Beta(2)GPI)(B2GPI)(Apolipoprotein H)(Apo-H)(Activated protein C-binding protein)(APC inhibitor)(Anticardiolipin cofactor) [Source: UniProt P027
4392.1.1	-6.6	4.57	2851.4521	0.0068	3	56.03	eegk	130	WSPELPVCAPIICPPPSIPTFATLR	154	vykp	C [137] 57.0215, C [142] 57.0215,	ENSP00000205948	Beta-2-glycoprotein 1 Precursor (Beta-2-glycoprotein I)(Beta(2)GPI)(B2GPI)(Apolipoprotein H)(Apo-H)(Activated protein C-binding protein)(APC inhibitor)(Anticardiolipin cofactor) [Source: UniProt P027
4393.1.1	-1.4	4.58	1573.782	-0.013	2	23.68	etar	235	AAEEALSSLGQSGTPR	250	lwar		ENSP00000377334:reversed	
4399.1.1	-5	4.56	2851.452	0.014	3	56.03	eegk	130	WSPELPVCAPIICPPPSIPTFATLR	154	vykp	C [137] 57.0215, C [142] 57.0215,	ENSP00000205948	Beta-2-glycoprotein 1 Precursor (Beta-2-glycoprotein I)(Beta(2)GPI)(B2GPI)(Apolipoprotein H)(Apo-H)(Activated protein C-binding protein)(APC inhibitor)(Anticardiolipin cofactor) [Source: UniProt P027
4404.1.1	-2	4.76	1573.782	-0.013	2	23.68	etar	235	AAEEALSSLGQSGTPR	250	lwar		ENSP00000377334:reversed	
4414.1.1	-5.1	4.42	1788.9717	0.0017	3	49.54	gmrk	64	FICPLTGLWPINTLK	78	ctpr	C [66] 57.0215, W [72] 15.9949	ENSP00000205948	Beta-2-glycoprotein 1 Precursor (Beta-2-glycoprotein I)(Beta(2)GPI)(B2GPI)(Apolipoprotein H)(Apo-H)(Activated protein C-binding protein)(APC inhibitor)(Anticardiolipin cofactor) [Source: UniProt P027
4415.1.1	-4.5	3.83	1804.9666	-0.003	3	49.54	gmrk	64	FICPLTGLWPINTLK	78	ctpr	C [66] 57.0215, W [72] 31.9898	ENSP00000205948	Beta-2-glycoprotein 1 Precursor (Beta-2-glycoprotein I)(Beta(2)GPI)(B2GPI)(Apolipoprotein H)(Apo-H)(Activated protein C-binding protein)(APC inhibitor)(Anticardiolipin cofactor) [Source: UniProt P027
4420.1.1	-3.1	4.4	1788.9717	0.0022	3	49.54	gmrk	64	FICPLTGLWPINTLK	78	ctpr	C [66] 57.0215, W [72] 15.9949	ENSP00000205948	Beta-2-glycoprotein 1 Precursor (Beta-2-glycoprotein I)(Beta(2)GPI)(B2GPI)(Apolipoprotein H)(Apo-H)(Activated protein C-binding protein)(APC inhibitor)(Anticardiolipin cofactor) [Source: UniProt P027
4425.1.1	-3.5	4.34	1788.9717	-0.0068	3	49.54	gmrk	64	FICPLTGLWPINTLK	78	ctpr	C [66] 57.0215, W [72] 15.9949	ENSP00000205948	Beta-2-glycoprotein 1 Precursor (Beta-2-glycoprotein I)(Beta(2)GPI)(B2GPI)(Apolipoprotein H)(Apo-H)(Activated protein C-binding protein)(APC inhibitor)(Anticardiolipin cofactor) [Source: UniProt P027
4455.1.1	-2.7	4.03	1502.7784	-0.0025	2	35.15	ctpr	83	VCPFAGILENGAVR	96	yttf	C [84] 57.0215	ENSP00000205948	Beta-2-glycoprotein 1 Precursor (Beta-2-glycoprotein I)(Beta(2)GPI)(B2GPI)(Apolipoprotein H)(Apo-H)(Activated protein C-binding protein)(APC inhibitor)(Anticardiolipin cofactor) [Source: UniProt P027
4457.1.1	-4.6	4.39	1772.9768	-0.0063	2	49.54	gmrk	64	FICPLTGLWPINTLK	78	ctpr	C [66] 57.0215	ENSP00000205948	Beta-2-glycoprotein 1 Precursor (Beta-2-glycoprotein I)(Beta(2)GPI)(B2GPI)(Apolipoprotein H)(Apo-H)(Activated protein C-binding protein)(APC inhibitor)(Anticardiolipin cofactor) [Source: UniProt P027
4461.1.1	-7.1	4.42	1772.9768	-0.0026	2	49.54	gmrk	64	FICPLTGLWPINTLK	78	ctpr	C [66] 57.0215	ENSP00000205948	Beta-2-glycoprotein 1 Precursor (Beta-2-glycoprotein I)(Beta(2)GPI)(B2GPI)(Apolipoprotein H)(Apo-H)(Activated protein C-binding protein)(APC inhibitor)(Anticardiolipin cofactor) [Source: UniProt P027
4462.1.1	-5.7	4.49	2819.4623	0.007	2	56.03	eegk	130	WSPELPVCAPIICPPPSIPTFATLR	154	vykp	C [137] 57.0215, C [142] 57.0215	ENSP00000205948	Beta-2-glycoprotein 1 Precursor (Beta-2-glycoprotein I)(Beta(2)GPI)(B2GPI)(Apolipoprotein H)(Apo-H)(Activated protein C-binding protein)(APC inhibitor)(Anticardiolipin cofactor) [Source: UniProt P027
4465.1.1	-4.7	4.45	1772.9768	-0.0005	2	49.54	gmrk	64	FICPLTGLWPINTLK	78	ctpr	C [66] 57.0215	ENSP00000205948	Beta-2-glycoprotein 1 Precursor (Beta-2-glycoprotein I)(Beta(2)GPI)(B2GPI)(Apolipoprotein H)(Apo-H)(Activated protein C-binding protein)(APC inhibitor)(Anticardiolipin cofactor) [Source: UniProt P027
4468.1.1	-4.6	5.06	2819.4623	0.0016	2	56.03	eegk	130	WSPELPVCAPIICPPPSIPTFATLR	154	vykp	C [137] 57.0215, C [142] 57.0215	ENSP00000205948	Beta-2-glycoprotein 1 Precursor (Beta-2-glycoprotein I)(Beta(2)GPI)(B2GPI)(Apolipoprotein H)(Apo-H)(Activated protein C-binding protein)(APC inhibitor)(Anticardiolipin cofactor) [Source: UniProt P027

4471.1.1	-6.1	5.13	3523.7422	0.0013	3	51.28	dsak	124	CTEEGKWSPELPVCAPIICPPPSIPT	154	vykp	C [124] 57.0215, C [137] 57.0215,	ENSP00000205948	Beta-2-glycoprotein 1 Precursor (Beta-2-glycoprotein I)(Beta(2)GPI)(B2GPI)(Apolipoprotein H)(Apo-H)(Activated protein C-binding protein)(APC inhibitor)(Anticardiolipin cofactor) [Source: UniProt P027
4472.1.1	-5.6	5.18	2819.4623	0.0019	2	56.03	eegk	130	WSPELPVCAPIICPPPSIPTFATLR	154	vykp	C [137] 57.0215, C [142] 57.0215	ENSP00000205948	Beta-2-glycoprotein 1 Precursor (Beta-2-glycoprotein I)(Beta(2)GPI)(B2GPI)(Apolipoprotein H)(Apo-H)(Activated protein C-binding protein)(APC inhibitor)(Anticardiolipin cofactor) [Source: UniProt P027
4474.1.1	-5	4.46	1788.9717	-0.0001	2	49.54	gmrk	64	FICPLTGLWPINTLK	78	ctpr	C [66] 57.0215, W [72] 15.9949	ENSP00000205948	Beta-2-glycoprotein 1 Precursor (Beta-2-glycoprotein I)(Beta(2)GPI)(B2GPI)(Apolipoprotein H)(Apo-H)(Activated protein C-binding protein)(APC inhibitor)(Anticardiolipin cofactor) [Source: UniProt P027
4477.1.1	-4.7	5.3	3523.7422	-0.0009	3	51.28	dsak	124	CTEEGKWSPELPVCAPIICPPPSIPT	154	vykp	C [124] 57.0215, C [137] 57.0215,	ENSP00000205948	Beta-2-glycoprotein 1 Precursor (Beta-2-glycoprotein I)(Beta(2)GPI)(B2GPI)(Apolipoprotein H)(Apo-H)(Activated protein C-binding protein)(APC inhibitor)(Anticardiolipin cofactor) [Source: UniProt P027
4479.1.1	-6.7	4.59	1788.9717	-0.0044	2	49.54	gmrk	64	FICPLTGLWPINTLK	78	ctpr	C [66] 57.0215, W [72] 15.9949	ENSP00000205948	Beta-2-glycoprotein 1 Precursor (Beta-2-glycoprotein I)(Beta(2)GPI)(B2GPI)(Apolipoprotein H)(Apo-H)(Activated protein C-binding protein)(APC inhibitor)(Anticardiolipin cofactor) [Source: UniProt P027
4482.1.1	-6.6	5.37	3523.7422	-0.0002	3	51.28	dsak	124	CTEEGKWSPELPVCAPIICPPPSIPT	154	vykp	C [124] 57.0215, C [137] 57.0215,	ENSP00000205948	Beta-2-glycoprotein 1 Precursor (Beta-2-glycoprotein I)(Beta(2)GPI)(B2GPI)(Apolipoprotein H)(Apo-H)(Activated protein C-binding protein)(APC inhibitor)(Anticardiolipin cofactor) [Source: UniProt P027
4485.1.1	-6	4.66	1788.9717	0.0027	2	49.54	gmrk	64	FICPLTGLWPINTLK	78	ctpr	C [66] 57.0215, W [72] 15.9949	ENSP00000205948	Beta-2-glycoprotein 1 Precursor (Beta-2-glycoprotein I)(Beta(2)GPI)(B2GPI)(Apolipoprotein H)(Apo-H)(Activated protein C-binding protein)(APC inhibitor)(Anticardiolipin cofactor) [Source: UniProt P027
4499.1.1	-7.4	5.59	2819.4623	0.0085	3	56.03	eegk	130	WSPELPVCAPIICPPPSIPTFATLR	154	vykp	C [137] 57.0215, C [142] 57.0215	ENSP00000205948	Beta-2-glycoprotein 1 Precursor (Beta-2-glycoprotein I)(Beta(2)GPI)(B2GPI)(Apolipoprotein H)(Apo-H)(Activated protein C-binding protein)(APC inhibitor)(Anticardiolipin cofactor) [Source: UniProt P027
4506.1.1	-10.3	5.33	2819.4623	0.0026	3	56.03	eegk	130	WSPELPVCAPIICPPPSIPTFATLR	154	vykp	C [137] 57.0215, C [142] 57.0215	ENSP00000205948	Beta-2-glycoprotein 1 Precursor (Beta-2-glycoprotein I)(Beta(2)GPI)(B2GPI)(Apolipoprotein H)(Apo-H)(Activated protein C-binding protein)(APC inhibitor)(Anticardiolipin cofactor) [Source: UniProt P027
4513.1.1	-7.2	5.21	2819.4623	0.0026	3	56.03	eegk	130	WSPELPVCAPIICPPPSIPTFATLR	154	vykp	C [137] 57.0215, C [142] 57.0215	ENSP00000205948	Beta-2-glycoprotein 1 Precursor (Beta-2-glycoprotein I)(Beta(2)GPI)(B2GPI)(Apolipoprotein H)(Apo-H)(Activated protein C-binding protein)(APC inhibitor)(Anticardiolipin cofactor) [Source: UniProt P027
4519.1.1	-8.2	5.39	3506.7157	-0.0057	3	51.28	dsak	124	CTEEGKWSPELPVCAPIICPPPSIPT	154	vykp	C [124] 57.0215, C [137] 57.0215,	ENSP00000205948	Beta-2-glycoprotein 1 Precursor (Beta-2-glycoprotein I)(Beta(2)GPI)(B2GPI)(Apolipoprotein H)(Apo-H)(Activated protein C-binding protein)(APC inhibitor)(Anticardiolipin cofactor) [Source: UniProt P027
4523.1.1	-3.6	4.13	1788.9717	-0.0044	3	49.54	gmrk	64	FICPLTGLWPINTLK	78	ctpr	C [66] 57.0215, W [72] 15.9949	ENSP00000205948	Beta-2-glycoprotein 1 Precursor (Beta-2-glycoprotein I)(Beta(2)GPI)(B2GPI)(Apolipoprotein H)(Apo-H)(Activated protein C-binding protein)(APC inhibitor)(Anticardiolipin cofactor) [Source: UniProt P027
4525.1.1	-7.5	5.24	3506.7157	-0.0014	3	51.28	dsak	124	CTEEGKWSPELPVCAPIICPPPSIPT	154	vykp	C [124] 57.0215, C [137] 57.0215,	ENSP00000205948	Beta-2-glycoprotein 1 Precursor (Beta-2-glycoprotein I)(Beta(2)GPI)(B2GPI)(Apolipoprotein H)(Apo-H)(Activated protein C-binding protein)(APC inhibitor)(Anticardiolipin cofactor) [Source: UniProt P027
4526.1.1	-5.2	4.92	1772.9768	0.0025	3	49.54	gmrk	64	FICPLTGLWPINTLK	78	ctpr	C [66] 57.0215	ENSP00000205948	Beta-2-glycoprotein 1 Precursor (Beta-2-glycoprotein I)(Beta(2)GPI)(B2GPI)(Apolipoprotein H)(Apo-H)(Activated protein C-binding protein)(APC inhibitor)(Anticardiolipin cofactor) [Source: UniProt P027
4532.1.1	-10.8	5.21	3506.7157	-0.0014	3	51.28	dsak	124	CTEEGKWSPELPVCAPIICPPPSIPT	154	vykp	C [124] 57.0215, C [137] 57.0215,	ENSP00000205948	Beta-2-glycoprotein 1 Precursor (Beta-2-glycoprotein I)(Beta(2)GPI)(B2GPI)(Apolipoprotein H)(Apo-H)(Activated protein C-binding protein)(APC inhibitor)(Anticardiolipin cofactor) [Source: UniProt P027
4533.1.1	-5.9	5.25	1772.9768	-0.0004	3	49.54	gmrk	64	FICPLTGLWPINTLK	78	ctpr	C [66] 57.0215	ENSP00000205948	Beta-2-glycoprotein 1 Precursor (Beta-2-glycoprotein I)(Beta(2)GPI)(B2GPI)(Apolipoprotein H)(Apo-H)(Activated protein C-binding protein)(APC inhibitor)(Anticardiolipin cofactor) [Source: UniProt P027
4539.1.1	-4.6	5.33	1772.9768	0.0018	3	49.54	gmrk	64	FICPLTGLWPINTLK	78	ctpr	C [66] 57.0215	ENSP00000205948	Beta-2-glycoprotein 1 Precursor (Beta-2-glycoprotein I)(Beta(2)GPI)(B2GPI)(Apolipoprotein H)(Apo-H)(Activated protein C-binding protein)(APC inhibitor)(Anticardiolipin cofactor) [Source: UniProt P027
4551.1.1	-7.5	6.59	1772.9768	0.0004	2	49.54	gmrk	64	FICPLTGLWPINTLK	78	ctpr	C [66] 57.0215	ENSP00000205948	Beta-2-glycoprotein 1 Precursor (Beta-2-glycoprotein I)(Beta(2)GPI)(B2GPI)(Apolipoprotein H)(Apo-H)(Activated protein C-binding protein)(APC inhibitor)(Anticardiolipin cofactor) [Source: UniProt P027
4554.1.1	-1.4	4.14	1557.7591	0.0092	2	27.55	aqgr	200	WSALSEGPCAPRAR	213	cssc	C [208] 57.0215	ENSP00000350111	49 kDa protein [Source: IPI (IPI00477516)]
4555.1.1	-1.1	3.75	1313.6862	-0.0092	2	29.92	reqr	111	AGWSRAGPAWVR	122	riwr		ENSP00000381377	
4557.1.1	-10.7	6.69	1772.9768	0.0004	2	49.54	gmrk	64	FICPLTGLWPINTLK	78	ctpr	C [66] 57.0215	ENSP00000205948	Beta-2-glycoprotein 1 Precursor (Beta-2-glycoprotein I)(Beta(2)GPI)(B2GPI)(Apolipoprotein H)(Apo-H)(Activated protein C-binding protein)(APC inhibitor)(Anticardiolipin cofactor) [Source: UniProt P027
4561.1.1	-9.4	6.82	1772.9768	0.0025	2	49.54	gmrk	64	FICPLTGLWPINTLK	78	ctpr	C [66] 57.0215	ENSP00000205948	Beta-2-glycoprotein 1 Precursor (Beta-2-glycoprotein I)(Beta(2)GPI)(B2GPI)(Apolipoprotein H)(Apo-H)(Activated protein C-binding protein)(APC inhibitor)(Anticardiolipin cofactor) [Source: UniProt P027
4567.1.1	-1.6	5.08	1788.9717	0.0024	2	49.54	gmrk	64	FICPLTGLWPINTLK	78	ctpr	C [66] 57.0215, W [72] 15.9949	ENSP00000205948	Beta-2-glycoprotein 1 Precursor (Beta-2-glycoprotein I)(Beta(2)GPI)(B2GPI)(Apolipoprotein H)(Apo-H)(Activated protein C-binding protein)(APC inhibitor)(Anticardiolipin cofactor) [Source: UniProt P027
4572.1.1	-1.6	5.08	1788.9717	0.0016	2	49.54	gmrk	64	FICPLTGLWPINTLK	78	ctpr	C [66] 57.0215, W [72] 15.9949	ENSP00000205948	Beta-2-glycoprotein 1 Precursor (Beta-2-glycoprotein I)(Beta(2)GPI)(B2GPI)(Apolipoprotein H)(Apo-H)(Activated protein C-binding protein)(APC inhibitor)(Anticardiolipin cofactor) [Source: UniProt P027
4575.1.1	-2.8	4.95	1788.9717	-0.0018	2	49.54	gmrk	64	FICPLTGLWPINTLK	78	ctpr	C [66] 57.0215, W [72] 15.9949	ENSP00000205948	Beta-2-glycoprotein 1 Precursor (Beta-2-glycoprotein I)(Beta(2)GPI)(B2GPI)(Apolipoprotein H)(Apo-H)(Activated protein C-binding protein)(APC inhibitor)(Anticardiolipin cofactor) [Source: UniProt P027
4613.1.1	-6	5.74	1772.9768	0.0018	3	49.54	gmrk	64	FICPLTGLWPINTLK	78	ctpr	C [66] 57.0215	ENSP00000205948	Beta-2-glycoprotein 1 Precursor (Beta-2-glycoprotein I)(Beta(2)GPI)(B2GPI)(Apolipoprotein H)(Apo-H)(Activated protein C-binding protein)(APC inhibitor)(Anticardiolipin cofactor) [Source: UniProt P027
4617.1.1	-7.5	5.54	1772.9768	0.0036	3	49.54	gmrk	64	FICPLTGLWPINTLK	78	ctpr	C [66] 57.0215	ENSP00000205948	Beta-2-glycoprotein 1 Precursor (Beta-2-glycoprotein I)(Beta(2)GPI)(B2GPI)(Apolipoprotein H)(Apo-H)(Activated protein C-binding protein)(APC inhibitor)(Anticardiolipin cofactor) [Source: UniProt P027
4622.1.1	-6.5	5.45	1772.9768	0.0001	3	49.54	gmrk	64	FICPLTGLWPINTLK	78	ctpr	C [66] 57.0215	ENSP00000205948	Beta-2-glycoprotein 1 Precursor (Beta-2-glycoprotein I)(Beta(2)GPI)(B2GPI)(Apolipoprotein H)(Apo-H)(Activated protein C-binding protein)(APC inhibitor)(Anticardiolipin cofactor) [Source: UniProt P027
4634.1.1	-8.5	6.44	1772.9768	0	2	49.54	gmrk	64	FICPLTGLWPINTLK	78	ctpr	C [66] 57.0215	ENSP00000205948	Beta-2-glycoprotein 1 Precursor (Beta-2-glycoprotein I)(Beta(2)GPI)(B2GPI)(Apolipoprotein H)(Apo-H)(Activated protein C-binding protein)(APC inhibitor)(Anticardiolipin cofactor) [Source: UniProt P027
4641.1.1	-8.7	6.55	1772.9768	-0.0022	2	49.54	gmrk	64	FICPLTGLWPINTLK	78	ctpr	C [66] 57.0215	ENSP00000205948	Beta-2-glycoprotein 1 Precursor (Beta-2-glycoprotein I)(Beta(2)GPI)(B2GPI)(Apolipoprotein H)(Apo-H)(Activated protein C-binding protein)(APC inhibitor)(Anticardiolipin cofactor) [Source: UniProt P027
4646.1.1	-7.1	6.34	1772.9768	0.0003	2	49.54	gmrk	64	FICPLTGLWPINTLK	78	ctpr	C [66] 57.0215	ENSP00000205948	Beta-2-glycoprotein 1 Precursor (Beta-2-glycoprotein I)(Beta(2)GPI)(B2GPI)(Apolipoprotein H)(Apo-H)(Activated protein C-binding protein)(APC inhibitor)(Anticardiolipin cofactor) [Source: UniProt P027
4692.1.1	-1.7	3.92	1788.972	0.987	2	49.54	gmrk	64	FICPLTGLWPINTLK	78	ctpr	C [66] 57.0215, W [72] 15.9949	ENSP00000205948	Beta-2-glycoprotein 1 Precursor (Beta-2-glycoprotein I)(Beta(2)GPI)(B2GPI)(Apolipoprotein H)(Apo-H)(Activated protein C-binding protein)(APC inhibitor)(Anticardiolipin cofactor) [Source: UniProt P027
4726.1.1	-5.5	3.89	3506.716	-0.011	3	51.28	dsak	124	CTEEGKWSPELPVCAPIICPPPSIPT	154	vykp	C [124] 57.0215, C [137] 57.0215,	ENSP00000205948	Beta-2-glycoprotein 1 Precursor (Beta-2-glycoprotein I)(Beta(2)GPI)(B2GPI)(Apolipoprotein H)(Apo-H)(Activated protein C-binding protein)(APC inhibitor)(Anticardiolipin cofactor) [Source: UniProt P027
4739.1.1	-1.1	4.01	1574.7657	-0.004	2	24.59	pfsk	1108	ETSSTSAISPSEAGLR	1123	crel	E [1108] -18.0106	ENSP00000374409:reversed	
4750.1.1	-1.3	4.08	1574.781	-0.014	2	30.2	mldr	556	EIEEYLGAIREDR	568	eeta	E [556] -18.0106	ENSP00000284509:reversed	
4763.1.1	-5.4	4.97	1772.9768	0.0007	2	49.54	gmrk	64	FICPLTGLWPINTLK	78	ctpr	C [66] 57.0215	ENSP00000205948	Beta-2-glycoprotein 1 Precursor (Beta-2-glycoprotein I)(Beta(2)GPI)(B2GPI)(Apolipoprotein H)(Apo-H)(Activated protein C-binding protein)(APC inhibitor)(Anticardiolipin cofactor) [Source: UniProt P027
4777.1.1	-6.2	4.88	1772.9768	0.0013	2	49.54	gmrk	64	FICPLTGLWPINTLK	78	ctpr	C [66] 57.0215	ENSP00000205948	Beta-2-glycoprotein 1 Precursor (Beta-2-glycoprotein I)(Beta(2)GPI)(B2GPI)(Apolipoprotein H)(Apo-H)(Activated protein C-binding protein)(APC inhibitor)(Anticardiolipin cofactor) [Source: UniProt P027
4779.1.1	-2	4.21	1788.972	0.991	2	49.54	gmrk	64	FICPLTGLWPINTLK	78	ctpr	C [66] 57.0215, W [72] 15.9949	ENSP00000205948	Beta-2-glycoprotein 1 Precursor (Beta-2-glycoprotein I)(Beta(2)GPI)(B2GPI)(Apolipoprotein H)(Apo-H)(Activated protein C-binding protein)(APC inhibitor)(Anticardiolipin cofactor) [Source: UniProt P027
4784.1.1	-5.8	4.86	1772.9768	-0.0003	2	49.54	gmrk	64	FICPLTGLWPINTLK	78	ctpr	C [66] 57.0215	ENSP00000205948	Beta-2-glycoprotein 1 Precursor (Beta-2-glycoprotein I)(Beta(2)GPI)(B2GPI)(Apolipoprotein H)(Apo-H)(Activated protein C-binding protein)(APC inhibitor)(Anticardiolipin cofactor) [Source: UniProt P027
4818.1.1	-1.6	3.76	2044.9	0.986	2	30.23	ndek	1518	AEWQIGEKECHPCSWK	1533	gepa	C [1527] 57.0215, C [1530] 57.021	ENSP00000312419:reversed	
4820.1.1	-4.6	3.84	3523.742	0.019	3	51.28	dsak	124	CTEEGKWSPELPVCAPIICPPPSIPT	154	vykp	C [124] 57.0215, C [137] 57.0215,	ENSP00000205948	Beta-2-glycoprotein 1 Precursor (Beta-2-glycoprotein I)(Beta(2)GPI)(B2GPI)(Apolipoprotein H)(Apo-H)(Activated protein C-binding protein)(APC inhibitor)(Anticardiolipin cofactor) [Source: UniProt P027
4821.1.1	-1.4	3.84	1543.7646	0.0016	2	16.4	[	1	MAAAPDVGNGGSKNVR	16	kprp		ENSP00000334077	No description
4853.1.1	-1.2	4.08	1742.933	2.017	2	28.05	einr	387	MIQRLRSEIDHVKK	400	qcas	M [387] 15.9949, I [395] -26.052	ENSP00000252250	Keratin, type II cytoskeletal 6E (Cytokeratin-6E) (CK 6E) (K6e keratin) (Keratin K6h). Source: Uniprot/SWISSPROT P48668
4855.1.1	-5.4	4.7	1772.9768	0.0001	2	49.54	gmrk	64	FICPLTGLWPINTLK	78	ctpr	C [66] 57.0215	ENSP00000205948	Beta-2-glycoprotein 1 Precursor (Beta-2-glycoprotein I)(Beta(2)GPI)(B2GPI)(Apolipoprotein H)(Apo-H)(Activated protein C-binding protein)(APC inhibitor)(Anticardiolipin cofactor) [Source: UniProt P027
4857.1.1	-5.5	4.67	1772.9768	0.0007	2	49.54	gmrk	64	FICPLTGLWPINTLK	78	ctpr	C [66] 57.0215	ENSP00000205948	Beta-2-glycoprotein 1 Precursor (Beta-2-glycoprotein I)(Beta(2)GPI)(B2GPI)(Apolipoprotein H)(Apo-H)(Activated protein C-binding protein)(APC inhibitor)(Anticardiolipin cofactor) [Source: UniProt P027
4859.1.1	-3.9	3.98	3523.742	0.015	3	51.28	dsak	124	CTEEGKWSPELPVCAPIICPPPSIPT	154	vykp	C [124] 57.0215, C [137] 57.0215,	ENSP00000205948	Beta-2-glycoprotein 1 Precursor (Beta-2-glycoprotein I)(Beta(2)GPI)(B2GPI)(Apolipoprotein H)(Apo-H)(Activated protein C-binding protein)(APC inhibitor)(Anticardiolipin cofactor) [Source: UniProt P027
4862.1.1	-6.4	4.67	1772.9768	0.0007	2	49.54	gmrk	64	FICPLTGLWPINTLK	78	ctpr	C [66] 57.0215	ENSP00000205948	Beta-2-glycoprotein 1 Precursor (Beta-2-glycoprotein I)(Beta(2)GPI)(B2GPI)(Apolipoprotein H)(Apo-H)(Activated protein C-binding protein)(APC inhibitor)(Anticardiolipin cofactor) [Source: UniProt P027
4865.1.1	-1.9	3.77	1502.7784	-0.0036	2	35.15	ctpr	83	VCPFAGILENGAVR	96	yttf	C [84] 57.0215	ENSP00000205948	Beta-2-glycoprotein 1 Precursor (Beta-2-glycoprotein I)(Beta(2)GPI)(B2GPI)(Apolipoprotein H)(Apo-H)(Activated protein C-binding protein)(APC inhibitor)(Anticardiolipin cofactor) [Source: UniProt P027
4885.1.1	-2.4	3.77	2714.307	-0.013	3	41.86	revk	205	CPFPSRPDNGFVNYPAKPTLYYK	227	dkat	C [205] 57.0215, C [205] -17.0265	ENSP00000205948	Beta-2-glycoprotein 1 Precursor (Beta-2-glycoprotein I)(Beta(2)GPI)(B2GPI)(Apolipoprotein H)(Apo-H)(Activated protein C-binding protein)(APC inhibitor)(Anticardiolipin cofactor) [Source: UniProt P027

Sample Beta-2-GPI/TRX-1/TRX-R/NADPH+MPB

False discovery rat 0.67%

Proteins

rank	log(e)	log(I)	% (measured	% (corrected	unique	total	Mr	Accession	Description
1	-625.8	7.67	73	100	57	357	38.3	ENSP0000020594	APOH, Beta-2-glycoprotein 1 Precursor (Beta-2-glycoprotein I)(Beta(2)GPI)(B2GPI)(Apolipoprotein H)(Apo-H)(Activated protein C-binding protein)(APC inhibitor)(Anticardiolipin cofactor) [Source: UniProt P02749]
2	-146.1	6.59	30	36	15	56	54.5	ENSP0000037350	TXNRD1, Thioredoxin reductase 1, cytoplasmic (TR)(EC 1.8.1.9)(Thioredoxin reductase TR1)(KM-102-derived reductase-like factor)(Gene associated with retinoid-IFN-induced mortality 12 protein)(GRIM-12) [Source: UniProt Q16881] Source: 1.8.1.9
3	-77.2	5.52	13	22	8	16	66	ENSP0000025224	KRT1, Keratin, type II cytoskeletal 1 (Cytokeratin-1) (CK-1) (Keratin-1) (K1) (67 kDa cytokeratin) (Hair alpha protein). Source: Uniprot/SWISSPROT P04264
4	-57.4	5.11	11	17	6	11	62	ENSP0000024666	KRT9, Keratin, type I cytoskeletal 9 (Cytokeratin-9) (CK-9) (Keratin-9) (K9). Source: Uniprot/SWISSPROT P35527
5	-37	5.19	9.2	16	4	8	58.8	ENSP0000026957	KRT10, Keratin, type I cytoskeletal 10 (Cytokeratin-10) (CK-10) (Keratin-10) (K10). Source: Uniprot/SWISSPROT P13645
6	-29.5	5.05	4.4	7	3	5	60	ENSP0000025225	KRT6B, Keratin, type II cytoskeletal 6B (Cytokeratin-6B) (CK 6B) (K6b keratin). Source: Uniprot/SWISSPROT P04259
7	-27.3	5.81	23	30	4	11	24.4	sp|TRYP_PIG|	Trypsin; EC 3.4.21.4; Flags: Precursor;
9	-19.3	5.59	26	30	3	7	11.7	ENSP0000036364	TXN, Thioredoxin (Trx)(ATL-derived factor)(ADF)(Surface-associated sulphydryl protein)(SASP) [Source: UniProt P10599]

Peptides

Spectrum	log(e)	log(I)	m+h	delta	z	retention	pre	start	sequence	end	post	modifications	Protein	Description
721.1.1	-1.9	3.59	1016.5921	0.0029	2	7	psck	262	ASCKVPVKK	270	atvv	C [264] 57.0215	ENSP00000205948	Beta-2-glycoprotein 1 Precursor (Beta-2-glycoprotein I)(Beta(2)GPI)(B2GPI)(Apolipoprotein H)(Apo-H)(Activated protein C-binding protein)(APC inhibitor)(Anticardiolipin cofactor) [Source: Ur
833.1.1	-2.2	3.71	1162.5674	0.0017	2	16.74	iqek	286	FKNGMLHGDK	295	vsff	M [290] 15.9949	ENSP00000205948	Beta-2-glycoprotein 1 Precursor (Beta-2-glycoprotein I)(Beta(2)GPI)(B2GPI)(Apolipoprotein H)(Apo-H)(Activated protein C-binding protein)(APC inhibitor)(Anticardiolipin cofactor) [Source: Ur
867.1.1	-4.1	4.01	1030.6078	0.0015	2	7	psck	262	ASCKVPVKK	270	atvv	C [264] 57.0215, V [266] 14.0157	ENSP00000205948	Beta-2-glycoprotein 1 Precursor (Beta-2-glycoprotein I)(Beta(2)GPI)(B2GPI)(Apolipoprotein H)(Apo-H)(Activated protein C-binding protein)(APC inhibitor)(Anticardiolipin cofactor) [Source: Ur
873.1.1	-3.2	3.99	1030.6078	0.0007	2	7	psck	262	ASCKVPVKK	270	atvv	C [264] 57.0215, V [266] 14.0157	ENSP00000205948	Beta-2-glycoprotein 1 Precursor (Beta-2-glycoprotein I)(Beta(2)GPI)(B2GPI)(Apolipoprotein H)(Apo-H)(Activated protein C-binding protein)(APC inhibitor)(Anticardiolipin cofactor) [Source: Ur
920.1.1	-2.1	4.53	1295.6049	0.0016	3	17.02	mank	236	IGEHMEEHGIK	246	firq	M [240] 15.9949	ENSP00000373506	Thioredoxin reductase 1, cytoplasmic (TR)(EC 1.8.1.9)(Thioredoxin reductase TR1)(KM-102-derived reductase-like factor)(Gene associated with retinoid-IFN-induced mortality 12 protein)(GR
922.1.1	-3.2	4.63	1295.6049	0.0005	3	17.02	mank	236	IGEHMEEHGIK	246	firq	M [240] 15.9949	ENSP00000373506	Thioredoxin reductase 1, cytoplasmic (TR)(EC 1.8.1.9)(Thioredoxin reductase TR1)(KM-102-derived reductase-like factor)(Gene associated with retinoid-IFN-induced mortality 12 protein)(GR
923.1.1	-1.9	3.77	1295.6049	-0.0023	2	17.02	mank	236	IGEHMEEHGIK	246	firq	M [240] 15.9949	ENSP00000373506	Thioredoxin reductase 1, cytoplasmic (TR)(EC 1.8.1.9)(Thioredoxin reductase TR1)(KM-102-derived reductase-like factor)(Gene associated with retinoid-IFN-induced mortality 12 protein)(GR
925.1.1	-2.7	4.66	1295.6049	0.0018	3	17.02	mank	236	IGEHMEEHGIK	246	firq	M [240] 15.9949	ENSP00000373506	Thioredoxin reductase 1, cytoplasmic (TR)(EC 1.8.1.9)(Thioredoxin reductase TR1)(KM-102-derived reductase-like factor)(Gene associated with retinoid-IFN-induced mortality 12 protein)(GR
926.1.1	-2.3	3.77	1295.6049	-0.0001	2	17.02	mank	236	IGEHMEEHGIK	246	firq	M [240] 15.9949	ENSP00000373506	Thioredoxin reductase 1, cytoplasmic (TR)(EC 1.8.1.9)(Thioredoxin reductase TR1)(KM-102-derived reductase-like factor)(Gene associated with retinoid-IFN-induced mortality 12 protein)(GR
928.1.1	-3.3	3.85	1295.6049	-0.0003	2	17.02	mank	236	IGEHMEEHGIK	246	firq	M [240] 15.9949	ENSP00000373506	Thioredoxin reductase 1, cytoplasmic (TR)(EC 1.8.1.9)(Thioredoxin reductase TR1)(KM-102-derived reductase-like factor)(Gene associated with retinoid-IFN-induced mortality 12 protein)(GR
945.1.1	-2.1	3.94	992.4353	0.001	2	6.52	afwk	337	TDASDVKPC	345	]	C [345] 57.0215	ENSP00000205948	Beta-2-glycoprotein 1 Precursor (Beta-2-glycoprotein I)(Beta(2)GPI)(B2GPI)(Apolipoprotein H)(Apo-H)(Activated protein C-binding protein)(APC inhibitor)(Anticardiolipin cofactor) [Source: Ur
947.1.1	-8.8	4.24	992.4353	0.0012	2	6.52	afwk	337	TDASDVKPC	345	]	C [345] 57.0215	ENSP00000205948	Beta-2-glycoprotein 1 Precursor (Beta-2-glycoprotein I)(Beta(2)GPI)(B2GPI)(Apolipoprotein H)(Apo-H)(Activated protein C-binding protein)(APC inhibitor)(Anticardiolipin cofactor) [Source: Ur
950.1.1	-6.6	4.52	992.4353	0.0008	2	6.52	afwk	337	TDASDVKPC	345	]	C [345] 57.0215	ENSP00000205948	Beta-2-glycoprotein 1 Precursor (Beta-2-glycoprotein I)(Beta(2)GPI)(B2GPI)(Apolipoprotein H)(Apo-H)(Activated protein C-binding protein)(APC inhibitor)(Anticardiolipin cofactor) [Source: Ur
965.1.1	-4.1	4.76	1041.4306	0.0009	2	13.35	illr	227	GFDQDMANK	235	igeh	M [232] 15.9949	ENSP00000373506	Thioredoxin reductase 1, cytoplasmic (TR)(EC 1.8.1.9)(Thioredoxin reductase TR1)(KM-102-derived reductase-like factor)(Gene associated with retinoid-IFN-induced mortality 12 protein)(GR
967.1.1	-4.1	4.7	1041.4306	0.0007	2	13.35	illr	227	GFDQDMANK	235	igeh	M [232] 15.9949	ENSP00000373506	Thioredoxin reductase 1, cytoplasmic (TR)(EC 1.8.1.9)(Thioredoxin reductase TR1)(KM-102-derived reductase-like factor)(Gene associated with retinoid-IFN-induced mortality 12 protein)(GR
1054.1.1	-2.2	4.39	1015.4302	0.0036	2	13.52	nhlr	144	FCYTGDPR	151	dvll	C [145] 57.0215	ENSP00000254940:reversed	
1088.1.1	-1.4	3.9	902.5128	0.0031	2	8	psck	262	ASCKVPVK	269	katv	C [264] 57.0215, V [266] 14.0157	ENSP00000205948	Beta-2-glycoprotein 1 Precursor (Beta-2-glycoprotein I)(Beta(2)GPI)(B2GPI)(Apolipoprotein H)(Apo-H)(Activated protein C-binding protein)(APC inhibitor)(Anticardiolipin cofactor) [Source: Ur
1091.1.1	-1.6	4	902.5128	0.0008	2	8	psck	262	ASCKVPVK	269	katv	C [264] 57.0215, V [266] 14.0157	ENSP00000205948	Beta-2-glycoprotein 1 Precursor (Beta-2-glycoprotein I)(Beta(2)GPI)(B2GPI)(Apolipoprotein H)(Apo-H)(Activated protein C-binding protein)(APC inhibitor)(Anticardiolipin cofactor) [Source: Ur
1093.1.1	-2.3	4.11	902.5128	0.0012	2	8	psck	262	ASCKVPVK	269	katv	C [264] 57.0215, V [266] 14.0157	ENSP00000205948	Beta-2-glycoprotein 1 Precursor (Beta-2-glycoprotein I)(Beta(2)GPI)(B2GPI)(Apolipoprotein H)(Apo-H)(Activated protein C-binding protein)(APC inhibitor)(Anticardiolipin cofactor) [Source: Ur
1112.1.1	-5.5	4.12	1150.6215	-0.0017	2	12.63	vpvk	270	KATVVYQGER	279	vkiq		ENSP00000205948	Beta-2-glycoprotein 1 Precursor (Beta-2-glycoprotein I)(Beta(2)GPI)(B2GPI)(Apolipoprotein H)(Apo-H)(Activated protein C-binding protein)(APC inhibitor)(Anticardiolipin cofactor) [Source: Ur
1114.1.1	-3.5	4.58	1150.6215	0.0014	2	12.63	vpvk	270	KATVVYQGER	279	vkiq		ENSP00000205948	Beta-2-glycoprotein 1 Precursor (Beta-2-glycoprotein I)(Beta(2)GPI)(B2GPI)(Apolipoprotein H)(Apo-H)(Activated protein C-binding protein)(APC inhibitor)(Anticardiolipin cofactor) [Source: Ur
1115.1.1	-3.4	4.87	1150.6215	0.0011	3	12.63	vpvk	270	KATVVYQGER	279	vkiq		ENSP00000205948	Beta-2-glycoprotein 1 Precursor (Beta-2-glycoprotein I)(Beta(2)GPI)(B2GPI)(Apolipoprotein H)(Apo-H)(Activated protein C-binding protein)(APC inhibitor)(Anticardiolipin cofactor) [Source: Ur
1121.1.1	-5	5.34	1150.6215	0.0006	2	12.63	vpvk	270	KATVVYQGER	279	vkiq		ENSP00000205948	Beta-2-glycoprotein 1 Precursor (Beta-2-glycoprotein I)(Beta(2)GPI)(B2GPI)(Apolipoprotein H)(Apo-H)(Activated protein C-binding protein)(APC inhibitor)(Anticardiolipin cofactor) [Source: Ur
1122.1.1	-3.3	5.5	1150.6215	0.0013	3	12.63	vpvk	270	KATVVYQGER	279	vkiq		ENSP00000205948	Beta-2-glycoprotein 1 Precursor (Beta-2-glycoprotein I)(Beta(2)GPI)(B2GPI)(Apolipoprotein H)(Apo-H)(Activated protein C-binding protein)(APC inhibitor)(Anticardiolipin cofactor) [Source: Ur
1123.1.1	-14.8	3.75	1791.7277	-0.0006	2	1.86	rgsr	491	GGSGGSYGGGGSGGGYGGGSG	513	ggsg		ENSP00000246662	Keratin, type I cytoskeletal 9 (Cytokeratin-9) (CK-9) (Keratin-9) (K9). Source: Uniprot/SWISSPROT P35527
1128.1.1	-3.8	6.01	1150.6215	0.0008	3	12.63	vpvk	270	KATVVYQGER	279	vkiq		ENSP00000205948	Beta-2-glycoprotein 1 Precursor (Beta-2-glycoprotein I)(Beta(2)GPI)(B2GPI)(Apolipoprotein H)(Apo-H)(Activated protein C-binding protein)(APC inhibitor)(Anticardiolipin cofactor) [Source: Ur
1161.1.1	-5.5	3.98	1649.795	0.003	3	9.6	ssyl	12	SRSGGGGGGGLGSGGSIRSS	31	ysrf		ENSP00000246662	Keratin, type I cytoskeletal 9 (Cytokeratin-9) (CK-9) (Keratin-9) (K9). Source: Uniprot/SWISSPROT P35527
1166.1.1	-5.4	4.15	1649.795	0.0021	3	9.6	ssyl	12	SRSGGGGGGGLGSGGSIRSS	31	ysrf		ENSP00000246662	Keratin, type I cytoskeletal 9 (Cytokeratin-9) (CK-9) (Keratin-9) (K9). Source: Uniprot/SWISSPROT P35527
1168.1.1	-2.2	4.11	906.468	0.0011	2	12.07	dkvr	183	FLEQQNK	189	vldt		ENSP00000252252	Keratin, type II cytoskeletal 6B (Cytokeratin-6B) (CK 6B) (K6b keratin). Source: Uniprot/SWISSPROT P04259
1171.1.1	-4.9	4.1	1649.795	0.0008	3	9.6	ssyl	12	SRSGGGGGGGLGSGGSIRSS	31	ysrf		ENSP00000246662	Keratin, type I cytoskeletal 9 (Cytokeratin-9) (CK-9) (Keratin-9) (K9). Source: Uniprot/SWISSPROT P35527
1176.1.1	-3.2	4.01	1235.5287	0.001	2	8.6	gggr	47	FSSSSGYGGGSSR	59	vcgr		ENSP00000246662	Keratin, type I cytoskeletal 9 (Cytokeratin-9) (CK-9) (Keratin-9) (K9). Source: Uniprot/SWISSPROT P35527
1180.1.1	-6.2	4.16	1235.5287	0.0014	2	8.6	gggr	47	FSSSSGYGGGSSR	59	vcgr		ENSP00000246662	Keratin, type I cytoskeletal 9 (Cytokeratin-9) (CK-9) (Keratin-9) (K9). Source: Uniprot/SWISSPROT P35527
1184.1.1	-7.9	4.12	1235.5287	0.0004	2	8.6	gggr	47	FSSSSGYGGGSSR	59	vcgr		ENSP00000246662	Keratin, type I cytoskeletal 9 (Cytokeratin-9) (CK-9) (Keratin-9) (K9). Source: Uniprot/SWISSPROT P35527
1188.1.1	-2.2	4.27	1377.7848	0.0031	3	16.03	vpvk	270	KATVVYQGERVK	281	iqek		ENSP00000205948	Beta-2-glycoprotein 1 Precursor (Beta-2-glycoprotein I)(Beta(2)GPI)(B2GPI)(Apolipoprotein H)(Apo-H)(Activated protein C-binding protein)(APC inhibitor)(Anticardiolipin cofactor) [Source: Ur
1189.1.1	-2.3	4.1	1475.731	0.0039	3	11.6	ssyl	12	SRSGGGGGGGLGSGGSIR	29	ssys		ENSP00000246662	Keratin, type I cytoskeletal 9 (Cytokeratin-9) (CK-9) (Keratin-9) (K9). Source: Uniprot/SWISSPROT P35527
1191.1.1	-2.9	4.46	1377.7848	0.0019	3	16.03	vpvk	270	KATVVYQGERVK	281	iqek		ENSP00000205948	Beta-2-glycoprotein 1 Precursor (Beta-2-glycoprotein I)(Beta(2)GPI)(B2GPI)(Apolipoprotein H)(Apo-H)(Activated protein C-binding protein)(APC inhibitor)(Anticardiolipin cofactor) [Source: Ur
1194.1.1	-2.2	4.51	1377.7848	0.002	3	16.03	vpvk	270	KATVVYQGERVK	281	iqek		ENSP00000205948	Beta-2-glycoprotein 1 Precursor (Beta-2-glycoprotein I)(Beta(2)GPI)(B2GPI)(Apolipoprotein H)(Apo-H)(Activated protein C-binding protein)(APC inhibitor)(Anticardiolipin cofactor) [Source: Ur
1202.1.1	-2.6	3.89	1150.6215	0.0007	2	12.63	vpvk	270	KATVVYQGER	279	vkiq		ENSP00000205948	Beta-2-glycoprotein 1 Precursor (Beta-2-glycoprotein I)(Beta(2)GPI)(B2GPI)(Apolipoprotein H)(Apo-H)(Activated protein C-binding protein)(APC inhibitor)(Anticardiolipin cofactor) [Source: Ur
1208.1.1	-2.3	3.84	1150.6215	0.0001	2	12.63	vpvk	270	KATVVYQGER	279	vkiq		ENSP00000205948	Beta-2-glycoprotein 1 Precursor (Beta-2-glycoprotein I)(Beta(2)GPI)(B2GPI)(Apolipoprotein H)(Apo-H)(Activated protein C-binding protein)(APC inhibitor)(Anticardiolipin cofactor) [Source: Ur
1213.1.1	-1.4	3.82	1150.6215	0.0003	2	12.63	vpvk	270	KATVVYQGER	279	vkiq		ENSP00000205948	Beta-2-glycoprotein 1 Precursor (Beta-2-glycoprotein I)(Beta(2)GPI)(B2GPI)(Apolipoprotein H)(Apo-H)(Activated protein C-binding protein)(APC inhibitor)(Anticardiolipin cofactor) [Source: Ur
1244.1.1	-3.4	4.23	1062.4772	0.0013	2	10.56	ysld	242	GPEEIECTK	250	lgnw	C [248] 57.0215	ENSP00000205948	Beta-2-glycoprotein 1 Precursor (Beta-2-glycoprotein I)(Beta(2)GPI)(B2GPI)(Apolipoprotein H)(Apo-H)(Activated protein C-binding protein)(APC inhibitor)(Anticardiolipin cofactor) [Source: Ur
1250.1.1	-4.1	4.22	1062.4772	0.0013	2	10.56	ysld	242	GPEEIECTK	250	lgnw	C [248] 57.0215	ENSP00000205948	Beta-2-glycoprotein 1 Precursor (Beta-2-glycoprotein I)(Beta(2)GPI)(B2GPI)(Apolipoprotein H)(Apo-H)(Activated protein C-binding protein)(APC inhibitor)(Anticardiolipin cofactor) [Source: Ur
1255.1.1	-2.2	4.23	1062.4772	0.0009	2	10.56	ysld	242	GPEEIECTK	250	lgnw	C [248] 57.0215	ENSP00000205948	Beta-2-glycoprotein 1 Precursor (Beta-2-glycoprotein I)(Beta(2)GPI)(B2GPI)(Apolipoprotein H)(Apo-H)(Activated protein C-binding protein)(APC inhibitor)(Anticardiolipin cofactor) [Source: Ur
1274.1.1	-1.7	3.8	1128.528	-0.0021	2	11.51	aaqk	43	ENAGEDPGLAR	53	qapk		ENSP00000293371	Dermcidin precursor (Preproteolysin) [Contains: Survival-promoting peptide; DCD-1]. Source: Uniprot/SWISSPROT P81605
1389.1.1	-6	6.42	1022.5265	0.0002	2	12.58	pvkk	271	ATVVYQGER	279	vkiq		ENSP00000205948	Beta-2-glycoprotein 1 Precursor (Beta-2-glycoprotein I)(Beta(2)GPI)(B2GPI)(Apolipoprotein H)(Apo-H)(Activated protein C-binding protein)(APC inhibitor)(Anticardiolipin cofactor) [Source: Ur
1393.1.1	-5.5	6.22	1022.5265	0.0009	2	12.58	pvkk	271	ATVVYQGER	279	vkiq		ENSP00000205948	Beta-2-glycoprotein 1 Precursor (Beta-2-glycoprotein I)(Beta(2)GPI)(B2GPI)(Apolipoprotein H)(Apo-H)(Activated protein C-binding protein)(APC inhibitor)(Anticardiolipin cofactor) [Source: Ur
1399.1.1	-6	6.02	1022.5265	0.0004	2	12.58	pvkk	271	ATVVYQGER	279	vkiq		ENSP00000205948	Beta-2-glycoprotein 1 Precursor (Beta-2-glycoprotein I)(Beta(2)GPI)(B2GPI)(Apolipoprotein H)(Apo-H)(Activated protein C-binding protein)(APC inhibitor)(Anticardiolipin cofactor) [Source: Ur
1426.1.1	-2.5	4.29	1249.6899	-0.0003	2	16.13	pvkk	271	ATVVYQGERVK	281	iqek		ENSP00000205948	Beta-2-glycoprotein 1 Precursor (Beta-2-glycoprotein I)(Beta(2)GPI)(B2GPI)(Apolipoprotein H)(Apo-H)(Activated protein C-binding protein)(APC inhibitor)(Anticardiolipin cofactor) [Source: Ur
1431.1.1	-3.8	4.09	1249.6899	0.0009	2	16.13	pvkk	271	ATVVYQGERVK	281	iqek		ENSP00000205948	Beta-2-glycoprotein 1 Precursor (Beta-2-glycoprotein I)(Beta(2)GPI)(B2GPI)(Apolipoprotein H)(Apo-H)(Activated protein C-binding protein)(APC inhibitor)(Anticardiolipin cofactor) [Source: Ur
1432.1.1	-2.6	4.19	1065.4993	0.0019	2	13.79	anek	155	STMQELNSR	163	lasy		ENSP00000246662	Keratin, type I cytoskeletal 9 (Cytokeratin-9) (CK-9) (Keratin-9) (K9). Source: Uniprot/SWISSPROT P35527
1437.1.1	-2.2	3.95	1249.6899	0.0036	2	16.13	pvkk	271	ATVVYQGERVK	281	iqek		ENSP00000205948	Beta-2-glycoprotein 1 Precursor (Beta-2-glycoprotein I)(Beta(2)GPI)(B2GPI)(Apolipoprotein H)(Apo-H)(Activated protein C-binding protein)(APC inhibitor)(Anticardiolipin cofactor) [Source: Ur
1441.1.1	-3.3	4.03	1065.4993	0.0001	2	13.79	anek	155	STMQELNSR	163	lasy		ENSP00000246662	Keratin, type I cytoskeletal 9 (Cytokeratin-9) (CK-9) (Keratin-9) (K9). Source: Uniprot/SWISSPROT P35527
1462.1.1	-3.5	3.9	1065.5211	0.0002	2	11.26	aevk	356	AQYEDIAQK	364	skae		ENSP00000252244	Keratin, type II cytoskeletal 1 (Cytokeratin-1) (CK-1) (Keratin-1) (K1) (67 kDa cytokeratin) (Hair alpha protein). Source: Uniprot/SWISSPROT P04264
1468.1.1	-5.3	4.09	1065.5211	-0.0002	2	11.26	aevk	356	AQYEDIAQK	364	skae		ENSP00000252244	Keratin, type II cytoskeletal 1 (Cytokeratin-1) (CK-1) (Keratin-1) (K1) (67 kDa cytokeratin) (Hair alpha protein). Source: Uniprot/SWISSPROT P04264
1500.1.1	-5.7	4.9	1022.5265	0.0007	2	12.58	pvkk	271	ATVVYQGER	279	vkiq		ENSP00000205948	Beta-2-glycoprotein 1 Precursor (Beta-2-glycoprotein I)(Beta(2)GPI)(B2GPI)(Apolipoprotein H)(Apo-H)(Activated protein C-binding protein)(APC inhibitor)(Anticardiolipin cofactor) [Source: Ur
1506.1.1	-4.1	5.89	1492.634	-0.0057	2	6.52	afwk	337	TDASDVKPC	345	]	C [345] 500.199, C [345] 57.0215	ENSP00000205948	Beta-2-glycoprotein 1 Precursor (Beta-2-glycoprotein I)(Beta(2)GPI)(B2GPI)(Apolipoprotein H)(Apo-H)(Activated protein C-binding protein)(APC inhibitor)(Anticardiolipin cofactor) [Source: Ur
1510.1.1	-2.1	5.83	1492.634	-0.0062	2	6.52	afwk	337	TDASDVKPC	345	]	C [345] 500.199, C [345] 57.0215	ENSP00000205948	Beta-2-glycoprotein 1 Precursor (Beta-2-glycoprotein I)(Beta(2)GPI)(B2GPI)(Apolipoprotein H)(Apo-H)(Activated protein C-binding protein)(APC inhibitor)(Anticardiolipin cofactor) [Source: Ur
1515.1.1	-2	5.63	1492.634	-0.0058	2	6.52	afwk	337	TDASDVKPC	345	]	C [345] 500.199, C [345] 57.0215	ENSP00000205948	Beta-2-glycoprotein 1 Precursor (Beta-2-glycoprotein I)(Beta(2)GPI)(B2GPI)(Apolipoprotein H)(Apo-H)(Activated protein C-binding protein)(APC inhibitor)(Anticardiolipin cofactor) [Source: Ur
1535.1.1	-1.1	3.93	1506.631	1.992	2	15.88	pner	259	SENECLGGVPECR	271	dlqv	C [263] 57.0215, C [270] 57.0215	ENSP00000286800	TRANSCRIPTION REGULATOR PROTEIN BACH1 (BTB AND CNC HOMOLOG 1) (HA2303). [Source: SWISSPROT (O14867)]
1559.1.1	-4.7	3.97	1093.5273	0.0003	2	13	aevk	344	AQYEEIANR	352	srte		ENSP00000252242	Keratin, type II cytoskeletal 5 (Cytokeratin-5) (CK-5) (Keratin-5) (K5) (58 kDa cytokeratin). Source: Uniprot/SWISSPROT P13647
1576.1.1	-1.5	4.78	1015.4302	0.0044	2	13.52	nhlr	144	FCYTGDPR	151	dvll	C [145] 57.0215	ENSP00000254940:reversed	
1580.1.1	-1.9	4.94	1045.5636	0.0016	2	15.5	mlik	98	LSSPATLNSR	107	vatv		sp|TRYP_PIG|	Trypsin; EC 3.4.21.4; Flags: Precursor;
1581.1.1	-1.5	4.75	1015.4302	0.0044	2	13.52	nhlr	144	FCYTGDPR	151	dvll	C [145] 57.0215	ENSP00000254940:reversed	
1588.1.1	-4.3	3.92	1107.5429	0.0023	2	13.01	aevk	339	AQYEEIAQR	347	srae		ENSP00000252252	Keratin, type II cytoskeletal 6B (Cytokeratin-6B) (CK 6B) (K6b keratin). Source: Uniprot/SWISSPROT P04259
1600.1.1	-1.9	4.4	1492.634	-0.0053	2	6.52	afwk	337	TDASDVKPC	345	]	C [345] 500.199, C [345] 57.0215	ENSP00000205948	Beta-2-glycoprotein 1 Precursor (Beta-2-glycoprotein I)(Beta(2)GPI)(B2GPI)(Apolipoprotein H)(Apo-H)(Activated protein C-binding protein)(APC inhibitor)(Anticardiolipin cofactor) [Source: Ur
1601.1.1	-5	4.65	1022.5265	0.0001	2	12.58	pvkk	271	ATVVYQGER	279	vkiq		ENSP00000205948	Beta-2-glycoprotein 1 Precursor (Beta-2-glycoprotein I)(Beta(2)GPI)(B2GPI)(Apolipoprotein H)(Apo-H)(Activated protein C-binding protein)(APC inhibitor)(Anticardiolipin cofactor) [Source: Ur
1607.1.1	-3.5	4.36	1492.634	-0.0039	2	6.52	afwk	337	TDASDVKPC	345	]	C [345] 500.199, C [345] 57.0215	ENSP00000205948	Beta-2-glycoprotein 1 Precursor (Beta-2-glycoprotein I)(Beta(2)GPI)(B2GPI)(Apolipoprotein H)(Apo-H)(Activated protein C-binding protein)(APC inhibitor)(Anticardiolipin cofactor) [Source: Ur
1609.1.1	-4.6	4.61	1022.5265	0.0002	2	12.58	pvkk	271	ATVVYQGER	279	vkiq		ENSP00000205948	Beta-2-glycoprotein 1 Precursor (Beta-2-glycoprotein I)(Beta(2)GPI)(B2GPI)(Apolipoprotein H)(Apo-H)(Activated protein C-binding protein)(APC inhibitor)(Anticardiolipin cofactor) [Source: Ur
1615.1.1	-2.2	4.32	1492.634	-0.0053	2	6.52	afwk	337	TDASDVKPC	345	]	C [345] 500.199, C [345] 57.0215	ENSP00000205948	Beta-2-glycoprotein 1 Precursor (Beta-2-glycoprotein I)(Beta(2)GPI)(B2GPI)(Apolipoprotein H)(Apo-H)(Activated protein C-binding protein)(APC inhibitor)(Anticardiolipin cofactor) [Source: Ur
1616.1.1	-5.4	4.59	1022.5265	0.0003	2	12.58	pvkk	271	ATVVYQGER	279	vkiq		ENSP00000205948	Beta-2-glycoprotein 1 Precursor (Beta-2-glycoprotein I)(Beta(2)GPI)(B2GPI)(Apolipoprotein H)(Apo-H)(Activated protein C-binding protein)(APC inhibitor)(Anticardiolipin cofactor) [Source: Ur
1630.1.1	-3.6	4.22	1090.531	0.0041	2	14.79	gnek	148	VTMQNLNDR	156	lasy		ENSP00000269576	Keratin, type I cytoskeletal 10 (Cytokeratin-10) (CK-10) (Keratin-10) (K10). Source: Uniprot/SWISSPROT P13645
1634.1.1	-4.1	4.41	1090.531	0.0037	2	14.79	gnek	148	VTMQNLNDR	156	lasy		ENSP00000269576	Keratin, type I cytoskeletal 10 (Cytokeratin-10) (CK-10) (Keratin-10) (K10). Source: Uniprot/SWISSPROT P13645
1637.1.1	-4.2	4.45	1090.531	0.0031	2	14.79	gnek	148	VTMQNLNDR	156	lasy		ENSP00000269576	Keratin, type I cytoskeletal 10 (Cytokeratin-10) (CK-10) (Keratin-10) (K10). Source: Uniprot/SWISSPROT P13645
1638.1.1	-2.3	3.93	2318.0176	0.0002	3	29.81	illr	227	GFDQDMANKIGEHMEEHGIK	246	firq	M [232] 15.9949, M [240] 15.9949	ENSP00000373506	Thioredoxin reductase 1, cytoplasmic (TR)(EC 1.8.1.9)(Thioredoxin reductase TR1)(KM-102-derived reductase-like factor)(Gene associated with retinoid-IFN-induced mortality 12 protein)(GR
1673.1.1	-1.4	4.68	1048.5422	0.0018	2	19.65	askk	157	ESVVYGGISR	166	lqad	E [157] -18.0106	ENSP00000387110	
1680.1.1	-2.8	4.15	1562.6759	-0.0036	2	10.56	ysld	242	GPEEIECTK	250	lgnw	C [248] 500.199, C [248] 57.0215	ENSP00000205948	Beta-2-glycoprotein 1 Precursor (Beta-2-glycoprotein I)(Beta(2)GPI)(B2GPI)(Apolipoprotein H)(Apo-H)(Activated protein C-binding protein)(APC inhibitor)(Anticardiolipin cofactor) [Source: Ur
1693.1.1	-5.6	4.48	1022.5265	0.0001	2	12.58	pvkk	271	ATVVYQGER	279	vkiq		ENSP00000205948	Beta-2-glycoprotein 1 Precursor (Beta-2-glycoprotein I)(Beta(2)GPI)(B2GPI)(Apolipoprotein H)(Apo-H)(Activated protein C-binding protein)(APC inhibitor)(Anticardiolipin cofactor) [Source: Ur
1701.1.1	-3	4.4	1022.5265	0.0013	2	12.58	pvkk	271	ATVVYQGER	279	vkiq		ENSP00000205948	Beta-2-glycoprotein 1 Precursor (Beta-2-glycoprotein I)(Beta(2)GPI)(B2GPI)(Apolipoprotein H)(Apo-H)(Activated protein C-binding protein)(APC inhibitor)(Anticardiolipin cofactor) [Source: Ur
1702.1.1	-3.3	4.12	1492.634	-0.0041	2	6.52	afwk	337	TDASDVKPC	345	]	C [345] 500.199, C [345] 57.0215	ENSP00000205948	Beta-2-glycoprotein 1 Precursor (Beta-2-glycoprotein I)(Beta(2)GPI)(B2GPI)(Apolipoprotein H)(Apo-H)(Activated protein C-binding protein)(APC inhibitor)(Anticardiolipin cofactor) [Source: Ur
1708.1.1	-6.5	4.41	1022.5265	0.0008	2	12.58	pvkk	271	ATVVYQGER	279	vkiq		ENSP00000205948	Beta-2-glycoprotein 1 Precursor (Beta-2-glycoprotein I)(Beta(2)GPI)(B2GPI)(Apolipoprotein H)(Apo-H)(Activated protein C-binding protein)(APC inhibitor)(Anticardiolipin cofactor) [Source: Ur

1709.1.1	-3.3	4.15	1492.634	-0.0069	2	6.52	afwk	337	TDASDVKPC	345	]	C [345] 500.199, C [345] 57.0215	ENSP00000205948	Beta-2-glycoprotein 1 Precursor (Beta-2-glycoprotein I)(Beta(2)GPI)(B2GPI)(Apolipoprotein H)(Apo-H)(Activated protein C-binding protein)(APC inhibitor)(Anticardiolipin cofactor) [Source: Ur
1712.1.1	-1.3	4.15	1492.634	-0.0063	2	6.52	afwk	337	TDASDVKPC	345	]	C [345] 500.199, C [345] 57.0215	ENSP00000205948	Beta-2-glycoprotein 1 Precursor (Beta-2-glycoprotein I)(Beta(2)GPI)(B2GPI)(Apolipoprotein H)(Apo-H)(Activated protein C-binding protein)(APC inhibitor)(Anticardiolipin cofactor) [Source: Ur
1714.1.1	-2.1	3.99	809.4403	0.001	2	16.24	lnsr	164	LASYLDK	170	vqal		ENSP00000246662	Keratin, type I cytoskeletal 9 (Cytokeratin-9) (CK-9) (Keratin-9) (K9). Source: Uniprot/SWISSPROT P35527
1781.1.1	-1.2	4.49	1747.9701	0.0011	3	22.15	pvkk	271	ATVVYQGERVKIQEK	285	fkng		ENSP00000205948	Beta-2-glycoprotein 1 Precursor (Beta-2-glycoprotein I)(Beta(2)GPI)(B2GPI)(Apolipoprotein H)(Apo-H)(Activated protein C-binding protein)(APC inhibitor)(Anticardiolipin cofactor) [Source: Ur
1782.1.1	-4.4	4.25	1022.5265	0.0013	2	12.58	pvkk	271	ATVVYQGER	279	vkiq		ENSP00000205948	Beta-2-glycoprotein 1 Precursor (Beta-2-glycoprotein I)(Beta(2)GPI)(B2GPI)(Apolipoprotein H)(Apo-H)(Activated protein C-binding protein)(APC inhibitor)(Anticardiolipin cofactor) [Source: Ur
1788.1.1	-1.1	4.52	1747.9701	0.0006	3	22.15	pvkk	271	ATVVYQGERVKIQEK	285	fkng		ENSP00000205948	Beta-2-glycoprotein 1 Precursor (Beta-2-glycoprotein I)(Beta(2)GPI)(B2GPI)(Apolipoprotein H)(Apo-H)(Activated protein C-binding protein)(APC inhibitor)(Anticardiolipin cofactor) [Source: Ur
1790.1.1	-1.4	4.15	1492.634	-0.0009	2	6.52	afwk	337	TDASDVKPC	345	]	C [345] 500.199, C [345] 57.0215	ENSP00000205948	Beta-2-glycoprotein 1 Precursor (Beta-2-glycoprotein I)(Beta(2)GPI)(B2GPI)(Apolipoprotein H)(Apo-H)(Activated protein C-binding protein)(APC inhibitor)(Anticardiolipin cofactor) [Source: Ur
1791.1.1	-3.8	4.19	1022.5265	0.0011	2	12.58	pvkk	271	ATVVYQGER	279	vkiq		ENSP00000205948	Beta-2-glycoprotein 1 Precursor (Beta-2-glycoprotein I)(Beta(2)GPI)(B2GPI)(Apolipoprotein H)(Apo-H)(Activated protein C-binding protein)(APC inhibitor)(Anticardiolipin cofactor) [Source: Ur
1796.1.1	-1.4	4.25	1492.634	-0.0044	2	6.52	afwk	337	TDASDVKPC	345	]	C [345] 500.199, C [345] 57.0215	ENSP00000205948	Beta-2-glycoprotein 1 Precursor (Beta-2-glycoprotein I)(Beta(2)GPI)(B2GPI)(Apolipoprotein H)(Apo-H)(Activated protein C-binding protein)(APC inhibitor)(Anticardiolipin cofactor) [Source: Ur
1801.1.1	-1.7	4.18	1492.634	-0.0041	2	6.52	afwk	337	TDASDVKPC	345	]	C [345] 500.199, C [345] 57.0215	ENSP00000205948	Beta-2-glycoprotein 1 Precursor (Beta-2-glycoprotein I)(Beta(2)GPI)(B2GPI)(Apolipoprotein H)(Apo-H)(Activated protein C-binding protein)(APC inhibitor)(Anticardiolipin cofactor) [Source: Ur
1803.1.1	-2.6	4.07	1022.5265	0.0013	2	12.58	pvkk	271	ATVVYQGER	279	vkiq		ENSP00000205948	Beta-2-glycoprotein 1 Precursor (Beta-2-glycoprotein I)(Beta(2)GPI)(B2GPI)(Apolipoprotein H)(Apo-H)(Activated protein C-binding protein)(APC inhibitor)(Anticardiolipin cofactor) [Source: Ur
1843.1.1	-2.2	4.23	928.5211	0.0008	2	19.91	cpfa	88	GILENGAVR	96	yttf		ENSP00000205948	Beta-2-glycoprotein 1 Precursor (Beta-2-glycoprotein I)(Beta(2)GPI)(B2GPI)(Apolipoprotein H)(Apo-H)(Activated protein C-binding protein)(APC inhibitor)(Anticardiolipin cofactor) [Source: Ur
1872.1.1	-1.7	4.19	842.5094	0.0003	2	18.3	lnsr	108	VATVSLPR	115	scaa		sp|TRYP_PIG|	Trypsin; EC 3.4.21.4; Flags: Precursor;
1876.1.1	-3.8	4.11	1022.5265	0.0019	2	12.58	pvkk	271	ATVVYQGER	279	vkiq		ENSP00000205948	Beta-2-glycoprotein 1 Precursor (Beta-2-glycoprotein I)(Beta(2)GPI)(B2GPI)(Apolipoprotein H)(Apo-H)(Activated protein C-binding protein)(APC inhibitor)(Anticardiolipin cofactor) [Source: Ur
1877.1.1	-1.6	3.87	1492.634	-0.0029	2	6.52	afwk	337	TDASDVKPC	345	]	C [345] 500.199, C [345] 57.0215	ENSP00000205948	Beta-2-glycoprotein 1 Precursor (Beta-2-glycoprotein I)(Beta(2)GPI)(B2GPI)(Apolipoprotein H)(Apo-H)(Activated protein C-binding protein)(APC inhibitor)(Anticardiolipin cofactor) [Source: Ur
1878.1.1	-1.1	4.24	842.5094	0.0014	2	18.3	lnsr	108	VATVSLPR	115	scaa		sp|TRYP_PIG|	Trypsin; EC 3.4.21.4; Flags: Precursor;
1882.1.1	-3.4	4.15	1022.5265	0.0028	2	12.58	pvkk	271	ATVVYQGER	279	vkiq		ENSP00000205948	Beta-2-glycoprotein 1 Precursor (Beta-2-glycoprotein I)(Beta(2)GPI)(B2GPI)(Apolipoprotein H)(Apo-H)(Activated protein C-binding protein)(APC inhibitor)(Anticardiolipin cofactor) [Source: Ur
1890.1.1	-3.9	4.25	1022.5265	0.0001	2	12.58	pvkk	271	ATVVYQGER	279	vkiq		ENSP00000205948	Beta-2-glycoprotein 1 Precursor (Beta-2-glycoprotein I)(Beta(2)GPI)(B2GPI)(Apolipoprotein H)(Apo-H)(Activated protein C-binding protein)(APC inhibitor)(Anticardiolipin cofactor) [Source: Ur
1893.1.1	-3.1	4.3	999.5582	0.0003	2	20.12	vcpf	87	AGILENGAVR	96	yttf		ENSP00000205948	Beta-2-glycoprotein 1 Precursor (Beta-2-glycoprotein I)(Beta(2)GPI)(B2GPI)(Apolipoprotein H)(Apo-H)(Activated protein C-binding protein)(APC inhibitor)(Anticardiolipin cofactor) [Source: Ur
1903.1.1	-2.5	4.55	999.5582	0.0012	2	20.12	vcpf	87	AGILENGAVR	96	yttf		ENSP00000205948	Beta-2-glycoprotein 1 Precursor (Beta-2-glycoprotein I)(Beta(2)GPI)(B2GPI)(Apolipoprotein H)(Apo-H)(Activated protein C-binding protein)(APC inhibitor)(Anticardiolipin cofactor) [Source: Ur
1907.1.1	-1.3	3.89	1492.634	-0.0058	2	6.52	afwk	337	TDASDVKPC	345	]	C [345] 500.199, C [345] 57.0215	ENSP00000205948	Beta-2-glycoprotein 1 Precursor (Beta-2-glycoprotein I)(Beta(2)GPI)(B2GPI)(Apolipoprotein H)(Apo-H)(Activated protein C-binding protein)(APC inhibitor)(Anticardiolipin cofactor) [Source: Ur
1922.1.1	-1.5	3.9	1492.634	-0.0041	2	6.52	afwk	337	TDASDVKPC	345	]	C [345] 500.199, C [345] 57.0215	ENSP00000205948	Beta-2-glycoprotein 1 Precursor (Beta-2-glycoprotein I)(Beta(2)GPI)(B2GPI)(Apolipoprotein H)(Apo-H)(Activated protein C-binding protein)(APC inhibitor)(Anticardiolipin cofactor) [Source: Ur
1937.1.1	-1.2	4.51	1393.7322	-0.0004	3	22.63	inkr	278	TNAENEFVTIKK	289	dvdg		ENSP00000252244	Keratin, type II cytoskeletal 1 (Cytokeratin-1) (CK-1) (Keratin-1) (K1) (67 kDa cytokeratin) (Hair alpha protein). Source: Uniprot/SWISSPROT P04264
1938.1.1	-1.9	4.25	1234.679	0.0021	3	23.36	ddfr	236	LKYENEVALR	245	qsve		ENSP00000269576	Keratin, type I cytoskeletal 10 (Cytokeratin-10) (CK-10) (Keratin-10) (K10). Source: Uniprot/SWISSPROT P13645
1940.1.1	-1.2	3.97	1234.679	-0.0042	2	23.36	ddfr	236	LKYENEVALR	245	qsve		ENSP00000269576	Keratin, type I cytoskeletal 10 (Cytokeratin-10) (CK-10) (Keratin-10) (K10). Source: Uniprot/SWISSPROT P13645
1954.1.1	-7.3	4.15	1381.6482	-0.0019	2	21.44	dkvr	166	ALEESNYELEGK	177	ikew		ENSP00000269576	Keratin, type I cytoskeletal 10 (Cytokeratin-10) (CK-10) (Keratin-10) (K10). Source: Uniprot/SWISSPROT P13645
1958.1.1	-6.9	4.33	1381.6482	-0.002	2	21.44	dkvr	166	ALEESNYELEGK	177	ikew		ENSP00000269576	Keratin, type I cytoskeletal 10 (Cytokeratin-10) (CK-10) (Keratin-10) (K10). Source: Uniprot/SWISSPROT P13645
1964.1.1	-1.9	4.37	874.4993	0.0013	2	18.24	fgsr	66	SLVNLGGSK	74	sisi		ENSP00000252244	Keratin, type II cytoskeletal 1 (Cytokeratin-1) (CK-1) (Keratin-1) (K1) (67 kDa cytokeratin) (Hair alpha protein). Source: Uniprot/SWISSPROT P04264
1969.1.1	-3.8	4.38	874.4993	0.001	2	18.24	fgsr	66	SLVNLGGSK	74	sisi		ENSP00000252244	Keratin, type II cytoskeletal 1 (Cytokeratin-1) (CK-1) (Keratin-1) (K1) (67 kDa cytokeratin) (Hair alpha protein). Source: Uniprot/SWISSPROT P04264
1976.1.1	-1.5	4.62	832.4887	0.0004	2	19.2	ggsk	75	SISISVAR	82	gggr		ENSP00000252244	Keratin, type II cytoskeletal 1 (Cytokeratin-1) (CK-1) (Keratin-1) (K1) (67 kDa cytokeratin) (Hair alpha protein). Source: Uniprot/SWISSPROT P04264
1978.1.1	-5	4.02	1022.5265	0.0028	2	12.58	pvkk	271	ATVVYQGER	279	vkiq		ENSP00000205948	Beta-2-glycoprotein 1 Precursor (Beta-2-glycoprotein I)(Beta(2)GPI)(B2GPI)(Apolipoprotein H)(Apo-H)(Activated protein C-binding protein)(APC inhibitor)(Anticardiolipin cofactor) [Source: Ur
1984.1.1	-4.1	4.04	1022.5265	-0.0013	2	12.58	pvkk	271	ATVVYQGER	279	vkiq		ENSP00000205948	Beta-2-glycoprotein 1 Precursor (Beta-2-glycoprotein I)(Beta(2)GPI)(B2GPI)(Apolipoprotein H)(Apo-H)(Activated protein C-binding protein)(APC inhibitor)(Anticardiolipin cofactor) [Source: Ur
1991.1.1	-4.5	4.65	1159.6582	0.0008	2	23.91	saer	157	FLIATGERPR	166	ylgi		ENSP00000373506	Thioredoxin reductase 1, cytoplasmic (TR)(EC 1.8.1.9)(Thioredoxin reductase TR1)(KM-102-derived reductase-like factor)(Gene associated with retinoid-IFN-induced mortality 12 protein)(GR
1996.1.1	-4.7	5.08	1159.6582	0.0002	2	23.91	saer	157	FLIATGERPR	166	ylgi		ENSP00000373506	Thioredoxin reductase 1, cytoplasmic (TR)(EC 1.8.1.9)(Thioredoxin reductase TR1)(KM-102-derived reductase-like factor)(Gene associated with retinoid-IFN-induced mortality 12 protein)(GR
2000.1.1	-4.7	4.1	1022.5265	0.0006	2	12.58	pvkk	271	ATVVYQGER	279	vkiq		ENSP00000205948	Beta-2-glycoprotein 1 Precursor (Beta-2-glycoprotein I)(Beta(2)GPI)(B2GPI)(Apolipoprotein H)(Apo-H)(Activated protein C-binding protein)(APC inhibitor)(Anticardiolipin cofactor) [Source: Ur
2072.1.1	-3.3	3.82	1159.6582	-0.0018	2	23.91	saer	157	FLIATGERPR	166	ylgi		ENSP00000373506	Thioredoxin reductase 1, cytoplasmic (TR)(EC 1.8.1.9)(Thioredoxin reductase TR1)(KM-102-derived reductase-like factor)(Gene associated with retinoid-IFN-induced mortality 12 protein)(GR
2074.1.1	-5	4.53	1655.7669	0.0008	3	31.46	ekfk	288	NGMLHGDKVSFFCK	301	nkek	C [300] 57.0215, M [290] 15.9949	ENSP00000205948	Beta-2-glycoprotein 1 Precursor (Beta-2-glycoprotein I)(Beta(2)GPI)(B2GPI)(Apolipoprotein H)(Apo-H)(Activated protein C-binding protein)(APC inhibitor)(Anticardiolipin cofactor) [Source: Ur
2077.1.1	-5.7	4.75	1655.7669	0.0015	3	31.46	ekfk	288	NGMLHGDKVSFFCK	301	nkek	C [300] 57.0215, M [290] 15.9949	ENSP00000205948	Beta-2-glycoprotein 1 Precursor (Beta-2-glycoprotein I)(Beta(2)GPI)(B2GPI)(Apolipoprotein H)(Apo-H)(Activated protein C-binding protein)(APC inhibitor)(Anticardiolipin cofactor) [Source: Ur
2082.1.1	-1.6	3.88	1159.6582	0.0022	2	23.91	saer	157	FLIATGERPR	166	ylgi		ENSP00000373506	Thioredoxin reductase 1, cytoplasmic (TR)(EC 1.8.1.9)(Thioredoxin reductase TR1)(KM-102-derived reductase-like factor)(Gene associated with retinoid-IFN-induced mortality 12 protein)(GR
2084.1.1	-3.6	5.13	1655.7669	-0.0005	3	31.46	ekfk	288	NGMLHGDKVSFFCK	301	nkek	C [300] 57.0215, M [290] 15.9949	ENSP00000205948	Beta-2-glycoprotein 1 Precursor (Beta-2-glycoprotein I)(Beta(2)GPI)(B2GPI)(Apolipoprotein H)(Apo-H)(Activated protein C-binding protein)(APC inhibitor)(Anticardiolipin cofactor) [Source: Ur
2089.1.1	-7.6	4.22	1475.7853	-0.001	2	24.75	dkvr	200	FLEQQNQVLQTK	211	well		ENSP00000252244	Keratin, type II cytoskeletal 1 (Cytokeratin-1) (CK-1) (Keratin-1) (K1) (67 kDa cytokeratin) (Hair alpha protein). Source: Uniprot/SWISSPROT P04264
2092.1.1	-4.5	3.83	1159.6582	0.0043	2	23.91	saer	157	FLIATGERPR	166	ylgi		ENSP00000373506	Thioredoxin reductase 1, cytoplasmic (TR)(EC 1.8.1.9)(Thioredoxin reductase TR1)(KM-102-derived reductase-like factor)(Gene associated with retinoid-IFN-induced mortality 12 protein)(GR
2096.1.1	-9.5	4.32	1475.7853	-0.0002	2	24.75	dkvr	200	FLEQQNQVLQTK	211	well		ENSP00000252244	Keratin, type II cytoskeletal 1 (Cytokeratin-1) (CK-1) (Keratin-1) (K1) (67 kDa cytokeratin) (Hair alpha protein). Source: Uniprot/SWISSPROT P04264
2099.1.1	-1.6	3.85	1020.4779	0.0037	2	8.94	rarr	117	VAEMEAQSR	125	alql		ENSP00000341451	
2103.1.1	-7.1	4.28	1475.7853	-0.0021	2	24.75	dkvr	200	FLEQQNQVLQTK	211	well		ENSP00000252244	Keratin, type II cytoskeletal 1 (Cytokeratin-1) (CK-1) (Keratin-1) (K1) (67 kDa cytokeratin) (Hair alpha protein). Source: Uniprot/SWISSPROT P04264
2113.1.1	-1.1	5.1	1930.9302	0.0007	4	34.25	iqek	286	FKNGMLHGDKVSFFCK	301	nkek	C [300] 57.0215, M [290] 15.9949	ENSP00000205948	Beta-2-glycoprotein 1 Precursor (Beta-2-glycoprotein I)(Beta(2)GPI)(B2GPI)(Apolipoprotein H)(Apo-H)(Activated protein C-binding protein)(APC inhibitor)(Anticardiolipin cofactor) [Source: Ur
2116.1.1	-2.2	4.46	1930.9302	-0.0085	3	34.25	iqek	286	FKNGMLHGDKVSFFCK	301	nkek	C [300] 57.0215, M [290] 15.9949	ENSP00000205948	Beta-2-glycoprotein 1 Precursor (Beta-2-glycoprotein I)(Beta(2)GPI)(B2GPI)(Apolipoprotein H)(Apo-H)(Activated protein C-binding protein)(APC inhibitor)(Anticardiolipin cofactor) [Source: Ur
2121.1.1	-1.7	4.36	1930.9302	-0.0043	3	34.25	iqek	286	FKNGMLHGDKVSFFCK	301	nkek	C [300] 57.0215, M [290] 15.9949	ENSP00000205948	Beta-2-glycoprotein 1 Precursor (Beta-2-glycoprotein I)(Beta(2)GPI)(B2GPI)(Apolipoprotein H)(Apo-H)(Activated protein C-binding protein)(APC inhibitor)(Anticardiolipin cofactor) [Source: Ur
2136.1.1	-2.2	4.56	862.4669	0.0012	2	18.8	erpr	167	YLGIPGDK	174	eyci		ENSP00000373506	Thioredoxin reductase 1, cytoplasmic (TR)(EC 1.8.1.9)(Thioredoxin reductase TR1)(KM-102-derived reductase-like factor)(Gene associated with retinoid-IFN-induced mortality 12 protein)(GR
2141.1.1	-2.6	4.53	862.4669	0.0002	2	18.8	erpr	167	YLGIPGDK	174	eyci		ENSP00000373506	Thioredoxin reductase 1, cytoplasmic (TR)(EC 1.8.1.9)(Thioredoxin reductase TR1)(KM-102-derived reductase-like factor)(Gene associated with retinoid-IFN-induced mortality 12 protein)(GR
2145.1.1	-2.7	4.62	862.4669	0.0009	2	18.8	erpr	167	YLGIPGDK	174	eyci		ENSP00000373506	Thioredoxin reductase 1, cytoplasmic (TR)(EC 1.8.1.9)(Thioredoxin reductase TR1)(KM-102-derived reductase-like factor)(Gene associated with retinoid-IFN-induced mortality 12 protein)(GR
2154.1.1	-1.4	3.93	1111.5742	0.0029	2	20.65	gskr	74	ISISTSGGSFR	84	nrfg		ENSP00000252242	Keratin, type II cytoskeletal 5 (Cytokeratin-5) (CK-5) (Keratin-5) (K5) (58 kDa cytokeratin). Source: Uniprot/SWISSPROT P13647
2176.1.1	-6.7	4.21	1179.6004	-0.0003	2	21.27	yqsk	377	YEELQITAGR	386	hgds		ENSP00000252244	Keratin, type II cytoskeletal 1 (Cytokeratin-1) (CK-1) (Keratin-1) (K1) (67 kDa cytokeratin) (Hair alpha protein). Source: Uniprot/SWISSPROT P04264
2181.1.1	-6.5	4.24	1179.6004	-0.0015	2	21.27	yqsk	377	YEELQITAGR	386	hgds		ENSP00000252244	Keratin, type II cytoskeletal 1 (Cytokeratin-1) (CK-1) (Keratin-1) (K1) (67 kDa cytokeratin) (Hair alpha protein). Source: Uniprot/SWISSPROT P04264
2194.1.1	-1.6	4.96	1931.915	0.0009	4	34.25	iqek	286	FKNGMLHGDKVSFFCK	301	nkek	C [300] 57.0215, M [290] 15.9949, N [288]	ENSP00000205948	Beta-2-glycoprotein 1 Precursor (Beta-2-glycoprotein I)(Beta(2)GPI)(B2GPI)(Apolipoprotein H)(Apo-H)(Activated protein C-binding protein)(APC inhibitor)(Anticardiolipin cofactor) [Source: Ur
2196.1.1	-3.2	3.99	1179.6004	-0.0013	2	21.27	yqsk	377	YEELQITAGR	386	hgds		ENSP00000252244	Keratin, type II cytoskeletal 1 (Cytokeratin-1) (CK-1) (Keratin-1) (K1) (67 kDa cytokeratin) (Hair alpha protein). Source: Uniprot/SWISSPROT P04264
2200.1.1	-1.1	4.4	1931.915	-0.0027	3	34.25	iqek	286	FKNGMLHGDKVSFFCK	301	nkek	C [300] 57.0215, M [290] 15.9949, N [288]	ENSP00000205948	Beta-2-glycoprotein 1 Precursor (Beta-2-glycoprotein I)(Beta(2)GPI)(B2GPI)(Apolipoprotein H)(Apo-H)(Activated protein C-binding protein)(APC inhibitor)(Anticardiolipin cofactor) [Source: Ur
2204.1.1	-3.5	3.88	1243.5953	0.0005	2	22.86	fscn	112	TGFYLNGADSAK	123	ctee		ENSP00000205948	Beta-2-glycoprotein 1 Precursor (Beta-2-glycoprotein I)(Beta(2)GPI)(B2GPI)(Apolipoprotein H)(Apo-H)(Activated protein C-binding protein)(APC inhibitor)(Anticardiolipin cofactor) [Source: Ur
2207.1.1	-5.4	4.99	1931.915	0.0009	3	34.25	iqek	286	FKNGMLHGDKVSFFCK	301	nkek	C [300] 57.0215, M [290] 15.9949, N [288]	ENSP00000205948	Beta-2-glycoprotein 1 Precursor (Beta-2-glycoprotein I)(Beta(2)GPI)(B2GPI)(Apolipoprotein H)(Apo-H)(Activated protein C-binding protein)(APC inhibitor)(Anticardiolipin cofactor) [Source: Ur
2210.1.1	-11.8	3.93	1765.7347	-0.0019	2	23.85	gggr	46	FSSCGGGGGSFGAGGGFGSR	65	slvn	C [49] 57.0215	ENSP00000252244	Keratin, type II cytoskeletal 1 (Cytokeratin-1) (CK-1) (Keratin-1) (K1) (67 kDa cytokeratin) (Hair alpha protein). Source: Uniprot/SWISSPROT P04264
2279.1.1	-1.7	5.14	1656.7517	0.0018	4	31.46	ekfk	288	NGMLHGDKVSFFCK	301	nkek	C [300] 57.0215, M [290] 15.9949, N [288]	ENSP00000205948	Beta-2-glycoprotein 1 Precursor (Beta-2-glycoprotein I)(Beta(2)GPI)(B2GPI)(Apolipoprotein H)(Apo-H)(Activated protein C-binding protein)(APC inhibitor)(Anticardiolipin cofactor) [Source: Ur
2285.1.1	-1.3	4.7	787.3807	0.0018	2	19.77	hgdk	296	VSFFCK	301	nkek	C [300] 57.0215	ENSP00000205948	Beta-2-glycoprotein 1 Precursor (Beta-2-glycoprotein I)(Beta(2)GPI)(B2GPI)(Apolipoprotein H)(Apo-H)(Activated protein C-binding protein)(APC inhibitor)(Anticardiolipin cofactor) [Source: Ur
2302.1.1	-3.8	4.61	1656.7517	-0.0017	3	31.46	ekfk	288	NGMLHGDKVSFFCK	301	nkek	C [300] 57.0215, M [290] 15.9949, N [288]	ENSP00000205948	Beta-2-glycoprotein 1 Precursor (Beta-2-glycoprotein I)(Beta(2)GPI)(B2GPI)(Apolipoprotein H)(Apo-H)(Activated protein C-binding protein)(APC inhibitor)(Anticardiolipin cofactor) [Source: Ur
2312.1.1	-3.8	4.3	1656.7517	-0.0006	3	31.46	ekfk	288	NGMLHGDKVSFFCK	301	nkek	C [300] 57.0215, M [290] 15.9949, N [288]	ENSP00000205948	Beta-2-glycoprotein 1 Precursor (Beta-2-glycoprotein I)(Beta(2)GPI)(B2GPI)(Apolipoprotein H)(Apo-H)(Activated protein C-binding protein)(APC inhibitor)(Anticardiolipin cofactor) [Source: Ur
2315.1.1	-1.3	4.92	1486.631	-0.0053	2	22.76	sqsr	191	CCVPVCYQCKI	201	]	C [191] 57.0215, C [192] 57.0215, C [196] !	ENSP00000355351	Keratin, ultra high-sulfur matrix protein A (UHS keratin A) (UHS KerA). [Source: UniProt P26371]
2321.1.1	-2	5.05	1486.631	-0.0029	2	22.76	sqsr	191	CCVPVCYQCKI	201	]	C [191] 57.0215, C [192] 57.0215, C [196] !	ENSP00000355351	Keratin, ultra high-sulfur matrix protein A (UHS keratin A) (UHS KerA). [Source: UniProt P26371]
2328.1.1	-2.5	4.04	1830.8658	-0.0038	2	21.18	yted	313	AQCIDGTIEVPK	324	cfke	C [315] 500.199, C [315] 57.0215	ENSP00000205948	Beta-2-glycoprotein 1 Precursor (Beta-2-glycoprotein I)(Beta(2)GPI)(B2GPI)(Apolipoprotein H)(Apo-H)(Activated protein C-binding protein)(APC inhibitor)(Anticardiolipin cofactor) [Source: Ur
2332.1.1	-5.5	3.99	1830.8658	-0.0005	2	21.18	yted	313	AQCIDGTIEVPK	324	cfke	C [315] 500.199, C [315] 57.0215	ENSP00000205948	Beta-2-glycoprotein 1 Precursor (Beta-2-glycoprotein I)(Beta(2)GPI)(B2GPI)(Apolipoprotein H)(Apo-H)(Activated protein C-binding protein)(APC inhibitor)(Anticardiolipin cofactor) [Source: Ur
2335.1.1	-7.4	4.02	1597.7051	0.0014	2	23.66	gchd	237	GYSLDGPEEIECTK	250	lgnw	C [248] 57.0215	ENSP00000205948	Beta-2-glycoprotein 1 Precursor (Beta-2-glycoprotein I)(Beta(2)GPI)(B2GPI)(Apolipoprotein H)(Apo-H)(Activated protein C-binding protein)(APC inhibitor)(Anticardiolipin cofactor) [Source: Ur
2347.1.1	-5.2	4.02	1597.7051	0.0013	2	23.66	gchd	237	GYSLDGPEEIECTK	250	lgnw	C [248] 57.0215	ENSP00000205948	Beta-2-glycoprotein 1 Precursor (Beta-2-glycoprotein I)(Beta(2)GPI)(B2GPI)(Apolipoprotein H)(Apo-H)(Activated protein C-binding protein)(APC inhibitor)(Anticardiolipin cofactor) [Source: Ur
2353.1.1	-4.1	3.98	1597.7051	0.0001	2	23.66	gchd	237	GYSLDGPEEIECTK	250	lgnw	C [248] 57.0215	ENSP00000205948	Beta-2-glycoprotein 1 Precursor (Beta-2-glycoprotein I)(Beta(2)GPI)(B2GPI)(Apolipoprotein H)(Apo-H)(Activated protein C-binding protein)(APC inhibitor)(Anticardiolipin cofactor) [Source: Ur
2360.1.1	-9.5	4.16	2629.1181	0.0036	3	28.85	lyyk	228	DKATFGCHDGYSLDGPEEIECTK	250	lgnw	C [234] 57.0215, C [248] 57.0215	ENSP00000205948	Beta-2-glycoprotein 1 Precursor (Beta-2-glycoprotein I)(Beta(2)GPI)(B2GPI)(Apolipoprotein H)(Apo-H)(Activated protein C-binding protein)(APC inhibitor)(Anticardiolipin cofactor) [Source: Ur
2361.1.1	-4.4	4.47	2629.1181	-0.0005	4	28.85	lyyk	228	DKATFGCHDGYSLDGPEEIECTK	250	lgnw	C [234] 57.0215, C [248] 57.0215	ENSP00000205948	Beta-2-glycoprotein 1 Precursor (Beta-2-glycoprotein I)(Beta(2)GPI)(B2GPI)(Apolipoprotein H)(Apo-H)(Activated protein C-binding protein)(APC inhibitor)(Anticardiolipin cofactor) [Source: Ur
2365.1.1	-11.3	4.38	2629.1181	-0.0005	3	28.85	lyyk	228	DKATFGCHDGYSLDGPEEIECTK	250	lgnw	C [234] 57.0215, C [248] 57.0215	ENSP00000205948	Beta-2-glycoprotein 1 Precursor (Beta-2-glycoprotein I)(Beta(2)GPI)(B2GPI)(Apolipoprotein H)(Apo-H)(Activated protein C-binding protein)(APC inhibitor)(Anticardiolipin cofactor) [Source: Ur
2371.1.1	-3.9	4.85	2629.1181	0.0014	4	28.85	lyyk	228	DKATFGCHDGYSLDGPEEIECTK	250	lgnw	C [234] 57.0215, C [248] 57.0215	ENSP00000205948	Beta-2-glycoprotein 1 Precursor (Beta-2-glycoprotein I)(Beta(2)GPI)(B2GPI)(Apolipoprotein H)(Apo-H)(Activated protein C-binding protein)(APC inhibitor)(Anticardiolipin cofactor) [Source: Ur
2375.1.1	-2.3	4.18	3011.385	-0.012	4	33.21	vvpl	38	KTFYEPGEEITYSCKPGYVSR	58	ggmr	C [51] 500.199, C [51] 57.0215	ENSP00000205948	Beta-2-glycoprotein 1 Precursor (Beta-2-glycoprotein I)(Beta(2)GPI)(B2GPI)(Apolipoprotein H)(Apo-H)(Activated protein C-binding protein)(APC inhibitor)(Anticardiolipin cofactor) [Source: Ur
2377.1.1	-4.3	4.83	2629.1181	-0.0003	4	28.85	lyyk	228	DKATFGCHDGYSLDGPEEIECTK	250	lgnw	C [234] 57.0215, C [248] 57.0215	ENSP00000205948	Beta-2-glycoprotein 1 Precursor (Beta-2-glycoprotein I)(Beta(2)GPI)(B2GPI)(Apolipoprotein H)(Apo-H)(Activated protein C-binding protein)(APC inhibitor)(Anticardiolipin cofactor) [Source: Ur
2378.1.1	-8.2	4.44	2629.1181	-0.003	3	28.85	lyyk	228	DKATFGCHDGYSLDGPEEIECTK	250	lgnw	C [234] 57.0215, C [248] 57.0215	ENSP00000205948	Beta-2-glycoprotein 1 Precursor (Beta-2-glycoprotein I)(Beta(2)GPI)(B2GPI)(Apolipoprotein H)(Apo-H)(Activated protein C-binding protein)(APC inhibitor)(Anticardiolipin cofactor) [Source: Ur
2392.1.1	-3.1	4.34	1352.6845	-0.0007	2	23.15	qdsr	85	NYGWKVEETVK	95	hdwd		ENSP00000373506	Thioredoxin reductase 1, cytoplasmic (TR)(EC 1.8.1.9)(Thioredoxin reductase TR1)(KM-102-derived reductase-like factor)(Gene associated with retinoid-IFN-induced mortality 12 protein)(GR
2398.1.1	-5	4.69	2214.0053	-0.0011	3	26.94	nkek	306	KCSYTEDAQCIDGTIEVPK	324	cfke	C [307] 57.0215, C [315] 57.0215	ENSP00000205948	Beta-2-glycoprotein 1 Precursor (Beta-2-glycoprotein I)(Beta(2)GPI)(B2GPI)(Apolipoprotein H)(Apo-H)(Activated protein C-binding protein)(APC inhibitor)(Anticardiolipin cofactor) [Source: Ur
2400.1.1	-4.5	4.37	1352.6845	0.0006	2	23.15	qdsr	85	NYGWKVEETVK	95	hdwd		ENSP00000373506	Thioredoxin reductase 1, cytoplasmic (TR)(EC 1.8.1.9)(Thioredoxin reductase TR1)(KM-102-derived reductase-like factor)(Gene associated with retinoid-IFN-induced mortality 12 protein)(GR
2404.1.1	-6.7	5.08	2214.0053	-0.0015	3	26.94	nkek	306	KCSYTEDAQCIDGTIEVPK	324	cfke	C [307] 57.0215, C [315] 57.0215	ENSP00000205948	Beta-2-glycoprotein 1 Precursor (Beta-2-glycoprotein I)(Beta(2)GPI)(B2GPI)(Apolipoprotein H)(Apo-H)(Activated protein C-binding protein)(APC inhibitor)(Anticardiolipin cofactor) [Source: Ur
2406.1.1	-2.7	4.2	1352.6845	-0.0007	2	23.15	qdsr	85	NYGWKVEETVK	95	hdwd		ENSP00000373506	Thioredoxin reductase 1, cytoplasmic (TR)(EC 1.8.1.9)(Thioredoxin reductase TR1)(KM-102-derived reductase-like factor)(Gene associated with retinoid-IFN-induced mortality 12 protein)(GR
2410.1.1	-7.7	5.06	2214.0053	0.0013	3	26.94	nkek	306	KCSYTEDAQCIDGTIEVPK	324	cfke	C [307] 57.0215, C [315] 57.0215	ENSP00000205948	Beta-2-glycoprotein 1 Precursor (Beta-2-glycoprotein I)(Beta(2)GPI)(B2GPI)(Apolipoprotein H)(Apo-H)(Activated protein C-binding protein)(APC inhibitor)(Anticardiolipin cofactor) [Source: Ur
2426.1.1	-7.1	3.93	1712.732	0.0012	2	24.13	fgch	236	DGYSLDGPEEIECTK	250	lgnw	C [248] 57.0215	ENSP00000205948	Beta-2-glycoprotein 1 Precursor (Beta-2-glycoprotein I)(Beta(2)GPI)(B2GPI)(Apolipoprotein H)(Apo-H)(Activated protein C-binding protein)(APC inhibitor)(Anticardiolipin cofactor) [Source: Ur
2432.1.1	-1.6	4.29	3129.3168	-0.0088	4	28.85	lyyk	228	DKATFGCHDGYSLDGPEEIECTK	250	lgnw	C [248] 57.0215, C [234] 500.199, C [234] !	ENSP00000205948	Beta-2-glycoprotein 1 Precursor (Beta-2-glycoprotein I)(Beta(2)GPI)(B2GPI)(Apolipoprotein H)(Apo-H)(Activated protein C-binding protein)(APC inhibitor)(Anticardiolipin cofactor) [Source: Ur
2439.1.1	-3.3	4.07	1431.7084	0.0004	2	23.77	gtrw	54	GLGGTCVNVGCIPK	67	klmh	C [59] 57.0215, C [64] 57.0215	ENSP00000373506	Thioredoxin reductase 1, cytoplasmic (TR)(EC 1.8.1.9)(Thioredoxin reductase TR1)(KM-102-derived reductase-like factor)(Gene associated with retinoid-IFN-induced mortality 12 protein)(GR
2444.1.1	-2.4	4.73	3129.317	-0.013	4	28.85	lyyk	228	DKATFGCHDGYSLDGPEEIECTK	250	lgnw	C [248] 57.0215, C [234] 500.199, C [234] !	ENSP00000205948	Beta-2-glycoprotein 1 Precursor (Beta-2-glycoprotein I)(Beta(2)GPI)(B2GPI)(Apolipoprotein H)(Apo-H)(Activated protein C-binding protein)(APC inhibitor)(Anticardiolipin cofactor) [Source: Ur
2451.1.1	-4.5	4.72	3129.3168	-0.0095	4	28.85	lyyk	228	DKATFGCHDGYSLDGPEEIECTK	250	lgnw	C [248] 57.0215, C [234] 500.199, C [234] !	ENSP00000205948	Beta-2-glycoprotein 1 Precursor (Beta-2-glycoprotein I)(Beta(2)GPI)(B2GPI)(Apolipoprotein H)(Apo-H)(Activated protein C-binding protein)(APC inhibitor)(Anticardiolipin cofactor) [Source: Ur
2460.1.1	-4.8	4.77	2385.9962	0.0024	3	29.48	ykdk	230	ATFGCHDGYSLDGPEEIECTK	250	lgnw	C [234] 57.0215, C [248] 57.0215	ENSP00000205948	Beta-2-glycoprotein 1 Precursor (Beta-2-glycoprotein I)(Beta(2)GPI)(B2GPI)(Apolipoprotein H)(Apo-H)(Activated protein C-binding protein)(APC inhibitor)(Anticardiolipin cofactor) [Source: Ur
2463.1.1	-3.4	4.28	3129.3168	0.004	3	28.85	lyyk	228	DKATFGCHDGYSLDGPEEIECTK	250	lgnw	C [248] 57.0215, C [234] 500.199, C [234] !	ENSP00000205948	Beta-2-glycoprotein 1 Precursor (Beta-2-glycoprotein I)(Beta(2)GPI)(B2GPI)(Apolipoprotein H)(Apo-H)(Activated protein C-binding protein)(APC inhibitor)(Anticardiolipin cofactor) [Source: Ur
2466.1.1	-2	4.74	2714.204	-0.0049	3	26.94	nkek	306	KCSYTEDAQCIDGTIEVPK	324	cfke	C [307] 57.0215, C [315] 500.199, C [315] !	ENSP00000205948	Beta-2-glycoprotein 1 Precursor (Beta-2-glycoprotein I)(Beta(2)GPI)(B2GPI)(Apolipoprotein H)(Apo-H)(Activated protein C-binding protein)(APC inhibitor)(Anticardiolipin cofactor) [Source: Ur
2469.1.1	-3.9	4.39	3129.3168	0.0029	3	28.85	lyyk	228	DKATFGCHDGYSLDGPEEIECTK	250	lgnw	C [248] 57.0215, C [234] 500.199, C [234] !	ENSP00000205948	Beta-2-glycoprotein 1 Precursor (Beta-2-glycoprotein I)(Beta(2)GPI)(B2GPI)(Apolipoprotein H)(Apo-H)(Activated protein C-binding protein)(APC inhibitor)(Anticardiolipin cofactor) [Source: Ur
2472.1.1	-3	5.06	2714.204	-0.0018	3	26.94	nkek	306	KCSYTEDAQCIDGTIEVPK	324	cfke	C [307] 57.0215, C [315] 500.199, C [315] !	ENSP00000205948	Beta-2-glycoprotein 1 Precursor (Beta-2-glycoprotein I)(Beta(2)GPI)(B2GPI)(Apolipoprotein H)(Apo-H)(Activated protein C-binding protein)(APC inhibitor)(Anticardiolipin cofactor) [Source: Ur
2473.1.1	-1.4	4.67	1287.5794	-0.0048	2	19.77	hgdk	296	VSFFCK	301	nkek	C [300] 500.199, C [300] 57.0215	ENSP00000205948	Beta-2-glycoprotein 1 Precursor (Beta-2-glycoprotein I)(Beta(2)GPI)(B2GPI)(Apolipoprotein H)(Apo-H)(Activated protein C-binding protein)(APC inhibitor)(Anticardiolipin cofactor) [Source: Ur
2479.1.1	-1.4	4.75	1287.5794	-0.0049	2	19.77	hgdk	296	VSFFCK	301	nkek	C [300] 500.199, C [300] 57.0215	ENSP00000205948	Beta-2-glycoprotein 1 Precursor (Beta-2-glycoprotein I)(Beta(2)GPI)(B2GPI)(Apolipoprotein H)(Apo-H)(Activated protein C-binding protein)(APC inhibitor)(Anticardiolipin cofactor) [Source: Ur

2483.1.1	-1.4	4.66	1539.7413	0.0017	3	33.42	evpk	325	CFKEHSSLAFWK	336	tdas	C [325] 57.0215	ENSP00000205948	Beta-2-glycoprotein 1 Precursor (Beta-2-glycoprotein I)(Beta(2)GPI)(B2GPI)(Apolipoprotein H)(Apo-H)(Activated protein C-binding protein)(APC inhibitor)(Anticardiolipin cofactor) [Source: Ur
2485.1.1	-2.4	4.9	1539.7413	0.0025	3	33.42	evpk	325	CFKEHSSLAFWK	336	tdas	C [325] 57.0215	ENSP00000205948	Beta-2-glycoprotein 1 Precursor (Beta-2-glycoprotein I)(Beta(2)GPI)(B2GPI)(Apolipoprotein H)(Apo-H)(Activated protein C-binding protein)(APC inhibitor)(Anticardiolipin cofactor) [Source: Ur
2492.1.1	-2.2	5.43	1539.7413	0.0014	3	33.42	evpk	325	CFKEHSSLAFWK	336	tdas	C [325] 57.0215	ENSP00000205948	Beta-2-glycoprotein 1 Precursor (Beta-2-glycoprotein I)(Beta(2)GPI)(B2GPI)(Apolipoprotein H)(Apo-H)(Activated protein C-binding protein)(APC inhibitor)(Anticardiolipin cofactor) [Source: Ur
2496.1.1	-7.7	4.26	1723.7521	-0.0001	2	27.37	vplk	39	TFYEPGEEITYSCK	52	pgyv	C [51] 57.0215	ENSP00000205948	Beta-2-glycoprotein 1 Precursor (Beta-2-glycoprotein I)(Beta(2)GPI)(B2GPI)(Apolipoprotein H)(Apo-H)(Activated protein C-binding protein)(APC inhibitor)(Anticardiolipin cofactor) [Source: Ur
2501.1.1	-9.1	4.27	1723.7521	0.0034	2	27.37	vplk	39	TFYEPGEEITYSCK	52	pgyv	C [51] 57.0215	ENSP00000205948	Beta-2-glycoprotein 1 Precursor (Beta-2-glycoprotein I)(Beta(2)GPI)(B2GPI)(Apolipoprotein H)(Apo-H)(Activated protein C-binding protein)(APC inhibitor)(Anticardiolipin cofactor) [Source: Ur
2503.1.1	-12.8	4.61	2383.091	0.013	2	33.69	vplk	39	TFYEPGEEITYSCKPGYVSR	58	ggmr	C [51] 57.0215	ENSP00000205948	Beta-2-glycoprotein 1 Precursor (Beta-2-glycoprotein I)(Beta(2)GPI)(B2GPI)(Apolipoprotein H)(Apo-H)(Activated protein C-binding protein)(APC inhibitor)(Anticardiolipin cofactor) [Source: Ur
2508.1.1	-7.9	4.8	2383.0911	0.0017	2	33.69	vplk	39	TFYEPGEEITYSCKPGYVSR	58	ggmr	C [51] 57.0215	ENSP00000205948	Beta-2-glycoprotein 1 Precursor (Beta-2-glycoprotein I)(Beta(2)GPI)(B2GPI)(Apolipoprotein H)(Apo-H)(Activated protein C-binding protein)(APC inhibitor)(Anticardiolipin cofactor) [Source: Ur
2510.1.1	-2.7	4.43	2293.986	0.994	3	29.97	scgf	54	GGGAGSGFGGGYGGGLGGGYG	83	gfag	F [81] 15.9949	ENSP00000377550	Keratin, type I cytoskeletal 13 (Cytokeratin-13) (CK-13) (Keratin-13) (K13). Source: Uniprot/SWISSPROT P13646
2511.1.1	-4.6	3.97	1723.7521	0.0018	2	27.37	vplk	39	TFYEPGEEITYSCK	52	pgyv	C [51] 57.0215	ENSP00000205948	Beta-2-glycoprotein 1 Precursor (Beta-2-glycoprotein I)(Beta(2)GPI)(B2GPI)(Apolipoprotein H)(Apo-H)(Activated protein C-binding protein)(APC inhibitor)(Anticardiolipin cofactor) [Source: Ur
2513.1.1	-6.4	4.72	2383.0911	-0.0014	2	33.69	vplk	39	TFYEPGEEITYSCKPGYVSR	58	ggmr	C [51] 57.0215	ENSP00000205948	Beta-2-glycoprotein 1 Precursor (Beta-2-glycoprotein I)(Beta(2)GPI)(B2GPI)(Apolipoprotein H)(Apo-H)(Activated protein C-binding protein)(APC inhibitor)(Anticardiolipin cofactor) [Source: Ur
2537.1.1	-1.2	5.37	1870.992	0.012	3	32.58	kfir	250	QFVPIKVEQIEAGTPGR	266	lrvv	Q [250] 0.9848, Q [258] 0.9848	ENSP00000373506	Thioredoxin reductase 1, cytoplasmic (TR)(EC 1.8.1.9)(Thioredoxin reductase TR1)(KM-102-derived reductase-like factor)(Gene associated with retinoid-IFN-induced mortality 12 protein)(GR
2548.1.1	-1.5	5.56	1104.5473	0.0017	3	27.81	kcfk	328	EHSSLAFWK	336	tdas		ENSP00000205948	Beta-2-glycoprotein 1 Precursor (Beta-2-glycoprotein I)(Beta(2)GPI)(B2GPI)(Apolipoprotein H)(Apo-H)(Activated protein C-binding protein)(APC inhibitor)(Anticardiolipin cofactor) [Source: Ur
2550.1.1	-1.1	4.84	2974.4556	-0.0038	4	38.61	revk	205	CPFPSRPDNGFVNYPAKPTLYYKD	229	atfg	C [205] 57.0215	ENSP00000205948	Beta-2-glycoprotein 1 Precursor (Beta-2-glycoprotein I)(Beta(2)GPI)(B2GPI)(Apolipoprotein H)(Apo-H)(Activated protein C-binding protein)(APC inhibitor)(Anticardiolipin cofactor) [Source: Ur
2551.1.1	-1.5	4.87	1870.008	0.996	2	32.58	kfir	250	QFVPIKVEQIEAGTPGR	266	lrvv	Q [258] 0.9848	ENSP00000373506	Thioredoxin reductase 1, cytoplasmic (TR)(EC 1.8.1.9)(Thioredoxin reductase TR1)(KM-102-derived reductase-like factor)(Gene associated with retinoid-IFN-induced mortality 12 protein)(GR
2553.1.1	-4.7	6.16	2883.2898	-0.0034	3	33.69	vplk	39	TFYEPGEEITYSCKPGYVSR	58	ggmr	C [51] 500.199, C [51] 57.0215	ENSP00000205948	Beta-2-glycoprotein 1 Precursor (Beta-2-glycoprotein I)(Beta(2)GPI)(B2GPI)(Apolipoprotein H)(Apo-H)(Activated protein C-binding protein)(APC inhibitor)(Anticardiolipin cofactor) [Source: Ur
2554.1.1	-2.3	5.49	1104.5473	0.0016	3	27.81	kcfk	328	EHSSLAFWK	336	tdas		ENSP00000205948	Beta-2-glycoprotein 1 Precursor (Beta-2-glycoprotein I)(Beta(2)GPI)(B2GPI)(Apolipoprotein H)(Apo-H)(Activated protein C-binding protein)(APC inhibitor)(Anticardiolipin cofactor) [Source: Ur
2560.1.1	-4.2	6.51	2883.2898	-0.0027	3	33.69	vplk	39	TFYEPGEEITYSCKPGYVSR	58	ggmr	C [51] 500.199, C [51] 57.0215	ENSP00000205948	Beta-2-glycoprotein 1 Precursor (Beta-2-glycoprotein I)(Beta(2)GPI)(B2GPI)(Apolipoprotein H)(Apo-H)(Activated protein C-binding protein)(APC inhibitor)(Anticardiolipin cofactor) [Source: Ur
2561.1.1	-4	5.19	2883.2898	-0.006	4	33.69	vplk	39	TFYEPGEEITYSCKPGYVSR	58	ggmr	C [51] 500.199, C [51] 57.0215	ENSP00000205948	Beta-2-glycoprotein 1 Precursor (Beta-2-glycoprotein I)(Beta(2)GPI)(B2GPI)(Apolipoprotein H)(Apo-H)(Activated protein C-binding protein)(APC inhibitor)(Anticardiolipin cofactor) [Source: Ur
2563.1.1	-2	4.52	1870.008	0.988	2	32.58	kfir	250	QFVPIKVEQIEAGTPGR	266	lrvv	Q [258] 0.9848	ENSP00000373506	Thioredoxin reductase 1, cytoplasmic (TR)(EC 1.8.1.9)(Thioredoxin reductase TR1)(KM-102-derived reductase-like factor)(Gene associated with retinoid-IFN-induced mortality 12 protein)(GR
2567.1.1	-3.1	6.62	2883.2898	-0.0027	3	33.69	vplk	39	TFYEPGEEITYSCKPGYVSR	58	ggmr	C [51] 500.199, C [51] 57.0215	ENSP00000205948	Beta-2-glycoprotein 1 Precursor (Beta-2-glycoprotein I)(Beta(2)GPI)(B2GPI)(Apolipoprotein H)(Apo-H)(Activated protein C-binding protein)(APC inhibitor)(Anticardiolipin cofactor) [Source: Ur
2568.1.1	-1.4	5.32	2883.2898	-0.0067	4	33.69	vplk	39	TFYEPGEEITYSCKPGYVSR	58	ggmr	C [51] 500.199, C [51] 57.0215	ENSP00000205948	Beta-2-glycoprotein 1 Precursor (Beta-2-glycoprotein I)(Beta(2)GPI)(B2GPI)(Apolipoprotein H)(Apo-H)(Activated protein C-binding protein)(APC inhibitor)(Anticardiolipin cofactor) [Source: Ur
2571.1.1	-3.6	5.43	2883.29	-0.01	4	33.69	vplk	39	TFYEPGEEITYSCKPGYVSR	58	ggmr	C [51] 500.199, C [51] 57.0215	ENSP00000205948	Beta-2-glycoprotein 1 Precursor (Beta-2-glycoprotein I)(Beta(2)GPI)(B2GPI)(Apolipoprotein H)(Apo-H)(Activated protein C-binding protein)(APC inhibitor)(Anticardiolipin cofactor) [Source: Ur
2578.1.1	-3.3	4.71	3214.4027	-0.006	3	26.94	nkek	306	KCSYTEDAQCIDGTIEVPK	324	cfke	C [307] 500.199, C [315] 500.199, C [307] !	ENSP00000205948	Beta-2-glycoprotein 1 Precursor (Beta-2-glycoprotein I)(Beta(2)GPI)(B2GPI)(Apolipoprotein H)(Apo-H)(Activated protein C-binding protein)(APC inhibitor)(Anticardiolipin cofactor) [Source: Ur
2585.1.1	-3.5	4.93	3214.4027	-0.0034	3	26.94	nkek	306	KCSYTEDAQCIDGTIEVPK	324	cfke	C [307] 500.199, C [315] 500.199, C [307] !	ENSP00000205948	Beta-2-glycoprotein 1 Precursor (Beta-2-glycoprotein I)(Beta(2)GPI)(B2GPI)(Apolipoprotein H)(Apo-H)(Activated protein C-binding protein)(APC inhibitor)(Anticardiolipin cofactor) [Source: Ur
2591.1.1	-4.6	4.95	3214.4027	0.0002	3	26.94	nkek	306	KCSYTEDAQCIDGTIEVPK	324	cfke	C [307] 500.199, C [315] 500.199, C [307] !	ENSP00000205948	Beta-2-glycoprotein 1 Precursor (Beta-2-glycoprotein I)(Beta(2)GPI)(B2GPI)(Apolipoprotein H)(Apo-H)(Activated protein C-binding protein)(APC inhibitor)(Anticardiolipin cofactor) [Source: Ur
2602.1.1	-1.5	4.82	3474.6543	-0.0057	4	38.61	revk	205	CPFPSRPDNGFVNYPAKPTLYYKD	229	atfg	C [205] 500.199, C [205] 57.0215	ENSP00000205948	Beta-2-glycoprotein 1 Precursor (Beta-2-glycoprotein I)(Beta(2)GPI)(B2GPI)(Apolipoprotein H)(Apo-H)(Activated protein C-binding protein)(APC inhibitor)(Anticardiolipin cofactor) [Source: Ur
2608.1.1	-2.6	4.99	3474.6543	0.0023	4	38.61	revk	205	CPFPSRPDNGFVNYPAKPTLYYKD	229	atfg	C [205] 500.199, C [205] 57.0215	ENSP00000205948	Beta-2-glycoprotein 1 Precursor (Beta-2-glycoprotein I)(Beta(2)GPI)(B2GPI)(Apolipoprotein H)(Apo-H)(Activated protein C-binding protein)(APC inhibitor)(Anticardiolipin cofactor) [Source: Ur
2628.1.1	-11	4.41	2085.9103	0.0001	2	27.19	kekk	307	CSYTEDAQCIDGTIEVPK	324	cfke	C [307] 57.0215, C [315] 57.0215	ENSP00000205948	Beta-2-glycoprotein 1 Precursor (Beta-2-glycoprotein I)(Beta(2)GPI)(B2GPI)(Apolipoprotein H)(Apo-H)(Activated protein C-binding protein)(APC inhibitor)(Anticardiolipin cofactor) [Source: Ur
2632.1.1	-5.3	4.54	2085.9103	0.0018	2	27.19	kekk	307	CSYTEDAQCIDGTIEVPK	324	cfke	C [307] 57.0215, C [315] 57.0215	ENSP00000205948	Beta-2-glycoprotein 1 Precursor (Beta-2-glycoprotein I)(Beta(2)GPI)(B2GPI)(Apolipoprotein H)(Apo-H)(Activated protein C-binding protein)(APC inhibitor)(Anticardiolipin cofactor) [Source: Ur
2633.1.1	-2.9	4.6	3386.3936	-0.0026	3	29.48	ykdk	230	ATFGCHDGYSLDGPEEIECTK	250	lgnw	C [234] 500.199, C [248] 500.199, C [234] !	ENSP00000205948	Beta-2-glycoprotein 1 Precursor (Beta-2-glycoprotein I)(Beta(2)GPI)(B2GPI)(Apolipoprotein H)(Apo-H)(Activated protein C-binding protein)(APC inhibitor)(Anticardiolipin cofactor) [Source: Ur
2634.1.1	-2.3	4.58	2048.012	-0.019	3	35.93	mrak	154	QHVLLAQTYAADDSGMSLK	172	rtes		ENSP00000286332:reversed	
2638.1.1	-12.7	4.61	2085.9103	0.0023	2	27.19	kekk	307	CSYTEDAQCIDGTIEVPK	324	cfke	C [307] 57.0215, C [315] 57.0215	ENSP00000205948	Beta-2-glycoprotein 1 Precursor (Beta-2-glycoprotein I)(Beta(2)GPI)(B2GPI)(Apolipoprotein H)(Apo-H)(Activated protein C-binding protein)(APC inhibitor)(Anticardiolipin cofactor) [Source: Ur
2649.1.1	-5.5	4.63	3386.3936	-0.0056	3	29.48	ykdk	230	ATFGCHDGYSLDGPEEIECTK	250	lgnw	C [234] 500.199, C [248] 500.199, C [234] !	ENSP00000205948	Beta-2-glycoprotein 1 Precursor (Beta-2-glycoprotein I)(Beta(2)GPI)(B2GPI)(Apolipoprotein H)(Apo-H)(Activated protein C-binding protein)(APC inhibitor)(Anticardiolipin cofactor) [Source: Ur
2659.1.1	-1.1	5.02	2005.945	1.987	3	37.52	dwdr	101	MIEAVQNHIGSLNWGYR	117	valr	M [101] 15.9949, N [107] 0.9848, Q [106]	ENSP00000373506	Thioredoxin reductase 1, cytoplasmic (TR)(EC 1.8.1.9)(Thioredoxin reductase TR1)(KM-102-derived reductase-like factor)(Gene associated with retinoid-IFN-induced mortality 12 protein)(GR
2666.1.1	-1.9	4.82	2883.2898	-0.0003	3	33.69	vplk	39	TFYEPGEEITYSCKPGYVSR	58	ggmr	C [51] 500.199, C [51] 57.0215	ENSP00000205948	Beta-2-glycoprotein 1 Precursor (Beta-2-glycoprotein I)(Beta(2)GPI)(B2GPI)(Apolipoprotein H)(Apo-H)(Activated protein C-binding protein)(APC inhibitor)(Anticardiolipin cofactor) [Source: Ur
2672.1.1	-4.3	4.25	2005.945	1.99	2	37.52	dwdr	101	MIEAVQNHIGSLNWGYR	117	valr	M [101] 15.9949, N [107] 0.9848, Q [106]	ENSP00000373506	Thioredoxin reductase 1, cytoplasmic (TR)(EC 1.8.1.9)(Thioredoxin reductase TR1)(KM-102-derived reductase-like factor)(Gene associated with retinoid-IFN-induced mortality 12 protein)(GR
2676.1.1	-1.2	4.71	2883.2898	0.0026	3	33.69	vplk	39	TFYEPGEEITYSCKPGYVSR	58	ggmr	C [51] 500.199, C [51] 57.0215	ENSP00000205948	Beta-2-glycoprotein 1 Precursor (Beta-2-glycoprotein I)(Beta(2)GPI)(B2GPI)(Apolipoprotein H)(Apo-H)(Activated protein C-binding protein)(APC inhibitor)(Anticardiolipin cofactor) [Source: Ur
2686.1.1	-1.4	5.55	3587.7384	-0.0026	4	38.47	pecr	202	EVKCPFPSRPDNGFVNYPAKPTLY	227	dkat	C [205] 500.199, C [205] 57.0215	ENSP00000205948	Beta-2-glycoprotein 1 Precursor (Beta-2-glycoprotein I)(Beta(2)GPI)(B2GPI)(Apolipoprotein H)(Apo-H)(Activated protein C-binding protein)(APC inhibitor)(Anticardiolipin cofactor) [Source: Ur
2687.1.1	-4.3	4.61	2586.109	-0.0038	3	27.19	kekk	307	CSYTEDAQCIDGTIEVPK	324	cfke	C [307] 57.0215, C [315] 500.199, C [315] !	ENSP00000205948	Beta-2-glycoprotein 1 Precursor (Beta-2-glycoprotein I)(Beta(2)GPI)(B2GPI)(Apolipoprotein H)(Apo-H)(Activated protein C-binding protein)(APC inhibitor)(Anticardiolipin cofactor) [Source: Ur
2690.1.1	-2.8	4.71	2586.109	0.0013	3	27.19	kekk	307	CSYTEDAQCIDGTIEVPK	324	cfke	C [307] 57.0215, C [315] 500.199, C [315] !	ENSP00000205948	Beta-2-glycoprotein 1 Precursor (Beta-2-glycoprotein I)(Beta(2)GPI)(B2GPI)(Apolipoprotein H)(Apo-H)(Activated protein C-binding protein)(APC inhibitor)(Anticardiolipin cofactor) [Source: Ur
2692.1.1	-2.4	4.46	2039.94	-0.0048	3	33.42	evpk	325	CFKEHSSLAFWK	336	tdas	C [325] 500.199, C [325] 57.0215	ENSP00000205948	Beta-2-glycoprotein 1 Precursor (Beta-2-glycoprotein I)(Beta(2)GPI)(B2GPI)(Apolipoprotein H)(Apo-H)(Activated protein C-binding protein)(APC inhibitor)(Anticardiolipin cofactor) [Source: Ur
2695.1.1	-3.9	4.67	2586.109	0.0017	3	27.19	kekk	307	CSYTEDAQCIDGTIEVPK	324	cfke	C [307] 57.0215, C [315] 500.199, C [315] !	ENSP00000205948	Beta-2-glycoprotein 1 Precursor (Beta-2-glycoprotein I)(Beta(2)GPI)(B2GPI)(Apolipoprotein H)(Apo-H)(Activated protein C-binding protein)(APC inhibitor)(Anticardiolipin cofactor) [Source: Ur
2701.1.1	-2.7	4.97	2731.3337	-0.0096	4	38.86	revk	205	CPFPSRPDNGFVNYPAKPTLYYK	227	dkat	C [205] 57.0215	ENSP00000205948	Beta-2-glycoprotein 1 Precursor (Beta-2-glycoprotein I)(Beta(2)GPI)(B2GPI)(Apolipoprotein H)(Apo-H)(Activated protein C-binding protein)(APC inhibitor)(Anticardiolipin cofactor) [Source: Ur
2705.1.1	-3	5.3	2731.3337	-0.0015	4	38.86	revk	205	CPFPSRPDNGFVNYPAKPTLYYK	227	dkat	C [205] 57.0215	ENSP00000205948	Beta-2-glycoprotein 1 Precursor (Beta-2-glycoprotein I)(Beta(2)GPI)(B2GPI)(Apolipoprotein H)(Apo-H)(Activated protein C-binding protein)(APC inhibitor)(Anticardiolipin cofactor) [Source: Ur
2706.1.1	-4.5	4.6	2731.3337	0	3	38.86	revk	205	CPFPSRPDNGFVNYPAKPTLYYK	227	dkat	C [205] 57.0215	ENSP00000205948	Beta-2-glycoprotein 1 Precursor (Beta-2-glycoprotein I)(Beta(2)GPI)(B2GPI)(Apolipoprotein H)(Apo-H)(Activated protein C-binding protein)(APC inhibitor)(Anticardiolipin cofactor) [Source: Ur
2714.1.1	-6.6	4.87	2731.3337	0.0071	3	38.86	revk	205	CPFPSRPDNGFVNYPAKPTLYYK	227	dkat	C [205] 57.0215	ENSP00000205948	Beta-2-glycoprotein 1 Precursor (Beta-2-glycoprotein I)(Beta(2)GPI)(B2GPI)(Apolipoprotein H)(Apo-H)(Activated protein C-binding protein)(APC inhibitor)(Anticardiolipin cofactor) [Source: Ur
2718.1.1	-9.9	4.9	2731.3337	0.0046	3	38.86	revk	205	CPFPSRPDNGFVNYPAKPTLYYK	227	dkat	C [205] 57.0215	ENSP00000205948	Beta-2-glycoprotein 1 Precursor (Beta-2-glycoprotein I)(Beta(2)GPI)(B2GPI)(Apolipoprotein H)(Apo-H)(Activated protein C-binding protein)(APC inhibitor)(Anticardiolipin cofactor) [Source: Ur
2734.1.1	-2.4	5.02	2925.3004	-0.0001	3	33.69	vplk	39	TFYEPGEEITYSCKPGYVSR	58	ggmr	C [51] 500.199, C [51] 57.0215, T [39] 42.0	ENSP00000205948	Beta-2-glycoprotein 1 Precursor (Beta-2-glycoprotein I)(Beta(2)GPI)(B2GPI)(Apolipoprotein H)(Apo-H)(Activated protein C-binding protein)(APC inhibitor)(Anticardiolipin cofactor) [Source: Ur
2748.1.1	-1.9	3.98	1649.7774	-0.0014	2	32.41	lgtr	53	WGLGGTCVNVGCIPK	67	klmh	C [59] 57.0215, C [64] 57.0215, W [53] 31.	ENSP00000373506	Thioredoxin reductase 1, cytoplasmic (TR)(EC 1.8.1.9)(Thioredoxin reductase TR1)(KM-102-derived reductase-like factor)(Gene associated with retinoid-IFN-induced mortality 12 protein)(GR
2754.1.1	-1	4.03	1649.7774	0.0002	2	32.41	lgtr	53	WGLGGTCVNVGCIPK	67	klmh	C [59] 57.0215, C [64] 57.0215, W [53] 31.	ENSP00000373506	Thioredoxin reductase 1, cytoplasmic (TR)(EC 1.8.1.9)(Thioredoxin reductase TR1)(KM-102-derived reductase-like factor)(Gene associated with retinoid-IFN-induced mortality 12 protein)(GR
2758.1.1	-6	4.75	1243.6794	-0.0007	2	28.31	prvc	85	PFAGILENGAVR	96	yttf		ENSP00000205948	Beta-2-glycoprotein 1 Precursor (Beta-2-glycoprotein I)(Beta(2)GPI)(B2GPI)(Apolipoprotein H)(Apo-H)(Activated protein C-binding protein)(APC inhibitor)(Anticardiolipin cofactor) [Source: Ur
2759.1.1	-1.5	4.34	2883.2898	0.0041	3	33.69	vplk	39	TFYEPGEEITYSCKPGYVSR	58	ggmr	C [51] 500.199, C [51] 57.0215	ENSP00000205948	Beta-2-glycoprotein 1 Precursor (Beta-2-glycoprotein I)(Beta(2)GPI)(B2GPI)(Apolipoprotein H)(Apo-H)(Activated protein C-binding protein)(APC inhibitor)(Anticardiolipin cofactor) [Source: Ur
2761.1.1	-6.7	4.9	1243.6794	-0.001	2	28.31	prvc	85	PFAGILENGAVR	96	yttf		ENSP00000205948	Beta-2-glycoprotein 1 Precursor (Beta-2-glycoprotein I)(Beta(2)GPI)(B2GPI)(Apolipoprotein H)(Apo-H)(Activated protein C-binding protein)(APC inhibitor)(Anticardiolipin cofactor) [Source: Ur
2762.1.1	-1.1	4.32	2883.2898	0.0059	3	33.69	vplk	39	TFYEPGEEITYSCKPGYVSR	58	ggmr	C [51] 500.199, C [51] 57.0215	ENSP00000205948	Beta-2-glycoprotein 1 Precursor (Beta-2-glycoprotein I)(Beta(2)GPI)(B2GPI)(Apolipoprotein H)(Apo-H)(Activated protein C-binding protein)(APC inhibitor)(Anticardiolipin cofactor) [Source: Ur
2764.1.1	-7.1	5.11	1243.6794	-0.0013	2	28.31	prvc	85	PFAGILENGAVR	96	yttf		ENSP00000205948	Beta-2-glycoprotein 1 Precursor (Beta-2-glycoprotein I)(Beta(2)GPI)(B2GPI)(Apolipoprotein H)(Apo-H)(Activated protein C-binding protein)(APC inhibitor)(Anticardiolipin cofactor) [Source: Ur
2778.1.1	-3.7	4.47	3086.3077	-0.0021	3	27.19	kekk	307	CSYTEDAQCIDGTIEVPK	324	cfke	C [307] 500.199, C [315] 500.199, C [307] !	ENSP00000205948	Beta-2-glycoprotein 1 Precursor (Beta-2-glycoprotein I)(Beta(2)GPI)(B2GPI)(Apolipoprotein H)(Apo-H)(Activated protein C-binding protein)(APC inhibitor)(Anticardiolipin cofactor) [Source: Ur
2779.1.1	-1.6	4.48	3231.5324	-0.0062	3	38.86	revk	205	CPFPSRPDNGFVNYPAKPTLYYK	227	dkat	C [205] 500.199, C [205] 57.0215	ENSP00000205948	Beta-2-glycoprotein 1 Precursor (Beta-2-glycoprotein I)(Beta(2)GPI)(B2GPI)(Apolipoprotein H)(Apo-H)(Activated protein C-binding protein)(APC inhibitor)(Anticardiolipin cofactor) [Source: Ur
2782.1.1	-1.8	4.7	3231.532	0.012	3	38.86	revk	205	CPFPSRPDNGFVNYPAKPTLYYK	227	dkat	C [205] 500.199, C [205] 57.0215	ENSP00000205948	Beta-2-glycoprotein 1 Precursor (Beta-2-glycoprotein I)(Beta(2)GPI)(B2GPI)(Apolipoprotein H)(Apo-H)(Activated protein C-binding protein)(APC inhibitor)(Anticardiolipin cofactor) [Source: Ur
2787.1.1	-2.5	5.21	2732.3185	0	4	38.86	revk	205	CPFPSRPDNGFVNYPAKPTLYYK	227	dkat	C [205] 57.0215, N [213] 0.9848	ENSP00000205948	Beta-2-glycoprotein 1 Precursor (Beta-2-glycoprotein I)(Beta(2)GPI)(B2GPI)(Apolipoprotein H)(Apo-H)(Activated protein C-binding protein)(APC inhibitor)(Anticardiolipin cofactor) [Source: Ur
2788.1.1	-5.5	5.05	1435.6265	0.0001	2	28.46	vplk	39	TFYEPGEEITYS	50	ckpg		ENSP00000205948	Beta-2-glycoprotein 1 Precursor (Beta-2-glycoprotein I)(Beta(2)GPI)(B2GPI)(Apolipoprotein H)(Apo-H)(Activated protein C-binding protein)(APC inhibitor)(Anticardiolipin cofactor) [Source: Ur
2789.1.1	-4.4	4.64	3231.5324	0.0052	3	38.86	revk	205	CPFPSRPDNGFVNYPAKPTLYYK	227	dkat	C [205] 500.199, C [205] 57.0215	ENSP00000205948	Beta-2-glycoprotein 1 Precursor (Beta-2-glycoprotein I)(Beta(2)GPI)(B2GPI)(Apolipoprotein H)(Apo-H)(Activated protein C-binding protein)(APC inhibitor)(Anticardiolipin cofactor) [Source: Ur
2792.1.1	-2.8	4.26	3086.3077	0.0052	3	27.19	kekk	307	CSYTEDAQCIDGTIEVPK	324	cfke	C [307] 500.199, C [315] 500.199, C [307] !	ENSP00000205948	Beta-2-glycoprotein 1 Precursor (Beta-2-glycoprotein I)(Beta(2)GPI)(B2GPI)(Apolipoprotein H)(Apo-H)(Activated protein C-binding protein)(APC inhibitor)(Anticardiolipin cofactor) [Source: Ur
2794.1.1	-5.8	5.18	1435.6265	-0.0024	2	28.46	vplk	39	TFYEPGEEITYS	50	ckpg		ENSP00000205948	Beta-2-glycoprotein 1 Precursor (Beta-2-glycoprotein I)(Beta(2)GPI)(B2GPI)(Apolipoprotein H)(Apo-H)(Activated protein C-binding protein)(APC inhibitor)(Anticardiolipin cofactor) [Source: Ur
2802.1.1	-5.8	5.16	1435.6265	-0.0027	2	28.46	vplk	39	TFYEPGEEITYS	50	ckpg		ENSP00000205948	Beta-2-glycoprotein 1 Precursor (Beta-2-glycoprotein I)(Beta(2)GPI)(B2GPI)(Apolipoprotein H)(Apo-H)(Activated protein C-binding protein)(APC inhibitor)(Anticardiolipin cofactor) [Source: Ur
2841.1.1	-4.2	5.02	2957.429	-0.01	4	38.61	revk	205	CPFPSRPDNGFVNYPAKPTLYYKD	229	atfg	C [205] 57.0215, C [205] -17.0265	ENSP00000205948	Beta-2-glycoprotein 1 Precursor (Beta-2-glycoprotein I)(Beta(2)GPI)(B2GPI)(Apolipoprotein H)(Apo-H)(Activated protein C-binding protein)(APC inhibitor)(Anticardiolipin cofactor) [Source: Ur
2846.1.1	-3.9	5.21	2957.4291	-0.0071	4	38.61	revk	205	CPFPSRPDNGFVNYPAKPTLYYKD	229	atfg	C [205] 57.0215, C [205] -17.0265	ENSP00000205948	Beta-2-glycoprotein 1 Precursor (Beta-2-glycoprotein I)(Beta(2)GPI)(B2GPI)(Apolipoprotein H)(Apo-H)(Activated protein C-binding protein)(APC inhibitor)(Anticardiolipin cofactor) [Source: Ur
2847.1.1	-6.6	4.49	2957.429	0.017	3	38.61	revk	205	CPFPSRPDNGFVNYPAKPTLYYKD	229	atfg	C [205] 57.0215, C [205] -17.0265	ENSP00000205948	Beta-2-glycoprotein 1 Precursor (Beta-2-glycoprotein I)(Beta(2)GPI)(B2GPI)(Apolipoprotein H)(Apo-H)(Activated protein C-binding protein)(APC inhibitor)(Anticardiolipin cofactor) [Source: Ur
2850.1.1	-2.9	5.28	2957.4291	-0.0032	4	38.61	revk	205	CPFPSRPDNGFVNYPAKPTLYYKD	229	atfg	C [205] 57.0215, C [205] -17.0265	ENSP00000205948	Beta-2-glycoprotein 1 Precursor (Beta-2-glycoprotein I)(Beta(2)GPI)(B2GPI)(Apolipoprotein H)(Apo-H)(Activated protein C-binding protein)(APC inhibitor)(Anticardiolipin cofactor) [Source: Ur
2851.1.1	-4.4	4.53	2957.429	0.01	3	38.61	revk	205	CPFPSRPDNGFVNYPAKPTLYYKD	229	atfg	C [205] 57.0215, C [205] -17.0265	ENSP00000205948	Beta-2-glycoprotein 1 Precursor (Beta-2-glycoprotein I)(Beta(2)GPI)(B2GPI)(Apolipoprotein H)(Apo-H)(Activated protein C-binding protein)(APC inhibitor)(Anticardiolipin cofactor) [Source: Ur
2853.1.1	-4	4.5	2957.429	0.018	3	38.61	revk	205	CPFPSRPDNGFVNYPAKPTLYYKD	229	atfg	C [205] 57.0215, C [205] -17.0265	ENSP00000205948	Beta-2-glycoprotein 1 Precursor (Beta-2-glycoprotein I)(Beta(2)GPI)(B2GPI)(Apolipoprotein H)(Apo-H)(Activated protein C-binding protein)(APC inhibitor)(Anticardiolipin cofactor) [Source: Ur
2857.1.1	-3.4	4.08	1086.5367	-0.0008	2	27.81	kcfk	328	EHSSLAFWK	336	tdas	E [328] -18.0106	ENSP00000205948	Beta-2-glycoprotein 1 Precursor (Beta-2-glycoprotein I)(Beta(2)GPI)(B2GPI)(Apolipoprotein H)(Apo-H)(Activated protein C-binding protein)(APC inhibitor)(Anticardiolipin cofactor) [Source: Ur
2861.1.1	-4.8	4.22	2070.105	-0.0001	3	37.03	aiag	21	RTCPKPDDLPFSTVVPLK	38	tfye	C [23] 57.0215	ENSP00000205948	Beta-2-glycoprotein 1 Precursor (Beta-2-glycoprotein I)(Beta(2)GPI)(B2GPI)(Apolipoprotein H)(Apo-H)(Activated protein C-binding protein)(APC inhibitor)(Anticardiolipin cofactor) [Source: Ur
2865.1.1	-4.7	4.31	2070.105	-0.0019	3	37.03	aiag	21	RTCPKPDDLPFSTVVPLK	38	tfye	C [23] 57.0215	ENSP00000205948	Beta-2-glycoprotein 1 Precursor (Beta-2-glycoprotein I)(Beta(2)GPI)(B2GPI)(Apolipoprotein H)(Apo-H)(Activated protein C-binding protein)(APC inhibitor)(Anticardiolipin cofactor) [Source: Ur
2866.1.1	-4.4	4.02	1086.5367	0.0017	2	27.81	kcfk	328	EHSSLAFWK	336	tdas	E [328] -18.0106	ENSP00000205948	Beta-2-glycoprotein 1 Precursor (Beta-2-glycoprotein I)(Beta(2)GPI)(B2GPI)(Apolipoprotein H)(Apo-H)(Activated protein C-binding protein)(APC inhibitor)(Anticardiolipin cofactor) [Source: Ur
2869.1.1	-7.1	4.54	2070.105	-0.0019	3	37.03	aiag	21	RTCPKPDDLPFSTVVPLK	38	tfye	C [23] 57.0215	ENSP00000205948	Beta-2-glycoprotein 1 Precursor (Beta-2-glycoprotein I)(Beta(2)GPI)(B2GPI)(Apolipoprotein H)(Apo-H)(Activated protein C-binding protein)(APC inhibitor)(Anticardiolipin cofactor) [Source: Ur
2879.1.1	-10.4	5.56	2127.1265	0.0006	3	36.23	vaia	20	GRTCPKPDDLPFSTVVPLK	38	tfye	C [23] 57.0215	ENSP00000205948	Beta-2-glycoprotein 1 Precursor (Beta-2-glycoprotein I)(Beta(2)GPI)(B2GPI)(Apolipoprotein H)(Apo-H)(Activated protein C-binding protein)(APC inhibitor)(Anticardiolipin cofactor) [Source: Ur
2884.1.1	-7.1	5.5	2127.1265	-0.0007	3	36.23	vaia	20	GRTCPKPDDLPFSTVVPLK	38	tfye	C [23] 57.0215	ENSP00000205948	Beta-2-glycoprotein 1 Precursor (Beta-2-glycoprotein I)(Beta(2)GPI)(B2GPI)(Apolipoprotein H)(Apo-H)(Activated protein C-binding protein)(APC inhibitor)(Anticardiolipin cofactor) [Source: Ur
2885.1.1	-2.3	5.74	2127.1265	0.0016	4	36.23	vaia	20	GRTCPKPDDLPFSTVVPLK	38	tfye	C [23] 57.0215	ENSP00000205948	Beta-2-glycoprotein 1 Precursor (Beta-2-glycoprotein I)(Beta(2)GPI)(B2GPI)(Apolipoprotein H)(Apo-H)(Activated protein C-binding protein)(APC inhibitor)(Anticardiolipin cofactor) [Source: Ur
2887.1.1	-6.7	4.98	2211.104	0.0001	3	36.26	iqvr	58	LGEHNIDVLEGNEQFINAAK	77	iith		sp|TRYP_PIG|	Trypsin; EC 3.4.21.4; Flags: Precursor;
2890.1.1	-7.4	5.27	2127.1265	-0.0005	3	36.23	vaia	20	GRTCPKPDDLPFSTVVPLK	38	tfye	C [23] 57.0215	ENSP00000205948	Beta-2-glycoprotein 1 Precursor (Beta-2-glycoprotein I)(Beta(2)GPI)(B2GPI)(Apolipoprotein H)(Apo-H)(Activated protein C-binding protein)(APC inhibitor)(Anticardiolipin cofactor) [Source: Ur
2891.1.1	-2	5.55	2127.1265	0.0031	4	36.23	vaia	20	GRTCPKPDDLPFSTVVPLK	38	tfye	C [23] 57.0215	ENSP00000205948	Beta-2-glycoprotein 1 Precursor (Beta-2-glycoprotein I)(Beta(2)GPI)(B2GPI)(Apolipoprotein H)(Apo-H)(Activated protein C-binding protein)(APC inhibitor)(Anticardiolipin cofactor) [Source: Ur
2892.1.1	-5.5	5.12	2211.104	0.002	3	36.26	iqvr	58	LGEHNIDVLEGNEQFINAAK	77	iith		sp|TRYP_PIG|	Trypsin; EC 3.4.21.4; Flags: Precursor;
2897.1.1	-7.6	5.19	2211.104	-0.0032	3	36.26	iqvr	58	LGEHNIDVLEGNEQFINAAK	77	iith		sp|TRYP_PIG|	Trypsin; EC 3.4.21.4; Flags: Precursor;
2928.1.1	-1.6	4.63	2958.4139	-0.0051	4	38.61	revk	205	CPFPSRPDNGFVNYPAKPTLYYKD	229	atfg	C [205] 57.0215, N [217] 0.9848, C [205] -1	ENSP00000205948	Beta-2-glycoprotein 1 Precursor (Beta-2-glycoprotein I)(Beta(2)GPI)(B2GPI)(Apolipoprotein H)(Apo-H)(Activated protein C-binding protein)(APC inhibitor)(Anticardiolipin cofactor) [Source: Ur
2932.1.1	-2.4	4.6	2958.4139	-0.0048	4	38.61	revk	205	CPFPSRPDNGFVNYPAKPTLYYKD	229	atfg	C [205] 57.0215, N [217] 0.9848, C [205] -1	ENSP00000205948	Beta-2-glycoprotein 1 Precursor (Beta-2-glycoprotein I)(Beta(2)GPI)(B2GPI)(Apolipoprotein H)(Apo-H)(Activated protein C-binding protein)(APC inhibitor)(Anticardiolipin cofactor) [Source: Ur
2937.1.1	-6.5	4.42	2958.4139	0.0035	4	38.61	revk	205	CPFPSRPDNGFVNYPAKPTLYYKD	229	atfg	C [205] 57.0215, N [217] 0.9848, C [205] -1	ENSP00000205948	Beta-2-glycoprotein 1 Precursor (Beta-2-glycoprotein I)(Beta(2)GPI)(B2GPI)(Apolipoprotein H)(Apo-H)(Activated protein C-binding protein)(APC inhibitor)(Anticardiolipin cofactor) [Source: Ur
2957.1.1	-3.1	4.22	2169.137	0.0027	3	36.23	vaia	20	GRTCPKPDDLPFSTVVPLK	38	tfye	C [23] 57.0215, G [20] 42.0106	ENSP00000205948	Beta-2-glycoprotein 1 Precursor (Beta-2-glycoprotein I)(Beta(2)GPI)(B2GPI)(Apolipoprotein H)(Apo-H)(Activated protein C-binding protein)(APC inhibitor)(Anticardiolipin cofactor) [Source: Ur
2984.1.1	-2	5.87	2627.3252	-0.0069	4	36.23	vaia	20	GRTCPKPDDLPFSTVVPLK	38	tfye	C [23] 500.199, C [23] 57.0215	ENSP00000205948	Beta-2-glycoprotein 1 Precursor (Beta-2-glycoprotein I)(Beta(2)GPI)(B2GPI)(Apolipoprotein H)(Apo-H)(Activated protein C-binding protein)(APC inhibitor)(Anticardiolipin cofactor) [Source: Ur
2985.1.1	-2.6	4.49	1255.6753	0.0011	2	7.6	mgsr	43	QKAATEGPAQVR	54	aarp		ENSP00000321601	No description
2991.1.1	-2.4	4.77	2996.331	-0.0022	4	36.49	evpk	325	CFKEHSSLAFWKTDASDVKPC	345	]	C [325] 57.0215, C [345] 500.199, C [345] !	ENSP00000205948	Beta-2-glycoprotein 1 Precursor (Beta-2-glycoprotein I)(Beta(2)GPI)(B2GPI)(Apolipoprotein H)(Apo-H)(Activated protein C-binding protein)(APC inhibitor)(Anticardiolipin cofactor) [Source: Ur
2992.1.1	-1.7	4.47	1255.6753	0.0004	2	7.6	mgsr	43	QKAATEGPAQVR	54	aarp		ENSP00000321601	No description
2994.1.1	-2	3.94	979.5611	-0.0012	2	27.58	ryqk	189	FALPQYLK	196	tvyq		sp|CAS2_BOVIN|	
2997.1.1	-3.3	4.8	2996.331	-0.0091	4	36.49	evpk	325	CFKEHSSLAFWKTDASDVKPC	345	]	C [325] 57.0215, C [345] 500.199, C [345] !	ENSP00000205948	Beta-2-glycoprotein 1 Precursor (Beta-2-glycoprotein I)(Beta(2)GPI)(B2GPI)(Apolipoprotein H)(Apo-H)(Activated protein C-binding protein)(APC inhibitor)(Anticardiolipin cofactor) [Source: Ur
2998.1.1	-13.5	4.47	2068.8838	-0.0083	2	27.19	kekk	307	CSYTEDAQCIDGTIEVPK	324	cfke	C [307] 57.0215, C [315] 57.0215, C [307] ·	ENSP00000205948	Beta-2-glycoprotein 1 Precursor (Beta-2-glycoprotein I)(Beta(2)GPI)(B2GPI)(Apolipoprotein H)(Apo-H)(Activated protein C-binding protein)(APC inhibitor)(Anticardiolipin cofactor) [Source: Ur
3006.1.1	-7.9	4.71	1617.7876	0.0032	2	32.41	lgtr	53	WGLGGTCVNVGCIPK	67	klmh	C [59] 57.0215, C [64] 57.0215	ENSP00000373506	Thioredoxin reductase 1, cytoplasmic (TR)(EC 1.8.1.9)(Thioredoxin reductase TR1)(KM-102-derived reductase-like factor)(Gene associated with retinoid-IFN-induced mortality 12 protein)(GR
3009.1.1	-7.8	4.77	1617.7876	0.0005	2	32.41	lgtr	53	WGLGGTCVNVGCIPK	67	klmh	C [59] 57.0215, C [64] 57.0215	ENSP00000373506	Thioredoxin reductase 1, cytoplasmic (TR)(EC 1.8.1.9)(Thioredoxin reductase TR1)(KM-102-derived reductase-like factor)(Gene associated with retinoid-IFN-induced mortality 12 protein)(GR

3010.1.1	-12.8	4.55	2068.8838	0.0005	2	27.19	kekk	307	CSYTEDAQCIDGTIEVPK	324	cfke	C [307] 57.0215, C [315] 57.0215, C [307]	ENSP00000205948	Beta-2-glycoprotein 1 Precursor (Beta-2-glycoprotein I)(Beta(2)GPI)(B2GPI)(Apolipoprotein H)(Apo-H)(Activated protein C-binding protein)(APC inhibitor)(Anticardiolipin cofactor) [Source: Ur
3016.1.1	-7.3	5.02	1617.7876	-0.0006	2	32.41	lgtr	53	WGLGGTCVNVGCIPK	67	klmh	C [59] 57.0215, C [64] 57.0215	ENSP00000373506	Thioredoxin reductase 1, cytoplasmic (TR)(EC 1.8.1.9)(Thioredoxin reductase TR1)(KM-102-derived reductase-like factor)(Gene associated with retinoid-IFN-induced mortality 12 protein)(GR
3029.1.1	-1.2	3.92	1997.91	0.016	2	9.99	riek	351	VEGNSLQPPPGCSR	364	vqgk	C [362] 500.199, C [362] 57.0215	ENSP00000356384:reversed	
3032.1.1	-5.9	4.01	2569.0825	0.0003	2	27.19	kekk	307	CSYTEDAQCIDGTIEVPK	324	cfke	C [307] 57.0215, C [315] 500.199, C [315]	ENSP00000205948	Beta-2-glycoprotein 1 Precursor (Beta-2-glycoprotein I)(Beta(2)GPI)(B2GPI)(Apolipoprotein H)(Apo-H)(Activated protein C-binding protein)(APC inhibitor)(Anticardiolipin cofactor) [Source: Ur
3034.1.1	-1.9	4.35	2237.106	0.011	3	38.12	srmk	110	CIPYSVLLKDLEMR	123	nlre	C [110] 500.199, C [110] 57.0215	ENSP00000386567	
3037.1.1	-4.2	4.68	2714.3072	0.0063	3	38.86	revk	205	CPFPSRPDNGFVNYPAKPTLYYK	227	dkat	C [205] 57.0215, C [205] -17.0265	ENSP00000205948	Beta-2-glycoprotein 1 Precursor (Beta-2-glycoprotein I)(Beta(2)GPI)(B2GPI)(Apolipoprotein H)(Apo-H)(Activated protein C-binding protein)(APC inhibitor)(Anticardiolipin cofactor) [Source: Ur
3038.1.1	-1.1	4.45	2237.106	0.016	3	38.12	srmk	110	CIPYSVLLKDLEMR	123	nlre	C [110] 500.199, C [110] 57.0215	ENSP00000386567	
3043.1.1	-11	5.15	2714.3072	0.004	3	38.86	revk	205	CPFPSRPDNGFVNYPAKPTLYYK	227	dkat	C [205] 57.0215, C [205] -17.0265	ENSP00000205948	Beta-2-glycoprotein 1 Precursor (Beta-2-glycoprotein I)(Beta(2)GPI)(B2GPI)(Apolipoprotein H)(Apo-H)(Activated protein C-binding protein)(APC inhibitor)(Anticardiolipin cofactor) [Source: Ur
3050.1.1	-8.6	5.39	2714.3072	0.003	3	38.86	revk	205	CPFPSRPDNGFVNYPAKPTLYYK	227	dkat	C [205] 57.0215, C [205] -17.0265	ENSP00000205948	Beta-2-glycoprotein 1 Precursor (Beta-2-glycoprotein I)(Beta(2)GPI)(B2GPI)(Apolipoprotein H)(Apo-H)(Activated protein C-binding protein)(APC inhibitor)(Anticardiolipin cofactor) [Source: Ur
3068.1.1	-5	5.01	1914.0041	0.0021	3	36.7	iagr	22	TCPKPDDLPFSTVVPLK	38	tfye	C [23] 57.0215	ENSP00000205948	Beta-2-glycoprotein 1 Precursor (Beta-2-glycoprotein I)(Beta(2)GPI)(B2GPI)(Apolipoprotein H)(Apo-H)(Activated protein C-binding protein)(APC inhibitor)(Anticardiolipin cofactor) [Source: Ur
3069.1.1	-2.7	4.4	1100.6714	0.0009	2	32.27	kpdd	29	LPFSTVVPLK	38	tfye		ENSP00000205948	Beta-2-glycoprotein 1 Precursor (Beta-2-glycoprotein I)(Beta(2)GPI)(B2GPI)(Apolipoprotein H)(Apo-H)(Activated protein C-binding protein)(APC inhibitor)(Anticardiolipin cofactor) [Source: Ur
3074.1.1	-6.2	5.37	1914.0041	-0.0001	3	36.7	iagr	22	TCPKPDDLPFSTVVPLK	38	tfye	C [23] 57.0215	ENSP00000205948	Beta-2-glycoprotein 1 Precursor (Beta-2-glycoprotein I)(Beta(2)GPI)(B2GPI)(Apolipoprotein H)(Apo-H)(Activated protein C-binding protein)(APC inhibitor)(Anticardiolipin cofactor) [Source: Ur
3078.1.1	-5.9	5.53	1914.0041	-0.0008	3	36.7	iagr	22	TCPKPDDLPFSTVVPLK	38	tfye	C [23] 57.0215	ENSP00000205948	Beta-2-glycoprotein 1 Precursor (Beta-2-glycoprotein I)(Beta(2)GPI)(B2GPI)(Apolipoprotein H)(Apo-H)(Activated protein C-binding protein)(APC inhibitor)(Anticardiolipin cofactor) [Source: Ur
3082.1.1	-5.6	4.9	1652.9257	0.0018	3	35.49	grtc	24	PKPDDLPFSTVVPLK	38	tfye		ENSP00000205948	Beta-2-glycoprotein 1 Precursor (Beta-2-glycoprotein I)(Beta(2)GPI)(B2GPI)(Apolipoprotein H)(Apo-H)(Activated protein C-binding protein)(APC inhibitor)(Anticardiolipin cofactor) [Source: Ur
3085.1.1	-3.7	5	1853.966	0.015	2	32.58	kfir	250	QFVPIKVEQIEAGTPGR	266	lrvv	Q [250] 0.9848, Q [258] 0.9848, Q [250] -1	ENSP00000373506	Thioredoxin reductase 1, cytoplasmic (TR)(EC 1.8.1.9)(Thioredoxin reductase TR1)(KM-102-derived reductase-like factor)(Gene associated with retinoid-IFN-induced mortality 12 protein)(GR
3088.1.1	-7.2	4.59	1914.0041	0.0008	2	36.7	iagr	22	TCPKPDDLPFSTVVPLK	38	tfye	C [23] 57.0215	ENSP00000205948	Beta-2-glycoprotein 1 Precursor (Beta-2-glycoprotein I)(Beta(2)GPI)(B2GPI)(Apolipoprotein H)(Apo-H)(Activated protein C-binding protein)(APC inhibitor)(Anticardiolipin cofactor) [Source: Ur
3092.1.1	-2.2	5.51	1853.966	0.01	2	32.58	kfir	250	QFVPIKVEQIEAGTPGR	266	lrvv	Q [250] 0.9848, Q [258] 0.9848, Q [250] -1	ENSP00000373506	Thioredoxin reductase 1, cytoplasmic (TR)(EC 1.8.1.9)(Thioredoxin reductase TR1)(KM-102-derived reductase-like factor)(Gene associated with retinoid-IFN-induced mortality 12 protein)(GR
3093.1.1	-1.7	5.19	1853.966	0.01	3	32.58	kfir	250	QFVPIKVEQIEAGTPGR	266	lrvv	Q [250] 0.9848, Q [258] 0.9848, Q [250] -1	ENSP00000373506	Thioredoxin reductase 1, cytoplasmic (TR)(EC 1.8.1.9)(Thioredoxin reductase TR1)(KM-102-derived reductase-like factor)(Gene associated with retinoid-IFN-induced mortality 12 protein)(GR
3094.1.1	-4.8	4.55	1914.0041	0.0005	2	36.7	iagr	22	TCPKPDDLPFSTVVPLK	38	tfye	C [23] 57.0215	ENSP00000205948	Beta-2-glycoprotein 1 Precursor (Beta-2-glycoprotein I)(Beta(2)GPI)(B2GPI)(Apolipoprotein H)(Apo-H)(Activated protein C-binding protein)(APC inhibitor)(Anticardiolipin cofactor) [Source: Ur
3097.1.1	-3.1	4.58	1258.6889	0.0023	2	29.92	gank	95	EKLEATINELV	105	]		ENSP00000363641	Thioredoxin (Trx)(ATL-derived factor)(ADF)(Surface-associated sulphydryl protein)(SASP) [Source: UniProt P10599]
3099.1.1	-2.1	5.74	1853.9659	0.0097	2	32.58	kfir	250	QFVPIKVEQIEAGTPGR	266	lrvv	Q [250] 0.9848, Q [258] 0.9848, Q [250] -1	ENSP00000373506	Thioredoxin reductase 1, cytoplasmic (TR)(EC 1.8.1.9)(Thioredoxin reductase TR1)(KM-102-derived reductase-like factor)(Gene associated with retinoid-IFN-induced mortality 12 protein)(GR
3100.1.1	-2.9	5.35	1522.7147	0.0007	3	33.42	evpk	325	CFKEHSSLAFWK	336	tdas	C [325] 57.0215, C [325] -17.0265	ENSP00000205948	Beta-2-glycoprotein 1 Precursor (Beta-2-glycoprotein I)(Beta(2)GPI)(B2GPI)(Apolipoprotein H)(Apo-H)(Activated protein C-binding protein)(APC inhibitor)(Anticardiolipin cofactor) [Source: Ur
3101.1.1	-5.1	4.55	1258.6889	0.0003	2	29.92	gank	95	EKLEATINELV	105	]		ENSP00000363641	Thioredoxin (Trx)(ATL-derived factor)(ADF)(Surface-associated sulphydryl protein)(SASP) [Source: UniProt P10599]
3105.1.1	-1.6	5.66	1522.7147	0.0028	3	33.42	evpk	325	CFKEHSSLAFWK	336	tdas	C [325] 57.0215, C [325] -17.0265	ENSP00000205948	Beta-2-glycoprotein 1 Precursor (Beta-2-glycoprotein I)(Beta(2)GPI)(B2GPI)(Apolipoprotein H)(Apo-H)(Activated protein C-binding protein)(APC inhibitor)(Anticardiolipin cofactor) [Source: Ur
3107.1.1	-6.7	4.75	1522.7147	0.0001	2	33.42	evpk	325	CFKEHSSLAFWK	336	tdas	C [325] 57.0215, C [325] -17.0265	ENSP00000205948	Beta-2-glycoprotein 1 Precursor (Beta-2-glycoprotein I)(Beta(2)GPI)(B2GPI)(Apolipoprotein H)(Apo-H)(Activated protein C-binding protein)(APC inhibitor)(Anticardiolipin cofactor) [Source: Ur
3109.1.1	-4.3	4.53	1296.7311	0.0026	2	28.12	piic	143	PPPSIPTFATLR	154	vykp		ENSP00000205948	Beta-2-glycoprotein 1 Precursor (Beta-2-glycoprotein I)(Beta(2)GPI)(B2GPI)(Apolipoprotein H)(Apo-H)(Activated protein C-binding protein)(APC inhibitor)(Anticardiolipin cofactor) [Source: Ur
3111.1.1	-2.8	5.71	1522.7147	0.0021	3	33.42	evpk	325	CFKEHSSLAFWK	336	tdas	C [325] 57.0215, C [325] -17.0265	ENSP00000205948	Beta-2-glycoprotein 1 Precursor (Beta-2-glycoprotein I)(Beta(2)GPI)(B2GPI)(Apolipoprotein H)(Apo-H)(Activated protein C-binding protein)(APC inhibitor)(Anticardiolipin cofactor) [Source: Ur
3113.1.1	-9.4	4.75	1522.7147	-0.0006	2	33.42	evpk	325	CFKEHSSLAFWK	336	tdas	C [325] 57.0215, C [325] -17.0265	ENSP00000205948	Beta-2-glycoprotein 1 Precursor (Beta-2-glycoprotein I)(Beta(2)GPI)(B2GPI)(Apolipoprotein H)(Apo-H)(Activated protein C-binding protein)(APC inhibitor)(Anticardiolipin cofactor) [Source: Ur
3119.1.1	-8	4.72	1522.7147	-0.0001	2	33.42	evpk	325	CFKEHSSLAFWK	336	tdas	C [325] 57.0215, C [325] -17.0265	ENSP00000205948	Beta-2-glycoprotein 1 Precursor (Beta-2-glycoprotein I)(Beta(2)GPI)(B2GPI)(Apolipoprotein H)(Apo-H)(Activated protein C-binding protein)(APC inhibitor)(Anticardiolipin cofactor) [Source: Ur
3120.1.1	-2.7	5.25	1789.9881	0.0001	3	38.62	qygk	37	KVMVLDFVTPTPLGTR	52	wglg	M [39] 15.9949	ENSP00000373506	Thioredoxin reductase 1, cytoplasmic (TR)(EC 1.8.1.9)(Thioredoxin reductase TR1)(KM-102-derived reductase-like factor)(Gene associated with retinoid-IFN-induced mortality 12 protein)(GR
3123.1.1	-3.4	4.21	1296.7311	0.0018	2	28.12	piic	143	PPPSIPTFATLR	154	vykp		ENSP00000205948	Beta-2-glycoprotein 1 Precursor (Beta-2-glycoprotein I)(Beta(2)GPI)(B2GPI)(Apolipoprotein H)(Apo-H)(Activated protein C-binding protein)(APC inhibitor)(Anticardiolipin cofactor) [Source: Ur
3126.1.1	-2.2	5.28	1789.9881	-0.0007	3	38.62	qygk	37	KVMVLDFVTPTPLGTR	52	wglg	M [39] 15.9949	ENSP00000373506	Thioredoxin reductase 1, cytoplasmic (TR)(EC 1.8.1.9)(Thioredoxin reductase TR1)(KM-102-derived reductase-like factor)(Gene associated with retinoid-IFN-induced mortality 12 protein)(GR
3127.1.1	-2.3	4.82	2517.2093	-0.0035	3	34.16	ntlk	79	CTPRVCPFAGILENGAVR	96	yttf	C [79] 57.0215, C [84] 500.199, C [84] 57.0	ENSP00000205948	Beta-2-glycoprotein 1 Precursor (Beta-2-glycoprotein I)(Beta(2)GPI)(B2GPI)(Apolipoprotein H)(Apo-H)(Activated protein C-binding protein)(APC inhibitor)(Anticardiolipin cofactor) [Source: Ur
3130.1.1	-5.4	4.46	1789.9881	0.0034	2	38.62	qygk	37	KVMVLDFVTPTPLGTR	52	wglg	M [39] 15.9949	ENSP00000373506	Thioredoxin reductase 1, cytoplasmic (TR)(EC 1.8.1.9)(Thioredoxin reductase TR1)(KM-102-derived reductase-like factor)(Gene associated with retinoid-IFN-induced mortality 12 protein)(GR
3132.1.1	-2.4	5.09	1789.9881	0.001	3	38.62	qygk	37	KVMVLDFVTPTPLGTR	52	wglg	M [39] 15.9949	ENSP00000373506	Thioredoxin reductase 1, cytoplasmic (TR)(EC 1.8.1.9)(Thioredoxin reductase TR1)(KM-102-derived reductase-like factor)(Gene associated with retinoid-IFN-induced mortality 12 protein)(GR
3133.1.1	-1.9	4.71	2517.2093	-0.001	3	34.16	ntlk	79	CTPRVCPFAGILENGAVR	96	yttf	C [79] 57.0215, C [84] 500.199, C [84] 57.0	ENSP00000205948	Beta-2-glycoprotein 1 Precursor (Beta-2-glycoprotein I)(Beta(2)GPI)(B2GPI)(Apolipoprotein H)(Apo-H)(Activated protein C-binding protein)(APC inhibitor)(Anticardiolipin cofactor) [Source: Ur
3135.1.1	-4.5	4.34	1789.9881	0.0045	2	38.62	qygk	37	KVMVLDFVTPTPLGTR	52	wglg	M [39] 15.9949	ENSP00000373506	Thioredoxin reductase 1, cytoplasmic (TR)(EC 1.8.1.9)(Thioredoxin reductase TR1)(KM-102-derived reductase-like factor)(Gene associated with retinoid-IFN-induced mortality 12 protein)(GR
3139.1.1	-8.9	4.67	2715.292	0.0041	3	38.86	revk	205	CPFPSRPDNGFVNYPAKPTLYYK	227	dkat	C [205] 57.0215, N [213] 0.9848, C [205] -1	ENSP00000205948	Beta-2-glycoprotein 1 Precursor (Beta-2-glycoprotein I)(Beta(2)GPI)(B2GPI)(Apolipoprotein H)(Apo-H)(Activated protein C-binding protein)(APC inhibitor)(Anticardiolipin cofactor) [Source: Ur
3146.1.1	-7.8	4.73	2715.292	0.0074	3	38.86	revk	205	CPFPSRPDNGFVNYPAKPTLYYK	227	dkat	C [205] 57.0215, N [213] 0.9848, C [205] -1	ENSP00000205948	Beta-2-glycoprotein 1 Precursor (Beta-2-glycoprotein I)(Beta(2)GPI)(B2GPI)(Apolipoprotein H)(Apo-H)(Activated protein C-binding protein)(APC inhibitor)(Anticardiolipin cofactor) [Source: Ur
3154.1.1	-6.2	4.54	2715.292	0.01	3	38.86	revk	205	CPFPSRPDNGFVNYPAKPTLYYK	227	dkat	C [205] 57.0215, N [213] 0.9848, C [205] -1	ENSP00000205948	Beta-2-glycoprotein 1 Precursor (Beta-2-glycoprotein I)(Beta(2)GPI)(B2GPI)(Apolipoprotein H)(Apo-H)(Activated protein C-binding protein)(APC inhibitor)(Anticardiolipin cofactor) [Source: Ur
3155.1.1	-4.1	4.64	2299.1751	-0.004	3	43.11	naak	78	IITHPNFNGNTLDNDIMLIK	97	lssp	M [94] 15.9949	sp|TRYP_PIG|	Trypsin; EC 3.4.21.4; Flags: Precursor;
3157.1.1	-6.1	4.72	2414.2028	-0.001	3	36.7	iagr	22	TCPKPDDLPFSTVVPLK	38	tfye	C [23] 500.199, C [23] 57.0215	ENSP00000205948	Beta-2-glycoprotein 1 Precursor (Beta-2-glycoprotein I)(Beta(2)GPI)(B2GPI)(Apolipoprotein H)(Apo-H)(Activated protein C-binding protein)(APC inhibitor)(Anticardiolipin cofactor) [Source: Ur
3160.1.1	-5.1	4.85	2414.2028	-0.0014	3	36.7	iagr	22	TCPKPDDLPFSTVVPLK	38	tfye	C [23] 500.199, C [23] 57.0215	ENSP00000205948	Beta-2-glycoprotein 1 Precursor (Beta-2-glycoprotein I)(Beta(2)GPI)(B2GPI)(Apolipoprotein H)(Apo-H)(Activated protein C-binding protein)(APC inhibitor)(Anticardiolipin cofactor) [Source: Ur
3163.1.1	-3.3	4.6	2299.1751	-0.0027	3	43.11	naak	78	IITHPNFNGNTLDNDIMLIK	97	lssp	M [94] 15.9949	sp|TRYP_PIG|	Trypsin; EC 3.4.21.4; Flags: Precursor;
3170.1.1	-2.2	4.33	2299.1751	0.0004	3	43.11	naak	78	IITHPNFNGNTLDNDIMLIK	97	lssp	M [94] 15.9949	sp|TRYP_PIG|	Trypsin; EC 3.4.21.4; Flags: Precursor;
3173.1.1	-4.7	5.02	2414.2028	-0.0047	3	36.7	iagr	22	TCPKPDDLPFSTVVPLK	38	tfye	C [23] 500.199, C [23] 57.0215	ENSP00000205948	Beta-2-glycoprotein 1 Precursor (Beta-2-glycoprotein I)(Beta(2)GPI)(B2GPI)(Apolipoprotein H)(Apo-H)(Activated protein C-binding protein)(APC inhibitor)(Anticardiolipin cofactor) [Source: Ur
3229.1.1	-8.5	4.36	1503.7632	-0.0002	2	32.15	ctpr	83	VCPFAGILENGAVR	96	yttf	C [84] 57.0215, N [92] 0.9848	ENSP00000205948	Beta-2-glycoprotein 1 Precursor (Beta-2-glycoprotein I)(Beta(2)GPI)(B2GPI)(Apolipoprotein H)(Apo-H)(Activated protein C-binding protein)(APC inhibitor)(Anticardiolipin cofactor) [Source: Ur
3233.1.1	-6.7	4.74	1503.7632	-0.0018	2	32.15	ctpr	83	VCPFAGILENGAVR	96	yttf	C [84] 57.0215, N [92] 0.9848	ENSP00000205948	Beta-2-glycoprotein 1 Precursor (Beta-2-glycoprotein I)(Beta(2)GPI)(B2GPI)(Apolipoprotein H)(Apo-H)(Activated protein C-binding protein)(APC inhibitor)(Anticardiolipin cofactor) [Source: Ur
3235.1.1	-2.1	4.59	2003.971	-0.016	3	33.52	adas	173	QSDYSKYYPLIEDLRN	188	kiis		ENSP00000251643	Keratin, type I cytoskeletal 12 (Cytokeratin 12) (K12) (CK 12). [Source: SWISSPROT (Q99456)]
3238.1.1	-9.9	5.1	1503.7632	-0.0007	2	32.15	ctpr	83	VCPFAGILENGAVR	96	yttf	C [84] 57.0215, N [92] 0.9848	ENSP00000205948	Beta-2-glycoprotein 1 Precursor (Beta-2-glycoprotein I)(Beta(2)GPI)(B2GPI)(Apolipoprotein H)(Apo-H)(Activated protein C-binding protein)(APC inhibitor)(Anticardiolipin cofactor) [Source: Ur
3248.1.1	-2.4	4.3	2300.1599	-0.0006	3	43.11	naak	78	IITHPNFNGNTLDNDIMLIK	97	lssp	M [94] 15.9949, N [83] 0.9848	sp|TRYP_PIG|	Trypsin; EC 3.4.21.4; Flags: Precursor;
3251.1.1	-1.8	4.35	2300.1599	0.0048	3	43.11	naak	78	IITHPNFNGNTLDNDIMLIK	97	lssp	M [94] 15.9949, N [85] 0.9848	sp|TRYP_PIG|	Trypsin; EC 3.4.21.4; Flags: Precursor;
3252.1.1	-2.2	5.24	1507.678	2.02	2	12.74	vtvk	197	CTPCYLHGKESR	208	gegs	C [197] 57.0215, C [200] 57.0215	ENSP00000377451:reversed	
3255.1.1	-3.4	4.5	2414.2028	-0.0021	3	36.7	iagr	22	TCPKPDDLPFSTVVPLK	38	tfye	C [23] 500.199, C [23] 57.0215	ENSP00000205948	Beta-2-glycoprotein 1 Precursor (Beta-2-glycoprotein I)(Beta(2)GPI)(B2GPI)(Apolipoprotein H)(Apo-H)(Activated protein C-binding protein)(APC inhibitor)(Anticardiolipin cofactor) [Source: Ur
3263.1.1	-3.8	4.24	2414.2028	-0.0014	3	36.7	iagr	22	TCPKPDDLPFSTVVPLK	38	tfye	C [23] 500.199, C [23] 57.0215	ENSP00000205948	Beta-2-glycoprotein 1 Precursor (Beta-2-glycoprotein I)(Beta(2)GPI)(B2GPI)(Apolipoprotein H)(Apo-H)(Activated protein C-binding protein)(APC inhibitor)(Anticardiolipin cofactor) [Source: Ur
3271.1.1	-3.2	4.04	2414.2028	-0.0017	3	36.7	iagr	22	TCPKPDDLPFSTVVPLK	38	tfye	C [23] 500.199, C [23] 57.0215	ENSP00000205948	Beta-2-glycoprotein 1 Precursor (Beta-2-glycoprotein I)(Beta(2)GPI)(B2GPI)(Apolipoprotein H)(Apo-H)(Activated protein C-binding protein)(APC inhibitor)(Anticardiolipin cofactor) [Source: Ur
3273.1.1	-4.7	5.18	1503.7632	0.0018	3	32.15	ctpr	83	VCPFAGILENGAVR	96	yttf	C [84] 57.0215, N [92] 0.9848	ENSP00000205948	Beta-2-glycoprotein 1 Precursor (Beta-2-glycoprotein I)(Beta(2)GPI)(B2GPI)(Apolipoprotein H)(Apo-H)(Activated protein C-binding protein)(APC inhibitor)(Anticardiolipin cofactor) [Source: Ur
3291.1.1	-7	5.5	1502.7784	0.001	3	32.15	ctpr	83	VCPFAGILENGAVR	96	yttf	C [84] 57.0215	ENSP00000205948	Beta-2-glycoprotein 1 Precursor (Beta-2-glycoprotein I)(Beta(2)GPI)(B2GPI)(Apolipoprotein H)(Apo-H)(Activated protein C-binding protein)(APC inhibitor)(Anticardiolipin cofactor) [Source: Ur
3297.1.1	-5.5	5.69	1502.7784	0.0007	3	32.15	ctpr	83	VCPFAGILENGAVR	96	yttf	C [84] 57.0215	ENSP00000205948	Beta-2-glycoprotein 1 Precursor (Beta-2-glycoprotein I)(Beta(2)GPI)(B2GPI)(Apolipoprotein H)(Apo-H)(Activated protein C-binding protein)(APC inhibitor)(Anticardiolipin cofactor) [Source: Ur
3308.1.1	-1.6	4.36	1984.946	0.012	3	26.95	ftsk	611	SDVQSGPSLGADPLSSADGPK	631	kkqv		ENSP00000380444:reversed	
3318.1.1	-9.2	6.03	1502.7784	-0.0005	2	32.15	ctpr	83	VCPFAGILENGAVR	96	yttf	C [84] 57.0215	ENSP00000205948	Beta-2-glycoprotein 1 Precursor (Beta-2-glycoprotein I)(Beta(2)GPI)(B2GPI)(Apolipoprotein H)(Apo-H)(Activated protein C-binding protein)(APC inhibitor)(Anticardiolipin cofactor) [Source: Ur
3319.1.1	-4.4	5.89	2002.9771	-0.0051	2	32.15	ctpr	83	VCPFAGILENGAVR	96	yttf	C [84] 500.199, C [84] 57.0215	ENSP00000205948	Beta-2-glycoprotein 1 Precursor (Beta-2-glycoprotein I)(Beta(2)GPI)(B2GPI)(Apolipoprotein H)(Apo-H)(Activated protein C-binding protein)(APC inhibitor)(Anticardiolipin cofactor) [Source: Ur
3325.1.1	-9.7	5.92	1502.7784	0.0002	2	32.15	ctpr	83	VCPFAGILENGAVR	96	yttf	C [84] 57.0215	ENSP00000205948	Beta-2-glycoprotein 1 Precursor (Beta-2-glycoprotein I)(Beta(2)GPI)(B2GPI)(Apolipoprotein H)(Apo-H)(Activated protein C-binding protein)(APC inhibitor)(Anticardiolipin cofactor) [Source: Ur
3326.1.1	-4.1	5.94	2002.9771	-0.0018	2	32.15	ctpr	83	VCPFAGILENGAVR	96	yttf	C [84] 500.199, C [84] 57.0215	ENSP00000205948	Beta-2-glycoprotein 1 Precursor (Beta-2-glycoprotein I)(Beta(2)GPI)(B2GPI)(Apolipoprotein H)(Apo-H)(Activated protein C-binding protein)(APC inhibitor)(Anticardiolipin cofactor) [Source: Ur
3327.1.1	-4.5	6.03	2002.9771	-0.0042	3	32.15	ctpr	83	VCPFAGILENGAVR	96	yttf	C [84] 500.199, C [84] 57.0215	ENSP00000205948	Beta-2-glycoprotein 1 Precursor (Beta-2-glycoprotein I)(Beta(2)GPI)(B2GPI)(Apolipoprotein H)(Apo-H)(Activated protein C-binding protein)(APC inhibitor)(Anticardiolipin cofactor) [Source: Ur
3332.1.1	-4.5	5.8	2002.9771	-0.0031	2	32.15	ctpr	83	VCPFAGILENGAVR	96	yttf	C [84] 500.199, C [84] 57.0215	ENSP00000205948	Beta-2-glycoprotein 1 Precursor (Beta-2-glycoprotein I)(Beta(2)GPI)(B2GPI)(Apolipoprotein H)(Apo-H)(Activated protein C-binding protein)(APC inhibitor)(Anticardiolipin cofactor) [Source: Ur
3333.1.1	-6.7	5.56	1502.7784	-0.0004	2	32.15	ctpr	83	VCPFAGILENGAVR	96	yttf	C [84] 57.0215	ENSP00000205948	Beta-2-glycoprotein 1 Precursor (Beta-2-glycoprotein I)(Beta(2)GPI)(B2GPI)(Apolipoprotein H)(Apo-H)(Activated protein C-binding protein)(APC inhibitor)(Anticardiolipin cofactor) [Source: Ur
3338.1.1	-6	5.96	2002.9771	-0.0049	3	32.15	ctpr	83	VCPFAGILENGAVR	96	yttf	C [84] 500.199, C [84] 57.0215	ENSP00000205948	Beta-2-glycoprotein 1 Precursor (Beta-2-glycoprotein I)(Beta(2)GPI)(B2GPI)(Apolipoprotein H)(Apo-H)(Activated protein C-binding protein)(APC inhibitor)(Anticardiolipin cofactor) [Source: Ur
3355.1.1	-1.7	4.38	1984.946	1.015	3	26.95	ftsk	611	SDVQSGPSLGADPLSSADGPK	631	kkqv		ENSP00000380444:reversed	
3364.1.1	-2	4.55	1984.946	0.015	3	26.95	ftsk	611	SDVQSGPSLGADPLSSADGPK	631	kkqv		ENSP00000380444:reversed	
3366.1.1	-2	4.18	1251.7096	0.0027	2	31.59	kiak	46	YIPIQYVLSR	55	ypsy		sp|CASK_BOVIN|	Kappa-casein precursor
3370.1.1	-2.9	4.57	1984.9458	0.0085	3	26.95	ftsk	611	SDVQSGPSLGADPLSSADGPK	631	kkqv		ENSP00000380444:reversed	
3371.1.1	-1.1	4.4	2019.951	0.018	3	27.17	eask	32	NETGGGEGVEVLVNEPYEK	50	dgek		ENSP00000316809	Phosphatidylinositol transfer protein alpha isoform (PtdIns transfer protein alpha) (PtdInsTP) (PI-TP-alpha). [Source: UniProt Q00169]
3373.1.1	-2.9	4.12	1251.7096	0.0038	2	31.59	kiak	46	YIPIQYVLSR	55	ypsy		sp|CASK_BOVIN|	Kappa-casein precursor
3378.1.1	-2.4	4.3	1215.6984	0.0008	2	33.72	pkpd	28	DLPFSTVVPLK	38	tfye		ENSP00000205948	Beta-2-glycoprotein 1 Precursor (Beta-2-glycoprotein I)(Beta(2)GPI)(B2GPI)(Apolipoprotein H)(Apo-H)(Activated protein C-binding protein)(APC inhibitor)(Anticardiolipin cofactor) [Source: Ur
3385.1.1	-1.7	4.21	2433.255	-0.013	3	38.38	evik	426	EFSNVSVGKLVEAGCVANNAVIR	448	knav	C [440] 57.0215	ENSP00000262429	Probable calcium-transporting ATPase KIAA0703 (EC 3.6.3.8). [Source: UniProt O75185]
3389.1.1	-2	4.47	1215.6984	0.0009	2	33.72	pkpd	28	DLPFSTVVPLK	38	tfye		ENSP00000205948	Beta-2-glycoprotein 1 Precursor (Beta-2-glycoprotein I)(Beta(2)GPI)(B2GPI)(Apolipoprotein H)(Apo-H)(Activated protein C-binding protein)(APC inhibitor)(Anticardiolipin cofactor) [Source: Ur
3394.1.1	-5.3	4.67	1503.7632	0.0017	3	32.15	ctpr	83	VCPFAGILENGAVR	96	yttf	C [84] 57.0215, N [92] 0.9848	ENSP00000205948	Beta-2-glycoprotein 1 Precursor (Beta-2-glycoprotein I)(Beta(2)GPI)(B2GPI)(Apolipoprotein H)(Apo-H)(Activated protein C-binding protein)(APC inhibitor)(Anticardiolipin cofactor) [Source: Ur
3396.1.1	-5.1	4.7	1503.7632	0.0002	3	32.15	ctpr	83	VCPFAGILENGAVR	96	yttf	C [84] 57.0215, N [92] 0.9848	ENSP00000205948	Beta-2-glycoprotein 1 Precursor (Beta-2-glycoprotein I)(Beta(2)GPI)(B2GPI)(Apolipoprotein H)(Apo-H)(Activated protein C-binding protein)(APC inhibitor)(Anticardiolipin cofactor) [Source: Ur
3400.1.1	-5.4	4.35	1503.7632	0.0013	3	32.15	ctpr	83	VCPFAGILENGAVR	96	yttf	C [84] 57.0215, N [92] 0.9848	ENSP00000205948	Beta-2-glycoprotein 1 Precursor (Beta-2-glycoprotein I)(Beta(2)GPI)(B2GPI)(Apolipoprotein H)(Apo-H)(Activated protein C-binding protein)(APC inhibitor)(Anticardiolipin cofactor) [Source: Ur
3408.1.1	-3.8	5.69	1569.7107	-0.0007	2	34.45	gavr	97	YTTFEYPNTISFS	109	cntg		ENSP00000205948	Beta-2-glycoprotein 1 Precursor (Beta-2-glycoprotein I)(Beta(2)GPI)(B2GPI)(Apolipoprotein H)(Apo-H)(Activated protein C-binding protein)(APC inhibitor)(Anticardiolipin cofactor) [Source: Ur
3412.1.1	-4.1	5.69	1569.7107	-0.0029	2	34.45	gavr	97	YTTFEYPNTISFS	109	cntg		ENSP00000205948	Beta-2-glycoprotein 1 Precursor (Beta-2-glycoprotein I)(Beta(2)GPI)(B2GPI)(Apolipoprotein H)(Apo-H)(Activated protein C-binding protein)(APC inhibitor)(Anticardiolipin cofactor) [Source: Ur
3414.1.1	-8.5	5.12	1503.7632	-0.0032	2	32.15	ctpr	83	VCPFAGILENGAVR	96	yttf	C [84] 57.0215, N [92] 0.9848	ENSP00000205948	Beta-2-glycoprotein 1 Precursor (Beta-2-glycoprotein I)(Beta(2)GPI)(B2GPI)(Apolipoprotein H)(Apo-H)(Activated protein C-binding protein)(APC inhibitor)(Anticardiolipin cofactor) [Source: Ur
3418.1.1	-3.5	5.64	1569.7107	-0.0032	2	34.45	gavr	97	YTTFEYPNTISFS	109	cntg		ENSP00000205948	Beta-2-glycoprotein 1 Precursor (Beta-2-glycoprotein I)(Beta(2)GPI)(B2GPI)(Apolipoprotein H)(Apo-H)(Activated protein C-binding protein)(APC inhibitor)(Anticardiolipin cofactor) [Source: Ur
3422.1.1	-9.4	4.95	1503.7632	-0.0032	2	32.15	ctpr	83	VCPFAGILENGAVR	96	yttf	C [84] 57.0215, N [92] 0.9848	ENSP00000205948	Beta-2-glycoprotein 1 Precursor (Beta-2-glycoprotein I)(Beta(2)GPI)(B2GPI)(Apolipoprotein H)(Apo-H)(Activated protein C-binding protein)(APC inhibitor)(Anticardiolipin cofactor) [Source: Ur
3428.1.1	-5.7	4.76	1503.7632	-0.0013	2	32.15	ctpr	83	VCPFAGILENGAVR	96	yttf	C [84] 57.0215, N [92] 0.9848	ENSP00000205948	Beta-2-glycoprotein 1 Precursor (Beta-2-glycoprotein I)(Beta(2)GPI)(B2GPI)(Apolipoprotein H)(Apo-H)(Activated protein C-binding protein)(APC inhibitor)(Anticardiolipin cofactor) [Source: Ur
3430.1.1	-2.2	4.43	2003.971	-0.015	3	33.52	adas	173	QSDYSKYYPLIEDLRN	188	kiis		ENSP00000251643	Keratin, type I cytoskeletal 12 (Cytokeratin 12) (K12) (CK 12). [Source: SWISSPROT (Q99456)]
3443.1.1	-2.5	4.02	1933.0616	-0.0064	3	47.69	ggmr	63	KFICPLTGLWPINTLK	78	ctpr	C [66] 57.0215, W [72] 31.9898	ENSP00000205948	Beta-2-glycoprotein 1 Precursor (Beta-2-glycoprotein I)(Beta(2)GPI)(B2GPI)(Apolipoprotein H)(Apo-H)(Activated protein C-binding protein)(APC inhibitor)(Anticardiolipin cofactor) [Source: Ur
3467.1.1	-1.3	5.02	4278.077	0.019	4	45.33	iagr	22	TCPKPDDLPFSTVVPLKTFYEPGEE	58	ggmr	C [23] 57.0215, C [51] 57.0215	ENSP00000205948	Beta-2-glycoprotein 1 Precursor (Beta-2-glycoprotein I)(Beta(2)GPI)(B2GPI)(Apolipoprotein H)(Apo-H)(Activated protein C-binding protein)(APC inhibitor)(Anticardiolipin cofactor) [Source: Ur
3468.1.1	-1.7	4.86	4778.276	0.031	4	45.33	iagr	22	TCPKPDDLPFSTVVPLKTFYEPGEE	58	ggmr	C [23] 57.0215, C [51] 500.199, C [51] 57.0	ENSP00000205948	Beta-2-glycoprotein 1 Precursor (Beta-2-glycoprotein I)(Beta(2)GPI)(B2GPI)(Apolipoprotein H)(Apo-H)(Activated protein C-binding protein)(APC inhibitor)(Anticardiolipin cofactor) [Source: Ur
3469.1.1	-1.7	4.38	1661.8931	0.0023	3	38.5	ygkk	38	VMVLDFVTPTPLGTR	52	wglg	M [39] 15.9949	ENSP00000373506	Thioredoxin reductase 1, cytoplasmic (TR)(EC 1.8.1.9)(Thioredoxin reductase TR1)(KM-102-derived reductase-like factor)(Gene associated with retinoid-IFN-induced mortality 12 protein)(GR
3473.1.1	-1.9	5.18	4278.0774	0.0063	4	45.33	iagr	22	TCPKPDDLPFSTVVPLKTFYEPGEE	58	ggmr	C [23] 57.0215, C [51] 57.0215	ENSP00000205948	Beta-2-glycoprotein 1 Precursor (Beta-2-glycoprotein I)(Beta(2)GPI)(B2GPI)(Apolipoprotein H)(Apo-H)(Activated protein C-binding protein)(APC inhibitor)(Anticardiolipin cofactor) [Source: Ur
3493.1.1	-1.2	4.93	2148.1182	-0.0005	3	38.88	kgqk	86	VGEFSGANKEKLEATINELV	105	]		ENSP00000363641	Thioredoxin (Trx)(ATL-derived factor)(ADF)(Surface-associated sulphydryl protein)(SASP) [Source: UniProt P10599]
3497.1.1	-1.5	5.28	2148.1182	-0.0009	3	38.88	kgqk	86	VGEFSGANKEKLEATINELV	105	]		ENSP00000363641	Thioredoxin (Trx)(ATL-derived factor)(ADF)(Surface-associated sulphydryl protein)(SASP) [Source: UniProt P10599]
3511.1.1	-6.2	4.96	2500.1828	-0.0001	3	34.16	ntlk	79	CTPRVCPFAGILENGAVR	96	yttf	C [84] 57.0215, C [79] 500.199, C [79] 57.0	ENSP00000205948	Beta-2-glycoprotein 1 Precursor (Beta-2-glycoprotein I)(Beta(2)GPI)(B2GPI)(Apolipoprotein H)(Apo-H)(Activated protein C-binding protein)(APC inhibitor)(Anticardiolipin cofactor) [Source: Ur
3515.1.1	-5.1	4.29	2351.218	-0.011	3	38.17	lpvc	138	APIICPPPSIPTFATLR	154	vykp	C [142] 500.199, C [142] 57.0215	ENSP00000205948	Beta-2-glycoprotein 1 Precursor (Beta-2-glycoprotein I)(Beta(2)GPI)(B2GPI)(Apolipoprotein H)(Apo-H)(Activated protein C-binding protein)(APC inhibitor)(Anticardiolipin cofactor) [Source: Ur

3516.1.1	-6.3	3.96	1503.7632	0.0056	2	32.15	ctpr	83	VCPFAGILENGAVR	96	yttf	C [84] 57.0215, N [92] 0.9848	ENSP00000205948	Beta-2-glycoprotein 1 Precursor (Beta-2-glycoprotein I)(Beta(2)GPI)(B2GPI)(Apolipoprotein H)(Apo-H)(Activated protein C-binding protein)(APC inhibitor)(Anticardiolipin cofactor) [Source: Ur
3518.1.1	-4.4	5	2500.1828	-0.0021	3	34.16	ntlk	79	CTPRVCPFAGILENGAVR	96	yttf	C [84] 57.0215, C [79] 500.199, C [79] 57.0	ENSP00000205948	Beta-2-glycoprotein 1 Precursor (Beta-2-glycoprotein I)(Beta(2)GPI)(B2GPI)(Apolipoprotein H)(Apo-H)(Activated protein C-binding protein)(APC inhibitor)(Anticardiolipin cofactor) [Source: Ur
3528.1.1	-2.6	4.37	2280.1813	-0.0017	3	39.22	pvca	139	PIICPPPSIPTFATLR	154	vykp	C [142] 500.199, C [142] 57.0215	ENSP00000205948	Beta-2-glycoprotein 1 Precursor (Beta-2-glycoprotein I)(Beta(2)GPI)(B2GPI)(Apolipoprotein H)(Apo-H)(Activated protein C-binding protein)(APC inhibitor)(Anticardiolipin cofactor) [Source: Ur
3534.1.1	-1.7	4.71	2500.1828	-0.0073	3	34.16	ntlk	79	CTPRVCPFAGILENGAVR	96	yttf	C [84] 57.0215, C [79] 500.199, C [79] 57.0	ENSP00000205948	Beta-2-glycoprotein 1 Precursor (Beta-2-glycoprotein I)(Beta(2)GPI)(B2GPI)(Apolipoprotein H)(Apo-H)(Activated protein C-binding protein)(APC inhibitor)(Anticardiolipin cofactor) [Source: Ur
3539.1.1	-11.2	5.23	2282.2292	-0.0008	3	41.25	dner	435	VVGFHVLGPNAGEVTQGFAAAL	457	cglt		ENSP00000373506	Thioredoxin reductase 1, cytoplasmic (TR)(EC 1.8.1.9)(Thioredoxin reductase TR1)(KM-102-derived reductase-like factor)(Gene associated with retinoid-IFN-induced mortality 12 protein)(GR
3543.1.1	-9.7	5.49	2282.2292	-0.0026	3	41.25	dner	435	VVGFHVLGPNAGEVTQGFAAAL	457	cglt		ENSP00000373506	Thioredoxin reductase 1, cytoplasmic (TR)(EC 1.8.1.9)(Thioredoxin reductase TR1)(KM-102-derived reductase-like factor)(Gene associated with retinoid-IFN-induced mortality 12 protein)(GR
3544.1.1	-1.8	4.22	1412.7501	0.0055	2	29.29	qlkr	199	LCIPCLDLPRR	209	laed	C [200] 57.0215, C [203] 57.0215	ENSP00000308149:reversed	
3555.1.1	-5.9	4.93	3568.5605	-0.0056	3	41.58	gavr	97	YTTFEYPNTISFSCNTGFYLNGADS	123	ctee	C [110] 500.199, C [110] 57.0215	ENSP00000205948	Beta-2-glycoprotein 1 Precursor (Beta-2-glycoprotein I)(Beta(2)GPI)(B2GPI)(Apolipoprotein H)(Apo-H)(Activated protein C-binding protein)(APC inhibitor)(Anticardiolipin cofactor) [Source: Ur
3559.1.1	-5.1	4.99	3568.5605	-0.0012	3	41.58	gavr	97	YTTFEYPNTISFSCNTGFYLNGADS	123	ctee	C [110] 500.199, C [110] 57.0215	ENSP00000205948	Beta-2-glycoprotein 1 Precursor (Beta-2-glycoprotein I)(Beta(2)GPI)(B2GPI)(Apolipoprotein H)(Apo-H)(Activated protein C-binding protein)(APC inhibitor)(Anticardiolipin cofactor) [Source: Ur
3564.1.1	-2.5	3.97	1412.7532	-0.0021	2	26.78	lppr	67	TPPYQGDVPLGIR	79	ntic		ENSP00000382991	
3566.1.1	-5.7	5.26	3568.5605	-0.0038	3	41.58	gavr	97	YTTFEYPNTISFSCNTGFYLNGADS	123	ctee	C [110] 500.199, C [110] 57.0215	ENSP00000205948	Beta-2-glycoprotein 1 Precursor (Beta-2-glycoprotein I)(Beta(2)GPI)(B2GPI)(Apolipoprotein H)(Apo-H)(Activated protein C-binding protein)(APC inhibitor)(Anticardiolipin cofactor) [Source: Ur
3623.1.1	-2.2	4.63	3543.7342	0.0077	4	44.98	vaia	20	GRTCPKPDDLPFSTVVPLKTFYEP	50	ckpg	C [23] 57.0215	ENSP00000205948	Beta-2-glycoprotein 1 Precursor (Beta-2-glycoprotein I)(Beta(2)GPI)(B2GPI)(Apolipoprotein H)(Apo-H)(Activated protein C-binding protein)(APC inhibitor)(Anticardiolipin cofactor) [Source: Ur
3631.1.1	-4.4	4.13	1482.6788	0.0017	2	34.42	gavr	97	YTTFEYPNTISF	108	scnt		ENSP00000205948	Beta-2-glycoprotein 1 Precursor (Beta-2-glycoprotein I)(Beta(2)GPI)(B2GPI)(Apolipoprotein H)(Apo-H)(Activated protein C-binding protein)(APC inhibitor)(Anticardiolipin cofactor) [Source: Ur
3635.1.1	-5.2	4.27	1482.6788	-0.0037	2	34.42	gavr	97	YTTFEYPNTISF	108	scnt		ENSP00000205948	Beta-2-glycoprotein 1 Precursor (Beta-2-glycoprotein I)(Beta(2)GPI)(B2GPI)(Apolipoprotein H)(Apo-H)(Activated protein C-binding protein)(APC inhibitor)(Anticardiolipin cofactor) [Source: Ur
3643.1.1	-2.9	4.3	1482.6788	-0.0024	2	34.42	gavr	97	YTTFEYPNTISF	108	scnt		ENSP00000205948	Beta-2-glycoprotein 1 Precursor (Beta-2-glycoprotein I)(Beta(2)GPI)(B2GPI)(Apolipoprotein H)(Apo-H)(Activated protein C-binding protein)(APC inhibitor)(Anticardiolipin cofactor) [Source: Ur
3658.1.1	-1.3	4.37	3568.561	0.992	3	41.58	gavr	97	YTTFEYPNTISFSCNTGFYLNGADS	123	ctee	C [110] 500.199, C [110] 57.0215	ENSP00000205948	Beta-2-glycoprotein 1 Precursor (Beta-2-glycoprotein I)(Beta(2)GPI)(B2GPI)(Apolipoprotein H)(Apo-H)(Activated protein C-binding protein)(APC inhibitor)(Anticardiolipin cofactor) [Source: Ur
3673.1.1	-4.1	4.23	1204.5166	0.002	2	32.64	cevk	73	CMPTFQFFK	81	kgqk	C [73] 57.0215, M [74] 15.9949, C [73] -17	ENSP00000363641	Thioredoxin (Trx)(ATL-derived factor)(ADF)(Surface-associated sulphydryl protein)(SASP) [Source: UniProt P10599]
3679.1.1	-5.3	4.13	1204.5166	0.0019	2	32.64	cevk	73	CMPTFQFFK	81	kgqk	C [73] 57.0215, M [74] 15.9949, C [73] -17	ENSP00000363641	Thioredoxin (Trx)(ATL-derived factor)(ADF)(Surface-associated sulphydryl protein)(SASP) [Source: UniProt P10599]
3685.1.1	-3.1	4.11	1204.5166	-0.0017	2	32.64	cevk	73	CMPTFQFFK	81	kgqk	C [73] 57.0215, M [74] 15.9949, C [73] -17	ENSP00000363641	Thioredoxin (Trx)(ATL-derived factor)(ADF)(Surface-associated sulphydryl protein)(SASP) [Source: UniProt P10599]
3688.1.1	-4.7	4.32	3494.589	0.018	3	44.67	erpr	167	YLGIPGDKEYCISSDDLFSLPYCPG	192	tlvv	C [177] 57.0215, C [189] 500.199, C [189]	ENSP00000373506	Thioredoxin reductase 1, cytoplasmic (TR)(EC 1.8.1.9)(Thioredoxin reductase TR1)(KM-102-derived reductase-like factor)(Gene associated with retinoid-IFN-induced mortality 12 protein)(GR
3695.1.1	-3.1	4.48	4079.97	-0.0013	4	47.55	pkpd	28	DLPFSTVVPLKTFYEPGEEITYSCKF	58	ggmr	C [51] 500.199, C [51] 57.0215	ENSP00000205948	Beta-2-glycoprotein 1 Precursor (Beta-2-glycoprotein I)(Beta(2)GPI)(B2GPI)(Apolipoprotein H)(Apo-H)(Activated protein C-binding protein)(APC inhibitor)(Anticardiolipin cofactor) [Source: Ur
3696.1.1	-5.1	4.61	1645.8982	0.0001	2	38.5	ygkk	38	VMVLDFVTPTPLGTR	52	wglg		ENSP00000373506	Thioredoxin reductase 1, cytoplasmic (TR)(EC 1.8.1.9)(Thioredoxin reductase TR1)(KM-102-derived reductase-like factor)(Gene associated with retinoid-IFN-induced mortality 12 protein)(GR
3701.1.1	-6.8	4.95	1645.8982	0.0005	2	38.5	ygkk	38	VMVLDFVTPTPLGTR	52	wglg		ENSP00000373506	Thioredoxin reductase 1, cytoplasmic (TR)(EC 1.8.1.9)(Thioredoxin reductase TR1)(KM-102-derived reductase-like factor)(Gene associated with retinoid-IFN-induced mortality 12 protein)(GR
3702.1.1	-2.5	4.68	2507.189	0.981	3	34.16	ntlk	79	CTPRVCPFAGILENGAVR	96	yttf	C [84] 57.0215, C [79] 500.199, C [79] 57.0	ENSP00000205948	Beta-2-glycoprotein 1 Precursor (Beta-2-glycoprotein I)(Beta(2)GPI)(B2GPI)(Apolipoprotein H)(Apo-H)(Activated protein C-binding protein)(APC inhibitor)(Anticardiolipin cofactor) [Source: Ur
3705.1.1	-8.5	3.82	2283.214	0.014	3	41.25	dner	435	VVGFHVLGPNAGEVTQGFAAAL	457	cglt	N [444] 0.9848	ENSP00000373506	Thioredoxin reductase 1, cytoplasmic (TR)(EC 1.8.1.9)(Thioredoxin reductase TR1)(KM-102-derived reductase-like factor)(Gene associated with retinoid-IFN-induced mortality 12 protein)(GR
3707.1.1	-7.7	4.86	1645.8982	0.002	2	38.5	ygkk	38	VMVLDFVTPTPLGTR	52	wglg		ENSP00000373506	Thioredoxin reductase 1, cytoplasmic (TR)(EC 1.8.1.9)(Thioredoxin reductase TR1)(KM-102-derived reductase-like factor)(Gene associated with retinoid-IFN-induced mortality 12 protein)(GR
3708.1.1	-4.1	4.62	2507.189	0.985	3	34.16	ntlk	79	CTPRVCPFAGILENGAVR	96	yttf	C [84] 57.0215, C [79] 500.199, C [79] 57.0	ENSP00000205948	Beta-2-glycoprotein 1 Precursor (Beta-2-glycoprotein I)(Beta(2)GPI)(B2GPI)(Apolipoprotein H)(Apo-H)(Activated protein C-binding protein)(APC inhibitor)(Anticardiolipin cofactor) [Source: Ur
3710.1.1	-2	4.14	4079.97	0.026	4	47.55	pkpd	28	DLPFSTVVPLKTFYEPGEEITYSCKF	58	ggmr	C [51] 500.199, C [51] 57.0215	ENSP00000205948	Beta-2-glycoprotein 1 Precursor (Beta-2-glycoprotein I)(Beta(2)GPI)(B2GPI)(Apolipoprotein H)(Apo-H)(Activated protein C-binding protein)(APC inhibitor)(Anticardiolipin cofactor) [Source: Ur
3716.1.1	-2.8	4.85	4043.9329	0.007	4	44.98	vaia	20	GRTCPKPDDLPFSTVVPLKTFYEP	50	ckpg	C [23] 500.199, C [23] 57.0215	ENSP00000205948	Beta-2-glycoprotein 1 Precursor (Beta-2-glycoprotein I)(Beta(2)GPI)(B2GPI)(Apolipoprotein H)(Apo-H)(Activated protein C-binding protein)(APC inhibitor)(Anticardiolipin cofactor) [Source: Ur
3718.1.1	-4.1	4.41	2994.39	0.0013	3	44.67	erpr	167	YLGIPGDKEYCISSDDLFSLPYCPG	192	tlvv	C [177] 57.0215, C [189] 57.0215	ENSP00000373506	Thioredoxin reductase 1, cytoplasmic (TR)(EC 1.8.1.9)(Thioredoxin reductase TR1)(KM-102-derived reductase-like factor)(Gene associated with retinoid-IFN-induced mortality 12 protein)(GR
3723.1.1	-3.8	4.44	2994.39	-0.0018	3	44.67	erpr	167	YLGIPGDKEYCISSDDLFSLPYCPG	192	tlvv	C [177] 57.0215, C [189] 57.0215	ENSP00000373506	Thioredoxin reductase 1, cytoplasmic (TR)(EC 1.8.1.9)(Thioredoxin reductase TR1)(KM-102-derived reductase-like factor)(Gene associated with retinoid-IFN-induced mortality 12 protein)(GR
3730.1.1	-4.2	4.62	2994.39	-0.0018	3	44.67	erpr	167	YLGIPGDKEYCISSDDLFSLPYCPG	192	tlvv	C [177] 57.0215, C [189] 57.0215	ENSP00000373506	Thioredoxin reductase 1, cytoplasmic (TR)(EC 1.8.1.9)(Thioredoxin reductase TR1)(KM-102-derived reductase-like factor)(Gene associated with retinoid-IFN-induced mortality 12 protein)(GR
3790.1.1	-3.1	4.4	2401.2704	-0.0056	3	47.69	ggmr	63	KFICPLTGLWPINTLK	78	ctpr	C [66] 500.199, C [66] 57.0215	ENSP00000205948	Beta-2-glycoprotein 1 Precursor (Beta-2-glycoprotein I)(Beta(2)GPI)(B2GPI)(Apolipoprotein H)(Apo-H)(Activated protein C-binding protein)(APC inhibitor)(Anticardiolipin cofactor) [Source: Ur
3797.1.1	-2.1	4.61	2401.2704	-0.0026	3	47.69	ggmr	63	KFICPLTGLWPINTLK	78	ctpr	C [66] 500.199, C [66] 57.0215	ENSP00000205948	Beta-2-glycoprotein 1 Precursor (Beta-2-glycoprotein I)(Beta(2)GPI)(B2GPI)(Apolipoprotein H)(Apo-H)(Activated protein C-binding protein)(APC inhibitor)(Anticardiolipin cofactor) [Source: Ur
3800.1.1	-5.3	4.14	1302.7151	0.0018	2	33.09	dnnr	344	SLDLDSIIAEVK	355	aqye		ENSP00000252244	Keratin, type II cytoskeletal 1 (Cytokeratin-1) (CK-1) (Keratin-1) (K1) (67 kDa cytokeratin) (Hair alpha protein). Source: Uniprot/SWISSPROT P04264
3802.1.1	-5.1	4.56	3330.612	0.015	3	47.14	iagr	22	TCPKPDDLPFSTVVPLKTFYEPGEE	50	ckpg	C [23] 57.0215	ENSP00000205948	Beta-2-glycoprotein 1 Precursor (Beta-2-glycoprotein I)(Beta(2)GPI)(B2GPI)(Apolipoprotein H)(Apo-H)(Activated protein C-binding protein)(APC inhibitor)(Anticardiolipin cofactor) [Source: Ur
3803.1.1	-4.4	4.3	1329.726	0.0003	2	32.62	dnnr	327	NLDLDSIIAEVK	338	aqye		ENSP00000252252	Keratin, type II cytoskeletal 6B (Cytokeratin-6B) (CK 6B) (K6b keratin). Source: Uniprot/SWISSPROT P04259
3804.1.1	-6	4.38	1302.7151	0.0025	2	33.09	dnnr	344	SLDLDSIIAEVK	355	aqye		ENSP00000252244	Keratin, type II cytoskeletal 1 (Cytokeratin-1) (CK-1) (Keratin-1) (K1) (67 kDa cytokeratin) (Hair alpha protein). Source: Uniprot/SWISSPROT P04264
3809.1.1	-6.3	4.63	1302.7151	-0.0007	2	33.09	dnnr	344	SLDLDSIIAEVK	355	aqye		ENSP00000252244	Keratin, type II cytoskeletal 1 (Cytokeratin-1) (CK-1) (Keratin-1) (K1) (67 kDa cytokeratin) (Hair alpha protein). Source: Uniprot/SWISSPROT P04264
3810.1.1	-4.4	4.29	1329.726	-0.0003	2	32.62	dnnr	327	NLDLDSIIAEVK	338	aqye		ENSP00000252252	Keratin, type II cytoskeletal 6B (Cytokeratin-6B) (CK 6B) (K6b keratin). Source: Uniprot/SWISSPROT P04259
3811.1.1	-3.4	4.49	3330.6119	-0.0058	3	47.14	iagr	22	TCPKPDDLPFSTVVPLKTFYEPGEE	50	ckpg	C [23] 57.0215	ENSP00000205948	Beta-2-glycoprotein 1 Precursor (Beta-2-glycoprotein I)(Beta(2)GPI)(B2GPI)(Apolipoprotein H)(Apo-H)(Activated protein C-binding protein)(APC inhibitor)(Anticardiolipin cofactor) [Source: Ur
3813.1.1	-2.4	4.56	3633.7802	-0.0024	3	47.72	egkw	131	SPELPVCAPIICPPPSIPTFATLR	154	vykp	C [137] 500.199, C [142] 500.199, C [137]	ENSP00000205948	Beta-2-glycoprotein 1 Precursor (Beta-2-glycoprotein I)(Beta(2)GPI)(B2GPI)(Apolipoprotein H)(Apo-H)(Activated protein C-binding protein)(APC inhibitor)(Anticardiolipin cofactor) [Source: Ur
3816.1.1	-2.7	4.43	4524.1396	-0.0077	4	48.28	dsak	124	CTEEGKWSPELPVCAPIICPPPSIP	154	vykp	C [124] 57.0215, C [137] 500.199, C [142]	ENSP00000205948	Beta-2-glycoprotein 1 Precursor (Beta-2-glycoprotein I)(Beta(2)GPI)(B2GPI)(Apolipoprotein H)(Apo-H)(Activated protein C-binding protein)(APC inhibitor)(Anticardiolipin cofactor) [Source: Ur
3817.1.1	-4	3.91	1329.726	0.0014	2	32.62	dnnr	327	NLDLDSIIAEVK	338	aqye		ENSP00000252252	Keratin, type II cytoskeletal 6B (Cytokeratin-6B) (CK 6B) (K6b keratin). Source: Uniprot/SWISSPROT P04259
3822.1.1	-3.3	4.76	3633.7802	-0.0068	3	47.72	egkw	131	SPELPVCAPIICPPPSIPTFATLR	154	vykp	C [137] 500.199, C [142] 500.199, C [137]	ENSP00000205948	Beta-2-glycoprotein 1 Precursor (Beta-2-glycoprotein I)(Beta(2)GPI)(B2GPI)(Apolipoprotein H)(Apo-H)(Activated protein C-binding protein)(APC inhibitor)(Anticardiolipin cofactor) [Source: Ur
3827.1.1	-3.4	4.69	3633.78	-0.011	3	47.72	egkw	131	SPELPVCAPIICPPPSIPTFATLR	154	vykp	C [137] 500.199, C [142] 500.199, C [137]	ENSP00000205948	Beta-2-glycoprotein 1 Precursor (Beta-2-glycoprotein I)(Beta(2)GPI)(B2GPI)(Apolipoprotein H)(Apo-H)(Activated protein C-binding protein)(APC inhibitor)(Anticardiolipin cofactor) [Source: Ur
3834.1.1	-1.8	4.21	3341.7067	0.0076	4	41.75	dner	435	VVGFHVLGPNAGEVTQGFAAAL	462	kqld	C [458] 500.199, C [458] 57.0215	ENSP00000373506	Thioredoxin reductase 1, cytoplasmic (TR)(EC 1.8.1.9)(Thioredoxin reductase TR1)(KM-102-derived reductase-like factor)(Gene associated with retinoid-IFN-induced mortality 12 protein)(GR
3839.1.1	-1.4	4.51	3287.6064	-0.0061	4	47.73	gmrk	64	FICPLTGLWPINTLKCTPR	82	vcpf	C [66] 500.199, C [79] 500.199, C [66] 57.0	ENSP00000205948	Beta-2-glycoprotein 1 Precursor (Beta-2-glycoprotein I)(Beta(2)GPI)(B2GPI)(Apolipoprotein H)(Apo-H)(Activated protein C-binding protein)(APC inhibitor)(Anticardiolipin cofactor) [Source: Ur
3842.1.1	-2.6	4.07	2841.508	-0.012	4	41.75	dner	435	VVGFHVLGPNAGEVTQGFAAAL	462	kqld	C [458] 57.0215	ENSP00000373506	Thioredoxin reductase 1, cytoplasmic (TR)(EC 1.8.1.9)(Thioredoxin reductase TR1)(KM-102-derived reductase-like factor)(Gene associated with retinoid-IFN-induced mortality 12 protein)(GR
3845.1.1	-2.3	4.58	3287.6064	-0.0096	4	47.73	gmrk	64	FICPLTGLWPINTLKCTPR	82	vcpf	C [66] 500.199, C [79] 500.199, C [66] 57.0	ENSP00000205948	Beta-2-glycoprotein 1 Precursor (Beta-2-glycoprotein I)(Beta(2)GPI)(B2GPI)(Apolipoprotein H)(Apo-H)(Activated protein C-binding protein)(APC inhibitor)(Anticardiolipin cofactor) [Source: Ur
3872.1.1	-7.1	4.23	1687.9088	0.0035	2	38.5	ygkk	38	VMVLDFVTPTPLGTR	52	wglg	V [38] 42.0106	ENSP00000373506	Thioredoxin reductase 1, cytoplasmic (TR)(EC 1.8.1.9)(Thioredoxin reductase TR1)(KM-102-derived reductase-like factor)(Gene associated with retinoid-IFN-induced mortality 12 protein)(GR
3874.1.1	-2.7	4.25	1901.0717	-0.0062	3	47.69	ggmr	63	KFICPLTGLWPINTLK	78	ctpr	C [66] 57.0215	ENSP00000205948	Beta-2-glycoprotein 1 Precursor (Beta-2-glycoprotein I)(Beta(2)GPI)(B2GPI)(Apolipoprotein H)(Apo-H)(Activated protein C-binding protein)(APC inhibitor)(Anticardiolipin cofactor) [Source: Ur
3876.1.1	-4.5	5.1	2401.2704	-0.0041	3	47.69	ggmr	63	KFICPLTGLWPINTLK	78	ctpr	C [66] 500.199, C [66] 57.0215	ENSP00000205948	Beta-2-glycoprotein 1 Precursor (Beta-2-glycoprotein I)(Beta(2)GPI)(B2GPI)(Apolipoprotein H)(Apo-H)(Activated protein C-binding protein)(APC inhibitor)(Anticardiolipin cofactor) [Source: Ur
3880.1.1	-3.7	4.14	1687.9088	0.0053	2	38.5	ygkk	38	VMVLDFVTPTPLGTR	52	wglg	V [38] 42.0106	ENSP00000373506	Thioredoxin reductase 1, cytoplasmic (TR)(EC 1.8.1.9)(Thioredoxin reductase TR1)(KM-102-derived reductase-like factor)(Gene associated with retinoid-IFN-induced mortality 12 protein)(GR
3881.1.1	-2.2	4.45	1901.0717	-0.0049	3	47.69	ggmr	63	KFICPLTGLWPINTLK	78	ctpr	C [66] 57.0215	ENSP00000205948	Beta-2-glycoprotein 1 Precursor (Beta-2-glycoprotein I)(Beta(2)GPI)(B2GPI)(Apolipoprotein H)(Apo-H)(Activated protein C-binding protein)(APC inhibitor)(Anticardiolipin cofactor) [Source: Ur
3883.1.1	-4.3	5.03	2401.2704	-0.0036	3	47.69	ggmr	63	KFICPLTGLWPINTLK	78	ctpr	C [66] 500.199, C [66] 57.0215	ENSP00000205948	Beta-2-glycoprotein 1 Precursor (Beta-2-glycoprotein I)(Beta(2)GPI)(B2GPI)(Apolipoprotein H)(Apo-H)(Activated protein C-binding protein)(APC inhibitor)(Anticardiolipin cofactor) [Source: Ur
3884.1.1	-6.1	4.45	3133.582	-0.017	3	47.72	egkw	131	SPELPVCAPIICPPPSIPTFATLR	154	vykp	C [137] 57.0215, C [142] 500.199, C [142]	ENSP00000205948	Beta-2-glycoprotein 1 Precursor (Beta-2-glycoprotein I)(Beta(2)GPI)(B2GPI)(Apolipoprotein H)(Apo-H)(Activated protein C-binding protein)(APC inhibitor)(Anticardiolipin cofactor) [Source: Ur
3885.1.1	-2	4.57	1901.0717	-0.0008	3	47.69	ggmr	63	KFICPLTGLWPINTLK	78	ctpr	C [66] 57.0215	ENSP00000205948	Beta-2-glycoprotein 1 Precursor (Beta-2-glycoprotein I)(Beta(2)GPI)(B2GPI)(Apolipoprotein H)(Apo-H)(Activated protein C-binding protein)(APC inhibitor)(Anticardiolipin cofactor) [Source: Ur
3886.1.1	-6	3.96	1687.9088	-0.002	2	38.5	ygkk	38	VMVLDFVTPTPLGTR	52	wglg	V [38] 42.0106	ENSP00000373506	Thioredoxin reductase 1, cytoplasmic (TR)(EC 1.8.1.9)(Thioredoxin reductase TR1)(KM-102-derived reductase-like factor)(Gene associated with retinoid-IFN-induced mortality 12 protein)(GR
3888.1.1	-3.1	5.17	2401.2704	-0.0008	3	47.69	ggmr	63	KFICPLTGLWPINTLK	78	ctpr	C [66] 500.199, C [66] 57.0215	ENSP00000205948	Beta-2-glycoprotein 1 Precursor (Beta-2-glycoprotein I)(Beta(2)GPI)(B2GPI)(Apolipoprotein H)(Apo-H)(Activated protein C-binding protein)(APC inhibitor)(Anticardiolipin cofactor) [Source: Ur
3889.1.1	-8.4	4.44	3133.5815	0.0052	3	47.72	egkw	131	SPELPVCAPIICPPPSIPTFATLR	154	vykp	C [142] 57.0215, C [137] 500.199, C [137]	ENSP00000205948	Beta-2-glycoprotein 1 Precursor (Beta-2-glycoprotein I)(Beta(2)GPI)(B2GPI)(Apolipoprotein H)(Apo-H)(Activated protein C-binding protein)(APC inhibitor)(Anticardiolipin cofactor) [Source: Ur
3891.1.1	-4.7	4.02	1788.9717	0.0036	2	46.54	gmrk	64	FICPLTGLWPINTLK	78	ctpr	C [66] 57.0215, W [72] 15.9949	ENSP00000205948	Beta-2-glycoprotein 1 Precursor (Beta-2-glycoprotein I)(Beta(2)GPI)(B2GPI)(Apolipoprotein H)(Apo-H)(Activated protein C-binding protein)(APC inhibitor)(Anticardiolipin cofactor) [Source: Ur
3894.1.1	-8.7	4.29	3133.5815	-0.0047	3	47.72	egkw	131	SPELPVCAPIICPPPSIPTFATLR	154	vykp	C [142] 57.0215, C [137] 500.199, C [137]	ENSP00000205948	Beta-2-glycoprotein 1 Precursor (Beta-2-glycoprotein I)(Beta(2)GPI)(B2GPI)(Apolipoprotein H)(Apo-H)(Activated protein C-binding protein)(APC inhibitor)(Anticardiolipin cofactor) [Source: Ur
3895.1.1	-5.7	4.24	1788.9717	0.0014	2	46.54	gmrk	64	FICPLTGLWPINTLK	78	ctpr	C [66] 57.0215, W [72] 15.9949	ENSP00000205948	Beta-2-glycoprotein 1 Precursor (Beta-2-glycoprotein I)(Beta(2)GPI)(B2GPI)(Apolipoprotein H)(Apo-H)(Activated protein C-binding protein)(APC inhibitor)(Anticardiolipin cofactor) [Source: Ur
3899.1.1	-5	4.2	1788.9717	0.0013	2	46.54	gmrk	64	FICPLTGLWPINTLK	78	ctpr	C [66] 57.0215, W [72] 15.9949	ENSP00000205948	Beta-2-glycoprotein 1 Precursor (Beta-2-glycoprotein I)(Beta(2)GPI)(B2GPI)(Apolipoprotein H)(Apo-H)(Activated protein C-binding protein)(APC inhibitor)(Anticardiolipin cofactor) [Source: Ur
3903.1.1	-1.4	4.19	2787.4077	-0.0007	3	47.73	gmrk	64	FICPLTGLWPINTLKCTPR	82	vcpf	C [66] 57.0215, C [79] 500.199, C [79] 57.0	ENSP00000205948	Beta-2-glycoprotein 1 Precursor (Beta-2-glycoprotein I)(Beta(2)GPI)(B2GPI)(Apolipoprotein H)(Apo-H)(Activated protein C-binding protein)(APC inhibitor)(Anticardiolipin cofactor) [Source: Ur
3904.1.1	-4.4	4.09	1788.9717	0.0002	3	46.54	gmrk	64	FICPLTGLWPINTLK	78	ctpr	C [66] 57.0215, W [72] 15.9949	ENSP00000205948	Beta-2-glycoprotein 1 Precursor (Beta-2-glycoprotein I)(Beta(2)GPI)(B2GPI)(Apolipoprotein H)(Apo-H)(Activated protein C-binding protein)(APC inhibitor)(Anticardiolipin cofactor) [Source: Ur
3905.1.1	-2.1	3.94	1917.0667	-0.0099	3	47.69	ggmr	63	KFICPLTGLWPINTLK	78	ctpr	C [66] 57.0215, W [72] 15.9949	ENSP00000205948	Beta-2-glycoprotein 1 Precursor (Beta-2-glycoprotein I)(Beta(2)GPI)(B2GPI)(Apolipoprotein H)(Apo-H)(Activated protein C-binding protein)(APC inhibitor)(Anticardiolipin cofactor) [Source: Ur
3908.1.1	-2.1	4.39	2787.4077	-0.003	3	47.73	gmrk	64	FICPLTGLWPINTLKCTPR	82	vcpf	C [66] 57.0215, C [79] 500.199, C [79] 57.0	ENSP00000205948	Beta-2-glycoprotein 1 Precursor (Beta-2-glycoprotein I)(Beta(2)GPI)(B2GPI)(Apolipoprotein H)(Apo-H)(Activated protein C-binding protein)(APC inhibitor)(Anticardiolipin cofactor) [Source: Ur
3912.1.1	-8.3	4.61	3523.7422	-0.0057	3	48.28	dsak	124	CTEEGKWSPELPVCAPIICPPPSIP	154	vykp	C [124] 57.0215, C [137] 57.0215, C [142]	ENSP00000205948	Beta-2-glycoprotein 1 Precursor (Beta-2-glycoprotein I)(Beta(2)GPI)(B2GPI)(Apolipoprotein H)(Apo-H)(Activated protein C-binding protein)(APC inhibitor)(Anticardiolipin cofactor) [Source: Ur
3913.1.1	-2.8	4.49	2787.4077	-0.0032	3	47.73	gmrk	64	FICPLTGLWPINTLKCTPR	82	vcpf	C [66] 57.0215, C [79] 500.199, C [79] 57.0	ENSP00000205948	Beta-2-glycoprotein 1 Precursor (Beta-2-glycoprotein I)(Beta(2)GPI)(B2GPI)(Apolipoprotein H)(Apo-H)(Activated protein C-binding protein)(APC inhibitor)(Anticardiolipin cofactor) [Source: Ur
3914.1.1	-6.7	3.84	1901.0717	0.007	2	47.69	ggmr	63	KFICPLTGLWPINTLK	78	ctpr	C [66] 57.0215	ENSP00000205948	Beta-2-glycoprotein 1 Precursor (Beta-2-glycoprotein I)(Beta(2)GPI)(B2GPI)(Apolipoprotein H)(Apo-H)(Activated protein C-binding protein)(APC inhibitor)(Anticardiolipin cofactor) [Source: Ur
3917.1.1	-7.1	4.39	3523.7422	-0.0002	3	48.28	dsak	124	CTEEGKWSPELPVCAPIICPPPSIP	154	vykp	C [124] 57.0215, C [137] 57.0215, C [142]	ENSP00000205948	Beta-2-glycoprotein 1 Precursor (Beta-2-glycoprotein I)(Beta(2)GPI)(B2GPI)(Apolipoprotein H)(Apo-H)(Activated protein C-binding protein)(APC inhibitor)(Anticardiolipin cofactor) [Source: Ur
3918.1.1	-3.4	4.04	1788.9717	0.0028	3	46.54	gmrk	64	FICPLTGLWPINTLK	78	ctpr	C [66] 57.0215, W [72] 15.9949	ENSP00000205948	Beta-2-glycoprotein 1 Precursor (Beta-2-glycoprotein I)(Beta(2)GPI)(B2GPI)(Apolipoprotein H)(Apo-H)(Activated protein C-binding protein)(APC inhibitor)(Anticardiolipin cofactor) [Source: Ur
3921.1.1	-8.5	4.31	3523.7422	-0.0042	3	48.28	dsak	124	CTEEGKWSPELPVCAPIICPPPSIP	154	vykp	C [124] 57.0215, C [137] 57.0215, C [142]	ENSP00000205948	Beta-2-glycoprotein 1 Precursor (Beta-2-glycoprotein I)(Beta(2)GPI)(B2GPI)(Apolipoprotein H)(Apo-H)(Activated protein C-binding protein)(APC inhibitor)(Anticardiolipin cofactor) [Source: Ur
3924.1.1	-1.7	3.85	1901.0717	0.0012	2	47.69	ggmr	63	KFICPLTGLWPINTLK	78	ctpr	C [66] 57.0215	ENSP00000205948	Beta-2-glycoprotein 1 Precursor (Beta-2-glycoprotein I)(Beta(2)GPI)(B2GPI)(Apolipoprotein H)(Apo-H)(Activated protein C-binding protein)(APC inhibitor)(Anticardiolipin cofactor) [Source: Ur
3925.1.1	-3.4	4.11	1788.9717	0.0022	3	46.54	gmrk	64	FICPLTGLWPINTLK	78	ctpr	C [66] 57.0215, W [72] 15.9949	ENSP00000205948	Beta-2-glycoprotein 1 Precursor (Beta-2-glycoprotein I)(Beta(2)GPI)(B2GPI)(Apolipoprotein H)(Apo-H)(Activated protein C-binding protein)(APC inhibitor)(Anticardiolipin cofactor) [Source: Ur
3928.1.1	-4	3.87	1901.0717	0.0079	2	47.69	ggmr	63	KFICPLTGLWPINTLK	78	ctpr	C [66] 57.0215	ENSP00000205948	Beta-2-glycoprotein 1 Precursor (Beta-2-glycoprotein I)(Beta(2)GPI)(B2GPI)(Apolipoprotein H)(Apo-H)(Activated protein C-binding protein)(APC inhibitor)(Anticardiolipin cofactor) [Source: Ur
3938.1.1	-5.1	3.91	2633.3828	0.0052	3	47.72	egkw	131	SPELPVCAPIICPPPSIPTFATLR	154	vykp	C [137] 57.0215, C [142] 57.0215	ENSP00000205948	Beta-2-glycoprotein 1 Precursor (Beta-2-glycoprotein I)(Beta(2)GPI)(B2GPI)(Apolipoprotein H)(Apo-H)(Activated protein C-binding protein)(APC inhibitor)(Anticardiolipin cofactor) [Source: Ur
3957.1.1	-11.1	4.22	1384.73	-0.0012	2	39.55	nllr	38	FFVAPFPEVFGK	49	ekvn		sp|CAS1_BOVIN|	Alpha-S1-casein; Contains: Antioxidant peptide; Flags: Precursor;
3961.1.1	-5.5	4.77	1901.0717	-0.0007	3	47.69	ggmr	63	KFICPLTGLWPINTLK	78	ctpr	C [66] 57.0215	ENSP00000205948	Beta-2-glycoprotein 1 Precursor (Beta-2-glycoprotein I)(Beta(2)GPI)(B2GPI)(Apolipoprotein H)(Apo-H)(Activated protein C-binding protein)(APC inhibitor)(Anticardiolipin cofactor) [Source: Ur
3962.1.1	-8.2	4.26	1384.73	-0.0019	2	39.55	nllr	38	FFVAPFPEVFGK	49	ekvn		sp|CAS1_BOVIN|	Alpha-S1-casein; Contains: Antioxidant peptide; Flags: Precursor;
3966.1.1	-8.5	4.74	1901.0717	-0.0012	3	47.69	ggmr	63	KFICPLTGLWPINTLK	78	ctpr	C [66] 57.0215	ENSP00000205948	Beta-2-glycoprotein 1 Precursor (Beta-2-glycoprotein I)(Beta(2)GPI)(B2GPI)(Apolipoprotein H)(Apo-H)(Activated protein C-binding protein)(APC inhibitor)(Anticardiolipin cofactor) [Source: Ur
3967.1.1	-10.3	4.35	1384.73	-0.0004	2	39.55	nllr	38	FFVAPFPEVFGK	49	ekvn		sp|CAS1_BOVIN|	Alpha-S1-casein; Contains: Antioxidant peptide; Flags: Precursor;
3968.1.1	-2.5	4.21	2401.2704	-0.0021	3	47.69	ggmr	63	KFICPLTGLWPINTLK	78	ctpr	C [66] 500.199, C [66] 57.0215	ENSP00000205948	Beta-2-glycoprotein 1 Precursor (Beta-2-glycoprotein I)(Beta(2)GPI)(B2GPI)(Apolipoprotein H)(Apo-H)(Activated protein C-binding protein)(APC inhibitor)(Anticardiolipin cofactor) [Source: Ur
3970.1.1	-5.5	4.62	1901.0717	0.001	3	47.69	ggmr	63	KFICPLTGLWPINTLK	78	ctpr	C [66] 57.0215	ENSP00000205948	Beta-2-glycoprotein 1 Precursor (Beta-2-glycoprotein I)(Beta(2)GPI)(B2GPI)(Apolipoprotein H)(Apo-H)(Activated protein C-binding protein)(APC inhibitor)(Anticardiolipin cofactor) [Source: Ur
3971.1.1	-3.5	4.15	2401.2704	-0.0036	3	47.69	ggmr	63	KFICPLTGLWPINTLK	78	ctpr	C [66] 500.199, C [66] 57.0215	ENSP00000205948	Beta-2-glycoprotein 1 Precursor (Beta-2-glycoprotein I)(Beta(2)GPI)(B2GPI)(Apolipoprotein H)(Apo-H)(Activated protein C-binding protein)(APC inhibitor)(Anticardiolipin cofactor) [Source: Ur
3973.1.1	-4.8	3.99	2888.388	0.011	3	38.41	kdlr	296	NVSTGDVNVEMNAAPGVDLTQL	322	sqye	M [306] 15.9949	ENSP00000269576	Keratin, type I cytoskeletal 10 (Cytokeratin-10) (CK-10) (Keratin-10) (K10). Source: Uniprot/SWISSPROT P13645
3979.1.1	-6.9	3.96	1788.9717	-0.0028	2	46.54	gmrk	64	FICPLTGLWPINTLK	78	ctpr	C [66] 57.0215, W [72] 15.9949	ENSP00000205948	Beta-2-glycoprotein 1 Precursor (Beta-2-glycoprotein I)(Beta(2)GPI)(B2GPI)(Apolipoprotein H)(Apo-H)(Activated protein C-binding protein)(APC inhibitor)(Anticardiolipin cofactor) [Source: Ur
3984.1.1	-5	3.98	1788.9717	0.003	2	46.54	gmrk	64	FICPLTGLWPINTLK	78	ctpr	C [66] 57.0215, W [72] 15.9949	ENSP00000205948	Beta-2-glycoprotein 1 Precursor (Beta-2-glycoprotein I)(Beta(2)GPI)(B2GPI)(Apolipoprotein H)(Apo-H)(Activated protein C-binding protein)(APC inhibitor)(Anticardiolipin cofactor) [Source: Ur
3992.1.1	-7.5	3.88	1788.9717	0.0016	2	46.54	gmrk	64	FICPLTGLWPINTLK	78	ctpr	C [66] 57.0215, W [72] 15.9949	ENSP00000205948	Beta-2-glycoprotein 1 Precursor (Beta-2-glycoprotein I)(Beta(2)GPI)(B2GPI)(Apolipoprotein H)(Apo-H)(Activated protein C-binding protein)(APC inhibitor)(Anticardiolipin cofactor) [Source: Ur
4001.1.1	-1.8	4.56	2787.4077	-0.0073	3	47.73	gmrk	64	FICPLTGLWPINTLKCTPR	82	vcpf	C [66] 57.0215, C [79] 500.199, C [79] 57.0	ENSP00000205948	Beta-2-glycoprotein 1 Precursor (Beta-2-glycoprotein I)(Beta(2)GPI)(B2GPI)(Apolipoprotein H)(Apo-H)(Activated protein C-binding protein)(APC inhibitor)(Anticardiolipin cofactor) [Source: Ur
4012.1.1	-2.3	4.41	2787.4077	-0.0063	3	47.73	gmrk	64	FICPLTGLWPINTLKCTPR	82	vcpf	C [66] 57.0215, C [79] 500.199, C [79] 57.0	ENSP00000205948	Beta-2-glycoprotein 1 Precursor (Beta-2-glycoprotein I)(Beta(2)GPI)(B2GPI)(Apolipoprotein H)(Apo-H)(Activated protein C-binding protein)(APC inhibitor)(Anticardiolipin cofactor) [Source: Ur
4015.1.1	-2.1	4.43	2787.408	-0.013	3	47.73	gmrk	64	FICPLTGLWPINTLKCTPR	82	vcpf	C [66] 57.0215, C [79] 500.199, C [79] 57.0	ENSP00000205948	Beta-2-glycoprotein 1 Precursor (Beta-2-glycoprotein I)(Beta(2)GPI)(B2GPI)(Apolipoprotein H)(Apo-H)(Activated protein C-binding protein)(APC inhibitor)(Anticardiolipin cofactor) [Source: Ur
4017.1.1	-2.2	3.86	1788.972	-0.014	3	46.54	gmrk	64	FICPLTGLWPINTLK	78	ctpr	C [66] 57.0215, W [72] 15.9949	ENSP00000205948	Beta-2-glycoprotein 1 Precursor (Beta-2-glycoprotein I)(Beta(2)GPI)(B2GPI)(Apolipoprotein H)(Apo-H)(Activated protein C-binding protein)(APC inhibitor)(Anticardiolipin cofactor) [Source: Ur
4057.1.1	-4.9	3.98	1804.9666	0.0084	2	46.54	gmrk	64	FICPLTGLWPINTLK	78	ctpr	C [66] 57.0215, W [72] 31.9898	ENSP00000205948	Beta-2-glycoprotein 1 Precursor (Beta-2-glycoprotein I)(Beta(2)GPI)(B2GPI)(Apolipoprotein H)(Apo-H)(Activated protein C-binding protein)(APC inhibitor)(Anticardiolipin cofactor) [Source: Ur

4061.1.1	-4.1	4.12	1804.9666	0.0027	2	46.54	gmrk	64	FICPLTGLWPINTLK	78	ctpr	C [66] 57.0215, W [72] 31.9898	ENSP00000205948	Beta-2-glycoprotein 1 Precursor (Beta-2-glycoprotein I)(Beta(2)GPI)(B2GPI)(Apolipoprotein H)(Apo-H)(Activated protein C-binding protein)(APC inhibitor)(Anticardiolipin cofactor) [Source: Ur
4062.1.1	-4.2	3.78	1788.9717	0.0027	2	46.54	gmrk	64	FICPLTGLWPINTLK	78	ctpr	C [66] 57.0215, W [72] 15.9949	ENSP00000205948	Beta-2-glycoprotein 1 Precursor (Beta-2-glycoprotein I)(Beta(2)GPI)(B2GPI)(Apolipoprotein H)(Apo-H)(Activated protein C-binding protein)(APC inhibitor)(Anticardiolipin cofactor) [Source: Ur
4064.1.1	-5.6	4.39	1804.9666	0.0057	2	46.54	gmrk	64	FICPLTGLWPINTLK	78	ctpr	C [66] 57.0215, W [72] 31.9898	ENSP00000205948	Beta-2-glycoprotein 1 Precursor (Beta-2-glycoprotein I)(Beta(2)GPI)(B2GPI)(Apolipoprotein H)(Apo-H)(Activated protein C-binding protein)(APC inhibitor)(Anticardiolipin cofactor) [Source: Ur
4065.1.1	-3.4	4.46	2273.1755	0.0033	2	46.54	gmrk	64	FICPLTGLWPINTLK	78	ctpr	C [66] 500.199, C [66] 57.0215	ENSP00000205948	Beta-2-glycoprotein 1 Precursor (Beta-2-glycoprotein I)(Beta(2)GPI)(B2GPI)(Apolipoprotein H)(Apo-H)(Activated protein C-binding protein)(APC inhibitor)(Anticardiolipin cofactor) [Source: Ur
4066.1.1	-5.4	4.49	2273.1755	0.0022	3	46.54	gmrk	64	FICPLTGLWPINTLK	78	ctpr	C [66] 500.199, C [66] 57.0215	ENSP00000205948	Beta-2-glycoprotein 1 Precursor (Beta-2-glycoprotein I)(Beta(2)GPI)(B2GPI)(Apolipoprotein H)(Apo-H)(Activated protein C-binding protein)(APC inhibitor)(Anticardiolipin cofactor) [Source: Ur
4068.1.1	-3.7	3.82	1788.9717	0.0004	2	46.54	gmrk	64	FICPLTGLWPINTLK	78	ctpr	C [66] 57.0215, W [72] 15.9949	ENSP00000205948	Beta-2-glycoprotein 1 Precursor (Beta-2-glycoprotein I)(Beta(2)GPI)(B2GPI)(Apolipoprotein H)(Apo-H)(Activated protein C-binding protein)(APC inhibitor)(Anticardiolipin cofactor) [Source: Ur
4070.1.1	-2.3	4.59	2273.1755	0.0001	2	46.54	gmrk	64	FICPLTGLWPINTLK	78	ctpr	C [66] 500.199, C [66] 57.0215	ENSP00000205948	Beta-2-glycoprotein 1 Precursor (Beta-2-glycoprotein I)(Beta(2)GPI)(B2GPI)(Apolipoprotein H)(Apo-H)(Activated protein C-binding protein)(APC inhibitor)(Anticardiolipin cofactor) [Source: Ur
4071.1.1	-4.1	4.68	2273.1755	-0.0057	3	46.54	gmrk	64	FICPLTGLWPINTLK	78	ctpr	C [66] 500.199, C [66] 57.0215	ENSP00000205948	Beta-2-glycoprotein 1 Precursor (Beta-2-glycoprotein I)(Beta(2)GPI)(B2GPI)(Apolipoprotein H)(Apo-H)(Activated protein C-binding protein)(APC inhibitor)(Anticardiolipin cofactor) [Source: Ur
4075.1.1	-1.5	4.84	2273.1755	-0.0043	2	46.54	gmrk	64	FICPLTGLWPINTLK	78	ctpr	C [66] 500.199, C [66] 57.0215	ENSP00000205948	Beta-2-glycoprotein 1 Precursor (Beta-2-glycoprotein I)(Beta(2)GPI)(B2GPI)(Apolipoprotein H)(Apo-H)(Activated protein C-binding protein)(APC inhibitor)(Anticardiolipin cofactor) [Source: Ur
4076.1.1	-6.8	4.87	2273.1755	-0.0068	3	46.54	gmrk	64	FICPLTGLWPINTLK	78	ctpr	C [66] 500.199, C [66] 57.0215	ENSP00000205948	Beta-2-glycoprotein 1 Precursor (Beta-2-glycoprotein I)(Beta(2)GPI)(B2GPI)(Apolipoprotein H)(Apo-H)(Activated protein C-binding protein)(APC inhibitor)(Anticardiolipin cofactor) [Source: Ur
4079.1.1	-2.4	3.87	1788.9717	0.0015	2	46.54	gmrk	64	FICPLTGLWPINTLK	78	ctpr	C [66] 57.0215, W [72] 15.9949	ENSP00000205948	Beta-2-glycoprotein 1 Precursor (Beta-2-glycoprotein I)(Beta(2)GPI)(B2GPI)(Apolipoprotein H)(Apo-H)(Activated protein C-binding protein)(APC inhibitor)(Anticardiolipin cofactor) [Source: Ur
4124.1.1	-10.3	4.39	3506.7157	-0.0014	3	48.28	dsak	124	CTEEGKWSPELPVCAPIICPPPSIP	154	vykp	C [124] 57.0215, C [137] 57.0215, C [142] 5	ENSP00000205948	Beta-2-glycoprotein 1 Precursor (Beta-2-glycoprotein I)(Beta(2)GPI)(B2GPI)(Apolipoprotein H)(Apo-H)(Activated protein C-binding protein)(APC inhibitor)(Anticardiolipin cofactor) [Source: Ur
4126.1.1	-11.3	4.49	3506.7157	0.0012	3	48.28	dsak	124	CTEEGKWSPELPVCAPIICPPPSIP	154	vykp	C [124] 57.0215, C [137] 57.0215, C [142] 5	ENSP00000205948	Beta-2-glycoprotein 1 Precursor (Beta-2-glycoprotein I)(Beta(2)GPI)(B2GPI)(Apolipoprotein H)(Apo-H)(Activated protein C-binding protein)(APC inhibitor)(Anticardiolipin cofactor) [Source: Ur
4131.1.1	-4.3	3.57	3522.711	1.025	3	48.28	dsak	124	CTEEGKWSPELPVCAPIICPPPSIP	154	vykp	C [124] 57.0215, C [137] 57.0215, C [142] 5	ENSP00000205948	Beta-2-glycoprotein 1 Precursor (Beta-2-glycoprotein I)(Beta(2)GPI)(B2GPI)(Apolipoprotein H)(Apo-H)(Activated protein C-binding protein)(APC inhibitor)(Anticardiolipin cofactor) [Source: Ur
4134.1.1	-3.1	5.15	2273.1755	-0.0055	2	46.54	gmrk	64	FICPLTGLWPINTLK	78	ctpr	C [66] 500.199, C [66] 57.0215	ENSP00000205948	Beta-2-glycoprotein 1 Precursor (Beta-2-glycoprotein I)(Beta(2)GPI)(B2GPI)(Apolipoprotein H)(Apo-H)(Activated protein C-binding protein)(APC inhibitor)(Anticardiolipin cofactor) [Source: Ur
4135.1.1	-4.9	5.19	2273.1755	-0.0039	3	46.54	gmrk	64	FICPLTGLWPINTLK	78	ctpr	C [66] 500.199, C [66] 57.0215	ENSP00000205948	Beta-2-glycoprotein 1 Precursor (Beta-2-glycoprotein I)(Beta(2)GPI)(B2GPI)(Apolipoprotein H)(Apo-H)(Activated protein C-binding protein)(APC inhibitor)(Anticardiolipin cofactor) [Source: Ur
4137.1.1	-11.6	4.46	3506.7157	-0.0025	3	48.28	dsak	124	CTEEGKWSPELPVCAPIICPPPSIP	154	vykp	C [124] 57.0215, C [137] 57.0215, C [142] 5	ENSP00000205948	Beta-2-glycoprotein 1 Precursor (Beta-2-glycoprotein I)(Beta(2)GPI)(B2GPI)(Apolipoprotein H)(Apo-H)(Activated protein C-binding protein)(APC inhibitor)(Anticardiolipin cofactor) [Source: Ur
4141.1.1	-3.1	5.29	2273.1755	-0.0026	2	46.54	gmrk	64	FICPLTGLWPINTLK	78	ctpr	C [66] 500.199, C [66] 57.0215	ENSP00000205948	Beta-2-glycoprotein 1 Precursor (Beta-2-glycoprotein I)(Beta(2)GPI)(B2GPI)(Apolipoprotein H)(Apo-H)(Activated protein C-binding protein)(APC inhibitor)(Anticardiolipin cofactor) [Source: Ur
4145.1.1	-4.4	3.88	1788.9717	0.0064	2	46.54	gmrk	64	FICPLTGLWPINTLK	78	ctpr	C [66] 57.0215, W [72] 15.9949	ENSP00000205948	Beta-2-glycoprotein 1 Precursor (Beta-2-glycoprotein I)(Beta(2)GPI)(B2GPI)(Apolipoprotein H)(Apo-H)(Activated protein C-binding protein)(APC inhibitor)(Anticardiolipin cofactor) [Source: Ur
4147.1.1	-2.9	5.28	2273.1755	-0.0062	2	46.54	gmrk	64	FICPLTGLWPINTLK	78	ctpr	C [66] 500.199, C [66] 57.0215	ENSP00000205948	Beta-2-glycoprotein 1 Precursor (Beta-2-glycoprotein I)(Beta(2)GPI)(B2GPI)(Apolipoprotein H)(Apo-H)(Activated protein C-binding protein)(APC inhibitor)(Anticardiolipin cofactor) [Source: Ur
4148.1.1	-4.1	5.1	2273.1755	-0.0066	3	46.54	gmrk	64	FICPLTGLWPINTLK	78	ctpr	C [66] 500.199, C [66] 57.0215	ENSP00000205948	Beta-2-glycoprotein 1 Precursor (Beta-2-glycoprotein I)(Beta(2)GPI)(B2GPI)(Apolipoprotein H)(Apo-H)(Activated protein C-binding protein)(APC inhibitor)(Anticardiolipin cofactor) [Source: Ur
4150.1.1	-3.3	4.1	1788.9717	0.0033	2	46.54	gmrk	64	FICPLTGLWPINTLK	78	ctpr	C [66] 57.0215, W [72] 15.9949	ENSP00000205948	Beta-2-glycoprotein 1 Precursor (Beta-2-glycoprotein I)(Beta(2)GPI)(B2GPI)(Apolipoprotein H)(Apo-H)(Activated protein C-binding protein)(APC inhibitor)(Anticardiolipin cofactor) [Source: Ur
4153.1.1	-7.1	4.14	1788.9717	0.0028	2	46.54	gmrk	64	FICPLTGLWPINTLK	78	ctpr	C [66] 57.0215, W [72] 15.9949	ENSP00000205948	Beta-2-glycoprotein 1 Precursor (Beta-2-glycoprotein I)(Beta(2)GPI)(B2GPI)(Apolipoprotein H)(Apo-H)(Activated protein C-binding protein)(APC inhibitor)(Anticardiolipin cofactor) [Source: Ur
4160.1.1	-4	4.4	1313.6862	-0.0014	2	26.92	reqr	111	AGWSRAGPAWVR	122	riwr		ENSP00000381377	
4170.1.1	-6	4.22	1772.9768	0.0007	2	46.54	gmrk	64	FICPLTGLWPINTLK	78	ctpr	C [66] 57.0215	ENSP00000205948	Beta-2-glycoprotein 1 Precursor (Beta-2-glycoprotein I)(Beta(2)GPI)(B2GPI)(Apolipoprotein H)(Apo-H)(Activated protein C-binding protein)(APC inhibitor)(Anticardiolipin cofactor) [Source: Ur
4174.1.1	-6.6	4.47	1772.9768	0.0012	2	46.54	gmrk	64	FICPLTGLWPINTLK	78	ctpr	C [66] 57.0215	ENSP00000205948	Beta-2-glycoprotein 1 Precursor (Beta-2-glycoprotein I)(Beta(2)GPI)(B2GPI)(Apolipoprotein H)(Apo-H)(Activated protein C-binding protein)(APC inhibitor)(Anticardiolipin cofactor) [Source: Ur
4177.1.1	-9.1	4.61	1772.9768	0.0003	2	46.54	gmrk	64	FICPLTGLWPINTLK	78	ctpr	C [66] 57.0215	ENSP00000205948	Beta-2-glycoprotein 1 Precursor (Beta-2-glycoprotein I)(Beta(2)GPI)(B2GPI)(Apolipoprotein H)(Apo-H)(Activated protein C-binding protein)(APC inhibitor)(Anticardiolipin cofactor) [Source: Ur
4217.1.1	-5.6	4.44	1772.9768	-0.0008	3	46.54	gmrk	64	FICPLTGLWPINTLK	78	ctpr	C [66] 57.0215	ENSP00000205948	Beta-2-glycoprotein 1 Precursor (Beta-2-glycoprotein I)(Beta(2)GPI)(B2GPI)(Apolipoprotein H)(Apo-H)(Activated protein C-binding protein)(APC inhibitor)(Anticardiolipin cofactor) [Source: Ur
4221.1.1	-2.2	4.05	1788.9717	0.002	2	46.54	gmrk	64	FICPLTGLWPINTLK	78	ctpr	C [66] 57.0215, W [72] 15.9949	ENSP00000205948	Beta-2-glycoprotein 1 Precursor (Beta-2-glycoprotein I)(Beta(2)GPI)(B2GPI)(Apolipoprotein H)(Apo-H)(Activated protein C-binding protein)(APC inhibitor)(Anticardiolipin cofactor) [Source: Ur
4223.1.1	-3.4	4.43	1772.9768	-0.0002	3	46.54	gmrk	64	FICPLTGLWPINTLK	78	ctpr	C [66] 57.0215	ENSP00000205948	Beta-2-glycoprotein 1 Precursor (Beta-2-glycoprotein I)(Beta(2)GPI)(B2GPI)(Apolipoprotein H)(Apo-H)(Activated protein C-binding protein)(APC inhibitor)(Anticardiolipin cofactor) [Source: Ur
4229.1.1	-1.7	3.78	2273.1755	-0.0053	2	46.54	gmrk	64	FICPLTGLWPINTLK	78	ctpr	C [66] 500.199, C [66] 57.0215	ENSP00000205948	Beta-2-glycoprotein 1 Precursor (Beta-2-glycoprotein I)(Beta(2)GPI)(B2GPI)(Apolipoprotein H)(Apo-H)(Activated protein C-binding protein)(APC inhibitor)(Anticardiolipin cofactor) [Source: Ur
4236.1.1	-5.5	5.5	1772.9768	0.0024	2	46.54	gmrk	64	FICPLTGLWPINTLK	78	ctpr	C [66] 57.0215	ENSP00000205948	Beta-2-glycoprotein 1 Precursor (Beta-2-glycoprotein I)(Beta(2)GPI)(B2GPI)(Apolipoprotein H)(Apo-H)(Activated protein C-binding protein)(APC inhibitor)(Anticardiolipin cofactor) [Source: Ur
4237.1.1	-1.1	3.86	1556.8002	-0.0096	2	33.82	naek	29	VPCSNLGALGWLNR	42	dlrk	C [31] 57.0215	ENSP00000231449:reversed	
4241.1.1	-5.5	5.4	1772.9768	0.0002	2	46.54	gmrk	64	FICPLTGLWPINTLK	78	ctpr	C [66] 57.0215	ENSP00000205948	Beta-2-glycoprotein 1 Precursor (Beta-2-glycoprotein I)(Beta(2)GPI)(B2GPI)(Apolipoprotein H)(Apo-H)(Activated protein C-binding protein)(APC inhibitor)(Anticardiolipin cofactor) [Source: Ur
4243.1.1	-7.7	5.34	1772.9768	-0.0008	2	46.54	gmrk	64	FICPLTGLWPINTLK	78	ctpr	C [66] 57.0215	ENSP00000205948	Beta-2-glycoprotein 1 Precursor (Beta-2-glycoprotein I)(Beta(2)GPI)(B2GPI)(Apolipoprotein H)(Apo-H)(Activated protein C-binding protein)(APC inhibitor)(Anticardiolipin cofactor) [Source: Ur
4244.1.1	-2	3.95	1556.7888	-0.0038	2	3.1	lpvr	27	AATASRGASQAGAPQGR	43	vpea		ENSP00000362924	Gelsolin precursor (Actin-depolymerizing factor) (ADF) (Brevin) (AGEL). Source: Uniprot/SWISSPROT P06396
4297.1.1	-6.9	4.31	1772.9768	0.0002	2	46.54	gmrk	64	FICPLTGLWPINTLK	78	ctpr	C [66] 57.0215	ENSP00000205948	Beta-2-glycoprotein 1 Precursor (Beta-2-glycoprotein I)(Beta(2)GPI)(B2GPI)(Apolipoprotein H)(Apo-H)(Activated protein C-binding protein)(APC inhibitor)(Anticardiolipin cofactor) [Source: Ur
4300.1.1	-4.6	4.21	1772.9768	-0.001	2	46.54	gmrk	64	FICPLTGLWPINTLK	78	ctpr	C [66] 57.0215	ENSP00000205948	Beta-2-glycoprotein 1 Precursor (Beta-2-glycoprotein I)(Beta(2)GPI)(B2GPI)(Apolipoprotein H)(Apo-H)(Activated protein C-binding protein)(APC inhibitor)(Anticardiolipin cofactor) [Source: Ur
4303.1.1	-8	4.15	1772.9768	-0.0009	2	46.54	gmrk	64	FICPLTGLWPINTLK	78	ctpr	C [66] 57.0215	ENSP00000205948	Beta-2-glycoprotein 1 Precursor (Beta-2-glycoprotein I)(Beta(2)GPI)(B2GPI)(Apolipoprotein H)(Apo-H)(Activated protein C-binding protein)(APC inhibitor)(Anticardiolipin cofactor) [Source: Ur
4348.1.1	-6.2	3.72	1772.9768	-0.0022	2	46.54	gmrk	64	FICPLTGLWPINTLK	78	ctpr	C [66] 57.0215	ENSP00000205948	Beta-2-glycoprotein 1 Precursor (Beta-2-glycoprotein I)(Beta(2)GPI)(B2GPI)(Apolipoprotein H)(Apo-H)(Activated protein C-binding protein)(APC inhibitor)(Anticardiolipin cofactor) [Source: Ur
4351.1.1	-2.3	3.66	1772.9768	-0.0065	2	46.54	gmrk	64	FICPLTGLWPINTLK	78	ctpr	C [66] 57.0215	ENSP00000205948	Beta-2-glycoprotein 1 Precursor (Beta-2-glycoprotein I)(Beta(2)GPI)(B2GPI)(Apolipoprotein H)(Apo-H)(Activated protein C-binding protein)(APC inhibitor)(Anticardiolipin cofactor) [Source: Ur
4353.1.1	-5.1	3.63	1772.9768	0.0023	2	46.54	gmrk	64	FICPLTGLWPINTLK	78	ctpr	C [66] 57.0215	ENSP00000205948	Beta-2-glycoprotein 1 Precursor (Beta-2-glycoprotein I)(Beta(2)GPI)(B2GPI)(Apolipoprotein H)(Apo-H)(Activated protein C-binding protein)(APC inhibitor)(Anticardiolipin cofactor) [Source: Ur
4392.1.1	-2.6	3.58	1772.9768	-0.0032	2	46.54	gmrk	64	FICPLTGLWPINTLK	78	ctpr	C [66] 57.0215	ENSP00000205948	Beta-2-glycoprotein 1 Precursor (Beta-2-glycoprotein I)(Beta(2)GPI)(B2GPI)(Apolipoprotein H)(Apo-H)(Activated protein C-binding protein)(APC inhibitor)(Anticardiolipin cofactor) [Source: Ur
4395.1.1	-1.9	3.6	1772.9768	0.0029	2	46.54	gmrk	64	FICPLTGLWPINTLK	78	ctpr	C [66] 57.0215	ENSP00000205948	Beta-2-glycoprotein 1 Precursor (Beta-2-glycoprotein I)(Beta(2)GPI)(B2GPI)(Apolipoprotein H)(Apo-H)(Activated protein C-binding protein)(APC inhibitor)(Anticardiolipin cofactor) [Source: Ur
4407.1.1	-1.6	3.51	1772.9768	0.0058	2	46.54	gmrk	64	FICPLTGLWPINTLK	78	ctpr	C [66] 57.0215	ENSP00000205948	Beta-2-glycoprotein 1 Precursor (Beta-2-glycoprotein I)(Beta(2)GPI)(B2GPI)(Apolipoprotein H)(Apo-H)(Activated protein C-binding protein)(APC inhibitor)(Anticardiolipin cofactor) [Source: Ur
