# Supplementary material for: Redox control of β2-glycoprotein I–von Willebrand factor interaction by thioredoxin-1
Source: J Thromb Haemost. 2010 Aug;8(8):1754–62. doi: 10.1111/j.1538-7836.2010.03944.x (PMC3017748; doi:10.1111/j.1538-7836.2010.03944.x)
Supplement: Supplementary file 3 [file jth0008-1754-SD3.pdf]

SI Table 2a b2gpI

Mascot Search Results

```
User          :
Email         :
Search title  : Freda_13_11_08_1.RAW relex2
MS data file  : D:\Data\Mark\13_11_08\Freda_13_11_08_1.RAW
Database      : Sprot 19_12_08 (402482 sequences; 145232059 residues)
Timestamp     : 11 Mar 2009 at 03:49:25 GMT
Protein hits  : APOH_HUMAN  Beta-2-glycoprotein 1 OS=Homo sapiens GN=APOH PE=1 SV=3
                APOH_CANFA  Beta-2-glycoprotein 1 OS=Canis familiaris GN=APOH PE=2 SV=1
                K2C1_PANTR  Keratin, type II cytoskeletal 1 OS=Pan troglodytes GN=KRT1 PE=2 SV=1
                THIO_ECOLI  Thioredoxin-1 OS=Escherichia coli (strain K12) GN=trxA PE=1 SV=2
                TRYP_PIG    Trypsin OS=Sus scrofa PE=1 SV=1
                THIO_HUMAN  Thioredoxin OS=Homo sapiens GN=TXN PE=1 SV=3
                CASB_BOVIN  Beta-casein OS=Bos taurus GN=CSN2 PE=1 SV=2
                CASA1_BOVIN Alpha-S1-casein OS=Bos taurus GN=CSN1S1 PE=1 SV=2
```

Probability Based Mowse Score

Ions score is -10*Log(P), where P is the probability that the observed match is a random event.
Individual ions scores > 35 indicate identity or extensive homology (p<0.05).
Protein scores are derived from ions scores as a non-probabilistic basis for ranking protein hits.

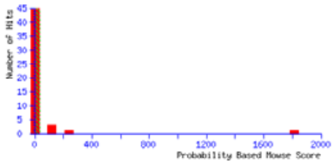

Peptide Summary Report

Format As [Peptide Summary] Help
Significance threshold p< [0.05] Max. number of hits [AUTO]
Standard scoring ○ MudPIT scoring ● Ions score or expect cut-off [1] Show sub-sets [0]
Show pop-ups ● Suppress pop-ups ○ Sort unassigned [Decreasing Score] Require bold red ☑

Select All | Select None | Search Selected | ☐ **Error tolerant** | Archive Report

1. APOH_HUMAN **Mass:** 38273 **Score:** 1811 **Queries matched:** 165 **emPAI:** 24.37
Beta-2-glycoprotein 1 OS=Homo sapiens GN=APOH PE=1 SV=3
☐ Check to include this hit in error tolerant search or archive report

	Query	Observed	Mr(expt)	Mr(calc)	ppm	Miss	Score	Expect	Rank	Peptide
☑	13	394.1942	786.3738	786.3735	0.49	0	(10)	3.4	1	K.VSFFCK.N + Carbamidomethyl (C)
☑	14	394.1943	786.3740	786.3735	0.75	0	25	0.1	1	K.VSFFCK.N + Carbamidomethyl (C)
	45	451.7606	901.5066	901.5055	1.26	1	21	1.2	2	K.ASCKVPVK.K + Acrylamide (C)
☑	72	496.7219	991.4292	991.4281	1.19	0	65	9.2e-006	1	K.TDASDVKPC.- + Carbamidomethyl (C)
☑	73	496.7219	991.4292	991.4281	1.19	0	(56)	6.7e-005	1	K.TDASDVKPC.- + Carbamidomethyl (C)
☑	74	497.2137	992.4128	992.4121	0.77	0	(55)	0.0002	1	K.TDASDVKPC.- + Carboxymethyl (C)
☑	89	511.7672	1021.5198	1021.5193	0.58	0	(21)	0.78	1	K.ATVVYQGER.V
☑	90	511.7673	1021.5200	1021.5193	0.77	0	(25)	0.32	1	K.ATVVYQGER.V
☑	91	511.7674	1021.5202	1021.5193	0.97	0	(58)	0.00015	1	K.ATVVYQGER.V
☑	92	511.7674	1021.5202	1021.5193	0.97	0	(47)	0.0021	1	K.ATVVYQGER.V

	Query	Observed	Mr(expt)	Mr(calc)	ppm	Miss	Score	Expect	Rank	Peptide
☑	93	511.7674	1021.5202	1021.5193	0.97	0	(42)	0.0058	1	K.ATVVYQGER.V
☑	94	511.7675	1021.5204	1021.5193	1.17	0	(34)	0.041	1	K.ATVVYQGER.V
☑	95	511.7675	1021.5204	1021.5193	1.17	0	(40)	0.01	1	K.ATVVYQGER.V
☑	96	511.7675	1021.5204	1021.5193	1.17	0	(58)	0.00016	1	K.ATVVYQGER.V
☑	97	511.7676	1021.5206	1021.5193	1.36	0	(45)	0.0039	1	K.ATVVYQGER.V
☑	98	511.7676	1021.5206	1021.5193	1.36	0	62	8.4e-005	1	K.ATVVYQGER.V
☑	99	511.7676	1021.5206	1021.5193	1.36	0	(51)	0.00087	1	K.ATVVYQGER.V
☑	100	511.7676	1021.5206	1021.5193	1.36	0	(60)	0.00012	1	K.ATVVYQGER.V
☑	157	368.8542	1103.5408	1103.5400	0.72	0	(16)	4.2	1	K.EHSSLAFWK.T
☑	158	368.8543	1103.5411	1103.5400	0.99	0	(27)	0.3	1	K.EHSSLAFWK.T
☑	159	368.8543	1103.5411	1103.5400	0.99	0	(17)	3.3	1	K.EHSSLAFWK.T
☑	160	552.7779	1103.5412	1103.5400	1.15	0	29	0.18	1	K.EHSSLAFWK.T
☑	161	552.7781	1103.5416	1103.5400	1.51	0	(3)	80	1	K.EHSSLAFWK.T
☑	162	368.8546	1103.5420	1103.5400	1.81	0	(16)	3.5	1	K.EHSSLAFWK.T
☑	194	384.2124	1149.6154	1149.6142	1.01	1	(21)	0.84	1	K.KATVVYQGER.V
☑	195	384.2124	1149.6154	1149.6142	1.01	1	(46)	0.0027	1	K.KATVVYQGER.V
☑	196	575.8150	1149.6154	1149.6142	1.08	1	(37)	0.022	1	K.KATVVYQGER.V
☑	197	575.8154	1149.6162	1149.6142	1.77	1	68	1.5e-005	1	K.KATVVYQGER.V
☑	198	575.8154	1149.6162	1149.6142	1.77	1	(49)	0.0013	1	K.KATVVYQGER.V
☑	204	388.1943	1161.5611	1161.5601	0.86	1	(21)	0.92	1	K.FKNGMLHGDK.V + Oxidation (M)
☑	205	581.7884	1161.5622	1161.5601	1.87	1	36	0.028	1	K.FKNGMLHGDK.V + Oxidation (M)
	251	417.2351	1248.6835	1248.6826	0.67	1	(3)	55	6	K.ATVVYQGERVK.I
☑	252	625.3496	1248.6846	1248.6826	1.61	1	16	2.3	1	K.ATVVYQGERVK.I
☑	307	501.5980	1501.7722	1501.7711	0.69	0	(31)	0.13	1	R.VCPFAGILENGAVR.Y + Carbamidomethyl (C)
☑	308	501.5983	1501.7731	1501.7711	1.29	0	(40)	0.013	1	R.VCPFAGILENGAVR.Y + Carbamidomethyl (C)
☑	309	751.8940	1501.7734	1501.7711	1.54	0	65	5.5e-005	1	R.VCPFAGILENGAVR.Y + Carbamidomethyl (C)
☑	310	751.8945	1501.7744	1501.7711	2.21	0	(25)	0.47	1	R.VCPFAGILENGAVR.Y + Carbamidomethyl (C)
☑	311	751.8950	1501.7754	1501.7711	2.87	0	(48)	0.0026	1	R.VCPFAGILENGAVR.Y + Carbamidomethyl (C)
☑	312	751.8951	1501.7756	1501.7711	3.01	0	(34)	0.065	1	R.VCPFAGILENGAVR.Y + Carbamidomethyl (C)
☑	313	751.8952	1501.7758	1501.7711	3.14	0	(20)	1.5	1	R.VCPFAGILENGAVR.Y + Carbamidomethyl (C)
☑	314	751.8959	1501.7772	1501.7711	4.07	0	(36)	0.037	1	R.VCPFAGILENGAVR.Y + Carbamidomethyl (C)
☑	315	752.3849	1502.7552	1502.7551	0.07	0	(22)	1.1	1	R.VCPFAGILENGAVR.Y + Carboxymethyl (C)
☑	316	501.9263	1502.7571	1502.7551	1.28	0	(24)	0.56	1	R.VCPFAGILENGAVR.Y + Carboxymethyl (C)
☑	318	752.3862	1502.7578	1502.7551	1.80	0	(28)	0.25	1	R.VCPFAGILENGAVR.Y + Carboxymethyl (C)
☑	319	752.3862	1502.7578	1502.7551	1.80	0	(19)	2	1	R.VCPFAGILENGAVR.Y + Carboxymethyl (C)
☑	320	752.3864	1502.7582	1502.7551	2.07	0	(18)	2.8	1	R.VCPFAGILENGAVR.Y + Carboxymethyl (C)
☑	321	752.3864	1502.7582	1502.7551	2.07	0	(35)	0.055	1	R.VCPFAGILENGAVR.Y + Carboxymethyl (C)
☑	322	752.3865	1502.7584	1502.7551	2.20	0	(39)	0.023	1	R.VCPFAGILENGAVR.Y + Carboxymethyl (C)
☑	323	752.3865	1502.7584	1502.7551	2.20	0	(19)	1.9	1	R.VCPFAGILENGAVR.Y + Carboxymethyl (C)
☑	324	752.3866	1502.7586	1502.7551	2.33	0	(16)	4.1	1	R.VCPFAGILENGAVR.Y + Carboxymethyl (C)
☑	325	752.3867	1502.7588	1502.7551	2.47	0	(44)	0.0059	1	R.VCPFAGILENGAVR.Y + Carboxymethyl (C)
☑	326	752.3870	1502.7594	1502.7551	2.87	0	(27)	0.37	1	R.VCPFAGILENGAVR.Y + Carboxymethyl (C)
☑	327	752.3874	1502.7602	1502.7551	3.40	0	(22)	0.91	1	R.VCPFAGILENGAVR.Y + Carboxymethyl (C)
	368	385.6911	1538.7353	1538.7340	0.84	1	(2)	74	4	K.CFKEHSSLAFWK.T + Carbamidomethyl (C)
☑	369	513.9191	1538.7355	1538.7340	0.95	1	(18)	2.3	1	K.CFKEHSSLAFWK.T + Carbamidomethyl (C)
☑	370	513.9193	1538.7361	1538.7340	1.34	1	(22)	0.77	1	K.CFKEHSSLAFWK.T + Carbamidomethyl (C)
☑	371	770.3756	1538.7366	1538.7340	1.72	1	37	0.027	1	K.CFKEHSSLAFWK.T + Carbamidomethyl (C)
☑	374	514.2474	1539.7204	1539.7180	1.53	1	(15)	4.4	1	K.CFKEHSSLAFWK.T + Carboxymethyl (C)
	428	410.6986	1638.7653	1638.7647	0.38	1	(3)	86	2	K.NGMLHGDKVSFFCK.N + Carbamidomethyl (C)
☑	429	547.2628	1638.7666	1638.7647	1.16	1	(36)	0.037	1	K.NGMLHGDKVSFFCK.N + Carbamidomethyl (C)
☑	430	547.5908	1639.7506	1639.7487	1.15	1	(16)	4.2	1	K.NGMLHGDKVSFFCK.N + Carboxymethyl (C)
☑	438	552.5944	1654.7614	1654.7596	1.07	1	(31)	0.11	1	K.NGMLHGDKVSFFCK.N + Carbamidomethyl (C); Oxidation (M)
☑	440	552.5949	1654.7629	1654.7596	1.98	1	(26)	0.35	1	K.NGMLHGDKVSFFCK.N + Carbamidomethyl (C); Oxidation (M)
☑	441	828.3890	1654.7634	1654.7596	2.33	1	46	0.0038	1	K.NGMLHGDKVSFFCK.N + Carbamidomethyl (C); Oxidation (M)
	444	552.9225	1655.7457	1655.7436	1.25	1	(10)	13	2	K.NGMLHGDKVSFFCK.N + Carboxymethyl (C); Oxidation (M)
☑	446	828.8810	1655.7474	1655.7436	2.32	1	(13)	6	1	K.NGMLHGDKVSFFCK.N + Carboxymethyl (C); Oxidation (M)
☑	492	591.6650	1771.9732	1771.9695	2.07	0	(30)	0.13	1	K.FICPLTGLWPINTLK.C + Carbamidomethyl (C)
☑	493	591.6650	1771.9732	1771.9695	2.07	0	(34)	0.043	1	K.FICPLTGLWPINTLK.C + Carbamidomethyl (C)
☑	494	886.9943	1771.9740	1771.9695	2.57	0	(63)	5.8e-005	1	K.FICPLTGLWPINTLK.C + Carbamidomethyl (C)

☑	495	886.9944	1771.9742	1771.9695	2.68	0	72	8.2e-006	1	K.FICPLTGLWPINTLK.C + Carbamidomethyl (C)
☑	496	886.9944	1771.9742	1771.9695	2.68	0	(51)	0.00094	1	K.FICPLTGLWPINTLK.C + Carbamidomethyl (C)
☑	497	886.9945	1771.9744	1771.9695	2.79	0	(70)	1.3e-005	1	K.FICPLTGLWPINTLK.C + Carbamidomethyl (C)
☑	498	886.9948	1771.9750	1771.9695	3.13	0	(55)	0.00043	1	K.FICPLTGLWPINTLK.C + Carbamidomethyl (C)
☑	554	634.3632	1900.0678	1900.0645	1.75	1	(21)	0.59	1	R.KFICPLTGLWPINTLK.C + Carbamidomethyl (C)
☑	555	951.0416	1900.0686	1900.0645	2.21	1	43	0.0034	1	R.KFICPLTGLWPINTLK.C + Carbamidomethyl (C)
☑	556	634.3635	1900.0687	1900.0645	2.22	1	(24)	0.27	1	R.KFICPLTGLWPINTLK.C + Carbamidomethyl (C)
☑	557	634.3635	1900.0687	1900.0645	2.22	1	(29)	0.09	1	R.KFICPLTGLWPINTLK.C + Carbamidomethyl (C)
☑	558	951.0420	1900.0694	1900.0645	2.63	1	(41)	0.0058	1	R.KFICPLTGLWPINTLK.C + Carbamidomethyl (C)
☑	559	634.3641	1900.0705	1900.0645	3.17	1	(16)	1.8	1	R.KFICPLTGLWPINTLK.C + Carbamidomethyl (C)
☑	569	638.6727	1912.9963	1912.9969	-0.31	0	(20)	1.5	1	R.TCPKPDDLPFSTVVPLK.T + Carbamidomethyl (C)
☑	570	638.6733	1912.9981	1912.9969	0.64	0	(30)	0.16	1	R.TCPKPDDLPFSTVVPLK.T + Carbamidomethyl (C)
☑	571	638.6736	1912.9990	1912.9969	1.11	0	(37)	0.03	1	R.TCPKPDDLPFSTVVPLK.T + Carbamidomethyl (C)
☑	572	638.6738	1912.9996	1912.9969	1.42	0	(32)	0.098	1	R.TCPKPDDLPFSTVVPLK.T + Carbamidomethyl (C)
☑	573	638.6738	1912.9996	1912.9969	1.42	0	(21)	1.4	1	R.TCPKPDDLPFSTVVPLK.T + Carbamidomethyl (C)
☑	574	638.6739	1912.9999	1912.9969	1.58	0	(17)	3.5	1	R.TCPKPDDLPFSTVVPLK.T + Carbamidomethyl (C)
☑	575	638.6740	1913.0002	1912.9969	1.73	0	(41)	0.013	1	R.TCPKPDDLPFSTVVPLK.T + Carbamidomethyl (C)
☑	576	638.6740	1913.0002	1912.9969	1.73	0	(15)	5.2	1	R.TCPKPDDLPFSTVVPLK.T + Carbamidomethyl (C)
☑	577	638.6740	1913.0002	1912.9969	1.73	0	(32)	0.1	1	R.TCPKPDDLPFSTVVPLK.T + Carbamidomethyl (C)
☑	578	638.6740	1913.0002	1912.9969	1.73	0	(13)	9.4	1	R.TCPKPDDLPFSTVVPLK.T + Carbamidomethyl (C)
☑	579	638.6741	1913.0005	1912.9969	1.89	0	(38)	0.03	1	R.TCPKPDDLPFSTVVPLK.T + Carbamidomethyl (C)
☑	580	638.6741	1913.0005	1912.9969	1.89	0	(34)	0.07	1	R.TCPKPDDLPFSTVVPLK.T + Carbamidomethyl (C)
☑	581	957.5077	1913.0008	1912.9969	2.09	0	(25)	0.62	1	R.TCPKPDDLPFSTVVPLK.T + Carbamidomethyl (C)
☑	582	957.5078	1913.0010	1912.9969	2.19	0	(13)	8.1	1	R.TCPKPDDLPFSTVVPLK.T + Carbamidomethyl (C)
☑	583	638.6743	1913.0011	1912.9969	2.20	0	(27)	0.35	1	R.TCPKPDDLPFSTVVPLK.T + Carbamidomethyl (C)
☑	584	638.6743	1913.0011	1912.9969	2.20	0	(25)	0.53	1	R.TCPKPDDLPFSTVVPLK.T + Carbamidomethyl (C)
☑	585	638.6743	1913.0011	1912.9969	2.20	0	(46)	0.0046	1	R.TCPKPDDLPFSTVVPLK.T + Carbamidomethyl (C)
☑	586	638.6744	1913.0014	1912.9969	2.36	0	(21)	1.3	1	R.TCPKPDDLPFSTVVPLK.T + Carbamidomethyl (C)
☑	587	638.6744	1913.0014	1912.9969	2.36	0	(15)	5.2	1	R.TCPKPDDLPFSTVVPLK.T + Carbamidomethyl (C)
☑	588	638.6745	1913.0017	1912.9969	2.52	0	(23)	0.86	1	R.TCPKPDDLPFSTVVPLK.T + Carbamidomethyl (C)
☑	589	638.6745	1913.0017	1912.9969	2.52	0	(24)	0.62	1	R.TCPKPDDLPFSTVVPLK.T + Carbamidomethyl (C)
☑	590	957.5084	1913.0022	1912.9969	2.82	0	46	0.004	1	R.TCPKPDDLPFSTVVPLK.T + Carbamidomethyl (C)
☑	591	638.6747	1913.0023	1912.9969	2.83	0	(20)	1.7	1	R.TCPKPDDLPFSTVVPLK.T + Carbamidomethyl (C)
☑	592	638.6749	1913.0029	1912.9969	3.14	0	(33)	0.087	1	R.TCPKPDDLPFSTVVPLK.T + Carbamidomethyl (C)
☑	599	644.3156	1929.9250	1929.9230	1.04	2	(15)	6.3	1	K.FKNGMLHGDKVSFFCK.N + Carbamidomethyl (C); Oxidation (M)
☑	600	483.4886	1929.9253	1929.9230	1.20	2	18	3.4	1	K.FKNGMLHGDKVSFFCK.N + Carbamidomethyl (C); Oxidation (M)
	603	966.4617	1930.9088	1930.9070	0.97	2	(7)	45	2	K.FKNGMLHGDKVSFFCK.N + Carboxymethyl (C); Oxidation (M)
☑	682	695.9762	2084.9068	2084.9031	1.77	0	(46)	0.0042	1	K.CSYTEDAQCIDGTIEVPK.C + 2 Carbamidomethyl (C)
☑	683	1043.4610	2084.9074	2084.9031	2.10	0	(58)	0.00031	1	K.CSYTEDAQCIDGTIEVPK.C + 2 Carbamidomethyl (C)
☑	684	1043.4620	2084.9094	2084.9031	3.06	0	(95)	5.7e-008	1	K.CSYTEDAQCIDGTIEVPK.C + 2 Carbamidomethyl (C)
☑	685	1043.4620	2084.9094	2084.9031	3.06	0	107	4.2e-009	1	K.CSYTEDAQCIDGTIEVPK.C + 2 Carbamidomethyl (C)
☑	686	1043.4620	2084.9094	2084.9031	3.06	0	(66)	4.3e-005	1	K.CSYTEDAQCIDGTIEVPK.C + 2 Carbamidomethyl (C)
☑	687	1043.4630	2084.9114	2084.9031	4.02	0	(59)	0.00022	1	K.CSYTEDAQCIDGTIEVPK.C + 2 Carbamidomethyl (C)
☑	689	1043.9550	2085.8954	2085.8871	4.01	0	(77)	3.7e-006	1	K.CSYTEDAQCIDGTIEVPK.C + Carbamidomethyl (C); Carboxymethyl (C)
☑	741	1107.5070	2212.9994	2212.9980	0.64	1	(40)	0.023	1	K.KCSYTEDAQCIDGTIEVPK.C + 2 Carbamidomethyl (C)
	742	738.6750	2213.0032	2212.9980	2.32	1	(4)	88	3	K.KCSYTEDAQCIDGTIEVPK.C + 2 Carbamidomethyl (C)
☑	743	1107.5090	2213.0034	2212.9980	2.45	1	78	4.1e-006	1	K.KCSYTEDAQCIDGTIEVPK.C + 2 Carbamidomethyl (C)
☑	744	738.6752	2213.0038	2212.9980	2.59	1	(38)	0.037	1	K.KCSYTEDAQCIDGTIEVPK.C + 2 Carbamidomethyl (C)
☑	745	738.6754	2213.0044	2212.9980	2.86	1	(35)	0.079	1	K.KCSYTEDAQCIDGTIEVPK.C + 2 Carbamidomethyl (C)
	761	763.0760	2286.2062	2286.2017	1.95	1	2	1.3e+002	2	K.FICPLTGLWPINTLKCTPR.V + 2 Carbamidomethyl (C)
☑	772	795.0341	2382.0805	2382.0838	-1.40	0	(4)	1.1e+002	1	K.TFYEPGEEITYSCKPGYVSR.G + Carbamidomethyl (C)
☑	774	795.0369	2382.0889	2382.0838	2.13	0	(8)	41	1	K.TFYEPGEEITYSCKPGYVSR.G + Carbamidomethyl (C)
☑	775	795.0369	2382.0889	2382.0838	2.13	0	(14)	12	1	K.TFYEPGEEITYSCKPGYVSR.G + Carbamidomethyl (C)
☑	776	795.0370	2382.0892	2382.0838	2.25	0	(18)	4	1	K.TFYEPGEEITYSCKPGYVSR.G + Carbamidomethyl (C)
☑	777	1192.0520	2382.0894	2382.0838	2.37	0	71	2.4e-005	1	K.TFYEPGEEITYSCKPGYVSR.G + Carbamidomethyl (C)
☑	778	795.0372	2382.0898	2382.0838	2.51	0	(7)	56	1	K.TFYEPGEEITYSCKPGYVSR.G + Carbamidomethyl (C)
☑	779	795.0372	2382.0898	2382.0838	2.51	0	(5)	79	1	K.TFYEPGEEITYSCKPGYVSR.G + Carbamidomethyl (C)
☑	780	795.0372	2382.0898	2382.0838	2.51	0	(13)	13	1	K.TFYEPGEEITYSCKPGYVSR.G + Carbamidomethyl (C)
☑	781	795.0372	2382.0898	2382.0838	2.51	0	(22)	1.6	1	K.TFYEPGEEITYSCKPGYVSR.G + Carbamidomethyl (C)

☑	782	795.0373	2382.0901	2382.0838	2.63	0	(9)	31	1	K.TFYEPGEEITYSCKPGYVSR.G + Carbamidomethyl (C)
☑	783	795.0373	2382.0901	2382.0838	2.63	0	(2)	1.7e+002	1	K.TFYEPGEEITYSCKPGYVSR.G + Carbamidomethyl (C)
☑	784	795.0374	2382.0904	2382.0838	2.76	0	(11)	24	1	K.TFYEPGEEITYSCKPGYVSR.G + Carbamidomethyl (C)
☑	785	795.0374	2382.0904	2382.0838	2.76	0	(6)	64	1	K.TFYEPGEEITYSCKPGYVSR.G + Carbamidomethyl (C)
☑	786	795.0375	2382.0907	2382.0838	2.88	0	(3)	1.5e+002	1	K.TFYEPGEEITYSCKPGYVSR.G + Carbamidomethyl (C)
☑	787	795.0375	2382.0907	2382.0838	2.88	0	(8)	47	1	K.TFYEPGEEITYSCKPGYVSR.G + Carbamidomethyl (C)
☑	788	795.0375	2382.0907	2382.0838	2.88	0	(4)	1e+002	1	K.TFYEPGEEITYSCKPGYVSR.G + Carbamidomethyl (C)
☑	789	795.0377	2382.0913	2382.0838	3.14	0	(13)	12	1	K.TFYEPGEEITYSCKPGYVSR.G + Carbamidomethyl (C)
☑	790	795.0377	2382.0913	2382.0838	3.14	0	(14)	9.8	1	K.TFYEPGEEITYSCKPGYVSR.G + Carbamidomethyl (C)
☑	791	795.0378	2382.0916	2382.0838	3.26	0	(7)	51	1	K.TFYEPGEEITYSCKPGYVSR.G + Carbamidomethyl (C)
☑	792	795.0379	2382.0919	2382.0838	3.39	0	(8)	48	1	K.TFYEPGEEITYSCKPGYVSR.G + Carbamidomethyl (C)
	793	795.0380	2382.0922	2382.0838	3.51	0	(5)	83	3	K.TFYEPGEEITYSCKPGYVSR.G + Carbamidomethyl (C)
☑	794	795.0383	2382.0931	2382.0838	3.89	0	(4)	1.1e+002	1	K.TFYEPGEEITYSCKPGYVSR.G + Carbamidomethyl (C)
☑	795	1192.0540	2382.0934	2382.0838	4.05	0	(57)	0.00054	1	K.TFYEPGEEITYSCKPGYVSR.G + Carbamidomethyl (C)
☑	796	1192.0560	2382.0974	2382.0838	5.73	0	(50)	0.0027	1	K.TFYEPGEEITYSCKPGYVSR.G + Carbamidomethyl (C)
☑	797	796.0056	2384.9950	2384.9889	2.53	0	(35)	0.045	1	K.ATFGCHDGYSLDGPEEIECTK.L + 2 Carbamidomethyl (C)
☑	798	1193.5060	2384.9974	2384.9889	3.57	0	61	0.00013	1	K.ATFGCHDGYSLDGPEEIECTK.L + 2 Carbamidomethyl (C)
☑	858	658.0360	2628.1149	2628.1108	1.54	1	(15)	6.7	1	K.DKATFGCHDGYSLDGPEEIECTK.L + 2 Carbamidomethyl (C)
☑	859	877.0463	2628.1171	2628.1108	2.37	1	61	0.00017	1	K.DKATFGCHDGYSLDGPEEIECTK.L + 2 Carbamidomethyl (C)
☑	904	911.1192	2730.3358	2730.3264	3.42	0	41	0.03	1	K.CPFPSRPDNGFVNYPAKPTLYYK.D + Carbamidomethyl (C)
☑	909	911.4460	2731.3162	2731.3104	2.10	0	(34)	0.17	1	K.CPFPSRPDNGFVNYPAKPTLYYK.D + Carboxymethyl (C)
☑	937	940.4955	2818.4647	2818.4550	3.43	0	39	0.028	1	K.WSPELPVCAPIICPPPSIPTFATLR.V + 2 Carbamidomethyl (C)
☑	938	1410.2420	2818.4694	2818.4550	5.12	0	(34)	0.084	1	K.WSPELPVCAPIICPPPSIPTFATLR.V + 2 Carbamidomethyl (C)
☑	941	945.4877	2833.4413	2833.4547	-4.73	0	(13)	13	1	K.WSPELPVCAPIICPPPSIPTFATLR.V + Acrylamide (C); Carboxymethyl (C)
☑	964	595.6981	2973.4541	2973.4483	1.95	1	(16)	10	1	K.CPFPSRPDNGFVNYPAKPTLYYKDK.A + Carbamidomethyl (C)
☑	965	744.3712	2973.4557	2973.4483	2.48	1	(12)	25	1	K.CPFPSRPDNGFVNYPAKPTLYYKDK.A + Carbamidomethyl (C)
☑	966	595.8942	2974.4346	2974.4323	0.77	1	30	0.47	1	K.CPFPSRPDNGFVNYPAKPTLYYKDK.A + Carboxymethyl (C)
☑	967	744.6172	2974.4397	2974.4323	2.47	1	(2)	2.5e+002	1	K.CPFPSRPDNGFVNYPAKPTLYYKDK.A + Carboxymethyl (C)
☑	974	1023.4630	3067.3672	3067.3546	4.11	0	13	20	1	R.YTTFEYPNTISFSCNTGFYLNGADSAK.C + Carbamidomethyl (C)
☑	1025	881.6930	3522.7429	3522.7350	2.25	1	(12)	28	1	K.CTEEGKWSPELPVCAPIICPPPSIPTFATLR.V + 3 Carbamidomethyl (C)
☑	1026	1175.2560	3522.7462	3522.7350	3.18	1	(41)	0.033	1	K.CTEEGKWSPELPVCAPIICPPPSIPTFATLR.V + 3 Carbamidomethyl (C)
☑	1027	881.6942	3522.7477	3522.7350	3.62	1	(19)	6.2	1	K.CTEEGKWSPELPVCAPIICPPPSIPTFATLR.V + 3 Carbamidomethyl (C)
☑	1028	1175.2570	3522.7492	3522.7350	4.04	1	64	0.00018	1	K.CTEEGKWSPELPVCAPIICPPPSIPTFATLR.V + 3 Carbamidomethyl (C)
☑	1039	885.6908	3538.7341	3538.7186	4.38	1	(8)	76	1	K.CTEEGKWSPELPVCAPIICPPPSIPTFATLR.V + Acrylamide (C); 2 Carboxymethyl (C)
	1040	885.6912	3538.7357	3538.7186	4.83	1	(5)	1.5e+002	2	K.CTEEGKWSPELPVCAPIICPPPSIPTFATLR.V + Acrylamide (C); 2 Carboxymethyl (C)
☑	1041	1180.5870	3538.7392	3538.7186	5.81	1	(15)	17	1	K.CTEEGKWSPELPVCAPIICPPPSIPTFATLR.V + Acrylamide (C); 2 Carboxymethyl (C)
☑	1042	1180.5870	3538.7392	3538.7186	5.81	1	(24)	2.1	1	K.CTEEGKWSPELPVCAPIICPPPSIPTFATLR.V + Acrylamide (C); 2 Carboxymethyl (C)

2. APOH_CANFA **Mass:** 38378 **Score:** 268 **Queries matched:** 38 **emPAI:** 1.10
Beta-2-glycoprotein 1 OS=Canis familiaris GN=APOH PE=2 SV=1
☐ Check to include this hit in error tolerant search or archive report

	Query	Observed	Mr(expt)	Mr(calc)	ppm	Miss	Score	Expect	Rank	Peptide
	21	402.1916	802.3686	802.3684	0.36	0	(1)	31	2	K.VSFYCK.N + Carbamidomethyl (C)
☑	22	402.1917	802.3688	802.3684	0.61	0	5	12	1	K.VSFYCK.N + Carbamidomethyl (C)
	72	496.7219	991.4292	991.4281	1.19	0	65	9.2e-006	1	K.TDASDVKPC.- + Carbamidomethyl (C)
	73	496.7219	991.4292	991.4281	1.19	0	(56)	6.7e-005	1	K.TDASDVKPC.- + Carbamidomethyl (C)
	74	497.2137	992.4128	992.4121	0.77	0	(55)	0.0002	1	K.TDASDVKPC.- + Carboxymethyl (C)
	157	368.8542	1103.5408	1103.5400	0.72	0	(16)	4.2	1	K.EHSSLAFWK.T
	158	368.8543	1103.5411	1103.5400	0.99	0	(27)	0.3	1	K.EHSSLAFWK.T
	159	368.8543	1103.5411	1103.5400	0.99	0	(17)	3.3	1	K.EHSSLAFWK.T
	160	552.7779	1103.5412	1103.5400	1.15	0	29	0.18	1	K.EHSSLAFWK.T
	161	552.7781	1103.5416	1103.5400	1.51	0	(3)	80	1	K.EHSSLAFWK.T
	162	368.8546	1103.5420	1103.5400	1.81	0	(16)	3.5	1	K.EHSSLAFWK.T
	307	501.5980	1501.7722	1501.7711	0.69	0	(31)	0.13	1	R.VCPFAGILENGAVR.Y + Carbamidomethyl (C)
	308	501.5983	1501.7731	1501.7711	1.29	0	(40)	0.013	1	R.VCPFAGILENGAVR.Y + Carbamidomethyl (C)
	309	751.8940	1501.7734	1501.7711	1.54	0	65	5.5e-005	1	R.VCPFAGILENGAVR.Y + Carbamidomethyl (C)
	310	751.8945	1501.7744	1501.7711	2.21	0	(25)	0.47	1	R.VCPFAGILENGAVR.Y + Carbamidomethyl (C)

	311	751.8950	1501.7754	1501.7711	2.87	0	(48)	0.0026	1	R.VCPFAGILENGAVR.Y + Carbamidomethyl (C)
	312	751.8951	1501.7756	1501.7711	3.01	0	(34)	0.065	1	R.VCPFAGILENGAVR.Y + Carbamidomethyl (C)
	313	751.8952	1501.7758	1501.7711	3.14	0	(20)	1.5	1	R.VCPFAGILENGAVR.Y + Carbamidomethyl (C)
	314	751.8959	1501.7772	1501.7711	4.07	0	(36)	0.037	1	R.VCPFAGILENGAVR.Y + Carbamidomethyl (C)
	315	752.3849	1502.7552	1502.7551	0.07	0	(22)	1.1	1	R.VCPFAGILENGAVR.Y + Carboxymethyl (C)
	316	501.9263	1502.7571	1502.7551	1.28	0	(24)	0.56	1	R.VCPFAGILENGAVR.Y + Carboxymethyl (C)
	318	752.3862	1502.7578	1502.7551	1.80	0	(28)	0.25	1	R.VCPFAGILENGAVR.Y + Carboxymethyl (C)
	319	752.3862	1502.7578	1502.7551	1.80	0	(19)	2	1	R.VCPFAGILENGAVR.Y + Carboxymethyl (C)
	320	752.3864	1502.7582	1502.7551	2.07	0	(18)	2.8	1	R.VCPFAGILENGAVR.Y + Carboxymethyl (C)
	321	752.3864	1502.7582	1502.7551	2.07	0	(35)	0.055	1	R.VCPFAGILENGAVR.Y + Carboxymethyl (C)
	322	752.3865	1502.7584	1502.7551	2.20	0	(39)	0.023	1	R.VCPFAGILENGAVR.Y + Carboxymethyl (C)
	323	752.3865	1502.7584	1502.7551	2.20	0	(19)	1.9	1	R.VCPFAGILENGAVR.Y + Carboxymethyl (C)
	324	752.3866	1502.7586	1502.7551	2.33	0	(16)	4.1	1	R.VCPFAGILENGAVR.Y + Carboxymethyl (C)
	325	752.3867	1502.7588	1502.7551	2.47	0	(44)	0.0059	1	R.VCPFAGILENGAVR.Y + Carboxymethyl (C)
	326	752.3870	1502.7594	1502.7551	2.87	0	(27)	0.37	1	R.VCPFAGILENGAVR.Y + Carboxymethyl (C)
	327	752.3874	1502.7602	1502.7551	3.40	0	(22)	0.91	1	R.VCPFAGILENGAVR.Y + Carboxymethyl (C)
	368	385.6911	1538.7353	1538.7340	0.84	1	(2)	74	4	K.CFKEHSSLAFWK.T + Carbamidomethyl (C)
	369	513.9191	1538.7355	1538.7340	0.95	1	(18)	2.3	1	K.CFKEHSSLAFWK.T + Carbamidomethyl (C)
	370	513.9193	1538.7361	1538.7340	1.34	1	(22)	0.77	1	K.CFKEHSSLAFWK.T + Carbamidomethyl (C)
	371	770.3756	1538.7366	1538.7340	1.72	1	37	0.027	1	K.CFKEHSSLAFWK.T + Carbamidomethyl (C)
	374	514.2474	1539.7204	1539.7180	1.53	1	(15)	4.4	1	K.CFKEHSSLAFWK.T + Carboxymethyl (C)
☑	641	**655.9820**	**1964.9242**	**1964.9203**	**1.96**	**0**	**22**	**1.3**	**1**	**K.CPFPSRPDNGFVNYPAK.Q + Carbamidomethyl (C)**
☑	642	**655.9823**	**1964.9251**	**1964.9203**	**2.42**	**0**	**(8)**	**34**	**1**	**K.CPFPSRPDNGFVNYPAK.Q + Carbamidomethyl (C)**

3. K2C1_PANTR **Mass:** 65450 **Score:** 132 **Queries matched:** 10 **emPAI:** 0.34
Keratin, type II cytoskeletal 1 OS=Pan troglodytes GN=KRT1 PE=2 SV=1
☐ Check to include this hit in error tolerant search or archive report

	Query	Observed	Mr(expt)	Mr(calc)	ppm	Miss	Score	Expect	Rank	Peptide
☑	29	**416.7484**	**831.4822**	**831.4814**	**1.02**	**0**	**19**	**1.1**	**1**	**K.SISISVAR.G**
☑	42	**437.7537**	**873.4928**	**873.4920**	**1.00**	**0**	**30**	**0.15**	**1**	**R.SLVNLGGSK.S**
☑	106	**517.2624**	**1032.5102**	**1032.5087**	**1.47**	**0**	**36**	**0.025**	**1**	**R.TLLEGEESR.M**
☑	132	**533.2651**	**1064.5156**	**1064.5138**	**1.73**	**0**	**20**	**0.98**	**1**	**K.AQYEDIAQK.S**
☑	218	**590.3044**	**1178.5942**	**1178.5931**	**0.95**	**0**	**63**	**5.6e-005**	**1**	**K.YEELQITAGR.H**
☑	264	**651.8627**	**1301.7108**	**1301.7078**	**2.32**	**0**	**72**	**7.8e-006**	**1**	**R.SLDLDSIIAEVK.A**
☑	277	**465.2492**	**1392.7258**	**1392.7249**	**0.65**	**1**	**13**	**7.4**	**1**	**R.TNAENEFVTIKK.D**
☑	292	**738.3979**	**1474.7812**	**1474.7780**	**2.21**	**0**	**50**	**0.0014**	**1**	**R.FLEQQNQVLQTK.W**
☑	426	**546.9589**	**1637.8549**	**1637.8525**	**1.42**	**1**	**24**	**0.52**	**1**	**K.SLNNQFASFIDKVR.F**
☑	427	**546.9590**	**1637.8552**	**1637.8525**	**1.61**	**1**	**(21)**	**1.1**	**1**	**K.SLNNQFASFIDKVR.F**

4. THIO_ECOLI **Mass:** 11799 **Score:** 87 **Queries matched:** 2 **emPAI:** 0.29
Thioredoxin-1 OS=Escherichia coli (strain K12) GN=trxA PE=1 SV=2
☐ Check to include this hit in error tolerant search or archive report

	Query	Observed	Mr(expt)	Mr(calc)	ppm	Miss	Score	Expect	Rank	Peptide
☑	78	**501.3242**	**1000.6338**	**1000.6321**	**1.75**	**0**	**11**	**4.2**	**1**	**R.GIPTLLLFK.N**
☑	256	**634.3367**	**1266.6588**	**1266.6568**	**1.62**	**0**	**87**	**2e-007**	**1**	**K.LNIDQNPGTAPK.Y**

Proteins matching the same set of peptides:
THIO_SALTI **Mass:** 11799 **Score:** 87 **Queries matched:** 2
Thioredoxin-1 OS=Salmonella typhi GN=trxA PE=3 SV=2
THIO_SALTY **Mass:** 11799 **Score:** 87 **Queries matched:** 2
Thioredoxin-1 OS=Salmonella typhimurium GN=trxA PE=3 SV=2
THIO_SHIFL **Mass:** 11799 **Score:** 87 **Queries matched:** 2
Thioredoxin-1 OS=Shigella flexneri GN=trxA PE=3 SV=2
THIO_ECOL6 **Mass:** 11799 **Score:** 87 **Queries matched:** 2
Thioredoxin-1 OS=Escherichia coli O6 GN=trxA PE=3 SV=2

THIO_ECO57 **Mass:** 11799 **Score:** 87 **Queries matched:** 2
Thioredoxin-1 OS=Escherichia coli O157:H7 GN=trxA PE=3 SV=2

5. TRYP_PIG **Mass:** 24394 **Score:** 85 **Queries matched:** 3 **emPAI:** 0.29
Trypsin OS=Sus scrofa PE=1 SV=1
☐ Check to include this hit in error tolerant search or archive report

	Query	Observed	Mr(expt)	Mr(calc)	ppm	Miss	Score	Expect	Rank	Peptide
☑	32	**421.7585**	**841.5024**	**841.5022**	**0.35**	**0**	**(37)**	**0.018**	**1**	**R.VATVSLPR.S**
☑	33	**421.7587**	**841.5028**	**841.5022**	**0.83**	**0**	**55**	**0.00028**	**1**	**R.VATVSLPR.S**
☑	740	**737.7077**	**2210.1013**	**2210.0967**	**2.06**	**0**	**60**	**0.00028**	**1**	**R.LGEHNIDVLEGNEQFINAAK.I**

6. THIO_HUMAN **Mass:** 11730 **Score:** 49 **Queries matched:** 5 **emPAI:** 1.16
Thioredoxin OS=Homo sapiens GN=TXN PE=1 SV=3
☐ Check to include this hit in error tolerant search or archive report

	Query	Observed	Mr(expt)	Mr(calc)	ppm	Miss	Score	Expect	Rank	Peptide
☑	47	**454.7278**	**907.4410**	**907.4399**	**1.22**	**0**	**38**	**0.018**	**1**	**K.VGEFSGANK.E**
☑	211	**583.2971**	**1164.5796**	**1164.5775**	**1.86**	**1**	**28**	**0.2**	**1**	**K.VGEFSGANKEK.L**
☑	255	**629.8489**	**1257.6832**	**1257.6816**	**1.31**	**1**	**35**	**0.039**	**1**	**K.EKLEATINELV.-**
☑	293	**493.9276**	**1478.7610**	**1478.7592**	**1.23**	**0**	**25**	**0.42**	**1**	**K.MIKPFFHSLSEK.Y + Oxidation (M)**
☑	472	**852.3691**	**1702.7236**	**1702.7194**	**2.51**	**0**	**13**	**4.4**	**1**	**K.CMPTFQFFK.K + Maleimide-Invitrogen-Biotin+0 (C); Oxidation (M)**

Proteins matching the same set of peptides:
THIO_MACMU **Mass:** 11730 **Score:** 49 **Queries matched:** 5
Thioredoxin OS=Macaca mulatta GN=TXN PE=3 SV=2

7. CASB_BOVIN **Mass:** 25091 **Score:** 46 **Queries matched:** 1 **emPAI:** 0.13
Beta-casein OS=Bos taurus GN=CSN2 PE=1 SV=2
☐ Check to include this hit in error tolerant search or archive report

	Query	Observed	Mr(expt)	Mr(calc)	ppm	Miss	Score	Expect	Rank	Peptide
☑	739	**734.7277**	**2201.1613**	**2201.1555**	**2.64**	**0**	**46**	**0.0053**	**1**	**R.DMPIQAFLLYQEPVLGPVR.G + Oxidation (M)**

Proteins matching the same set of peptides:
CASB_BUBBU **Mass:** 25090 **Score:** 46 **Queries matched:** 1
Beta-casein OS=Bubalus bubalis GN=CSN2 PE=2 SV=1
CASB_CAPHI **Mass:** 24849 **Score:** 46 **Queries matched:** 1
Beta-casein OS=Capra hircus GN=CSN2 PE=2 SV=1
CASB_SHEEP **Mass:** 24859 **Score:** 46 **Queries matched:** 1
Beta-casein OS=Ovis aries GN=CSN2 PE=1 SV=3

8. CASA1_BOVIN **Mass:** 24513 **Score:** 38 **Queries matched:** 1 **emPAI:** 0.14
Alpha-S1-casein OS=Bos taurus GN=CSN1S1 PE=1 SV=2
☐ Check to include this hit in error tolerant search or archive report

	Query	Observed	Mr(expt)	Mr(calc)	ppm	Miss	Score	Expect	Rank	Peptide
☑	275	**692.8703**	**1383.7260**	**1383.7227**	**2.40**	**0**	**38**	**0.018**	**1**	**R.FFVAPFPEVFGK.E**

Proteins matching the same set of peptides:
CASA1_BUBBU **Mass:** 24311 **Score:** 38 **Queries matched:** 1
Alpha-S1-casein OS=Bubalus bubalis GN=CSN1S1 PE=2 SV=2

Peptide matches not assigned to protein hits: (no details means no match)

	Query	Observed	Mr(expt)	Mr(calc)	ppm	Miss	Score	Expect	Rank	Peptide
☑	274	**691.3296**	**1380.6446**	**1380.6408**	**2.76**	**0**	**34**	**0.049**	**1**	**ALEESNYELEGK**

SI Table 2B b2gpI/TRX-1/TRX-R/NADPH+MPB

Mascot Search Results

```
User          :
Email         :
Search title  : Freda_13_11_08_2.RAW relex2
MS data file  : D:\Data\Mark\13_11_08\Freda_13_11_08_2.RAW
Database      : Sprot 19_12_08 (402482 sequences; 145232059 residues)
Timestamp     : 11 Mar 2009 at 03:54:14 GMT
Protein hits  : APOH_HUMAN   Beta-2-glycoprotein 1 OS=Homo sapiens GN=APOH PE=1 SV=3
                TRXR1_RAT    Thioredoxin reductase 1, cytoplasmic OS=Rattus norvegicus GN=Txnrd1 PE=1 SV=4
                TRXR1_MOUSE  Thioredoxin reductase 1, cytoplasmic OS=Mus musculus GN=Txnrd1 PE=1 SV=3
                TRXR1_BOVIN  Thioredoxin reductase 1, cytoplasmic OS=Bos taurus GN=TXNRD1 PE=2 SV=3
                K2C1_PANTR   Keratin, type II cytoskeletal 1 OS=Pan troglodytes GN=KRT1 PE=2 SV=1
                K1C9_HUMAN   Keratin, type I cytoskeletal 9 OS=Homo sapiens GN=KRT9 PE=1 SV=2
                K2C75_BOVIN  Keratin, type II cytoskeletal 75 OS=Bos taurus GN=KRT75 PE=2 SV=1
                K2C5_HUMAN   Keratin, type II cytoskeletal 5 OS=Homo sapiens GN=KRT5 PE=1 SV=3
                K22E_HUMAN   Keratin, type II cytoskeletal 2 epidermal OS=Homo sapiens GN=KRT2 PE=1 SV=1
                K1C10_HUMAN  Keratin, type I cytoskeletal 10 OS=Homo sapiens GN=KRT10 PE=1 SV=4
                TRYP_PIG     Trypsin OS=Sus scrofa PE=1 SV=1
                THIO_ECOLI   Thioredoxin-1 OS=Escherichia coli (strain K12) GN=trxA PE=1 SV=2
                THIO_HUMAN   Thioredoxin OS=Homo sapiens GN=TXN PE=1 SV=3
                CASA1_BOVIN  Alpha-S1-casein OS=Bos taurus GN=CSN1S1 PE=1 SV=2
                HAS2_XENLA   Hyaluronan synthase 2 OS=Xenopus laevis GN=has2 PE=2 SV=2
```

Probability Based Mowse Score

Ions score is -10*Log(P), where P is the probability that the observed match is a random event.
Individual ions scores > 35 indicate identity or extensive homology (p<0.05).
Protein scores are derived from ions scores as a non-probabilistic basis for ranking protein hits.

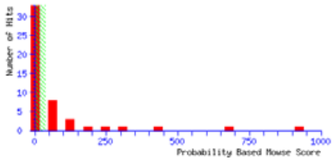

Peptide Summary Report

Format As [Peptide Summary] Help

Significance threshold p< 0.05 Max. number of hits AUTO

Standard scoring ○ MudPIT scoring ● Ions score or expect cut-off 1 Show sub-sets 0

Show pop-ups ● Suppress pop-ups ○ Sort unassigned [Decreasing Score] Require bold red ☑

Select All | Select None | Search Selected | ☐ **Error tolerant** | Archive Report

1. APOH_HUMAN **Mass:** 38273 **Score:** 924 **Queries matched:** 146 **emPAI:** 62.15
Beta-2-glycoprotein 1 OS=Homo sapiens GN=APOH PE=1 SV=3
☐ Check to include this hit in error tolerant search or archive report

	Query	Observed	Mr(expt)	Mr(calc)	ppm	Miss	Score	Expect	Rank	Peptide
☑	25	394.1943	786.3740	786.3735	0.75	0	25	0.11	1	K.VSFFCK.N + Carbamidomethyl (C)
	26	396.7346	791.4546	791.4541	0.67	1	29	0.16	7	K.IQEKFK.N
☑	72	451.7606	901.5066	901.5055	1.26	1	25	0.41	1	K.ASCKVPVK.K + Acrylamide (C)
☑	105	496.7219	991.4292	991.4281	1.19	0	55	8.9e-005	1	K.TDASDVKPC.- + Carbamidomethyl (C)

	Query	Observed	Mr(expt)	Mr(calc)	ppm	Miss	Score	Expect	Rank	Peptide
☑	123	508.8003	1015.5860	1015.5848	1.21	2	15	3.3	1	K.ASCKVPVKK.A + Carbamidomethyl (C)
☑	130	511.7673	1021.5200	1021.5193	0.77	0	60	0.0001	1	K.ATVVYQGER.V
☑	131	511.7675	1021.5204	1021.5193	1.17	0	(33)	0.049	1	K.ATVVYQGER.V
☑	132	511.7675	1021.5204	1021.5193	1.17	0	(31)	0.085	1	K.ATVVYQGER.V
☑	133	511.7675	1021.5204	1021.5193	1.17	0	(39)	0.014	1	K.ATVVYQGER.V
☑	134	511.7675	1021.5204	1021.5193	1.17	0	(38)	0.015	1	K.ATVVYQGER.V
☑	135	511.7676	1021.5206	1021.5193	1.36	0	(34)	0.048	1	K.ATVVYQGER.V
☑	136	511.7676	1021.5206	1021.5193	1.36	0	(32)	0.068	1	K.ATVVYQGER.V
☑	137	511.7676	1021.5206	1021.5193	1.36	0	(38)	0.019	1	K.ATVVYQGER.V
☑	140	515.8083	1029.6020	1029.6005	1.54	2	(11)	7.4	1	K.ASCKVPVKK.A + Acrylamide (C)
☑	211	368.8542	1103.5408	1103.5400	0.72	0	29	0.19	1	K.EHSSLAFWK.T
☑	212	552.7781	1103.5416	1103.5400	1.51	0	(23)	0.68	1	K.EHSSLAFWK.T
☑	229	384.2123	1149.6151	1149.6142	0.75	1	(44)	0.004	1	K.KATVVYQGER.V
☑	230	575.8151	1149.6156	1149.6142	1.25	1	(43)	0.0055	1	K.KATVVYQGER.V
☑	231	575.8151	1149.6156	1149.6142	1.25	1	60	9.8e-005	1	K.KATVVYQGER.V
☑	235	578.7607	1155.5068	1155.5052	1.38	0	14	2.5	1	K.LPECR.E + Maleimide-Invitrogen-Biotin+0 (C)
☑	241	388.1943	1161.5611	1161.5601	0.86	1	(20)	1.1	1	K.FKNGMLHGDK.V + Oxidation (M)
☑	242	581.7881	1161.5616	1161.5601	1.36	1	27	0.26	1	K.FKNGMLHGDK.V + Oxidation (M)
☑	268	603.2449	1204.4752	1204.4740	1.02	0	10	2.7	1	K.CTEEGK.W + Maleimide-Invitrogen-Biotin+0 (C)
☑	269	603.2451	1204.4756	1204.4740	1.36	0	(10)	3	1	K.CTEEGK.W + Maleimide-Invitrogen-Biotin+0 (C)
☑	270	603.2452	1204.4758	1204.4740	1.52	0	(9)	3.4	1	K.CTEEGK.W + Maleimide-Invitrogen-Biotin+0 (C)
☑	297	417.2352	1248.6838	1248.6826	0.91	1	(22)	0.63	1	K.ATVVYQGERVK.I
☑	299	625.3497	1248.6848	1248.6826	1.77	1	40	0.009	1	K.ATVVYQGERVK.I
☑	322	644.2921	1286.5696	1286.5675	1.64	0	(17)	2	1	K.VSFFCK.N + Maleimide-Invitrogen-Biotin+H20+O (C)
☑	366	459.9337	1376.7793	1376.7776	1.22	2	31	0.058	1	K.KATVVYQGERVK.I
	370	463.5688	1387.6846	1387.6839	0.45	1	(7)	27	2	K.ASCKVPVK.K + Maleimide-Invitrogen-Biotin+H20+O (C)
	371	694.8502	1387.6858	1387.6839	1.37	1	(12)	7.3	3	K.ASCKVPVK.K + Maleimide-Invitrogen-Biotin+H20+O (C)
☑	420	737.8141	1473.6136	1473.6116	1.40	0	(29)	0.077	1	K.TDASDVKPC.- + Maleimide-Invitrogen-Biotin+0 (C)
☑	427	738.8230	1475.6314	1475.6272	2.86	0	(22)	0.6	1	K.TDASDVKPC.- + Maleimide-Invitrogen-Biotin+H20 (C)
☑	448	746.8195	1491.6244	1491.6221	1.54	0	(16)	2.7	1	K.TDASDVKPC.- + Maleimide-Invitrogen-Biotin+H20+O (C)
☑	449	746.8196	1491.6246	1491.6221	1.68	0	(14)	2.9	1	K.TDASDVKPC.- + Maleimide-Invitrogen-Biotin+H20+O (C)
☑	450	746.8197	1491.6248	1491.6221	1.81	0	(12)	4.7	1	K.TDASDVKPC.- + Maleimide-Invitrogen-Biotin+H20+O (C)
☑	451	746.8199	1491.6252	1491.6221	2.08	0	(16)	1.8	1	K.TDASDVKPC.- + Maleimide-Invitrogen-Biotin+H20+O (C)
☑	452	746.8199	1491.6252	1491.6221	2.08	0	(28)	0.11	1	K.TDASDVKPC.- + Maleimide-Invitrogen-Biotin+H20+O (C)
☑	453	746.8199	1491.6252	1491.6221	2.08	0	(16)	2.1	1	K.TDASDVKPC.- + Maleimide-Invitrogen-Biotin+H20+O (C)
☑	454	746.8201	1491.6256	1491.6221	2.35	0	(27)	0.17	1	K.TDASDVKPC.- + Maleimide-Invitrogen-Biotin+H20+O (C)
	455	746.8206	1491.6266	1491.6221	3.02	0	(1)	64	4	K.TDASDVKPC.- + Maleimide-Invitrogen-Biotin+H20+O (C)
☑	463	500.2640	1497.7702	1497.7683	1.22	2	(9)	14	1	K.ASCKVPVKK.A + Maleimide-Invitrogen-Biotin+0 (C)
☑	468	501.5980	1501.7722	1501.7711	0.69	0	(41)	0.015	1	R.VCPFAGILENGAVR.Y + Carbamidomethyl (C)
☑	469	751.8947	1501.7748	1501.7711	2.47	0	57	0.00032	1	R.VCPFAGILENGAVR.Y + Carbamidomethyl (C)
	470	501.9262	1502.7568	1502.7551	1.08	0	(13)	8.9	5	R.VCPFAGILENGAVR.Y + Carboxymethyl (C)
	471	501.9263	1502.7571	1502.7551	1.28	0	(22)	1.1	2	R.VCPFAGILENGAVR.Y + Carboxymethyl (C)
☑	472	752.3865	1502.7584	1502.7551	2.20	0	(12)	9.6	1	R.VCPFAGILENGAVR.Y + Carboxymethyl (C)
☑	473	752.3866	1502.7586	1502.7551	2.33	0	(20)	1.5	1	R.VCPFAGILENGAVR.Y + Carboxymethyl (C)
	499	506.2674	1515.7804	1515.7789	0.97	2	(5)	49	3	K.ASCKVPVKK.A + Maleimide-Invitrogen-Biotin+H20+O (C)
	502	758.8983	1515.7820	1515.7789	2.08	2	(7)	34	5	K.ASCKVPVKK.A + Maleimide-Invitrogen-Biotin+H20+O (C)
☑	550	513.9192	1538.7358	1538.7340	1.15	1	(20)	1.2	1	K.CFKEHSSLAFWK.T + Carbamidomethyl (C)
☑	648	552.5943	1654.7611	1654.7596	0.89	1	27	0.33	1	K.NGMLHGDKVSFFCK.N + Carbamidomethyl (C); Oxidation (M)
☑	650	552.9223	1655.7451	1655.7436	0.88	1	(8)	22	1	K.NGMLHGDKVSFFCK.N + Carboxymethyl (C); Oxidation (M)
☑	685	437.7484	1746.9645	1746.9628	0.96	2	(6)	28	1	K.ATVVYQGERVKIQEK.F
☑	686	583.3291	1746.9655	1746.9628	1.52	2	22	0.66	1	K.ATVVYQGERVKIQEK.F
☑	694	591.6647	1771.9723	1771.9695	1.57	0	(30)	0.11	1	K.FICPLTGLWPINTLK.C + Carbamidomethyl (C)
☑	695	886.9943	1771.9740	1771.9695	2.57	0	(39)	0.015	1	K.FICPLTGLWPINTLK.C + Carbamidomethyl (C)
☑	696	886.9944	1771.9742	1771.9695	2.68	0	77	2.6e-006	1	K.FICPLTGLWPINTLK.C + Carbamidomethyl (C)
☑	697	886.9944	1771.9742	1771.9695	2.68	0	(59)	0.00015	1	K.FICPLTGLWPINTLK.C + Carbamidomethyl (C)
☑	698	886.9945	1771.9744	1771.9695	2.79	0	(66)	3.1e-005	1	K.FICPLTGLWPINTLK.C + Carbamidomethyl (C)
☑	699	886.9950	1771.9754	1771.9695	3.36	0	(36)	0.03	1	K.FICPLTGLWPINTLK.C + Carbamidomethyl (C)
☑	766	634.3632	1900.0678	1900.0645	1.75	1	(26)	0.18	1	R.KFICPLTGLWPINTLK.C + Carbamidomethyl (C)
☑	767	634.3635	1900.0687	1900.0645	2.22	1	(14)	2.9	1	R.KFICPLTGLWPINTLK.C + Carbamidomethyl (C)

☑	768	951.0424	1900.0702	1900.0645	3.05	1	48	0.001	1	R.KFICPLTGLWPINTLK.C + Carbamidomethyl (C)
☑	773	638.6739	1912.9999	1912.9969	1.58	0	(35)	0.049	1	R.TCPKPDDLPFSTVVPLK.T + Carbamidomethyl (C)
☑	774	957.5085	1913.0024	1912.9969	2.92	0	41	0.013	1	R.TCPKPDDLPFSTVVPLK.T + Carbamidomethyl (C)
☑	787	644.3154	1929.9244	1929.9230	0.72	2	(14)	8.2	1	K.FKNGMLHGDKVSFFCK.N + Carbamidomethyl (C); Oxidation (M)
☑	788	483.4885	1929.9249	1929.9230	1.00	2	17	4.8	1	K.FKNGMLHGDKVSFFCK.N + Carbamidomethyl (C); Oxidation (M)
☑	790	483.7345	1930.9089	1930.9070	0.99	2	(6)	56	1	K.FKNGMLHGDKVSFFCK.N + Carboxymethyl (C); Oxidation (M)
	791	644.6440	1930.9102	1930.9070	1.65	2	(5)	74	3	K.FKNGMLHGDKVSFFCK.N + Carboxymethyl (C); Oxidation (M)
☑	807	662.3262	1983.9568	1983.9547	1.07	0	(46)	0.0055	1	R.VCPFAGILENGAVR.Y + Maleimide-Invitrogen-Biotin+0 (C)
☑	808	992.9860	1983.9574	1983.9547	1.41	0	(33)	0.13	1	R.VCPFAGILENGAVR.Y + Maleimide-Invitrogen-Biotin+0 (C)
☑	809	662.3267	1983.9583	1983.9547	1.83	0	(46)	0.0067	1	R.VCPFAGILENGAVR.Y + Maleimide-Invitrogen-Biotin+0 (C)
☑	810	992.9871	1983.9596	1983.9547	2.52	0	(27)	0.45	1	R.VCPFAGILENGAVR.Y + Maleimide-Invitrogen-Biotin+0 (C)
☑	813	662.9985	1985.9737	1985.9703	1.70	0	(32)	0.14	1	R.VCPFAGILENGAVR.Y + Maleimide-Invitrogen-Biotin+H20 (C)
☑	822	668.3303	2001.9691	2001.9652	1.93	0	(47)	0.0051	1	R.VCPFAGILENGAVR.Y + Maleimide-Invitrogen-Biotin+H20+O (C)
☑	823	1001.9930	2001.9714	2001.9652	3.12	0	(23)	1.1	1	R.VCPFAGILENGAVR.Y + Maleimide-Invitrogen-Biotin+H20+O (C)
☑	829	673.6608	2017.9606	2017.9714	-5.35	1	(31)	0.2	1	K.CTPRVCPFAGILENGAVR.Y + 2 Carboxymethyl (C)
☑	830	673.6625	2017.9657	2017.9714	-2.82	1	(31)	0.21	1	K.CTPRVCPFAGILENGAVR.Y + 2 Carboxymethyl (C)
☑	835	674.6476	2020.9210	2020.9175	1.71	1	(3)	1.1e+002	1	K.CFKEHSSLAFWK.T + Maleimide-Invitrogen-Biotin+0 (C)
☑	836	1011.4700	2020.9254	2020.9175	3.92	1	30	0.22	1	K.CFKEHSSLAFWK.T + Maleimide-Invitrogen-Biotin+0 (C)
☑	848	510.7398	2038.9301	2038.9281	0.99	1	(8)	35	1	K.CFKEHSSLAFWK.T + Maleimide-Invitrogen-Biotin+H20+O (C)
☑	849	680.6513	2038.9321	2038.9281	1.96	1	(6)	56	1	K.CFKEHSSLAFWK.T + Maleimide-Invitrogen-Biotin+H20+O (C)
☑	873	1043.4610	2084.9074	2084.9031	2.10	0	82	1.1e-006	1	K.CSYTEDAQCIDGTIEVPK.C + 2 Carbamidomethyl (C)
☑	902	719.3265	2154.9577	2154.9537	1.86	1	(1)	1.5e+002	1	K.NGMLHGDKVSFFCK.N + Maleimide-Invitrogen-Biotin+H20+O (C); Oxidation (M)
☑	903	719.3265	2154.9577	2154.9537	1.86	1	(10)	20	1	K.NGMLHGDKVSFFCK.N + Maleimide-Invitrogen-Biotin+H20+O (C); Oxidation (M)
☑	914	738.6750	2213.0032	2212.9980	2.32	1	34	0.086	1	K.KCSYTEDAQCIDGTIEVPK.C + 2 Carbamidomethyl (C)
☑	917	752.3939	2254.1599	2254.1530	3.04	0	(16)	5.2	1	K.FICPLTGLWPINTLK.C + Maleimide-Invitrogen-Biotin+0 (C)
☑	918	1128.0890	2254.1634	2254.1530	4.63	0	(23)	1	1	K.FICPLTGLWPINTLK.C + Maleimide-Invitrogen-Biotin+0 (C)
☑	919	758.3968	2272.1686	2272.1636	2.20	0	(25)	0.71	1	K.FICPLTGLWPINTLK.C + Maleimide-Invitrogen-Biotin+H20+O (C)
☑	920	758.3969	2272.1689	2272.1636	2.33	0	(22)	1.6	1	K.FICPLTGLWPINTLK.C + Maleimide-Invitrogen-Biotin+H20+O (C)
☑	921	758.3969	2272.1689	2272.1636	2.33	0	(30)	0.25	1	K.FICPLTGLWPINTLK.C + Maleimide-Invitrogen-Biotin+H20+O (C)
☑	922	1137.0930	2272.1714	2272.1636	3.47	0	(7)	41	1	K.FICPLTGLWPINTLK.C + Maleimide-Invitrogen-Biotin+H20+O (C)
☑	923	1137.0930	2272.1714	2272.1636	3.47	0	(10)	20	1	K.FICPLTGLWPINTLK.C + Maleimide-Invitrogen-Biotin+H20+O (C)
☑	924	1137.0940	2272.1734	2272.1636	4.35	0	(11)	18	1	K.FICPLTGLWPINTLK.C + Maleimide-Invitrogen-Biotin+H20+O (C)
☑	964	1192.0520	2382.0894	2382.0838	2.37	0	71	2.2e-005	1	K.TFYEPGEEITYSCKPGYVSR.G + Carbamidomethyl (C)
☑	965	795.0372	2382.0898	2382.0838	2.51	0	(11)	25	1	K.TFYEPGEEITYSCKPGYVSR.G + Carbamidomethyl (C)
☑	966	796.0062	2384.9968	2384.9889	3.29	0	33	0.09	1	K.ATFGCHDGYSLDGPEEIECTK.L + 2 Carbamidomethyl (C)
☑	968	799.4028	2395.1866	2395.1804	2.59	0	(27)	0.64	1	R.TCPKPDDLPFSTVVPLK.T + Maleimide-Invitrogen-Biotin+0 (C)
☑	971	801.0956	2400.2650	2400.2585	2.68	1	(15)	5.9	1	R.KFICPLTGLWPINTLK.C + Maleimide-Invitrogen-Biotin+H20+O (C)
☑	972	801.0956	2400.2650	2400.2585	2.68	1	(28)	0.3	1	R.KFICPLTGLWPINTLK.C + Maleimide-Invitrogen-Biotin+H20+O (C)
☑	973	801.0958	2400.2656	2400.2585	2.93	1	(16)	4.9	1	R.KFICPLTGLWPINTLK.C + Maleimide-Invitrogen-Biotin+H20+O (C)
☑	980	805.4064	2413.1974	2413.1909	2.67	0	(26)	0.75	1	R.TCPKPDDLPFSTVVPLK.T + Maleimide-Invitrogen-Biotin+H20+O (C)
☑	981	805.4066	2413.1980	2413.1909	2.92	0	(21)	2.5	1	R.TCPKPDDLPFSTVVPLK.T + Maleimide-Invitrogen-Biotin+H20+O (C)
☑	1019	834.0663	2499.1771	2499.1709	2.48	1	(23)	1.6	1	K.CTPRVCPFAGILENGAVR.Y + Carboxymethyl (C); Maleimide-Invitrogen-Biotin+0 (C)
☑	1020	834.0674	2499.1804	2499.1709	3.80	1	45	0.0098	1	K.CTPRVCPFAGILENGAVR.Y + Carboxymethyl (C); Maleimide-Invitrogen-Biotin+0 (C)
☑	1025	839.7416	2516.2030	2516.1974	2.21	1	(24)	1.3	1	K.CTPRVCPFAGILENGAVR.Y + Carbamidomethyl (C); Maleimide-Invitrogen-Biotin+H20+O (C)
☑	1026	840.0684	2517.1834	2517.1814	0.77	1	(22)	1.9	1	K.CTPRVCPFAGILENGAVR.Y + Carboxymethyl (C); Maleimide-Invitrogen-Biotin+H20+O (C)
☑	1033	856.7051	2567.0935	2567.0866	2.68	0	(17)	3.9	1	K.CSYTEDAQCIDGTIEVPK.C + Carbamidomethyl (C); Maleimide-Invitrogen-Biotin+0 (C)
☑	1040	862.7088	2585.1046	2585.0972	2.87	0	(26)	0.59	1	K.CSYTEDAQCIDGTIEVPK.C + Carbamidomethyl (C); Maleimide-Invitrogen-Biotin+H20+O (C)
☑	1057	658.0361	2628.1153	2628.1108	1.70	1	(15)	6.3	1	K.DKATFGCHDGYSLDGPEEIECTK.L + 2 Carbamidomethyl (C)
☑	1058	877.0460	2628.1162	2628.1108	2.03	1	58	0.00033	1	K.DKATFGCHDGYSLDGPEEIECTK.L + 2 Carbamidomethyl (C)
☑	1082	899.4037	2695.1893	2695.1815	2.87	1	(15)	9.3	1	K.KCSYTEDAQCIDGTIEVPK.C + Carbamidomethyl (C); Maleimide-Invitrogen-Biotin+0 (C)
☑	1092	905.4069	2713.1989	2713.1921	2.49	1	(20)	3.2	1	K.KCSYTEDAQCIDGTIEVPK.C + Carbamidomethyl (C); Maleimide-Invitrogen-Biotin+H20+O (C)
☑	1098	683.5901	2730.3313	2730.3264	1.78	0	(13)	18	1	K.CPFPSRPDNGFVNYPAKPTLYYK.D + Carbamidomethyl (C)
☑	1099	911.1179	2730.3319	2730.3264	1.99	0	31	0.28	1	K.CPFPSRPDNGFVNYPAKPTLYYK.D + Carbamidomethyl (C)
☑	1100	683.8362	2731.3157	2731.3104	1.92	0	(3)	1.9e+002	1	K.CPFPSRPDNGFVNYPAKPTLYYK.D + Carboxymethyl (C)
☑	1108	929.8085	2786.4037	2786.3958	2.83	1	(4)	1.4e+002	1	K.FICPLTGLWPINTLKCTPR.V + Carbamidomethyl (C); Maleimide-Invitrogen-Biotin+H20+O (C)
☑	1109	929.8085	2786.4037	2786.3958	2.83	1	(7)	63	1	K.FICPLTGLWPINTLKCTPR.V + Carbamidomethyl (C); Maleimide-Invitrogen-Biotin+H20+O (C)
☑	1120	955.7653	2864.2741	2864.2673	2.36	0	(13)	18	1	K.TFYEPGEEITYSCKPGYVSR.G + Maleimide-Invitrogen-Biotin+0 (C)
☑	1124	961.7687	2882.2843	2882.2779	2.22	0	(13)	19	1	K.TFYEPGEEITYSCKPGYVSR.G + Maleimide-Invitrogen-Biotin+H20+O (C)
☑	1125	721.5784	2882.2845	2882.2779	2.30	0	(24)	1.7	1	K.TFYEPGEEITYSCKPGYVSR.G + Maleimide-Invitrogen-Biotin+H20+O (C)

☑	1127	961.7690	2882.2852	2882.2779	2.53	0	(6)	89	1	K.TFYEPGEEITYSCKPGYVSR.G + Maleimide-Invitrogen-Biotin+H20+O (C)
☑	1159	595.6981	2973.4541	2973.4483	1.95	1	20	3.9	1	K.CPFPSRPDNGFVNYPAKPTLYYKDK.A + Carbamidomethyl (C)
☑	1160	744.3715	2973.4569	2973.4483	2.88	1	(11)	29	1	K.CPFPSRPDNGFVNYPAKPTLYYKDK.A + Carbamidomethyl (C)
☑	1161	744.3718	2973.4581	2973.4483	3.28	1	(13)	19	1	K.CPFPSRPDNGFVNYPAKPTLYYKDK.A + Carbamidomethyl (C)
☑	1179	1023.4370	3067.2892	3067.2807	2.77	0	(5)	78	1	K.CSYTEDAQCIDGTIEVPK.C + Maleimide-Invitrogen-Biotin+0 (C); Maleimide-Invitrogen-Biotin+H20+O (C)
☑	1182	1029.4390	3085.2952	3085.2912	1.28	0	(16)	7	1	K.CSYTEDAQCIDGTIEVPK.C + 2 Maleimide-Invitrogen-Biotin+H20+O (C)
☑	1183	1029.4410	3085.3012	3085.2912	3.22	0	(10)	30	1	K.CSYTEDAQCIDGTIEVPK.C + 2 Maleimide-Invitrogen-Biotin+H20+O (C)
☑	1191	783.0847	3128.3097	3128.3049	1.53	1	(5)	81	1	K.DKATFGCHDGYSLDGPEEIECTK.L + Carbamidomethyl (C); Maleimide-Invitrogen-Biotin+H20+O (C)
☑	1192	1043.7800	3128.3182	3128.3049	4.24	1	(12)	17	1	K.DKATFGCHDGYSLDGPEEIECTK.L + Carbamidomethyl (C); Maleimide-Invitrogen-Biotin+H20+O (C)
☑	1198	1071.8470	3212.5192	3212.5099	2.87	0	(29)	0.59	1	K.CPFPSRPDNGFVNYPAKPTLYYK.D + Maleimide-Invitrogen-Biotin+0 (C)
☑	1199	1072.1390	3213.3952	3213.3862	2.80	1	(18)	5.8	1	K.KCSYTEDAQCIDGTIEVPK.C + 2 Maleimide-Invitrogen-Biotin+H20+O (C)
☑	1201	1077.8510	3230.5312	3230.5205	3.30	0	(22)	3	1	K.CPFPSRPDNGFVNYPAKPTLYYK.D + Maleimide-Invitrogen-Biotin+H20+O (C)
☑	1205	822.6571	3286.5993	3286.5899	2.87	1	17	9.8	1	K.FICPLTGLWPINTLKCTPR.V + 2 Maleimide-Invitrogen-Biotin+H20+O (C)
☑	1206	1096.5410	3286.6012	3286.5899	3.44	1	(6)	1.1e+002	1	K.FICPLTGLWPINTLKCTPR.V + 2 Maleimide-Invitrogen-Biotin+H20+O (C)
☑	1207	1096.5410	3286.6012	3286.5899	3.44	1	(6)	1.3e+002	1	K.FICPLTGLWPINTLKCTPR.V + 2 Maleimide-Invitrogen-Biotin+H20+O (C)
	1225	1129.4700	3385.3882	3385.3771	3.27	0	(5)	94	4	K.ATFGCHDGYSLDGPEEIECTK.L + 2 Maleimide-Invitrogen-Biotin+H20+O (C)
☑	1226	1129.4710	3385.3912	3385.3771	4.16	0	(9)	35	1	K.ATFGCHDGYSLDGPEEIECTK.L + 2 Maleimide-Invitrogen-Biotin+H20+O (C)
☑	1229	854.6808	3414.6941	3414.6848	2.72	2	(5)	1.2e+002	1	R.KFICPLTGLWPINTLKCTPR.V + 2 Maleimide-Invitrogen-Biotin+H20+O (C)
☑	1230	854.6824	3414.7005	3414.6848	4.59	2	6	1.1e+002	1	R.KFICPLTGLWPINTLKCTPR.V + 2 Maleimide-Invitrogen-Biotin+H20+O (C)
☑	1234	864.9176	3455.6413	3455.6319	2.73	1	(9)	69	1	K.CPFPSRPDNGFVNYPAKPTLYYKDK.A + Maleimide-Invitrogen-Biotin+0 (C)
☑	1239	695.7373	3473.6501	3473.6424	2.22	1	(7)	1.1e+002	1	K.CPFPSRPDNGFVNYPAKPTLYYKDK.A + Maleimide-Invitrogen-Biotin+H20+O (C)
☑	1249	1175.2540	3522.7402	3522.7350	1.48	1	(8)	68	1	K.CTEEGKWSPELPVCAPIICPPPSIPTFATLR.V + 3 Carbamidomethyl (C)
☑	1250	1175.2550	3522.7432	3522.7350	2.33	1	51	0.0035	1	K.CTEEGKWSPELPVCAPIICPPPSIPTFATLR.V + 3 Carbamidomethyl (C)
☑	1258	1190.1930	3567.5572	3567.5486	2.39	0	32	0.31	1	R.YTTFEYPNTISFSCNTGFYLNGADSAK.C + Maleimide-Invitrogen-Biotin+H20+O (C)

2. TRXR1_RAT **Mass:** 54352 **Score:** 677 **Queries matched:** 57 **emPAI:** 6.38

Thioredoxin reductase 1, cytoplasmic OS=Rattus norvegicus GN=Txnrd1 PE=1 SV=4

☐ Check to include this hit in error tolerant search or archive report

	Query	Observed	Mr(expt)	Mr(calc)	ppm	Miss	Score	Expect	Rank	Peptide
☑	7	360.2083	718.4020	718.4014	0.93	0	11	8.5	1	R.QFVPTK.I
☑	11	373.7154	745.4162	745.4156	0.82	0	25	0.54	1	K.VICNLK.D + Carbamidomethyl (C)
	37	412.7297	823.4448	823.4440	1.07	0	18	1.3	2	R.LYGGSTVK.C
	57	431.7375	861.4604	861.4596	0.97	0	19	1.7	2	R.YLGIPGDK.E
☑	99	491.2541	980.4936	980.4927	0.99	1	32	0.038	1	K.EKVYSAER.F
☑	124	508.8035	1015.5924	1015.5914	1.06	0	40	0.0087	1	R.TIGLETVGVK.I
☑	147	521.2195	1040.4244	1040.4233	1.09	0	26	0.14	1	R.GFDQDMANK.I + Oxidation (M)
☑	148	521.7643	1041.5140	1041.5131	0.92	0	40	0.013	1	K.VVYENAYGK.F
	169	530.7446	1059.4746	1059.4729	1.65	0	4	29	3	K.CGLTK.Q + Maleimide-Invitrogen-Biotin+0 (C)
	189	539.7498	1077.4850	1077.4835	1.47	0	(4)	34	8	K.CGLTK.Q + Maleimide-Invitrogen-Biotin+H20+O (C)
	225	569.7293	1137.4440	1137.4431	0.87	0	(6)	10	5	R.DSCTR.T + Maleimide-Invitrogen-Biotin+H20+O (C)
	226	569.7296	1137.4446	1137.4431	1.40	0	7	8.5	2	R.DSCTR.T + Maleimide-Invitrogen-Biotin+H20+O (C)
☑	237	387.2245	1158.6517	1158.6509	0.64	0	(22)	0.7	1	R.FLIATGERPR.Y
☑	239	580.3335	1158.6524	1158.6509	1.31	0	(19)	1.2	1	R.FLIATGERPR.Y
☑	240	580.3335	1158.6524	1158.6509	1.31	0	41	0.0083	1	R.FLIATGERPR.Y
☑	248	585.8102	1169.6058	1169.6040	1.55	0	80	8.2e-007	1	K.IEQIEAGTPGR.L
☑	249	390.8768	1169.6086	1169.6080	0.45	1	(33)	0.049	1	K.KVVYENAYGK.F
☑	250	585.8118	1169.6090	1169.6080	0.86	1	62	5.6e-005	1	K.KVVYENAYGK.F
☑	307	420.8841	1259.6305	1259.6292	1.01	1	(23)	0.79	1	K.VICNLKDNER.V + Carbamidomethyl (C)
☑	308	630.8229	1259.6312	1259.6292	1.63	1	41	0.011	1	K.VICNLKDNER.V + Carbamidomethyl (C)
☑	313	634.3732	1266.7318	1266.7296	1.80	0	52	0.00025	1	K.LELTPVAIQAGR.L
☑	327	432.5401	1294.5985	1294.5976	0.70	0	(35)	0.032	1	K.IGEHMEEHGIK.F + Oxidation (M)
☑	328	648.3073	1294.6000	1294.5976	1.92	0	45	0.0032	1	K.IGEHMEEHGIK.F + Oxidation (M)
☑	360	676.8472	1351.6798	1351.6772	1.96	1	42	0.0096	1	R.NYGWKLEDTVK.H
☑	382	471.2688	1410.7846	1410.7831	1.08	1	41	0.0064	1	K.IEQIEAGTPGRLK.V
☑	389	476.5896	1426.7470	1426.7456	0.97	2	24	0.49	1	R.EKKVVYENAYGK.F
☑	442	496.2665	1485.7777	1485.7762	0.99	1	22	0.84	1	K.FIGPHKIMATNNK.G + Oxidation (M)
☑	466	500.9598	1499.8576	1499.8559	1.12	1	(39)	0.0098	1	R.TIGLETVGVKINEK.T

☑	467	750.9369	1499.8592	1499.8559	2.23	1	63	3.6e-005	1	R.TIGLETVGVKINEK.T
☑	545	385.2278	1536.8821	1536.8810	0.73	1	33	0.024	1	K.KLMHQAALLGQALK.D + Oxidation (M)
☑	546	513.3015	1536.8827	1536.8810	1.10	1	(29)	0.066	1	K.KLMHQAALLGQALK.D + Oxidation (M)
☑	547	769.4495	1536.8844	1536.8810	2.26	1	(31)	0.039	1	K.KLMHQAALLGQALK.D + Oxidation (M)
	627	541.9441	1622.8105	1622.8086	1.14	1	7	34	2	R.SILLRGFDQDMANK.I + Oxidation (M)
☑	668	428.7204	1710.8525	1710.8512	0.79	1	9	22	1	K.IGEHMEEHGIKFIR.Q + Oxidation (M)
☑	692	442.7443	1766.9481	1766.9461	1.13	1	26	0.33	1	K.LMHQAALLGQALKDSR.N + Oxidation (M)
☑	706	596.3478	1786.0216	1786.0200	0.88	2	14	2.2	1	R.TIGLETVGVKINEKTGK.I
	707	447.5130	1786.0229	1786.0200	1.62	2	(1)	42	7	R.TIGLETVGVKINEKTGK.I
☑	750	624.3401	1869.9985	1869.9949	1.93	1	(40)	0.016	1	R.QFVPTKIEQIEAGTPGR.L
☑	751	936.0073	1870.0000	1869.9949	2.78	1	66	3.9e-005	1	R.QFVPTKIEQIEAGTPGR.L
☑	759	474.7679	1895.0425	1895.0411	0.76	2	(32)	0.066	1	K.KLMHQAALLGQALKDSR.N + Oxidation (M)
☑	760	632.6884	1895.0434	1895.0411	1.22	2	37	0.019	1	K.KLMHQAALLGQALKDSR.N + Oxidation (M)
☑	783	963.0070	1923.9994	1923.9942	2.73	0	75	4.9e-006	1	K.SYDFDLIIIGGGSGGLAAAK.E
☑	827	669.9841	2006.9305	2006.9268	1.81	0	(30)	0.23	1	K.MTESVQNHIGSLNWGYR.V + Oxidation (M)
☑	828	1004.4730	2006.9314	2006.9268	2.29	0	32	0.12	1	K.MTESVQNHIGSLNWGYR.V + Oxidation (M)
☑	856	512.7529	2046.9825	2046.9799	1.26	2	(7)	50	1	R.NYGWKLEDTVKHDWEK.M
☑	857	683.3353	2046.9841	2046.9799	2.03	2	14	9.9	1	R.NYGWKLEDTVKHDWEK.M
☑	877	704.7328	2111.1766	2111.1739	1.28	2	30	0.08	1	R.QFVPTKIEQIEAGTPGRLK.V
☑	878	528.8015	2111.1769	2111.1739	1.43	2	(10)	7.9	1	R.QFVPTKIEQIEAGTPGRLK.V
☑	879	704.7332	2111.1778	2111.1739	1.85	2	(11)	7.5	1	R.QFVPTKIEQIEAGTPGRLK.V
☑	952	773.3453	2317.0141	2317.0103	1.62	1	5	69	1	R.GFDQDMANKIGEHMEEHGIK.F + 2 Oxidation (M)
☑	953	775.3822	2323.1248	2323.1179	2.94	0	(41)	0.021	1	K.STNSEETIEDEFNTVLLAVGR.D
☑	954	1162.5710	2323.1274	2323.1179	4.09	0	111	2.1e-009	1	K.STNSEETIEDEFNTVLLAVGR.D
☑	1078	893.1273	2676.3601	2676.3534	2.49	0	23	1.5	1	K.IPVTDEEQTNVPYIYAIGDILEGK.L
	1101	684.3245	2733.2689	2733.2639	1.83	2	4	1.6e+002	3	R.GFDQDMANKIGEHMEEHGIKFIR.Q + 2 Oxidation (M)
☑	1166	998.8039	2993.3899	2993.3827	2.40	1	30	0.42	1	R.YLGIPGDKEYCISSDDLFSLPYCPGK.T + 2 Carbamidomethyl (C)
☑	1233	1148.5270	3442.5592	3442.5504	2.54	1	75	1.5e-005	1	K.STNSEETIEDEFNTVLLAVGRDSCTR.T + Maleimide-Invitrogen-Biotin+H20+O (C)
☑	1245	1165.5380	3493.5922	3493.5768	4.41	1	(13)	25	1	R.YLGIPGDKEYCISSDDLFSLPYCPGK.T + Carbamidomethyl (C); Maleimide-Invitrogen-Biotin+H20+O (C)

3. TRXR1_MOUSE **Mass:** 67042 **Score:** 450 **Queries matched:** 39 **emPAI:** 1.60
Thioredoxin reductase 1, cytoplasmic OS=Mus musculus GN=Txnrd1 PE=1 SV=3
☐ Check to include this hit in error tolerant search or archive report

	Query	Observed	Mr(expt)	Mr(calc)	ppm	Miss	Score	Expect	Rank	Peptide
	7	360.2083	718.4020	718.4014	0.93	0	11	8.5	1	R.QFVPTK.I
	57	431.7375	861.4604	861.4596	0.97	0	19	1.7	2	R.YLGIPGDK.E
	124	508.8035	1015.5924	1015.5914	1.06	0	40	0.0087	1	R.TIGLETVGVK.I
	147	521.2195	1040.4244	1040.4233	1.09	0	26	0.14	1	R.GFDQDMANK.I + Oxidation (M)
	169	530.7446	1059.4746	1059.4729	1.65	0	4	29	3	K.CGLTK.Q + Maleimide-Invitrogen-Biotin+0 (C)
	189	539.7498	1077.4850	1077.4835	1.47	0	(4)	34	8	K.CGLTK.Q + Maleimide-Invitrogen-Biotin+H20+O (C)
	225	569.7293	1137.4440	1137.4431	0.87	0	(6)	10	5	R.DSCTR.T + Maleimide-Invitrogen-Biotin+H20+O (C)
	226	569.7296	1137.4446	1137.4431	1.40	0	7	8.5	2	R.DSCTR.T + Maleimide-Invitrogen-Biotin+H20+O (C)
	237	387.2245	1158.6517	1158.6509	0.64	0	(22)	0.7	1	R.FLIATGERPR.Y
	239	580.3335	1158.6524	1158.6509	1.31	0	(19)	1.2	1	R.FLIATGERPR.Y
	240	580.3335	1158.6524	1158.6509	1.31	0	41	0.0083	1	R.FLIATGERPR.Y
	248	585.8102	1169.6058	1169.6040	1.55	0	80	8.2e-007	1	K.IEQIEAGTPGR.L
	313	634.3732	1266.7318	1266.7296	1.80	0	52	0.00025	1	K.LELTPVAIQAGR.L
	327	432.5401	1294.5985	1294.5976	0.70	0	(35)	0.032	1	K.IGEHMEEHGIK.F + Oxidation (M)
	328	648.3073	1294.6000	1294.5976	1.92	0	45	0.0032	1	K.IGEHMEEHGIK.F + Oxidation (M)
	466	500.9598	1499.8576	1499.8559	1.12	1	(39)	0.0098	1	R.TIGLETVGVKINEK.T
	467	750.9369	1499.8592	1499.8559	2.23	1	63	3.6e-005	1	R.TIGLETVGVKINEK.T
	545	385.2278	1536.8821	1536.8810	0.73	1	33	0.024	1	K.KLMHQAALLGQALK.D + Oxidation (M)
	546	513.3015	1536.8827	1536.8810	1.10	1	(29)	0.066	1	K.KLMHQAALLGQALK.D + Oxidation (M)
	547	769.4495	1536.8844	1536.8810	2.26	1	(31)	0.039	1	K.KLMHQAALLGQALK.D + Oxidation (M)
☑	622	809.3997	1616.7848	1616.7804	2.78	0	120	1.6e-010	1	R.WGLGGTCVNVGCIPK.K + 2 Carbamidomethyl (C)
	627	541.9441	1622.8105	1622.8086	1.14	1	7	34	2	R.SILLRGFDQDMANK.I + Oxidation (M)
	668	428.7204	1710.8525	1710.8512	0.79	1	9	22	1	K.IGEHMEEHGIKFIR.Q + Oxidation (M)

	692	442.7443	1766.9481	1766.9461	1.13	1	26	0.33	1	K.LMHQAALLGQALKDSR.N + Oxidation (M)
	706	596.3478	1786.0216	1786.0200	0.88	2	14	2.2	1	R.TIGLETVGVKINEKTGK.I
	707	447.5130	1786.0229	1786.0200	1.62	2	(1)	42	7	R.TIGLETVGVKINEKTGK.I
	750	624.3401	1869.9985	1869.9949	1.93	1	(40)	0.016	1	R.QFVPTKIEQIEAGTPGR.L
	751	936.0073	1870.0000	1869.9949	2.78	1	66	3.9e-005	1	R.QFVPTKIEQIEAGTPGR.L
	759	474.7679	1895.0425	1895.0411	0.76	2	(32)	0.066	1	K.KLMHQAALLGQALKDSR.N + Oxidation (M)
	760	632.6884	1895.0434	1895.0411	1.22	2	37	0.019	1	K.KLMHQAALLGQALKDSR.N + Oxidation (M)
☑	880	1059.4980	2116.9814	2116.9744	3.32	0	(20)	2.8	1	R.WGLGGTCVNVGCIPK.K + Carbamidomethyl (C); Maleimide-Invitrogen-Biotin+H20+O (C)
☑	927	761.4166	2281.2280	2281.2219	2.65	0	68	1.9e-005	1	R.VVGFHVLGPNAGEVTQGFAAALK.C
	952	773.3453	2317.0141	2317.0103	1.62	1	5	69	1	R.GFDQDMANKIGEHMEEHGIK.F + 2 Oxidation (M)
	1078	893.1273	2676.3601	2676.3534	2.49	0	23	1.5	1	K.IPVTDEEQTNVPYIYAIGDILEGK.L
	1101	684.3245	2733.2689	2733.2639	1.83	2	4	1.6e+002	3	R.GFDQDMANKIGEHMEEHGIKFIR.Q + 2 Oxidation (M)
☑	1119	711.1343	2840.5081	2840.5007	2.59	1	34	0.072	1	R.VVGFHVLGPNAGEVTQGFAAALKCGLTK.Q + Carbamidomethyl (C)
	1166	998.8039	2993.3899	2993.3827	2.40	1	30	0.42	1	R.YLGIPGDKEYCISSDDLFSLPYCPGK.T + 2 Carbamidomethyl (C)
☑	1218	836.1838	3340.7061	3340.6948	3.37	1	(8)	50	1	R.VVGFHVLGPNAGEVTQGFAAALKCGLTK.Q + Maleimide-Invitrogen-Biotin+H20+O (C)
	1245	1165.5380	3493.5922	3493.5768	4.41	1	(13)	25	1	R.YLGIPGDKEYCISSDDLFSLPYCPGK.T + Carbamidomethyl (C); Maleimide-Invitrogen-Biotin+H20+O (C)

4. TRXR1_BOVIN **Mass:** 54737 **Score:** 290 **Queries matched:** 26 **emPAI:** 0.79
Thioredoxin reductase 1, cytoplasmic OS=Bos taurus GN=TXNRD1 PE=2 SV=3
☐ Check to include this hit in error tolerant search or archive report

	Query	Observed	Mr(expt)	Mr(calc)	ppm	Miss	Score	Expect	Rank	Peptide
	37	412.7297	823.4448	823.4440	1.07	0	18	1.3	2	R.LYGGSTVK.C
	57	431.7375	861.4604	861.4596	0.97	0	19	1.7	2	R.YLGIPGDK.E
	147	521.2195	1040.4244	1040.4233	1.09	0	26	0.14	1	R.GFDQDMANK.I + Oxidation (M)
	169	530.7446	1059.4746	1059.4729	1.65	0	4	29	3	K.CGLTK.D + Maleimide-Invitrogen-Biotin+0 (C)
	189	539.7498	1077.4850	1077.4835	1.47	0	(4)	34	8	K.CGLTK.D + Maleimide-Invitrogen-Biotin+H20+O (C)
	237	387.2245	1158.6517	1158.6509	0.64	0	(22)	0.7	1	R.FLIATGERPR.Y
	239	580.3335	1158.6524	1158.6509	1.31	0	(19)	1.2	1	R.FLIATGERPR.Y
	240	580.3335	1158.6524	1158.6509	1.31	0	41	0.0083	1	R.FLIATGERPR.Y
	307	420.8841	1259.6305	1259.6292	1.01	1	(8)	28	4	K.VVCNIKDNER.V + Acrylamide (C)
	308	630.8229	1259.6312	1259.6292	1.63	1	26	0.42	2	K.VVCNIKDNER.V + Acrylamide (C)
	313	634.3732	1266.7318	1266.7296	1.80	0	52	0.00025	1	K.LELTPVAIQAGR.L
	622	809.3997	1616.7848	1616.7804	2.78	0	120	1.6e-010	1	R.WGLGGTCVNVGCIPK.K + 2 Carbamidomethyl (C)
	627	541.9441	1622.8105	1622.8086	1.14	1	7	34	2	R.SILLRGFDQDMANK.I + Oxidation (M)
☑	640	823.4551	1644.8956	1644.8909	2.86	0	76	3.6e-006	1	K.VMVLDFVTPTPLGTR.W
☑	655	554.6371	1660.8895	1660.8859	2.17	0	(25)	0.45	1	K.VMVLDFVTPTPLGTR.W + Oxidation (M)
	716	597.3351	1788.9835	1788.9808	1.48	1	(16)	2.8	2	K.KVMVLDFVTPTPLGTR.W + Oxidation (M)
☑	717	895.4999	1788.9852	1788.9808	2.48	1	56	0.00031	1	K.KVMVLDFVTPTPLGTR.W + Oxidation (M)
	880	1059.4980	2116.9814	2116.9744	3.32	0	(20)	2.8	1	R.WGLGGTCVNVGCIPK.K + Carbamidomethyl (C); Maleimide-Invitrogen-Biotin+H20+O (C)
☑	909	545.8005	2179.1729	2179.1711	0.81	2	(9)	18	1	K.YDKKVMVLDFVTPTPLGTR.W
☑	910	727.3991	2179.1755	2179.1711	1.99	2	27	0.26	1	K.YDKKVMVLDFVTPTPLGTR.W
	927	761.4166	2281.2280	2281.2219	2.65	0	68	1.9e-005	1	R.VVGFHVLGPNAGEVTQGFAAALK.C
	1039	645.6042	2578.3877	2578.3829	1.86	3	4	59	2	K.EAAKYDKKVMVLDFVTPTPLGTR.W
	1119	711.1343	2840.5081	2840.5007	2.59	1	34	0.072	1	R.VVGFHVLGPNAGEVTQGFAAALKCGLTK.D + Carbamidomethyl (C)
	1166	998.8039	2993.3899	2993.3827	2.40	1	30	0.42	1	R.YLGIPGDKEYCISSDDLFSLPYCPGK.T + 2 Carbamidomethyl (C)
	1218	836.1838	3340.7061	3340.6948	3.37	1	(8)	50	1	R.VVGFHVLGPNAGEVTQGFAAALKCGLTK.D + Maleimide-Invitrogen-Biotin+H20+O (C)
	1245	1165.5380	3493.5922	3493.5768	4.41	1	(13)	25	1	R.YLGIPGDKEYCISSDDLFSLPYCPGK.T + Carbamidomethyl (C); Maleimide-Invitrogen-Biotin+H20+O (C)

5. K2C1_PANTR **Mass:** 65450 **Score:** 231 **Queries matched:** 11 **emPAI:** 0.48
Keratin, type II cytoskeletal 1 OS=Pan troglodytes GN=KRT1 PE=2 SV=1
☐ Check to include this hit in error tolerant search or archive report

	Query	Observed	Mr(expt)	Mr(calc)	ppm	Miss	Score	Expect	Rank	Peptide
☑	40	416.7483	831.4820	831.4814	0.78	0	15	2.7	1	K.SISISVAR.G
☑	67	437.7537	873.4928	873.4920	1.00	0	40	0.018	1	R.SLVNLGGSK.S
☑	94	487.2703	972.5260	972.5240	2.13	0	23	0.71	1	K.IEISELNR.V

☑	181	533.2649	1064.5152	1064.5138	1.35	0	36	0.024	1	K.AQYEDIAQK.S
☑	258	590.3046	1178.5946	1178.5931	1.29	0	(53)	0.00061	1	K.YEELQITAGR.H
☑	259	590.3047	1178.5948	1178.5931	1.46	0	59	0.00015	1	K.YEELQITAGR.H
☑	338	651.8624	1301.7102	1301.7078	1.86	0	92	7.7e-008	1	R.SLDLDSIIAEVK.A
☑	372	465.2494	1392.7264	1392.7249	1.08	1	23	0.64	1	R.TNAENEFVTIKK.D
☑	426	738.3978	1474.7810	1474.7780	2.08	0	55	0.00049	1	R.FLEQQNQVLQTK.W
☑	635	546.9589	1637.8549	1637.8525	1.42	1	36	0.034	1	K.SLNNQFASFIDKVR.F
☑	691	883.3733	1764.7320	1764.7275	2.58	0	69	1.2e-005	1	R.FSSCGGGGGSFGAGGGFGSR.S + Carbamidomethyl (C)

6. K1C9_HUMAN **Mass:** 62092 **Score:** 159 **Queries matched:** 5 **emPAI:** 0.17
Keratin, type I cytoskeletal 9 OS=Homo sapiens GN=KRT9 PE=1 SV=2
☐ Check to include this hit in error tolerant search or archive report

	Query	Observed	Mr(expt)	Mr(calc)	ppm	Miss	Score	Expect	Rank	Peptide
	30	405.2239	808.4332	808.4330	0.25	0	14	4.3	2	R.LASYLDK.V
☑	180	533.2539	1064.4932	1064.4920	1.14	0	31	0.067	1	K.STMQELNSR.L
☑	289	618.2690	1234.5234	1234.5215	1.62	0	90	4.9e-008	1	R.FSSSSGYGGGSSR.V
☑	352	441.8957	1322.6653	1322.6652	0.03	1	15	4.6	1	R.IKFEMEQNLR.Q + Oxidation (M)
☑	720	896.3693	1790.7240	1790.7205	1.99	0	88	1.2e-007	1	R.GGSGGSYGGGGSGGGYGGGSGSR.G

7. K2C75_BOVIN **Mass:** 59000 **Score:** 105 **Queries matched:** 3 **emPAI:** 0.11
Keratin, type II cytoskeletal 75 OS=Bos taurus GN=KRT75 PE=2 SV=1
☐ Check to include this hit in error tolerant search or archive report

	Query	Observed	Mr(expt)	Mr(calc)	ppm	Miss	Score	Expect	Rank	Peptide
	74	453.7380	905.4614	905.4607	0.87	0	9	21	6	R.FLEQQNK.V
☑	203	547.2677	1092.5208	1092.5199	0.83	0	45	0.003	1	K.AQYEEIANR.S
	338	651.8624	1301.7102	1301.7078	1.86	0	92	7.7e-008	1	R.SLDLDSIIAEVK.A

Proteins matching the same set of peptides:
K2C7_BOVIN **Mass:** 51546 **Score:** 105 **Queries matched:** 3
Keratin, type II cytoskeletal 7 OS=Bos taurus GN=KRT7 PE=2 SV=1

8. K2C5_HUMAN **Mass:** 62340 **Score:** 96 **Queries matched:** 4 **emPAI:** 0.17
Keratin, type II cytoskeletal 5 OS=Homo sapiens GN=KRT5 PE=1 SV=3
☐ Check to include this hit in error tolerant search or archive report

	Query	Observed	Mr(expt)	Mr(calc)	ppm	Miss	Score	Expect	Rank	Peptide
	74	453.7380	905.4614	905.4607	0.87	0	9	21	6	R.FLEQQNK.V
	203	547.2677	1092.5208	1092.5199	0.83	0	45	0.003	1	K.AQYEEIANR.S
☑	216	556.2919	1110.5692	1110.5669	2.08	0	46	0.0025	1	R.ISISTSGGSFR.N
☑	353	665.3677	1328.7208	1328.7187	1.60	0	62	6.8e-005	1	R.NLDLDSIIAEVK.A

9. K22E_HUMAN **Mass:** 65825 **Score:** 95 **Queries matched:** 4 **emPAI:** 0.16
Keratin, type II cytoskeletal 2 epidermal OS=Homo sapiens GN=KRT2 PE=1 SV=1
☐ Check to include this hit in error tolerant search or archive report

	Query	Observed	Mr(expt)	Mr(calc)	ppm	Miss	Score	Expect	Rank	Peptide
	94	487.2703	972.5260	972.5240	2.13	0	23	0.71	1	K.IEISELNR.V
☑	214	554.2758	1106.5370	1106.5356	1.31	0	40	0.012	1	K.AQYEEIAQR.S
	353	665.3677	1328.7208	1328.7187	1.60	0	62	6.8e-005	1	R.NLDLDSIIAEVK.A
	426	738.3978	1474.7810	1474.7780	2.08	0	55	0.00049	1	R.FLEQQNQVLQTK.W

10. K1C10_HUMAN **Mass:** 59475 **Score:** 87 **Queries matched:** 10 **emPAI:** 0.11
Keratin, type I cytoskeletal 10 OS=Homo sapiens GN=KRT10 PE=1 SV=4

☐ Check to include this hit in error tolerant search or archive report

	Query	Observed	Mr(expt)	Mr(calc)	ppm	Miss	Score	Expect	Rank	Peptide
☑	29	404.2037	806.3928	806.3923	0.73	0	28	0.25	1	R.LAADDFR.L
	30	405.2239	808.4332	808.4330	0.25	0	14	4.3	2	R.LASYLDK.V
☑	178	355.5416	1063.6030	1063.6026	0.38	1	16	1.5	1	R.LASYLDKVR.A
☑	179	532.8088	1063.6030	1063.6026	0.45	1	(8)	10	1	R.LASYLDKVR.A
☑	200	545.7698	1089.5250	1089.5237	1.25	0	57	0.00029	1	K.VTMQNLNDR.L
☑	287	412.2316	1233.6730	1233.6717	1.04	1	28	0.16	1	R.LKYENEVALR.Q
☑	288	617.8444	1233.6742	1233.6717	2.07	1	(14)	4	1	R.LKYENEVALR.Q
☑	368	691.3286	1380.6426	1380.6408	1.31	0	63	6.1e-005	1	R.ALEESNYELEGK.I
☑	457	498.5834	1492.7284	1492.7270	0.94	1	21	1.1	1	R.SQYEQLAEQNRK.D
☑	1129	963.4701	2887.3885	2887.3804	2.79	0	10	44	1	R.NVSTGDVNVEMNAAPGVDLTQLLNNMR.S + Oxidation (M)

11. TRYP_PIG **Mass:** 24394 **Score:** 71 **Queries matched:** 6 **emPAI:** 0.67
Trypsin OS=Sus scrofa PE=1 SV=1
☐ Check to include this hit in error tolerant search or archive report

	Query	Observed	Mr(expt)	Mr(calc)	ppm	Miss	Score	Expect	Rank	Peptide
☑	46	421.7582	841.5018	841.5022	-0.36	0	(22)	0.65	1	R.VATVSLPR.S
☑	47	421.7586	841.5026	841.5022	0.59	0	45	0.0029	1	R.VATVSLPR.S
☑	48	421.7589	841.5032	841.5022	1.30	0	(33)	0.047	1	R.VATVSLPR.S
☑	151	523.2861	1044.5576	1044.5564	1.24	0	37	0.03	1	K.LSSPATLNSR.V
☑	913	737.7079	2210.1019	2210.0967	2.33	0	52	0.0016	1	R.LGEHNIDVLEGNEQFINAAK.I
☑	945	767.0651	2298.1735	2298.1678	2.47	0	26	0.62	1	K.IITHPNFNGNTLDNDIMLIK.L + Oxidation (M)

12. THIO_ECOLI **Mass:** 11799 **Score:** 62 **Queries matched:** 2 **emPAI:** 0.67
Thioredoxin-1 OS=Escherichia coli (strain K12) GN=trxA PE=1 SV=2
☐ Check to include this hit in error tolerant search or archive report

	Query	Observed	Mr(expt)	Mr(calc)	ppm	Miss	Score	Expect	Rank	Peptide
☑	112	501.3240	1000.6334	1000.6321	1.35	0	25	0.17	1	R.GIPTLLLFK.N
☑	312	634.3367	1266.6588	1266.6568	1.62	0	61	7.7e-005	1	K.LNIDQNPGTAPK.Y

Proteins matching the same set of peptides:
THIO_SALTI **Mass:** 11799 **Score:** 62 **Queries matched:** 2
Thioredoxin-1 OS=Salmonella typhi GN=trxA PE=3 SV=2
THIO_SALTY **Mass:** 11799 **Score:** 62 **Queries matched:** 2
Thioredoxin-1 OS=Salmonella typhimurium GN=trxA PE=3 SV=2
THIO_SHIFL **Mass:** 11799 **Score:** 62 **Queries matched:** 2
Thioredoxin-1 OS=Shigella flexneri GN=trxA PE=3 SV=2
THIO_ECOL6 **Mass:** 11799 **Score:** 62 **Queries matched:** 2
Thioredoxin-1 OS=Escherichia coli O6 GN=trxA PE=3 SV=2
THIO_ECO57 **Mass:** 11799 **Score:** 62 **Queries matched:** 2
Thioredoxin-1 OS=Escherichia coli O157:H7 GN=trxA PE=3 SV=2

13. THIO_HUMAN **Mass:** 11730 **Score:** 61 **Queries matched:** 5 **emPAI:** 1.16
Thioredoxin OS=Homo sapiens GN=TXN PE=1 SV=3
☐ Check to include this hit in error tolerant search or archive report

	Query	Observed	Mr(expt)	Mr(calc)	ppm	Miss	Score	Expect	Rank	Peptide
☑	246	389.1999	1164.5779	1164.5775	0.33	1	20	1.2	1	K.VGEFSGANKEK.L
☑	306	629.8491	1257.6836	1257.6816	1.63	1	48	0.0019	1	K.EKLEATINELV.-
	673	861.3749	1720.7352	1720.7299	3.09	0	3	57	2	K.CMPTFQFFK.K + Maleimide-Invitrogen-Biotin+H20+O (C); Oxidation (M)
☑	899	1074.5630	2147.1114	2147.1110	0.22	2	30	0.22	1	K.VGEFSGANKEKLEATINELV.-
☑	900	716.7125	2147.1157	2147.1110	2.19	2	(17)	3.7	1	K.VGEFSGANKEKLEATINELV.-

Proteins matching the same set of peptides:
THIO_MACMU **Mass:** 11730 **Score:** 61 **Queries matched:** 5
Thioredoxin OS=Macaca mulatta GN=TXN PE=3 SV=2

---

14. CASA1_BOVIN **Mass:** 24513 **Score:** 42 **Queries matched:** 1 **emPAI:** 0.14
Alpha-S1-casein OS=Bos taurus GN=CSN1S1 PE=1 SV=2
☐ Check to include this hit in error tolerant search or archive report

	Query	Observed	Mr(expt)	Mr(calc)	ppm	Miss	Score	Expect	Rank	Peptide
☑	369	692.8702	1383.7258	1383.7227	2.25	0	42	0.0078	1	R.FFVAPFPEVFGK.E

Proteins matching the same set of peptides:
CASA1_BUBBU **Mass:** 24311 **Score:** 42 **Queries matched:** 1
Alpha-S1-casein OS=Bubalus bubalis GN=CSN1S1 PE=2 SV=2

---

15. HAS2_XENLA **Mass:** 63643 **Score:** 35 **Queries matched:** 1 **emPAI:** 0.05
Hyaluronan synthase 2 OS=Xenopus laevis GN=has2 PE=2 SV=2
☐ Check to include this hit in error tolerant search or archive report

	Query	Observed	Mr(expt)	Mr(calc)	ppm	Miss	Score	Expect	Rank	Peptide
☑	43	419.7269	837.4392	837.4344	5.73	0	35	0.027	1	K.SSFASALR.G

Peptide matches not assigned to protein hits: (no details means no match)

	Query	Observed	Mr(expt)	Mr(calc)	ppm	Miss	Score	Expect	Rank	Peptide
☑	746	927.4936	1852.9726	1852.9615	5.99	2	34	0.074	1	AERPTQINEGAEARRR
☑	579	780.8973	1559.7800	1559.7726	4.78	0	33	0.075	1	TVVCVISGGNNDINR
☑	471	501.9263	1502.7571	1502.7585	-0.96	0	32	0.089	1	EVCDQLVQMLVR + Acrylamide (C)
☑	333	649.3496	1296.6846	1296.6826	1.57	1	31	0.073	1	KFSPIYSQATR
☑	666	852.4575	1702.9004	1702.9036	-1.84	1	31	0.14	1	NLCLLKTSSSGLPSAR + Carbamidomethyl (C)
☑	26	396.7346	791.4546	791.4575	-3.57	2	30	0.11	1	IKKEMK + Oxidation (M)
☑	245	582.2803	1162.5460	1162.5466	-0.44	2	29	0.14	1	ETEKERDEK
☑	346	657.3474	1312.6802	1312.6847	-3.41	1	29	0.15	1	KANEIAGNNGIGR
☑	861	687.0177	2058.0313	2058.0303	0.49	0	28	0.37	1	TEAMALLQEEANLEEIVR
☑	355	670.3022	1338.5898	1338.5842	4.19	2	27	0.13	1	KCRECCGGIGK + Acrylamide (C); Carbamidomethyl (C); Carboxymethyl (C)
☑	916	746.3797	2236.1173	2236.1166	0.29	3	24	0.9	1	CTSPKQCLPACKEKFGIAAGK + Carbamidomethyl (C)
☑	53	426.2185	850.4224	850.4185	4.68	0	24	0.43	1	LTDFAER
☑	797	648.3671	1942.0795	1942.0888	-4.78	1	23	0.42	1	LVLSSGKVSIDISVHFNK
☑	197	545.2577	1088.5008	1088.5033	-2.24	0	23	0.34	1	ANGCVANGEVR
☑	37	412.7297	823.4448	823.4440	1.07	0	23	0.43	1	LYGSVGTK
☑	390	715.3943	1428.7740	1428.7799	-4.08	0	23	0.7	1	WLCIELIGQLNK
☑	57	431.7375	861.4604	861.4596	0.97	0	23	0.81	1	YLVAPDGK
☑	252	586.8061	1171.5976	1171.5986	-0.80	0	23	0.54	1	SSWPTVPGSVR
☑	470	501.9262	1502.7568	1502.7585	-1.16	0	22	0.93	1	EVCDQLVQMLVR + Acrylamide (C)
☑	302	626.3593	1250.7040	1250.7023	1.41	0	22	0.46	1	YIPIQYVLSR
☑	265	601.3387	1200.6628	1200.6575	4.48	1	22	0.82	1	LTGRTQEIQR
☑	30	405.2239	808.4332	808.4330	0.25	0	22	0.71	1	LASLYDK
☑	278	608.3538	1214.6930	1214.6870	4.96	1	22	0.87	1	LDKVISELNGK
☑	56	428.7664	855.5182	855.5178	0.53	0	21	0.63	1	SPTVIALR
☑	51	425.7374	849.4602	849.4630	-3.24	0	21	1.2	1	IMVTSVGK + Oxidation (M)
☑	855	1023.4910	2044.9674	2044.9605	3.42	2	21	2.1	1	AQCLTTCRSVRER + Maleimide-Invitrogen-Biotin (C)
☑	262	596.8201	1191.6256	1191.6248	0.74	0	20	1.1	1	GALEAYVQSVR
☑	540	768.3817	1534.7488	1534.7418	4.59	1	20	1.6	1	ACMNMGTLSGAPKVR
☑	667	852.4576	1702.9006	1702.9036	-1.72	1	20	1.6	1	NLCLLKTSSSGLPSAR + Carbamidomethyl (C)
☑	713	894.9922	1787.9698	1787.9669	1.65	1	20	1.5	1	EYQIPTGIVDILGKDK
☑	277	607.3397	1212.6648	1212.6588	4.99	3	20	0.94	1	RTGREWRRP
☑	494	758.3674	1514.7202	1514.7260	-3.77	1	19	1.6	1	CVSNIEGPGEQRR + Acrylamide (C)

☑	153	524.3140	1046.6134	1046.6124	0.99	1	19	0.7	1	VKQLYPATK
☑	36	409.7426	817.4706	817.4731	-3.03	1	19	1.6	1	IKEGMIK
☑	371	694.8502	1387.6858	1387.6884	-1.87	1	19	1.5	1	WRWTTAEDPVK
☑	110	500.2832	998.5518	998.5509	0.96	0	19	0.78	1	GALIQDVQR
☑	859	1029.0000	2055.9854	2055.9969	-5.57	1	19	3.4	1	EGKCVQLIQGDPTK + Maleimide-Invitrogen-Biotin+H20 (C)
☑	103	495.7670	989.5194	989.5215	-2.10	0	19	2.2	1	MENLNLIK + Oxidation (M)
☑	414	727.8865	1453.7584	1453.7565	1.34	0	19	1.6	1	IIEINPEEGWVR
☑	52	425.7376	849.4606	849.4630	-2.73	1	19	1.8	1	LTSCLKK + Carboxymethyl (C)
☑	82	467.7323	933.4500	933.4556	-5.94	0	19	1.2	1	QQYGPVDK
☑	129	511.2899	1020.5652	1020.5603	4.80	1	18	1.3	1	LENNIKYK
☑	458	747.3763	1492.7380	1492.7377	0.20	2	18	2.2	1	ENCMEIIKDVKR + Oxidation (M)
☑	201	546.7703	1091.5260	1091.5248	1.19	0	18	1.5	1	DPEFSVGSVR
☑	824	668.6574	2002.9504	2002.9452	2.58	0	18	3.7	1	ENDCLLIVDEVQCGIGR + Acrylamide (C); Carbamidomethyl (C)
☑	725	901.9500	1801.8854	1801.8880	-1.43	1	18	3.2	1	AADVVVDCTPGKVGATNK + Carboxymethyl (C)
☑	348	657.8396	1313.6646	1313.6584	4.77	0	18	1.8	1	TQCLEIIMHR + Acrylamide (C)
☑	562	774.8514	1547.6882	1547.6894	-0.77	0	18	2	1	MICSPQNNTGAPMK + Carbamidomethyl (C)
☑	870	1036.9970	2071.9794	2071.9918	-5.97	1	18	4.6	1	EGKCVQLIQGDPTK + Maleimide-Invitrogen-Biotin+H20+O (C)
☑	257	589.8126	1177.6106	1177.6091	1.29	0	18	2.1	1	LSQVTDAFAAR
☑	553	515.6016	1543.7830	1543.7850	-1.34	2	18	3	1	CKIKEGAVR + Maleimide-Invitrogen-Biotin+H20 (C)
☑	70	450.2693	898.5240	898.5236	0.51	0	17	1.4	1	QAEVLALR
☑	524	510.2701	1527.7885	1527.7794	5.96	2	17	2.5	1	FDHKDQIDKINR
☑	383	706.8817	1411.7488	1411.7459	2.06	2	17	2.2	1	SDAKDIGFIKYR
☑	84	468.7018	935.3890	935.3906	-1.64	0	17	3.5	1	EIECEDK + Acrylamide (C)
☑	583	782.3798	1562.7450	1562.7437	0.83	1	17	2.9	1	RDSQGFDADGIGAVR
☑	504	506.9299	1517.7679	1517.7712	-2.20	2	17	3.4	1	DTARHHRNWAVR
☑	318	641.8140	1281.6134	1281.6135	-0.07	1	17	2.2	1	SAPYSMIDRSR
☑	244	388.5223	1162.5451	1162.5515	-5.50	2	17	2.3	1	KMFSCEFKK + Oxidation (M)
☑	716	597.3351	1788.9835	1788.9767	3.78	3	17	2.7	1	AADLLEQTIMEKRKK + Oxidation (M)
☑	159	525.7650	1049.5154	1049.5141	1.24	0	16	2.8	1	LADINYEGR
☑	729	902.9897	1803.9648	1803.9553	5.31	2	16	3.3	1	TACHFVSKKTEELLK + Acrylamide (C)
☑	528	765.3950	1528.7754	1528.7845	-5.92	1	16	3.8	1	DEALTAVADALRER
☑	711	894.9922	1787.9698	1787.9669	1.65	1	16	3.2	1	EYQIPTGIVDILGKDK
☑	296	622.8365	1243.6584	1243.6594	-0.80	1	16	3	1	QMLLNKDGTPK
☑	839	677.0021	2027.9845	2027.9880	-1.76	1	16	5.9	1	CLIRNEANATQVR + Maleimide-Invitrogen-Biotin+H20 (C)
☑	199	545.7371	1089.4596	1089.4583	1.24	0	16	1.4	1	CNLACTPPR + 2 Carboxymethyl (C)
☑	402	719.8900	1437.7654	1437.7650	0.34	1	16	3.1	1	SVAMQYLELTKR
☑	293	622.3443	1242.6740	1242.6754	-1.11	0	16	3.2	1	LVCLLPSSQAR + Carbamidomethyl (C)
☑	415	727.8867	1453.7588	1453.7599	-0.70	1	16	2.8	1	SVAMQYLELTKR + Oxidation (M)
☑	13	378.1812	754.3478	754.3472	0.82	0	16	0.56	1	WMFQK + Oxidation (M)
☑	375	701.8585	1401.7024	1401.7108	-5.97	1	16	4	1	CREATASLMPPVK
☑	79	464.7645	927.5144	927.5138	0.72	0	16	1.8	1	IGAQIDGVR
☑	3	351.2316	700.4486	700.4483	0.47	0	16	5	1	GALTVLK
☑	74	453.7380	905.4614	905.4640	-2.84	1	15	4.6	1	MEKGSNLK
☑	589	786.8976	1571.7806	1571.7726	5.14	1	15	4.9	1	SSVSCQKHAASAPTK + Acrylamide (C)
☑	580	780.8987	1559.7828	1559.7878	-3.19	1	15	5	1	ICKNITWASNPTR + Carbamidomethyl (C)
☑	83	468.7017	935.3888	935.3906	-1.85	0	15	5.4	1	EIECEDK + Acrylamide (C)
☑	459	747.3764	1492.7382	1492.7377	0.34	2	15	4.6	1	ENCMEIIKDVKR + Oxidation (M)
☑	77	458.2419	914.4692	914.4723	-3.29	0	15	1.9	1	QVQHTFR
☑	190	540.2780	1078.5414	1078.5441	-2.46	0	15	3	1	QTMLTQSVR + Oxidation (M)
☑	499	506.2674	1515.7804	1515.7794	0.67	2	15	5.4	1	DSNVNWNNLKGKK
☑	272	604.3259	1206.6372	1206.6357	1.32	1	15	3.4	1	ANAVIKSYGER
☑	796	648.3666	1942.0780	1942.0888	-5.55	1	15	3.4	1	LVLSSGKVSIDISVHFNK
☑	98	490.2846	978.5546	978.5538	0.83	0	15	2.4	1	FALPQYLK
☑	145	517.2263	1032.4380	1032.4407	-2.54	0	15	2.2	1	NMNNTNGPR + Oxidation (M)
☑	243	388.5223	1162.5451	1162.5515	-5.50	2	15	3.5	1	KMFSCEFKK + Oxidation (M)
☑	172	532.2950	1062.5754	1062.5709	4.26	0	15	3.3	1	EAALIYLDR
☑	871	1037.4970	2072.9794	2072.9797	-0.11	0	15	8.9	1	SSGQSAQLLSQEAGDPPMVR + Oxidation (M)
☑	174	532.7728	1063.5310	1063.5332	-2.01	0	15	4.2	1	ITVCNITSR + Carboxymethyl (C)
☑	441	743.8188	1485.6230	1485.6163	4.55	2	15	2.3	1	KGGRCCCFSPDGK + Acrylamide (C); Carboxymethyl (C)

SI Table 2C b2gpI/TRX-1/TRX-R/NADPH

Mascot Search Results

```
User          :
Email         :
Search title  : Freda_13_11_08_3.RAW relex2
MS data file  : D:\Data\Mark\13_11_08\Freda_13_11_08_3.RAW
Database      : Sprot 19_12_08 (402482 sequences; 145232059 residues)
Timestamp     : 11 Mar 2009 at 03:50:11 GMT
Protein hits  : APOH_HUMAN  Beta-2-glycoprotein 1 OS=Homo sapiens GN=APOH PE=1 SV=3
                TRXR1_RAT   Thioredoxin reductase 1, cytoplasmic OS=Rattus norvegicus GN=Txnrd1 PE=1 SV=4
                APOH_CANFA  Beta-2-glycoprotein 1 OS=Canis familiaris GN=APOH PE=2 SV=1
                TRXR1_BOVIN Thioredoxin reductase 1, cytoplasmic OS=Bos taurus GN=TXNRD1 PE=2 SV=3
                K2C1_HUMAN  Keratin, type II cytoskeletal 1 OS=Homo sapiens GN=KRT1 PE=1 SV=5
                K22E_HUMAN  Keratin, type II cytoskeletal 2 epidermal OS=Homo sapiens GN=KRT2 PE=1 SV=1
                THIO_HUMAN  Thioredoxin OS=Homo sapiens GN=TXN PE=1 SV=3
                LACB_BOVIN  Beta-lactoglobulin OS=Bos taurus GN=LGB PE=1 SV=3
                K1C10_HUMAN Keratin, type I cytoskeletal 10 OS=Homo sapiens GN=KRT10 PE=1 SV=4
                TRYP_PIG    Trypsin OS=Sus scrofa PE=1 SV=1
                K1C9_HUMAN  Keratin, type I cytoskeletal 9 OS=Homo sapiens GN=KRT9 PE=1 SV=2
                CASB_BOVIN  Beta-casein OS=Bos taurus GN=CSN2 PE=1 SV=2
                LACB_OVIMU  Beta-lactoglobulin OS=Ovis orientalis musimon GN=LGB PE=1 SV=1
                IPSP_HUMAN  Plasma serine protease inhibitor OS=Homo sapiens GN=SERPINA5 PE=1 SV=2
                THIO_ECOLI  Thioredoxin-1 OS=Escherichia coli (strain K12) GN=trxA PE=1 SV=2
                PROP_HUMAN  Properdin OS=Homo sapiens GN=CFP PE=1 SV=2
                APOH_RAT    Beta-2-glycoprotein 1 OS=Rattus norvegicus GN=Apoh PE=2 SV=2
                IGHG1_HUMAN Ig gamma-1 chain C region OS=Homo sapiens GN=IGHG1 PE=1 SV=1
                CASA1_BOVIN Alpha-S1-casein OS=Bos taurus GN=CSN1S1 PE=1 SV=2
                CASK_BOVIN  Kappa-casein OS=Bos taurus GN=CSN3 PE=1 SV=1
                HAS2_XENLA  Hyaluronan synthase 2 OS=Xenopus laevis GN=has2 PE=2 SV=2
                Y1720_HAEIN Uncharacterized protein HI1720 OS=Haemophilus influenzae GN=HI1720 PE=3 SV=1
```

Probability Based Mowse Score

Ions score is -10*Log(P), where P is the probability that the observed match is a random event.
Individual ions scores > 32 indicate identity or extensive homology (p<0.05).
Protein scores are derived from ions scores as a non-probabilistic basis for ranking protein hits.

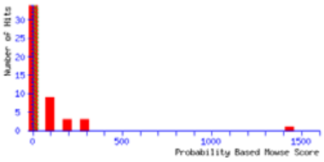

Peptide Summary Report

Format As [Peptide Summary] Help

Significance threshold p< 0.05 Max. number of hits AUTO

Standard scoring ○ MudPIT scoring ◉ Ions score or expect cut-off 1 Show sub-sets 0

Show pop-ups ◉ Suppress pop-ups ○ Sort unassigned [Decreasing Score] Require bold red ☑

Select All | Select None | Search Selected | ☐ **Error tolerant** | Archive Report

1. APOH_HUMAN **Mass:** 38273 **Score:** 1430 **Queries matched:** 117 **emPAI:** 28.94
Beta-2-glycoprotein 1 OS=Homo sapiens GN=APOH PE=1 SV=3
☐ Check to include this hit in error tolerant search or archive report

	Query	Observed	Mr(expt)	Mr(calc)	ppm	Miss	Score	Expect	Rank	Peptide
☑	23	394.1943	786.3740	786.3735	0.75	0	25	0.098	1	K.VSFFCK.N + Carbamidomethyl (C)
	30	396.7346	791.4546	791.4541	0.67	1	30	0.095	4	K.IQEKFK.N
☑	79	436.2087	870.4028	870.4018	1.23	0	35	0.0099	1	K.NGMLHGDK.V
☑	92	451.7604	901.5062	901.5055	0.82	1	25	0.21	1	K.ASCKVPVK.K + Acrylamide (C)
☑	115	468.2112	934.4078	934.4066	1.33	0	(40)	0.0022	1	K.TDASDVKPC.-
☑	142	496.7216	991.4286	991.4281	0.58	0	59	3.3e-005	1	K.TDASDVKPC.- + Carbamidomethyl (C)
☑	143	496.7216	991.4286	991.4281	0.58	0	(35)	0.0083	1	K.TDASDVKPC.- + Carbamidomethyl (C)
☑	144	496.7218	991.4290	991.4281	0.99	0	(46)	0.00061	1	K.TDASDVKPC.- + Carbamidomethyl (C)
☑	180	511.7671	1021.5196	1021.5193	0.38	0	(50)	0.00082	1	K.ATVVYQGER.V
☑	181	511.7673	1021.5200	1021.5193	0.77	0	(6)	19	1	K.ATVVYQGER.V
☑	182	511.7674	1021.5202	1021.5193	0.97	0	(42)	0.0051	1	K.ATVVYQGER.V
☑	183	511.7674	1021.5202	1021.5193	0.97	0	(40)	0.0068	1	K.ATVVYQGER.V
☑	184	511.7674	1021.5202	1021.5193	0.97	0	(50)	0.00072	1	K.ATVVYQGER.V
☑	185	511.7674	1021.5202	1021.5193	0.97	0	57	0.00016	1	K.ATVVYQGER.V
☑	186	511.7674	1021.5202	1021.5193	0.97	0	(50)	0.00076	1	K.ATVVYQGER.V
☑	187	511.7674	1021.5202	1021.5193	0.97	0	(41)	0.0063	1	K.ATVVYQGER.V
☑	300	368.8542	1103.5408	1103.5400	0.72	0	(18)	2.2	1	K.EHSSLAFWK.T
☑	301	552.7780	1103.5414	1103.5400	1.33	0	20	1.3	1	K.EHSSLAFWK.T
☑	345	573.7905	1145.5664	1145.5652	1.13	1	(15)	2.5	1	K.FKNGMLHGDK.V
☑	352	384.2122	1149.6148	1149.6142	0.49	1	(13)	3.5	1	K.KATVVYQGER.V
☑	354	575.8148	1149.6150	1149.6142	0.73	1	54	0.00027	1	K.KATVVYQGER.V
☑	355	575.8148	1149.6150	1149.6142	0.73	1	(41)	0.0056	1	K.KATVVYQGER.V
☑	356	384.2123	1149.6151	1149.6142	0.75	1	(13)	3.2	1	K.KATVVYQGER.V
☑	357	384.2123	1149.6151	1149.6142	0.75	1	(34)	0.027	1	K.KATVVYQGER.V
☑	358	575.8152	1149.6158	1149.6142	1.42	1	(8)	10	1	K.KATVVYQGER.V
☑	366	388.1942	1161.5608	1161.5601	0.60	1	(25)	0.25	1	K.FKNGMLHGDK.V + Oxidation (M)
☑	367	388.1943	1161.5611	1161.5601	0.86	1	(19)	1	1	K.FKNGMLHGDK.V + Oxidation (M)
☑	368	581.7882	1161.5618	1161.5601	1.53	1	32	0.051	1	K.FKNGMLHGDK.V + Oxidation (M)
☑	470	625.3493	1248.6840	1248.6826	1.13	1	18	0.86	1	K.ATVVYQGERVK.I
☑	485	633.7847	1265.5548	1265.5533	1.25	0	34	0.022	1	K.LGNWSAMPSCK.A + Carbamidomethyl (C); Oxidation (M)
☑	590	490.2568	1467.7486	1467.7470	1.08	0	32	0.045	1	R.VYKPSAGNNSLYR.D
☑	622	501.5981	1501.7725	1501.7711	0.89	0	(42)	0.0054	1	R.VCPFAGILENGAVR.Y + Carbamidomethyl (C)
☑	623	751.8937	1501.7728	1501.7711	1.14	0	(32)	0.061	1	R.VCPFAGILENGAVR.Y + Carbamidomethyl (C)
☑	624	751.8938	1501.7730	1501.7711	1.28	0	(42)	0.0052	1	R.VCPFAGILENGAVR.Y + Carbamidomethyl (C)
☑	625	751.8939	1501.7732	1501.7711	1.41	0	63	4e-005	1	R.VCPFAGILENGAVR.Y + Carbamidomethyl (C)
☑	626	751.8940	1501.7734	1501.7711	1.54	0	(24)	0.35	1	R.VCPFAGILENGAVR.Y + Carbamidomethyl (C)
☑	627	751.8942	1501.7738	1501.7711	1.81	0	(45)	0.0028	1	R.VCPFAGILENGAVR.Y + Carbamidomethyl (C)
☑	628	751.8946	1501.7746	1501.7711	2.34	0	(32)	0.06	1	R.VCPFAGILENGAVR.Y + Carbamidomethyl (C)
☑	629	751.8948	1501.7750	1501.7711	2.61	0	(26)	0.25	1	R.VCPFAGILENGAVR.Y + Carbamidomethyl (C)
☑	630	751.8949	1501.7752	1501.7711	2.74	0	(44)	0.0039	1	R.VCPFAGILENGAVR.Y + Carbamidomethyl (C)
☑	631	751.8951	1501.7756	1501.7711	3.01	0	(16)	2.6	1	R.VCPFAGILENGAVR.Y + Carbamidomethyl (C)
☑	632	751.8956	1501.7766	1501.7711	3.67	0	(38)	0.014	1	R.VCPFAGILENGAVR.Y + Carbamidomethyl (C)
☑	693	513.9190	1538.7352	1538.7340	0.76	1	(19)	1.1	1	K.CFKEHSSLAFWK.T + Carbamidomethyl (C)
☑	694	770.3760	1538.7374	1538.7340	2.23	1	44	0.0032	1	K.CFKEHSSLAFWK.T + Carbamidomethyl (C)
☑	775	547.2628	1638.7666	1638.7647	1.16	1	(28)	0.16	1	K.NGMLHGDKVSFFCK.N + Carbamidomethyl (C)
☑	792	414.6975	1654.7609	1654.7596	0.78	1	(2)	57	1	K.NGMLHGDKVSFFCK.N + Carbamidomethyl (C); Oxidation (M)
☑	793	552.5943	1654.7611	1654.7596	0.89	1	(31)	0.089	1	K.NGMLHGDKVSFFCK.N + Carbamidomethyl (C); Oxidation (M)
☑	794	414.6977	1654.7617	1654.7596	1.27	1	(7)	18	1	K.NGMLHGDKVSFFCK.N + Carbamidomethyl (C); Oxidation (M)
☑	795	828.3883	1654.7620	1654.7596	1.48	1	56	0.00022	1	K.NGMLHGDKVSFFCK.N + Carbamidomethyl (C); Oxidation (M)
☑	867	886.9935	1771.9724	1771.9695	1.66	0	72	2.4e-006	1	K.FICPLTGLWPINTLK.C + Carbamidomethyl (C)
☑	868	886.9937	1771.9728	1771.9695	1.89	0	(62)	2.6e-005	1	K.FICPLTGLWPINTLK.C + Carbamidomethyl (C)
☑	869	591.6649	1771.9729	1771.9695	1.90	0	(34)	0.018	1	K.FICPLTGLWPINTLK.C + Carbamidomethyl (C)
☑	870	886.9941	1771.9736	1771.9695	2.34	0	(66)	1.1e-005	1	K.FICPLTGLWPINTLK.C + Carbamidomethyl (C)
☑	871	591.6652	1771.9738	1771.9695	2.41	0	(38)	0.006	1	K.FICPLTGLWPINTLK.C + Carbamidomethyl (C)
☑	872	886.9943	1771.9740	1771.9695	2.57	0	(53)	0.00022	1	K.FICPLTGLWPINTLK.C + Carbamidomethyl (C)
☑	873	886.9944	1771.9742	1771.9695	2.68	0	(34)	0.016	1	K.FICPLTGLWPINTLK.C + Carbamidomethyl (C)
☑	874	886.9946	1771.9746	1771.9695	2.91	0	(63)	2.1e-005	1	K.FICPLTGLWPINTLK.C + Carbamidomethyl (C)
☑	987	634.3612	1900.0618	1900.0645	-1.41	1	(11)	1.9	1	R.KFICPLTGLWPINTLK.C + Carbamidomethyl (C)

☑	988	634.3629	1900.0669	1900.0645	1.27	1	(16)	0.47	1	R.KFICPLTGLWPINTLK.C + Carbamidomethyl (C)
☑	989	634.3632	1900.0678	1900.0645	1.75	1	(29)	0.025	1	R.KFICPLTGLWPINTLK.C + Carbamidomethyl (C)
☑	990	634.3633	1900.0681	1900.0645	1.90	1	(21)	0.16	1	R.KFICPLTGLWPINTLK.C + Carbamidomethyl (C)
☑	991	951.0417	1900.0688	1900.0645	2.31	1	(51)	0.00015	1	R.KFICPLTGLWPINTLK.C + Carbamidomethyl (C)
☑	992	951.0420	1900.0694	1900.0645	2.63	1	51	0.00012	1	R.KFICPLTGLWPINTLK.C + Carbamidomethyl (C)
☑	1005	638.6733	1912.9981	1912.9969	0.64	0	(37)	0.013	1	R.TCPKPDDLPFSTVVPLK.T + Carbamidomethyl (C)
☑	1006	957.5065	1912.9984	1912.9969	0.83	0	41	0.005	1	R.TCPKPDDLPFSTVVPLK.T + Carbamidomethyl (C)
☑	1007	638.6736	1912.9990	1912.9969	1.11	0	(31)	0.045	1	R.TCPKPDDLPFSTVVPLK.T + Carbamidomethyl (C)
☑	1008	638.6739	1912.9999	1912.9969	1.58	0	(31)	0.051	1	R.TCPKPDDLPFSTVVPLK.T + Carbamidomethyl (C)
☑	1009	638.6752	1913.0038	1912.9969	3.61	0	(26)	0.2	1	R.TCPKPDDLPFSTVVPLK.T + Carbamidomethyl (C)
☑	1169	695.9760	2084.9062	2084.9031	1.48	0	(38)	0.015	1	K.CSYTEDAQCIDGTIEVPK.C + 2 Carbamidomethyl (C)
☑	1170	1043.4620	2084.9094	2084.9031	3.06	0	(7)	21	1	K.CSYTEDAQCIDGTIEVPK.C + 2 Carbamidomethyl (C)
☑	1171	1043.4620	2084.9094	2084.9031	3.06	0	82	5.9e-007	1	K.CSYTEDAQCIDGTIEVPK.C + 2 Carbamidomethyl (C)
☑	1172	1043.4630	2084.9114	2084.9031	4.02	0	(66)	2.6e-005	1	K.CSYTEDAQCIDGTIEVPK.C + 2 Carbamidomethyl (C)
☑	1173	1043.4630	2084.9114	2084.9031	4.02	0	(24)	0.44	1	K.CSYTEDAQCIDGTIEVPK.C + 2 Carbamidomethyl (C)
☑	1258	738.6747	2213.0023	2212.9980	1.92	1	(24)	0.45	1	K.KCSYTEDAQCIDGTIEVPK.C + 2 Carbamidomethyl (C)
☑	1259	738.6749	2213.0029	2212.9980	2.19	1	(49)	0.0015	1	K.KCSYTEDAQCIDGTIEVPK.C + 2 Carbamidomethyl (C)
☑	1260	1107.5090	2213.0034	2212.9980	2.45	1	78	1.8e-006	1	K.KCSYTEDAQCIDGTIEVPK.C + 2 Carbamidomethyl (C)
☑	1275	763.0765	2286.2077	2286.2017	2.61	1	12	3.3	1	K.FICPLTGLWPINTLKCTPR.V + 2 Carbamidomethyl (C)
☑	1276	763.0768	2286.2086	2286.2017	3.00	1	(8)	8.7	1	K.FICPLTGLWPINTLKCTPR.V + 2 Carbamidomethyl (C)
☑	1294	795.0364	2382.0874	2382.0838	1.50	0	(21)	0.95	1	K.TFYEPGEEITYSCKPGYVSR.G + Carbamidomethyl (C)
☑	1295	795.0365	2382.0877	2382.0838	1.63	0	(5)	41	1	K.TFYEPGEEITYSCKPGYVSR.G + Carbamidomethyl (C)
☑	1296	795.0367	2382.0883	2382.0838	1.88	0	(9)	18	1	K.TFYEPGEEITYSCKPGYVSR.G + Carbamidomethyl (C)
☑	1297	795.0367	2382.0883	2382.0838	1.88	0	(2)	82	1	K.TFYEPGEEITYSCKPGYVSR.G + Carbamidomethyl (C)
☑	1298	795.0368	2382.0886	2382.0838	2.00	0	(7)	26	1	K.TFYEPGEEITYSCKPGYVSR.G + Carbamidomethyl (C)
☑	1299	795.0368	2382.0886	2382.0838	2.00	0	(7)	26	1	K.TFYEPGEEITYSCKPGYVSR.G + Carbamidomethyl (C)
☑	1300	795.0369	2382.0889	2382.0838	2.13	0	(12)	8.6	1	K.TFYEPGEEITYSCKPGYVSR.G + Carbamidomethyl (C)
☑	1301	795.0372	2382.0898	2382.0838	2.51	0	(10)	14	1	K.TFYEPGEEITYSCKPGYVSR.G + Carbamidomethyl (C)
☑	1302	795.0374	2382.0904	2382.0838	2.76	0	(4)	48	1	K.TFYEPGEEITYSCKPGYVSR.G + Carbamidomethyl (C)
☑	1303	795.0375	2382.0907	2382.0838	2.88	0	(2)	86	1	K.TFYEPGEEITYSCKPGYVSR.G + Carbamidomethyl (C)
☑	1304	795.0377	2382.0913	2382.0838	3.14	0	(7)	26	1	K.TFYEPGEEITYSCKPGYVSR.G + Carbamidomethyl (C)
☑	1305	795.0377	2382.0913	2382.0838	3.14	0	23	0.69	1	K.TFYEPGEEITYSCKPGYVSR.G + Carbamidomethyl (C)
☑	1306	795.0378	2382.0916	2382.0838	3.26	0	(10)	12	1	K.TFYEPGEEITYSCKPGYVSR.G + Carbamidomethyl (C)
☑	1307	795.0378	2382.0916	2382.0838	3.26	0	(10)	12	1	K.TFYEPGEEITYSCKPGYVSR.G + Carbamidomethyl (C)
☑	1308	795.0384	2382.0934	2382.0838	4.02	0	(3)	66	1	K.TFYEPGEEITYSCKPGYVSR.G + Carbamidomethyl (C)
☑	1309	796.0052	2384.9938	2384.9889	2.03	0	(40)	0.008	1	K.ATFGCHDGYSLDGPEEIECTK.L + 2 Carbamidomethyl (C)
☑	1310	1193.5050	2384.9954	2384.9889	2.73	0	65	2.7e-005	1	K.ATFGCHDGYSLDGPEEIECTK.L + 2 Carbamidomethyl (C)
☑	1313	799.7083	2396.1031	2396.0994	1.51	0	(13)	7.2	1	K.TFYEPGEEITYSCKPGYVSR.G + Acrylamide (C)
☑	1314	799.7086	2396.1040	2396.0994	1.89	0	(13)	6	1	K.TFYEPGEEITYSCKPGYVSR.G + Acrylamide (C)
☑	1378	658.0365	2628.1169	2628.1108	2.30	1	(17)	1.8	1	K.DKATFGCHDGYSLDGPEEIECTK.L + 2 Carbamidomethyl (C)
☑	1379	877.0463	2628.1171	2628.1108	2.37	1	(48)	0.0015	1	K.DKATFGCHDGYSLDGPEEIECTK.L + 2 Carbamidomethyl (C)
☑	1380	877.0472	2628.1198	2628.1108	3.40	1	52	0.00068	1	K.DKATFGCHDGYSLDGPEEIECTK.L + 2 Carbamidomethyl (C)
☑	1420	683.5900	2730.3309	2730.3264	1.64	0	(8)	21	1	K.CPFPSRPDNGFVNYPAKPTLYYK.D + Carbamidomethyl (C)
☑	1421	911.1180	2730.3322	2730.3264	2.10	0	31	0.1	1	K.CPFPSRPDNGFVNYPAKPTLYYK.D + Carbamidomethyl (C)
☑	1441	940.4949	2818.4629	2818.4550	2.79	0	28	0.12	1	K.WSPELPVCAPIICPPPSIPTFATLR.V + 2 Carbamidomethyl (C)
☑	1442	1410.2410	2818.4674	2818.4550	4.41	0	(28)	0.11	1	K.WSPELPVCAPIICPPPSIPTFATLR.V + 2 Carbamidomethyl (C)
☑	1480	744.3700	2973.4509	2973.4483	0.86	1	(17)	2.2	1	K.CPFPSRPDNGFVNYPAKPTLYYKDK.A + Carbamidomethyl (C)
☑	1481	595.6976	2973.4516	2973.4483	1.11	1	(26)	0.31	1	K.CPFPSRPDNGFVNYPAKPTLYYKDK.A + Carbamidomethyl (C)
☑	1482	992.1584	2973.4534	2973.4483	1.69	1	33	0.053	1	K.CPFPSRPDNGFVNYPAKPTLYYKDK.A + Carbamidomethyl (C)
☑	1499	1023.4600	3067.3582	3067.3546	1.17	0	(44)	0.0056	1	R.YTTFEYPNTISFSCNTGFYLNGADSAK.C + Carbamidomethyl (C)
☑	1500	1534.6910	3067.3674	3067.3546	4.20	0	60	0.00015	1	R.YTTFEYPNTISFSCNTGFYLNGADSAK.C + Carbamidomethyl (C)
☑	1510	772.6425	3086.5409	3086.5324	2.75	1	2	61	1	R.EVKCPFPSRPDNGFVNYPAKPTLYYK.D + Carbamidomethyl (C)
☑	1594	1175.2510	3522.7312	3522.7350	-1.07	1	(31)	0.081	1	K.CTEEGKWSPELPVCAPIICPPPSIPTFATLR.V + 3 Carbamidomethyl (C)
☑	1595	705.5551	3522.7391	3522.7350	1.18	1	(22)	0.74	1	K.CTEEGKWSPELPVCAPIICPPPSIPTFATLR.V + 3 Carbamidomethyl (C)
☑	1596	1175.2550	3522.7432	3522.7350	2.33	1	(46)	0.0027	1	K.CTEEGKWSPELPVCAPIICPPPSIPTFATLR.V + 3 Carbamidomethyl (C)
☑	1597	881.6937	3522.7457	3522.7350	3.05	1	(24)	0.4	1	K.CTEEGKWSPELPVCAPIICPPPSIPTFATLR.V + 3 Carbamidomethyl (C)
☑	1598	1175.2560	3522.7462	3522.7350	3.18	1	63	5.5e-005	1	K.CTEEGKWSPELPVCAPIICPPPSIPTFATLR.V + 3 Carbamidomethyl (C)
☑	1599	1175.2560	3522.7462	3522.7350	3.18	1	(33)	0.056	1	K.CTEEGKWSPELPVCAPIICPPPSIPTFATLR.V + 3 Carbamidomethyl (C)
☑	1600	881.6945	3522.7489	3522.7350	3.96	1	(1)	72	1	K.CTEEGKWSPELPVCAPIICPPPSIPTFATLR.V + 3 Carbamidomethyl (C)

2. TRXR1_RAT **Mass:** 54352 **Score:** 291 **Queries matched:** 16 **emPAI:** 1.02

Thioredoxin reductase 1, cytoplasmic OS=Rattus norvegicus GN=Txnrd1 PE=1 SV=4

☐ Check to include this hit in error tolerant search or archive report

	Query	Observed	Mr(expt)	Mr(calc)	ppm	Miss	Score	Expect	Rank	Peptide
	46	412.7296	823.4446	823.4440	0.82	0	13	3.2	2	R.LYGGSTVK.C
☑	72	431.7374	861.4602	861.4596	0.74	0	19	1.5	1	R.YLGIPGDK.E
☑	167	508.8035	1015.5924	1015.5914	1.06	0	46	0.0012	1	R.TIGLETVGVK.I
☑	201	521.7644	1041.5142	1041.5131	1.11	0	35	0.046	1	K.VVYENAYGK.F
☑	363	387.2245	1158.6517	1158.6509	0.64	0	(24)	0.19	1	R.FLIATGERPR.Y
☑	364	580.3334	1158.6522	1158.6509	1.14	0	37	0.0094	1	R.FLIATGERPR.Y
☑	382	585.8099	1169.6052	1169.6040	1.04	0	72	3.8e-006	1	K.IEQIEAGTPGR.L
☑	383	585.8120	1169.6094	1169.6080	1.20	1	56	0.00014	1	K.KVVYENAYGK.F
☑	478	630.8226	1259.6306	1259.6292	1.15	1	39	0.014	1	K.VICNLKDNER.V + Carbamidomethyl (C)
☑	479	630.8228	1259.6310	1259.6292	1.47	1	(38)	0.019	1	K.VICNLKDNER.V + Carbamidomethyl (C)
☑	689	513.3013	1536.8821	1536.8810	0.71	1	14	0.82	1	K.KLMHQAALLGQALK.D + Oxidation (M)
☑	967	624.3395	1869.9967	1869.9949	0.97	1	18	0.97	1	R.QFVPTKIEQIEAGTPGR.L
☑	1030	963.0052	1923.9958	1923.9942	0.86	0	56	0.00015	1	K.SYDFDLIIIGGGSGGLAAAK.E
☑	1281	775.3817	2323.1233	2323.1179	2.30	0	(45)	0.0037	1	K.STNSEETIEDEFNTVLLAVGR.D
☑	1282	1162.5700	2323.1254	2323.1179	3.23	0	111	9.1e-010	1	K.STNSEETIEDEFNTVLLAVGR.D
☑	1491	998.8046	2993.3920	2993.3827	3.10	1	30	0.14	1	R.YLGIPGDKEYCISSDDLFSLPYCPGK.T + 2 Carbamidomethyl (C)

3. APOH_CANFA **Mass:** 38378 **Score:** 275 **Queries matched:** 22 **emPAI:** 0.64

Beta-2-glycoprotein 1 OS=Canis familiaris GN=APOH PE=2 SV=1

☐ Check to include this hit in error tolerant search or archive report

	Query	Observed	Mr(expt)	Mr(calc)	ppm	Miss	Score	Expect	Rank	Peptide
	30	396.7346	791.4546	791.4541	0.67	1	30	0.095	4	K.LQEKFK.D
☑	34	**402.1918**	**802.3690**	**802.3684**	**0.85**	**0**	**7**	**8.3**	**1**	**K.VSFYCK.N + Carbamidomethyl (C)**
	115	468.2112	934.4078	934.4066	1.33	0	(40)	0.0022	1	K.TDASDVKPC.-
	142	496.7216	991.4286	991.4281	0.58	0	59	3.3e-005	1	K.TDASDVKPC.- + Carbamidomethyl (C)
	143	496.7216	991.4286	991.4281	0.58	0	(35)	0.0083	1	K.TDASDVKPC.- + Carbamidomethyl (C)
	144	496.7218	991.4290	991.4281	0.99	0	(46)	0.00061	1	K.TDASDVKPC.- + Carbamidomethyl (C)
	300	368.8542	1103.5408	1103.5400	0.72	0	(18)	2.2	1	K.EHSSLAFWK.T
	301	552.7780	1103.5414	1103.5400	1.33	0	20	1.3	1	K.EHSSLAFWK.T
	622	501.5981	1501.7725	1501.7711	0.89	0	(42)	0.0054	1	R.VCPFAGILENGAVR.Y + Carbamidomethyl (C)
	623	751.8937	1501.7728	1501.7711	1.14	0	(32)	0.061	1	R.VCPFAGILENGAVR.Y + Carbamidomethyl (C)
	624	751.8938	1501.7730	1501.7711	1.28	0	(42)	0.0052	1	R.VCPFAGILENGAVR.Y + Carbamidomethyl (C)
	625	751.8939	1501.7732	1501.7711	1.41	0	63	4e-005	1	R.VCPFAGILENGAVR.Y + Carbamidomethyl (C)
	626	751.8940	1501.7734	1501.7711	1.54	0	(24)	0.35	1	R.VCPFAGILENGAVR.Y + Carbamidomethyl (C)
	627	751.8942	1501.7738	1501.7711	1.81	0	(45)	0.0028	1	R.VCPFAGILENGAVR.Y + Carbamidomethyl (C)
	628	751.8946	1501.7746	1501.7711	2.34	0	(32)	0.06	1	R.VCPFAGILENGAVR.Y + Carbamidomethyl (C)
	629	751.8948	1501.7750	1501.7711	2.61	0	(26)	0.25	1	R.VCPFAGILENGAVR.Y + Carbamidomethyl (C)
	630	751.8949	1501.7752	1501.7711	2.74	0	(44)	0.0039	1	R.VCPFAGILENGAVR.Y + Carbamidomethyl (C)
	631	751.8951	1501.7756	1501.7711	3.01	0	(16)	2.6	1	R.VCPFAGILENGAVR.Y + Carbamidomethyl (C)
	632	751.8956	1501.7766	1501.7711	3.67	0	(38)	0.014	1	R.VCPFAGILENGAVR.Y + Carbamidomethyl (C)
	693	513.9190	1538.7352	1538.7340	0.76	1	(19)	1.1	1	K.CFKEHSSLAFWK.T + Carbamidomethyl (C)
	694	770.3760	1538.7374	1538.7340	2.23	1	44	0.0032	1	K.CFKEHSSLAFWK.T + Carbamidomethyl (C)
☑	1093	**655.9820**	**1964.9242**	**1964.9203**	**1.96**	**0**	**13**	**5.6**	**1**	**K.CPFPSRPDNGFVNYPAK.Q + Carbamidomethyl (C)**

4. TRXR1_BOVIN **Mass:** 54737 **Score:** 244 **Queries matched:** 11 **emPAI:** 0.51

Thioredoxin reductase 1, cytoplasmic OS=Bos taurus GN=TXNRD1 PE=2 SV=3

☐ Check to include this hit in error tolerant search or archive report

Query	Observed	Mr(expt)	Mr(calc)	ppm	Miss	Score	Expect	Rank	Peptide

	Query	Observed	Mr(expt)	Mr(calc)	ppm	Miss	Score	Expect	Rank	Peptide
	46	412.7296	823.4446	823.4440	0.82	0	13	3.2	2	R.LYGGSTVK.C
	72	431.7374	861.4602	861.4596	0.74	0	19	1.5	1	R.YLGIPGDK.E
	363	387.2245	1158.6517	1158.6509	0.64	0	(24)	0.19	1	R.FLIATGERPR.Y
	364	580.3334	1158.6522	1158.6509	1.14	0	37	0.0094	1	R.FLIATGERPR.Y
	478	630.8226	1259.6306	1259.6292	1.15	1	(25)	0.4	2	K.VVCNIKDNER.V + Acrylamide (C)
	479	630.8228	1259.6310	1259.6292	1.47	1	26	0.29	2	K.VVCNIKDNER.V + Acrylamide (C)
☑	765	809.4000	1616.7854	1616.7804	3.15	0	85	2.9e-007	1	R.WGLGGTCVNVGCIPK.K + 2 Carbamidomethyl (C)
☑	782	823.4550	1644.8954	1644.8909	2.74	0	79	7.8e-007	1	K.VMVLDFVTPTPLGTR.W
☑	803	831.4523	1660.8900	1660.8859	2.52	0	(76)	1.7e-006	1	K.VMVLDFVTPTPLGTR.W + Oxidation (M)
☑	1270	761.4160	2281.2262	2281.2219	1.86	0	56	9.1e-005	1	R.VVGFHVLGPNAGEVTQGFAAALK.C
	1491	998.8046	2993.3920	2993.3827	3.10	1	30	0.14	1	R.YLGIPGDKEYCISSDDLFSLPYCPGK.T + 2 Carbamidomethyl (C)

5. K2C1_HUMAN **Mass:** 65978 **Score:** 231 **Queries matched:** 11 **emPAI:** 0.55
Keratin, type II cytoskeletal 1 OS=Homo sapiens GN=KRT1 PE=1 SV=5
☐ Check to include this hit in error tolerant search or archive report

	Query	Observed	Mr(expt)	Mr(calc)	ppm	Miss	Score	Expect	Rank	Peptide
☑	84	437.7536	873.4926	873.4920	0.77	0	32	0.073	1	R.SLVNLGGSK.S
☑	165	508.2243	1014.4340	1014.4328	1.21	0	33	0.0073	1	K.DVDGAYMTK.V + Oxidation (M)
☑	197	517.2622	1032.5098	1032.5087	1.08	0	31	0.057	1	R.TLLEGEESR.M
☑	395	590.3044	1178.5942	1178.5931	0.95	0	65	2.9e-005	1	K.YEELQITAGR.H
☑	506	651.8624	1301.7102	1301.7078	1.86	0	89	1e-007	1	R.SLDLDSIIAEVK.A
☑	548	465.2492	1392.7258	1392.7249	0.65	1	(21)	0.67	1	R.TNAENEFVTIKK.D
☑	549	697.3717	1392.7288	1392.7249	2.86	1	52	0.00049	1	R.TNAENEFVTIKK.D
☑	593	738.3974	1474.7802	1474.7780	1.53	0	61	6e-005	1	R.FLEQQNQVLQTK.W
☑	772	546.9587	1637.8543	1637.8525	1.06	1	(23)	0.39	1	K.SLNNQFASFIDKVR.F
☑	773	546.9588	1637.8546	1637.8525	1.24	1	45	0.0021	1	K.SLNNQFASFIDKVR.F
☑	1125	665.3309	1992.9709	1992.9693	0.77	0	41	0.0093	1	R.THNLEPYFESFINNLR.R

Proteins matching the same set of peptides:
K2C1_PANTR **Mass:** 65450 **Score:** 231 **Queries matched:** 11
Keratin, type II cytoskeletal 1 OS=Pan troglodytes GN=KRT1 PE=2 SV=1

6. K22E_HUMAN **Mass:** 65825 **Score:** 179 **Queries matched:** 7 **emPAI:** 0.28
Keratin, type II cytoskeletal 2 epidermal OS=Homo sapiens GN=KRT2 PE=1 SV=1
☐ Check to include this hit in error tolerant search or archive report

	Query	Observed	Mr(expt)	Mr(calc)	ppm	Miss	Score	Expect	Rank	Peptide
☑	52	416.2506	830.4866	830.4862	0.57	0	9	15	1	R.SLVGLGGTK.S
☑	307	554.2757	1106.5368	1106.5356	1.13	0	45	0.0028	1	K.AQYEEIAQR.S
☑	327	566.2594	1130.5042	1130.5026	1.44	0	11	5.2	1	R.STSSFSCLSR.H + Carbamidomethyl (C)
☑	473	627.8083	1253.6020	1253.6001	1.59	0	83	4.2e-007	1	R.GFSSGSAVVSGGSR.R
☑	519	440.8661	1319.5765	1319.5756	0.66	0	46	0.0013	1	R.HGGGGGGFGGGGFGSR.S
☑	523	665.3679	1328.7212	1328.7187	1.90	0	64	2.5e-005	1	R.NLDLDSIIAEVK.A
	593	738.3974	1474.7802	1474.7780	1.53	0	61	6e-005	1	R.FLEQQNQVLQTK.W

7. THIO_HUMAN **Mass:** 11730 **Score:** 144 **Queries matched:** 9 **emPAI:** 2.60
Thioredoxin OS=Homo sapiens GN=TXN PE=1 SV=3
☐ Check to include this hit in error tolerant search or archive report

	Query	Observed	Mr(expt)	Mr(calc)	ppm	Miss	Score	Expect	Rank	Peptide
☑	97	454.7276	907.4406	907.4399	0.78	0	46	0.0027	1	K.VGEFSGANK.E
☑	155	501.2797	1000.5448	1000.5441	0.80	0	38	0.011	1	K.LEATINELV.-
☑	376	583.2965	1164.5784	1164.5775	0.83	1	30	0.08	1	K.VGEFSGANKE.L
☑	446	611.2755	1220.5364	1220.5359	0.48	0	18	1.4	1	K.CMPTFQFFK.K + Carbamidomethyl (C); Oxidation (M)
☑	477	629.8486	1257.6826	1257.6816	0.84	1	59	0.00011	1	K.EKLEATINELV.-
☑	526	668.8237	1335.6328	1335.6306	1.65	0	79	1.1e-006	1	K.TAFQEALDAAGDK.L

	Query	Observed	Mr(expt)	Mr(calc)	ppm	Miss	Score	Expect	Rank	Peptide
	596	370.6974	1478.7605	1478.7592	0.91	0	(9)	9.4	2	K.MIKPFFHSLSEK.Y + Oxidation (M)
☑	597	493.9275	1478.7607	1478.7592	1.03	0	25	0.24	1	K.MIKPFFHSLSEK.Y + Oxidation (M)
☑	828	852.3692	1702.7238	1702.7194	2.63	0	(16)	1.7	1	K.CMPTFQFFK.K + Maleimide-Invitrogen-Biotin+0 (C); Oxidation (M)

8. LACB_BOVIN **Mass:** 19870 **Score:** 137 **Queries matched:** 9 **emPAI:** 1.99
Beta-lactoglobulin OS=Bos taurus GN=LGB PE=1 SV=3
☐ Check to include this hit in error tolerant search or archive report

	Query	Observed	Mr(expt)	Mr(calc)	ppm	Miss	Score	Expect	Rank	Peptide
☑	68	427.2397	852.4648	852.4640	1.03	0	15	0.8	1	K.ALPMHIR.L + Oxidation (M)
☑	94	452.2870	902.5594	902.5589	0.58	1	24	0.069	1	K.TKIPAVFK.I
☑	103	458.7407	915.4668	915.4661	0.79	0	38	0.011	1	K.IDALNENK.V
☑	407	398.5643	1192.6711	1192.6703	0.62	1	44	0.00096	1	K.VLVLDTDYKK.Y
☑	408	597.3430	1192.6714	1192.6703	0.93	1	(41)	0.0022	1	K.VLVLDTDYKK.Y
☑	467	623.2968	1244.5790	1244.5772	1.48	0	53	0.00034	1	R.TPEVDDEALEK.F
☑	841	858.4081	1714.8016	1714.7985	1.86	0	63	4.7e-005	1	R.LSFNPTQLEEQCHI.- + Carbamidomethyl (C)
☑	1279	771.7596	2312.2570	2312.2515	2.37	0	29	0.041	1	R.VYVEELKPTPEGDLEILLQK.W
☑	1416	908.4643	2722.3711	2722.3636	2.75	0	35	0.029	1	K.VAGTWYSLAMAASDISLLDAQSAPLR.V + Oxidation (M)

9. K1C10_HUMAN **Mass:** 59475 **Score:** 126 **Queries matched:** 14 **emPAI:** 0.46
Keratin, type I cytoskeletal 10 OS=Homo sapiens GN=KRT10 PE=1 SV=4
☐ Check to include this hit in error tolerant search or archive report

	Query	Observed	Mr(expt)	Mr(calc)	ppm	Miss	Score	Expect	Rank	Peptide
☑	38	404.2037	806.3928	806.3923	0.73	0	29	0.2	1	R.LAADDFR.L
☑	160	502.2805	1002.5464	1002.5458	0.67	1	11	6.3	1	K.SEITELRR.N
☑	252	355.5417	1063.6033	1063.6026	0.66	1	19	0.41	1	R.LASYLDKVR.A
☑	285	545.7698	1089.5250	1089.5237	1.25	0	48	0.0021	1	K.VTMQNLNDR.L
☑	304	553.7668	1105.5190	1105.5186	0.41	0	(27)	0.15	1	K.VTMQNLNDR.L + Oxidation (M)
☑	305	553.7670	1105.5194	1105.5186	0.77	0	(44)	0.0032	1	K.VTMQNLNDR.L + Oxidation (M)
☑	457	412.2313	1233.6721	1233.6717	0.31	1	31	0.054	1	R.LKYENEVALR.Q
☑	458	617.8440	1233.6734	1233.6717	1.42	1	(20)	0.58	1	R.LKYENEVALR.Q
☑	481	631.8030	1261.5914	1261.5899	1.26	0	62	6.3e-005	1	R.SLLEGEGSSGGGGR.G
☑	504	434.2036	1299.5890	1299.5877	0.97	1	19	1.3	1	K.NHEEEMKDLR.N
☑	518	439.5352	1315.5838	1315.5826	0.87	1	(9)	11	1	K.NHEEEMKDLR.N + Oxidation (M)
☑	543	691.3287	1380.6428	1380.6408	1.46	0	57	0.00017	1	R.ALEESNYELEGK.I
☑	611	498.5834	1492.7284	1492.7270	0.94	1	(26)	0.23	1	R.SQYEQLAEQNRK.D
☑	612	747.3719	1492.7292	1492.7270	1.52	1	33	0.049	1	R.SQYEQLAEQNRK.D

10. TRYP_PIG **Mass:** 24394 **Score:** 93 **Queries matched:** 3 **emPAI:** 0.29
Trypsin OS=Sus scrofa PE=1 SV=1
☐ Check to include this hit in error tolerant search or archive report

	Query	Observed	Mr(expt)	Mr(calc)	ppm	Miss	Score	Expect	Rank	Peptide
☑	60	421.7586	841.5026	841.5022	0.59	0	(50)	0.00082	1	R.VATVSLPR.S
☑	61	421.7586	841.5026	841.5022	0.59	0	56	0.00023	1	R.VATVSLPR.S
☑	1256	737.7076	2210.1010	2210.0967	1.92	0	52	0.00065	1	R.LGEHNIDVLEGNEQFINAAK.I

11. K1C9_HUMAN **Mass:** 62092 **Score:** 80 **Queries matched:** 2 **emPAI:** 0.11
Keratin, type I cytoskeletal 9 OS=Homo sapiens GN=KRT9 PE=1 SV=2
☐ Check to include this hit in error tolerant search or archive report

	Query	Observed	Mr(expt)	Mr(calc)	ppm	Miss	Score	Expect	Rank	Peptide
☑	253	533.2538	1064.4930	1064.4920	0.95	0	28	0.094	1	K.STMQELNSR.L
☑	459	618.2687	1234.5228	1234.5215	1.13	0	77	9.2e-007	1	R.FSSSSGYGGGSSR.V

12. CASB_BOVIN **Mass:** 25091 **Score:** 79 **Queries matched:** 4 **emPAI:** 0.28
Beta-casein OS=Bos taurus GN=CSN2 PE=1 SV=2
☐ Check to include this hit in error tolerant search or archive report

	Query	Observed	Mr(expt)	Mr(calc)	ppm	Miss	Score	Expect	Rank	Peptide
☑	18	390.7528	779.4910	779.4905	0.67	0	8	0.63	1	K.VLPVPQK.A
	50	415.7297	829.4448	829.4446	0.27	0	4	58	10	K.AVPYPQR.D
☑	1252	734.7276	2201.1610	2201.1555	2.51	0	(37)	0.014	1	R.DMPIQAFLLYQEPVLGPVR.G + Oxidation (M)
☑	1253	1101.5890	2201.1634	2201.1555	3.63	0	73	3e-006	1	R.DMPIQAFLLYQEPVLGPVR.G + Oxidation (M)

Proteins matching the same set of peptides:
CASB_BUBBU **Mass:** 25090 **Score:** 79 **Queries matched:** 4
Beta-casein OS=Bubalus bubalis GN=CSN2 PE=2 SV=1

13. LACB_OVIMU **Mass:** 18139 **Score:** 75 **Queries matched:** 6 **emPAI:** 0.67
Beta-lactoglobulin OS=Ovis orientalis musimon GN=LGB PE=1 SV=1
☐ Check to include this hit in error tolerant search or archive report

	Query	Observed	Mr(expt)	Mr(calc)	ppm	Miss	Score	Expect	Rank	Peptide
	68	427.2397	852.4648	852.4640	1.03	0	15	0.8	1	K.ALPMHIR.L + Oxidation (M)
	94	452.2870	902.5594	902.5589	0.58	1	24	0.069	1	K.TKIPAVFK.I
	103	458.7407	915.4668	915.4661	0.79	0	38	0.011	1	K.IDALNENK.V
☑	121	475.2734	948.5322	948.5314	0.89	0	11	6.1	1	-.IIVTQTMK.G + Oxidation (M)
	407	398.5643	1192.6711	1192.6703	0.62	1	44	0.00096	1	K.VLVLDTDYKK.Y
	408	597.3430	1192.6714	1192.6703	0.93	1	(41)	0.0022	1	K.VLVLDTDYKK.Y

14. IPSP_HUMAN **Mass:** 45673 **Score:** 74 **Queries matched:** 3 **emPAI:** 0.23
Plasma serine protease inhibitor OS=Homo sapiens GN=SERPINA5 PE=1 SV=2
☐ Check to include this hit in error tolerant search or archive report

	Query	Observed	Mr(expt)	Mr(calc)	ppm	Miss	Score	Expect	Rank	Peptide
☑	365	581.2914	1160.5682	1160.5673	0.78	0	52	0.00054	1	K.AVVEVDESGTR.A
☑	451	613.8313	1225.6480	1225.6455	2.06	0	43	0.0028	1	R.AAAATGTIFTFR.S
☑	495	639.8046	1277.5946	1277.5921	1.96	0	28	0.14	1	K.MQQVENGLSEK.T + Oxidation (M)

15. THIO_ECOLI **Mass:** 11799 **Score:** 60 **Queries matched:** 3 **emPAI:** 0.67
Thioredoxin-1 OS=Escherichia coli (strain K12) GN=trxA PE=1 SV=2
☐ Check to include this hit in error tolerant search or archive report

	Query	Observed	Mr(expt)	Mr(calc)	ppm	Miss	Score	Expect	Rank	Peptide
☑	157	501.3241	1000.6336	1000.6321	1.55	0	23	0.042	1	R.GIPTLLLFK.N
☑	486	634.3364	1266.6582	1266.6568	1.14	0	60	7.2e-005	1	K.LNIDQNPGTAPK.Y
☑	1284	778.7484	2333.2234	2333.2188	1.95	1	4	25	1	K.MIAPILDEIADEYQGKLTVAK.L + Oxidation (M)

Proteins matching the same set of peptides:
THIO_SALTI **Mass:** 11799 **Score:** 60 **Queries matched:** 3
Thioredoxin-1 OS=Salmonella typhi GN=trxA PE=3 SV=2
THIO_SALTY **Mass:** 11799 **Score:** 60 **Queries matched:** 3
Thioredoxin-1 OS=Salmonella typhimurium GN=trxA PE=3 SV=2
THIO_SHIFL **Mass:** 11799 **Score:** 60 **Queries matched:** 3
Thioredoxin-1 OS=Shigella flexneri GN=trxA PE=3 SV=2
THIO_ECOL6 **Mass:** 11799 **Score:** 60 **Queries matched:** 3
Thioredoxin-1 OS=Escherichia coli O6 GN=trxA PE=3 SV=2
THIO_ECO57 **Mass:** 11799 **Score:** 60 **Queries matched:** 3

Thioredoxin-1 OS=Escherichia coli O157:H7 GN=trxA PE=3 SV=2

16. PROP_HUMAN **Mass:** 51242 **Score:** 54 **Queries matched:** 11 **emPAI:** 0.28
Properdin OS=Homo sapiens GN=CFP PE=1 SV=2
☐ Check to include this hit in error tolerant search or archive report

	Query	Observed	Mr(expt)	Mr(calc)	ppm	Miss	Score	Expect	Rank	Peptide
☑	2	358.7132	715.4118	715.4116	0.36	0	25	0.33	1	K.LVVEEK.R
☑	116	471.2785	940.5424	940.5416	0.94	0	22	0.23	1	R.LCTPLLPK.Y + Carbamidomethyl (C)
	199	517.7271	1033.4396	1033.4433	-3.58	0	3	15	3	R.SGGLCQPCR.S + 2 Carbamidomethyl (C)
☑	398	397.5222	1189.5448	1189.5444	0.27	1	16	1.9	1	K.RSGGLCQPCR.S + 2 Carbamidomethyl (C)
☑	472	626.7639	1251.5132	1251.5125	0.60	0	28	0.06	1	R.CVGWNGQCSGK.V + 2 Carbamidomethyl (C)
☑	572	481.5608	1441.6606	1441.6595	0.76	0	24	0.25	1	R.HCYSIQHCPLK.G + 2 Carbamidomethyl (C)
☑	610	745.3582	1488.7018	1488.6991	1.86	0	42	0.0088	1	K.SISCQEIPGQQSR.G + Carbamidomethyl (C)
☑	1165	693.6603	2077.9591	2077.9561	1.44	1	22	0.85	1	K.RPCLHVPACKDPEEEEL.- + 2 Carbamidomethyl (C)
☑	1364	639.3106	2553.2133	2553.2104	1.13	0	2	82	1	K.CSAPEPSQKPPGKPCPGLAYEQR.R + 2 Carbamidomethyl (C)
☑	1397	671.3347	2681.3097	2681.3054	1.62	1	15	4.3	1	R.KCSAPEPSQKPPGKPCPGLAYEQR.R + 2 Carbamidomethyl (C)
☑	1398	671.3348	2681.3101	2681.3054	1.77	1	(12)	7.7	1	R.KCSAPEPSQKPPGKPCPGLAYEQR.R + 2 Carbamidomethyl (C)

17. APOH_RAT **Mass:** 33175 **Score:** 40 **Queries matched:** 5 **emPAI:** 0.21
Beta-2-glycoprotein 1 OS=Rattus norvegicus GN=Apoh PE=2 SV=2
☐ Check to include this hit in error tolerant search or archive report

	Query	Observed	Mr(expt)	Mr(calc)	ppm	Miss	Score	Expect	Rank	Peptide
	300	368.8542	1103.5408	1103.5400	0.72	0	(18)	2.2	1	K.EHSSLAFWK.T
	301	552.7780	1103.5414	1103.5400	1.33	0	20	1.3	1	K.EHSSLAFWK.T
☑	498	645.7984	1289.5822	1289.5809	1.03	0	14	3.2	1	K.LDGPEEVECTK.T + Acrylamide (C)
	693	513.9190	1538.7352	1538.7340	0.76	1	(19)	1.1	1	K.CFKEHSSLAFWK.T + Carbamidomethyl (C)
	694	770.3760	1538.7374	1538.7340	2.23	1	44	0.0032	1	K.CFKEHSSLAFWK.T + Carbamidomethyl (C)

18. IGHG1_HUMAN **Mass:** 36083 **Score:** 40 **Queries matched:** 3 **emPAI:** 0.09
Ig gamma-1 chain C region OS=Homo sapiens GN=IGHG1 PE=1 SV=1
☐ Check to include this hit in error tolerant search or archive report

	Query	Observed	Mr(expt)	Mr(calc)	ppm	Miss	Score	Expect	Rank	Peptide
☑	66	426.2184	850.4222	850.4218	0.48	0	27	0.2	1	K.DTLMISR.T + Oxidation (M)
	67	426.2185	850.4224	850.4218	0.71	0	(14)	3.7	2	K.DTLMISR.T + Oxidation (M)
☑	968	937.4686	1872.9226	1872.9146	4.32	0	40	0.011	1	K.TTPPVLDSDGSFFLYSK.L

19. CASA1_BOVIN **Mass:** 24513 **Score:** 40 **Queries matched:** 2 **emPAI:** 0.14
Alpha-S1-casein OS=Bos taurus GN=CSN1S1 PE=1 SV=2
☐ Check to include this hit in error tolerant search or archive report

	Query	Observed	Mr(expt)	Mr(calc)	ppm	Miss	Score	Expect	Rank	Peptide
☑	544	692.8699	1383.7252	1383.7227	1.82	0	40	0.0075	1	R.FFVAPFPEVFGK.E
☑	777	547.9616	1640.8630	1640.8603	1.64	1	20	0.66	1	R.FFVAPFPEVFGKEK.V

Proteins matching the same set of peptides:
CASA1_BUBBU **Mass:** 24311 **Score:** 40 **Queries matched:** 2
Alpha-S1-casein OS=Bubalus bubalis GN=CSN1S1 PE=2 SV=2

20. CASK_BOVIN **Mass:** 21256 **Score:** 37 **Queries matched:** 1 **emPAI:** 0.16
Kappa-casein OS=Bos taurus GN=CSN3 PE=1 SV=1
☐ Check to include this hit in error tolerant search or archive report

Query	Observed	Mr(expt)	Mr(calc)	ppm	Miss	Score	Expect	Rank	Peptide

471	626.3596	1250.7046	1250.7023	1.89	0	37	0.0081	1	K.YIPIQYVLSR.Y

Proteins matching the same set of peptides:

CASK_BUBBU **Mass:** 21384 **Score:** 37 **Queries matched:** 1
Kappa-casein OS=Bubalus bubalis GN=CSN3 PE=1 SV=2
CASK_CAPCR **Mass:** 21473 **Score:** 37 **Queries matched:** 1
Kappa-casein OS=Capricornis crispus GN=CSN3 PE=2 SV=1
CASK_CAPHI **Mass:** 21428 **Score:** 37 **Queries matched:** 1
Kappa-casein OS=Capra hircus GN=CSN3 PE=1 SV=2
CASK_CAPSU **Mass:** 21500 **Score:** 37 **Queries matched:** 1
Kappa-casein OS=Capricornis sumatrensis GN=CSN3 PE=2 SV=1
CASK_CAPSW **Mass:** 21563 **Score:** 37 **Queries matched:** 1
Kappa-casein OS=Capricornis swinhoei GN=CSN3 PE=2 SV=1
CASK_GIRCA **Mass:** 17085 **Score:** 37 **Queries matched:** 1
Kappa-casein (Fragment) OS=Giraffa camelopardalis GN=CSN3 PE=2 SV=1
CASK_NEMGO **Mass:** 21472 **Score:** 37 **Queries matched:** 1
Kappa-casein OS=Nemorhaedus goral GN=CSN3 PE=2 SV=1
CASK_OREAM **Mass:** 21512 **Score:** 37 **Queries matched:** 1
Kappa-casein OS=Oreamnos americanus GN=CSN3 PE=2 SV=1
CASK_RUPRU **Mass:** 21429 **Score:** 37 **Queries matched:** 1
Kappa-casein OS=Rupicapra rupicapra GN=CSN3 PE=2 SV=1
CASK_SAITA **Mass:** 22641 **Score:** 37 **Queries matched:** 1
Kappa-casein OS=Saiga tatarica GN=CSN3 PE=2 SV=1
CASK_SHEEP **Mass:** 21425 **Score:** 37 **Queries matched:** 1
Kappa-casein OS=Ovis aries GN=CSN3 PE=1 SV=2

21. HAS2_XENLA **Mass:** 63643 **Score:** 36 **Queries matched:** 1 **emPAI:** 0.05
Hyaluronan synthase 2 OS=Xenopus laevis GN=has2 PE=2 SV=2
Check to include this hit in error tolerant search or archive report

Query	Observed	Mr(expt)	Mr(calc)	ppm	Miss	Score	Expect	Rank	Peptide
57	419.7270	837.4394	837.4344	5.97	0	36	0.022	1	K.SSFASALR.G

22. Y1720_HAEIN **Mass:** 21734 **Score:** 32 **Queries matched:** 1 **emPAI:** 0.15
Uncharacterized protein HI1720 OS=Haemophilus influenzae GN=HI1720 PE=3 SV=1
Check to include this hit in error tolerant search or archive report

Query	Observed	Mr(expt)	Mr(calc)	ppm	Miss	Score	Expect	Rank	Peptide
678	765.8925	1529.7704	1529.7685	1.28	1	32	0.065	1	K.TREEELELENLR.L

Peptide matches not assigned to protein hits: (no details means no match)

Query	Observed	Mr(expt)	Mr(calc)	ppm	Miss	Score	Expect	Rank	Peptide
16	386.7321	771.4496	771.4490	0.79	1	33	0.057	1	GPTLKEK
30	396.7346	791.4546	791.4541	0.67	1	31	0.067	1	IKQEFK
80	436.2542	870.4938	870.4923	1.80	0	31	0.076	1	ILENNIR
145	497.2138	992.4130	992.4121	0.98	0	29	0.076	1	TDASELTPC + Carbamidomethyl (C)
106	464.7683	927.5220	927.5178	4.57	1	29	0.087	1	DGKVWPVK
634	501.9262	1502.7568	1502.7585	-1.16	0	28	0.16	1	EVCDQLVQMLVR + Acrylamide (C)
585	727.8882	1453.7618	1453.7565	3.68	0	28	0.12	1	IIEINPEEGWVR
605	495.9225	1484.7457	1484.7372	5.73	0	28	0.14	1	QHSYDNILDKPR
10	372.2188	742.4230	742.4225	0.76	0	27	0.11	1	ASLDLPK
747	787.8871	1573.7596	1573.7518	4.96	1	27	0.21	1	LDRMEGDLQDQVR
65	425.2455	848.4764	848.4756	1.03	1	26	0.2	1	IKEANFK
314	559.7870	1117.5594	1117.5550	3.96	0	26	0.22	1	MQGGLGVGGTGGK
533	676.9014	1351.7882	1351.7936	-3.93	1	26	0.052	1	LPTGLGELLRQR
315	560.2796	1118.5446	1118.5390	5.08	0	26	0.32	1	ACEEAGILSR + Acrylamide (C)

464	622.3409	1242.6672	1242.6680	-0.62	1	25	0.22	1	VESQAIRQANK
132	486.7630	971.5114	971.5110	0.48	0	25	0.21	1	QLMQLNPL + Oxidation (M)
228	529.7384	1057.4622	1057.4611	1.12	0	25	0.47	1	ESANCHEIR
633	501.9262	1502.7568	1502.7585	-1.16	0	24	0.38	1	EVCDQLVQMLVR + Acrylamide (C)
728	780.8969	1559.7792	1559.7726	4.26	0	24	0.41	1	TVVCVISGGNNDINR
177	511.2896	1020.5646	1020.5604	4.20	1	24	0.32	1	AGAIASFEKK
105	464.7645	927.5144	927.5138	0.72	0	23	0.25	1	IGAQIDGVR
153	500.7751	999.5356	999.5349	0.75	0	23	0.29	1	AGLLAGDDLR
273	543.8031	1085.5916	1085.5968	-4.76	0	23	0.37	1	EVELVELAGK
635	752.3863	1502.7580	1502.7664	-5.53	1	23	0.54	1	ICETAKHFLASAR + Carbamidomethyl (C)
380	584.7701	1167.5256	1167.5230	2.25	0	23	0.28	1	CTDGLQGFEK + Acrylamide (C)
56	417.7400	833.4654	833.4680	-3.12	1	23	0.55	1	IKEGMIK + Oxidation (M)
556	704.8658	1407.7170	1407.7180	-0.68	1	23	0.51	1	MEIFLNKENVR + Oxidation (M)
440	608.3537	1214.6928	1214.6870	4.79	1	22	0.35	1	LDKVISELNGK
405	596.8203	1191.6260	1191.6248	1.07	0	22	0.5	1	GALEAYVQSVR
150	500.2831	998.5516	998.5509	0.76	0	22	0.29	1	GALIQDVQR
490	635.3521	1268.6896	1268.6911	-1.13	0	22	0.34	1	IVGELMQPAGVR
404	596.8203	1191.6260	1191.6248	1.07	0	22	0.56	1	GALEAYVQSVR
541	689.3145	1376.6144	1376.6183	-2.80	0	22	0.69	1	AEDSWHACFLK + Acrylamide (C)
217	525.7648	1049.5150	1049.5110	3.86	1	22	0.66	1	IGEVRMCR + Acrylamide (C); Oxidation (M)
704	515.6018	1543.7836	1543.7776	3.84	1	21	0.64	1	AAMAAVEEVDRGLGR
36	403.7299	805.4452	805.4446	0.77	0	21	0.92	1	KPSVGYR
45	410.7321	819.4496	819.4524	-3.35	1	21	0.94	1	IKECLSK
257	359.5538	1075.6396	1075.6389	0.58	1	21	0.18	1	INIKNLSFK
12	382.1816	762.3486	762.3483	0.46	0	21	0.58	1	GCPFSPR
613	747.3760	1492.7374	1492.7463	-5.93	0	21	0.8	1	YPVGGIFHGYEVR
370	388.5223	1162.5451	1162.5441	0.85	0	21	0.78	1	QMLNFHTEK + Oxidation (M)
93	452.2398	902.4650	902.4644	0.73	0	20	0.87	1	DGGLLLCR + Carbamidomethyl (C)
100	457.7605	913.5064	913.5055	1.03	1	20	0.88	1	APQKMPVK + Oxidation (M)
262	540.2511	1078.4876	1078.4900	-2.14	0	20	0.77	1	SCLCPTVSR + 2 Carbamidomethyl (C)
69	428.7664	855.5182	855.5178	0.53	0	20	0.66	1	LASVLTPR
569	719.8901	1437.7656	1437.7729	-5.01	1	20	0.74	1	NTFFGVKQQITR
1	352.6901	703.3656	703.3687	-4.31	0	19	1.3	1	MIVADR
209	524.7751	1047.5356	1047.5309	4.58	1	19	1.2	1	NISSASKGER
561	714.3942	1426.7738	1426.7780	-2.89	0	19	0.77	1	VIDINNIDLTAAR
476	629.3336	1256.6526	1256.6547	-1.62	0	19	0.82	1	ASPMPSAAAALVR + Oxidation (M)
524	665.8381	1329.6616	1329.6565	3.89	0	19	0.92	1	DAGVLTHYETPK
684	768.3813	1534.7480	1534.7418	4.07	1	19	1.3	1	ACMNMGTLSGAPKVR
743	786.8974	1571.7802	1571.7726	4.89	1	19	1.3	1	SSVSCQKHAASAPTK + Acrylamide (C)
956	929.0043	1855.9940	1855.9978	-2.04	1	19	0.69	1	GFGQPQLAMILPLDGRK + Oxidation (M)
418	598.7650	1195.5154	1195.5148	0.55	0	19	0.51	1	CICVVESGCR + Acrylamide (C); Carbamidomethyl (C)
265	540.7704	1079.5262	1079.5256	0.60	0	19	1.1	1	SLGLGMCFPR
42	409.7423	817.4700	817.4731	-3.77	1	18	1.5	1	IKEGMIK
114	467.7354	933.4562	933.4516	5.03	0	18	1.6	1	AQSTSAQNK
128	483.2706	964.5266	964.5229	3.84	0	18	1	1	IIVDTYGGK
260	359.8820	1076.6242	1076.6230	1.10	1	18	0.58	1	KVVYNIDVK
745	787.3951	1572.7756	1572.7831	-4.73	1	18	1.4	1	CELLGDQLRWQR + Carbamidomethyl (C)
67	426.2185	850.4224	850.4218	0.73	1	18	1.4	1	ESSLCKK + Carbamidomethyl (C)
387	588.8231	1175.6316	1175.6299	1.53	0	18	1.3	1	KPSLVGQYER
297	550.8107	1099.6068	1099.6059	0.84	1	18	1.1	1	KQMPLNLEK
55	417.7399	833.4652	833.4647	0.67	0	18	1.8	1	AGIFGELK
263	540.2780	1078.5414	1078.5441	-2.46	0	17	1.3	1	GATVTICTSR + Acrylamide (C)
637	752.3864	1502.7582	1502.7664	-5.40	1	17	1.9	1	ICETAKHFLASAR + Carbamidomethyl (C)
445	610.8181	1219.6216	1219.6197	1.61	0	17	1.7	1	SNISSYVPTPR
925	908.9890	1815.9634	1815.9578	3.12	1	17	1.2	1	SRIEDITNVGTELIEK
239	531.7248	1061.4350	1061.4374	-2.20	0	17	0.58	1	NAGDDGEQTR
685	768.3817	1534.7488	1534.7418	4.59	1	17	2.1	1	ACMNMGTLSGAPKVR
44	410.2036	818.3926	818.3956	-3.63	0	17	1.9	1	SLMAEPR + Oxidation (M)
511	657.3465	1312.6784	1312.6847	-4.78	1	17	1.4	1	KANEIAGNNGIGR
