# Supplementary material for: Redox control of β2-glycoprotein I–von Willebrand factor interaction by thioredoxin-1
Source: J Thromb Haemost. 2010 Aug;8(8):1754–62. doi: 10.1111/j.1538-7836.2010.03944.x (PMC3017748; doi:10.1111/j.1538-7836.2010.03944.x)
Supplement: Supplementary file 4 [file jth0008-1754-SD4.doc]

**Supplementary Table 3.** MPB/IA labeled abundance ratios of cysteine containing peptides in nβ2GPI treated with TRX-1/TRX-R/NADPH/MPB.

|  |  |  | **Solvent Accessibilty (Å^2^)^#^** | **Retention time (Mins)** | | **Area (ion current^2^)** | |  |
| --- | --- | --- | --- | --- | --- | --- | --- | --- |
| **Peptide** | **Residues** | **Disulfide** |  | **IA (+57)** | **MPB (+557^)** | **IA (+57)** | **MPB (+557^)** | **Ratio (MPB/IA)** |
| T**C**PKPDDLPFSTVVPLK | 3-19 | 4--47 | 0 | 36.32 | 37.1 | 3965432 | 4016859 | 1.0130 |
| TFYEPGEEITYS**C**KPGYVSR | 20-39 | 32--60 | 33 | 32.62 | 32.92 | 13157518 | 21101547 | 1.6038 |
| FI**C**PLTGLW+16PINTLK | 45-59 | 4--47 | 1 | 41.7 | 40.82 | 1258484 | 1162234 | 0.9235 |
| V**C**PFAGILENGAVR | 64-77 | 65--105 | 4 | 37.65 | 37.55 | 29515771 | 32062704 | 1.0863 |
| **C**PFPSRPDNGFVNYPAKPTLYYK | 186-208 | 186--229 | 10 | 33.86 | 34.37 | 687255 | 456194 | 0.6638 |
| GPEEIE**C**TK | 223-231 | 186--229 | 1 | 24.38 | 27.11 | 153070 | 154157 | 1.0071 |
| NGM+16LHGDKVSFF**C**K | 269-282 | 281--306 | 1 | 29.84 | 30.92 | 758964 | 767426 | 1.0111 |
| K**C**SYTEDAQ**C**IDGTIEVPK | 287-305 | 288--326;245--296 | 4;0 | 31.97 | 33.07 | 823707 | 726976 | 0.8826 |
| **C**FKEHSSLAFWK | 306-317 | 281--306 | 31 | 32.54 | 34.01 | 2293252 | 523495 | 0.2283 |
| TDASDVKP**C** | 318-326 | 288-326 | 103 | 22.26 | 25.83 | 457060 | 34905072 | 76.3687 |

^#^ Solvent accessibility based on PDB structure 1QUB as calculated by DSSP

^ Typically the modification of cysteine by MPB results in a mass increase of +523.2101. In these experiments +557.2155 (+MPB+H_2_O_2_, m/z 34.0055) was found to be the primary Cys-MPB modification. Succinimidyl thioethers have the tendency to undergo spontaneous hydrolysis (adding H_2_O, m/z 18.0106) producing succinamic acid thioethers at pH > 8 [Suppl Information Ref 6] and thioethers may oxidise (adding O, m/z 15.9949) forming sulfoxides [Suppl Information Ref 7]. The complexity of the MPB-labeled products detected was attributed to these processes.
